# Diverse actions of 15 structurally unrelated mitochondrial uncouplers in cells and mice

**DOI:** 10.1016/j.molmet.2025.102204

**Published:** 2025-07-08

**Authors:** Divya P. Shah, Calum S. Vancuylenburg, Ellen M. Olzomer, Sing-Young Chen, Robert J. Grams, Martina Beretta, Frances L. Byrne, Webster L. Santos, Kyle L. Hoehn

**Affiliations:** 1 School of Biotechnology and Biomolecular Sciences, University of New South Wales, Sydney, NSW, 2052, Australia; 2 Faculty of Medicine, The University of British Columbia, Vancouver, BC, V6T 1Z4, Canada; 3 Department of Chemistry and Virginia Tech Centre for Drug Discovery, Virginia Tech, Blacksburg, VA, 24061, USA

**Keywords:** Mitochondrial uncoupling, Obesity, Metabolic disease, Diabetes

## Abstract

**Objective:**

Mitochondrial uncouplers are used as chemical tools to study mitochondrial function *in vitro* and *in vivo*, and some molecules are in development for the treatment of metabolic diseases. One problem in the field is that any molecule that increases proton transport into the mitochondrial matrix independent of ATP production can be classified as an uncoupler regardless of off-target activities. Therefore, there are dozens of classes of molecules that exhibit a wide spectrum of phenotypes. Herein we directly compared 15 mitochondrial uncouplers side-by-side in a well-defined cell system to better understand their *in vitro* dose response profiles and the top molecules with suitable pharmacology and safety profiles were compared in *db/db* mice.

**Methods:**

Fifteen mitochondrial uncouplers were characterised *in vitro* in CHO–K1 cells. The top five candidates were selected for further characterisation in male *db/db* mice based on their *in vitro* dose response and/or tolerability. We tested two doses of each mitochondrial uncoupler in mice and benchmarked their efficacy to a lifestyle intervention of 35% calorie restriction as well as to lean *db/+* metabolically healthy mice. Eleven groups of mice were fed *ad libitum* either; 1) chow (control), 2) chow with 0.15% BAM15 (w/w), 3) chow with 0.2% BAM15 (w/w), 4) chow with 0.1% NEN (w/w), 5) chow with 0.25% NEN (w/w), 6) chow with 0.01% OPC-163493 (w/w), 7) chow with 0.02% OPC-63493 (w/w), 8) chow with 0.015% ES9 (w/w), 9) chow with 0.03% ES9 (w/w), 10) chow with 0.2% NTZ (w/w), and 11) chow with 0.4% NTZ (w/w). Another group of mice was fed chow to receive ∼65% of the average daily food intake of control mice as a model of calorie restriction (CR). Mice were metabolically phenotyped over 4 weeks of treatment with assessment of key readouts including body weight, HbA1c, blood glucose and glucose tolerance tests. At termination, key tissues were collected and plasma was analysed for markers of toxicity.

**Results:**

Few mitochondrial uncouplers behaved similarly *in vitro*, with 11 molecules impairing maximal mitochondrial capacity. *In vivo*, BAM15 dose-dependently improved body weight and metabolic parameters in *db/db* mice, with 0.2% BAM15 treatment yielding statistically significant improvements in body weight, fat pad weight, glucose tolerance, blood glucose, HbA1c, liver weight and triglyceride content. The next-best treatment was 0.03% ES9 which significantly improved glucose tolerance, blood glucose levels, and HbA1c, but increased body weight, liver size and steatosis relative to *db/db* controls.

**Conclusions:**

Mitochondrial uncouplers BAM15 and ES9 had the greatest dose tolerance range *in vitro*, while BAM15 had the best overall effects on body weight, glucose control and liver steatosis in *db/db* mice. This study reveals diverse phenotypes across 15 classes of mitochondrial uncouplers and underscores the need for rigorous evaluation to identify molecules that drive stable mitochondrial respiration without unwanted mitochondrial inhibition or off-target effects. Ultimately, mitochondrial uncouplers should not be generalized and each uncoupler molecule needs to be considered by its own actions in well-defined experimental conditions.

## Highlights

• FCCP underestimates maximal mitochondrial respiration in most cell lines.
• BAM15 is a superior chemical probe to study mitochondrial function *in vitro.*
• Eleven mitochondrial uncouplers impair mitochondrial function with dose escalation.
• BAM15 and ES9 had the best *in vitro* activity profiles.
• BAM15 treatment resulted in the best improvement in metabolic phenotype in *db/db* mice.

## 1 Introduction

Mitochondria convert metabolites into ATP through a process that involves a proton cycle across the mitochondrial inner membrane. Specifically, metabolite oxidation in the mitochondrial matrix drives proton efflux via the electron transport chain complexes I, III, and IV to create an electrochemical gradient across the mitochondrial inner membrane such that ∼10-fold fewer protons are in the mitochondrial matrix than in the intermembrane space. The proton-motive force across the mitochondrial inner membrane drives proton re-entry into the matrix via ATP synthase to result in ATP production. Mitochondrial uncouplers transport protons from the mitochondrial intermembrane space into the mitochondrial matrix without ATP production, thus decreasing the coupling efficiency between metabolite oxidation and ATP production [1]. However, not all agents that make mitochondrial oxidative phosphorylation less efficient are uncouplers and we restrict our focus herein on small molecule mitochondrial uncoupler agents that have reported activity to increase proton transport into mitochondria independent of ATP synthase. Mitochondrial uncouplers can promote weight loss by increasing metabolite oxidation, and they create a more oxidized state of the mitochondrial electron transport chain (ETC) to have antioxidant effects [2]. There are dozens of known classes of mitochondrial uncouplers [1], but the relative superiority of one molecule over another remains poorly understood because few mitochondrial uncouplers have been compared head-to-head and compounds are studied across heterologous experimental systems using inconsistent methods. Therefore, in this study, we compared 15 of the most used mitochondrial uncouplers head-to-head *in vitro* and in the *db/db* mouse model of severe metabolic disease to determine the therapeutic potential of diverse classes of molecules.

The best characterised mitochondrial uncoupler is 2,4-dinitrophenol (DNP), which was incidentally discovered to induce weight loss in munitions workers during World War I [3]. Humans taking DNP experienced an 11% increase in metabolic rate for every 100 mg ingested and lost 1.5 kg of body weight per week [4,5]. However, despite the strong efficacy of DNP it has a narrow therapeutic window between efficacy and significant adverse side effects including hyperthermia, tachycardia, tachypnoea and cataracts that resulted in it being banned by the U.S. Food and Drug Administration (FDA) in 1938 [6]. DNP was never developed as a drug per se as it was used before the FDA existed and did not go through rigorous clinical trials. DNP contains phenolic nitro groups that are medicinal chemistry structural alerts [7]. Indeed, DNP metabolism leads to the formation of 2-amino-4-nitrophenol and 4-amino-2-nitrophenol, which are susceptible to oxidation to highly reactive iminoquinones [8]. Other well-known mitochondrial uncouplers like FCCP also have a therapeutic window that is too narrow for human use. Therefore, it remained unclear for decades whether the adverse effects of DNP and FCCP were due to on-target effects in mitochondria or due to off-target effects like plasma membrane depolarization [[9], [10], [11]]. However, in recent years it has been shown that DNP toxicity can be decreased by liver-targeting pro-drug and formulation approaches, suggesting DNP toxicity may be primarily due to drug exposure in key organs like muscle, eyes, and kidney [12,13]. Notably, newer mitochondrial uncoupler drugs have been developed in the past decade that have better therapeutic windows without need for pro-drug or formulation approaches [1,2]. The improved safety profiles of newer mitochondrial uncouplers have challenged the common misconception that all mitochondrial uncouplers are inherently dangerous and have revived interest in mitochondrial uncoupler therapeutic development.

Curiously, not all mitochondrial uncouplers cause weight loss in rodents, as summarized in Table 1. For example, **DNP** did not cause weight loss in obese mice housed at room temperature but did at thermoneutrality [14]. The novel uncoupler **OPC-163493** reduced hepatic lipids in *ob/ob* mice, improved glucose control in Zucker diabetic fatty rats, and had anti-diabetic effects in Akita mice but had no significant effect on body weight in any model [[15], [16], [17]]. In contrast, the uncoupler **BAM15** improved metabolic parameters and reversed adiposity in diet-induced obese C57BL/6 mice [18,19] and improved body composition and restored glucose tolerance in *db/db* mice housed at room temperature [20,21]. Dodecyl-triphenylphosphonium (**C_12_TPP**) significantly reduced body weight and body fat mass in HFD-fed male C57BL/6 mice housed at thermoneutrality [22]. Niclosamide ethanolamine (**NEN**), the salt form of FDA-approved anthelminthic drug, niclosamide, promoted body weight loss in HFD-fed C57BL/6J mice, but increased body mass in *db/db* mice [23]. In the aforementioned study, NEN improved glucose tolerance in male *db/db* mice, but this effect was not reproducible in other studies in either male or female mice [20,21,23,24]. Low dose treatment of mitochondrial uncoupler polysphondylium pseudo-candidum-1 (**Ppc-1**) induced 7–10% weight loss in albino mice given a standard chow diet, however this effect was not observed in mice administered higher doses [25]. In a diet-induced mouse model of NASH, **sorafenib**, an FDA-approved kinase inhibitor for advanced hepatocellular carcinoma, reversed weight gain and improved liver histopathology in male C57BL/6 mice [26]. **SR4** decreased body weight and improved glycaemic control in HFD-fed male C57BL/6 mice [27]. Finally, the mitochondrial uncouplers ellipticine (**ELL**), endosidin-9 (**ES9**), **FCCP**, **TTFB**, nitazoxanide (**NTZ**), tyrphostin A9 (**TyrA9**), and usnic acid (**UsAc**) have not been tested in obesity models.

**Table 1:** Structure and comments on therapeutic potential of mitochondrial uncouplers investigated in this study.

Structure	Comments on Therapeutic Potential
**BAM15**	**(2-fluorophenyl){6-[(2-fluorophenyl)amino](1,2,5-oxadiazolo[3,4-e]pyrazin-5-yl)}1amine (BAM15)** • BAM15 (0.1% w/w in HFD) reverses diet-induced obesity and glucose intolerance in C57BL/6J mice without decreasing lean mass [18,19], and has additive effects in combination with semaglutide (0.05% w/w in HFD) [61]. • BAM15 (0.2% w/w in HFD) improves body composition and completely normalises glucose tolerance in male and female *db/db* mice [20,21]. • Injection with BAM15 prevented renal damage in ischemia-reperfusion injury [62] and sepsis [63] models in C57BL/6J mice. • BAM15 (0.1% w/w in HFD) reduced breast tumour growth in C57BL/6 mice [64].
**C**_**12**_**TPP**	**Dodecyl-triphenylphosphonium (C_12_TPP)** • C_12_TPP (50 μmol/day/kg in drinking water) decreased food intake by ∼60% in C57BL/6J mice fed HFD in the first week of treatment and reduced body weight and fat mass. The study was performed at thermoneutrality because only mild weight loss occurred at room temperature [22]. A lower dose had no effect, and tolerability of higher doses is unknown.
**DNP**	**2,4-Dinitrophenol (DNP)** • DNP caused weight loss of up to 1.5 kg/week in humans, but was banned by the FDA in 1938 due to adverse side effects [6]. • DNP (∼89 mg/kg/day in drinking water) protected against weight gain and improved glucose tolerance in C57BL/6J mice fed HFD and housed at thermoneutrality, but not room temperature [14]. • DNP (∼30–105 μg/kg/day in drinking water) extended lifespan in Swiss mice by 7% [65]. • A controlled-release DNP formulation had a 10-fold improvement in lethal dose compared to non-formulated DNP and improved metabolic physiology without affecting body weight in obese nonhuman primates (5 mg/kg in banana mash) and rats (1 mg/kg in peanut butter) [13,66].
**ELL**	**Ellipticine (ELL)** • ELL has not been tested in mouse models of metabolic disease. It is an intercalative anti-tumour drug from the plant *Ochrosia elliptica* which inhibits topoisomerase II-β [67,68], and increases respiration in mitochondria isolated from both plants and animals at concentrations lower than ∼100 nmol/mg protein [69,70].
**ES9**	**Endosidin 9 (ES9)** • ES9 has not been tested in mouse models of metabolic disease. It was identified in a chemical library screen for inhibitors of endomembrane trafficking and increased respiration in *Arabidopsis thaliana* cells at concentrations as low as 1 μM [71].
**FCCP**	**Carbonyl cyanide p-trifluoromethoxyphenylhydrazone (FCCP)** • FCCP has not been tested in mouse models of metabolic disease, but has been tested acutely in C57BL/6J mice where it worsened stroke outcomes in an ischemic stroke model (1 mg/kg via IP injection) [72], and significantly inhibited tumour growth when combined with cisplatin in a subcutaneous tumour model (1 mg/kg via IP injection) [73]. • FCCP (1.2 mg/kg via IP injection) has also been tested in Fibrosarcoma MethA-bearing BALB/c mice where it was shown to synergistically decrease tumour volume when co-administered with PD-L1 mAB [74]. • Additionally, embryos from Swiss mice treated with FCCP (125 nM) produced offspring with increased adiposity, glucose intolerance and insulin resistance [75].
**NEN**	**Niclosamide ethanolamine (NEN)** • C57BL/6 mice treated with 0.15% NEN in HFD had improved whole body glucose metabolism [23,76]. • One study found that male *db/db* mice treated with 0.15% NEN in chow had improved glycaemic control [23]. However, three other studies performed in both male and female *db/db* mice were unable to replicate any antidiabetic effect of NEN at this dose [20,21,24]. • NEN reversed hyperglycaemia and improved diabetic kidney disease in mice with streptozotocin-induced type 1 diabetes when admixed in chow at 1% (w/w) [77].
**NTZ**	**Nitazoxanide (NTZ)** • NTZ was neuroprotective in a MPTP-induced mouse model of parkinsonism when administered at a dose of 50 mg/kg via daily IP injection [37]. • NTZ (administered as two 300 mg extended release tablets) was found to prevent progression to severe illness in those infected with SARS-CoV-2 (0.5% vs. 3.6% of patients progressed to severe disease when treated with NTZ vs placebo) [78]. • NTZ (100 mg/kg/day) decreased liver fibrosis by 32% in a CDAA/c mouse model of NASH, with synergistic effects observed when co-administered with Elafibranor (60% decrease in liver fibrosis) [52,53]. • A phase 2 trial to determine the safety and tolerability of NTZ (500 mg BID for 24 weeks) in humans with NASH was completed in 2020 and found that NTZ is well tolerated with only one patient reporting a significant adverse event [79].
**OPC-163493**	**4-(5-methyl-2-(4-(trifluoromethyl)phenyl)thiazol-4-yl)-1H-1,2,3-triazole-5-carbonitrile (OPC-163493)** • OPC-163493 had anti-diabetic effects (decreased HbA_1c_ and lowered fasting blood glucose) in Zucker diabetic fatty rats (10 mg/kg BID via oral gavage), Akita mice (0.01–0.02% w/w in chow), and Otsuka long-Evans Tokushima fatty rats (0.06% in w/w chow) [16]. • Kanemoto et al. found that OPC-163493 (0.01–0.02% w/w in chow) reduced hepatic lipids in *ob/ob* mice without affecting body weight and food intake [16]. • Rats treated with 300 mg/kg OPC-163493 in a repeated dose toxicity study had increased body temperature and rapid rigor mortis - phenotypes commonly observed with high-dose DNP treatment [15]
**Ppc-1**	***Polysphondylium pseudo-candidum*-1 (Ppc-1)** • Ppc-1 resulted in 7–10% weight loss in ICR mice (IP injection of 0.8 mg/kg QW) over eight weeks [25].
**Sorafenib**	**4-[4-({[4-chloro-3 (trifluoromethyl)phenyl]carbamoyl} amino)phenoxy]-N-methylpyridine-2-carboxamide (sorafenib)** • Sorafenib is a multikinase inhibitor that is FDA-approved for the treatment of advanced hepatocellular carcinoma [80]. • Low-dose sorafenib has anti-NASH effects in mice treated with diethylnitrosamine (DEN) and high-fat/high-cholesterol diet (15 mg/kg QAD via oral gavage) and in monkeys fed high-fat/high-cholesterol diet (1 mg/kg/3 days via IV injection) [26]. • Treatment with 1 μM sorafenib improved locomotor activity and extended lifespan in *C. elegans* [81].
**SR4**	**1, 3-bis(3,5-dichlorophenyl)urea (SR4)** • SR4 is a dichlorophenyl urea compound synthesised by Rahbar et al. that has antiproliferative and mitochondrial uncoupling effects in HepG2 hepatocarcinoma cells [82]. • SR4 (5 mg/kg via oral gavage) reduced body weight and improved metabolic parameters in HFD-induced obese C57BL/6J mice [27]. • SR4 ameliorated glucose intolerance and improved insulin sensitivity in *db/db* mice, though data was not shown [27]. • SR4 (4 mg/kg via oral gavage) decreased tumour burden in both syngeneic and nude mouse models of melanoma [83].
**TTFB**	**4,5,6,7-Tetrachloro-2-trifluoromethylbenzimidazole (TTFB)** • TTFB has not been tested in mouse models of metabolic disease.
**TyrA9**	**Tyrphostin A9 (TyrA9)** • TyrA9 has not been tested in mouse models of metabolic disease. It is hepatotoxic; inhibiting oxygen consumption in isolated rat hepatocytes with an EC_50_ value of 78 μM [35,36].
**UsAc**	**(+)-usnic acid (UsAc)** • UsAc has not been tested in mouse models of metabolic disease. Concentrations of 2–5 μM UsAc are acutely toxic to rat hepatocytes [84,85]. • UsAc is found in the unregulated weight-loss supplement LipoKinetix [86].

Abbreviations: BID; twice daily, CDAA/c; choline deficient, defined amino acid diet +1% choline, EC_50_; half maximal effective concentration, HFD; high fat diet, IP; intraperitoneal, IV; intravenous, MPTP; 1-methyl-4-phenyl-1,2,3,6-tetrahydropyridine, PD-L1 mAB; Programmed Cell Death Ligand 1 monoclonal antibody, QAD; every other day, QW; one weekly.

Herein, we compared the 15 mitochondrial uncouplers described above head-to-head in an *in vitro* oxygen consumption rate dose response screen to assess their dosing range and potential for unwanted mitochondrial inhibition during dose escalation. Molecules with the broadest dose-tolerance range were then assessed for pharmacokinetics, tolerability, and *in vivo* efficacy in mice. These experiments showed that less than half of the molecules tested had a broad dosing range *in vitro* without causing mitochondrial inhibition, and only five had drug exposure and tolerability suitable for testing *in vivo*. We assessed the therapeutic potential of these molecules by determining their efficacy in terms of improving metabolic parameters in *db/db* mice. *Db/db* mice are homozygous for the diabetes spontaneous mutation (Lepr^db^) and by nine weeks of age develop severe obesity, hyperglycaemia, hypertriglyceridemia and liver steatosis [28]. Conversely, *db/+* mice are metabolically normal and are used herein as littermate lean healthy controls. Finally, we benchmarked the efficacy of mitochondrial uncouplers to a lifestyle intervention of 35% calorie restriction, which is the amount of food consumed by non-hyperphagic *db/+* mice. In this study no mitochondrial uncoupler exhibited observed adverse effects at the doses tested for efficacy *in vivo* nor did they alter liver or kidney function enzymes. However, each mitochondrial uncoupler had a unique phenotype with respect to body weight, glucose control, and liver fat content, with some molecules exacerbating deleterious metabolic phenotypes and BAM15 generally outperforming all molecules and the calorie restriction positive control.

## 2 Materials and methods

### 2.1 Cell culture

Cells were grown in 10 cm cell culture dishes in a 37 °C humidified incubator with 5% CO_2_, in DMEM or DMEM/F12 supplemented with 10% (v/v) FBS, and 0.1% penicillin (10,000 IU/mL)/streptomycin (10 mg/mL). The origin and type of each cell line used is indicated in brackets: CHO–K1 (*Cricetulus griseus*; ovary epithelial), HEK-293T (*Homo sapiens*; kidney epithelial), HeLa (*Homo sapiens*; cervix epithelioid carcinoma), L6 (*Rattus norvegicus*; skeletal muscle myoblast), MCF7 (*Homo sapiens*; mammary gland epithelioid carcinoma), NIH-3T3 (*Mus musculus*; embryonic fibroblast), NMuLi (*Mus musculus*; liver epithelial), and RAW 264.7 (*Mus musculus;* Abelson murine leukaemia virus transformed macrophage). CHO–K1, HEK-293T, HeLa, MCF7, NIH-3T3, NMuLi and RAW 264.7 cells were passaged at 70–80% confluency. To prevent differentiation into multinucleated primary myotubes, L6 cells were passaged at 50–60% confluency.

### 2.2 Seahorse XF-96 oxygen consumption assays

**Figure 1:**
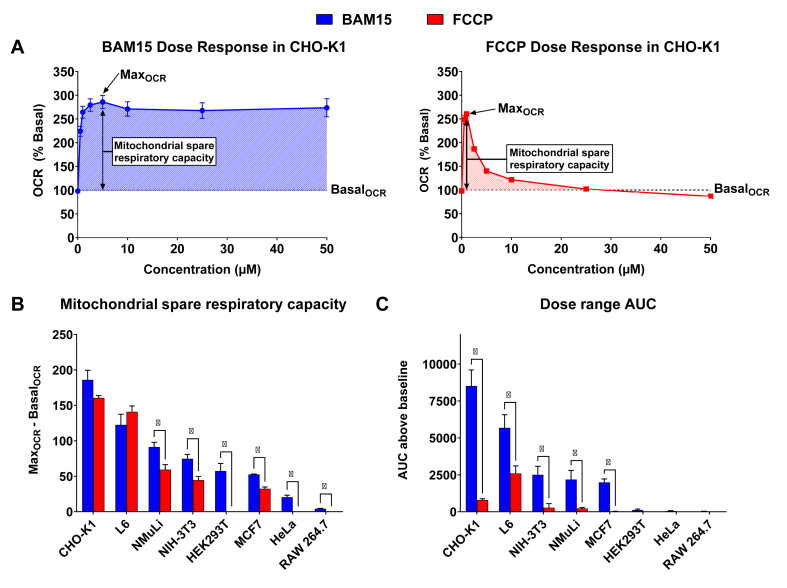
**BAM15 outperforms FCCP in detecting mitochondrial spare respiratory capacity in eight cell lines with CHO–K1 cells having the greatest spare respiratory capacity. A)** OCR of CHO–K1 cells is presented as a percentage of basal respiration. Data points represent the mean OCR at 24 min post injection of BAM15 or FCCP. The dashed line indicates basal respiration, defined as the mean OCR over the first 24 min of the assay prior to the addition of uncoupler. Shaded areas represent *in vitro* activity as defined by area under the curve (AUC) above baseline. **B)** Mitochondrial spare respiratory capacity of all cell lines. Mitochondrial spare respiratory capacity is defined as the difference between the Max_OCR_ induced by BAM15 or FCCP and the Basal_OCR_. **C)***In vitro* activity as determined by area under the curve (AUC) above baseline OCR. Error bars show SEM, *n* = 3 per condition from three separate experiments. Statistical significance was determined by unpaired t test with Welch correction. ∗ indicates *p* < 0.05, a statistically significant difference between either the mitochondrial spare respiratory capacity **(B)** or *in vitro* activity **(C)** when cells are treated with BAM15 or FCCP.

**Figure 2:**
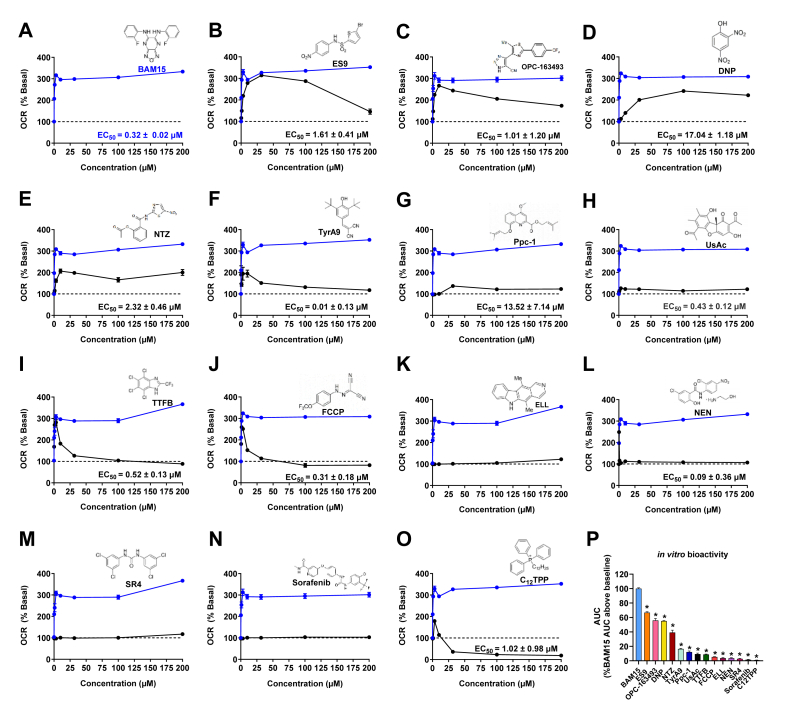
**BAM15, ES9, OPC-163493, NEN and NTZ stimulate Max_OCR_ at low concentrations and sustain basal or enhanced OCR over a wide concentration range in CHO–K1 cells. A–O)** OCR of CHO–K1 cells is presented as a percentage of basal. Data points represent the mean OCR over the first 24 min post injection of indicated uncoupler. The dashed line indicates basal respiration, defined as the mean OCR over the first 24 min of the assay prior to the addition of uncoupler. Uncouplers (structures shown) were run on Seahorse plates in groups of 3–4 with BAM15 run on each plate as a positive control – plate groupings were as follows; DNP, FCCP and UsAc; C_12_TPP, ES9 and TyrA9; NEN, NTZ and Ppc-1; SR4, TTFB, and ELL; OPC-163493 and Sorafenib. The effect of BAM15 is indicated by the blue line in each panel, while the activity of the illustrated uncoupler in indicated by the black line. Half-maximal effective concentration (EC_50_) ± standard error was determined by fitting the mean (*n* = 3) of three independent experiments to the [Agonist] vs. response – Variable Slope (four parameters) model for non-linear regression on GraphPad Prism (V10.2.1), where response was the mean OCR (% Basal) over the first 24 min post injection of uncoupler. **P)***In vitro* activity as determined by total area under the curve (AUC), expressed as a percentage of the AUC above baseline (100%) when CHO–K1 cells are treated with BAM15. Error bars show SEM, *n* = 3 per condition from three separate experiments for all uncouplers except **A)** BAM15 where *n* = 5 per condition from five separate experiments. ∗ indicates *p* < 0.05 as assessed by one-way ANOVA with all groups compared to BAM15. (For interpretation of the references to colour in this figure legend, the reader is referred to the Web version of this article.)

**Figure 3:**
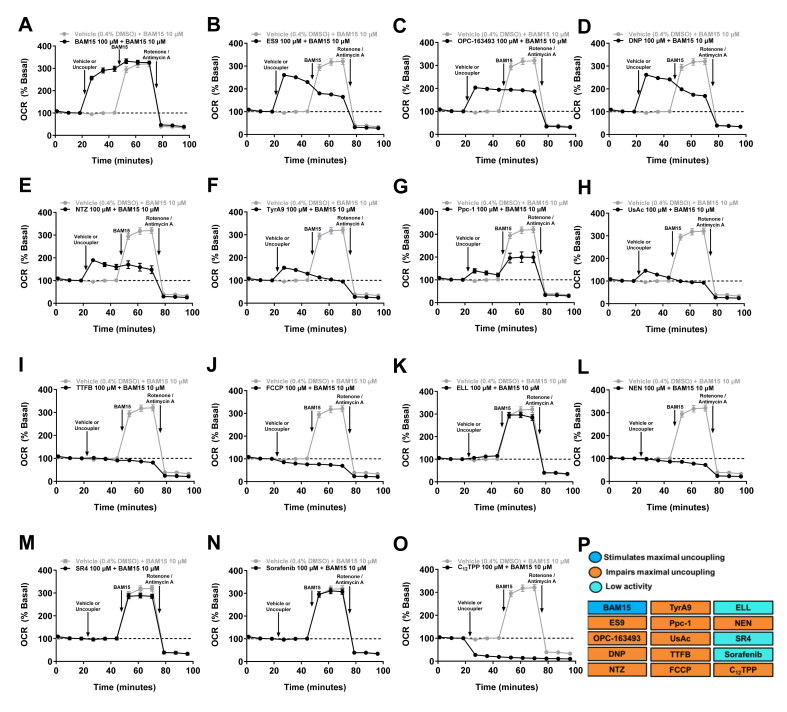
**Effect of uncouplers on maximal mitochondrial respiration in CHO–K1 cells in the absence or presence of BAM15. A–O)** OCR of CHO–K1 cells is presented as a percentage of basal over time. The dashed line indicates basal respiration, defined as the mean OCR over the first 24 min of the assay prior to the addition of uncoupler. At ∼24 min, 100 μM of uncouplers or vehicle (0.4% DMSO) was injected, followed by the injection of 10 μM BAM15 at ∼48 min, and 0.5 μM Rotenone/Antimycin A at ∼72 min. **P)** Table summarising the effect of the uncouplers on maximal mitochondrial respiration in intact CHO–K1 cells *in vitro.* Error bars show SEM, *n* = 3 per condition from three separate experiments for all uncouplers and vehicle.

CHO–K1, HEK-293T, HeLa, L6, MCF7, NIH-3T3, NMuLi and RAW 264.7 cells were seeded in Seahorse 96-well tissue culture plates at a density of 2.0 × 10^4^ cells/well in complete media (DMEM or DMEM/F12 supplemented with 10% (v/v) FBS, and 0.1% penicillin (10,000 IU/mL)/streptomycin (10 mg/mL). Cells were then allowed to adhere for 24 h in a 37 °C humidified incubator with 5% CO_2_. Prior to the commencement of the assay, cell culture media was replaced with Seahorse media (unbuffered phenol-red free DMEM supplemented with 25 mM glucose, 4 mM l-glutamine, and 1 mM sodium pyruvate, pH 7.4) and cells were incubated at 37 °C in a non-CO_2_ incubator for 40 min to allow for outgassing. Stock solutions of the uncouplers and/or Rotenone/Antimycin A were diluted in Seahorse media to give the final concentration indicated in Figure 1, Figure 2, Figure 3 with 0.4% (v/v) DMSO in wells. The total duration of the assay was ∼120 min (Figure 1, Figure 2) or ∼95 min (Figure 3). Basal oxygen consumption rate (OCR) was defined as the mean OCR over the first 24 min of the assay prior to the addition of uncouplers. For Figure 1, Figure 2, uncouplers were injected at ∼24 min, with each measurement cycle consisting of a 3-minute waiting, 3-minute mixing, and 2-minute measurement period. For Figure 3, 100 μM of uncouplers or vehicle (0.4% DMSO) was injected at ∼24 min, followed by 10 μM of BAM15 at ∼48 min, and 0.5 μM Rotenone/Antimycin A at ∼72 min. Data from Seahorse assays was acquired using the Agilent Seahorse Wave Desktop (V2.6.3) software. EC50 ± standard error was determined by fitting the mean (*n* = 3) of three independent experiments to the [Agonist] vs. response – Variable Slope (four parameters) model for non-linear regression on GraphPad Prism (V10.2.1), where response was mean OCR (% Basal) over 24 min after CHO–K1 cells were injected with the uncouplers (Figure 2).

### 2.3 Animal husbandry

All mouse experiments were conducted at UNSW under the approval of the UNSW Animal Care and Ethics Committee (project approval 20/67A). C57BL6J/Ausb mice were bred at Australian BioResources (Moss Vale, NSW, Australia). C57BL6J/Ausb mice were group-housed with up to five littermates and had *ad libitum* access to water and standard chow diet (Gordons Specialty Feeds, NSW, Australia). *Db/db* mice (BKS.Cg-Dock^7m^+/+Lepr^db^/J^Ausb^) were obtained from an existing line and bred at Australian BioResources (Moss Vale, NSW, Australia). Prior to drug treatment, *db/db* mice were group-housed with up to five littermates and provided with *ad libitum* access to food and water. When treatment commenced, all *db/db* mice were single-housed and provided with *ad libitum* access to food and water, unless otherwise specified. All mice were housed at 22 °C in pathogen-free conditions, with a light/dark cycle of 12 h. Mice were monitored as per ethical guidelines.

### 2.4 Drug exposure

8-week-old male C57BL/6J mice were administered 10 mg/kg of uncoupler by oral gavage (0.7% w/v methylcellulose (93% (v/v)), 2% v/v Tween-80, 5% DMSO). Blood samples were collected in heparinised capillary collection tubes (Sarstedt, 20.1309, Germany) at the time points indicated in Supplementary Fig. 3. Standards were prepared by spiking known concentrations (0.1, 1, 10, 100 and 1000 ng) of the uncoupler into whole blood prior to extraction. Plasma was obtained by centrifugation at 2000×*g* for 10 min at 4 °C. Seven microlitres (7 μL) of plasma was precipitated in 100 μL of 90% (v/v) acetonitrile and 10% (v/v) methanol. The resulting solution was vortexed for 5 s and then centrifuged at 21,130×*g* for 10 min at 4 °C. The supernatant was collected in auto-sampler vials for mass spectrometry.

### 2.5 LC-MS/MS determination of drug concentration

LC-MS/MS was performed using a Shimadzu Prominence LCMS-8030 as previously described [18]. All uncouplers were detected in positive mode; BAM15 with a transition of *m/z* 341 > 162 and retention time of 5.9 min, OPC-163493 with a transition of *m/z* 336 > 110 and retention time of 4.6 min, NEN with a transition of *m/z* 324 > 171 and retention time of 4.5 min, and ES9 with a transition of *m/z* 363 > 217 and retention time of 4.2 min. After entering systemic circulation, NTZ is rapidly deacetylated to the active metabolite tizoxanide (TIZ), which was detected in positive mode with a transition of *m/z* 264 > 263 and retention time of 3.9 min [29,30].

### 2.6 Preparation of animal diets

Chow diet (Gordons Specialty Feeds, NSW, Australia) was powdered using a food processor and then formed into pellets using a pellet mill (Gemco ZLSP-120 B). For diets containing drug, the chow diet was first powdered and mixed with the relevant concentration of drug and then formed into pellets as above.

### 2.7 Study design

Body weight and glucose tolerance of male *db/+* and *db/db* mice was assessed at 8–9 weeks of age to obtain baseline measurements. *Db/+* were used as lean metabolically healthy controls and given *ad libitum* access to chow diet. *Db/db* mice were stratified into 12 groups using R (V4.4.0). Eleven groups of mice were fed either; 1) *ad libitum* - chow (control), 2) chow with 0.15% BAM15 (w/w), 3) chow with 0.2% BAM15 (w/w), 4) chow with 0.1% NEN (w/w), 5) chow with 0.25% NEN (w/w), 6) chow with 0.01% OPC-163493 (w/w), 7) chow with 0.02% OPC-63493 (w/w), 8) chow with 0.015% ES9 (w/w), 9) chow with 0.03% ES9 (w/w), 10) chow with 0.2% NTZ (w/w), and 11) chow with 0.4% NTZ (w/w). Another group of mice was fed chow to receive ∼65% of the average daily food intake of control mice as a model of calorie restriction (CR).

Mice were on treatment for a period of four weeks, during which body weight and food intake were measured daily. Pilot studies demonstrated that food intake in *db/db* mice is difficult to measure accurately due to a propensity to powder diet leading to a high volume of pulverised food at the bottom of the cage. To counter this, food traps were placed beneath hoppers and leftover food in both the traps and hoppers was weighed daily. HbA1c was measured weekly. At the end of the treatment period, a glucose tolerance test was conducted, with fed and fasted bloods collected. At termination, blood was collected via cardiac puncture under isoflurane anaesthesia. Mice were euthanised by cervical dislocation and harvested tissues were flash-frozen in liquid nitrogen and stored at −80 °C.

### 2.8 Glucose tolerance test

Mice were fasted for 6 h and then received intraperitoneal injections of 20% (w/v) dextrose at a dose of 1 g/kg lean body mass. Blood glucose (BG) was measured at the time of fasting (random-fed BG) and then again prior to the commencement of the GTT following the 6-hour daytime fast (fasted BG). Blood glucose was measured at regular intervals over 120 min post-injection using an Accu-Chek Performa glucometer. The upper detection limit of the glucometer is 33.3 mM, as such readings above this (HI) were recorded as 33.3 mM. The rate of glucose clearance was measured using the trapezoidal method for calculation of area under the curve.

### 2.9 HbA1c measurement

HbA1c was measured once weekly using an A1cNow + Portable HbA1c Test Kit. As the lower detection limit of the kit is 4.4% HbA1c, readings below 4.4% (<4.4) were recorded as 4.4%. HbA1c was measured in the random-fed state.

### 2.10 Plasma insulin measurement

A glucose tolerance test was conducted at the end of the treatment period, with random fed and 6-hour fasted bloods collected. Blood was collected in heparin tubes and kept on ice prior to centrifugation at 2000×*g* for 10 min at 4 °C to extract plasma. Plasma insulin concentration was determined using the Crystal Chem Ultra-Sensitive Mouse Insulin ELISA kit, according to manufacturers' instructions for the high range (1–64 ng/mL) assay.

### 2.11 Homeostatic model assessment for insulin resistance (HOMA-IR)

The HOMA-IR index was calculated using fasting glucose and fasting insulin concentrations using the equation:

$$\text{HOMA}-\text{IR}=\frac{fasting\;glucose\;\left(mM\right)\times fasting\;insulin\;\text{to}\;\left(\mu \text{IU}/L\right)}{22.5}$$

Insulin concentration was converted from ng/mL to μIU/L using a molar mass of 5808 Da and a conversion factor of 1 μIU/L = 6000 pmol/L.

### 2.12 Blood collection and analysis

At termination, blood was collected via cardiac puncture under isoflurane anaesthesia. Blood was collected in EDTA tubes and kept on ice prior to centrifugation at 2000×*g* for 10 min at 4 °C to extract plasma. A clinical biochemistry panel was performed by Laverty Pathology Vetnostics (West Ryde, NSW, Australia) using a Cobas 8000 modular analyser (Roche, USA). This panel included markers of liver damage: AST, ALT and GLDH, as well as markers of kidney damage: creatinine and urea.

### 2.13 Hepatic lipid content

Frozen liver tissue was powdered in liquid nitrogen using a tissue pulveriser (CellCrusher, Cork, Ireland). A modified version of the Folch method was used to extract lipids [31]. Briefly, ∼25 mg of frozen powdered tissue was weighed out and vortexed with 533 μL chloroform and 267 μL methanol. Samples were sonicated for 10 min then digested on a rocker at room temperature for 1 h. A solution of sodium chloride (0.9% NaCl, 400 μL) was added and each sample was vortexed before centrifugation at 3000 rpm at room temperature for 10 min. The bottom-phase (chloroform containing lipid extract) was collected. Centrifugation and collection of the lipid extracted was repeated once. The lipid extract was dried under nitrogen gas using a TurboVap® Evaporator (Biotage, Uppsala, Sweden). Dried lipids were resuspended in 400 μL 95% (v/v) ethanol and heated to 37 °C prior to lipid assays. Colorimetric assays were used to measure triglyceride (Pointe Scientific, T7532, MI, USA) and cholesterol (ThermoScientific, TR13421) levels.

### 2.14 Histology

At euthanasia, the left lobe of the mouse livers was fixed in 10% formalin at room temperature. After ∼24 h, the sample was washed with 10% PBS, transferred to 70% (v/v) ethanol and stored at 4 °C until processing. Sample processing and staining was performed by the Biological Specimen Preparation facility at the Mark Wainwright Analytical Centre (UNSW, Sydney). Samples were embedded in paraffin and 5 μm sections were cut using a microtome. Prior to staining, slides were dewaxed by baking for 60 min at 58 °C and then put through a xylene/ethanol series. For Hematoxylin-eosin (H&E) staining, slides were stained in Harris hematoxylin for 5 min, rinsed with water, Scott’s solution, and then tap water again. Slides were then dipped in 0.5% acid alcohol and rinsed in tap water, with this process repeated twice. Finally, slides were stained with eosin for 4 min, then dehydrated and mounted. Microscope images were acquired for all stained slides using an Olympus VS200 Slide Scanner with a magnification of 40×. Analysis of scanned images was conducted in QuPath (V.0.4.4).

### 2.15 Data and statistical analysis

Statistical analysis was performed using GraphPad Prism (V10.2.1). Results are expressed as the mean of each treatment group, with error bars representing the standard error of the mean (SEM). A minimum of three biological replicates was included for each condition per experiment, with three independent experiments carried out per assay to serve as technical replicates. When data was normally distributed, the statistical significance of differences between groups was examined using the following statistical tests: unpaired t test with Welch correction was employed to determine if the mean between two independent groups was significantly different, one-way analysis of variance (ANOVA) was employed to determine whether the difference in the mean of more than two groups was significant, with Dunnett’s test applied to correct for multiple comparisons, and two-way ANOVA was employed to determine whether the difference in the mean of more than two groups with multiple treatments was significant, with Sidak’s test applied to correct for multiple comparisons.

## 3 Results

### 3.1 BAM15 outperforms FCCP in detecting mitochondrial spare respiratory capacity in cell lines representing diverse lineages with CHO–K1 cells having the greatest spare respiratory capacity

Molecular oxygen is the final electron acceptor in the ETC. When mitochondrial uncoupling occurs, nutrient oxidation and oxygen consumption increase as the cell increases flux through the ETC to maintain ATP production. Thus, oxygen consumption rate (OCR) is used as a proxy for mitochondrial respiration. The difference between basal respiration (Basal_OCR_) and maximum respiration (Max_OCR_) defines mitochondrial spare respiratory capacity.

To establish a standardised assay for comparing mitochondrial uncouplers, we first sought to identify a cell line with high mitochondrial spare respiratory capacity as this would maximise the ability to screen compounds for their efficacy in increasing OCR. The commonly used immortalised cell lines CHO–K1, HEK-293T, HeLa, L6, MCF7, NIH-3T3, NMuLi, and RAW 264.7 cells represent diverse lineages and were selected to identify cell lines with a large spare respiratory capacity suitable for comparing all 15 mitochondrial uncouplers. We first assessed the eight cell lines with two common uncouplers used commercially in Agilent Seahorse XF assay kits - BAM15 and FCCP [32].

All cell lines were treated with BAM15 or FCCP at concentrations over a 100-fold range from 0.5 to 50 μM with OCR measured over 120 min using the Seahorse XF Analyzer. Figure 1A illustrates the effect of BAM15 and FCCP on OCR in CHO–K1 cells. BAM15 stimulated a Max_OCR_ of ∼286% basal at 5 μM and sustained elevated OCR from 0.5 μM over a 100-fold range to 50 μM (Figure 1A). FCCP increased OCR to ∼260% basal at 1 μM, but higher concentrations induced a decline in OCR to sub-basal levels, indicating a narrow maximally effective concentration range before mitochondrial inhibition or failure (Figure 1A).

CHO–K1 and L6 cells had the highest spare respiratory capacity of the cell lines tested, with Max_OCR_ more than 2-fold higher than Basal_OCR_ (Figure 1B). Conversely, HeLa and RAW 264.7 cells had the lowest spare respiratory capacity (Figure 1B). Notably, BAM15 revealed a higher spare respiratory capacity than FCCP in seven of the eight cell lines (Figure 1B). FCCP failed to stimulate OCR above basal levels in HEK293T, HeLa and RAW 264.7; however, BAM15 treatment was able reveal the small spare respiratory capacity present in each cell line (Figure 1B and Supplementary Fig. 1).

We next determined the *in vitro* activity of FCCP and BAM15 in each cell line by calculating the incremental area under the curve (AUC) of OCR above Basal_OCR_ (shaded area in Figure 1A). High AUC levels indicate a wider effective concentration range and/or greater uncoupling activity over Basal_OCR._ BAM15 had a statistically superior dose-tolerance range compared to FCCP in CHO–K1, MCF7, NIH-3T3, NMuLi, and L6 cells (Figure 1C). Importantly, the decreased activity of FCCP with dose escalation above 1 μM was not due to oxygen depletion in the wells as there was more oxygen available in the wells than with similar concentrations of BAM15 (Supplementary Table 1).

### 3.2 Head-to-head *in vitro* activity comparison of 15 mitochondrial uncouplers

CHO–K1 cells had the greatest mitochondrial spare respiratory capacity, therefore they were used as the cell model to evaluate all uncouplers head-to-head (Figure 1). The mitochondrial uncouplers BAM15, DNP, FCCP, UsAc, SR4, ES9, TyrA9, NEN, NTZ, Ppc-1, C_12_TPP, TTFB, ELL, OPC-163493 and sorafenib were tested at concentrations of 0.32, 1, 3.16, 10, 31.6, 100 and 200 μM (high doses used to reach maximum solubility for most compounds) with OCR measured over 24 min post compound injection onto cells (Figure 2). The average of only the first 24 min was used because several compounds caused mitochondrial inhibition gradually over longer periods of time during the 120-minute assay (Supplementary Fig. 2). BAM15 was used as a reference control in every run and the activity of each compound tested was normalised to BAM15 to account for impacts of cells being assayed on different days where differences in cell passage number, confluence, media, or incubator conditions could influence OCR.

BAM15 sustained enhanced OCR (OCR ≥ 150% basal) at all tested concentrations over a 200-fold range between 1 and 200 μM (Figure 2A). BAM15 increased OCR with an average EC_50_ of 0.32 μM and an average Max_OCR_ of 333% of basal respiration (Figure 2A). Low doses of ES9 induced a steady increase in OCR, with respiration maintained at enhanced levels with dose escalation to 100 μM (Figure 2B). ES9 averaged an AUC activity that was 66% of BAM15 and had an EC_50_ of 1.6 μM (Figure 2B and P). DNP and OPC-163493 had the next best OCR and AUC activity ∼55% that of BAM15 (Figure 2C,D, and P). OPC-163493 increased OCR at low concentrations, with an EC_50_ of 1 μM and max efficacy at 10 μM (Figure 2C). DNP lacked the EC_50_ potency of BAM15 and ES9 with >10-fold higher EC_50_ of 17 μM, but it maintained enhanced OCR from 31.6 to 200 μM (Figure 2D). Neither DNP or OPC-163493 were able to induce Max_OCR_ as high as BAM15, with the Max_OCR_ stimulated by DNP and OPC-163492 respectively ∼27% and ∼22% lower than that seen with BAM15 treatment (Figure 2C,D).

NTZ lacked the potency and efficacy of BAM15 with a lower EC_50_ and lower Max_OCR_ but was able to enhance and maintain OCR levels at or above baseline over a wide concentration range and had an AUC activity of 35% of BAM15 (Figure 2E and P). TyrA9 stimulated Max_OCR_ ∼140% less than that observed with BAM15 treatment and had an AUC activity ∼39% of BAM15 (Figure 2F and P). Ppc-1 and UsAc had minimal uncoupling activity with OCR increasing to ∼130% and ∼125% basal respectively at 31.6 μM (Figure 2G,H). Additionally, both compounds induced a decline in OCR at concentrations over 31.6 μM to sub-basal levels after ∼40 min of treatment (Supplementary Fig. 2).

TTFB and FCCP had similar dose response profiles (Figure 2I,J). TTFB had an EC_50_ of 0.52 μM and induced a decline in OCR to sub-basal levels at 200 μM, with activity only ∼8.8% of BAM15 (Figure 2I and P). FCCP had similar potency to BAM15 with an EC_50_ of 0.31 μM (Figure 2J). However, higher concentrations of FCCP induced a decline in OCR that plateaued to a sub-basal level, with a dose-tolerance range less than 5% of BAM15 indicating a narrow effective concentration range (Figure 2J and P). Both TTFB and FCCP stimulated a Max_OCR_ that was lower than that seen with BAM15 (280% and 260% basal, respectively) at a low micromolar concentration before causing mitochondrial inhibition (Figure 2I,J).

ELL increased OCR above basal levels at concentrations >100 μM; however, the Max_OCR_ stimulated was only ∼122% of basal respiration and the *in vitro* dose-tolerance range was only ∼3.8% that of BAM15 (Figure 2K and P). NEN stimulated ∼25% lower Max_OCR_ than that seen with BAM15 but was more potent with an EC_50_ of 0.09 μM (Figure 2L). Notably, NEN only sustained enhanced OCR at a concentration of 0.32 μM, with a dose-tolerance range only ∼3.7% that of BAM15 (Figure 2L and P).

The three compounds with the lowest AUC were SR4, sorafenib and C_12_TPP. SR4 only increased OCR above basal levels at 200 μM, with the Max_OCR_ stimulated ∼117% of basal respiration (Figure 2M). Sorafenib did not have any uncoupling activity within the tested concentration range over the 120-minute assay period (Figure 2N and Supplementary Fig. 2). Sorafenib has not been shown to act as a mitochondrial uncoupler in intact cells. Previous studies have only demonstrated mitochondrial uncoupling action in isolated mitochondria and permeabilised hepatocytes, suggesting that the molecule may be unable to induce significant acute mitochondrial uncoupling in intact cells [26]. C_12_TPP induced a Max_OCR_ of ∼178% basal achieved with 3.16 μM treatment (Figure 2O); however, higher doses resulted in severe mitochondrial inhibition with OCR falling to ∼18% of baseline (Figure 2O and P). These data with C_12_TPP are consistent with previous studies where C_12_TPP had a mild effect to increase OCR in isolated mitochondria but did not significantly increase OCR in intact yeast cells unless co-administered with FCCP or DNP [33].

ATP synthase is the major conduit through which protons re-enter the mitochondrial matrix. Thus, we sought to investigate whether the selected uncouplers could stimulate oxygen consumption in the presence of the ATP synthase inhibitor oligomycin. CHO–K1 cells were serially treated with oligomycin (1 μM), then each uncoupler at the lowest concentration which yielded Max_OCR_ in the dose response assay, and finally rotenone/antimycin A (0.5 μM) to block mitochondrial ETC-derived respiration. As expected, treatment with oligomycin induced a decrease in OCR due to the loss of mitochondrial respiration associated with ATP production (Supplementary Fig. 6). Twelve uncouplers including BAM15, ES9, OPC-163493, DNP, NTZ, TyrA9, Ppc-1, UsAc, TTFB, FCCP, NEN and C_12_TPP were largely unaffected by the presence of oligomycin. However, two uncouplers including NTZ and Ppc-1 were mildly impaired by oligomycin, suggesting that their activity may be partially dependent on ATP synthase, while C12-TPP stimulated ∼60% higher OCR in the presence of oligomycin initially before the OCR curves converged over time (Supplementary Fig. 6). The higher OCR in the presence of oligomycin may indicate that C12-TPP works better under hyperpolarised conditions as TPP is positively charged.

### 3.3 Most uncouplers impair maximal mitochondrial respiration *in vitro*

Figure 2 shows that only 4 of the 15 mitochondrial uncouplers, including BAM15, FCCP, TTFB and ES9, could stimulate near maximal respiration, and only BAM15 maintained the high OCR over a broad dose response. To determine if decreased OCR vs BAM15 is a consequence of mitochondrial impairment or a result of another phenotype like self-limiting behaviour, we conducted an assay in which CHO–K1 cells were treated with each uncoupler followed by BAM15. In this assay, there are four possible outcomes for molecules that do not stimulate maximal respiration. The first possibility is that the uncoupler alone has minimal effect to increase OCR, and when BAM15 is added OCR still does not increase. This outcome would mean that the molecule is impairing mitochondria as it blocks BAM15 from increasing respiration. A second outcome could be that the molecule alone does not increase OCR, but when BAM15 is added the cells respire to their Max_OCR_. This outcome would mean that the first molecule lacks uncoupling activity in CHO–K1 cells but does not impair mitochondrial respiration. The third possible outcome is that the molecule alone partially increases mitochondrial respiration, and BAM15 addition stimulates Max_OCR_. This outcome would indicate that the first uncoupler is not impairing maximal mitochondrial respiration and may be self-limiting or have mild activity for other reasons. Finally, the fourth potential outcome is that the uncoupler alone partially increases mitochondrial respiration, but BAM15 addition cannot stimulate Max_OCR_. This outcome would indicate that the uncoupler is impairing maximal mitochondrial respiration.

Through this assay, we observed that 11 of the 15 molecules tested impaired maximal mitochondrial respiration including ES9, OPC-163493, DNP, NTZ, TyrA9, Ppc-1, UsAc, TTFB, FCCP, NEN, and C_12_TPP (Figure 3). Of these molecules, it should be noted that FCCP and C_12_TPP induced a decrease in OCR to sub-basal levels where basal OCR could not be restored with BAM15 treatment, suggesting significant inhibition of mitochondrial respiration and/or cytotoxicity (Figure 3J and O). A similar trend of mitochondrial impairment was observed with TTFB and NEN, where these molecules did not significantly increase OCR above basal levels and blocked the ability of BAM15 to increase respiration (Figure 3I and L). ES9, OPC-163493, DNP, NTZ, TyrA9, Ppc-1 and UsAc all stimulated an increase OCR to sub-maximal levels, and blocked BAM15 from stimulating maximal respiration (Figure 3B–H). Only 100 μM BAM15 triggered maximal respiration and additional treatment with 10 μM BAM15 had minimal impact, as expected (Figure 3A).

Three of the 15 molecules tested had essentially no uncoupling activity in intact CHO–K1 cells including ELL, SR4 and Sorafenib (Figure 3K, m-n). However, these compounds did not appear to impair maximal mitochondrial respiration, as BAM15 injection was able to stimulate Max_OCR_ (Figure 3K, M and N). In summary, 11 of the 15 molecules tested impaired maximal mitochondrial respiration, three molecules had no uncoupling activity, and only BAM15 was able to stimulate Max_OCR_ over a broad dose range without observed mitochondrial inhibition (Figure 3A-P).

### 3.4 High dose BAM15 treatment prevents weight gain and lowers fat mass in *db/db* mice

We next selected molecules for *in vivo* testing. Sorafenib, Ppc-1, SR4, UsAc, and ELL were excluded from animal studies due to minimal activity in living cultured cells (Figure 2, Figure 3). FCCP, C_12_TPP and TTFB were excluded from animal studies due to the very narrow range of activity before observed signs of mitochondrial fatigue or inhibition (Figure 2, Figure 3). TyrA9 had moderate activity, but published data indicates that the compound induces mitochondrial fragmentation and severe dysfunction [34]. Moreover, malonitrile, a product of TyrA9 hydrolysis has been shown to metabolise into cyanide *in vivo* by the liver and thus the compound has significant hepatotoxicity – this, in addition to its low LD_50_ in rodents led to its exclusion from animal studies [35,36]. Similarly, DNP was not further investigated in mice due to its lack of anti-obesity efficacy in obese mice housed at room temperature and its well-characterised narrow therapeutic window [6,14]. The top three compounds BAM15, ES9, and OPC-163493 were selected for mouse studies based on their broad effective dosing range without triggering mitochondrial inhibition (Figure 2, Figure 3). NEN and NTZ were selected for further characterisation in mice despite lower AUC activity as both molecules have FDA approval for use in humans and/or have reported efficacy in mouse models [23,37].

Dose selection was guided by published studies available for BAM15, NEN and OPC-163493, as well as by pharmacokinetic testing conducted in C57BL/6J mice administered 10 mg/kg of BAM15, NEN, NTZ, ES9 or OPC-163493 via oral gavage. Published studies showed BAM15 efficacy in *db/db* male mice at 0.2% (w/w) admixture in chow diet [20,21] and NEN efficacy in *db/db* male mice at 0.15% (w/w) (1500 ppm) admixture in chow diet [23]. OPC-163493 has been shown to improve glucose tolerance in *ob/ob* mice as a 0.01% (w/w) admixture in chow diet, however higher doses (0.02% w/w) resulted in significant hyperphagia [16]. The pharmacokinetic studies revealed that ES9 had the highest maximum plasma concentration (C_max_) of 72.3 μM, followed by OPC-163493 (30.89 μM) and BAM15 (6.76 μM), while NEN and NTZ had nanomolar C_max_ values of 820 nM and 410 nM respectively (Supplementary Fig. 3). Half-life (t_1/2_) followed a similar trend, with ES9 having the longest half-life of 21.6 h, followed by OPC-163493 (3.06 h), BAM15 (2.09 h), NEN (1.35 h) and NTZ (0.75 h) (Supplementary Fig. 3). Considering the range of drug exposures and half-lives that would necessitate complex oral gavage dosing regimens and corresponding vehicle controls, we elected to administer all molecules to mice by admixture into food.

**Figure 4:**
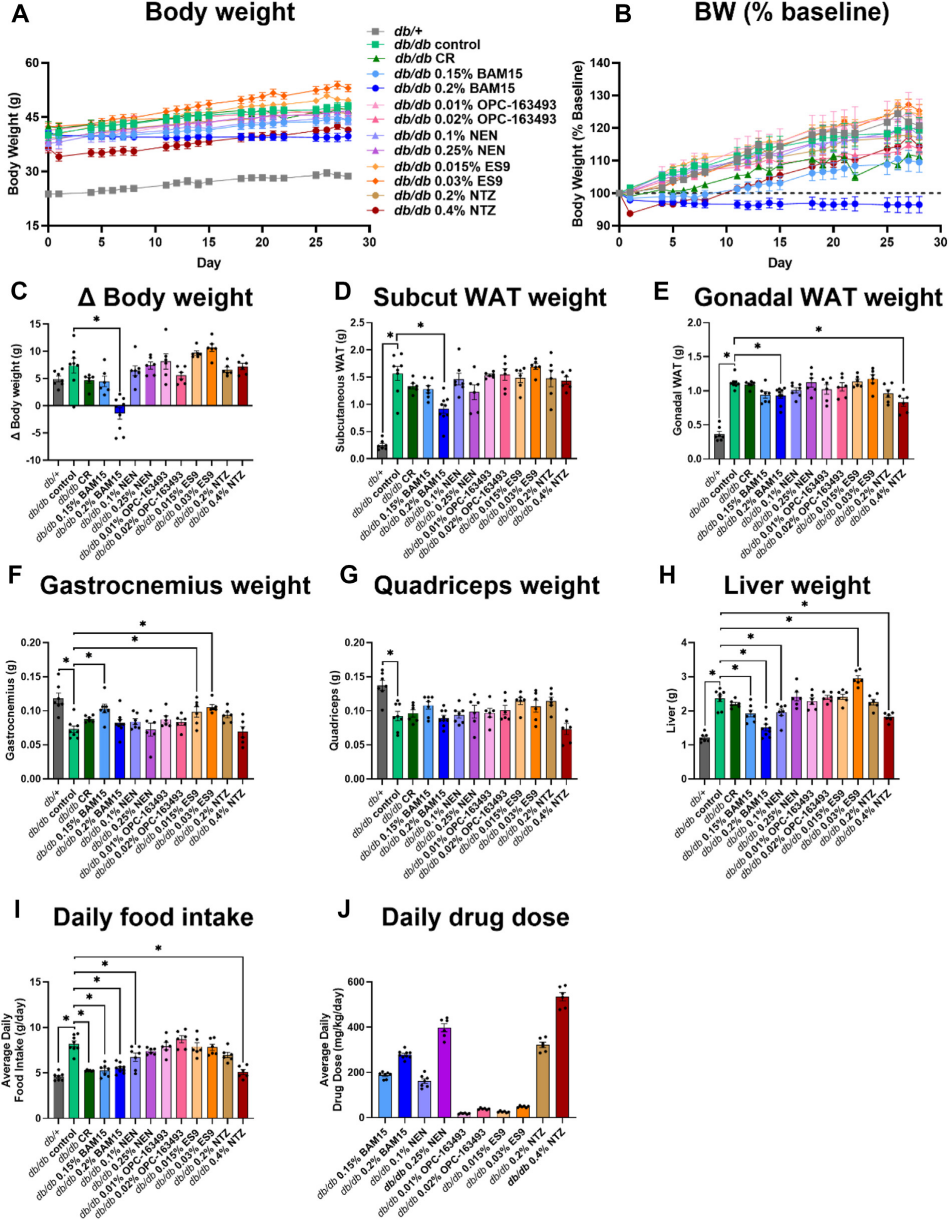
**Efficacy of mitochondrial uncouplers on body composition and food intake in *db/db* mice. *Db/db* mice were treated with uncouplers or calorie restricted for four weeks, with body weight/food intake measured daily. Δ indicates the difference between the post-treatment and pre-treatment measurement. A)** Body weight over time. **B)** Body weight (BW) over time expressed as % of baseline body weight. **C)** Δ Body weight. Wet weight of **D)** subcutaneous white adipose tissue (WAT), **E)** gonadal WAT, **F)** gastrocnemius, **G)** quadriceps, and **H)** liver. **I)** Daily average food intake. **J)** Daily average drug dose calculated from food intake. Graphs show mean ± SEM, *n* = 6–9 per group. ∗ indicates *p* < 0.05 as assessed by one-way ANOVA with all groups compared to *db/db* control.

*Db/db* mice were stratified into 12 groups where 11 groups of mice were fed chow diet *ad libitum* and one group was subjected to 35% calorie restriction (CR). The *ad libitum* fed groups consisted of mice fed chow (control), or chow containing either 0.15% BAM15 (w/w), 0.2% BAM15 (w/w), 0.1% NEN (w/w), 0.25% NEN (w/w), 0.01% OPC-163493 (w/w), 0.02% OPC-163493 (w/w), 0.015% ES9 (w/w), 0.03% ES9 (w/w), 0.2% NTZ (w/w), or 0.4% NTZ (w/w). Mice were fed chow or one of the 10 custom diets over a period of 4 weeks from 9 to 13 weeks of age. At the end of the study period, the *db/db* control mice were 20% heavier than they were at the start of the study and weighed approximately ∼75% more than the *db/+* mice (Figure 4A–B). There were small differences in the baseline pre-treatment body weight across groups that were not statistically significant (Supplementary Fig. 4); therefore, we accounted for the variability by calculating the change (Δ) between the post-treatment and pre-treatment results. The only group to have a statistically significant decrease in weight gain compared to *db/db* controls were mice fed 0.2% BAM15 that weighed ∼17% less than controls (39.4 g vs 47.7 g, respectively) (Figure 4A–C). The CR mice and those fed 0.15% BAM15 or 0.02% OPC-163493 exhibited a non-significant trend for lower body weight while mice fed 0.015% and 0.03% ES9 had a non-significant trend for increased weight gain compared to controls (Figure 4C). NTZ and NEN had little impact on body weight compared to controls (Figure 4A–C).

The masses of key metabolic tissues including liver, adipose, and muscle were measured at study termination. All *db/db* mice had greater gonadal and subcutaneous adipose tissue weight than *db/+* mice (Figure 4D–E). Only the *db/db* mice fed high dose BAM15 had statistically less subcutaneous and gonadal fat compared to the control group (41% and 17% less respectively) (Figure 4D–E). The only other group with statistically less gonadal fat than the control group was the 0.4% NTZ group (26% less) (Figure 4E). Subcutaneous fat mass trended lower in the CR, 0.15% BAM15 and 0.25% NEN groups, though not statistically significant (Figure 4D). Gonadal fat mass trended lower in the 0.15% BAM15 and 0.2% NTZ groups, though not statistically significant (Figure 4E). All *db/db* mice had smaller quadriceps and gastrocnemius muscles than *db/+* mice (Figure 4F). Mice treated with 0.15% BAM15, 0.015% ES9 and 0.03% ES9 had significantly larger gastrocnemius than the control group; however, no intervention significantly impacted quadriceps mass (Figure 4F–G). Liver weight nearly doubled in the *db/db* control mice compared to the *db/+* mice (Figure 4H), and four treatment groups statistically decreased liver mass including both doses of BAM15, low dose NEN, and high dose NTZ (Figure 4H). In contrast, mice treated with 0.3% ES9 had significantly larger livers, with a ∼25% increase in wet weight compared to the *db/db* control group (Figure 4H).

The calorie restricted group of mice was fed 35% less food than *db/db* controls to match the food intake of metabolically normal *db/+* controls (Figure 4I). Both doses of BAM15, the low dose of NEN and the high dose of NTZ all restored normal food intake to levels consistent with the amount of food consumed by *db/+* mice, which was statistically less than *db/db* controls (Figure 4I). The average daily drug dose for mice treated with BAM15, NEN, OPC-163493, ES9, and NTZ is presented in Figure 4J.

Mice treated with 0.25% NEN, 0.2% NTZ, 0.01% OPC-163493, and 0.02% OPC-163493 were not significantly different in their body weight, food intake, or metabolic tissue weights compared to control mice (Figure 4).

### 3.5 BAM15 and high dose ES9 treatments improved glucose control in *db/db* mice

**Figure 5:**
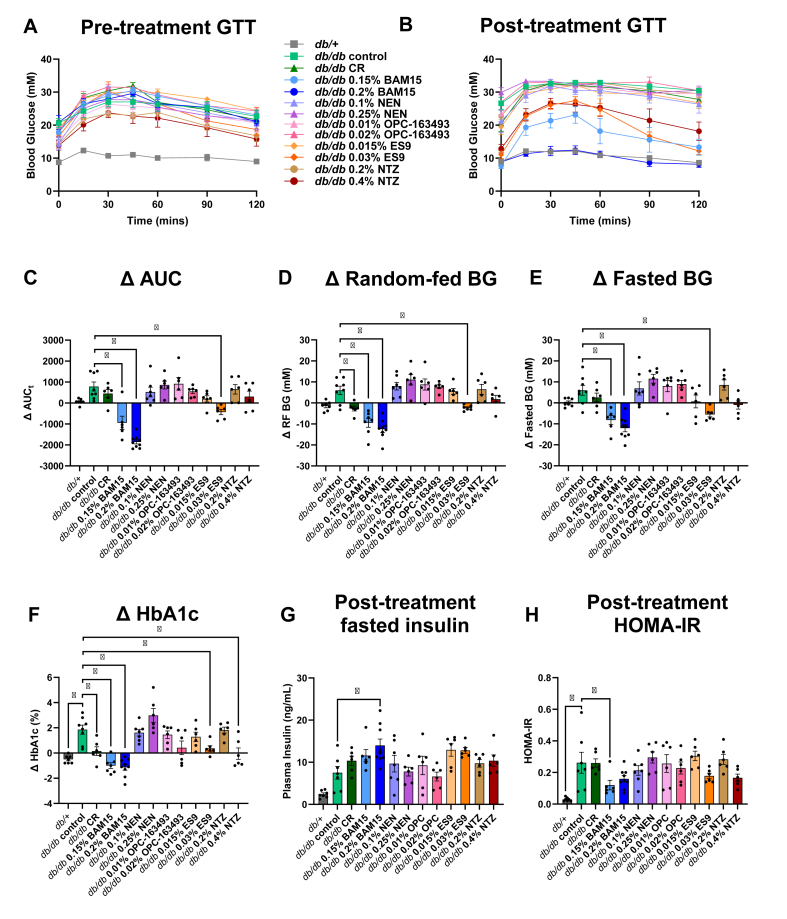
**BAM15 and ES9 treatments improve glucose control in *db/db* mice. Intraperitoneal glucose tolerance tests were conducted A)** prior to treatment and **B)** after four weeks of treatment with uncouplers or calorie restriction with total area under the curve (AUC) calculated. Δ indicates the difference between the post-treatment and pre-treatment measurement. **C)** Δ AUC, **D)** Δ blood glucose in random fed state (RF BG), **E)** Δ blood glucose after a 6-hour daytime fast (Fasted BG), **F)** Δ HbA1c. **G)** Post treatment fasted insulin. **H)** Post-treatment HOMA-IR calculated using fasted BG and fasted plasma insulin. Graphs show mean ± SEM, *n* = 6–9 per group. ∗ indicates *p* < 0.05 as assessed by one-way ANOVA with all groups compared to *db/db* control.

Glucose tolerance tests (GTTs) were conducted prior to starting treatment (pre-treatment) and after four weeks of treatment (post-treatment) to assess the effect of the uncouplers or calorie restriction on glucose homeostasis. Both GTT experiments showed impaired glucose tolerance in *db/db* control mice compared to *db/+* mice (Figure 5A–B). The post-treatment GTT shows that mice treated with 0.15% BAM15, 0.2% BAM15, 0.03% ES9, and 0.4% NTZ had significantly better glucose tolerance than the control group, with the AUC respectively ∼44%, ∼68%, ∼35%, and ∼29% lower than the control group (Figure 5B, Supplementary Fig. 5a). BAM15 dose-dependently improved glucose tolerance, with the 0.2% BAM15 group showing complete restoration of glucose tolerance to the level seen in metabolically healthy *db/+* mice (Figure 5B).

There were small differences in the baseline pre-treatment glucose parameters across groups that were not statistically significant (Supplementary Fig. 5); therefore, we accounted for the variability by calculating the change (Δ) between the post-treatment and pre-treatment results. The GTT ΔAUC increased for all treatment groups (worsening glucose tolerance) except for the 0.15% BAM15, 0.2% BAM15 and 0.3% ES9 groups that showed statistically significant protection from worsening glucose tolerance (Figure 5C).

Four treatment groups improved fasting and random fed glucose control including both BAM15 groups, high dose ES9 and high dose NTZ (Supplementary Fig. 5b and c). Specifically, mice treated with either dose of BAM15 had blood glucose levels similar to *db/+* mice, while high dose ES9 and high dose NTZ had partial efficacy (Supplementary Fig. 5b and c). When comparing the change in blood glucose from pre-treatment to post-treatment, only the BAM15 groups and high dose ES9 statistically prevented worsening hyperglycaemia in the fed and fasted states, while calorie restriction prevented an increase in random-fed blood glucose (Figure 5D,E).

In mice, erythrocytes (which carry haemoglobin) have a lifespan of 55 days so measuring the levels of glycated haemoglobin (HbA1c) provides insight into average blood glucose levels over a longer period. HbA1c in the *db/db* control mice increased from ∼6% to ∼8% over the course of the study (Supplementary Fig. 5d). Calorie restriction and treatment with both doses of BAM15, 0.3% ES9 and 0.4% NTZ all lowered HbA1c levels (Supplementary Fig. 5d) with the BAM15 treatments having the greatest efficacy restoring HbA1c to the ∼4% levels seen in the *db/+* group (Figure 5F). Mice treated with 0.02% OPC-163493 also saw a less substantial (but not statistically significant) increase in HbA1c compared to the control group (Figure 5F).

Mice treated with BAM15 ate a similar amount of chow diet as the calorie-restricted mice but had lower GTT AUC, HbA1c, random-fed and 6-hour fasted blood glucose levels (Supplementary Fig. 5). Mice fed NEN, 0.01% OPC-163494, 0.015% ES9, and 0.2% NTZ did not have significantly lower random-fed or 6-hour fasted blood glucose, lower HbA1c or improved glucose tolerance compared to the control group (Figure 5).

*Db/db* control mice had ∼3 times greater concentration of insulin in their fasted plasma than *db/+* mice (Figure 5G). Mice fed 0.2% BAM15 had significantly higher concentrations of insulin in their fasted plasma after four weeks of treatment compared to *db/db* control mice (Figure 5G). No other *db/db* groups had a statistically significant insulin phenotype (Figure 5G). The homeostatic model assessment for insulin resistance (HOMA-IR) was calculated using fasted glucose and insulin concentrations at the end of treatment to give an approximation of insulin resistance. Only two groups had statistically significant differences in HOMA-IR compared to the *db/db* controls - these were the *db/+* and the 0.15% BAM15 groups (Figure 5H). The *db/db* controls had HOMA-IR ∼10x greater than the *db/+* mice (Figure 5H). HOMA-IR in the 0.15% BAM15 group was 50% lower than in the control group (Figure 5H). Similarly, HOMA-IR is ∼33% lower in the 0.2% BAM15 group suggesting that the increased insulin concentration was not secondary to increased insulin resistance relative to *db/db* control mice (Figure 5H).

### 3.6 Opposing effects of BAM15 and ES9 on liver fat

**Figure 6:**
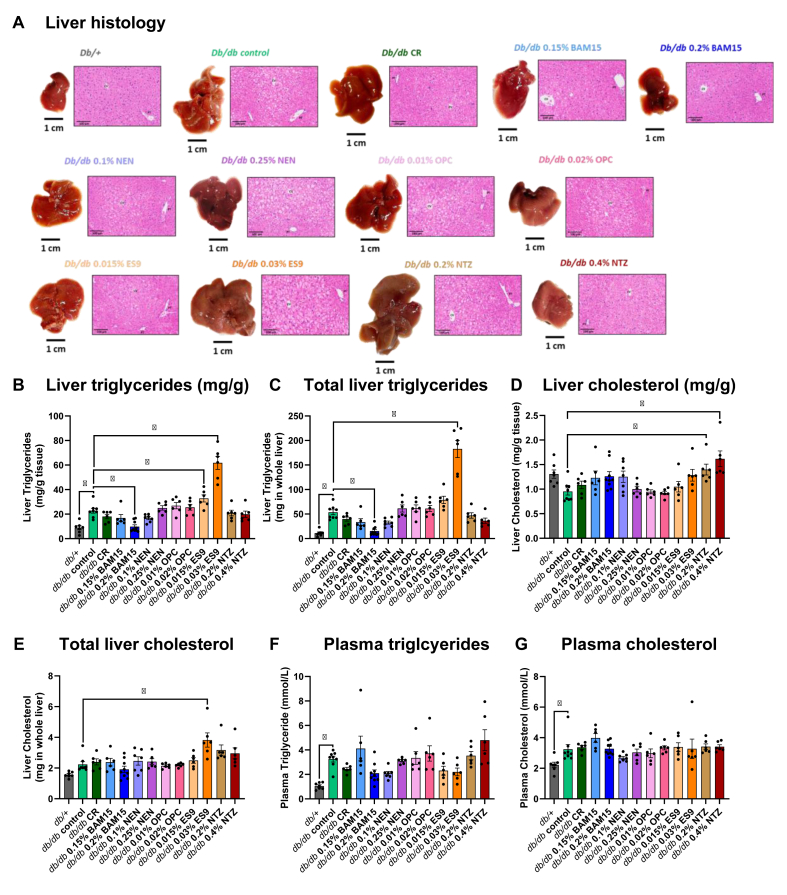
**BAM15 and ES9 have opposing effects on liver lipid levels in *db/db* mice. A)** Representative images of liver from *db/+* and *db/db* mice treated with uncouplers or calorie restricted for four weeks stained with haematoxylin-eosin. Liver triglyceride levels as determined by colorimetric assay through reaction with GPO reagent presented as **B)** triglyceride **(**mg) per g of liver tissue and **C)** total liver triglyceride content. Liver cholesterol levels determined by colorimetric assay using Infinity cholesterol liquid stable reagent presented as **D)** cholesterol **(**mg) per g of liver tissue and **E)** total liver cholesterol content. **F)** Plasma triglyceride content, and **G)** plasma cholesterol content. Graphs show mean ± SEM, *n* = 6–9 per group. ∗ indicates *p* < 0.05 as assessed by one-way ANOVA with all groups compared to *db/db* control.

At study termination, liver and plasma triglyceride and cholesterol content were determined. Fixed sections of liver were stained with hematoxylin-eosin to visualise steatosis (Figure 6A). Liver triglyceride concentration was ∼2.5 time higher in *db/db* control mice compared to *db/+* mice with total liver triglyceride content ∼4.9 times higher in *db/db* control mice (Figure 6B–C). The only treatment groups that significantly altered liver triglyceride concentration and total liver triglyceride content in *db/db* mice were the high dose BAM15 group and high dose ES9 groups (Figure 6B–C). The high dose BAM15 group decreased liver triglyceride concentration ∼2.3 fold and decreased total liver triglyceride content ∼3.6-fold compared to *db/db* controls. Conversely, the high dose ES9 group increased liver triglyceride concentration ∼2.7 fold and increased total triglyceride content ∼3.4-fold compared to *db/db* controls (Figure 6B). The lower dose ES9 group also significantly increased liver triglyceride concentration but this effect was not statistically significant when considering the total liver triglyceride content (Figure 6B–C).

The only treatment groups that significantly increased liver cholesterol concentration were the two doses of NTZ, with the low dose increasing liver cholesterol concentration ∼1.5 fold and the high dose increasing liver cholesterol concentration ∼1.7 fold compared to *db/db* control mice (Figure 6D). However, this effect was not statistically significant when considering the total liver cholesterol content (Figure 6E). The only treatment that significantly altered total cholesterol content was 0.03% ES9, which increased cholesterol ∼1.7-fold compared to *db/db* control mice (Figure 6E.)

Plasma triglyceride concentration was ∼3 times higher in *db/db* control mice than in *db/+* mice (Figure 6F). Plasma triglyceride levels were statistically unchanged by all treatment interventions compared to *db/db* controls. Plasma cholesterol concentration was ∼1.5 times higher in *db/db* controls compared to *db/+* mice, with no other statistically significant differences between *db/db* treatment groups (Figure 6G).

### 3.7 Mitochondrial uncoupler treatment did not raise liver or kidney function test enzymes

**Figure 7:**
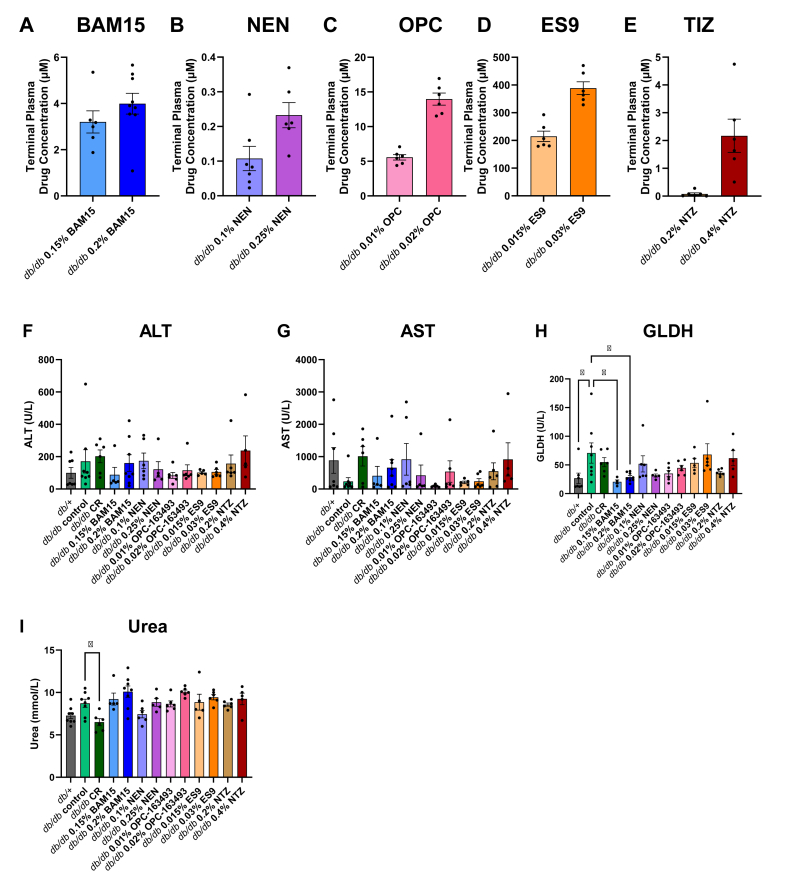
**Plasma drug concentration and markers of tissue damage in *db/db* mice at study endpoint. Plasma was collected via cardiac puncture at time of euthanasia. A–E)** Terminal plasma drug concentration was measured by LC-MS/MS. **F–I)** Terminal plasma concentration of liver function indicators (ALT, AST and GLDH) and kidney function indicator (urea). Graphs show mean ± SEM, *n* = 6–9 per group for plasma drug concentration measurement. Due to the limited volume of samples, not all liver and kidney function tests could be performed for all mice. For ALT, AST, GLDH, and urea measurements *n* = 8 for *db/db* controls, *n* = 5 for 0.15% BAM15, *n* = 8 for 0.2% BAM15, *n* = 6 for 0.1% NEN, *n* = 5 for 0.25% NEN, *n* = 5 for 0.015% ES9, and *n* = 5 for 0.4% NTZ.

The biggest concern for mitochondrial uncoupler therapeutics is safety, therefore we assessed drug levels and biomarkers of tissue damage from terminal blood samples. Plasma drug concentration for all uncouplers were within the concentration range found to induce uncoupling *in vitro*, except for in mice fed 0.2% NTZ in which the terminal plasma concentration of TIZ, the bioactive product of NTZ metabolism, was lower than the concentration at which oxygen consumption rate was enhanced *in vitro* (Figure 7A–E).

No compound significantly increased plasma markers of liver or kidney damage including ALT, AST, GLDH, or urea compared to the *db/db* control (Figure 7F–I). *Db/db* mice had increased liver damage marker GLDH compared to *db/+* mice, which was restored to normal levels by both doses of BAM15 but no other compound (Figure 7H). Elevated urea is a marker of impaired renal function. Urea levels were not statistically different between *db/db* and *db/+* mice and the only intervention to lower urea levels was calorie restriction (Figure 7I).

### 3.8 *Db/db* mice treated with mitochondrial uncouplers have diverse metabolic phenotypes

**Figure 8:**
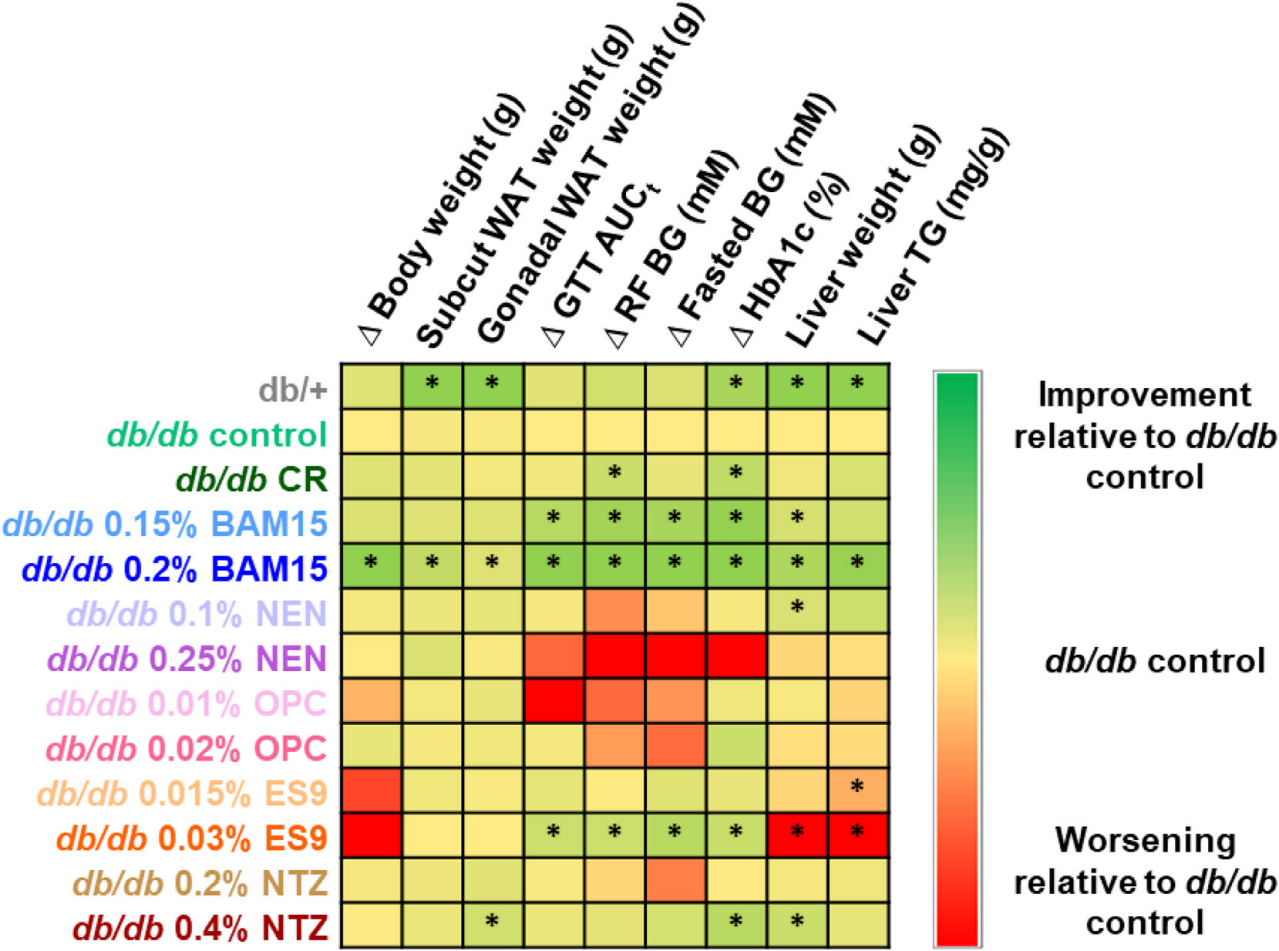
**Heat map summarising the effect of mitochondrial uncouplers or calorie restriction on *db/db* mice after four weeks of treatment.** Delta (Δ) indicates the difference between the post-treatment and pre-treatment measurement. *n* = 6–9 per group. Abbreviations: BG; blood glucose, RF; random fed, subcut; subcutaneous, WAT; white adipose tissue. ∗*p* < 0.05 vs. *db/db* control.

The metabolic data obtained from this study (with all uncouplers and calorie restriction) has been summarised and shown graphically in Figure 8. Overall, the results show BAM15 dose-dependently improved body weight and metabolic parameters in *db/db* mice, with 0.2% BAM15 treatment yielding statistically significant improvements in body weight, fat pad weight, glucose tolerance, blood glucose, HbA1c, liver weight and triglyceride content (Figure 8). Treatment with 0.03% ES9 significantly improved glucose tolerance, blood glucose levels, and HbA1c, but increased body weight, liver size and steatosis relative to *db/db* controls (Figure 8). This same pattern was reflected in mice treated with 0.015% ES9, though the measured changes in glucose tolerance, blood glucose levels, HbA1c and liver size were not statistically significant (Figure 8). Mice treated with 0.2% NTZ had no statistically significant phenotype, while higher dose 0.4% NTZ resulted in statistically significant improvements in gonadal fat weight, HbA1c and liver weight (Figure 8).

Treatment with NEN resulted in no significant changes compared to the *db/db* control except lower liver weight in mice treated with low dose 0.1% NEN (Figure 8). Although not statistically significant, mice treated with the higher dose of 0.25% NEN trended to have worse phenotypes in terms of glucose control than those treated with the lower dose (Figure 8). No statistically significant changes were observed in mice treated with OPC-163493 compared to *db/db* controls (Figure 8).

## 4 Discussion

Over the past 90 years, dozens of mitochondrial uncouplers have been discovered and characterized; however, their actions in cells and animals have been somewhat inconsistent. The inconsistency could be related to diverse activity profiles in cells, non-mitochondrial off-target activity, on-target complete mitochondrial depolarisation resulting in mitochondrial inhibition, differences in pharmacology, tissue distribution, and/or drug metabolism. The observed differences in compound action may also be due to a lack of standardisation in the methods employed to characterise them.

In this study, we compared 15 mitochondrial uncouplers head-to-head in an *in vitro* activity screen to identify those with the broadest dosing range and then tested the *in vivo* efficacy of those with suitable drug exposure and tolerability in the *db/db* model of diabetes and obesity. To establish a standardised assay for comparing the uncouplers *in vitro*, we first identified a cell line with robust mitochondrial spare respiratory capacity to maximise the potential differences in OCR readout during treatment. CHO–K1 cells were found to have the highest mitochondrial spare respiratory capacity and thus were used in the subsequent activity screen. FCCP is widely used as a probe in mitochondrial bioenergetics studies. However, BAM15 outperformed FCCP and stimulated a statistically higher MaxOCR than FCCP in seven cell lines from diverse lineages. These results demonstrate that FCCP use in mitochondrial bioenergetics studies can lead to under-estimation of mitochondrial spare respiratory capacity and challenge the use of FCCP as a chemical tool for studying mitochondrial function. Conversely, our studies highlight the utility of using BAM15 over FCCP as a prototype chemical probe. The utility of BAM15 was particularly evident in cell lines with low spare respiratory capacity. For example, FCCP treatment of RAW 264.7, HEK293 and HeLa cells led to a rapid decline in OCR to sub-basal levels, while BAM15 was able to identify their small but measurable spare respiratory capacity.

The *in vitro* dose-tolerance range of a mitochondrial uncoupler can be estimated by the concentration range over which the compound effectively enhances respiration before inducing a decline in basal respiration that is indicative of mitochondrial exhaustion or inhibition. A wide effective concentration range is desirable in molecules being considered for therapeutic development as it maximises the dosing range between the effective dose and one that is potentially toxic to mitochondria. FCCP and TTFB stimulated Max_OCR_ at concentrations as low as 1 μM in CHO–K1 cells. However, higher concentrations induced a decline in OCR that plateaued to sub-basal levels indicating toxicity and a narrow effective concentration range that curtails therapeutic potential. UsAc, Ppc-1, ELL, SR4, sorafenib and C_12_TPP had minimal or no uncoupling activity in CHO–K1 cells over the 120-minute assay. Thus, we found that less than half of the molecules tested had a broad effective concentration range *in vitro*. Of the 15 mitochondrial uncouplers tested, BAM15, ES9, DNP and OPC-163493, were the only molecules that sustained enhanced OCR over the tested concentration range. However, DNP and OPC-163493 were unable to stimulate Max_OCR_ as high as BAM15. TyrA9 stimulated Max_OCR_ at low concentrations and maintained enhanced OCR at concentrations ≤100 μM. NEN and NTZ induced milder uncoupling but did so at low concentrations and were well tolerated at higher concentrations with OCR maintained at basal levels. Notably, the self-limiting assay demonstrates that for 11 of the 15 mitochondrial uncouplers tested, the inability to stimulate or sustain Max_OCR_ at high concentrations is a result of the molecules impairing mitochondrial respiration as opposed to low efficacy. The *in vitro* OCR data highlights the diverse range of activity in terms of Max_OCR_ and potency in terms of EC_50_ across a series of molecules that are all commonly termed mitochondrial uncouplers.

The molecules with the broadest effective range were then assessed for pharmacokinetics, tolerability, and *in vivo* efficacy in mice. However, DNP and TyrA9 were not further investigated due to their lack of efficacy in rodent models of obesity or well-characterised systemic toxicity [6,34]. BAM15, ES9, OPC-163493, NEN and NTZ had drug exposure and tolerability suitable for testing *in vivo*. We assessed the therapeutic potential of these molecules by determining their efficacy in terms of improving metabolic parameters in *db/db* mice which are widely considered a gold-standard model of type 2 diabetes with a phenotype of severe obesity, hyperglycaemia and dyslipidaemia due to a spontaneous mutation of leptin receptor (Lepr^db^) which in homozygous *db/db* mice leads to hyperphagia [28]. We tested two different doses of each mitochondrial uncoupler and benchmarked their efficacy by comparing to a lifestyle intervention of 35% calorie restriction as well as to *db/+* lean metabolically healthy mice. No treatment resulted in observed adverse effects nor did any negatively alter liver or kidney function enzymes. However, each mitochondrial uncoupler produced a unique phenotype with regard to body weight, glucose homeostasis, and liver adiposity, with some molecules worsening disease phenotypes and others improving them.

BAM15 treatment generally outperformed the other molecules by dose-dependently improving body weight, glucose homeostasis and liver triglyceride content. These results echo those produced previously in younger male and female *db/db* mice, where 0.2% BAM15 treatment had similar positive effects on body weight, glucose tolerance, and steatohepatitis [20,21]. The previous studies utilised doses of 0.1% and 0.2% BAM15, in this study we investigated the effects of an intermediate dose of 0.15% BAM15 and showed that this dose outperforms the 0.1% BAM15 treatment with respect to improving glucose tolerance and lowering fed and fasted blood glucose [20,21]. However, like the 0.1% BAM15 treatment in previous studies, 0.15% BAM15 treatment was not sufficient to replicate the beneficial effects of 0.2% BAM15 treatment on body weight.

In the *db/db* study, three of the five uncouplers normalised food intake towards that of *db/*+ mice, these were BAM15, NEN, and NTZ. The normalisation of food intake for BAM15-fed mice was unexpected because BAM15 had no impact on food intake in wild type C57BL/6 mice fed high fat diet or in male *db/db* mice fed 0.2% BAM15 from 5 to 10 weeks of age [19,21]. However, in this study the mice were older male *db/db* mice fed BAM15 from 8 to 13 weeks of age. One potential explanation for the normalisation of food intake in this study compared to past studies may be the increased age of the mice, which are beyond the adolescent growth phase. Other mitochondria-targeting compounds with uncoupling action such as C_12_TPP, the antioxidant mitoquinone (mitoQ, C_10_TPP-CoQ), and C_4_R1 (a butyl ester of Rhodamine 19) have also been shown to significantly decrease food intake in HFD-fed C57BL/6 mice [22,38,39]. Future studies are required to investigate whether BAM15, NEN and NTZ regulate food intake in the *db/db* model via neuroendocrine actions or alterations in hypothalamic signalling.

The mice fed BAM15 ate a similar amount of food as the *db/+* controls (65% of what *db/db* mice ate) and therefore a calorie-restricted group of *db/db* mice was matched for the same food intake to assess how much of the metabolic phenotype was due to food intake. This comparison revealed that mice fed BAM15 had significant improvements in body weight, glucose tolerance, fasted blood glucose and HbA1c that were not replicated in the calorie-restricted group, suggesting that these effects were driven by the action of BAM15 and not by decreased caloric intake alone. Additionally, BAM15 treatment was the only intervention which statistically decreased fat pad weight and maintained or statistically increased the raw weight of gastrocnemius muscle in this study. This data is particularly important because the preservation of lean mass is critical in long-term weight loss treatment [40,41]. Reduction in lean mass is not only detrimental in that it increases the risk of sarcopenia but because skeletal muscle has a critical role in regulating insulin sensitivity and glucose homeostasis [42]. Notably, reductions in lean mass have been reported in several of the registration trials for the GLP-1 receptor agonists which have gained approval for chronic weight management. Specifically, semaglutide has been associated with loss of lean mass of up to 40% of total weight lost and liraglutide with up to 60% [43,44].

The only other uncoupler treatment that resulted in significant improvements in glucose homeostasis was high dose ES9. Treatment with ES9 dose-dependently improved glucose tolerance, lowered fed/fasted blood glucose and lowered HbA1c compared to the *db/db* control mice, with the high dose of ES9 yielding statistically significant results. Interestingly, mice treated with ES9 also had a somewhat paradoxical and unexpected phenotype of increased weight gain with significantly larger livers and higher total liver triglycerides and cholesterol compared to *db/db* mice. It is possible that these effects are a product of liver enzyme induction or drug-induced liver injury; however, the plasma markers of liver tissue damage assessed in this study were not significantly elevated in ES9-treated mice. Little is known about the mechanisms underpinning ES9 action - this study is the first to publish *in vivo* data from mammals on the compound, with previous studies only performed in *Arabidopsis thaliana* cells and *Drosophila melanogaster* larvae [45]. Besides its activity as a mitochondrial uncoupler, ES9 has been characterised as a clathrin-mediated endocytosis (CME) inhibitor [45]. Hepatocellular endocytosis is responsible for the internalisation of lipids and is important in the maintenance of normal serum lipid levels as well as lipid droplet formation and degradation [46]. Endocytosis controls which lipids are internalised at the plasma membrane and thus influences both the formation and the function of lipid droplets. Moreover, lipid droplets have been shown to undergo autophagic degradation that is at least in part dependent on endocytosis to facilitate trafficking to the lysosome [47]. It has been shown that pharmacological inhibition of autophagy using 3-methyladenine, a compound which also interferes with endocytosis, results in an increase in the number and size of lipid droplets in cultured hepatocytes [48]. Thus, we hypothesise that through its action as an endocytosis inhibitor, ES9 may be blocking or reducing the rate of hepatocellular lipid droplet degradation by preventing trafficking of lipid droplets to the lysosome, resulting in accumulation and retention of lipids as was observed in the livers of ES9-treated *db/db* mice in this study. Endocytosis-facilitated autophagy is also an important process in the pancreas, with basal levels of autophagy required for maintenance of beta cells and inductive autophagy shown to be important in the adaptive response of beta cells to insulin resistance induced by high-fat diet [49,50]. Given the importance of autophagy in beta-cell maintenance, it is interesting that ES9-treated mice did not appear to have perturbed serum insulin levels and that they had improved glucose homeostasis. One potential explanation is that ES9 may distribute largely to the liver, with concentrations of ES9 at the pancreas being too low to facilitate inhibition of endocytosis or significantly impact autophagy. Future investigations should aim to further characterise ES9, both in terms of its tissue localisation and to uncover the mechanism underlying the observed steatohepatitis. In contrast to ES9, mice treated with high dose BAM15, had significantly less fat mass and lower total body weight at the end of treatment, as well as significantly lower total liver triglycerides. Considering the lack of steatohepatitis observed in BAM15 and other uncoupler-treated mice, it seems likely that this phenotype is mediated by an off-target effect of ES9 and not due directly to its action as a mitochondrial uncoupler.

Niclosamide and NTZ are FDA-approved anti-helminthic drugs that have been shown to have mitochondrial uncoupling action [51]. Niclosamide ethanolamine (NEN) has been shown to increase oxygen consumption *in vitro* and to increase energy expenditure in mice fed a high-fat diet [23]. One study also found that 0.15% NEN admixed in diet improved blood glucose levels in male *db/db* mice [23]. However, other studies in both male and female *db/db* mice using the same dose were unable to replicate these effects, with no significant improvements in blood glucose control [20,21,24]. In this study, we tested both a lower and higher dose of NEN and similarly saw no significant effect on any metabolic parameter tested, except improved liver weight in the 0.1% NEN treated mice. In fact, the higher dose of NEN worsened glucose tolerance and HbA1c relative to the lower dose and the *db/db* control group, though this effect was not statistically significant. When considering the dose response curve of NEN in CHO–K1 cells, we see that the compound has greater efficacy at lower concentrations, with uncoupling activity lost at higher doses. This data suggests that investigating a lower dose of NEN could potentially be more effective in terms of yielding greater uncoupling and potentially improving metabolic parameters by inducing a more significant negative energy balance. Similar to NEN, neither dose of NTZ yielded statistically significant improvements to body weight or blood glucose control, except for improved HbA1c in mice treated with 0.4% NTZ. Additionally, mice treated with 0.4% NTZ had significantly smaller livers than *db/db* control mice, a trend that has also been seen in studies which have shown NTZ has antifibrotic effects in mouse models of liver-fibrosis and diet-induced NASH [52,53]. Given that mice treated with 0.4% NTZ had improved HbA1c scores and liver size, future studies should include an intervention group with higher dose NEN treatment to determine if dose escalation could yield further improvements to glucose control or liver steatosis. However, dose escalation should proceed with caution as a toxicology study conducted in rats found increased salivation and lethargy, as well as increased liver and spleen size at doses ≥450 mg/kg/day (mouse equivalent dose of ∼900 mg/kg/day) [54]. In our study the average daily dose of NTZ received by mice in the 0.4% NTZ group was ∼530 mg/kg/day, thus our dose is within only 2-fold of the estimated adverse effect level.

OPC-163493 is a liver-localised mitochondrial uncoupler that has been shown to have significant anti-diabetic effects (decreased HbA_1c_ and lowered fasting blood glucose) in Zucker diabetic fatty rats, Akita mice, and Otsuka Long-Evans Tokushima Fatty rats at doses as low as 2 mg/kg/day [16]. In our study, neither treatment with 0.01% or 0.02% OPC-163493 (approximately 20–40 mg/kg/day) had any significant effects on body weight, blood glucose control or liver steatosis in male *db/db* mice. In fact, we observed a slight trend for worsened glucose control and increased body weight gain. This trend could in part be attributed to the marginally increased food intake of mice treated with OPC-163493, with previous work by Kanemoto et al. finding that in *ob/ob* mice doses exceeding 0.02% resulted in significant hyperphagia [16].

This study has made significant strides in highlighting the importance of characterising uncouplers using standardised methods and within the same model system to effectively compare their therapeutic potential. The CHO–K1 cell line was chosen for this study based on its high mitochondrial spare respiratory capacity. The *db/db* mouse model was used in this study as it closely resembles a severe form of human metabolic disease [28]. *Db/db* mice are homozygous for the diabetes spontaneous mutation (Lepr^db^), which leads to significant hyperphagia [28]. However, the metabolic derangements in this model cannot be corrected by restoring normal food intake, as evidenced in this study by the calorie-restricted controls. Future studies could utilise diet-induced obese mice to better compare the effects of uncouplers on appetite and body composition. Future studies should also aim to investigate the molecular mechanisms underpinning the effects of BAM15 and ES9, in particular to distinguish them from the other uncouplers and calorie restriction which showed little or no effect in the *db/db* model, as well as to better understand the opposing effects of the two uncouplers on liver steatosis.

The relationship between mitochondrial uncoupling and body weight involves complex systems biology where white adipose tissue physiology including adipokine release (e.g., leptin, adiponectin), the hypothalamic-WAT axis, immune system crosstalk (e.g., macrophage infiltration), and other factors play an important role. The goal of the present study was to benchmark uncouplers head-to-head versus calorie restriction and it was beyond the scope of the study to investigate all potential reasons for phenotypic discrepancies. For example, a limitation of our study is that we did not directly assess adipokines or immune responses. The *db/db* mouse is an excellent model for severe obesity-related metabolic disease, but it is characterized by leptin resistance by definition due to leptin receptor deficiency. Therefore, the *db/db* model does not fully reflect diet-induced obesity or human physiology. Future studies in high-fat diet models are necessary to better understand the actions of diverse mitochondrial uncouplers in broader obesity contexts.

In addition to increasing energy expenditure, another therapeutically relevant phenotype induced by mitochondrial uncoupler treatment is decreased ROS production. Most relevant to this study is that increased mitochondrial ROS is implicated in the pathogenesis of insulin resistance and is reversible *in vitro* and in mice by small molecule antioxidants, SOD2 overexpression, and mitochondrial uncouplers [55,56]. Uncoupling has been proposed to decrease mitochondrial ROS production by lowering oxygen tension, creating a more oxidised state of the electron transport chain, and preventing reverse electron transport [[57], [58], [59], [60]]. BAM15 is previously reported to increase reduced glutathione by > 2-fold (without a change in oxidised glutathione) in liver tissue of mice fed high fat diet [18]. Therefore, future studies aimed at discerning the mechanisms whereby BAM15 outperforms other uncouplers should assess mitochondrial ROS.

Although the sample size used in our study of 6–9 mice per group) provided sufficient statistical power for resolving many differences, future studies with larger group sizes could provide insight into the subtler differences or trends observed between the treatment groups. Future studies should also be conducted in female mice to account for sexual dimorphism and increase translational relevance. Despite these acknowledged limitations, the results provide a strong foundation for investigating clinical applications of mitochondrial uncouplers.

Finally, the *in vivo* study phenotypes relate to the whole animal and have not been traced to mitochondria or specific cell types. The pharmacokinetics of compounds, their actions in different organs, and the varying degrees of glycolytic vs oxidative metabolism for energy production among cell types are all important questions that remain to be addressed. Off-target actions for each compound also remain incompletely studied as some compounds will be more or less likely to affect proton transport or symport processes across non-mitochondrial membranes, and the on-target actions are equally important that may be influenced by lipophilicity and ability to penetrate membranes, or pKa and ability to act as a weak acid in different cellular compartments that have different pH and lipid content. For example, where all other parameters may be considered equal, a molecule with a low pKa would be more likely to fully depolarise mitochondria and result in mitochondrial inhibition than a molecule with a higher pKa, which could be more concerning in organs such as the heart and brain that are primarily oxidative. Therefore, this study represents a big picture overview comparing the therapeutic potential of diverse mitochondrial uncouplers and mechanistic underpinnings of the diverse phenotypes require further elucidation.

Overall, our study demonstrates the unique behaviour of different mitochondrial uncouplers and highlights the importance of head-to-head comparison within the same model system to identify the best candidates for therapeutic development. BAM15 and high dose ES9 were the most effective molecules for improving glucose tolerance, blood glucose levels and HbA1c in *db/db* mice. However, ES9 treatment yielded undesirable effects on the liver, with significantly increased steatosis and a trend for increased body weight and adiposity. In contrast, high dose BAM15 treatment was the only intervention to prevent significant weight gain and lower liver triglycerides. Importantly, the *in vivo* data showed that no compound elevated markers of liver or kidney injury despite chronic treatment for 4 weeks at biologically active doses. In summary, this study reveals that mitochondrial uncoupler drug development is a niche and nuanced field where each uncoupler molecule needs to be considered by its own actions in well-defined experimental conditions *in vitro* and *in vivo*.

## CRediT authorship contribution statement

**Divya P. Shah:** Writing – review & editing, Writing – original draft, Project administration, Methodology, Investigation, Formal analysis, Data curation, Conceptualization. **Calum S. Vancuylenburg:** Investigation. **Ellen M. Olzomer:** Investigation. **Sing-Young Chen:** Writing – review & editing, Methodology, Conceptualization. **Robert J. Grams:** Writing – review & editing, Resources. **Martina Beretta:** Methodology, Investigation. **Frances L. Byrne:** Writing – review & editing. **Webster L. Santos:** Writing – review & editing, Resources. **Kyle L. Hoehn:** Writing – review & editing, Supervision, Funding acquisition, Conceptualization.

## Declaration of competing interest

This study utilised BAM15 provided by Life Biosciences. K.L.H. and W.L.S. declare a commercial interest in Life Biosciences and Uncoupler Biosciences. No other potential conflicts of interest relevant to this article were reported.

## Data Availability

Data will be made available on request.
