# Supplementary material for: Diverse actions of 15 structurally unrelated mitochondrial uncouplers in cells and mice
Source: Mol Metab. 2025 Jul 8;99:102204. doi: 10.1016/j.molmet.2025.102204 (PMC12303080; doi:10.1016/j.molmet.2025.102204)
Supplement: Multimedia component 1 [file mmc1.docx]

Supplementary Information

Supplementary Figure 1. Effect of BAM15 or FCCP over a wide concentration range in CHO-K1, MCF7, HEK293T, HeLa, NIH-3T3, RAW 264.7, NMuLi, and L6 cells*.* a-h) OCR is presented as a percentage of basal. Data points represent the mean OCR over 24 minutes post injection of BAM15 or FCCP. The dashed line indicates basal respiration, defined as the mean OCR over the first 24 minutes of the assay. Error bars show SEM, n = 3 per condition from three separate experiments. Statistical significance was determined by two-way ANOVA with Sidak’s correction for multiple comparisons. *(p<0.05) indicates a statistically significant difference between OCR stimulated by BAM15 vs FCCP.

Supplementary Figure 2. Effect of uncouplers on OCR in CHO-K1 cells over time. a-g) OCR of CHO-K1 cells is presented as a percentage of basal. Uncouplers at concentrations of a) 0.32 µM, b) 1 µM, c) 3.2 µM, d) 10 µM, e) 32 µM, f) 100 µM, and g) 200 µM were injected at 24 minutes, with OCR measured over a total of 132 minutes. The dashed line indicates basal respiration, defined as the mean OCR over the first 24 minutes of the assay. Data points are the mean OCR at each time point. Error bars show SEM, n = 3 per condition from three separate experiments, except BAM15 n = 5 per condition from 5 separate experiments.

Supplementary Figure 3. Pharmacokinetic properties of uncouplers in C57BL/6J mice*.* a) BAM15, b) NEN, c) OPC-163493, d) ES9, and e) TIZ. Male C57BL/6J mice were given 10 mg/kg of the indicated mitochondrial uncoupler by oral gavage (98% methylcellulose (0.7%), 2% Tween-80). Plasma concentration of mitochondrial uncouplers at indicated timepoints was determined by LC-MS/MS. Error bars show SEM, *n* = 3 per mitochondrial uncoupler.


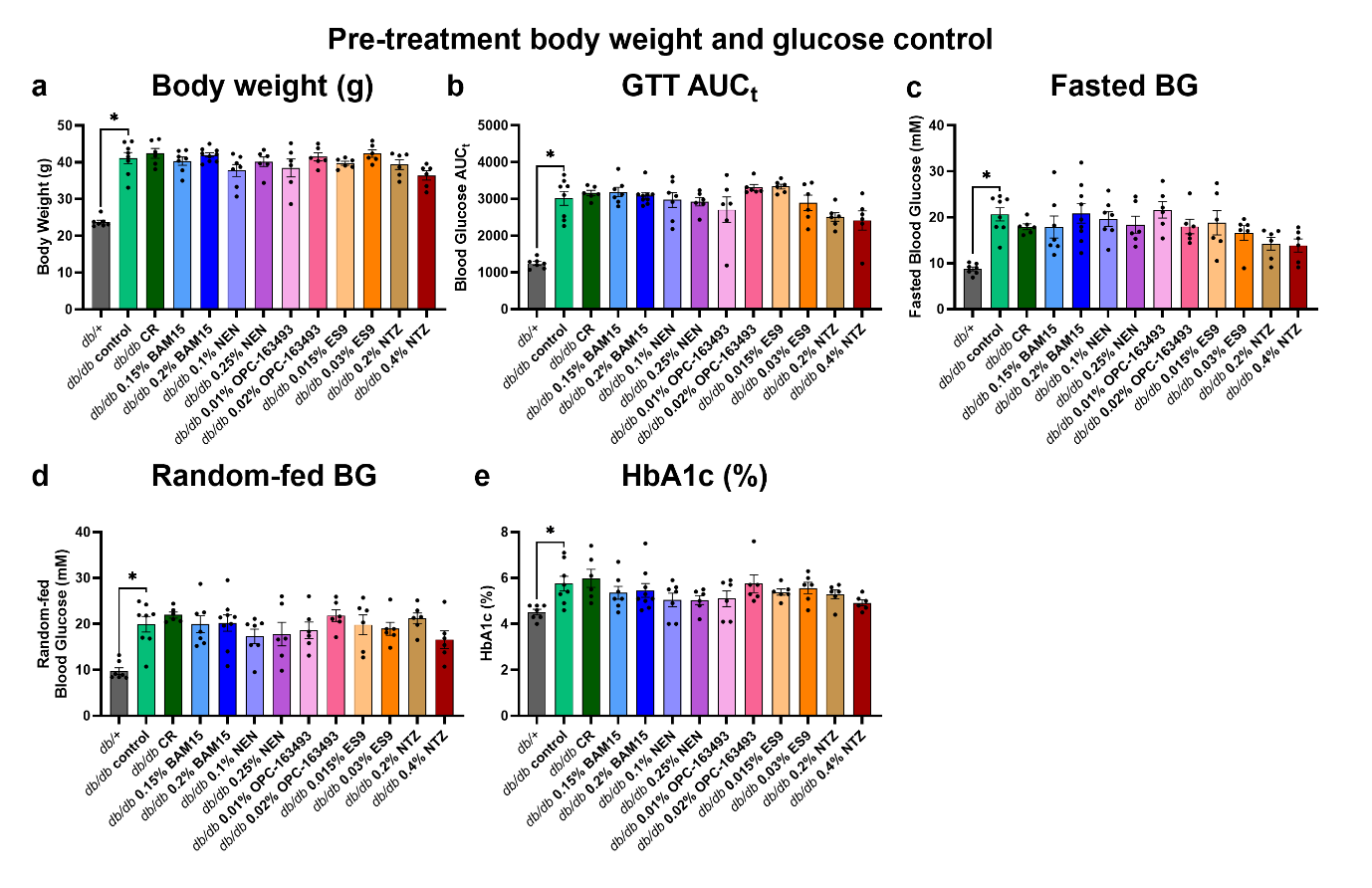


Supplementary Figure 4. Baseline body weight and glucose tolerance in *db/db* mice prior to study commencement. a) Body weight at baseline. b) AUC of baseline GTT. c) Fasted blood glucose at baseline. d) Random-fed blood glucose at baseline. e) HbA1c (%) at baseline. Graphs show mean $\boldsymbol{\pm}$ SEM, *n* = 6-9 per group. * indicates p<0.05 as assessed by one-way ANOVA with all groups compared to *db/db* control.

Supplementary Figure 5. Pre-treatment and post-treatment glucose tolerance, HbA1c and blood glucose in *db/db* mice. a) GTT AUC. b) HbA1c (%). c) fasted blood glucose. d) random-fed blood glucose. Graphs show mean $\boldsymbol{\pm}$ SEM, *n* = 6-9 per group. * indicates p<0.05 as assessed by one-way ANOVA with all groups compared to *db/db* control.

**Supplementary Figure 6.** Effect of uncouplers on maximal mitochondrial respiration in CHO-K1 cells in the absence or presence of oligomycin. a)-o) OCR of CHO-K1 cells is presented as a percentage of basal over time. The dashed line indicates basal respiration, defined as the mean OCR over the first 24 minutes of the assay prior to the addition of uncoupler. At ~24 minutes, 1 μM of oligomycin or vehicle was injected, followed by the injection of the indicated uncoupler or vehicle at ~44 minutes, and 0.5 μM Rotenone / Antimycin A at ~88 minutes. Error bars show SEM, n = 3 per condition from three separate experiments for all uncouplers and vehicles.

Supplementary Table 1. Raw oxygen levels in CHO-K1 cells treated with BAM15 or FCCP measured using the Seahorse XF96 Analyzer. Indicated dose of BAM15 or FCCP was injected following measurement three for each well.

| **Measurement** | **Tick** | **Well** | **Group** | **TimeStamp** | **O2 (mmHg)** |
| --- | --- | --- | --- | --- | --- |
| 1 | 0 | B01 | 0.5 µM BAM15 | 00:40:06 | 141.83 |
| 1 | 1 | B01 | 0.5 µM BAM15 | 00:40:20 | 137.98 |
| 1 | 2 | B01 | 0.5 µM BAM15 | 00:40:34 | 136.33 |
| 1 | 3 | B01 | 0.5 µM BAM15 | 00:40:48 | 134.91 |
| 1 | 4 | B01 | 0.5 µM BAM15 | 00:41:02 | 133.60 |
| 1 | 5 | B01 | 0.5 µM BAM15 | 00:41:17 | 132.52 |
| 1 | 6 | B01 | 0.5 µM BAM15 | 00:41:31 | 131.49 |
| 1 | 7 | B01 | 0.5 µM BAM15 | 00:41:45 | 130.43 |
| 1 | 8 | B01 | 0.5 µM BAM15 | 00:41:59 | 129.50 |
| 1 | 9 | B01 | 0.5 µM BAM15 | 00:42:13 | 128.56 |
| 1 | 10 | B01 | 0.5 µM BAM15 | 00:42:28 | 127.67 |
| 1 | 11 | B01 | 0.5 µM BAM15 | 00:42:42 | 126.83 |
| 1 | 12 | B01 | 0.5 µM BAM15 | 00:42:56 | 125.93 |
| 1 | 13 | B01 | 0.5 µM BAM15 | 00:43:10 | 125.14 |
| 2 | 14 | B01 | 0.5 µM BAM15 | 00:48:43 | 142.20 |
| 2 | 15 | B01 | 0.5 µM BAM15 | 00:48:57 | 137.74 |
| 2 | 16 | B01 | 0.5 µM BAM15 | 00:49:12 | 135.90 |
| 2 | 17 | B01 | 0.5 µM BAM15 | 00:49:26 | 134.34 |
| 2 | 18 | B01 | 0.5 µM BAM15 | 00:49:40 | 133.11 |
| 2 | 19 | B01 | 0.5 µM BAM15 | 00:49:54 | 131.84 |
| 2 | 20 | B01 | 0.5 µM BAM15 | 00:50:08 | 130.82 |
| 2 | 21 | B01 | 0.5 µM BAM15 | 00:50:22 | 129.74 |
| 2 | 22 | B01 | 0.5 µM BAM15 | 00:50:37 | 128.83 |
| 2 | 23 | B01 | 0.5 µM BAM15 | 00:50:51 | 127.91 |
| 2 | 24 | B01 | 0.5 µM BAM15 | 00:51:05 | 126.93 |
| 2 | 25 | B01 | 0.5 µM BAM15 | 00:51:19 | 126.09 |
| 2 | 26 | B01 | 0.5 µM BAM15 | 00:51:33 | 125.14 |
| 2 | 27 | B01 | 0.5 µM BAM15 | 00:51:48 | 124.43 |
| 3 | 28 | B01 | 0.5 µM BAM15 | 00:57:21 | 141.68 |
| 3 | 29 | B01 | 0.5 µM BAM15 | 00:57:35 | 137.41 |
| 3 | 30 | B01 | 0.5 µM BAM15 | 00:57:49 | 135.50 |
| 3 | 31 | B01 | 0.5 µM BAM15 | 00:58:03 | 133.95 |
| 3 | 32 | B01 | 0.5 µM BAM15 | 00:58:17 | 132.71 |
| 3 | 33 | B01 | 0.5 µM BAM15 | 00:58:31 | 131.52 |
| 3 | 34 | B01 | 0.5 µM BAM15 | 00:58:46 | 130.48 |
| 3 | 35 | B01 | 0.5 µM BAM15 | 00:59:00 | 129.37 |
| 3 | 36 | B01 | 0.5 µM BAM15 | 00:59:14 | 128.23 |
| 3 | 37 | B01 | 0.5 µM BAM15 | 00:59:28 | 127.44 |
| 3 | 38 | B01 | 0.5 µM BAM15 | 00:59:42 | 126.52 |
| 3 | 39 | B01 | 0.5 µM BAM15 | 00:59:56 | 125.69 |
| 3 | 40 | B01 | 0.5 µM BAM15 | 01:00:11 | 124.85 |
| 3 | 41 | B01 | 0.5 µM BAM15 | 01:00:25 | 124.00 |
| 4 | 42 | B01 | 0.5 µM BAM15 | 01:06:04 | 136.17 |
| 4 | 43 | B01 | 0.5 µM BAM15 | 01:06:18 | 123.33 |
| 4 | 44 | B01 | 0.5 µM BAM15 | 01:06:33 | 117.67 |
| 4 | 45 | B01 | 0.5 µM BAM15 | 01:06:47 | 113.40 |
| 4 | 46 | B01 | 0.5 µM BAM15 | 01:07:01 | 109.79 |
| 4 | 47 | B01 | 0.5 µM BAM15 | 01:07:15 | 106.33 |
| 4 | 48 | B01 | 0.5 µM BAM15 | 01:07:29 | 103.18 |
| 4 | 49 | B01 | 0.5 µM BAM15 | 01:07:43 | 100.25 |
| 4 | 50 | B01 | 0.5 µM BAM15 | 01:07:58 | 97.65 |
| 4 | 51 | B01 | 0.5 µM BAM15 | 01:08:12 | 95.10 |
| 4 | 52 | B01 | 0.5 µM BAM15 | 01:08:26 | 92.69 |
| 4 | 53 | B01 | 0.5 µM BAM15 | 01:08:40 | 90.46 |
| 4 | 54 | B01 | 0.5 µM BAM15 | 01:08:54 | 88.31 |
| 4 | 55 | B01 | 0.5 µM BAM15 | 01:09:09 | 86.33 |
| 5 | 56 | B01 | 0.5 µM BAM15 | 01:14:42 | 130.26 |
| 5 | 57 | B01 | 0.5 µM BAM15 | 01:14:56 | 115.39 |
| 5 | 58 | B01 | 0.5 µM BAM15 | 01:15:10 | 109.89 |
| 5 | 59 | B01 | 0.5 µM BAM15 | 01:15:25 | 105.77 |
| 5 | 60 | B01 | 0.5 µM BAM15 | 01:15:39 | 102.20 |
| 5 | 61 | B01 | 0.5 µM BAM15 | 01:15:53 | 98.92 |
| 5 | 62 | B01 | 0.5 µM BAM15 | 01:16:07 | 96.02 |
| 5 | 63 | B01 | 0.5 µM BAM15 | 01:16:21 | 93.24 |
| 5 | 64 | B01 | 0.5 µM BAM15 | 01:16:35 | 90.81 |
| 5 | 65 | B01 | 0.5 µM BAM15 | 01:16:50 | 88.42 |
| 5 | 66 | B01 | 0.5 µM BAM15 | 01:17:04 | 86.12 |
| 5 | 67 | B01 | 0.5 µM BAM15 | 01:17:18 | 84.01 |
| 5 | 68 | B01 | 0.5 µM BAM15 | 01:17:32 | 81.96 |
| 5 | 69 | B01 | 0.5 µM BAM15 | 01:17:46 | 80.11 |
| 6 | 70 | B01 | 0.5 µM BAM15 | 01:23:20 | 127.33 |
| 6 | 71 | B01 | 0.5 µM BAM15 | 01:23:34 | 112.34 |
| 6 | 72 | B01 | 0.5 µM BAM15 | 01:23:48 | 107.05 |
| 6 | 73 | B01 | 0.5 µM BAM15 | 01:24:02 | 102.98 |
| 6 | 74 | B01 | 0.5 µM BAM15 | 01:24:16 | 99.61 |
| 6 | 75 | B01 | 0.5 µM BAM15 | 01:24:30 | 96.38 |
| 6 | 76 | B01 | 0.5 µM BAM15 | 01:24:44 | 93.66 |
| 6 | 77 | B01 | 0.5 µM BAM15 | 01:24:59 | 90.93 |
| 6 | 78 | B01 | 0.5 µM BAM15 | 01:25:13 | 88.48 |
| 6 | 79 | B01 | 0.5 µM BAM15 | 01:25:27 | 86.16 |
| 6 | 80 | B01 | 0.5 µM BAM15 | 01:25:41 | 83.94 |
| 6 | 81 | B01 | 0.5 µM BAM15 | 01:25:55 | 81.90 |
| 6 | 82 | B01 | 0.5 µM BAM15 | 01:26:10 | 79.86 |
| 6 | 83 | B01 | 0.5 µM BAM15 | 01:26:24 | 78.01 |
| 7 | 84 | B01 | 0.5 µM BAM15 | 01:31:58 | 125.59 |
| 7 | 85 | B01 | 0.5 µM BAM15 | 01:32:12 | 111.00 |
| 7 | 86 | B01 | 0.5 µM BAM15 | 01:32:26 | 105.72 |
| 7 | 87 | B01 | 0.5 µM BAM15 | 01:32:40 | 101.89 |
| 7 | 88 | B01 | 0.5 µM BAM15 | 01:32:54 | 98.49 |
| 7 | 89 | B01 | 0.5 µM BAM15 | 01:33:08 | 95.44 |
| 7 | 90 | B01 | 0.5 µM BAM15 | 01:33:23 | 92.65 |
| 7 | 91 | B01 | 0.5 µM BAM15 | 01:33:37 | 90.02 |
| 7 | 92 | B01 | 0.5 µM BAM15 | 01:33:51 | 87.61 |
| 7 | 93 | B01 | 0.5 µM BAM15 | 01:34:05 | 85.32 |
| 7 | 94 | B01 | 0.5 µM BAM15 | 01:34:19 | 83.13 |
| 7 | 95 | B01 | 0.5 µM BAM15 | 01:34:33 | 81.14 |
| 7 | 96 | B01 | 0.5 µM BAM15 | 01:34:48 | 79.21 |
| 7 | 97 | B01 | 0.5 µM BAM15 | 01:35:02 | 77.34 |
| 8 | 98 | B01 | 0.5 µM BAM15 | 01:40:36 | 125.22 |
| 8 | 99 | B01 | 0.5 µM BAM15 | 01:40:50 | 111.30 |
| 8 | 100 | B01 | 0.5 µM BAM15 | 01:41:04 | 106.09 |
| 8 | 101 | B01 | 0.5 µM BAM15 | 01:41:18 | 102.21 |
| 8 | 102 | B01 | 0.5 µM BAM15 | 01:41:32 | 98.84 |
| 8 | 103 | B01 | 0.5 µM BAM15 | 01:41:46 | 95.82 |
| 8 | 104 | B01 | 0.5 µM BAM15 | 01:42:00 | 93.00 |
| 8 | 105 | B01 | 0.5 µM BAM15 | 01:42:15 | 90.45 |
| 8 | 106 | B01 | 0.5 µM BAM15 | 01:42:29 | 87.99 |
| 8 | 107 | B01 | 0.5 µM BAM15 | 01:42:43 | 85.67 |
| 8 | 108 | B01 | 0.5 µM BAM15 | 01:42:57 | 83.53 |
| 8 | 109 | B01 | 0.5 µM BAM15 | 01:43:11 | 81.50 |
| 8 | 110 | B01 | 0.5 µM BAM15 | 01:43:26 | 79.50 |
| 8 | 111 | B01 | 0.5 µM BAM15 | 01:43:40 | 77.72 |
| 9 | 112 | B01 | 0.5 µM BAM15 | 01:49:14 | 128.76 |
| 9 | 113 | B01 | 0.5 µM BAM15 | 01:49:28 | 118.05 |
| 9 | 114 | B01 | 0.5 µM BAM15 | 01:49:42 | 112.93 |
| 9 | 115 | B01 | 0.5 µM BAM15 | 01:49:56 | 108.80 |
| 9 | 116 | B01 | 0.5 µM BAM15 | 01:50:10 | 105.17 |
| 9 | 117 | B01 | 0.5 µM BAM15 | 01:50:24 | 101.99 |
| 9 | 118 | B01 | 0.5 µM BAM15 | 01:50:38 | 99.01 |
| 9 | 119 | B01 | 0.5 µM BAM15 | 01:50:53 | 96.20 |
| 9 | 120 | B01 | 0.5 µM BAM15 | 01:51:07 | 93.53 |
| 9 | 121 | B01 | 0.5 µM BAM15 | 01:51:21 | 91.08 |
| 9 | 122 | B01 | 0.5 µM BAM15 | 01:51:35 | 88.81 |
| 9 | 123 | B01 | 0.5 µM BAM15 | 01:51:50 | 86.57 |
| 9 | 124 | B01 | 0.5 µM BAM15 | 01:52:04 | 84.49 |
| 9 | 125 | B01 | 0.5 µM BAM15 | 01:52:18 | 82.44 |
| 10 | 126 | B01 | 0.5 µM BAM15 | 01:57:52 | 128.71 |
| 10 | 127 | B01 | 0.5 µM BAM15 | 01:58:06 | 117.64 |
| 10 | 128 | B01 | 0.5 µM BAM15 | 01:58:20 | 112.51 |
| 10 | 129 | B01 | 0.5 µM BAM15 | 01:58:34 | 108.58 |
| 10 | 130 | B01 | 0.5 µM BAM15 | 01:58:48 | 105.09 |
| 10 | 131 | B01 | 0.5 µM BAM15 | 01:59:03 | 101.86 |
| 10 | 132 | B01 | 0.5 µM BAM15 | 01:59:17 | 99.00 |
| 10 | 133 | B01 | 0.5 µM BAM15 | 01:59:31 | 96.20 |
| 10 | 134 | B01 | 0.5 µM BAM15 | 01:59:45 | 93.75 |
| 10 | 135 | B01 | 0.5 µM BAM15 | 01:59:59 | 91.31 |
| 10 | 136 | B01 | 0.5 µM BAM15 | 02:00:14 | 89.06 |
| 10 | 137 | B01 | 0.5 µM BAM15 | 02:00:28 | 86.97 |
| 10 | 138 | B01 | 0.5 µM BAM15 | 02:00:42 | 84.83 |
| 10 | 139 | B01 | 0.5 µM BAM15 | 02:00:56 | 82.93 |
| 11 | 140 | B01 | 0.5 µM BAM15 | 02:06:30 | 129.43 |
| 11 | 141 | B01 | 0.5 µM BAM15 | 02:06:44 | 118.65 |
| 11 | 142 | B01 | 0.5 µM BAM15 | 02:06:58 | 113.62 |
| 11 | 143 | B01 | 0.5 µM BAM15 | 02:07:12 | 109.59 |
| 11 | 144 | B01 | 0.5 µM BAM15 | 02:07:26 | 106.22 |
| 11 | 145 | B01 | 0.5 µM BAM15 | 02:07:41 | 102.99 |
| 11 | 146 | B01 | 0.5 µM BAM15 | 02:07:55 | 100.11 |
| 11 | 147 | B01 | 0.5 µM BAM15 | 02:08:09 | 97.35 |
| 11 | 148 | B01 | 0.5 µM BAM15 | 02:08:23 | 94.86 |
| 11 | 149 | B01 | 0.5 µM BAM15 | 02:08:37 | 92.49 |
| 11 | 150 | B01 | 0.5 µM BAM15 | 02:08:52 | 90.23 |
| 11 | 151 | B01 | 0.5 µM BAM15 | 02:09:06 | 88.06 |
| 11 | 152 | B01 | 0.5 µM BAM15 | 02:09:20 | 85.99 |
| 11 | 153 | B01 | 0.5 µM BAM15 | 02:09:34 | 84.03 |
| 12 | 154 | B01 | 0.5 µM BAM15 | 02:15:08 | 129.63 |
| 12 | 155 | B01 | 0.5 µM BAM15 | 02:15:22 | 118.92 |
| 12 | 156 | B01 | 0.5 µM BAM15 | 02:15:37 | 113.72 |
| 12 | 157 | B01 | 0.5 µM BAM15 | 02:15:53 | 109.40 |
| 12 | 158 | B01 | 0.5 µM BAM15 | 02:16:08 | 105.87 |
| 12 | 159 | B01 | 0.5 µM BAM15 | 02:16:22 | 102.94 |
| 12 | 160 | B01 | 0.5 µM BAM15 | 02:16:36 | 100.05 |
| 12 | 161 | B01 | 0.5 µM BAM15 | 02:16:50 | 97.44 |
| 12 | 162 | B01 | 0.5 µM BAM15 | 02:17:04 | 94.99 |
| 12 | 163 | B01 | 0.5 µM BAM15 | 02:17:18 | 92.63 |
| 12 | 164 | B01 | 0.5 µM BAM15 | 02:17:33 | 90.44 |
| 12 | 165 | B01 | 0.5 µM BAM15 | 02:17:47 | 88.33 |
| 12 | 166 | B01 | 0.5 µM BAM15 | 02:18:01 | 86.33 |
| 13 | 167 | B01 | 0.5 µM BAM15 | 02:23:39 | 126.06 |
| 13 | 168 | B01 | 0.5 µM BAM15 | 02:23:54 | 117.78 |
| 13 | 169 | B01 | 0.5 µM BAM15 | 02:24:08 | 113.30 |
| 13 | 170 | B01 | 0.5 µM BAM15 | 02:24:22 | 109.56 |
| 13 | 171 | B01 | 0.5 µM BAM15 | 02:24:36 | 106.24 |
| 13 | 172 | B01 | 0.5 µM BAM15 | 02:24:51 | 103.07 |
| 13 | 173 | B01 | 0.5 µM BAM15 | 02:25:07 | 99.97 |
| 13 | 174 | B01 | 0.5 µM BAM15 | 02:25:22 | 97.28 |
| 13 | 175 | B01 | 0.5 µM BAM15 | 02:25:36 | 95.03 |
| 13 | 176 | B01 | 0.5 µM BAM15 | 02:25:50 | 92.69 |
| 13 | 177 | B01 | 0.5 µM BAM15 | 02:26:04 | 90.52 |
| 13 | 178 | B01 | 0.5 µM BAM15 | 02:26:18 | 88.45 |
| 13 | 179 | B01 | 0.5 µM BAM15 | 02:26:33 | 86.49 |
| 14 | 180 | B01 | 0.5 µM BAM15 | 02:32:07 | 129.90 |
| 14 | 181 | B01 | 0.5 µM BAM15 | 02:32:21 | 119.42 |
| 14 | 182 | B01 | 0.5 µM BAM15 | 02:32:35 | 114.60 |
| 14 | 183 | B01 | 0.5 µM BAM15 | 02:32:50 | 110.81 |
| 14 | 184 | B01 | 0.5 µM BAM15 | 02:33:04 | 107.54 |
| 14 | 185 | B01 | 0.5 µM BAM15 | 02:33:18 | 104.49 |
| 14 | 186 | B01 | 0.5 µM BAM15 | 02:33:32 | 101.64 |
| 14 | 187 | B01 | 0.5 µM BAM15 | 02:33:46 | 99.00 |
| 14 | 188 | B01 | 0.5 µM BAM15 | 02:34:01 | 96.59 |
| 14 | 189 | B01 | 0.5 µM BAM15 | 02:34:15 | 94.34 |
| 14 | 190 | B01 | 0.5 µM BAM15 | 02:34:29 | 92.12 |
| 14 | 191 | B01 | 0.5 µM BAM15 | 02:34:43 | 90.05 |
| 14 | 192 | B01 | 0.5 µM BAM15 | 02:34:57 | 87.94 |
| 14 | 193 | B01 | 0.5 µM BAM15 | 02:35:12 | 86.15 |
| 15 | 194 | B01 | 0.5 µM BAM15 | 02:40:46 | 130.51 |
| 15 | 195 | B01 | 0.5 µM BAM15 | 02:41:00 | 120.31 |
| 15 | 196 | B01 | 0.5 µM BAM15 | 02:41:14 | 115.34 |
| 15 | 197 | B01 | 0.5 µM BAM15 | 02:41:28 | 111.59 |
| 15 | 198 | B01 | 0.5 µM BAM15 | 02:41:43 | 108.28 |
| 15 | 199 | B01 | 0.5 µM BAM15 | 02:41:57 | 105.29 |
| 15 | 200 | B01 | 0.5 µM BAM15 | 02:42:11 | 102.53 |
| 15 | 201 | B01 | 0.5 µM BAM15 | 02:42:25 | 99.94 |
| 15 | 202 | B01 | 0.5 µM BAM15 | 02:42:39 | 97.57 |
| 15 | 203 | B01 | 0.5 µM BAM15 | 02:42:53 | 95.30 |
| 15 | 204 | B01 | 0.5 µM BAM15 | 02:43:08 | 93.04 |
| 15 | 205 | B01 | 0.5 µM BAM15 | 02:43:22 | 91.03 |
| 15 | 206 | B01 | 0.5 µM BAM15 | 02:43:36 | 88.99 |
| 15 | 207 | B01 | 0.5 µM BAM15 | 02:43:50 | 87.15 |
| 16 | 208 | B01 | 0.5 µM BAM15 | 02:49:25 | 130.69 |
| 16 | 209 | B01 | 0.5 µM BAM15 | 02:49:40 | 120.63 |
| 16 | 210 | B01 | 0.5 µM BAM15 | 02:49:54 | 115.76 |
| 16 | 211 | B01 | 0.5 µM BAM15 | 02:50:08 | 112.02 |
| 16 | 212 | B01 | 0.5 µM BAM15 | 02:50:22 | 108.85 |
| 16 | 213 | B01 | 0.5 µM BAM15 | 02:50:36 | 105.77 |
| 16 | 214 | B01 | 0.5 µM BAM15 | 02:50:50 | 103.06 |
| 16 | 215 | B01 | 0.5 µM BAM15 | 02:51:05 | 100.57 |
| 16 | 216 | B01 | 0.5 µM BAM15 | 02:51:19 | 98.16 |
| 16 | 217 | B01 | 0.5 µM BAM15 | 02:51:33 | 95.80 |
| 16 | 218 | B01 | 0.5 µM BAM15 | 02:51:47 | 93.73 |
| 16 | 219 | B01 | 0.5 µM BAM15 | 02:52:01 | 91.69 |
| 16 | 220 | B01 | 0.5 µM BAM15 | 02:52:16 | 89.67 |
| 16 | 221 | B01 | 0.5 µM BAM15 | 02:52:30 | 87.79 |
| 1 | 0 | B02 | 0.5 µM BAM15 | 00:40:06 | 138.05 |
| 1 | 1 | B02 | 0.5 µM BAM15 | 00:40:20 | 133.41 |
| 1 | 2 | B02 | 0.5 µM BAM15 | 00:40:34 | 131.20 |
| 1 | 3 | B02 | 0.5 µM BAM15 | 00:40:48 | 129.32 |
| 1 | 4 | B02 | 0.5 µM BAM15 | 00:41:02 | 127.74 |
| 1 | 5 | B02 | 0.5 µM BAM15 | 00:41:17 | 126.18 |
| 1 | 6 | B02 | 0.5 µM BAM15 | 00:41:31 | 124.89 |
| 1 | 7 | B02 | 0.5 µM BAM15 | 00:41:45 | 123.62 |
| 1 | 8 | B02 | 0.5 µM BAM15 | 00:41:59 | 122.34 |
| 1 | 9 | B02 | 0.5 µM BAM15 | 00:42:13 | 121.20 |
| 1 | 10 | B02 | 0.5 µM BAM15 | 00:42:28 | 119.99 |
| 1 | 11 | B02 | 0.5 µM BAM15 | 00:42:42 | 118.93 |
| 1 | 12 | B02 | 0.5 µM BAM15 | 00:42:56 | 117.90 |
| 1 | 13 | B02 | 0.5 µM BAM15 | 00:43:10 | 116.85 |
| 2 | 14 | B02 | 0.5 µM BAM15 | 00:48:43 | 139.26 |
| 2 | 15 | B02 | 0.5 µM BAM15 | 00:48:57 | 134.20 |
| 2 | 16 | B02 | 0.5 µM BAM15 | 00:49:12 | 131.88 |
| 2 | 17 | B02 | 0.5 µM BAM15 | 00:49:26 | 129.89 |
| 2 | 18 | B02 | 0.5 µM BAM15 | 00:49:40 | 128.30 |
| 2 | 19 | B02 | 0.5 µM BAM15 | 00:49:54 | 126.69 |
| 2 | 20 | B02 | 0.5 µM BAM15 | 00:50:08 | 125.35 |
| 2 | 21 | B02 | 0.5 µM BAM15 | 00:50:22 | 123.96 |
| 2 | 22 | B02 | 0.5 µM BAM15 | 00:50:37 | 122.79 |
| 2 | 23 | B02 | 0.5 µM BAM15 | 00:50:51 | 121.66 |
| 2 | 24 | B02 | 0.5 µM BAM15 | 00:51:05 | 120.42 |
| 2 | 25 | B02 | 0.5 µM BAM15 | 00:51:19 | 119.40 |
| 2 | 26 | B02 | 0.5 µM BAM15 | 00:51:33 | 118.27 |
| 2 | 27 | B02 | 0.5 µM BAM15 | 00:51:48 | 117.35 |
| 3 | 28 | B02 | 0.5 µM BAM15 | 00:57:21 | 139.62 |
| 3 | 29 | B02 | 0.5 µM BAM15 | 00:57:35 | 134.64 |
| 3 | 30 | B02 | 0.5 µM BAM15 | 00:57:49 | 132.28 |
| 3 | 31 | B02 | 0.5 µM BAM15 | 00:58:03 | 130.39 |
| 3 | 32 | B02 | 0.5 µM BAM15 | 00:58:17 | 128.79 |
| 3 | 33 | B02 | 0.5 µM BAM15 | 00:58:31 | 127.19 |
| 3 | 34 | B02 | 0.5 µM BAM15 | 00:58:46 | 125.82 |
| 3 | 35 | B02 | 0.5 µM BAM15 | 00:59:00 | 124.46 |
| 3 | 36 | B02 | 0.5 µM BAM15 | 00:59:14 | 123.15 |
| 3 | 37 | B02 | 0.5 µM BAM15 | 00:59:28 | 122.02 |
| 3 | 38 | B02 | 0.5 µM BAM15 | 00:59:42 | 120.82 |
| 3 | 39 | B02 | 0.5 µM BAM15 | 00:59:56 | 119.81 |
| 3 | 40 | B02 | 0.5 µM BAM15 | 01:00:11 | 118.80 |
| 3 | 41 | B02 | 0.5 µM BAM15 | 01:00:25 | 117.75 |
| 4 | 42 | B02 | 0.5 µM BAM15 | 01:06:04 | 133.90 |
| 4 | 43 | B02 | 0.5 µM BAM15 | 01:06:18 | 118.69 |
| 4 | 44 | B02 | 0.5 µM BAM15 | 01:06:33 | 111.82 |
| 4 | 45 | B02 | 0.5 µM BAM15 | 01:06:47 | 106.40 |
| 4 | 46 | B02 | 0.5 µM BAM15 | 01:07:01 | 101.86 |
| 4 | 47 | B02 | 0.5 µM BAM15 | 01:07:15 | 97.54 |
| 4 | 48 | B02 | 0.5 µM BAM15 | 01:07:29 | 93.81 |
| 4 | 49 | B02 | 0.5 µM BAM15 | 01:07:43 | 90.11 |
| 4 | 50 | B02 | 0.5 µM BAM15 | 01:07:58 | 86.92 |
| 4 | 51 | B02 | 0.5 µM BAM15 | 01:08:12 | 83.81 |
| 4 | 52 | B02 | 0.5 µM BAM15 | 01:08:26 | 80.81 |
| 4 | 53 | B02 | 0.5 µM BAM15 | 01:08:40 | 78.11 |
| 4 | 54 | B02 | 0.5 µM BAM15 | 01:08:54 | 75.46 |
| 4 | 55 | B02 | 0.5 µM BAM15 | 01:09:09 | 73.01 |
| 5 | 56 | B02 | 0.5 µM BAM15 | 01:14:42 | 127.84 |
| 5 | 57 | B02 | 0.5 µM BAM15 | 01:14:56 | 110.63 |
| 5 | 58 | B02 | 0.5 µM BAM15 | 01:15:10 | 103.63 |
| 5 | 59 | B02 | 0.5 µM BAM15 | 01:15:25 | 98.36 |
| 5 | 60 | B02 | 0.5 µM BAM15 | 01:15:39 | 93.92 |
| 5 | 61 | B02 | 0.5 µM BAM15 | 01:15:53 | 89.75 |
| 5 | 62 | B02 | 0.5 µM BAM15 | 01:16:07 | 86.11 |
| 5 | 63 | B02 | 0.5 µM BAM15 | 01:16:21 | 82.64 |
| 5 | 64 | B02 | 0.5 µM BAM15 | 01:16:35 | 79.51 |
| 5 | 65 | B02 | 0.5 µM BAM15 | 01:16:50 | 76.54 |
| 5 | 66 | B02 | 0.5 µM BAM15 | 01:17:04 | 73.72 |
| 5 | 67 | B02 | 0.5 µM BAM15 | 01:17:18 | 71.05 |
| 5 | 68 | B02 | 0.5 µM BAM15 | 01:17:32 | 68.52 |
| 5 | 69 | B02 | 0.5 µM BAM15 | 01:17:46 | 66.10 |
| 6 | 70 | B02 | 0.5 µM BAM15 | 01:23:20 | 124.98 |
| 6 | 71 | B02 | 0.5 µM BAM15 | 01:23:34 | 107.56 |
| 6 | 72 | B02 | 0.5 µM BAM15 | 01:23:48 | 100.81 |
| 6 | 73 | B02 | 0.5 µM BAM15 | 01:24:02 | 95.63 |
| 6 | 74 | B02 | 0.5 µM BAM15 | 01:24:16 | 91.29 |
| 6 | 75 | B02 | 0.5 µM BAM15 | 01:24:30 | 87.22 |
| 6 | 76 | B02 | 0.5 µM BAM15 | 01:24:44 | 83.72 |
| 6 | 77 | B02 | 0.5 µM BAM15 | 01:24:59 | 80.30 |
| 6 | 78 | B02 | 0.5 µM BAM15 | 01:25:13 | 77.24 |
| 6 | 79 | B02 | 0.5 µM BAM15 | 01:25:27 | 74.32 |
| 6 | 80 | B02 | 0.5 µM BAM15 | 01:25:41 | 71.51 |
| 6 | 81 | B02 | 0.5 µM BAM15 | 01:25:55 | 68.96 |
| 6 | 82 | B02 | 0.5 µM BAM15 | 01:26:10 | 66.40 |
| 6 | 83 | B02 | 0.5 µM BAM15 | 01:26:24 | 64.06 |
| 7 | 84 | B02 | 0.5 µM BAM15 | 01:31:58 | 123.05 |
| 7 | 85 | B02 | 0.5 µM BAM15 | 01:32:12 | 106.20 |
| 7 | 86 | B02 | 0.5 µM BAM15 | 01:32:26 | 99.50 |
| 7 | 87 | B02 | 0.5 µM BAM15 | 01:32:40 | 94.57 |
| 7 | 88 | B02 | 0.5 µM BAM15 | 01:32:54 | 90.27 |
| 7 | 89 | B02 | 0.5 µM BAM15 | 01:33:08 | 86.35 |
| 7 | 90 | B02 | 0.5 µM BAM15 | 01:33:23 | 82.80 |
| 7 | 91 | B02 | 0.5 µM BAM15 | 01:33:37 | 79.50 |
| 7 | 92 | B02 | 0.5 µM BAM15 | 01:33:51 | 76.44 |
| 7 | 93 | B02 | 0.5 µM BAM15 | 01:34:05 | 73.62 |
| 7 | 94 | B02 | 0.5 µM BAM15 | 01:34:19 | 70.84 |
| 7 | 95 | B02 | 0.5 µM BAM15 | 01:34:33 | 68.35 |
| 7 | 96 | B02 | 0.5 µM BAM15 | 01:34:48 | 65.89 |
| 7 | 97 | B02 | 0.5 µM BAM15 | 01:35:02 | 63.56 |
| 8 | 98 | B02 | 0.5 µM BAM15 | 01:40:36 | 122.35 |
| 8 | 99 | B02 | 0.5 µM BAM15 | 01:40:50 | 105.79 |
| 8 | 100 | B02 | 0.5 µM BAM15 | 01:41:04 | 99.21 |
| 8 | 101 | B02 | 0.5 µM BAM15 | 01:41:18 | 94.26 |
| 8 | 102 | B02 | 0.5 µM BAM15 | 01:41:32 | 90.07 |
| 8 | 103 | B02 | 0.5 µM BAM15 | 01:41:46 | 86.31 |
| 8 | 104 | B02 | 0.5 µM BAM15 | 01:42:00 | 82.79 |
| 8 | 105 | B02 | 0.5 µM BAM15 | 01:42:15 | 79.51 |
| 8 | 106 | B02 | 0.5 µM BAM15 | 01:42:29 | 76.51 |
| 8 | 107 | B02 | 0.5 µM BAM15 | 01:42:43 | 73.66 |
| 8 | 108 | B02 | 0.5 µM BAM15 | 01:42:57 | 70.97 |
| 8 | 109 | B02 | 0.5 µM BAM15 | 01:43:11 | 68.44 |
| 8 | 110 | B02 | 0.5 µM BAM15 | 01:43:26 | 65.98 |
| 8 | 111 | B02 | 0.5 µM BAM15 | 01:43:40 | 63.74 |
| 9 | 112 | B02 | 0.5 µM BAM15 | 01:49:14 | 122.17 |
| 9 | 113 | B02 | 0.5 µM BAM15 | 01:49:28 | 106.06 |
| 9 | 114 | B02 | 0.5 µM BAM15 | 01:49:42 | 99.62 |
| 9 | 115 | B02 | 0.5 µM BAM15 | 01:49:56 | 94.74 |
| 9 | 116 | B02 | 0.5 µM BAM15 | 01:50:10 | 90.56 |
| 9 | 117 | B02 | 0.5 µM BAM15 | 01:50:24 | 86.82 |
| 9 | 118 | B02 | 0.5 µM BAM15 | 01:50:38 | 83.34 |
| 9 | 119 | B02 | 0.5 µM BAM15 | 01:50:53 | 80.12 |
| 9 | 120 | B02 | 0.5 µM BAM15 | 01:51:07 | 77.11 |
| 9 | 121 | B02 | 0.5 µM BAM15 | 01:51:21 | 74.31 |
| 9 | 122 | B02 | 0.5 µM BAM15 | 01:51:35 | 71.61 |
| 9 | 123 | B02 | 0.5 µM BAM15 | 01:51:50 | 69.10 |
| 9 | 124 | B02 | 0.5 µM BAM15 | 01:52:04 | 66.68 |
| 9 | 125 | B02 | 0.5 µM BAM15 | 01:52:18 | 64.38 |
| 10 | 126 | B02 | 0.5 µM BAM15 | 01:57:52 | 124.66 |
| 10 | 127 | B02 | 0.5 µM BAM15 | 01:58:06 | 112.52 |
| 10 | 128 | B02 | 0.5 µM BAM15 | 01:58:20 | 106.14 |
| 10 | 129 | B02 | 0.5 µM BAM15 | 01:58:34 | 101.20 |
| 10 | 130 | B02 | 0.5 µM BAM15 | 01:58:48 | 96.81 |
| 10 | 131 | B02 | 0.5 µM BAM15 | 01:59:03 | 92.74 |
| 10 | 132 | B02 | 0.5 µM BAM15 | 01:59:17 | 89.09 |
| 10 | 133 | B02 | 0.5 µM BAM15 | 01:59:31 | 85.62 |
| 10 | 134 | B02 | 0.5 µM BAM15 | 01:59:45 | 82.61 |
| 10 | 135 | B02 | 0.5 µM BAM15 | 01:59:59 | 79.54 |
| 10 | 136 | B02 | 0.5 µM BAM15 | 02:00:14 | 76.70 |
| 10 | 137 | B02 | 0.5 µM BAM15 | 02:00:28 | 74.05 |
| 10 | 138 | B02 | 0.5 µM BAM15 | 02:00:42 | 71.46 |
| 10 | 139 | B02 | 0.5 µM BAM15 | 02:00:56 | 69.04 |
| 11 | 140 | B02 | 0.5 µM BAM15 | 02:06:30 | 126.07 |
| 11 | 141 | B02 | 0.5 µM BAM15 | 02:06:44 | 113.83 |
| 11 | 142 | B02 | 0.5 µM BAM15 | 02:06:58 | 107.53 |
| 11 | 143 | B02 | 0.5 µM BAM15 | 02:07:12 | 102.54 |
| 11 | 144 | B02 | 0.5 µM BAM15 | 02:07:26 | 98.21 |
| 11 | 145 | B02 | 0.5 µM BAM15 | 02:07:41 | 94.18 |
| 11 | 146 | B02 | 0.5 µM BAM15 | 02:07:55 | 90.57 |
| 11 | 147 | B02 | 0.5 µM BAM15 | 02:08:09 | 87.16 |
| 11 | 148 | B02 | 0.5 µM BAM15 | 02:08:23 | 84.01 |
| 11 | 149 | B02 | 0.5 µM BAM15 | 02:08:37 | 81.07 |
| 11 | 150 | B02 | 0.5 µM BAM15 | 02:08:52 | 78.29 |
| 11 | 151 | B02 | 0.5 µM BAM15 | 02:09:06 | 75.57 |
| 11 | 152 | B02 | 0.5 µM BAM15 | 02:09:20 | 73.09 |
| 11 | 153 | B02 | 0.5 µM BAM15 | 02:09:34 | 70.66 |
| 12 | 154 | B02 | 0.5 µM BAM15 | 02:15:08 | 126.48 |
| 12 | 155 | B02 | 0.5 µM BAM15 | 02:15:22 | 114.42 |
| 12 | 156 | B02 | 0.5 µM BAM15 | 02:15:37 | 107.82 |
| 12 | 157 | B02 | 0.5 µM BAM15 | 02:15:53 | 102.37 |
| 12 | 158 | B02 | 0.5 µM BAM15 | 02:16:08 | 98.00 |
| 12 | 159 | B02 | 0.5 µM BAM15 | 02:16:22 | 94.27 |
| 12 | 160 | B02 | 0.5 µM BAM15 | 02:16:36 | 90.70 |
| 12 | 161 | B02 | 0.5 µM BAM15 | 02:16:50 | 87.43 |
| 12 | 162 | B02 | 0.5 µM BAM15 | 02:17:04 | 84.36 |
| 12 | 163 | B02 | 0.5 µM BAM15 | 02:17:18 | 81.38 |
| 12 | 164 | B02 | 0.5 µM BAM15 | 02:17:33 | 78.69 |
| 12 | 165 | B02 | 0.5 µM BAM15 | 02:17:47 | 76.09 |
| 12 | 166 | B02 | 0.5 µM BAM15 | 02:18:01 | 73.57 |
| 13 | 167 | B02 | 0.5 µM BAM15 | 02:23:39 | 123.02 |
| 13 | 168 | B02 | 0.5 µM BAM15 | 02:23:54 | 113.36 |
| 13 | 169 | B02 | 0.5 µM BAM15 | 02:24:08 | 107.64 |
| 13 | 170 | B02 | 0.5 µM BAM15 | 02:24:22 | 102.93 |
| 13 | 171 | B02 | 0.5 µM BAM15 | 02:24:36 | 98.77 |
| 13 | 172 | B02 | 0.5 µM BAM15 | 02:24:51 | 94.78 |
| 13 | 173 | B02 | 0.5 µM BAM15 | 02:25:07 | 90.90 |
| 13 | 174 | B02 | 0.5 µM BAM15 | 02:25:22 | 87.58 |
| 13 | 175 | B02 | 0.5 µM BAM15 | 02:25:36 | 84.70 |
| 13 | 176 | B02 | 0.5 µM BAM15 | 02:25:50 | 81.81 |
| 13 | 177 | B02 | 0.5 µM BAM15 | 02:26:04 | 79.17 |
| 13 | 178 | B02 | 0.5 µM BAM15 | 02:26:18 | 76.57 |
| 13 | 179 | B02 | 0.5 µM BAM15 | 02:26:33 | 74.08 |
| 14 | 180 | B02 | 0.5 µM BAM15 | 02:32:07 | 127.49 |
| 14 | 181 | B02 | 0.5 µM BAM15 | 02:32:21 | 115.67 |
| 14 | 182 | B02 | 0.5 µM BAM15 | 02:32:35 | 109.62 |
| 14 | 183 | B02 | 0.5 µM BAM15 | 02:32:50 | 104.84 |
| 14 | 184 | B02 | 0.5 µM BAM15 | 02:33:04 | 100.60 |
| 14 | 185 | B02 | 0.5 µM BAM15 | 02:33:18 | 96.74 |
| 14 | 186 | B02 | 0.5 µM BAM15 | 02:33:32 | 93.27 |
| 14 | 187 | B02 | 0.5 µM BAM15 | 02:33:46 | 89.91 |
| 14 | 188 | B02 | 0.5 µM BAM15 | 02:34:01 | 86.94 |
| 14 | 189 | B02 | 0.5 µM BAM15 | 02:34:15 | 84.10 |
| 14 | 190 | B02 | 0.5 µM BAM15 | 02:34:29 | 81.34 |
| 14 | 191 | B02 | 0.5 µM BAM15 | 02:34:43 | 78.76 |
| 14 | 192 | B02 | 0.5 µM BAM15 | 02:34:57 | 76.21 |
| 14 | 193 | B02 | 0.5 µM BAM15 | 02:35:12 | 73.93 |
| 15 | 194 | B02 | 0.5 µM BAM15 | 02:40:46 | 127.89 |
| 15 | 195 | B02 | 0.5 µM BAM15 | 02:41:00 | 116.18 |
| 15 | 196 | B02 | 0.5 µM BAM15 | 02:41:14 | 110.13 |
| 15 | 197 | B02 | 0.5 µM BAM15 | 02:41:28 | 105.47 |
| 15 | 198 | B02 | 0.5 µM BAM15 | 02:41:43 | 101.28 |
| 15 | 199 | B02 | 0.5 µM BAM15 | 02:41:57 | 97.52 |
| 15 | 200 | B02 | 0.5 µM BAM15 | 02:42:11 | 94.06 |
| 15 | 201 | B02 | 0.5 µM BAM15 | 02:42:25 | 90.82 |
| 15 | 202 | B02 | 0.5 µM BAM15 | 02:42:39 | 87.83 |
| 15 | 203 | B02 | 0.5 µM BAM15 | 02:42:53 | 85.01 |
| 15 | 204 | B02 | 0.5 µM BAM15 | 02:43:08 | 82.29 |
| 15 | 205 | B02 | 0.5 µM BAM15 | 02:43:22 | 79.80 |
| 15 | 206 | B02 | 0.5 µM BAM15 | 02:43:36 | 77.26 |
| 15 | 207 | B02 | 0.5 µM BAM15 | 02:43:50 | 74.96 |
| 16 | 208 | B02 | 0.5 µM BAM15 | 02:49:25 | 128.33 |
| 16 | 209 | B02 | 0.5 µM BAM15 | 02:49:40 | 116.89 |
| 16 | 210 | B02 | 0.5 µM BAM15 | 02:49:54 | 110.91 |
| 16 | 211 | B02 | 0.5 µM BAM15 | 02:50:08 | 106.21 |
| 16 | 212 | B02 | 0.5 µM BAM15 | 02:50:22 | 102.14 |
| 16 | 213 | B02 | 0.5 µM BAM15 | 02:50:36 | 98.38 |
| 16 | 214 | B02 | 0.5 µM BAM15 | 02:50:50 | 94.98 |
| 16 | 215 | B02 | 0.5 µM BAM15 | 02:51:05 | 91.77 |
| 16 | 216 | B02 | 0.5 µM BAM15 | 02:51:19 | 88.76 |
| 16 | 217 | B02 | 0.5 µM BAM15 | 02:51:33 | 85.99 |
| 16 | 218 | B02 | 0.5 µM BAM15 | 02:51:47 | 83.38 |
| 16 | 219 | B02 | 0.5 µM BAM15 | 02:52:01 | 80.87 |
| 16 | 220 | B02 | 0.5 µM BAM15 | 02:52:16 | 78.39 |
| 16 | 221 | B02 | 0.5 µM BAM15 | 02:52:30 | 76.08 |
| 1 | 0 | B03 | 0.5 µM BAM15 | 00:40:06 | 134.13 |
| 1 | 1 | B03 | 0.5 µM BAM15 | 00:40:20 | 128.31 |
| 1 | 2 | B03 | 0.5 µM BAM15 | 00:40:34 | 126.02 |
| 1 | 3 | B03 | 0.5 µM BAM15 | 00:40:48 | 124.14 |
| 1 | 4 | B03 | 0.5 µM BAM15 | 00:41:02 | 122.44 |
| 1 | 5 | B03 | 0.5 µM BAM15 | 00:41:17 | 120.81 |
| 1 | 6 | B03 | 0.5 µM BAM15 | 00:41:31 | 119.38 |
| 1 | 7 | B03 | 0.5 µM BAM15 | 00:41:45 | 117.99 |
| 1 | 8 | B03 | 0.5 µM BAM15 | 00:41:59 | 116.68 |
| 1 | 9 | B03 | 0.5 µM BAM15 | 00:42:13 | 115.49 |
| 1 | 10 | B03 | 0.5 µM BAM15 | 00:42:28 | 114.14 |
| 1 | 11 | B03 | 0.5 µM BAM15 | 00:42:42 | 113.08 |
| 1 | 12 | B03 | 0.5 µM BAM15 | 00:42:56 | 111.82 |
| 1 | 13 | B03 | 0.5 µM BAM15 | 00:43:10 | 110.88 |
| 2 | 14 | B03 | 0.5 µM BAM15 | 00:48:43 | 135.12 |
| 2 | 15 | B03 | 0.5 µM BAM15 | 00:48:57 | 129.32 |
| 2 | 16 | B03 | 0.5 µM BAM15 | 00:49:12 | 126.96 |
| 2 | 17 | B03 | 0.5 µM BAM15 | 00:49:26 | 124.99 |
| 2 | 18 | B03 | 0.5 µM BAM15 | 00:49:40 | 123.33 |
| 2 | 19 | B03 | 0.5 µM BAM15 | 00:49:54 | 121.77 |
| 2 | 20 | B03 | 0.5 µM BAM15 | 00:50:08 | 120.36 |
| 2 | 21 | B03 | 0.5 µM BAM15 | 00:50:22 | 118.95 |
| 2 | 22 | B03 | 0.5 µM BAM15 | 00:50:37 | 117.65 |
| 2 | 23 | B03 | 0.5 µM BAM15 | 00:50:51 | 116.42 |
| 2 | 24 | B03 | 0.5 µM BAM15 | 00:51:05 | 115.29 |
| 2 | 25 | B03 | 0.5 µM BAM15 | 00:51:19 | 114.17 |
| 2 | 26 | B03 | 0.5 µM BAM15 | 00:51:33 | 112.95 |
| 2 | 27 | B03 | 0.5 µM BAM15 | 00:51:48 | 111.95 |
| 3 | 28 | B03 | 0.5 µM BAM15 | 00:57:21 | 135.57 |
| 3 | 29 | B03 | 0.5 µM BAM15 | 00:57:35 | 130.05 |
| 3 | 30 | B03 | 0.5 µM BAM15 | 00:57:49 | 127.72 |
| 3 | 31 | B03 | 0.5 µM BAM15 | 00:58:03 | 125.73 |
| 3 | 32 | B03 | 0.5 µM BAM15 | 00:58:17 | 124.10 |
| 3 | 33 | B03 | 0.5 µM BAM15 | 00:58:31 | 122.46 |
| 3 | 34 | B03 | 0.5 µM BAM15 | 00:58:46 | 121.07 |
| 3 | 35 | B03 | 0.5 µM BAM15 | 00:59:00 | 119.68 |
| 3 | 36 | B03 | 0.5 µM BAM15 | 00:59:14 | 118.30 |
| 3 | 37 | B03 | 0.5 µM BAM15 | 00:59:28 | 117.17 |
| 3 | 38 | B03 | 0.5 µM BAM15 | 00:59:42 | 115.91 |
| 3 | 39 | B03 | 0.5 µM BAM15 | 00:59:56 | 114.85 |
| 3 | 40 | B03 | 0.5 µM BAM15 | 01:00:11 | 113.71 |
| 3 | 41 | B03 | 0.5 µM BAM15 | 01:00:25 | 112.68 |
| 4 | 42 | B03 | 0.5 µM BAM15 | 01:06:04 | 129.99 |
| 4 | 43 | B03 | 0.5 µM BAM15 | 01:06:18 | 113.72 |
| 4 | 44 | B03 | 0.5 µM BAM15 | 01:06:33 | 106.51 |
| 4 | 45 | B03 | 0.5 µM BAM15 | 01:06:47 | 100.87 |
| 4 | 46 | B03 | 0.5 µM BAM15 | 01:07:01 | 96.20 |
| 4 | 47 | B03 | 0.5 µM BAM15 | 01:07:15 | 91.72 |
| 4 | 48 | B03 | 0.5 µM BAM15 | 01:07:29 | 87.69 |
| 4 | 49 | B03 | 0.5 µM BAM15 | 01:07:43 | 83.96 |
| 4 | 50 | B03 | 0.5 µM BAM15 | 01:07:58 | 80.56 |
| 4 | 51 | B03 | 0.5 µM BAM15 | 01:08:12 | 77.33 |
| 4 | 52 | B03 | 0.5 µM BAM15 | 01:08:26 | 74.17 |
| 4 | 53 | B03 | 0.5 µM BAM15 | 01:08:40 | 71.38 |
| 4 | 54 | B03 | 0.5 µM BAM15 | 01:08:54 | 68.51 |
| 4 | 55 | B03 | 0.5 µM BAM15 | 01:09:09 | 65.96 |
| 5 | 56 | B03 | 0.5 µM BAM15 | 01:14:42 | 123.67 |
| 5 | 57 | B03 | 0.5 µM BAM15 | 01:14:56 | 105.08 |
| 5 | 58 | B03 | 0.5 µM BAM15 | 01:15:10 | 97.72 |
| 5 | 59 | B03 | 0.5 µM BAM15 | 01:15:25 | 92.22 |
| 5 | 60 | B03 | 0.5 µM BAM15 | 01:15:39 | 87.47 |
| 5 | 61 | B03 | 0.5 µM BAM15 | 01:15:53 | 83.12 |
| 5 | 62 | B03 | 0.5 µM BAM15 | 01:16:07 | 79.18 |
| 5 | 63 | B03 | 0.5 µM BAM15 | 01:16:21 | 75.57 |
| 5 | 64 | B03 | 0.5 µM BAM15 | 01:16:35 | 72.26 |
| 5 | 65 | B03 | 0.5 µM BAM15 | 01:16:50 | 69.09 |
| 5 | 66 | B03 | 0.5 µM BAM15 | 01:17:04 | 66.05 |
| 5 | 67 | B03 | 0.5 µM BAM15 | 01:17:18 | 63.20 |
| 5 | 68 | B03 | 0.5 µM BAM15 | 01:17:32 | 60.54 |
| 5 | 69 | B03 | 0.5 µM BAM15 | 01:17:46 | 58.02 |
| 6 | 70 | B03 | 0.5 µM BAM15 | 01:23:20 | 120.89 |
| 6 | 71 | B03 | 0.5 µM BAM15 | 01:23:34 | 101.95 |
| 6 | 72 | B03 | 0.5 µM BAM15 | 01:23:48 | 94.80 |
| 6 | 73 | B03 | 0.5 µM BAM15 | 01:24:02 | 89.34 |
| 6 | 74 | B03 | 0.5 µM BAM15 | 01:24:16 | 84.72 |
| 6 | 75 | B03 | 0.5 µM BAM15 | 01:24:30 | 80.43 |
| 6 | 76 | B03 | 0.5 µM BAM15 | 01:24:44 | 76.66 |
| 6 | 77 | B03 | 0.5 µM BAM15 | 01:24:59 | 73.06 |
| 6 | 78 | B03 | 0.5 µM BAM15 | 01:25:13 | 69.78 |
| 6 | 79 | B03 | 0.5 µM BAM15 | 01:25:27 | 66.61 |
| 6 | 80 | B03 | 0.5 µM BAM15 | 01:25:41 | 63.67 |
| 6 | 81 | B03 | 0.5 µM BAM15 | 01:25:55 | 60.97 |
| 6 | 82 | B03 | 0.5 µM BAM15 | 01:26:10 | 58.18 |
| 6 | 83 | B03 | 0.5 µM BAM15 | 01:26:24 | 55.72 |
| 7 | 84 | B03 | 0.5 µM BAM15 | 01:31:58 | 119.34 |
| 7 | 85 | B03 | 0.5 µM BAM15 | 01:32:12 | 100.82 |
| 7 | 86 | B03 | 0.5 µM BAM15 | 01:32:26 | 93.72 |
| 7 | 87 | B03 | 0.5 µM BAM15 | 01:32:40 | 88.45 |
| 7 | 88 | B03 | 0.5 µM BAM15 | 01:32:54 | 83.84 |
| 7 | 89 | B03 | 0.5 µM BAM15 | 01:33:08 | 79.64 |
| 7 | 90 | B03 | 0.5 µM BAM15 | 01:33:23 | 75.94 |
| 7 | 91 | B03 | 0.5 µM BAM15 | 01:33:37 | 72.38 |
| 7 | 92 | B03 | 0.5 µM BAM15 | 01:33:51 | 69.16 |
| 7 | 93 | B03 | 0.5 µM BAM15 | 01:34:05 | 66.08 |
| 7 | 94 | B03 | 0.5 µM BAM15 | 01:34:19 | 63.17 |
| 7 | 95 | B03 | 0.5 µM BAM15 | 01:34:33 | 60.50 |
| 7 | 96 | B03 | 0.5 µM BAM15 | 01:34:48 | 57.86 |
| 7 | 97 | B03 | 0.5 µM BAM15 | 01:35:02 | 55.38 |
| 8 | 98 | B03 | 0.5 µM BAM15 | 01:40:36 | 118.89 |
| 8 | 99 | B03 | 0.5 µM BAM15 | 01:40:50 | 100.72 |
| 8 | 100 | B03 | 0.5 µM BAM15 | 01:41:04 | 93.75 |
| 8 | 101 | B03 | 0.5 µM BAM15 | 01:41:18 | 88.48 |
| 8 | 102 | B03 | 0.5 µM BAM15 | 01:41:32 | 84.00 |
| 8 | 103 | B03 | 0.5 µM BAM15 | 01:41:46 | 79.89 |
| 8 | 104 | B03 | 0.5 µM BAM15 | 01:42:00 | 76.20 |
| 8 | 105 | B03 | 0.5 µM BAM15 | 01:42:15 | 72.71 |
| 8 | 106 | B03 | 0.5 µM BAM15 | 01:42:29 | 69.49 |
| 8 | 107 | B03 | 0.5 µM BAM15 | 01:42:43 | 66.49 |
| 8 | 108 | B03 | 0.5 µM BAM15 | 01:42:57 | 63.58 |
| 8 | 109 | B03 | 0.5 µM BAM15 | 01:43:11 | 60.88 |
| 8 | 110 | B03 | 0.5 µM BAM15 | 01:43:26 | 58.24 |
| 8 | 111 | B03 | 0.5 µM BAM15 | 01:43:40 | 55.87 |
| 9 | 112 | B03 | 0.5 µM BAM15 | 01:49:14 | 118.87 |
| 9 | 113 | B03 | 0.5 µM BAM15 | 01:49:28 | 101.57 |
| 9 | 114 | B03 | 0.5 µM BAM15 | 01:49:42 | 94.73 |
| 9 | 115 | B03 | 0.5 µM BAM15 | 01:49:56 | 89.53 |
| 9 | 116 | B03 | 0.5 µM BAM15 | 01:50:10 | 85.07 |
| 9 | 117 | B03 | 0.5 µM BAM15 | 01:50:24 | 81.02 |
| 9 | 118 | B03 | 0.5 µM BAM15 | 01:50:38 | 77.34 |
| 9 | 119 | B03 | 0.5 µM BAM15 | 01:50:53 | 73.81 |
| 9 | 120 | B03 | 0.5 µM BAM15 | 01:51:07 | 70.60 |
| 9 | 121 | B03 | 0.5 µM BAM15 | 01:51:21 | 67.56 |
| 9 | 122 | B03 | 0.5 µM BAM15 | 01:51:35 | 64.70 |
| 9 | 123 | B03 | 0.5 µM BAM15 | 01:51:50 | 62.01 |
| 9 | 124 | B03 | 0.5 µM BAM15 | 01:52:04 | 59.40 |
| 9 | 125 | B03 | 0.5 µM BAM15 | 01:52:18 | 56.95 |
| 10 | 126 | B03 | 0.5 µM BAM15 | 01:57:52 | 119.94 |
| 10 | 127 | B03 | 0.5 µM BAM15 | 01:58:06 | 106.03 |
| 10 | 128 | B03 | 0.5 µM BAM15 | 01:58:20 | 99.57 |
| 10 | 129 | B03 | 0.5 µM BAM15 | 01:58:34 | 94.48 |
| 10 | 130 | B03 | 0.5 µM BAM15 | 01:58:48 | 89.97 |
| 10 | 131 | B03 | 0.5 µM BAM15 | 01:59:03 | 85.74 |
| 10 | 132 | B03 | 0.5 µM BAM15 | 01:59:17 | 81.96 |
| 10 | 133 | B03 | 0.5 µM BAM15 | 01:59:31 | 78.36 |
| 10 | 134 | B03 | 0.5 µM BAM15 | 01:59:45 | 75.15 |
| 10 | 135 | B03 | 0.5 µM BAM15 | 01:59:59 | 72.00 |
| 10 | 136 | B03 | 0.5 µM BAM15 | 02:00:14 | 68.99 |
| 10 | 137 | B03 | 0.5 µM BAM15 | 02:00:28 | 66.22 |
| 10 | 138 | B03 | 0.5 µM BAM15 | 02:00:42 | 63.54 |
| 10 | 139 | B03 | 0.5 µM BAM15 | 02:00:56 | 60.99 |
| 11 | 140 | B03 | 0.5 µM BAM15 | 02:06:30 | 120.97 |
| 11 | 141 | B03 | 0.5 µM BAM15 | 02:06:44 | 107.39 |
| 11 | 142 | B03 | 0.5 µM BAM15 | 02:06:58 | 101.07 |
| 11 | 143 | B03 | 0.5 µM BAM15 | 02:07:12 | 95.94 |
| 11 | 144 | B03 | 0.5 µM BAM15 | 02:07:26 | 91.56 |
| 11 | 145 | B03 | 0.5 µM BAM15 | 02:07:41 | 87.40 |
| 11 | 146 | B03 | 0.5 µM BAM15 | 02:07:55 | 83.70 |
| 11 | 147 | B03 | 0.5 µM BAM15 | 02:08:09 | 80.12 |
| 11 | 148 | B03 | 0.5 µM BAM15 | 02:08:23 | 76.89 |
| 11 | 149 | B03 | 0.5 µM BAM15 | 02:08:37 | 73.78 |
| 11 | 150 | B03 | 0.5 µM BAM15 | 02:08:52 | 70.87 |
| 11 | 151 | B03 | 0.5 µM BAM15 | 02:09:06 | 68.06 |
| 11 | 152 | B03 | 0.5 µM BAM15 | 02:09:20 | 65.38 |
| 11 | 153 | B03 | 0.5 µM BAM15 | 02:09:34 | 62.87 |
| 12 | 154 | B03 | 0.5 µM BAM15 | 02:15:08 | 121.46 |
| 12 | 155 | B03 | 0.5 µM BAM15 | 02:15:22 | 108.08 |
| 12 | 156 | B03 | 0.5 µM BAM15 | 02:15:37 | 101.61 |
| 12 | 157 | B03 | 0.5 µM BAM15 | 02:15:53 | 96.14 |
| 12 | 158 | B03 | 0.5 µM BAM15 | 02:16:08 | 91.61 |
| 12 | 159 | B03 | 0.5 µM BAM15 | 02:16:22 | 87.72 |
| 12 | 160 | B03 | 0.5 µM BAM15 | 02:16:36 | 84.04 |
| 12 | 161 | B03 | 0.5 µM BAM15 | 02:16:50 | 80.62 |
| 12 | 162 | B03 | 0.5 µM BAM15 | 02:17:04 | 77.43 |
| 12 | 163 | B03 | 0.5 µM BAM15 | 02:17:18 | 74.41 |
| 12 | 164 | B03 | 0.5 µM BAM15 | 02:17:33 | 71.56 |
| 12 | 165 | B03 | 0.5 µM BAM15 | 02:17:47 | 68.79 |
| 12 | 166 | B03 | 0.5 µM BAM15 | 02:18:01 | 66.20 |
| 13 | 167 | B03 | 0.5 µM BAM15 | 02:23:39 | 117.24 |
| 13 | 168 | B03 | 0.5 µM BAM15 | 02:23:54 | 107.18 |
| 13 | 169 | B03 | 0.5 µM BAM15 | 02:24:08 | 101.34 |
| 13 | 170 | B03 | 0.5 µM BAM15 | 02:24:22 | 96.64 |
| 13 | 171 | B03 | 0.5 µM BAM15 | 02:24:36 | 92.36 |
| 13 | 172 | B03 | 0.5 µM BAM15 | 02:24:51 | 88.32 |
| 13 | 173 | B03 | 0.5 µM BAM15 | 02:25:07 | 84.27 |
| 13 | 174 | B03 | 0.5 µM BAM15 | 02:25:22 | 80.84 |
| 13 | 175 | B03 | 0.5 µM BAM15 | 02:25:36 | 77.80 |
| 13 | 176 | B03 | 0.5 µM BAM15 | 02:25:50 | 74.80 |
| 13 | 177 | B03 | 0.5 µM BAM15 | 02:26:04 | 72.04 |
| 13 | 178 | B03 | 0.5 µM BAM15 | 02:26:18 | 69.33 |
| 13 | 179 | B03 | 0.5 µM BAM15 | 02:26:33 | 66.72 |
| 14 | 180 | B03 | 0.5 µM BAM15 | 02:32:07 | 122.51 |
| 14 | 181 | B03 | 0.5 µM BAM15 | 02:32:21 | 109.51 |
| 14 | 182 | B03 | 0.5 µM BAM15 | 02:32:35 | 103.48 |
| 14 | 183 | B03 | 0.5 µM BAM15 | 02:32:50 | 98.61 |
| 14 | 184 | B03 | 0.5 µM BAM15 | 02:33:04 | 94.38 |
| 14 | 185 | B03 | 0.5 µM BAM15 | 02:33:18 | 90.36 |
| 14 | 186 | B03 | 0.5 µM BAM15 | 02:33:32 | 86.77 |
| 14 | 187 | B03 | 0.5 µM BAM15 | 02:33:46 | 83.38 |
| 14 | 188 | B03 | 0.5 µM BAM15 | 02:34:01 | 80.23 |
| 14 | 189 | B03 | 0.5 µM BAM15 | 02:34:15 | 77.24 |
| 14 | 190 | B03 | 0.5 µM BAM15 | 02:34:29 | 74.44 |
| 14 | 191 | B03 | 0.5 µM BAM15 | 02:34:43 | 71.69 |
| 14 | 192 | B03 | 0.5 µM BAM15 | 02:34:57 | 69.06 |
| 14 | 193 | B03 | 0.5 µM BAM15 | 02:35:12 | 66.68 |
| 15 | 194 | B03 | 0.5 µM BAM15 | 02:40:46 | 122.86 |
| 15 | 195 | B03 | 0.5 µM BAM15 | 02:41:00 | 110.11 |
| 15 | 196 | B03 | 0.5 µM BAM15 | 02:41:14 | 104.10 |
| 15 | 197 | B03 | 0.5 µM BAM15 | 02:41:28 | 99.34 |
| 15 | 198 | B03 | 0.5 µM BAM15 | 02:41:43 | 95.08 |
| 15 | 199 | B03 | 0.5 µM BAM15 | 02:41:57 | 91.25 |
| 15 | 200 | B03 | 0.5 µM BAM15 | 02:42:11 | 87.74 |
| 15 | 201 | B03 | 0.5 µM BAM15 | 02:42:25 | 84.37 |
| 15 | 202 | B03 | 0.5 µM BAM15 | 02:42:39 | 81.27 |
| 15 | 203 | B03 | 0.5 µM BAM15 | 02:42:53 | 78.33 |
| 15 | 204 | B03 | 0.5 µM BAM15 | 02:43:08 | 75.48 |
| 15 | 205 | B03 | 0.5 µM BAM15 | 02:43:22 | 72.86 |
| 15 | 206 | B03 | 0.5 µM BAM15 | 02:43:36 | 70.23 |
| 15 | 207 | B03 | 0.5 µM BAM15 | 02:43:50 | 67.82 |
| 16 | 208 | B03 | 0.5 µM BAM15 | 02:49:25 | 123.11 |
| 16 | 209 | B03 | 0.5 µM BAM15 | 02:49:40 | 110.62 |
| 16 | 210 | B03 | 0.5 µM BAM15 | 02:49:54 | 104.70 |
| 16 | 211 | B03 | 0.5 µM BAM15 | 02:50:08 | 99.97 |
| 16 | 212 | B03 | 0.5 µM BAM15 | 02:50:22 | 95.87 |
| 16 | 213 | B03 | 0.5 µM BAM15 | 02:50:36 | 92.08 |
| 16 | 214 | B03 | 0.5 µM BAM15 | 02:50:50 | 88.54 |
| 16 | 215 | B03 | 0.5 µM BAM15 | 02:51:05 | 85.25 |
| 16 | 216 | B03 | 0.5 µM BAM15 | 02:51:19 | 82.16 |
| 16 | 217 | B03 | 0.5 µM BAM15 | 02:51:33 | 79.33 |
| 16 | 218 | B03 | 0.5 µM BAM15 | 02:51:47 | 76.54 |
| 16 | 219 | B03 | 0.5 µM BAM15 | 02:52:01 | 73.93 |
| 16 | 220 | B03 | 0.5 µM BAM15 | 02:52:16 | 71.35 |
| 16 | 221 | B03 | 0.5 µM BAM15 | 02:52:30 | 68.96 |
| 1 | 0 | B04 | 0.5 µM FCCP | 00:40:06 | 136.18 |
| 1 | 1 | B04 | 0.5 µM FCCP | 00:40:20 | 130.76 |
| 1 | 2 | B04 | 0.5 µM FCCP | 00:40:34 | 128.12 |
| 1 | 3 | B04 | 0.5 µM FCCP | 00:40:48 | 126.18 |
| 1 | 4 | B04 | 0.5 µM FCCP | 00:41:02 | 124.52 |
| 1 | 5 | B04 | 0.5 µM FCCP | 00:41:17 | 123.04 |
| 1 | 6 | B04 | 0.5 µM FCCP | 00:41:31 | 121.56 |
| 1 | 7 | B04 | 0.5 µM FCCP | 00:41:45 | 120.29 |
| 1 | 8 | B04 | 0.5 µM FCCP | 00:41:59 | 119.06 |
| 1 | 9 | B04 | 0.5 µM FCCP | 00:42:13 | 117.92 |
| 1 | 10 | B04 | 0.5 µM FCCP | 00:42:28 | 116.74 |
| 1 | 11 | B04 | 0.5 µM FCCP | 00:42:42 | 115.79 |
| 1 | 12 | B04 | 0.5 µM FCCP | 00:42:56 | 114.75 |
| 1 | 13 | B04 | 0.5 µM FCCP | 00:43:10 | 113.70 |
| 2 | 14 | B04 | 0.5 µM FCCP | 00:48:43 | 137.66 |
| 2 | 15 | B04 | 0.5 µM FCCP | 00:48:57 | 132.29 |
| 2 | 16 | B04 | 0.5 µM FCCP | 00:49:12 | 130.02 |
| 2 | 17 | B04 | 0.5 µM FCCP | 00:49:26 | 128.09 |
| 2 | 18 | B04 | 0.5 µM FCCP | 00:49:40 | 126.48 |
| 2 | 19 | B04 | 0.5 µM FCCP | 00:49:54 | 124.86 |
| 2 | 20 | B04 | 0.5 µM FCCP | 00:50:08 | 123.50 |
| 2 | 21 | B04 | 0.5 µM FCCP | 00:50:22 | 122.28 |
| 2 | 22 | B04 | 0.5 µM FCCP | 00:50:37 | 121.04 |
| 2 | 23 | B04 | 0.5 µM FCCP | 00:50:51 | 119.82 |
| 2 | 24 | B04 | 0.5 µM FCCP | 00:51:05 | 118.70 |
| 2 | 25 | B04 | 0.5 µM FCCP | 00:51:19 | 117.68 |
| 2 | 26 | B04 | 0.5 µM FCCP | 00:51:33 | 116.59 |
| 2 | 27 | B04 | 0.5 µM FCCP | 00:51:48 | 115.67 |
| 3 | 28 | B04 | 0.5 µM FCCP | 00:57:21 | 137.94 |
| 3 | 29 | B04 | 0.5 µM FCCP | 00:57:35 | 132.86 |
| 3 | 30 | B04 | 0.5 µM FCCP | 00:57:49 | 130.38 |
| 3 | 31 | B04 | 0.5 µM FCCP | 00:58:03 | 128.44 |
| 3 | 32 | B04 | 0.5 µM FCCP | 00:58:17 | 127.00 |
| 3 | 33 | B04 | 0.5 µM FCCP | 00:58:31 | 125.35 |
| 3 | 34 | B04 | 0.5 µM FCCP | 00:58:46 | 124.05 |
| 3 | 35 | B04 | 0.5 µM FCCP | 00:59:00 | 122.73 |
| 3 | 36 | B04 | 0.5 µM FCCP | 00:59:14 | 121.48 |
| 3 | 37 | B04 | 0.5 µM FCCP | 00:59:28 | 120.42 |
| 3 | 38 | B04 | 0.5 µM FCCP | 00:59:42 | 119.24 |
| 3 | 39 | B04 | 0.5 µM FCCP | 00:59:56 | 118.27 |
| 3 | 40 | B04 | 0.5 µM FCCP | 01:00:11 | 117.16 |
| 3 | 41 | B04 | 0.5 µM FCCP | 01:00:25 | 116.16 |
| 4 | 42 | B04 | 0.5 µM FCCP | 01:06:04 | 133.21 |
| 4 | 43 | B04 | 0.5 µM FCCP | 01:06:18 | 120.91 |
| 4 | 44 | B04 | 0.5 µM FCCP | 01:06:33 | 115.61 |
| 4 | 45 | B04 | 0.5 µM FCCP | 01:06:47 | 111.30 |
| 4 | 46 | B04 | 0.5 µM FCCP | 01:07:01 | 107.18 |
| 4 | 47 | B04 | 0.5 µM FCCP | 01:07:15 | 102.75 |
| 4 | 48 | B04 | 0.5 µM FCCP | 01:07:29 | 98.14 |
| 4 | 49 | B04 | 0.5 µM FCCP | 01:07:43 | 93.59 |
| 4 | 50 | B04 | 0.5 µM FCCP | 01:07:58 | 89.30 |
| 4 | 51 | B04 | 0.5 µM FCCP | 01:08:12 | 85.26 |
| 4 | 52 | B04 | 0.5 µM FCCP | 01:08:26 | 81.53 |
| 4 | 53 | B04 | 0.5 µM FCCP | 01:08:40 | 78.19 |
| 4 | 54 | B04 | 0.5 µM FCCP | 01:08:54 | 75.03 |
| 4 | 55 | B04 | 0.5 µM FCCP | 01:09:09 | 72.24 |
| 5 | 56 | B04 | 0.5 µM FCCP | 01:14:42 | 125.03 |
| 5 | 57 | B04 | 0.5 µM FCCP | 01:14:56 | 104.48 |
| 5 | 58 | B04 | 0.5 µM FCCP | 01:15:10 | 96.14 |
| 5 | 59 | B04 | 0.5 µM FCCP | 01:15:25 | 90.17 |
| 5 | 60 | B04 | 0.5 µM FCCP | 01:15:39 | 85.22 |
| 5 | 61 | B04 | 0.5 µM FCCP | 01:15:53 | 80.80 |
| 5 | 62 | B04 | 0.5 µM FCCP | 01:16:07 | 77.07 |
| 5 | 63 | B04 | 0.5 µM FCCP | 01:16:21 | 73.60 |
| 5 | 64 | B04 | 0.5 µM FCCP | 01:16:35 | 70.59 |
| 5 | 65 | B04 | 0.5 µM FCCP | 01:16:50 | 67.79 |
| 5 | 66 | B04 | 0.5 µM FCCP | 01:17:04 | 65.18 |
| 5 | 67 | B04 | 0.5 µM FCCP | 01:17:18 | 62.76 |
| 5 | 68 | B04 | 0.5 µM FCCP | 01:17:32 | 60.55 |
| 5 | 69 | B04 | 0.5 µM FCCP | 01:17:46 | 58.59 |
| 6 | 70 | B04 | 0.5 µM FCCP | 01:23:20 | 121.74 |
| 6 | 71 | B04 | 0.5 µM FCCP | 01:23:34 | 100.99 |
| 6 | 72 | B04 | 0.5 µM FCCP | 01:23:48 | 93.02 |
| 6 | 73 | B04 | 0.5 µM FCCP | 01:24:02 | 87.17 |
| 6 | 74 | B04 | 0.5 µM FCCP | 01:24:16 | 82.47 |
| 6 | 75 | B04 | 0.5 µM FCCP | 01:24:30 | 78.24 |
| 6 | 76 | B04 | 0.5 µM FCCP | 01:24:44 | 74.63 |
| 6 | 77 | B04 | 0.5 µM FCCP | 01:24:59 | 71.28 |
| 6 | 78 | B04 | 0.5 µM FCCP | 01:25:13 | 68.35 |
| 6 | 79 | B04 | 0.5 µM FCCP | 01:25:27 | 65.59 |
| 6 | 80 | B04 | 0.5 µM FCCP | 01:25:41 | 63.03 |
| 6 | 81 | B04 | 0.5 µM FCCP | 01:25:55 | 60.72 |
| 6 | 82 | B04 | 0.5 µM FCCP | 01:26:10 | 58.44 |
| 6 | 83 | B04 | 0.5 µM FCCP | 01:26:24 | 56.44 |
| 7 | 84 | B04 | 0.5 µM FCCP | 01:31:58 | 119.39 |
| 7 | 85 | B04 | 0.5 µM FCCP | 01:32:12 | 99.56 |
| 7 | 86 | B04 | 0.5 µM FCCP | 01:32:26 | 91.83 |
| 7 | 87 | B04 | 0.5 µM FCCP | 01:32:40 | 86.35 |
| 7 | 88 | B04 | 0.5 µM FCCP | 01:32:54 | 81.70 |
| 7 | 89 | B04 | 0.5 µM FCCP | 01:33:08 | 77.73 |
| 7 | 90 | B04 | 0.5 µM FCCP | 01:33:23 | 74.16 |
| 7 | 91 | B04 | 0.5 µM FCCP | 01:33:37 | 70.90 |
| 7 | 92 | B04 | 0.5 µM FCCP | 01:33:51 | 68.08 |
| 7 | 93 | B04 | 0.5 µM FCCP | 01:34:05 | 65.39 |
| 7 | 94 | B04 | 0.5 µM FCCP | 01:34:19 | 62.84 |
| 7 | 95 | B04 | 0.5 µM FCCP | 01:34:33 | 60.57 |
| 7 | 96 | B04 | 0.5 µM FCCP | 01:34:48 | 58.39 |
| 7 | 97 | B04 | 0.5 µM FCCP | 01:35:02 | 56.35 |
| 8 | 98 | B04 | 0.5 µM FCCP | 01:40:36 | 118.39 |
| 8 | 99 | B04 | 0.5 µM FCCP | 01:40:50 | 99.26 |
| 8 | 100 | B04 | 0.5 µM FCCP | 01:41:04 | 91.84 |
| 8 | 101 | B04 | 0.5 µM FCCP | 01:41:18 | 86.51 |
| 8 | 102 | B04 | 0.5 µM FCCP | 01:41:32 | 82.08 |
| 8 | 103 | B04 | 0.5 µM FCCP | 01:41:46 | 78.23 |
| 8 | 104 | B04 | 0.5 µM FCCP | 01:42:00 | 74.80 |
| 8 | 105 | B04 | 0.5 µM FCCP | 01:42:15 | 71.70 |
| 8 | 106 | B04 | 0.5 µM FCCP | 01:42:29 | 68.85 |
| 8 | 107 | B04 | 0.5 µM FCCP | 01:42:43 | 66.26 |
| 8 | 108 | B04 | 0.5 µM FCCP | 01:42:57 | 63.82 |
| 8 | 109 | B04 | 0.5 µM FCCP | 01:43:11 | 61.57 |
| 8 | 110 | B04 | 0.5 µM FCCP | 01:43:26 | 59.37 |
| 8 | 111 | B04 | 0.5 µM FCCP | 01:43:40 | 57.45 |
| 9 | 112 | B04 | 0.5 µM FCCP | 01:49:14 | 118.49 |
| 9 | 113 | B04 | 0.5 µM FCCP | 01:49:28 | 100.19 |
| 9 | 114 | B04 | 0.5 µM FCCP | 01:49:42 | 93.02 |
| 9 | 115 | B04 | 0.5 µM FCCP | 01:49:56 | 87.88 |
| 9 | 116 | B04 | 0.5 µM FCCP | 01:50:10 | 83.56 |
| 9 | 117 | B04 | 0.5 µM FCCP | 01:50:24 | 79.82 |
| 9 | 118 | B04 | 0.5 µM FCCP | 01:50:38 | 76.49 |
| 9 | 119 | B04 | 0.5 µM FCCP | 01:50:53 | 73.41 |
| 9 | 120 | B04 | 0.5 µM FCCP | 01:51:07 | 70.67 |
| 9 | 121 | B04 | 0.5 µM FCCP | 01:51:21 | 68.09 |
| 9 | 122 | B04 | 0.5 µM FCCP | 01:51:35 | 65.74 |
| 9 | 123 | B04 | 0.5 µM FCCP | 01:51:50 | 63.50 |
| 9 | 124 | B04 | 0.5 µM FCCP | 01:52:04 | 61.41 |
| 9 | 125 | B04 | 0.5 µM FCCP | 01:52:18 | 59.41 |
| 10 | 126 | B04 | 0.5 µM FCCP | 01:57:52 | 120.70 |
| 10 | 127 | B04 | 0.5 µM FCCP | 01:58:06 | 105.35 |
| 10 | 128 | B04 | 0.5 µM FCCP | 01:58:20 | 98.12 |
| 10 | 129 | B04 | 0.5 µM FCCP | 01:58:34 | 92.80 |
| 10 | 130 | B04 | 0.5 µM FCCP | 01:58:48 | 88.30 |
| 10 | 131 | B04 | 0.5 µM FCCP | 01:59:03 | 84.25 |
| 10 | 132 | B04 | 0.5 µM FCCP | 01:59:17 | 80.75 |
| 10 | 133 | B04 | 0.5 µM FCCP | 01:59:31 | 77.54 |
| 10 | 134 | B04 | 0.5 µM FCCP | 01:59:45 | 74.74 |
| 10 | 135 | B04 | 0.5 µM FCCP | 01:59:59 | 72.01 |
| 10 | 136 | B04 | 0.5 µM FCCP | 02:00:14 | 69.51 |
| 10 | 137 | B04 | 0.5 µM FCCP | 02:00:28 | 67.22 |
| 10 | 138 | B04 | 0.5 µM FCCP | 02:00:42 | 64.99 |
| 10 | 139 | B04 | 0.5 µM FCCP | 02:00:56 | 62.93 |
| 11 | 140 | B04 | 0.5 µM FCCP | 02:06:30 | 122.98 |
| 11 | 141 | B04 | 0.5 µM FCCP | 02:06:44 | 108.61 |
| 11 | 142 | B04 | 0.5 µM FCCP | 02:06:58 | 101.39 |
| 11 | 143 | B04 | 0.5 µM FCCP | 02:07:12 | 96.04 |
| 11 | 144 | B04 | 0.5 µM FCCP | 02:07:26 | 91.53 |
| 11 | 145 | B04 | 0.5 µM FCCP | 02:07:41 | 87.57 |
| 11 | 146 | B04 | 0.5 µM FCCP | 02:07:55 | 83.93 |
| 11 | 147 | B04 | 0.5 µM FCCP | 02:08:09 | 80.73 |
| 11 | 148 | B04 | 0.5 µM FCCP | 02:08:23 | 77.82 |
| 11 | 149 | B04 | 0.5 µM FCCP | 02:08:37 | 75.11 |
| 11 | 150 | B04 | 0.5 µM FCCP | 02:08:52 | 72.59 |
| 11 | 151 | B04 | 0.5 µM FCCP | 02:09:06 | 70.21 |
| 11 | 152 | B04 | 0.5 µM FCCP | 02:09:20 | 68.05 |
| 11 | 153 | B04 | 0.5 µM FCCP | 02:09:34 | 65.93 |
| 12 | 154 | B04 | 0.5 µM FCCP | 02:15:08 | 123.85 |
| 12 | 155 | B04 | 0.5 µM FCCP | 02:15:22 | 109.57 |
| 12 | 156 | B04 | 0.5 µM FCCP | 02:15:37 | 102.19 |
| 12 | 157 | B04 | 0.5 µM FCCP | 02:15:53 | 96.48 |
| 12 | 158 | B04 | 0.5 µM FCCP | 02:16:08 | 91.95 |
| 12 | 159 | B04 | 0.5 µM FCCP | 02:16:22 | 88.19 |
| 12 | 160 | B04 | 0.5 µM FCCP | 02:16:36 | 84.81 |
| 12 | 161 | B04 | 0.5 µM FCCP | 02:16:50 | 81.74 |
| 12 | 162 | B04 | 0.5 µM FCCP | 02:17:04 | 78.88 |
| 12 | 163 | B04 | 0.5 µM FCCP | 02:17:18 | 76.24 |
| 12 | 164 | B04 | 0.5 µM FCCP | 02:17:33 | 73.77 |
| 12 | 165 | B04 | 0.5 µM FCCP | 02:17:47 | 71.48 |
| 12 | 166 | B04 | 0.5 µM FCCP | 02:18:01 | 69.30 |
| 13 | 167 | B04 | 0.5 µM FCCP | 02:23:39 | 119.85 |
| 13 | 168 | B04 | 0.5 µM FCCP | 02:23:54 | 108.76 |
| 13 | 169 | B04 | 0.5 µM FCCP | 02:24:08 | 102.49 |
| 13 | 170 | B04 | 0.5 µM FCCP | 02:24:22 | 97.61 |
| 13 | 171 | B04 | 0.5 µM FCCP | 02:24:36 | 93.38 |
| 13 | 172 | B04 | 0.5 µM FCCP | 02:24:51 | 89.50 |
| 13 | 173 | B04 | 0.5 µM FCCP | 02:25:07 | 85.84 |
| 13 | 174 | B04 | 0.5 µM FCCP | 02:25:22 | 82.77 |
| 13 | 175 | B04 | 0.5 µM FCCP | 02:25:36 | 80.10 |
| 13 | 176 | B04 | 0.5 µM FCCP | 02:25:50 | 77.53 |
| 13 | 177 | B04 | 0.5 µM FCCP | 02:26:04 | 75.21 |
| 13 | 178 | B04 | 0.5 µM FCCP | 02:26:18 | 72.91 |
| 13 | 179 | B04 | 0.5 µM FCCP | 02:26:33 | 70.76 |
| 14 | 180 | B04 | 0.5 µM FCCP | 02:32:07 | 125.21 |
| 14 | 181 | B04 | 0.5 µM FCCP | 02:32:21 | 111.80 |
| 14 | 182 | B04 | 0.5 µM FCCP | 02:32:35 | 105.31 |
| 14 | 183 | B04 | 0.5 µM FCCP | 02:32:50 | 100.26 |
| 14 | 184 | B04 | 0.5 µM FCCP | 02:33:04 | 96.12 |
| 14 | 185 | B04 | 0.5 µM FCCP | 02:33:18 | 92.32 |
| 14 | 186 | B04 | 0.5 µM FCCP | 02:33:32 | 88.97 |
| 14 | 187 | B04 | 0.5 µM FCCP | 02:33:46 | 85.89 |
| 14 | 188 | B04 | 0.5 µM FCCP | 02:34:01 | 83.23 |
| 14 | 189 | B04 | 0.5 µM FCCP | 02:34:15 | 80.67 |
| 14 | 190 | B04 | 0.5 µM FCCP | 02:34:29 | 78.19 |
| 14 | 191 | B04 | 0.5 µM FCCP | 02:34:43 | 75.92 |
| 14 | 192 | B04 | 0.5 µM FCCP | 02:34:57 | 73.74 |
| 14 | 193 | B04 | 0.5 µM FCCP | 02:35:12 | 71.77 |
| 15 | 194 | B04 | 0.5 µM FCCP | 02:40:46 | 125.78 |
| 15 | 195 | B04 | 0.5 µM FCCP | 02:41:00 | 112.80 |
| 15 | 196 | B04 | 0.5 µM FCCP | 02:41:14 | 106.36 |
| 15 | 197 | B04 | 0.5 µM FCCP | 02:41:28 | 101.52 |
| 15 | 198 | B04 | 0.5 µM FCCP | 02:41:43 | 97.42 |
| 15 | 199 | B04 | 0.5 µM FCCP | 02:41:57 | 93.85 |
| 15 | 200 | B04 | 0.5 µM FCCP | 02:42:11 | 90.60 |
| 15 | 201 | B04 | 0.5 µM FCCP | 02:42:25 | 87.58 |
| 15 | 202 | B04 | 0.5 µM FCCP | 02:42:39 | 84.91 |
| 15 | 203 | B04 | 0.5 µM FCCP | 02:42:53 | 82.33 |
| 15 | 204 | B04 | 0.5 µM FCCP | 02:43:08 | 79.92 |
| 15 | 205 | B04 | 0.5 µM FCCP | 02:43:22 | 77.76 |
| 15 | 206 | B04 | 0.5 µM FCCP | 02:43:36 | 75.52 |
| 15 | 207 | B04 | 0.5 µM FCCP | 02:43:50 | 73.54 |
| 16 | 208 | B04 | 0.5 µM FCCP | 02:49:25 | 126.31 |
| 16 | 209 | B04 | 0.5 µM FCCP | 02:49:40 | 113.75 |
| 16 | 210 | B04 | 0.5 µM FCCP | 02:49:54 | 107.53 |
| 16 | 211 | B04 | 0.5 µM FCCP | 02:50:08 | 102.81 |
| 16 | 212 | B04 | 0.5 µM FCCP | 02:50:22 | 98.82 |
| 16 | 213 | B04 | 0.5 µM FCCP | 02:50:36 | 95.23 |
| 16 | 214 | B04 | 0.5 µM FCCP | 02:50:50 | 92.13 |
| 16 | 215 | B04 | 0.5 µM FCCP | 02:51:05 | 89.14 |
| 16 | 216 | B04 | 0.5 µM FCCP | 02:51:19 | 86.48 |
| 16 | 217 | B04 | 0.5 µM FCCP | 02:51:33 | 83.99 |
| 16 | 218 | B04 | 0.5 µM FCCP | 02:51:47 | 81.64 |
| 16 | 219 | B04 | 0.5 µM FCCP | 02:52:01 | 79.48 |
| 16 | 220 | B04 | 0.5 µM FCCP | 02:52:16 | 77.31 |
| 16 | 221 | B04 | 0.5 µM FCCP | 02:52:30 | 75.30 |
| 1 | 0 | B05 | 0.5 µM FCCP | 00:40:06 | 138.92 |
| 1 | 1 | B05 | 0.5 µM FCCP | 00:40:20 | 131.15 |
| 1 | 2 | B05 | 0.5 µM FCCP | 00:40:34 | 128.20 |
| 1 | 3 | B05 | 0.5 µM FCCP | 00:40:48 | 125.96 |
| 1 | 4 | B05 | 0.5 µM FCCP | 00:41:02 | 124.09 |
| 1 | 5 | B05 | 0.5 µM FCCP | 00:41:17 | 122.47 |
| 1 | 6 | B05 | 0.5 µM FCCP | 00:41:31 | 120.96 |
| 1 | 7 | B05 | 0.5 µM FCCP | 00:41:45 | 119.64 |
| 1 | 8 | B05 | 0.5 µM FCCP | 00:41:59 | 118.23 |
| 1 | 9 | B05 | 0.5 µM FCCP | 00:42:13 | 117.06 |
| 1 | 10 | B05 | 0.5 µM FCCP | 00:42:28 | 115.79 |
| 1 | 11 | B05 | 0.5 µM FCCP | 00:42:42 | 114.65 |
| 1 | 12 | B05 | 0.5 µM FCCP | 00:42:56 | 113.57 |
| 1 | 13 | B05 | 0.5 µM FCCP | 00:43:10 | 112.52 |
| 2 | 14 | B05 | 0.5 µM FCCP | 00:48:43 | 139.54 |
| 2 | 15 | B05 | 0.5 µM FCCP | 00:48:57 | 133.17 |
| 2 | 16 | B05 | 0.5 µM FCCP | 00:49:12 | 130.55 |
| 2 | 17 | B05 | 0.5 µM FCCP | 00:49:26 | 128.35 |
| 2 | 18 | B05 | 0.5 µM FCCP | 00:49:40 | 126.61 |
| 2 | 19 | B05 | 0.5 µM FCCP | 00:49:54 | 124.91 |
| 2 | 20 | B05 | 0.5 µM FCCP | 00:50:08 | 123.45 |
| 2 | 21 | B05 | 0.5 µM FCCP | 00:50:22 | 121.95 |
| 2 | 22 | B05 | 0.5 µM FCCP | 00:50:37 | 120.74 |
| 2 | 23 | B05 | 0.5 µM FCCP | 00:50:51 | 119.39 |
| 2 | 24 | B05 | 0.5 µM FCCP | 00:51:05 | 118.20 |
| 2 | 25 | B05 | 0.5 µM FCCP | 00:51:19 | 117.11 |
| 2 | 26 | B05 | 0.5 µM FCCP | 00:51:33 | 115.91 |
| 2 | 27 | B05 | 0.5 µM FCCP | 00:51:48 | 114.78 |
| 3 | 28 | B05 | 0.5 µM FCCP | 00:57:21 | 139.11 |
| 3 | 29 | B05 | 0.5 µM FCCP | 00:57:35 | 132.97 |
| 3 | 30 | B05 | 0.5 µM FCCP | 00:57:49 | 130.40 |
| 3 | 31 | B05 | 0.5 µM FCCP | 00:58:03 | 128.40 |
| 3 | 32 | B05 | 0.5 µM FCCP | 00:58:17 | 126.65 |
| 3 | 33 | B05 | 0.5 µM FCCP | 00:58:31 | 125.02 |
| 3 | 34 | B05 | 0.5 µM FCCP | 00:58:46 | 123.58 |
| 3 | 35 | B05 | 0.5 µM FCCP | 00:59:00 | 122.17 |
| 3 | 36 | B05 | 0.5 µM FCCP | 00:59:14 | 120.73 |
| 3 | 37 | B05 | 0.5 µM FCCP | 00:59:28 | 119.65 |
| 3 | 38 | B05 | 0.5 µM FCCP | 00:59:42 | 118.43 |
| 3 | 39 | B05 | 0.5 µM FCCP | 00:59:56 | 117.28 |
| 3 | 40 | B05 | 0.5 µM FCCP | 01:00:11 | 116.19 |
| 3 | 41 | B05 | 0.5 µM FCCP | 01:00:25 | 115.10 |
| 4 | 42 | B05 | 0.5 µM FCCP | 01:06:04 | 135.27 |
| 4 | 43 | B05 | 0.5 µM FCCP | 01:06:18 | 123.05 |
| 4 | 44 | B05 | 0.5 µM FCCP | 01:06:33 | 118.23 |
| 4 | 45 | B05 | 0.5 µM FCCP | 01:06:47 | 114.26 |
| 4 | 46 | B05 | 0.5 µM FCCP | 01:07:01 | 110.56 |
| 4 | 47 | B05 | 0.5 µM FCCP | 01:07:15 | 106.74 |
| 4 | 48 | B05 | 0.5 µM FCCP | 01:07:29 | 102.59 |
| 4 | 49 | B05 | 0.5 µM FCCP | 01:07:43 | 98.27 |
| 4 | 50 | B05 | 0.5 µM FCCP | 01:07:58 | 93.82 |
| 4 | 51 | B05 | 0.5 µM FCCP | 01:08:12 | 89.41 |
| 4 | 52 | B05 | 0.5 µM FCCP | 01:08:26 | 85.10 |
| 4 | 53 | B05 | 0.5 µM FCCP | 01:08:40 | 81.21 |
| 4 | 54 | B05 | 0.5 µM FCCP | 01:08:54 | 77.48 |
| 4 | 55 | B05 | 0.5 µM FCCP | 01:09:09 | 74.11 |
| 5 | 56 | B05 | 0.5 µM FCCP | 01:14:42 | 126.57 |
| 5 | 57 | B05 | 0.5 µM FCCP | 01:14:56 | 105.42 |
| 5 | 58 | B05 | 0.5 µM FCCP | 01:15:10 | 96.94 |
| 5 | 59 | B05 | 0.5 µM FCCP | 01:15:25 | 90.59 |
| 5 | 60 | B05 | 0.5 µM FCCP | 01:15:39 | 85.19 |
| 5 | 61 | B05 | 0.5 µM FCCP | 01:15:53 | 80.35 |
| 5 | 62 | B05 | 0.5 µM FCCP | 01:16:07 | 75.99 |
| 5 | 63 | B05 | 0.5 µM FCCP | 01:16:21 | 72.03 |
| 5 | 64 | B05 | 0.5 µM FCCP | 01:16:35 | 68.55 |
| 5 | 65 | B05 | 0.5 µM FCCP | 01:16:50 | 65.29 |
| 5 | 66 | B05 | 0.5 µM FCCP | 01:17:04 | 62.24 |
| 5 | 67 | B05 | 0.5 µM FCCP | 01:17:18 | 59.43 |
| 5 | 68 | B05 | 0.5 µM FCCP | 01:17:32 | 56.82 |
| 5 | 69 | B05 | 0.5 µM FCCP | 01:17:46 | 54.44 |
| 6 | 70 | B05 | 0.5 µM FCCP | 01:23:20 | 122.01 |
| 6 | 71 | B05 | 0.5 µM FCCP | 01:23:34 | 99.43 |
| 6 | 72 | B05 | 0.5 µM FCCP | 01:23:48 | 90.79 |
| 6 | 73 | B05 | 0.5 µM FCCP | 01:24:02 | 84.32 |
| 6 | 74 | B05 | 0.5 µM FCCP | 01:24:16 | 79.00 |
| 6 | 75 | B05 | 0.5 µM FCCP | 01:24:30 | 74.30 |
| 6 | 76 | B05 | 0.5 µM FCCP | 01:24:44 | 70.17 |
| 6 | 77 | B05 | 0.5 µM FCCP | 01:24:59 | 66.36 |
| 6 | 78 | B05 | 0.5 µM FCCP | 01:25:13 | 62.91 |
| 6 | 79 | B05 | 0.5 µM FCCP | 01:25:27 | 59.74 |
| 6 | 80 | B05 | 0.5 µM FCCP | 01:25:41 | 56.81 |
| 6 | 81 | B05 | 0.5 µM FCCP | 01:25:55 | 54.11 |
| 6 | 82 | B05 | 0.5 µM FCCP | 01:26:10 | 51.55 |
| 6 | 83 | B05 | 0.5 µM FCCP | 01:26:24 | 49.13 |
| 7 | 84 | B05 | 0.5 µM FCCP | 01:31:58 | 119.17 |
| 7 | 85 | B05 | 0.5 µM FCCP | 01:32:12 | 97.08 |
| 7 | 86 | B05 | 0.5 µM FCCP | 01:32:26 | 88.61 |
| 7 | 87 | B05 | 0.5 µM FCCP | 01:32:40 | 82.48 |
| 7 | 88 | B05 | 0.5 µM FCCP | 01:32:54 | 77.25 |
| 7 | 89 | B05 | 0.5 µM FCCP | 01:33:08 | 72.73 |
| 7 | 90 | B05 | 0.5 µM FCCP | 01:33:23 | 68.73 |
| 7 | 91 | B05 | 0.5 µM FCCP | 01:33:37 | 64.94 |
| 7 | 92 | B05 | 0.5 µM FCCP | 01:33:51 | 61.63 |
| 7 | 93 | B05 | 0.5 µM FCCP | 01:34:05 | 58.50 |
| 7 | 94 | B05 | 0.5 µM FCCP | 01:34:19 | 55.59 |
| 7 | 95 | B05 | 0.5 µM FCCP | 01:34:33 | 52.92 |
| 7 | 96 | B05 | 0.5 µM FCCP | 01:34:48 | 50.40 |
| 7 | 97 | B05 | 0.5 µM FCCP | 01:35:02 | 48.04 |
| 8 | 98 | B05 | 0.5 µM FCCP | 01:40:36 | 118.00 |
| 8 | 99 | B05 | 0.5 µM FCCP | 01:40:50 | 96.37 |
| 8 | 100 | B05 | 0.5 µM FCCP | 01:41:04 | 88.15 |
| 8 | 101 | B05 | 0.5 µM FCCP | 01:41:18 | 82.14 |
| 8 | 102 | B05 | 0.5 µM FCCP | 01:41:32 | 77.16 |
| 8 | 103 | B05 | 0.5 µM FCCP | 01:41:46 | 72.74 |
| 8 | 104 | B05 | 0.5 µM FCCP | 01:42:00 | 68.79 |
| 8 | 105 | B05 | 0.5 µM FCCP | 01:42:15 | 65.15 |
| 8 | 106 | B05 | 0.5 µM FCCP | 01:42:29 | 61.77 |
| 8 | 107 | B05 | 0.5 µM FCCP | 01:42:43 | 58.73 |
| 8 | 108 | B05 | 0.5 µM FCCP | 01:42:57 | 55.86 |
| 8 | 109 | B05 | 0.5 µM FCCP | 01:43:11 | 53.22 |
| 8 | 110 | B05 | 0.5 µM FCCP | 01:43:26 | 50.64 |
| 8 | 111 | B05 | 0.5 µM FCCP | 01:43:40 | 48.33 |
| 9 | 112 | B05 | 0.5 µM FCCP | 01:49:14 | 117.68 |
| 9 | 113 | B05 | 0.5 µM FCCP | 01:49:28 | 96.76 |
| 9 | 114 | B05 | 0.5 µM FCCP | 01:49:42 | 88.69 |
| 9 | 115 | B05 | 0.5 µM FCCP | 01:49:56 | 82.83 |
| 9 | 116 | B05 | 0.5 µM FCCP | 01:50:10 | 77.88 |
| 9 | 117 | B05 | 0.5 µM FCCP | 01:50:24 | 73.50 |
| 9 | 118 | B05 | 0.5 µM FCCP | 01:50:38 | 69.62 |
| 9 | 119 | B05 | 0.5 µM FCCP | 01:50:53 | 66.02 |
| 9 | 120 | B05 | 0.5 µM FCCP | 01:51:07 | 62.70 |
| 9 | 121 | B05 | 0.5 µM FCCP | 01:51:21 | 59.69 |
| 9 | 122 | B05 | 0.5 µM FCCP | 01:51:35 | 56.87 |
| 9 | 123 | B05 | 0.5 µM FCCP | 01:51:50 | 54.16 |
| 9 | 124 | B05 | 0.5 µM FCCP | 01:52:04 | 51.67 |
| 9 | 125 | B05 | 0.5 µM FCCP | 01:52:18 | 49.25 |
| 10 | 126 | B05 | 0.5 µM FCCP | 01:57:52 | 118.67 |
| 10 | 127 | B05 | 0.5 µM FCCP | 01:58:06 | 100.56 |
| 10 | 128 | B05 | 0.5 µM FCCP | 01:58:20 | 92.56 |
| 10 | 129 | B05 | 0.5 µM FCCP | 01:58:34 | 86.73 |
| 10 | 130 | B05 | 0.5 µM FCCP | 01:58:48 | 81.64 |
| 10 | 131 | B05 | 0.5 µM FCCP | 01:59:03 | 77.13 |
| 10 | 132 | B05 | 0.5 µM FCCP | 01:59:17 | 73.07 |
| 10 | 133 | B05 | 0.5 µM FCCP | 01:59:31 | 69.37 |
| 10 | 134 | B05 | 0.5 µM FCCP | 01:59:45 | 66.10 |
| 10 | 135 | B05 | 0.5 µM FCCP | 01:59:59 | 62.93 |
| 10 | 136 | B05 | 0.5 µM FCCP | 02:00:14 | 60.00 |
| 10 | 137 | B05 | 0.5 µM FCCP | 02:00:28 | 57.25 |
| 10 | 138 | B05 | 0.5 µM FCCP | 02:00:42 | 54.65 |
| 10 | 139 | B05 | 0.5 µM FCCP | 02:00:56 | 52.24 |
| 11 | 140 | B05 | 0.5 µM FCCP | 02:06:30 | 120.03 |
| 11 | 141 | B05 | 0.5 µM FCCP | 02:06:44 | 103.21 |
| 11 | 142 | B05 | 0.5 µM FCCP | 02:06:58 | 95.45 |
| 11 | 143 | B05 | 0.5 µM FCCP | 02:07:12 | 89.56 |
| 11 | 144 | B05 | 0.5 µM FCCP | 02:07:26 | 84.53 |
| 11 | 145 | B05 | 0.5 µM FCCP | 02:07:41 | 80.04 |
| 11 | 146 | B05 | 0.5 µM FCCP | 02:07:55 | 76.01 |
| 11 | 147 | B05 | 0.5 µM FCCP | 02:08:09 | 72.34 |
| 11 | 148 | B05 | 0.5 µM FCCP | 02:08:23 | 68.98 |
| 11 | 149 | B05 | 0.5 µM FCCP | 02:08:37 | 65.83 |
| 11 | 150 | B05 | 0.5 µM FCCP | 02:08:52 | 62.92 |
| 11 | 151 | B05 | 0.5 µM FCCP | 02:09:06 | 60.14 |
| 11 | 152 | B05 | 0.5 µM FCCP | 02:09:20 | 57.56 |
| 11 | 153 | B05 | 0.5 µM FCCP | 02:09:34 | 55.11 |
| 12 | 154 | B05 | 0.5 µM FCCP | 02:15:08 | 120.14 |
| 12 | 155 | B05 | 0.5 µM FCCP | 02:15:22 | 103.72 |
| 12 | 156 | B05 | 0.5 µM FCCP | 02:15:37 | 95.94 |
| 12 | 157 | B05 | 0.5 µM FCCP | 02:15:53 | 89.66 |
| 12 | 158 | B05 | 0.5 µM FCCP | 02:16:08 | 84.70 |
| 12 | 159 | B05 | 0.5 µM FCCP | 02:16:22 | 80.58 |
| 12 | 160 | B05 | 0.5 µM FCCP | 02:16:36 | 76.74 |
| 12 | 161 | B05 | 0.5 µM FCCP | 02:16:50 | 73.21 |
| 12 | 162 | B05 | 0.5 µM FCCP | 02:17:04 | 69.94 |
| 12 | 163 | B05 | 0.5 µM FCCP | 02:17:18 | 66.92 |
| 12 | 164 | B05 | 0.5 µM FCCP | 02:17:33 | 64.05 |
| 12 | 165 | B05 | 0.5 µM FCCP | 02:17:47 | 61.37 |
| 12 | 166 | B05 | 0.5 µM FCCP | 02:18:01 | 58.86 |
| 13 | 167 | B05 | 0.5 µM FCCP | 02:23:39 | 115.09 |
| 13 | 168 | B05 | 0.5 µM FCCP | 02:23:54 | 102.87 |
| 13 | 169 | B05 | 0.5 µM FCCP | 02:24:08 | 96.17 |
| 13 | 170 | B05 | 0.5 µM FCCP | 02:24:22 | 90.81 |
| 13 | 171 | B05 | 0.5 µM FCCP | 02:24:36 | 86.23 |
| 13 | 172 | B05 | 0.5 µM FCCP | 02:24:51 | 81.81 |
| 13 | 173 | B05 | 0.5 µM FCCP | 02:25:07 | 77.72 |
| 13 | 174 | B05 | 0.5 µM FCCP | 02:25:22 | 74.20 |
| 13 | 175 | B05 | 0.5 µM FCCP | 02:25:36 | 71.19 |
| 13 | 176 | B05 | 0.5 µM FCCP | 02:25:50 | 68.23 |
| 13 | 177 | B05 | 0.5 µM FCCP | 02:26:04 | 65.46 |
| 13 | 178 | B05 | 0.5 µM FCCP | 02:26:18 | 62.87 |
| 13 | 179 | B05 | 0.5 µM FCCP | 02:26:33 | 60.39 |
| 14 | 180 | B05 | 0.5 µM FCCP | 02:32:07 | 120.98 |
| 14 | 181 | B05 | 0.5 µM FCCP | 02:32:21 | 105.72 |
| 14 | 182 | B05 | 0.5 µM FCCP | 02:32:35 | 98.75 |
| 14 | 183 | B05 | 0.5 µM FCCP | 02:32:50 | 93.25 |
| 14 | 184 | B05 | 0.5 µM FCCP | 02:33:04 | 88.73 |
| 14 | 185 | B05 | 0.5 µM FCCP | 02:33:18 | 84.58 |
| 14 | 186 | B05 | 0.5 µM FCCP | 02:33:32 | 80.85 |
| 14 | 187 | B05 | 0.5 µM FCCP | 02:33:46 | 77.38 |
| 14 | 188 | B05 | 0.5 µM FCCP | 02:34:01 | 74.25 |
| 14 | 189 | B05 | 0.5 µM FCCP | 02:34:15 | 71.31 |
| 14 | 190 | B05 | 0.5 µM FCCP | 02:34:29 | 68.50 |
| 14 | 191 | B05 | 0.5 µM FCCP | 02:34:43 | 65.88 |
| 14 | 192 | B05 | 0.5 µM FCCP | 02:34:57 | 63.34 |
| 14 | 193 | B05 | 0.5 µM FCCP | 02:35:12 | 61.00 |
| 15 | 194 | B05 | 0.5 µM FCCP | 02:40:46 | 121.14 |
| 15 | 195 | B05 | 0.5 µM FCCP | 02:41:00 | 106.33 |
| 15 | 196 | B05 | 0.5 µM FCCP | 02:41:14 | 99.57 |
| 15 | 197 | B05 | 0.5 µM FCCP | 02:41:28 | 94.31 |
| 15 | 198 | B05 | 0.5 µM FCCP | 02:41:43 | 89.83 |
| 15 | 199 | B05 | 0.5 µM FCCP | 02:41:57 | 85.87 |
| 15 | 200 | B05 | 0.5 µM FCCP | 02:42:11 | 82.23 |
| 15 | 201 | B05 | 0.5 µM FCCP | 02:42:25 | 78.90 |
| 15 | 202 | B05 | 0.5 µM FCCP | 02:42:39 | 75.78 |
| 15 | 203 | B05 | 0.5 µM FCCP | 02:42:53 | 72.94 |
| 15 | 204 | B05 | 0.5 µM FCCP | 02:43:08 | 70.12 |
| 15 | 205 | B05 | 0.5 µM FCCP | 02:43:22 | 67.64 |
| 15 | 206 | B05 | 0.5 µM FCCP | 02:43:36 | 65.08 |
| 15 | 207 | B05 | 0.5 µM FCCP | 02:43:50 | 62.78 |
| 16 | 208 | B05 | 0.5 µM FCCP | 02:49:25 | 121.23 |
| 16 | 209 | B05 | 0.5 µM FCCP | 02:49:40 | 107.03 |
| 16 | 210 | B05 | 0.5 µM FCCP | 02:49:54 | 100.45 |
| 16 | 211 | B05 | 0.5 µM FCCP | 02:50:08 | 95.31 |
| 16 | 212 | B05 | 0.5 µM FCCP | 02:50:22 | 91.01 |
| 16 | 213 | B05 | 0.5 µM FCCP | 02:50:36 | 87.09 |
| 16 | 214 | B05 | 0.5 µM FCCP | 02:50:50 | 83.63 |
| 16 | 215 | B05 | 0.5 µM FCCP | 02:51:05 | 80.27 |
| 16 | 216 | B05 | 0.5 µM FCCP | 02:51:19 | 77.25 |
| 16 | 217 | B05 | 0.5 µM FCCP | 02:51:33 | 74.43 |
| 16 | 218 | B05 | 0.5 µM FCCP | 02:51:47 | 71.81 |
| 16 | 219 | B05 | 0.5 µM FCCP | 02:52:01 | 69.29 |
| 16 | 220 | B05 | 0.5 µM FCCP | 02:52:16 | 66.81 |
| 16 | 221 | B05 | 0.5 µM FCCP | 02:52:30 | 64.47 |
| 1 | 0 | B06 | 0.5 µM FCCP | 00:40:06 | 135.77 |
| 1 | 1 | B06 | 0.5 µM FCCP | 00:40:20 | 129.50 |
| 1 | 2 | B06 | 0.5 µM FCCP | 00:40:34 | 126.83 |
| 1 | 3 | B06 | 0.5 µM FCCP | 00:40:48 | 124.71 |
| 1 | 4 | B06 | 0.5 µM FCCP | 00:41:02 | 123.01 |
| 1 | 5 | B06 | 0.5 µM FCCP | 00:41:17 | 121.36 |
| 1 | 6 | B06 | 0.5 µM FCCP | 00:41:31 | 119.95 |
| 1 | 7 | B06 | 0.5 µM FCCP | 00:41:45 | 118.62 |
| 1 | 8 | B06 | 0.5 µM FCCP | 00:41:59 | 117.38 |
| 1 | 9 | B06 | 0.5 µM FCCP | 00:42:13 | 116.23 |
| 1 | 10 | B06 | 0.5 µM FCCP | 00:42:28 | 114.99 |
| 1 | 11 | B06 | 0.5 µM FCCP | 00:42:42 | 113.96 |
| 1 | 12 | B06 | 0.5 µM FCCP | 00:42:56 | 112.87 |
| 1 | 13 | B06 | 0.5 µM FCCP | 00:43:10 | 111.86 |
| 2 | 14 | B06 | 0.5 µM FCCP | 00:48:43 | 137.82 |
| 2 | 15 | B06 | 0.5 µM FCCP | 00:48:57 | 131.88 |
| 2 | 16 | B06 | 0.5 µM FCCP | 00:49:12 | 129.40 |
| 2 | 17 | B06 | 0.5 µM FCCP | 00:49:26 | 127.48 |
| 2 | 18 | B06 | 0.5 µM FCCP | 00:49:40 | 125.78 |
| 2 | 19 | B06 | 0.5 µM FCCP | 00:49:54 | 124.25 |
| 2 | 20 | B06 | 0.5 µM FCCP | 00:50:08 | 122.79 |
| 2 | 21 | B06 | 0.5 µM FCCP | 00:50:22 | 121.41 |
| 2 | 22 | B06 | 0.5 µM FCCP | 00:50:37 | 120.23 |
| 2 | 23 | B06 | 0.5 µM FCCP | 00:50:51 | 119.01 |
| 2 | 24 | B06 | 0.5 µM FCCP | 00:51:05 | 117.83 |
| 2 | 25 | B06 | 0.5 µM FCCP | 00:51:19 | 116.77 |
| 2 | 26 | B06 | 0.5 µM FCCP | 00:51:33 | 115.61 |
| 2 | 27 | B06 | 0.5 µM FCCP | 00:51:48 | 114.62 |
| 3 | 28 | B06 | 0.5 µM FCCP | 00:57:21 | 138.16 |
| 3 | 29 | B06 | 0.5 µM FCCP | 00:57:35 | 132.51 |
| 3 | 30 | B06 | 0.5 µM FCCP | 00:57:49 | 130.03 |
| 3 | 31 | B06 | 0.5 µM FCCP | 00:58:03 | 128.09 |
| 3 | 32 | B06 | 0.5 µM FCCP | 00:58:17 | 126.48 |
| 3 | 33 | B06 | 0.5 µM FCCP | 00:58:31 | 124.92 |
| 3 | 34 | B06 | 0.5 µM FCCP | 00:58:46 | 123.48 |
| 3 | 35 | B06 | 0.5 µM FCCP | 00:59:00 | 122.12 |
| 3 | 36 | B06 | 0.5 µM FCCP | 00:59:14 | 120.90 |
| 3 | 37 | B06 | 0.5 µM FCCP | 00:59:28 | 119.75 |
| 3 | 38 | B06 | 0.5 µM FCCP | 00:59:42 | 118.58 |
| 3 | 39 | B06 | 0.5 µM FCCP | 00:59:56 | 117.52 |
| 3 | 40 | B06 | 0.5 µM FCCP | 01:00:11 | 116.40 |
| 3 | 41 | B06 | 0.5 µM FCCP | 01:00:25 | 115.46 |
| 4 | 42 | B06 | 0.5 µM FCCP | 01:06:04 | 133.84 |
| 4 | 43 | B06 | 0.5 µM FCCP | 01:06:18 | 121.88 |
| 4 | 44 | B06 | 0.5 µM FCCP | 01:06:33 | 117.05 |
| 4 | 45 | B06 | 0.5 µM FCCP | 01:06:47 | 113.10 |
| 4 | 46 | B06 | 0.5 µM FCCP | 01:07:01 | 109.20 |
| 4 | 47 | B06 | 0.5 µM FCCP | 01:07:15 | 104.97 |
| 4 | 48 | B06 | 0.5 µM FCCP | 01:07:29 | 100.57 |
| 4 | 49 | B06 | 0.5 µM FCCP | 01:07:43 | 95.87 |
| 4 | 50 | B06 | 0.5 µM FCCP | 01:07:58 | 91.49 |
| 4 | 51 | B06 | 0.5 µM FCCP | 01:08:12 | 87.33 |
| 4 | 52 | B06 | 0.5 µM FCCP | 01:08:26 | 83.42 |
| 4 | 53 | B06 | 0.5 µM FCCP | 01:08:40 | 79.98 |
| 4 | 54 | B06 | 0.5 µM FCCP | 01:08:54 | 76.57 |
| 4 | 55 | B06 | 0.5 µM FCCP | 01:09:09 | 73.56 |
| 5 | 56 | B06 | 0.5 µM FCCP | 01:14:42 | 125.14 |
| 5 | 57 | B06 | 0.5 µM FCCP | 01:14:56 | 103.88 |
| 5 | 58 | B06 | 0.5 µM FCCP | 01:15:10 | 95.29 |
| 5 | 59 | B06 | 0.5 µM FCCP | 01:15:25 | 89.13 |
| 5 | 60 | B06 | 0.5 µM FCCP | 01:15:39 | 83.96 |
| 5 | 61 | B06 | 0.5 µM FCCP | 01:15:53 | 79.38 |
| 5 | 62 | B06 | 0.5 µM FCCP | 01:16:07 | 75.45 |
| 5 | 63 | B06 | 0.5 µM FCCP | 01:16:21 | 71.82 |
| 5 | 64 | B06 | 0.5 µM FCCP | 01:16:35 | 68.62 |
| 5 | 65 | B06 | 0.5 µM FCCP | 01:16:50 | 65.65 |
| 5 | 66 | B06 | 0.5 µM FCCP | 01:17:04 | 62.87 |
| 5 | 67 | B06 | 0.5 µM FCCP | 01:17:18 | 60.35 |
| 5 | 68 | B06 | 0.5 µM FCCP | 01:17:32 | 58.01 |
| 5 | 69 | B06 | 0.5 µM FCCP | 01:17:46 | 55.91 |
| 6 | 70 | B06 | 0.5 µM FCCP | 01:23:20 | 121.22 |
| 6 | 71 | B06 | 0.5 µM FCCP | 01:23:34 | 99.06 |
| 6 | 72 | B06 | 0.5 µM FCCP | 01:23:48 | 90.51 |
| 6 | 73 | B06 | 0.5 µM FCCP | 01:24:02 | 84.37 |
| 6 | 74 | B06 | 0.5 µM FCCP | 01:24:16 | 79.37 |
| 6 | 75 | B06 | 0.5 µM FCCP | 01:24:30 | 74.90 |
| 6 | 76 | B06 | 0.5 µM FCCP | 01:24:44 | 71.13 |
| 6 | 77 | B06 | 0.5 µM FCCP | 01:24:59 | 67.51 |
| 6 | 78 | B06 | 0.5 µM FCCP | 01:25:13 | 64.38 |
| 6 | 79 | B06 | 0.5 µM FCCP | 01:25:27 | 61.47 |
| 6 | 80 | B06 | 0.5 µM FCCP | 01:25:41 | 58.77 |
| 6 | 81 | B06 | 0.5 µM FCCP | 01:25:55 | 56.31 |
| 6 | 82 | B06 | 0.5 µM FCCP | 01:26:10 | 53.96 |
| 6 | 83 | B06 | 0.5 µM FCCP | 01:26:24 | 51.81 |
| 7 | 84 | B06 | 0.5 µM FCCP | 01:31:58 | 118.79 |
| 7 | 85 | B06 | 0.5 µM FCCP | 01:32:12 | 97.15 |
| 7 | 86 | B06 | 0.5 µM FCCP | 01:32:26 | 88.88 |
| 7 | 87 | B06 | 0.5 µM FCCP | 01:32:40 | 83.02 |
| 7 | 88 | B06 | 0.5 µM FCCP | 01:32:54 | 78.10 |
| 7 | 89 | B06 | 0.5 µM FCCP | 01:33:08 | 73.83 |
| 7 | 90 | B06 | 0.5 µM FCCP | 01:33:23 | 70.04 |
| 7 | 91 | B06 | 0.5 µM FCCP | 01:33:37 | 66.58 |
| 7 | 92 | B06 | 0.5 µM FCCP | 01:33:51 | 63.49 |
| 7 | 93 | B06 | 0.5 µM FCCP | 01:34:05 | 60.64 |
| 7 | 94 | B06 | 0.5 µM FCCP | 01:34:19 | 57.95 |
| 7 | 95 | B06 | 0.5 µM FCCP | 01:34:33 | 55.53 |
| 7 | 96 | B06 | 0.5 µM FCCP | 01:34:48 | 53.23 |
| 7 | 97 | B06 | 0.5 µM FCCP | 01:35:02 | 51.09 |
| 8 | 98 | B06 | 0.5 µM FCCP | 01:40:36 | 117.74 |
| 8 | 99 | B06 | 0.5 µM FCCP | 01:40:50 | 96.71 |
| 8 | 100 | B06 | 0.5 µM FCCP | 01:41:04 | 88.74 |
| 8 | 101 | B06 | 0.5 µM FCCP | 01:41:18 | 82.98 |
| 8 | 102 | B06 | 0.5 µM FCCP | 01:41:32 | 78.25 |
| 8 | 103 | B06 | 0.5 µM FCCP | 01:41:46 | 74.08 |
| 8 | 104 | B06 | 0.5 µM FCCP | 01:42:00 | 70.45 |
| 8 | 105 | B06 | 0.5 µM FCCP | 01:42:15 | 67.06 |
| 8 | 106 | B06 | 0.5 µM FCCP | 01:42:29 | 64.02 |
| 8 | 107 | B06 | 0.5 µM FCCP | 01:42:43 | 61.23 |
| 8 | 108 | B06 | 0.5 µM FCCP | 01:42:57 | 58.63 |
| 8 | 109 | B06 | 0.5 µM FCCP | 01:43:11 | 56.19 |
| 8 | 110 | B06 | 0.5 µM FCCP | 01:43:26 | 53.86 |
| 8 | 111 | B06 | 0.5 µM FCCP | 01:43:40 | 51.76 |
| 9 | 112 | B06 | 0.5 µM FCCP | 01:49:14 | 117.60 |
| 9 | 113 | B06 | 0.5 µM FCCP | 01:49:28 | 97.21 |
| 9 | 114 | B06 | 0.5 µM FCCP | 01:49:42 | 89.45 |
| 9 | 115 | B06 | 0.5 µM FCCP | 01:49:56 | 83.93 |
| 9 | 116 | B06 | 0.5 µM FCCP | 01:50:10 | 79.30 |
| 9 | 117 | B06 | 0.5 µM FCCP | 01:50:24 | 75.29 |
| 9 | 118 | B06 | 0.5 µM FCCP | 01:50:38 | 71.73 |
| 9 | 119 | B06 | 0.5 µM FCCP | 01:50:53 | 68.42 |
| 9 | 120 | B06 | 0.5 µM FCCP | 01:51:07 | 65.49 |
| 9 | 121 | B06 | 0.5 µM FCCP | 01:51:21 | 62.77 |
| 9 | 122 | B06 | 0.5 µM FCCP | 01:51:35 | 60.25 |
| 9 | 123 | B06 | 0.5 µM FCCP | 01:51:50 | 57.85 |
| 9 | 124 | B06 | 0.5 µM FCCP | 01:52:04 | 55.63 |
| 9 | 125 | B06 | 0.5 µM FCCP | 01:52:18 | 53.51 |
| 10 | 126 | B06 | 0.5 µM FCCP | 01:57:52 | 119.62 |
| 10 | 127 | B06 | 0.5 µM FCCP | 01:58:06 | 101.12 |
| 10 | 128 | B06 | 0.5 µM FCCP | 01:58:20 | 93.33 |
| 10 | 129 | B06 | 0.5 µM FCCP | 01:58:34 | 87.63 |
| 10 | 130 | B06 | 0.5 µM FCCP | 01:58:48 | 82.88 |
| 10 | 131 | B06 | 0.5 µM FCCP | 01:59:03 | 78.68 |
| 10 | 132 | B06 | 0.5 µM FCCP | 01:59:17 | 74.97 |
| 10 | 133 | B06 | 0.5 µM FCCP | 01:59:31 | 71.62 |
| 10 | 134 | B06 | 0.5 µM FCCP | 01:59:45 | 68.64 |
| 10 | 135 | B06 | 0.5 µM FCCP | 01:59:59 | 65.78 |
| 10 | 136 | B06 | 0.5 µM FCCP | 02:00:14 | 63.15 |
| 10 | 137 | B06 | 0.5 µM FCCP | 02:00:28 | 60.78 |
| 10 | 138 | B06 | 0.5 µM FCCP | 02:00:42 | 58.45 |
| 10 | 139 | B06 | 0.5 µM FCCP | 02:00:56 | 56.32 |
| 11 | 140 | B06 | 0.5 µM FCCP | 02:06:30 | 122.21 |
| 11 | 141 | B06 | 0.5 µM FCCP | 02:06:44 | 106.45 |
| 11 | 142 | B06 | 0.5 µM FCCP | 02:06:58 | 98.83 |
| 11 | 143 | B06 | 0.5 µM FCCP | 02:07:12 | 93.01 |
| 11 | 144 | B06 | 0.5 µM FCCP | 02:07:26 | 88.29 |
| 11 | 145 | B06 | 0.5 µM FCCP | 02:07:41 | 83.92 |
| 11 | 146 | B06 | 0.5 µM FCCP | 02:07:55 | 80.13 |
| 11 | 147 | B06 | 0.5 µM FCCP | 02:08:09 | 76.68 |
| 11 | 148 | B06 | 0.5 µM FCCP | 02:08:23 | 73.56 |
| 11 | 149 | B06 | 0.5 µM FCCP | 02:08:37 | 70.62 |
| 11 | 150 | B06 | 0.5 µM FCCP | 02:08:52 | 67.88 |
| 11 | 151 | B06 | 0.5 µM FCCP | 02:09:06 | 65.37 |
| 11 | 152 | B06 | 0.5 µM FCCP | 02:09:20 | 63.01 |
| 11 | 153 | B06 | 0.5 µM FCCP | 02:09:34 | 60.75 |
| 12 | 154 | B06 | 0.5 µM FCCP | 02:15:08 | 122.54 |
| 12 | 155 | B06 | 0.5 µM FCCP | 02:15:22 | 106.92 |
| 12 | 156 | B06 | 0.5 µM FCCP | 02:15:37 | 99.18 |
| 12 | 157 | B06 | 0.5 µM FCCP | 02:15:53 | 93.10 |
| 12 | 158 | B06 | 0.5 µM FCCP | 02:16:08 | 88.33 |
| 12 | 159 | B06 | 0.5 µM FCCP | 02:16:22 | 84.37 |
| 12 | 160 | B06 | 0.5 µM FCCP | 02:16:36 | 80.75 |
| 12 | 161 | B06 | 0.5 µM FCCP | 02:16:50 | 77.48 |
| 12 | 162 | B06 | 0.5 µM FCCP | 02:17:04 | 74.41 |
| 12 | 163 | B06 | 0.5 µM FCCP | 02:17:18 | 71.61 |
| 12 | 164 | B06 | 0.5 µM FCCP | 02:17:33 | 68.99 |
| 12 | 165 | B06 | 0.5 µM FCCP | 02:17:47 | 66.53 |
| 12 | 166 | B06 | 0.5 µM FCCP | 02:18:01 | 64.16 |
| 13 | 167 | B06 | 0.5 µM FCCP | 02:23:39 | 117.99 |
| 13 | 168 | B06 | 0.5 µM FCCP | 02:23:54 | 106.11 |
| 13 | 169 | B06 | 0.5 µM FCCP | 02:24:08 | 99.54 |
| 13 | 170 | B06 | 0.5 µM FCCP | 02:24:22 | 94.35 |
| 13 | 171 | B06 | 0.5 µM FCCP | 02:24:36 | 89.91 |
| 13 | 172 | B06 | 0.5 µM FCCP | 02:24:51 | 85.82 |
| 13 | 173 | B06 | 0.5 µM FCCP | 02:25:07 | 81.89 |
| 13 | 174 | B06 | 0.5 µM FCCP | 02:25:22 | 78.57 |
| 13 | 175 | B06 | 0.5 µM FCCP | 02:25:36 | 75.77 |
| 13 | 176 | B06 | 0.5 µM FCCP | 02:25:50 | 72.94 |
| 13 | 177 | B06 | 0.5 µM FCCP | 02:26:04 | 70.45 |
| 13 | 178 | B06 | 0.5 µM FCCP | 02:26:18 | 68.05 |
| 13 | 179 | B06 | 0.5 µM FCCP | 02:26:33 | 65.72 |
| 14 | 180 | B06 | 0.5 µM FCCP | 02:32:07 | 123.71 |
| 14 | 181 | B06 | 0.5 µM FCCP | 02:32:21 | 109.11 |
| 14 | 182 | B06 | 0.5 µM FCCP | 02:32:35 | 102.24 |
| 14 | 183 | B06 | 0.5 µM FCCP | 02:32:50 | 96.96 |
| 14 | 184 | B06 | 0.5 µM FCCP | 02:33:04 | 92.52 |
| 14 | 185 | B06 | 0.5 µM FCCP | 02:33:18 | 88.50 |
| 14 | 186 | B06 | 0.5 µM FCCP | 02:33:32 | 85.01 |
| 14 | 187 | B06 | 0.5 µM FCCP | 02:33:46 | 81.74 |
| 14 | 188 | B06 | 0.5 µM FCCP | 02:34:01 | 78.79 |
| 14 | 189 | B06 | 0.5 µM FCCP | 02:34:15 | 76.04 |
| 14 | 190 | B06 | 0.5 µM FCCP | 02:34:29 | 73.50 |
| 14 | 191 | B06 | 0.5 µM FCCP | 02:34:43 | 70.99 |
| 14 | 192 | B06 | 0.5 µM FCCP | 02:34:57 | 68.65 |
| 14 | 193 | B06 | 0.5 µM FCCP | 02:35:12 | 66.51 |
| 15 | 194 | B06 | 0.5 µM FCCP | 02:40:46 | 124.13 |
| 15 | 195 | B06 | 0.5 µM FCCP | 02:41:00 | 110.09 |
| 15 | 196 | B06 | 0.5 µM FCCP | 02:41:14 | 103.32 |
| 15 | 197 | B06 | 0.5 µM FCCP | 02:41:28 | 98.17 |
| 15 | 198 | B06 | 0.5 µM FCCP | 02:41:43 | 93.86 |
| 15 | 199 | B06 | 0.5 µM FCCP | 02:41:57 | 90.04 |
| 15 | 200 | B06 | 0.5 µM FCCP | 02:42:11 | 86.57 |
| 15 | 201 | B06 | 0.5 µM FCCP | 02:42:25 | 83.36 |
| 15 | 202 | B06 | 0.5 µM FCCP | 02:42:39 | 80.45 |
| 15 | 203 | B06 | 0.5 µM FCCP | 02:42:53 | 77.80 |
| 15 | 204 | B06 | 0.5 µM FCCP | 02:43:08 | 75.15 |
| 15 | 205 | B06 | 0.5 µM FCCP | 02:43:22 | 72.89 |
| 15 | 206 | B06 | 0.5 µM FCCP | 02:43:36 | 70.46 |
| 15 | 207 | B06 | 0.5 µM FCCP | 02:43:50 | 68.37 |
| 16 | 208 | B06 | 0.5 µM FCCP | 02:49:25 | 124.58 |
| 16 | 209 | B06 | 0.5 µM FCCP | 02:49:40 | 111.06 |
| 16 | 210 | B06 | 0.5 µM FCCP | 02:49:54 | 104.48 |
| 16 | 211 | B06 | 0.5 µM FCCP | 02:50:08 | 99.47 |
| 16 | 212 | B06 | 0.5 µM FCCP | 02:50:22 | 95.24 |
| 16 | 213 | B06 | 0.5 µM FCCP | 02:50:36 | 91.42 |
| 16 | 214 | B06 | 0.5 µM FCCP | 02:50:50 | 88.06 |
| 16 | 215 | B06 | 0.5 µM FCCP | 02:51:05 | 84.98 |
| 16 | 216 | B06 | 0.5 µM FCCP | 02:51:19 | 82.15 |
| 16 | 217 | B06 | 0.5 µM FCCP | 02:51:33 | 79.42 |
| 16 | 218 | B06 | 0.5 µM FCCP | 02:51:47 | 76.94 |
| 16 | 219 | B06 | 0.5 µM FCCP | 02:52:01 | 74.64 |
| 16 | 220 | B06 | 0.5 µM FCCP | 02:52:16 | 72.34 |
| 16 | 221 | B06 | 0.5 µM FCCP | 02:52:30 | 70.18 |
| 1 | 0 | C01 | 1 µM BAM15 | 00:40:06 | 138.83 |
| 1 | 1 | C01 | 1 µM BAM15 | 00:40:20 | 135.29 |
| 1 | 2 | C01 | 1 µM BAM15 | 00:40:34 | 133.51 |
| 1 | 3 | C01 | 1 µM BAM15 | 00:40:48 | 132.15 |
| 1 | 4 | C01 | 1 µM BAM15 | 00:41:02 | 130.82 |
| 1 | 5 | C01 | 1 µM BAM15 | 00:41:17 | 129.68 |
| 1 | 6 | C01 | 1 µM BAM15 | 00:41:31 | 128.51 |
| 1 | 7 | C01 | 1 µM BAM15 | 00:41:45 | 127.55 |
| 1 | 8 | C01 | 1 µM BAM15 | 00:41:59 | 126.53 |
| 1 | 9 | C01 | 1 µM BAM15 | 00:42:13 | 125.63 |
| 1 | 10 | C01 | 1 µM BAM15 | 00:42:28 | 124.66 |
| 1 | 11 | C01 | 1 µM BAM15 | 00:42:42 | 123.78 |
| 1 | 12 | C01 | 1 µM BAM15 | 00:42:56 | 122.89 |
| 1 | 13 | C01 | 1 µM BAM15 | 00:43:10 | 122.05 |
| 2 | 14 | C01 | 1 µM BAM15 | 00:48:43 | 139.38 |
| 2 | 15 | C01 | 1 µM BAM15 | 00:48:57 | 135.53 |
| 2 | 16 | C01 | 1 µM BAM15 | 00:49:12 | 133.65 |
| 2 | 17 | C01 | 1 µM BAM15 | 00:49:26 | 132.23 |
| 2 | 18 | C01 | 1 µM BAM15 | 00:49:40 | 130.90 |
| 2 | 19 | C01 | 1 µM BAM15 | 00:49:54 | 129.62 |
| 2 | 20 | C01 | 1 µM BAM15 | 00:50:08 | 128.48 |
| 2 | 21 | C01 | 1 µM BAM15 | 00:50:22 | 127.42 |
| 2 | 22 | C01 | 1 µM BAM15 | 00:50:37 | 126.41 |
| 2 | 23 | C01 | 1 µM BAM15 | 00:50:51 | 125.43 |
| 2 | 24 | C01 | 1 µM BAM15 | 00:51:05 | 124.46 |
| 2 | 25 | C01 | 1 µM BAM15 | 00:51:19 | 123.68 |
| 2 | 26 | C01 | 1 µM BAM15 | 00:51:33 | 122.70 |
| 2 | 27 | C01 | 1 µM BAM15 | 00:51:48 | 121.90 |
| 3 | 28 | C01 | 1 µM BAM15 | 00:57:21 | 139.14 |
| 3 | 29 | C01 | 1 µM BAM15 | 00:57:35 | 135.33 |
| 3 | 30 | C01 | 1 µM BAM15 | 00:57:49 | 133.49 |
| 3 | 31 | C01 | 1 µM BAM15 | 00:58:03 | 131.99 |
| 3 | 32 | C01 | 1 µM BAM15 | 00:58:17 | 130.68 |
| 3 | 33 | C01 | 1 µM BAM15 | 00:58:31 | 129.38 |
| 3 | 34 | C01 | 1 µM BAM15 | 00:58:46 | 128.35 |
| 3 | 35 | C01 | 1 µM BAM15 | 00:59:00 | 127.18 |
| 3 | 36 | C01 | 1 µM BAM15 | 00:59:14 | 126.17 |
| 3 | 37 | C01 | 1 µM BAM15 | 00:59:28 | 125.24 |
| 3 | 38 | C01 | 1 µM BAM15 | 00:59:42 | 124.33 |
| 3 | 39 | C01 | 1 µM BAM15 | 00:59:56 | 123.48 |
| 3 | 40 | C01 | 1 µM BAM15 | 01:00:11 | 122.64 |
| 3 | 41 | C01 | 1 µM BAM15 | 01:00:25 | 121.77 |
| 4 | 42 | C01 | 1 µM BAM15 | 01:06:04 | 131.58 |
| 4 | 43 | C01 | 1 µM BAM15 | 01:06:18 | 119.72 |
| 4 | 44 | C01 | 1 µM BAM15 | 01:06:33 | 113.86 |
| 4 | 45 | C01 | 1 µM BAM15 | 01:06:47 | 109.25 |
| 4 | 46 | C01 | 1 µM BAM15 | 01:07:01 | 105.28 |
| 4 | 47 | C01 | 1 µM BAM15 | 01:07:15 | 101.57 |
| 4 | 48 | C01 | 1 µM BAM15 | 01:07:29 | 98.30 |
| 4 | 49 | C01 | 1 µM BAM15 | 01:07:43 | 95.23 |
| 4 | 50 | C01 | 1 µM BAM15 | 01:07:58 | 92.38 |
| 4 | 51 | C01 | 1 µM BAM15 | 01:08:12 | 89.70 |
| 4 | 52 | C01 | 1 µM BAM15 | 01:08:26 | 87.15 |
| 4 | 53 | C01 | 1 µM BAM15 | 01:08:40 | 84.86 |
| 4 | 54 | C01 | 1 µM BAM15 | 01:08:54 | 82.54 |
| 4 | 55 | C01 | 1 µM BAM15 | 01:09:09 | 80.41 |
| 5 | 56 | C01 | 1 µM BAM15 | 01:14:42 | 125.28 |
| 5 | 57 | C01 | 1 µM BAM15 | 01:14:56 | 111.87 |
| 5 | 58 | C01 | 1 µM BAM15 | 01:15:10 | 106.00 |
| 5 | 59 | C01 | 1 µM BAM15 | 01:15:25 | 101.42 |
| 5 | 60 | C01 | 1 µM BAM15 | 01:15:39 | 97.45 |
| 5 | 61 | C01 | 1 µM BAM15 | 01:15:53 | 93.87 |
| 5 | 62 | C01 | 1 µM BAM15 | 01:16:07 | 90.73 |
| 5 | 63 | C01 | 1 µM BAM15 | 01:16:21 | 87.67 |
| 5 | 64 | C01 | 1 µM BAM15 | 01:16:35 | 84.96 |
| 5 | 65 | C01 | 1 µM BAM15 | 01:16:50 | 82.39 |
| 5 | 66 | C01 | 1 µM BAM15 | 01:17:04 | 79.88 |
| 5 | 67 | C01 | 1 µM BAM15 | 01:17:18 | 77.64 |
| 5 | 68 | C01 | 1 µM BAM15 | 01:17:32 | 75.45 |
| 5 | 69 | C01 | 1 µM BAM15 | 01:17:46 | 73.44 |
| 6 | 70 | C01 | 1 µM BAM15 | 01:23:20 | 122.31 |
| 6 | 71 | C01 | 1 µM BAM15 | 01:23:34 | 108.71 |
| 6 | 72 | C01 | 1 µM BAM15 | 01:23:48 | 102.93 |
| 6 | 73 | C01 | 1 µM BAM15 | 01:24:02 | 98.34 |
| 6 | 74 | C01 | 1 µM BAM15 | 01:24:16 | 94.44 |
| 6 | 75 | C01 | 1 µM BAM15 | 01:24:30 | 90.98 |
| 6 | 76 | C01 | 1 µM BAM15 | 01:24:44 | 87.84 |
| 6 | 77 | C01 | 1 µM BAM15 | 01:24:59 | 84.82 |
| 6 | 78 | C01 | 1 µM BAM15 | 01:25:13 | 82.11 |
| 6 | 79 | C01 | 1 µM BAM15 | 01:25:27 | 79.54 |
| 6 | 80 | C01 | 1 µM BAM15 | 01:25:41 | 77.09 |
| 6 | 81 | C01 | 1 µM BAM15 | 01:25:55 | 74.81 |
| 6 | 82 | C01 | 1 µM BAM15 | 01:26:10 | 72.59 |
| 6 | 83 | C01 | 1 µM BAM15 | 01:26:24 | 70.58 |
| 7 | 84 | C01 | 1 µM BAM15 | 01:31:58 | 120.94 |
| 7 | 85 | C01 | 1 µM BAM15 | 01:32:12 | 107.87 |
| 7 | 86 | C01 | 1 µM BAM15 | 01:32:26 | 102.11 |
| 7 | 87 | C01 | 1 µM BAM15 | 01:32:40 | 97.67 |
| 7 | 88 | C01 | 1 µM BAM15 | 01:32:54 | 93.76 |
| 7 | 89 | C01 | 1 µM BAM15 | 01:33:08 | 90.29 |
| 7 | 90 | C01 | 1 µM BAM15 | 01:33:23 | 87.08 |
| 7 | 91 | C01 | 1 µM BAM15 | 01:33:37 | 84.13 |
| 7 | 92 | C01 | 1 µM BAM15 | 01:33:51 | 81.37 |
| 7 | 93 | C01 | 1 µM BAM15 | 01:34:05 | 78.88 |
| 7 | 94 | C01 | 1 µM BAM15 | 01:34:19 | 76.40 |
| 7 | 95 | C01 | 1 µM BAM15 | 01:34:33 | 74.13 |
| 7 | 96 | C01 | 1 µM BAM15 | 01:34:48 | 71.93 |
| 7 | 97 | C01 | 1 µM BAM15 | 01:35:02 | 69.91 |
| 8 | 98 | C01 | 1 µM BAM15 | 01:40:36 | 122.07 |
| 8 | 99 | C01 | 1 µM BAM15 | 01:40:50 | 111.39 |
| 8 | 100 | C01 | 1 µM BAM15 | 01:41:04 | 105.94 |
| 8 | 101 | C01 | 1 µM BAM15 | 01:41:18 | 101.53 |
| 8 | 102 | C01 | 1 µM BAM15 | 01:41:32 | 97.63 |
| 8 | 103 | C01 | 1 µM BAM15 | 01:41:46 | 94.08 |
| 8 | 104 | C01 | 1 µM BAM15 | 01:42:00 | 90.84 |
| 8 | 105 | C01 | 1 µM BAM15 | 01:42:15 | 87.84 |
| 8 | 106 | C01 | 1 µM BAM15 | 01:42:29 | 84.99 |
| 8 | 107 | C01 | 1 µM BAM15 | 01:42:43 | 82.34 |
| 8 | 108 | C01 | 1 µM BAM15 | 01:42:57 | 79.79 |
| 8 | 109 | C01 | 1 µM BAM15 | 01:43:11 | 77.46 |
| 8 | 110 | C01 | 1 µM BAM15 | 01:43:26 | 75.15 |
| 8 | 111 | C01 | 1 µM BAM15 | 01:43:40 | 73.06 |
| 9 | 112 | C01 | 1 µM BAM15 | 01:49:14 | 121.89 |
| 9 | 113 | C01 | 1 µM BAM15 | 01:49:28 | 111.57 |
| 9 | 114 | C01 | 1 µM BAM15 | 01:49:42 | 106.09 |
| 9 | 115 | C01 | 1 µM BAM15 | 01:49:56 | 101.81 |
| 9 | 116 | C01 | 1 µM BAM15 | 01:50:10 | 98.01 |
| 9 | 117 | C01 | 1 µM BAM15 | 01:50:24 | 94.52 |
| 9 | 118 | C01 | 1 µM BAM15 | 01:50:38 | 91.33 |
| 9 | 119 | C01 | 1 µM BAM15 | 01:50:53 | 88.25 |
| 9 | 120 | C01 | 1 µM BAM15 | 01:51:07 | 85.48 |
| 9 | 121 | C01 | 1 µM BAM15 | 01:51:21 | 82.83 |
| 9 | 122 | C01 | 1 µM BAM15 | 01:51:35 | 80.37 |
| 9 | 123 | C01 | 1 µM BAM15 | 01:51:50 | 78.00 |
| 9 | 124 | C01 | 1 µM BAM15 | 01:52:04 | 75.78 |
| 9 | 125 | C01 | 1 µM BAM15 | 01:52:18 | 73.65 |
| 10 | 126 | C01 | 1 µM BAM15 | 01:57:52 | 122.09 |
| 10 | 127 | C01 | 1 µM BAM15 | 01:58:06 | 111.64 |
| 10 | 128 | C01 | 1 µM BAM15 | 01:58:20 | 106.21 |
| 10 | 129 | C01 | 1 µM BAM15 | 01:58:34 | 101.96 |
| 10 | 130 | C01 | 1 µM BAM15 | 01:58:48 | 98.19 |
| 10 | 131 | C01 | 1 µM BAM15 | 01:59:03 | 94.65 |
| 10 | 132 | C01 | 1 µM BAM15 | 01:59:17 | 91.47 |
| 10 | 133 | C01 | 1 µM BAM15 | 01:59:31 | 88.44 |
| 10 | 134 | C01 | 1 µM BAM15 | 01:59:45 | 85.80 |
| 10 | 135 | C01 | 1 µM BAM15 | 01:59:59 | 83.16 |
| 10 | 136 | C01 | 1 µM BAM15 | 02:00:14 | 80.69 |
| 10 | 137 | C01 | 1 µM BAM15 | 02:00:28 | 78.30 |
| 10 | 138 | C01 | 1 µM BAM15 | 02:00:42 | 76.11 |
| 10 | 139 | C01 | 1 µM BAM15 | 02:00:56 | 74.00 |
| 11 | 140 | C01 | 1 µM BAM15 | 02:06:30 | 122.38 |
| 11 | 141 | C01 | 1 µM BAM15 | 02:06:44 | 111.98 |
| 11 | 142 | C01 | 1 µM BAM15 | 02:06:58 | 106.65 |
| 11 | 143 | C01 | 1 µM BAM15 | 02:07:12 | 102.35 |
| 11 | 144 | C01 | 1 µM BAM15 | 02:07:26 | 98.67 |
| 11 | 145 | C01 | 1 µM BAM15 | 02:07:41 | 95.14 |
| 11 | 146 | C01 | 1 µM BAM15 | 02:07:55 | 92.02 |
| 11 | 147 | C01 | 1 µM BAM15 | 02:08:09 | 89.04 |
| 11 | 148 | C01 | 1 µM BAM15 | 02:08:23 | 86.27 |
| 11 | 149 | C01 | 1 µM BAM15 | 02:08:37 | 83.73 |
| 11 | 150 | C01 | 1 µM BAM15 | 02:08:52 | 81.25 |
| 11 | 151 | C01 | 1 µM BAM15 | 02:09:06 | 78.93 |
| 11 | 152 | C01 | 1 µM BAM15 | 02:09:20 | 76.71 |
| 11 | 153 | C01 | 1 µM BAM15 | 02:09:34 | 74.56 |
| 12 | 154 | C01 | 1 µM BAM15 | 02:15:08 | 122.53 |
| 12 | 155 | C01 | 1 µM BAM15 | 02:15:22 | 112.36 |
| 12 | 156 | C01 | 1 µM BAM15 | 02:15:37 | 106.84 |
| 12 | 157 | C01 | 1 µM BAM15 | 02:15:53 | 102.23 |
| 12 | 158 | C01 | 1 µM BAM15 | 02:16:08 | 98.38 |
| 12 | 159 | C01 | 1 µM BAM15 | 02:16:22 | 95.13 |
| 12 | 160 | C01 | 1 µM BAM15 | 02:16:36 | 91.98 |
| 12 | 161 | C01 | 1 µM BAM15 | 02:16:50 | 89.09 |
| 12 | 162 | C01 | 1 µM BAM15 | 02:17:04 | 86.39 |
| 12 | 163 | C01 | 1 µM BAM15 | 02:17:18 | 83.83 |
| 12 | 164 | C01 | 1 µM BAM15 | 02:17:33 | 81.39 |
| 12 | 165 | C01 | 1 µM BAM15 | 02:17:47 | 79.13 |
| 12 | 166 | C01 | 1 µM BAM15 | 02:18:01 | 76.92 |
| 13 | 167 | C01 | 1 µM BAM15 | 02:23:39 | 119.36 |
| 13 | 168 | C01 | 1 µM BAM15 | 02:23:54 | 111.33 |
| 13 | 169 | C01 | 1 µM BAM15 | 02:24:08 | 106.45 |
| 13 | 170 | C01 | 1 µM BAM15 | 02:24:22 | 102.37 |
| 13 | 171 | C01 | 1 µM BAM15 | 02:24:36 | 98.75 |
| 13 | 172 | C01 | 1 µM BAM15 | 02:24:51 | 95.19 |
| 13 | 173 | C01 | 1 µM BAM15 | 02:25:07 | 91.83 |
| 13 | 174 | C01 | 1 µM BAM15 | 02:25:22 | 88.89 |
| 13 | 175 | C01 | 1 µM BAM15 | 02:25:36 | 86.33 |
| 13 | 176 | C01 | 1 µM BAM15 | 02:25:50 | 83.84 |
| 13 | 177 | C01 | 1 µM BAM15 | 02:26:04 | 81.43 |
| 13 | 178 | C01 | 1 µM BAM15 | 02:26:18 | 79.16 |
| 13 | 179 | C01 | 1 µM BAM15 | 02:26:33 | 76.96 |
| 14 | 180 | C01 | 1 µM BAM15 | 02:32:07 | 123.07 |
| 14 | 181 | C01 | 1 µM BAM15 | 02:32:21 | 113.06 |
| 14 | 182 | C01 | 1 µM BAM15 | 02:32:35 | 107.92 |
| 14 | 183 | C01 | 1 µM BAM15 | 02:32:50 | 103.71 |
| 14 | 184 | C01 | 1 µM BAM15 | 02:33:04 | 100.17 |
| 14 | 185 | C01 | 1 µM BAM15 | 02:33:18 | 96.76 |
| 14 | 186 | C01 | 1 µM BAM15 | 02:33:32 | 93.64 |
| 14 | 187 | C01 | 1 µM BAM15 | 02:33:46 | 90.68 |
| 14 | 188 | C01 | 1 µM BAM15 | 02:34:01 | 88.05 |
| 14 | 189 | C01 | 1 µM BAM15 | 02:34:15 | 85.57 |
| 14 | 190 | C01 | 1 µM BAM15 | 02:34:29 | 83.16 |
| 14 | 191 | C01 | 1 µM BAM15 | 02:34:43 | 80.89 |
| 14 | 192 | C01 | 1 µM BAM15 | 02:34:57 | 78.57 |
| 14 | 193 | C01 | 1 µM BAM15 | 02:35:12 | 76.61 |
| 15 | 194 | C01 | 1 µM BAM15 | 02:40:46 | 123.57 |
| 15 | 195 | C01 | 1 µM BAM15 | 02:41:00 | 113.74 |
| 15 | 196 | C01 | 1 µM BAM15 | 02:41:14 | 108.57 |
| 15 | 197 | C01 | 1 µM BAM15 | 02:41:28 | 104.57 |
| 15 | 198 | C01 | 1 µM BAM15 | 02:41:43 | 100.90 |
| 15 | 199 | C01 | 1 µM BAM15 | 02:41:57 | 97.60 |
| 15 | 200 | C01 | 1 µM BAM15 | 02:42:11 | 94.50 |
| 15 | 201 | C01 | 1 µM BAM15 | 02:42:25 | 91.62 |
| 15 | 202 | C01 | 1 µM BAM15 | 02:42:39 | 89.05 |
| 15 | 203 | C01 | 1 µM BAM15 | 02:42:53 | 86.50 |
| 15 | 204 | C01 | 1 µM BAM15 | 02:43:08 | 84.04 |
| 15 | 205 | C01 | 1 µM BAM15 | 02:43:22 | 81.85 |
| 15 | 206 | C01 | 1 µM BAM15 | 02:43:36 | 79.57 |
| 15 | 207 | C01 | 1 µM BAM15 | 02:43:50 | 77.56 |
| 16 | 208 | C01 | 1 µM BAM15 | 02:49:25 | 123.85 |
| 16 | 209 | C01 | 1 µM BAM15 | 02:49:40 | 114.14 |
| 16 | 210 | C01 | 1 µM BAM15 | 02:49:54 | 109.10 |
| 16 | 211 | C01 | 1 µM BAM15 | 02:50:08 | 105.02 |
| 16 | 212 | C01 | 1 µM BAM15 | 02:50:22 | 101.51 |
| 16 | 213 | C01 | 1 µM BAM15 | 02:50:36 | 98.18 |
| 16 | 214 | C01 | 1 µM BAM15 | 02:50:50 | 95.16 |
| 16 | 215 | C01 | 1 µM BAM15 | 02:51:05 | 92.32 |
| 16 | 216 | C01 | 1 µM BAM15 | 02:51:19 | 89.69 |
| 16 | 217 | C01 | 1 µM BAM15 | 02:51:33 | 87.20 |
| 16 | 218 | C01 | 1 µM BAM15 | 02:51:47 | 84.90 |
| 16 | 219 | C01 | 1 µM BAM15 | 02:52:01 | 82.65 |
| 16 | 220 | C01 | 1 µM BAM15 | 02:52:16 | 80.41 |
| 16 | 221 | C01 | 1 µM BAM15 | 02:52:30 | 78.36 |
| 1 | 0 | C02 | 1 µM BAM15 | 00:40:06 | 134.90 |
| 1 | 1 | C02 | 1 µM BAM15 | 00:40:20 | 129.16 |
| 1 | 2 | C02 | 1 µM BAM15 | 00:40:34 | 126.71 |
| 1 | 3 | C02 | 1 µM BAM15 | 00:40:48 | 124.67 |
| 1 | 4 | C02 | 1 µM BAM15 | 00:41:02 | 123.00 |
| 1 | 5 | C02 | 1 µM BAM15 | 00:41:17 | 121.46 |
| 1 | 6 | C02 | 1 µM BAM15 | 00:41:31 | 119.99 |
| 1 | 7 | C02 | 1 µM BAM15 | 00:41:45 | 118.71 |
| 1 | 8 | C02 | 1 µM BAM15 | 00:41:59 | 117.45 |
| 1 | 9 | C02 | 1 µM BAM15 | 00:42:13 | 116.37 |
| 1 | 10 | C02 | 1 µM BAM15 | 00:42:28 | 115.10 |
| 1 | 11 | C02 | 1 µM BAM15 | 00:42:42 | 114.05 |
| 1 | 12 | C02 | 1 µM BAM15 | 00:42:56 | 112.98 |
| 1 | 13 | C02 | 1 µM BAM15 | 00:43:10 | 112.03 |
| 2 | 14 | C02 | 1 µM BAM15 | 00:48:43 | 136.58 |
| 2 | 15 | C02 | 1 µM BAM15 | 00:48:57 | 131.74 |
| 2 | 16 | C02 | 1 µM BAM15 | 00:49:12 | 129.43 |
| 2 | 17 | C02 | 1 µM BAM15 | 00:49:26 | 127.48 |
| 2 | 18 | C02 | 1 µM BAM15 | 00:49:40 | 125.85 |
| 2 | 19 | C02 | 1 µM BAM15 | 00:49:54 | 124.21 |
| 2 | 20 | C02 | 1 µM BAM15 | 00:50:08 | 122.90 |
| 2 | 21 | C02 | 1 µM BAM15 | 00:50:22 | 121.48 |
| 2 | 22 | C02 | 1 µM BAM15 | 00:50:37 | 120.23 |
| 2 | 23 | C02 | 1 µM BAM15 | 00:50:51 | 119.05 |
| 2 | 24 | C02 | 1 µM BAM15 | 00:51:05 | 117.83 |
| 2 | 25 | C02 | 1 µM BAM15 | 00:51:19 | 116.81 |
| 2 | 26 | C02 | 1 µM BAM15 | 00:51:33 | 115.65 |
| 2 | 27 | C02 | 1 µM BAM15 | 00:51:48 | 114.60 |
| 3 | 28 | C02 | 1 µM BAM15 | 00:57:21 | 137.17 |
| 3 | 29 | C02 | 1 µM BAM15 | 00:57:35 | 132.54 |
| 3 | 30 | C02 | 1 µM BAM15 | 00:57:49 | 130.20 |
| 3 | 31 | C02 | 1 µM BAM15 | 00:58:03 | 128.30 |
| 3 | 32 | C02 | 1 µM BAM15 | 00:58:17 | 126.65 |
| 3 | 33 | C02 | 1 µM BAM15 | 00:58:31 | 125.09 |
| 3 | 34 | C02 | 1 µM BAM15 | 00:58:46 | 123.76 |
| 3 | 35 | C02 | 1 µM BAM15 | 00:59:00 | 122.35 |
| 3 | 36 | C02 | 1 µM BAM15 | 00:59:14 | 121.13 |
| 3 | 37 | C02 | 1 µM BAM15 | 00:59:28 | 119.94 |
| 3 | 38 | C02 | 1 µM BAM15 | 00:59:42 | 118.76 |
| 3 | 39 | C02 | 1 µM BAM15 | 00:59:56 | 117.64 |
| 3 | 40 | C02 | 1 µM BAM15 | 01:00:11 | 116.62 |
| 3 | 41 | C02 | 1 µM BAM15 | 01:00:25 | 115.57 |
| 4 | 42 | C02 | 1 µM BAM15 | 01:06:04 | 127.35 |
| 4 | 43 | C02 | 1 µM BAM15 | 01:06:18 | 111.52 |
| 4 | 44 | C02 | 1 µM BAM15 | 01:06:33 | 103.41 |
| 4 | 45 | C02 | 1 µM BAM15 | 01:06:47 | 97.20 |
| 4 | 46 | C02 | 1 µM BAM15 | 01:07:01 | 91.65 |
| 4 | 47 | C02 | 1 µM BAM15 | 01:07:15 | 86.52 |
| 4 | 48 | C02 | 1 µM BAM15 | 01:07:29 | 81.79 |
| 4 | 49 | C02 | 1 µM BAM15 | 01:07:43 | 77.34 |
| 4 | 50 | C02 | 1 µM BAM15 | 01:07:58 | 73.31 |
| 4 | 51 | C02 | 1 µM BAM15 | 01:08:12 | 69.47 |
| 4 | 52 | C02 | 1 µM BAM15 | 01:08:26 | 65.70 |
| 4 | 53 | C02 | 1 µM BAM15 | 01:08:40 | 62.26 |
| 4 | 54 | C02 | 1 µM BAM15 | 01:08:54 | 58.87 |
| 4 | 55 | C02 | 1 µM BAM15 | 01:09:09 | 55.77 |
| 5 | 56 | C02 | 1 µM BAM15 | 01:14:42 | 119.55 |
| 5 | 57 | C02 | 1 µM BAM15 | 01:14:56 | 101.59 |
| 5 | 58 | C02 | 1 µM BAM15 | 01:15:10 | 93.38 |
| 5 | 59 | C02 | 1 µM BAM15 | 01:15:25 | 87.13 |
| 5 | 60 | C02 | 1 µM BAM15 | 01:15:39 | 81.59 |
| 5 | 61 | C02 | 1 µM BAM15 | 01:15:53 | 76.49 |
| 5 | 62 | C02 | 1 µM BAM15 | 01:16:07 | 71.86 |
| 5 | 63 | C02 | 1 µM BAM15 | 01:16:21 | 67.51 |
| 5 | 64 | C02 | 1 µM BAM15 | 01:16:35 | 63.56 |
| 5 | 65 | C02 | 1 µM BAM15 | 01:16:50 | 59.72 |
| 5 | 66 | C02 | 1 µM BAM15 | 01:17:04 | 56.08 |
| 5 | 67 | C02 | 1 µM BAM15 | 01:17:18 | 52.62 |
| 5 | 68 | C02 | 1 µM BAM15 | 01:17:32 | 49.36 |
| 5 | 69 | C02 | 1 µM BAM15 | 01:17:46 | 46.34 |
| 6 | 70 | C02 | 1 µM BAM15 | 01:23:20 | 116.12 |
| 6 | 71 | C02 | 1 µM BAM15 | 01:23:34 | 97.86 |
| 6 | 72 | C02 | 1 µM BAM15 | 01:23:48 | 89.80 |
| 6 | 73 | C02 | 1 µM BAM15 | 01:24:02 | 83.39 |
| 6 | 74 | C02 | 1 µM BAM15 | 01:24:16 | 77.92 |
| 6 | 75 | C02 | 1 µM BAM15 | 01:24:30 | 72.85 |
| 6 | 76 | C02 | 1 µM BAM15 | 01:24:44 | 68.27 |
| 6 | 77 | C02 | 1 µM BAM15 | 01:24:59 | 63.87 |
| 6 | 78 | C02 | 1 µM BAM15 | 01:25:13 | 59.88 |
| 6 | 79 | C02 | 1 µM BAM15 | 01:25:27 | 56.08 |
| 6 | 80 | C02 | 1 µM BAM15 | 01:25:41 | 52.41 |
| 6 | 81 | C02 | 1 µM BAM15 | 01:25:55 | 49.05 |
| 6 | 82 | C02 | 1 µM BAM15 | 01:26:10 | 45.74 |
| 6 | 83 | C02 | 1 µM BAM15 | 01:26:24 | 42.71 |
| 7 | 84 | C02 | 1 µM BAM15 | 01:31:58 | 114.00 |
| 7 | 85 | C02 | 1 µM BAM15 | 01:32:12 | 95.96 |
| 7 | 86 | C02 | 1 µM BAM15 | 01:32:26 | 87.89 |
| 7 | 87 | C02 | 1 µM BAM15 | 01:32:40 | 81.64 |
| 7 | 88 | C02 | 1 µM BAM15 | 01:32:54 | 76.10 |
| 7 | 89 | C02 | 1 µM BAM15 | 01:33:08 | 71.08 |
| 7 | 90 | C02 | 1 µM BAM15 | 01:33:23 | 66.45 |
| 7 | 91 | C02 | 1 µM BAM15 | 01:33:37 | 62.07 |
| 7 | 92 | C02 | 1 µM BAM15 | 01:33:51 | 58.07 |
| 7 | 93 | C02 | 1 µM BAM15 | 01:34:05 | 54.27 |
| 7 | 94 | C02 | 1 µM BAM15 | 01:34:19 | 50.64 |
| 7 | 95 | C02 | 1 µM BAM15 | 01:34:33 | 47.30 |
| 7 | 96 | C02 | 1 µM BAM15 | 01:34:48 | 44.05 |
| 7 | 97 | C02 | 1 µM BAM15 | 01:35:02 | 41.03 |
| 8 | 98 | C02 | 1 µM BAM15 | 01:40:36 | 112.86 |
| 8 | 99 | C02 | 1 µM BAM15 | 01:40:50 | 94.90 |
| 8 | 100 | C02 | 1 µM BAM15 | 01:41:04 | 86.94 |
| 8 | 101 | C02 | 1 µM BAM15 | 01:41:18 | 80.59 |
| 8 | 102 | C02 | 1 µM BAM15 | 01:41:32 | 75.12 |
| 8 | 103 | C02 | 1 µM BAM15 | 01:41:46 | 70.14 |
| 8 | 104 | C02 | 1 µM BAM15 | 01:42:00 | 65.55 |
| 8 | 105 | C02 | 1 µM BAM15 | 01:42:15 | 61.23 |
| 8 | 106 | C02 | 1 µM BAM15 | 01:42:29 | 57.17 |
| 8 | 107 | C02 | 1 µM BAM15 | 01:42:43 | 53.38 |
| 8 | 108 | C02 | 1 µM BAM15 | 01:42:57 | 49.78 |
| 8 | 109 | C02 | 1 µM BAM15 | 01:43:11 | 46.42 |
| 8 | 110 | C02 | 1 µM BAM15 | 01:43:26 | 43.14 |
| 8 | 111 | C02 | 1 µM BAM15 | 01:43:40 | 40.21 |
| 9 | 112 | C02 | 1 µM BAM15 | 01:49:14 | 112.64 |
| 9 | 113 | C02 | 1 µM BAM15 | 01:49:28 | 94.92 |
| 9 | 114 | C02 | 1 µM BAM15 | 01:49:42 | 86.87 |
| 9 | 115 | C02 | 1 µM BAM15 | 01:49:56 | 80.61 |
| 9 | 116 | C02 | 1 µM BAM15 | 01:50:10 | 75.14 |
| 9 | 117 | C02 | 1 µM BAM15 | 01:50:24 | 70.18 |
| 9 | 118 | C02 | 1 µM BAM15 | 01:50:38 | 65.61 |
| 9 | 119 | C02 | 1 µM BAM15 | 01:50:53 | 61.23 |
| 9 | 120 | C02 | 1 µM BAM15 | 01:51:07 | 57.24 |
| 9 | 121 | C02 | 1 µM BAM15 | 01:51:21 | 53.47 |
| 9 | 122 | C02 | 1 µM BAM15 | 01:51:35 | 49.87 |
| 9 | 123 | C02 | 1 µM BAM15 | 01:51:50 | 46.48 |
| 9 | 124 | C02 | 1 µM BAM15 | 01:52:04 | 43.27 |
| 9 | 125 | C02 | 1 µM BAM15 | 01:52:18 | 40.25 |
| 10 | 126 | C02 | 1 µM BAM15 | 01:57:52 | 115.03 |
| 10 | 127 | C02 | 1 µM BAM15 | 01:58:06 | 99.42 |
| 10 | 128 | C02 | 1 µM BAM15 | 01:58:20 | 91.45 |
| 10 | 129 | C02 | 1 µM BAM15 | 01:58:34 | 85.10 |
| 10 | 130 | C02 | 1 µM BAM15 | 01:58:48 | 79.45 |
| 10 | 131 | C02 | 1 µM BAM15 | 01:59:03 | 74.21 |
| 10 | 132 | C02 | 1 µM BAM15 | 01:59:17 | 69.48 |
| 10 | 133 | C02 | 1 µM BAM15 | 01:59:31 | 64.93 |
| 10 | 134 | C02 | 1 µM BAM15 | 01:59:45 | 60.85 |
| 10 | 135 | C02 | 1 µM BAM15 | 01:59:59 | 56.84 |
| 10 | 136 | C02 | 1 µM BAM15 | 02:00:14 | 53.10 |
| 10 | 137 | C02 | 1 µM BAM15 | 02:00:28 | 49.58 |
| 10 | 138 | C02 | 1 µM BAM15 | 02:00:42 | 46.18 |
| 10 | 139 | C02 | 1 µM BAM15 | 02:00:56 | 43.05 |
| 11 | 140 | C02 | 1 µM BAM15 | 02:06:30 | 115.91 |
| 11 | 141 | C02 | 1 µM BAM15 | 02:06:44 | 101.38 |
| 11 | 142 | C02 | 1 µM BAM15 | 02:06:58 | 93.49 |
| 11 | 143 | C02 | 1 µM BAM15 | 02:07:12 | 87.07 |
| 11 | 144 | C02 | 1 µM BAM15 | 02:07:26 | 81.46 |
| 11 | 145 | C02 | 1 µM BAM15 | 02:07:41 | 76.18 |
| 11 | 146 | C02 | 1 µM BAM15 | 02:07:55 | 71.42 |
| 11 | 147 | C02 | 1 µM BAM15 | 02:08:09 | 66.89 |
| 11 | 148 | C02 | 1 µM BAM15 | 02:08:23 | 62.64 |
| 11 | 149 | C02 | 1 µM BAM15 | 02:08:37 | 58.68 |
| 11 | 150 | C02 | 1 µM BAM15 | 02:08:52 | 54.87 |
| 11 | 151 | C02 | 1 µM BAM15 | 02:09:06 | 51.26 |
| 11 | 152 | C02 | 1 µM BAM15 | 02:09:20 | 47.84 |
| 11 | 153 | C02 | 1 µM BAM15 | 02:09:34 | 44.59 |
| 12 | 154 | C02 | 1 µM BAM15 | 02:15:08 | 116.66 |
| 12 | 155 | C02 | 1 µM BAM15 | 02:15:22 | 102.45 |
| 12 | 156 | C02 | 1 µM BAM15 | 02:15:37 | 94.23 |
| 12 | 157 | C02 | 1 µM BAM15 | 02:15:53 | 87.28 |
| 12 | 158 | C02 | 1 µM BAM15 | 02:16:08 | 81.54 |
| 12 | 159 | C02 | 1 µM BAM15 | 02:16:22 | 76.58 |
| 12 | 160 | C02 | 1 µM BAM15 | 02:16:36 | 71.86 |
| 12 | 161 | C02 | 1 µM BAM15 | 02:16:50 | 67.41 |
| 12 | 162 | C02 | 1 µM BAM15 | 02:17:04 | 63.26 |
| 12 | 163 | C02 | 1 µM BAM15 | 02:17:18 | 59.27 |
| 12 | 164 | C02 | 1 µM BAM15 | 02:17:33 | 55.58 |
| 12 | 165 | C02 | 1 µM BAM15 | 02:17:47 | 52.00 |
| 12 | 166 | C02 | 1 µM BAM15 | 02:18:01 | 48.60 |
| 13 | 167 | C02 | 1 µM BAM15 | 02:23:39 | 113.24 |
| 13 | 168 | C02 | 1 µM BAM15 | 02:23:54 | 101.61 |
| 13 | 169 | C02 | 1 µM BAM15 | 02:24:08 | 94.32 |
| 13 | 170 | C02 | 1 µM BAM15 | 02:24:22 | 88.26 |
| 13 | 171 | C02 | 1 µM BAM15 | 02:24:36 | 82.73 |
| 13 | 172 | C02 | 1 µM BAM15 | 02:24:51 | 77.40 |
| 13 | 173 | C02 | 1 µM BAM15 | 02:25:07 | 72.19 |
| 13 | 174 | C02 | 1 µM BAM15 | 02:25:22 | 67.75 |
| 13 | 175 | C02 | 1 µM BAM15 | 02:25:36 | 63.79 |
| 13 | 176 | C02 | 1 µM BAM15 | 02:25:50 | 59.85 |
| 13 | 177 | C02 | 1 µM BAM15 | 02:26:04 | 56.22 |
| 13 | 178 | C02 | 1 µM BAM15 | 02:26:18 | 52.68 |
| 13 | 179 | C02 | 1 µM BAM15 | 02:26:33 | 49.30 |
| 14 | 180 | C02 | 1 µM BAM15 | 02:32:07 | 117.88 |
| 14 | 181 | C02 | 1 µM BAM15 | 02:32:21 | 103.68 |
| 14 | 182 | C02 | 1 µM BAM15 | 02:32:35 | 96.01 |
| 14 | 183 | C02 | 1 µM BAM15 | 02:32:50 | 89.81 |
| 14 | 184 | C02 | 1 µM BAM15 | 02:33:04 | 84.36 |
| 14 | 185 | C02 | 1 µM BAM15 | 02:33:18 | 79.21 |
| 14 | 186 | C02 | 1 µM BAM15 | 02:33:32 | 74.54 |
| 14 | 187 | C02 | 1 µM BAM15 | 02:33:46 | 70.10 |
| 14 | 188 | C02 | 1 µM BAM15 | 02:34:01 | 66.11 |
| 14 | 189 | C02 | 1 µM BAM15 | 02:34:15 | 62.18 |
| 14 | 190 | C02 | 1 µM BAM15 | 02:34:29 | 58.46 |
| 14 | 191 | C02 | 1 µM BAM15 | 02:34:43 | 54.92 |
| 14 | 192 | C02 | 1 µM BAM15 | 02:34:57 | 51.47 |
| 14 | 193 | C02 | 1 µM BAM15 | 02:35:12 | 48.34 |
| 15 | 194 | C02 | 1 µM BAM15 | 02:40:46 | 118.07 |
| 15 | 195 | C02 | 1 µM BAM15 | 02:41:00 | 104.09 |
| 15 | 196 | C02 | 1 µM BAM15 | 02:41:14 | 96.50 |
| 15 | 197 | C02 | 1 µM BAM15 | 02:41:28 | 90.35 |
| 15 | 198 | C02 | 1 µM BAM15 | 02:41:43 | 84.92 |
| 15 | 199 | C02 | 1 µM BAM15 | 02:41:57 | 79.98 |
| 15 | 200 | C02 | 1 µM BAM15 | 02:42:11 | 75.36 |
| 15 | 201 | C02 | 1 µM BAM15 | 02:42:25 | 71.01 |
| 15 | 202 | C02 | 1 µM BAM15 | 02:42:39 | 66.98 |
| 15 | 203 | C02 | 1 µM BAM15 | 02:42:53 | 63.18 |
| 15 | 204 | C02 | 1 µM BAM15 | 02:43:08 | 59.43 |
| 15 | 205 | C02 | 1 µM BAM15 | 02:43:22 | 56.01 |
| 15 | 206 | C02 | 1 µM BAM15 | 02:43:36 | 52.58 |
| 15 | 207 | C02 | 1 µM BAM15 | 02:43:50 | 49.43 |
| 16 | 208 | C02 | 1 µM BAM15 | 02:49:25 | 118.39 |
| 16 | 209 | C02 | 1 µM BAM15 | 02:49:40 | 104.68 |
| 16 | 210 | C02 | 1 µM BAM15 | 02:49:54 | 97.14 |
| 16 | 211 | C02 | 1 µM BAM15 | 02:50:08 | 91.13 |
| 16 | 212 | C02 | 1 µM BAM15 | 02:50:22 | 85.78 |
| 16 | 213 | C02 | 1 µM BAM15 | 02:50:36 | 80.79 |
| 16 | 214 | C02 | 1 µM BAM15 | 02:50:50 | 76.32 |
| 16 | 215 | C02 | 1 µM BAM15 | 02:51:05 | 71.95 |
| 16 | 216 | C02 | 1 µM BAM15 | 02:51:19 | 67.99 |
| 16 | 217 | C02 | 1 µM BAM15 | 02:51:33 | 64.23 |
| 16 | 218 | C02 | 1 µM BAM15 | 02:51:47 | 60.56 |
| 16 | 219 | C02 | 1 µM BAM15 | 02:52:01 | 57.19 |
| 16 | 220 | C02 | 1 µM BAM15 | 02:52:16 | 53.77 |
| 16 | 221 | C02 | 1 µM BAM15 | 02:52:30 | 50.66 |
| 1 | 0 | C03 | 1 µM BAM15 | 00:40:06 | 131.24 |
| 1 | 1 | C03 | 1 µM BAM15 | 00:40:20 | 122.34 |
| 1 | 2 | C03 | 1 µM BAM15 | 00:40:34 | 119.44 |
| 1 | 3 | C03 | 1 µM BAM15 | 00:40:48 | 117.28 |
| 1 | 4 | C03 | 1 µM BAM15 | 00:41:02 | 115.49 |
| 1 | 5 | C03 | 1 µM BAM15 | 00:41:17 | 113.86 |
| 1 | 6 | C03 | 1 µM BAM15 | 00:41:31 | 112.42 |
| 1 | 7 | C03 | 1 µM BAM15 | 00:41:45 | 111.11 |
| 1 | 8 | C03 | 1 µM BAM15 | 00:41:59 | 109.76 |
| 1 | 9 | C03 | 1 µM BAM15 | 00:42:13 | 108.60 |
| 1 | 10 | C03 | 1 µM BAM15 | 00:42:28 | 107.37 |
| 1 | 11 | C03 | 1 µM BAM15 | 00:42:42 | 106.25 |
| 1 | 12 | C03 | 1 µM BAM15 | 00:42:56 | 105.11 |
| 1 | 13 | C03 | 1 µM BAM15 | 00:43:10 | 104.07 |
| 2 | 14 | C03 | 1 µM BAM15 | 00:48:43 | 133.30 |
| 2 | 15 | C03 | 1 µM BAM15 | 00:48:57 | 127.37 |
| 2 | 16 | C03 | 1 µM BAM15 | 00:49:12 | 124.65 |
| 2 | 17 | C03 | 1 µM BAM15 | 00:49:26 | 122.51 |
| 2 | 18 | C03 | 1 µM BAM15 | 00:49:40 | 120.67 |
| 2 | 19 | C03 | 1 µM BAM15 | 00:49:54 | 118.95 |
| 2 | 20 | C03 | 1 µM BAM15 | 00:50:08 | 117.41 |
| 2 | 21 | C03 | 1 µM BAM15 | 00:50:22 | 115.87 |
| 2 | 22 | C03 | 1 µM BAM15 | 00:50:37 | 114.53 |
| 2 | 23 | C03 | 1 µM BAM15 | 00:50:51 | 113.21 |
| 2 | 24 | C03 | 1 µM BAM15 | 00:51:05 | 111.84 |
| 2 | 25 | C03 | 1 µM BAM15 | 00:51:19 | 110.76 |
| 2 | 26 | C03 | 1 µM BAM15 | 00:51:33 | 109.46 |
| 2 | 27 | C03 | 1 µM BAM15 | 00:51:48 | 108.41 |
| 3 | 28 | C03 | 1 µM BAM15 | 00:57:21 | 134.15 |
| 3 | 29 | C03 | 1 µM BAM15 | 00:57:35 | 128.78 |
| 3 | 30 | C03 | 1 µM BAM15 | 00:57:49 | 126.17 |
| 3 | 31 | C03 | 1 µM BAM15 | 00:58:03 | 124.06 |
| 3 | 32 | C03 | 1 µM BAM15 | 00:58:17 | 122.20 |
| 3 | 33 | C03 | 1 µM BAM15 | 00:58:31 | 120.54 |
| 3 | 34 | C03 | 1 µM BAM15 | 00:58:46 | 119.04 |
| 3 | 35 | C03 | 1 µM BAM15 | 00:59:00 | 117.52 |
| 3 | 36 | C03 | 1 µM BAM15 | 00:59:14 | 116.13 |
| 3 | 37 | C03 | 1 µM BAM15 | 00:59:28 | 114.81 |
| 3 | 38 | C03 | 1 µM BAM15 | 00:59:42 | 113.49 |
| 3 | 39 | C03 | 1 µM BAM15 | 00:59:56 | 112.37 |
| 3 | 40 | C03 | 1 µM BAM15 | 01:00:11 | 111.18 |
| 3 | 41 | C03 | 1 µM BAM15 | 01:00:25 | 110.02 |
| 4 | 42 | C03 | 1 µM BAM15 | 01:06:04 | 123.87 |
| 4 | 43 | C03 | 1 µM BAM15 | 01:06:18 | 105.13 |
| 4 | 44 | C03 | 1 µM BAM15 | 01:06:33 | 95.92 |
| 4 | 45 | C03 | 1 µM BAM15 | 01:06:47 | 88.70 |
| 4 | 46 | C03 | 1 µM BAM15 | 01:07:01 | 82.45 |
| 4 | 47 | C03 | 1 µM BAM15 | 01:07:15 | 76.43 |
| 4 | 48 | C03 | 1 µM BAM15 | 01:07:29 | 71.05 |
| 4 | 49 | C03 | 1 µM BAM15 | 01:07:43 | 65.85 |
| 4 | 50 | C03 | 1 µM BAM15 | 01:07:58 | 61.15 |
| 4 | 51 | C03 | 1 µM BAM15 | 01:08:12 | 56.64 |
| 4 | 52 | C03 | 1 µM BAM15 | 01:08:26 | 52.29 |
| 4 | 53 | C03 | 1 µM BAM15 | 01:08:40 | 48.32 |
| 4 | 54 | C03 | 1 µM BAM15 | 01:08:54 | 44.47 |
| 4 | 55 | C03 | 1 µM BAM15 | 01:09:09 | 41.03 |
| 5 | 56 | C03 | 1 µM BAM15 | 01:14:42 | 115.57 |
| 5 | 57 | C03 | 1 µM BAM15 | 01:14:56 | 94.51 |
| 5 | 58 | C03 | 1 µM BAM15 | 01:15:10 | 85.08 |
| 5 | 59 | C03 | 1 µM BAM15 | 01:15:25 | 77.92 |
| 5 | 60 | C03 | 1 µM BAM15 | 01:15:39 | 71.59 |
| 5 | 61 | C03 | 1 µM BAM15 | 01:15:53 | 65.75 |
| 5 | 62 | C03 | 1 µM BAM15 | 01:16:07 | 60.50 |
| 5 | 63 | C03 | 1 µM BAM15 | 01:16:21 | 55.46 |
| 5 | 64 | C03 | 1 µM BAM15 | 01:16:35 | 50.99 |
| 5 | 65 | C03 | 1 µM BAM15 | 01:16:50 | 46.65 |
| 5 | 66 | C03 | 1 µM BAM15 | 01:17:04 | 42.59 |
| 5 | 67 | C03 | 1 µM BAM15 | 01:17:18 | 38.98 |
| 5 | 68 | C03 | 1 µM BAM15 | 01:17:32 | 35.88 |
| 5 | 69 | C03 | 1 µM BAM15 | 01:17:46 | 33.36 |
| 6 | 70 | C03 | 1 µM BAM15 | 01:23:20 | 111.97 |
| 6 | 71 | C03 | 1 µM BAM15 | 01:23:34 | 91.04 |
| 6 | 72 | C03 | 1 µM BAM15 | 01:23:48 | 81.94 |
| 6 | 73 | C03 | 1 µM BAM15 | 01:24:02 | 74.81 |
| 6 | 74 | C03 | 1 µM BAM15 | 01:24:16 | 68.66 |
| 6 | 75 | C03 | 1 µM BAM15 | 01:24:30 | 63.00 |
| 6 | 76 | C03 | 1 µM BAM15 | 01:24:44 | 57.84 |
| 6 | 77 | C03 | 1 µM BAM15 | 01:24:59 | 52.94 |
| 6 | 78 | C03 | 1 µM BAM15 | 01:25:13 | 48.46 |
| 6 | 79 | C03 | 1 µM BAM15 | 01:25:27 | 44.28 |
| 6 | 80 | C03 | 1 µM BAM15 | 01:25:41 | 40.35 |
| 6 | 81 | C03 | 1 µM BAM15 | 01:25:55 | 37.01 |
| 6 | 82 | C03 | 1 µM BAM15 | 01:26:10 | 34.06 |
| 6 | 83 | C03 | 1 µM BAM15 | 01:26:24 | 31.71 |
| 7 | 84 | C03 | 1 µM BAM15 | 01:31:58 | 110.24 |
| 7 | 85 | C03 | 1 µM BAM15 | 01:32:12 | 89.73 |
| 7 | 86 | C03 | 1 µM BAM15 | 01:32:26 | 80.65 |
| 7 | 87 | C03 | 1 µM BAM15 | 01:32:40 | 73.72 |
| 7 | 88 | C03 | 1 µM BAM15 | 01:32:54 | 67.63 |
| 7 | 89 | C03 | 1 µM BAM15 | 01:33:08 | 62.05 |
| 7 | 90 | C03 | 1 µM BAM15 | 01:33:23 | 56.95 |
| 7 | 91 | C03 | 1 µM BAM15 | 01:33:37 | 52.14 |
| 7 | 92 | C03 | 1 µM BAM15 | 01:33:51 | 47.73 |
| 7 | 93 | C03 | 1 µM BAM15 | 01:34:05 | 43.69 |
| 7 | 94 | C03 | 1 µM BAM15 | 01:34:19 | 39.83 |
| 7 | 95 | C03 | 1 µM BAM15 | 01:34:33 | 36.55 |
| 7 | 96 | C03 | 1 µM BAM15 | 01:34:48 | 33.70 |
| 7 | 97 | C03 | 1 µM BAM15 | 01:35:02 | 31.40 |
| 8 | 98 | C03 | 1 µM BAM15 | 01:40:36 | 109.68 |
| 8 | 99 | C03 | 1 µM BAM15 | 01:40:50 | 89.31 |
| 8 | 100 | C03 | 1 µM BAM15 | 01:41:04 | 80.32 |
| 8 | 101 | C03 | 1 µM BAM15 | 01:41:18 | 73.40 |
| 8 | 102 | C03 | 1 µM BAM15 | 01:41:32 | 67.40 |
| 8 | 103 | C03 | 1 µM BAM15 | 01:41:46 | 61.90 |
| 8 | 104 | C03 | 1 µM BAM15 | 01:42:00 | 56.89 |
| 8 | 105 | C03 | 1 µM BAM15 | 01:42:15 | 52.16 |
| 8 | 106 | C03 | 1 µM BAM15 | 01:42:29 | 47.77 |
| 8 | 107 | C03 | 1 µM BAM15 | 01:42:43 | 43.70 |
| 8 | 108 | C03 | 1 µM BAM15 | 01:42:57 | 39.93 |
| 8 | 109 | C03 | 1 µM BAM15 | 01:43:11 | 36.63 |
| 8 | 110 | C03 | 1 µM BAM15 | 01:43:26 | 33.71 |
| 8 | 111 | C03 | 1 µM BAM15 | 01:43:40 | 31.42 |
| 9 | 112 | C03 | 1 µM BAM15 | 01:49:14 | 108.73 |
| 9 | 113 | C03 | 1 µM BAM15 | 01:49:28 | 88.93 |
| 9 | 114 | C03 | 1 µM BAM15 | 01:49:42 | 80.15 |
| 9 | 115 | C03 | 1 µM BAM15 | 01:49:56 | 73.26 |
| 9 | 116 | C03 | 1 µM BAM15 | 01:50:10 | 67.34 |
| 9 | 117 | C03 | 1 µM BAM15 | 01:50:24 | 61.88 |
| 9 | 118 | C03 | 1 µM BAM15 | 01:50:38 | 56.96 |
| 9 | 119 | C03 | 1 µM BAM15 | 01:50:53 | 52.18 |
| 9 | 120 | C03 | 1 µM BAM15 | 01:51:07 | 47.88 |
| 9 | 121 | C03 | 1 µM BAM15 | 01:51:21 | 43.86 |
| 9 | 122 | C03 | 1 µM BAM15 | 01:51:35 | 40.15 |
| 9 | 123 | C03 | 1 µM BAM15 | 01:51:50 | 36.78 |
| 9 | 124 | C03 | 1 µM BAM15 | 01:52:04 | 33.89 |
| 9 | 125 | C03 | 1 µM BAM15 | 01:52:18 | 31.54 |
| 10 | 126 | C03 | 1 µM BAM15 | 01:57:52 | 109.32 |
| 10 | 127 | C03 | 1 µM BAM15 | 01:58:06 | 90.03 |
| 10 | 128 | C03 | 1 µM BAM15 | 01:58:20 | 81.22 |
| 10 | 129 | C03 | 1 µM BAM15 | 01:58:34 | 74.41 |
| 10 | 130 | C03 | 1 µM BAM15 | 01:58:48 | 68.44 |
| 10 | 131 | C03 | 1 µM BAM15 | 01:59:03 | 62.92 |
| 10 | 132 | C03 | 1 µM BAM15 | 01:59:17 | 57.93 |
| 10 | 133 | C03 | 1 µM BAM15 | 01:59:31 | 53.21 |
| 10 | 134 | C03 | 1 µM BAM15 | 01:59:45 | 48.98 |
| 10 | 135 | C03 | 1 µM BAM15 | 01:59:59 | 44.87 |
| 10 | 136 | C03 | 1 µM BAM15 | 02:00:14 | 41.09 |
| 10 | 137 | C03 | 1 µM BAM15 | 02:00:28 | 37.72 |
| 10 | 138 | C03 | 1 µM BAM15 | 02:00:42 | 34.71 |
| 10 | 139 | C03 | 1 µM BAM15 | 02:00:56 | 32.25 |
| 11 | 140 | C03 | 1 µM BAM15 | 02:06:30 | 111.49 |
| 11 | 141 | C03 | 1 µM BAM15 | 02:06:44 | 94.99 |
| 11 | 142 | C03 | 1 µM BAM15 | 02:06:58 | 86.47 |
| 11 | 143 | C03 | 1 µM BAM15 | 02:07:12 | 79.54 |
| 11 | 144 | C03 | 1 µM BAM15 | 02:07:26 | 73.53 |
| 11 | 145 | C03 | 1 µM BAM15 | 02:07:41 | 67.86 |
| 11 | 146 | C03 | 1 µM BAM15 | 02:07:55 | 62.76 |
| 11 | 147 | C03 | 1 µM BAM15 | 02:08:09 | 57.87 |
| 11 | 148 | C03 | 1 µM BAM15 | 02:08:23 | 53.38 |
| 11 | 149 | C03 | 1 µM BAM15 | 02:08:37 | 49.18 |
| 11 | 150 | C03 | 1 µM BAM15 | 02:08:52 | 45.16 |
| 11 | 151 | C03 | 1 µM BAM15 | 02:09:06 | 41.45 |
| 11 | 152 | C03 | 1 µM BAM15 | 02:09:20 | 38.12 |
| 11 | 153 | C03 | 1 µM BAM15 | 02:09:34 | 35.17 |
| 12 | 154 | C03 | 1 µM BAM15 | 02:15:08 | 112.90 |
| 12 | 155 | C03 | 1 µM BAM15 | 02:15:22 | 97.25 |
| 12 | 156 | C03 | 1 µM BAM15 | 02:15:37 | 88.38 |
| 12 | 157 | C03 | 1 µM BAM15 | 02:15:53 | 80.93 |
| 12 | 158 | C03 | 1 µM BAM15 | 02:16:08 | 74.71 |
| 12 | 159 | C03 | 1 µM BAM15 | 02:16:22 | 69.33 |
| 12 | 160 | C03 | 1 µM BAM15 | 02:16:36 | 64.23 |
| 12 | 161 | C03 | 1 µM BAM15 | 02:16:50 | 59.49 |
| 12 | 162 | C03 | 1 µM BAM15 | 02:17:04 | 55.04 |
| 12 | 163 | C03 | 1 µM BAM15 | 02:17:18 | 50.78 |
| 12 | 164 | C03 | 1 µM BAM15 | 02:17:33 | 46.81 |
| 12 | 165 | C03 | 1 µM BAM15 | 02:17:47 | 43.11 |
| 12 | 166 | C03 | 1 µM BAM15 | 02:18:01 | 39.66 |
| 13 | 167 | C03 | 1 µM BAM15 | 02:23:39 | 107.95 |
| 13 | 168 | C03 | 1 µM BAM15 | 02:23:54 | 95.36 |
| 13 | 169 | C03 | 1 µM BAM15 | 02:24:08 | 87.59 |
| 13 | 170 | C03 | 1 µM BAM15 | 02:24:22 | 81.11 |
| 13 | 171 | C03 | 1 µM BAM15 | 02:24:36 | 75.24 |
| 13 | 172 | C03 | 1 µM BAM15 | 02:24:51 | 69.52 |
| 13 | 173 | C03 | 1 µM BAM15 | 02:25:07 | 64.02 |
| 13 | 174 | C03 | 1 µM BAM15 | 02:25:22 | 59.24 |
| 13 | 175 | C03 | 1 µM BAM15 | 02:25:36 | 54.98 |
| 13 | 176 | C03 | 1 µM BAM15 | 02:25:50 | 50.81 |
| 13 | 177 | C03 | 1 µM BAM15 | 02:26:04 | 46.98 |
| 13 | 178 | C03 | 1 µM BAM15 | 02:26:18 | 43.28 |
| 13 | 179 | C03 | 1 µM BAM15 | 02:26:33 | 39.94 |
| 14 | 180 | C03 | 1 µM BAM15 | 02:32:07 | 113.37 |
| 14 | 181 | C03 | 1 µM BAM15 | 02:32:21 | 98.00 |
| 14 | 182 | C03 | 1 µM BAM15 | 02:32:35 | 89.93 |
| 14 | 183 | C03 | 1 µM BAM15 | 02:32:50 | 83.30 |
| 14 | 184 | C03 | 1 µM BAM15 | 02:33:04 | 77.47 |
| 14 | 185 | C03 | 1 µM BAM15 | 02:33:18 | 71.98 |
| 14 | 186 | C03 | 1 µM BAM15 | 02:33:32 | 67.05 |
| 14 | 187 | C03 | 1 µM BAM15 | 02:33:46 | 62.26 |
| 14 | 188 | C03 | 1 µM BAM15 | 02:34:01 | 57.94 |
| 14 | 189 | C03 | 1 µM BAM15 | 02:34:15 | 53.80 |
| 14 | 190 | C03 | 1 µM BAM15 | 02:34:29 | 49.80 |
| 14 | 191 | C03 | 1 µM BAM15 | 02:34:43 | 46.09 |
| 14 | 192 | C03 | 1 µM BAM15 | 02:34:57 | 42.49 |
| 14 | 193 | C03 | 1 µM BAM15 | 02:35:12 | 39.39 |
| 15 | 194 | C03 | 1 µM BAM15 | 02:40:46 | 113.64 |
| 15 | 195 | C03 | 1 µM BAM15 | 02:41:00 | 98.55 |
| 15 | 196 | C03 | 1 µM BAM15 | 02:41:14 | 90.49 |
| 15 | 197 | C03 | 1 µM BAM15 | 02:41:28 | 83.99 |
| 15 | 198 | C03 | 1 µM BAM15 | 02:41:43 | 78.18 |
| 15 | 199 | C03 | 1 µM BAM15 | 02:41:57 | 72.93 |
| 15 | 200 | C03 | 1 µM BAM15 | 02:42:11 | 68.00 |
| 15 | 201 | C03 | 1 µM BAM15 | 02:42:25 | 63.34 |
| 15 | 202 | C03 | 1 µM BAM15 | 02:42:39 | 59.01 |
| 15 | 203 | C03 | 1 µM BAM15 | 02:42:53 | 54.92 |
| 15 | 204 | C03 | 1 µM BAM15 | 02:43:08 | 50.91 |
| 15 | 205 | C03 | 1 µM BAM15 | 02:43:22 | 47.31 |
| 15 | 206 | C03 | 1 µM BAM15 | 02:43:36 | 43.70 |
| 15 | 207 | C03 | 1 µM BAM15 | 02:43:50 | 40.54 |
| 16 | 208 | C03 | 1 µM BAM15 | 02:49:25 | 113.83 |
| 16 | 209 | C03 | 1 µM BAM15 | 02:49:40 | 99.00 |
| 16 | 210 | C03 | 1 µM BAM15 | 02:49:54 | 91.07 |
| 16 | 211 | C03 | 1 µM BAM15 | 02:50:08 | 84.65 |
| 16 | 212 | C03 | 1 µM BAM15 | 02:50:22 | 78.99 |
| 16 | 213 | C03 | 1 µM BAM15 | 02:50:36 | 73.71 |
| 16 | 214 | C03 | 1 µM BAM15 | 02:50:50 | 68.91 |
| 16 | 215 | C03 | 1 µM BAM15 | 02:51:05 | 64.26 |
| 16 | 216 | C03 | 1 µM BAM15 | 02:51:19 | 59.96 |
| 16 | 217 | C03 | 1 µM BAM15 | 02:51:33 | 55.92 |
| 16 | 218 | C03 | 1 µM BAM15 | 02:51:47 | 52.09 |
| 16 | 219 | C03 | 1 µM BAM15 | 02:52:01 | 48.47 |
| 16 | 220 | C03 | 1 µM BAM15 | 02:52:16 | 44.88 |
| 16 | 221 | C03 | 1 µM BAM15 | 02:52:30 | 41.67 |
| 1 | 0 | C04 | 1 µM FCCP | 00:40:06 | 131.44 |
| 1 | 1 | C04 | 1 µM FCCP | 00:40:20 | 123.44 |
| 1 | 2 | C04 | 1 µM FCCP | 00:40:34 | 120.69 |
| 1 | 3 | C04 | 1 µM FCCP | 00:40:48 | 118.61 |
| 1 | 4 | C04 | 1 µM FCCP | 00:41:02 | 116.79 |
| 1 | 5 | C04 | 1 µM FCCP | 00:41:17 | 115.22 |
| 1 | 6 | C04 | 1 µM FCCP | 00:41:31 | 113.73 |
| 1 | 7 | C04 | 1 µM FCCP | 00:41:45 | 112.39 |
| 1 | 8 | C04 | 1 µM FCCP | 00:41:59 | 111.12 |
| 1 | 9 | C04 | 1 µM FCCP | 00:42:13 | 109.93 |
| 1 | 10 | C04 | 1 µM FCCP | 00:42:28 | 108.69 |
| 1 | 11 | C04 | 1 µM FCCP | 00:42:42 | 107.65 |
| 1 | 12 | C04 | 1 µM FCCP | 00:42:56 | 106.47 |
| 1 | 13 | C04 | 1 µM FCCP | 00:43:10 | 105.49 |
| 2 | 14 | C04 | 1 µM FCCP | 00:48:43 | 132.90 |
| 2 | 15 | C04 | 1 µM FCCP | 00:48:57 | 126.53 |
| 2 | 16 | C04 | 1 µM FCCP | 00:49:12 | 123.77 |
| 2 | 17 | C04 | 1 µM FCCP | 00:49:26 | 121.48 |
| 2 | 18 | C04 | 1 µM FCCP | 00:49:40 | 119.72 |
| 2 | 19 | C04 | 1 µM FCCP | 00:49:54 | 117.88 |
| 2 | 20 | C04 | 1 µM FCCP | 00:50:08 | 116.42 |
| 2 | 21 | C04 | 1 µM FCCP | 00:50:22 | 114.87 |
| 2 | 22 | C04 | 1 µM FCCP | 00:50:37 | 113.55 |
| 2 | 23 | C04 | 1 µM FCCP | 00:50:51 | 112.36 |
| 2 | 24 | C04 | 1 µM FCCP | 00:51:05 | 111.02 |
| 2 | 25 | C04 | 1 µM FCCP | 00:51:19 | 109.84 |
| 2 | 26 | C04 | 1 µM FCCP | 00:51:33 | 108.55 |
| 2 | 27 | C04 | 1 µM FCCP | 00:51:48 | 107.55 |
| 3 | 28 | C04 | 1 µM FCCP | 00:57:21 | 134.91 |
| 3 | 29 | C04 | 1 µM FCCP | 00:57:35 | 129.61 |
| 3 | 30 | C04 | 1 µM FCCP | 00:57:49 | 126.77 |
| 3 | 31 | C04 | 1 µM FCCP | 00:58:03 | 124.58 |
| 3 | 32 | C04 | 1 µM FCCP | 00:58:17 | 122.75 |
| 3 | 33 | C04 | 1 µM FCCP | 00:58:31 | 120.98 |
| 3 | 34 | C04 | 1 µM FCCP | 00:58:46 | 119.40 |
| 3 | 35 | C04 | 1 µM FCCP | 00:59:00 | 117.88 |
| 3 | 36 | C04 | 1 µM FCCP | 00:59:14 | 116.39 |
| 3 | 37 | C04 | 1 µM FCCP | 00:59:28 | 115.14 |
| 3 | 38 | C04 | 1 µM FCCP | 00:59:42 | 113.77 |
| 3 | 39 | C04 | 1 µM FCCP | 00:59:56 | 112.61 |
| 3 | 40 | C04 | 1 µM FCCP | 01:00:11 | 111.39 |
| 3 | 41 | C04 | 1 µM FCCP | 01:00:25 | 110.26 |
| 4 | 42 | C04 | 1 µM FCCP | 01:06:04 | 132.08 |
| 4 | 43 | C04 | 1 µM FCCP | 01:06:18 | 123.53 |
| 4 | 44 | C04 | 1 µM FCCP | 01:06:33 | 119.47 |
| 4 | 45 | C04 | 1 µM FCCP | 01:06:47 | 116.38 |
| 4 | 46 | C04 | 1 µM FCCP | 01:07:01 | 113.51 |
| 4 | 47 | C04 | 1 µM FCCP | 01:07:15 | 110.54 |
| 4 | 48 | C04 | 1 µM FCCP | 01:07:29 | 107.62 |
| 4 | 49 | C04 | 1 µM FCCP | 01:07:43 | 104.44 |
| 4 | 50 | C04 | 1 µM FCCP | 01:07:58 | 101.06 |
| 4 | 51 | C04 | 1 µM FCCP | 01:08:12 | 97.48 |
| 4 | 52 | C04 | 1 µM FCCP | 01:08:26 | 93.48 |
| 4 | 53 | C04 | 1 µM FCCP | 01:08:40 | 89.35 |
| 4 | 54 | C04 | 1 µM FCCP | 01:08:54 | 84.82 |
| 4 | 55 | C04 | 1 µM FCCP | 01:09:09 | 80.35 |
| 5 | 56 | C04 | 1 µM FCCP | 01:14:42 | 125.16 |
| 5 | 57 | C04 | 1 µM FCCP | 01:14:56 | 111.19 |
| 5 | 58 | C04 | 1 µM FCCP | 01:15:10 | 104.76 |
| 5 | 59 | C04 | 1 µM FCCP | 01:15:25 | 99.55 |
| 5 | 60 | C04 | 1 µM FCCP | 01:15:39 | 94.65 |
| 5 | 61 | C04 | 1 µM FCCP | 01:15:53 | 89.68 |
| 5 | 62 | C04 | 1 µM FCCP | 01:16:07 | 84.83 |
| 5 | 63 | C04 | 1 µM FCCP | 01:16:21 | 79.83 |
| 5 | 64 | C04 | 1 µM FCCP | 01:16:35 | 75.06 |
| 5 | 65 | C04 | 1 µM FCCP | 01:16:50 | 70.29 |
| 5 | 66 | C04 | 1 µM FCCP | 01:17:04 | 65.70 |
| 5 | 67 | C04 | 1 µM FCCP | 01:17:18 | 61.45 |
| 5 | 68 | C04 | 1 µM FCCP | 01:17:32 | 57.33 |
| 5 | 69 | C04 | 1 µM FCCP | 01:17:46 | 53.57 |
| 6 | 70 | C04 | 1 µM FCCP | 01:23:20 | 118.63 |
| 6 | 71 | C04 | 1 µM FCCP | 01:23:34 | 100.24 |
| 6 | 72 | C04 | 1 µM FCCP | 01:23:48 | 91.89 |
| 6 | 73 | C04 | 1 µM FCCP | 01:24:02 | 85.11 |
| 6 | 74 | C04 | 1 µM FCCP | 01:24:16 | 79.14 |
| 6 | 75 | C04 | 1 µM FCCP | 01:24:30 | 73.38 |
| 6 | 76 | C04 | 1 µM FCCP | 01:24:44 | 68.25 |
| 6 | 77 | C04 | 1 µM FCCP | 01:24:59 | 63.17 |
| 6 | 78 | C04 | 1 µM FCCP | 01:25:13 | 58.66 |
| 6 | 79 | C04 | 1 µM FCCP | 01:25:27 | 54.34 |
| 6 | 80 | C04 | 1 µM FCCP | 01:25:41 | 50.25 |
| 6 | 81 | C04 | 1 µM FCCP | 01:25:55 | 46.62 |
| 6 | 82 | C04 | 1 µM FCCP | 01:26:10 | 43.11 |
| 6 | 83 | C04 | 1 µM FCCP | 01:26:24 | 40.08 |
| 7 | 84 | C04 | 1 µM FCCP | 01:31:58 | 114.31 |
| 7 | 85 | C04 | 1 µM FCCP | 01:32:12 | 93.78 |
| 7 | 86 | C04 | 1 µM FCCP | 01:32:26 | 84.13 |
| 7 | 87 | C04 | 1 µM FCCP | 01:32:40 | 76.88 |
| 7 | 88 | C04 | 1 µM FCCP | 01:32:54 | 70.46 |
| 7 | 89 | C04 | 1 µM FCCP | 01:33:08 | 64.67 |
| 7 | 90 | C04 | 1 µM FCCP | 01:33:23 | 59.46 |
| 7 | 91 | C04 | 1 µM FCCP | 01:33:37 | 54.65 |
| 7 | 92 | C04 | 1 µM FCCP | 01:33:51 | 50.28 |
| 7 | 93 | C04 | 1 µM FCCP | 01:34:05 | 46.34 |
| 7 | 94 | C04 | 1 µM FCCP | 01:34:19 | 42.63 |
| 7 | 95 | C04 | 1 µM FCCP | 01:34:33 | 39.52 |
| 7 | 96 | C04 | 1 µM FCCP | 01:34:48 | 36.71 |
| 7 | 97 | C04 | 1 µM FCCP | 01:35:02 | 34.40 |
| 8 | 98 | C04 | 1 µM FCCP | 01:40:36 | 111.94 |
| 8 | 99 | C04 | 1 µM FCCP | 01:40:50 | 90.80 |
| 8 | 100 | C04 | 1 µM FCCP | 01:41:04 | 80.95 |
| 8 | 101 | C04 | 1 µM FCCP | 01:41:18 | 73.40 |
| 8 | 102 | C04 | 1 µM FCCP | 01:41:32 | 66.98 |
| 8 | 103 | C04 | 1 µM FCCP | 01:41:46 | 61.27 |
| 8 | 104 | C04 | 1 µM FCCP | 01:42:00 | 56.18 |
| 8 | 105 | C04 | 1 µM FCCP | 01:42:15 | 51.46 |
| 8 | 106 | C04 | 1 µM FCCP | 01:42:29 | 47.19 |
| 8 | 107 | C04 | 1 µM FCCP | 01:42:43 | 43.32 |
| 8 | 108 | C04 | 1 µM FCCP | 01:42:57 | 39.85 |
| 8 | 109 | C04 | 1 µM FCCP | 01:43:11 | 36.89 |
| 8 | 110 | C04 | 1 µM FCCP | 01:43:26 | 34.30 |
| 8 | 111 | C04 | 1 µM FCCP | 01:43:40 | 32.29 |
| 9 | 112 | C04 | 1 µM FCCP | 01:49:14 | 110.56 |
| 9 | 113 | C04 | 1 µM FCCP | 01:49:28 | 89.48 |
| 9 | 114 | C04 | 1 µM FCCP | 01:49:42 | 79.60 |
| 9 | 115 | C04 | 1 µM FCCP | 01:49:56 | 72.13 |
| 9 | 116 | C04 | 1 µM FCCP | 01:50:10 | 65.77 |
| 9 | 117 | C04 | 1 µM FCCP | 01:50:24 | 60.14 |
| 9 | 118 | C04 | 1 µM FCCP | 01:50:38 | 55.09 |
| 9 | 119 | C04 | 1 µM FCCP | 01:50:53 | 50.35 |
| 9 | 120 | C04 | 1 µM FCCP | 01:51:07 | 46.16 |
| 9 | 121 | C04 | 1 µM FCCP | 01:51:21 | 42.31 |
| 9 | 122 | C04 | 1 µM FCCP | 01:51:35 | 38.86 |
| 9 | 123 | C04 | 1 µM FCCP | 01:51:50 | 35.91 |
| 9 | 124 | C04 | 1 µM FCCP | 01:52:04 | 33.41 |
| 9 | 125 | C04 | 1 µM FCCP | 01:52:18 | 31.39 |
| 10 | 126 | C04 | 1 µM FCCP | 01:57:52 | 109.82 |
| 10 | 127 | C04 | 1 µM FCCP | 01:58:06 | 89.09 |
| 10 | 128 | C04 | 1 µM FCCP | 01:58:20 | 79.27 |
| 10 | 129 | C04 | 1 µM FCCP | 01:58:34 | 71.91 |
| 10 | 130 | C04 | 1 µM FCCP | 01:58:48 | 65.60 |
| 10 | 131 | C04 | 1 µM FCCP | 01:59:03 | 59.90 |
| 10 | 132 | C04 | 1 µM FCCP | 01:59:17 | 54.84 |
| 10 | 133 | C04 | 1 µM FCCP | 01:59:31 | 50.13 |
| 10 | 134 | C04 | 1 µM FCCP | 01:59:45 | 46.04 |
| 10 | 135 | C04 | 1 µM FCCP | 01:59:59 | 42.10 |
| 10 | 136 | C04 | 1 µM FCCP | 02:00:14 | 38.64 |
| 10 | 137 | C04 | 1 µM FCCP | 02:00:28 | 35.63 |
| 10 | 138 | C04 | 1 µM FCCP | 02:00:42 | 33.11 |
| 10 | 139 | C04 | 1 µM FCCP | 02:00:56 | 31.09 |
| 11 | 140 | C04 | 1 µM FCCP | 02:06:30 | 112.26 |
| 11 | 141 | C04 | 1 µM FCCP | 02:06:44 | 92.74 |
| 11 | 142 | C04 | 1 µM FCCP | 02:06:58 | 82.69 |
| 11 | 143 | C04 | 1 µM FCCP | 02:07:12 | 74.96 |
| 11 | 144 | C04 | 1 µM FCCP | 02:07:26 | 68.43 |
| 11 | 145 | C04 | 1 µM FCCP | 02:07:41 | 62.44 |
| 11 | 146 | C04 | 1 µM FCCP | 02:07:55 | 57.15 |
| 11 | 147 | C04 | 1 µM FCCP | 02:08:09 | 52.26 |
| 11 | 148 | C04 | 1 µM FCCP | 02:08:23 | 47.84 |
| 11 | 149 | C04 | 1 µM FCCP | 02:08:37 | 43.74 |
| 11 | 150 | C04 | 1 µM FCCP | 02:08:52 | 40.03 |
| 11 | 151 | C04 | 1 µM FCCP | 02:09:06 | 36.71 |
| 11 | 152 | C04 | 1 µM FCCP | 02:09:20 | 33.97 |
| 11 | 153 | C04 | 1 µM FCCP | 02:09:34 | 31.69 |
| 12 | 154 | C04 | 1 µM FCCP | 02:15:08 | 113.40 |
| 12 | 155 | C04 | 1 µM FCCP | 02:15:22 | 94.98 |
| 12 | 156 | C04 | 1 µM FCCP | 02:15:37 | 84.45 |
| 12 | 157 | C04 | 1 µM FCCP | 02:15:53 | 75.98 |
| 12 | 158 | C04 | 1 µM FCCP | 02:16:08 | 69.15 |
| 12 | 159 | C04 | 1 µM FCCP | 02:16:22 | 63.39 |
| 12 | 160 | C04 | 1 µM FCCP | 02:16:36 | 58.06 |
| 12 | 161 | C04 | 1 µM FCCP | 02:16:50 | 53.14 |
| 12 | 162 | C04 | 1 µM FCCP | 02:17:04 | 48.66 |
| 12 | 163 | C04 | 1 µM FCCP | 02:17:18 | 44.43 |
| 12 | 164 | C04 | 1 µM FCCP | 02:17:33 | 40.65 |
| 12 | 165 | C04 | 1 µM FCCP | 02:17:47 | 37.24 |
| 12 | 166 | C04 | 1 µM FCCP | 02:18:01 | 34.28 |
| 13 | 167 | C04 | 1 µM FCCP | 02:23:39 | 106.69 |
| 13 | 168 | C04 | 1 µM FCCP | 02:23:54 | 91.69 |
| 13 | 169 | C04 | 1 µM FCCP | 02:24:08 | 82.52 |
| 13 | 170 | C04 | 1 µM FCCP | 02:24:22 | 75.14 |
| 13 | 171 | C04 | 1 µM FCCP | 02:24:36 | 68.75 |
| 13 | 172 | C04 | 1 µM FCCP | 02:24:51 | 62.65 |
| 13 | 173 | C04 | 1 µM FCCP | 02:25:07 | 56.89 |
| 13 | 174 | C04 | 1 µM FCCP | 02:25:22 | 52.00 |
| 13 | 175 | C04 | 1 µM FCCP | 02:25:36 | 47.74 |
| 13 | 176 | C04 | 1 µM FCCP | 02:25:50 | 43.61 |
| 13 | 177 | C04 | 1 µM FCCP | 02:26:04 | 39.96 |
| 13 | 178 | C04 | 1 µM FCCP | 02:26:18 | 36.58 |
| 13 | 179 | C04 | 1 µM FCCP | 02:26:33 | 33.70 |
| 14 | 180 | C04 | 1 µM FCCP | 02:32:07 | 112.93 |
| 14 | 181 | C04 | 1 µM FCCP | 02:32:21 | 94.58 |
| 14 | 182 | C04 | 1 µM FCCP | 02:32:35 | 84.93 |
| 14 | 183 | C04 | 1 µM FCCP | 02:32:50 | 77.37 |
| 14 | 184 | C04 | 1 µM FCCP | 02:33:04 | 70.86 |
| 14 | 185 | C04 | 1 µM FCCP | 02:33:18 | 64.95 |
| 14 | 186 | C04 | 1 µM FCCP | 02:33:32 | 59.70 |
| 14 | 187 | C04 | 1 µM FCCP | 02:33:46 | 54.76 |
| 14 | 188 | C04 | 1 µM FCCP | 02:34:01 | 50.36 |
| 14 | 189 | C04 | 1 µM FCCP | 02:34:15 | 46.16 |
| 14 | 190 | C04 | 1 µM FCCP | 02:34:29 | 42.27 |
| 14 | 191 | C04 | 1 µM FCCP | 02:34:43 | 38.69 |
| 14 | 192 | C04 | 1 µM FCCP | 02:34:57 | 35.45 |
| 14 | 193 | C04 | 1 µM FCCP | 02:35:12 | 32.82 |
| 15 | 194 | C04 | 1 µM FCCP | 02:40:46 | 112.98 |
| 15 | 195 | C04 | 1 µM FCCP | 02:41:00 | 94.99 |
| 15 | 196 | C04 | 1 µM FCCP | 02:41:14 | 85.35 |
| 15 | 197 | C04 | 1 µM FCCP | 02:41:28 | 77.89 |
| 15 | 198 | C04 | 1 µM FCCP | 02:41:43 | 71.42 |
| 15 | 199 | C04 | 1 µM FCCP | 02:41:57 | 65.69 |
| 15 | 200 | C04 | 1 µM FCCP | 02:42:11 | 60.43 |
| 15 | 201 | C04 | 1 µM FCCP | 02:42:25 | 55.54 |
| 15 | 202 | C04 | 1 µM FCCP | 02:42:39 | 51.13 |
| 15 | 203 | C04 | 1 µM FCCP | 02:42:53 | 46.98 |
| 15 | 204 | C04 | 1 µM FCCP | 02:43:08 | 42.97 |
| 15 | 205 | C04 | 1 µM FCCP | 02:43:22 | 39.46 |
| 15 | 206 | C04 | 1 µM FCCP | 02:43:36 | 36.08 |
| 15 | 207 | C04 | 1 µM FCCP | 02:43:50 | 33.29 |
| 16 | 208 | C04 | 1 µM FCCP | 02:49:25 | 112.81 |
| 16 | 209 | C04 | 1 µM FCCP | 02:49:40 | 95.01 |
| 16 | 210 | C04 | 1 µM FCCP | 02:49:54 | 85.54 |
| 16 | 211 | C04 | 1 µM FCCP | 02:50:08 | 78.11 |
| 16 | 212 | C04 | 1 µM FCCP | 02:50:22 | 71.81 |
| 16 | 213 | C04 | 1 µM FCCP | 02:50:36 | 66.01 |
| 16 | 214 | C04 | 1 µM FCCP | 02:50:50 | 60.89 |
| 16 | 215 | C04 | 1 µM FCCP | 02:51:05 | 56.01 |
| 16 | 216 | C04 | 1 µM FCCP | 02:51:19 | 51.58 |
| 16 | 217 | C04 | 1 µM FCCP | 02:51:33 | 47.46 |
| 16 | 218 | C04 | 1 µM FCCP | 02:51:47 | 43.55 |
| 16 | 219 | C04 | 1 µM FCCP | 02:52:01 | 40.00 |
| 16 | 220 | C04 | 1 µM FCCP | 02:52:16 | 36.55 |
| 16 | 221 | C04 | 1 µM FCCP | 02:52:30 | 33.64 |
| 1 | 0 | C05 | 1 µM FCCP | 00:40:06 | 136.27 |
| 1 | 1 | C05 | 1 µM FCCP | 00:40:20 | 127.34 |
| 1 | 2 | C05 | 1 µM FCCP | 00:40:34 | 124.43 |
| 1 | 3 | C05 | 1 µM FCCP | 00:40:48 | 122.32 |
| 1 | 4 | C05 | 1 µM FCCP | 00:41:02 | 120.53 |
| 1 | 5 | C05 | 1 µM FCCP | 00:41:17 | 119.00 |
| 1 | 6 | C05 | 1 µM FCCP | 00:41:31 | 117.47 |
| 1 | 7 | C05 | 1 µM FCCP | 00:41:45 | 116.21 |
| 1 | 8 | C05 | 1 µM FCCP | 00:41:59 | 114.96 |
| 1 | 9 | C05 | 1 µM FCCP | 00:42:13 | 113.84 |
| 1 | 10 | C05 | 1 µM FCCP | 00:42:28 | 112.67 |
| 1 | 11 | C05 | 1 µM FCCP | 00:42:42 | 111.54 |
| 1 | 12 | C05 | 1 µM FCCP | 00:42:56 | 110.56 |
| 1 | 13 | C05 | 1 µM FCCP | 00:43:10 | 109.55 |
| 2 | 14 | C05 | 1 µM FCCP | 00:48:43 | 138.02 |
| 2 | 15 | C05 | 1 µM FCCP | 00:48:57 | 131.18 |
| 2 | 16 | C05 | 1 µM FCCP | 00:49:12 | 128.30 |
| 2 | 17 | C05 | 1 µM FCCP | 00:49:26 | 125.98 |
| 2 | 18 | C05 | 1 µM FCCP | 00:49:40 | 124.20 |
| 2 | 19 | C05 | 1 µM FCCP | 00:49:54 | 122.43 |
| 2 | 20 | C05 | 1 µM FCCP | 00:50:08 | 120.92 |
| 2 | 21 | C05 | 1 µM FCCP | 00:50:22 | 119.51 |
| 2 | 22 | C05 | 1 µM FCCP | 00:50:37 | 118.15 |
| 2 | 23 | C05 | 1 µM FCCP | 00:50:51 | 117.00 |
| 2 | 24 | C05 | 1 µM FCCP | 00:51:05 | 115.67 |
| 2 | 25 | C05 | 1 µM FCCP | 00:51:19 | 114.57 |
| 2 | 26 | C05 | 1 µM FCCP | 00:51:33 | 113.39 |
| 2 | 27 | C05 | 1 µM FCCP | 00:51:48 | 112.33 |
| 3 | 28 | C05 | 1 µM FCCP | 00:57:21 | 138.84 |
| 3 | 29 | C05 | 1 µM FCCP | 00:57:35 | 132.75 |
| 3 | 30 | C05 | 1 µM FCCP | 00:57:49 | 129.96 |
| 3 | 31 | C05 | 1 µM FCCP | 00:58:03 | 127.77 |
| 3 | 32 | C05 | 1 µM FCCP | 00:58:17 | 125.93 |
| 3 | 33 | C05 | 1 µM FCCP | 00:58:31 | 124.28 |
| 3 | 34 | C05 | 1 µM FCCP | 00:58:46 | 122.67 |
| 3 | 35 | C05 | 1 µM FCCP | 00:59:00 | 121.35 |
| 3 | 36 | C05 | 1 µM FCCP | 00:59:14 | 119.89 |
| 3 | 37 | C05 | 1 µM FCCP | 00:59:28 | 118.69 |
| 3 | 38 | C05 | 1 µM FCCP | 00:59:42 | 117.39 |
| 3 | 39 | C05 | 1 µM FCCP | 00:59:56 | 116.29 |
| 3 | 40 | C05 | 1 µM FCCP | 01:00:11 | 115.24 |
| 3 | 41 | C05 | 1 µM FCCP | 01:00:25 | 114.07 |
| 4 | 42 | C05 | 1 µM FCCP | 01:06:04 | 136.88 |
| 4 | 43 | C05 | 1 µM FCCP | 01:06:18 | 128.20 |
| 4 | 44 | C05 | 1 µM FCCP | 01:06:33 | 124.55 |
| 4 | 45 | C05 | 1 µM FCCP | 01:06:47 | 121.66 |
| 4 | 46 | C05 | 1 µM FCCP | 01:07:01 | 119.06 |
| 4 | 47 | C05 | 1 µM FCCP | 01:07:15 | 116.56 |
| 4 | 48 | C05 | 1 µM FCCP | 01:07:29 | 113.94 |
| 4 | 49 | C05 | 1 µM FCCP | 01:07:43 | 111.24 |
| 4 | 50 | C05 | 1 µM FCCP | 01:07:58 | 108.41 |
| 4 | 51 | C05 | 1 µM FCCP | 01:08:12 | 105.25 |
| 4 | 52 | C05 | 1 µM FCCP | 01:08:26 | 101.94 |
| 4 | 53 | C05 | 1 µM FCCP | 01:08:40 | 98.33 |
| 4 | 54 | C05 | 1 µM FCCP | 01:08:54 | 94.46 |
| 4 | 55 | C05 | 1 µM FCCP | 01:09:09 | 90.58 |
| 5 | 56 | C05 | 1 µM FCCP | 01:14:42 | 131.16 |
| 5 | 57 | C05 | 1 µM FCCP | 01:14:56 | 118.23 |
| 5 | 58 | C05 | 1 µM FCCP | 01:15:10 | 112.60 |
| 5 | 59 | C05 | 1 µM FCCP | 01:15:25 | 108.06 |
| 5 | 60 | C05 | 1 µM FCCP | 01:15:39 | 103.85 |
| 5 | 61 | C05 | 1 µM FCCP | 01:15:53 | 99.61 |
| 5 | 62 | C05 | 1 µM FCCP | 01:16:07 | 95.39 |
| 5 | 63 | C05 | 1 µM FCCP | 01:16:21 | 91.13 |
| 5 | 64 | C05 | 1 µM FCCP | 01:16:35 | 86.90 |
| 5 | 65 | C05 | 1 µM FCCP | 01:16:50 | 82.75 |
| 5 | 66 | C05 | 1 µM FCCP | 01:17:04 | 78.67 |
| 5 | 67 | C05 | 1 µM FCCP | 01:17:18 | 74.87 |
| 5 | 68 | C05 | 1 µM FCCP | 01:17:32 | 71.17 |
| 5 | 69 | C05 | 1 µM FCCP | 01:17:46 | 67.78 |
| 6 | 70 | C05 | 1 µM FCCP | 01:23:20 | 125.86 |
| 6 | 71 | C05 | 1 µM FCCP | 01:23:34 | 109.57 |
| 6 | 72 | C05 | 1 µM FCCP | 01:23:48 | 102.67 |
| 6 | 73 | C05 | 1 µM FCCP | 01:24:02 | 96.90 |
| 6 | 74 | C05 | 1 µM FCCP | 01:24:16 | 91.78 |
| 6 | 75 | C05 | 1 µM FCCP | 01:24:30 | 86.82 |
| 6 | 76 | C05 | 1 µM FCCP | 01:24:44 | 82.14 |
| 6 | 77 | C05 | 1 µM FCCP | 01:24:59 | 77.61 |
| 6 | 78 | C05 | 1 µM FCCP | 01:25:13 | 73.43 |
| 6 | 79 | C05 | 1 µM FCCP | 01:25:27 | 69.46 |
| 6 | 80 | C05 | 1 µM FCCP | 01:25:41 | 65.66 |
| 6 | 81 | C05 | 1 µM FCCP | 01:25:55 | 62.26 |
| 6 | 82 | C05 | 1 µM FCCP | 01:26:10 | 58.91 |
| 6 | 83 | C05 | 1 µM FCCP | 01:26:24 | 55.82 |
| 7 | 84 | C05 | 1 µM FCCP | 01:31:58 | 121.52 |
| 7 | 85 | C05 | 1 µM FCCP | 01:32:12 | 103.24 |
| 7 | 86 | C05 | 1 µM FCCP | 01:32:26 | 95.03 |
| 7 | 87 | C05 | 1 µM FCCP | 01:32:40 | 88.68 |
| 7 | 88 | C05 | 1 µM FCCP | 01:32:54 | 83.01 |
| 7 | 89 | C05 | 1 µM FCCP | 01:33:08 | 77.83 |
| 7 | 90 | C05 | 1 µM FCCP | 01:33:23 | 73.10 |
| 7 | 91 | C05 | 1 µM FCCP | 01:33:37 | 68.64 |
| 7 | 92 | C05 | 1 µM FCCP | 01:33:51 | 64.60 |
| 7 | 93 | C05 | 1 µM FCCP | 01:34:05 | 60.81 |
| 7 | 94 | C05 | 1 µM FCCP | 01:34:19 | 57.25 |
| 7 | 95 | C05 | 1 µM FCCP | 01:34:33 | 53.99 |
| 7 | 96 | C05 | 1 µM FCCP | 01:34:48 | 50.87 |
| 7 | 97 | C05 | 1 µM FCCP | 01:35:02 | 47.95 |
| 8 | 98 | C05 | 1 µM FCCP | 01:40:36 | 118.75 |
| 8 | 99 | C05 | 1 µM FCCP | 01:40:50 | 99.21 |
| 8 | 100 | C05 | 1 µM FCCP | 01:41:04 | 90.35 |
| 8 | 101 | C05 | 1 µM FCCP | 01:41:18 | 83.56 |
| 8 | 102 | C05 | 1 µM FCCP | 01:41:32 | 77.72 |
| 8 | 103 | C05 | 1 µM FCCP | 01:41:46 | 72.46 |
| 8 | 104 | C05 | 1 µM FCCP | 01:42:00 | 67.71 |
| 8 | 105 | C05 | 1 µM FCCP | 01:42:15 | 63.35 |
| 8 | 106 | C05 | 1 µM FCCP | 01:42:29 | 59.30 |
| 8 | 107 | C05 | 1 µM FCCP | 01:42:43 | 55.55 |
| 8 | 108 | C05 | 1 µM FCCP | 01:42:57 | 52.05 |
| 8 | 109 | C05 | 1 µM FCCP | 01:43:11 | 48.83 |
| 8 | 110 | C05 | 1 µM FCCP | 01:43:26 | 45.71 |
| 8 | 111 | C05 | 1 µM FCCP | 01:43:40 | 42.90 |
| 9 | 112 | C05 | 1 µM FCCP | 01:49:14 | 116.90 |
| 9 | 113 | C05 | 1 µM FCCP | 01:49:28 | 96.83 |
| 9 | 114 | C05 | 1 µM FCCP | 01:49:42 | 87.63 |
| 9 | 115 | C05 | 1 µM FCCP | 01:49:56 | 80.68 |
| 9 | 116 | C05 | 1 µM FCCP | 01:50:10 | 74.73 |
| 9 | 117 | C05 | 1 µM FCCP | 01:50:24 | 69.48 |
| 9 | 118 | C05 | 1 µM FCCP | 01:50:38 | 64.73 |
| 9 | 119 | C05 | 1 µM FCCP | 01:50:53 | 60.30 |
| 9 | 120 | C05 | 1 µM FCCP | 01:51:07 | 56.27 |
| 9 | 121 | C05 | 1 µM FCCP | 01:51:21 | 52.51 |
| 9 | 122 | C05 | 1 µM FCCP | 01:51:35 | 49.07 |
| 9 | 123 | C05 | 1 µM FCCP | 01:51:50 | 45.79 |
| 9 | 124 | C05 | 1 µM FCCP | 01:52:04 | 42.72 |
| 9 | 125 | C05 | 1 µM FCCP | 01:52:18 | 39.88 |
| 10 | 126 | C05 | 1 µM FCCP | 01:57:52 | 115.68 |
| 10 | 127 | C05 | 1 µM FCCP | 01:58:06 | 95.46 |
| 10 | 128 | C05 | 1 µM FCCP | 01:58:20 | 86.17 |
| 10 | 129 | C05 | 1 µM FCCP | 01:58:34 | 79.18 |
| 10 | 130 | C05 | 1 µM FCCP | 01:58:48 | 73.29 |
| 10 | 131 | C05 | 1 µM FCCP | 01:59:03 | 67.89 |
| 10 | 132 | C05 | 1 µM FCCP | 01:59:17 | 63.12 |
| 10 | 133 | C05 | 1 µM FCCP | 01:59:31 | 58.67 |
| 10 | 134 | C05 | 1 µM FCCP | 01:59:45 | 54.75 |
| 10 | 135 | C05 | 1 µM FCCP | 01:59:59 | 50.90 |
| 10 | 136 | C05 | 1 µM FCCP | 02:00:14 | 47.39 |
| 10 | 137 | C05 | 1 µM FCCP | 02:00:28 | 44.17 |
| 10 | 138 | C05 | 1 µM FCCP | 02:00:42 | 41.04 |
| 10 | 139 | C05 | 1 µM FCCP | 02:00:56 | 38.25 |
| 11 | 140 | C05 | 1 µM FCCP | 02:06:30 | 119.03 |
| 11 | 141 | C05 | 1 µM FCCP | 02:06:44 | 100.60 |
| 11 | 142 | C05 | 1 µM FCCP | 02:06:58 | 91.15 |
| 11 | 143 | C05 | 1 µM FCCP | 02:07:12 | 83.82 |
| 11 | 144 | C05 | 1 µM FCCP | 02:07:26 | 77.52 |
| 11 | 145 | C05 | 1 µM FCCP | 02:07:41 | 71.82 |
| 11 | 146 | C05 | 1 µM FCCP | 02:07:55 | 66.72 |
| 11 | 147 | C05 | 1 µM FCCP | 02:08:09 | 62.01 |
| 11 | 148 | C05 | 1 µM FCCP | 02:08:23 | 57.71 |
| 11 | 149 | C05 | 1 µM FCCP | 02:08:37 | 53.72 |
| 11 | 150 | C05 | 1 µM FCCP | 02:08:52 | 49.94 |
| 11 | 151 | C05 | 1 µM FCCP | 02:09:06 | 46.39 |
| 11 | 152 | C05 | 1 µM FCCP | 02:09:20 | 43.16 |
| 11 | 153 | C05 | 1 µM FCCP | 02:09:34 | 40.09 |
| 12 | 154 | C05 | 1 µM FCCP | 02:15:08 | 118.78 |
| 12 | 155 | C05 | 1 µM FCCP | 02:15:22 | 101.62 |
| 12 | 156 | C05 | 1 µM FCCP | 02:15:37 | 91.81 |
| 12 | 157 | C05 | 1 µM FCCP | 02:15:53 | 83.99 |
| 12 | 158 | C05 | 1 µM FCCP | 02:16:08 | 77.56 |
| 12 | 159 | C05 | 1 µM FCCP | 02:16:22 | 72.17 |
| 12 | 160 | C05 | 1 µM FCCP | 02:16:36 | 67.12 |
| 12 | 161 | C05 | 1 µM FCCP | 02:16:50 | 62.50 |
| 12 | 162 | C05 | 1 µM FCCP | 02:17:04 | 58.14 |
| 12 | 163 | C05 | 1 µM FCCP | 02:17:18 | 54.10 |
| 12 | 164 | C05 | 1 µM FCCP | 02:17:33 | 50.32 |
| 12 | 165 | C05 | 1 µM FCCP | 02:17:47 | 46.78 |
| 12 | 166 | C05 | 1 µM FCCP | 02:18:01 | 43.42 |
| 13 | 167 | C05 | 1 µM FCCP | 02:23:39 | 113.46 |
| 13 | 168 | C05 | 1 µM FCCP | 02:23:54 | 99.39 |
| 13 | 169 | C05 | 1 µM FCCP | 02:24:08 | 90.84 |
| 13 | 170 | C05 | 1 µM FCCP | 02:24:22 | 83.97 |
| 13 | 171 | C05 | 1 µM FCCP | 02:24:36 | 77.92 |
| 13 | 172 | C05 | 1 µM FCCP | 02:24:51 | 72.08 |
| 13 | 173 | C05 | 1 µM FCCP | 02:25:07 | 66.65 |
| 13 | 174 | C05 | 1 µM FCCP | 02:25:22 | 61.99 |
| 13 | 175 | C05 | 1 µM FCCP | 02:25:36 | 57.91 |
| 13 | 176 | C05 | 1 µM FCCP | 02:25:50 | 53.94 |
| 13 | 177 | C05 | 1 µM FCCP | 02:26:04 | 50.30 |
| 13 | 178 | C05 | 1 µM FCCP | 02:26:18 | 46.76 |
| 13 | 179 | C05 | 1 µM FCCP | 02:26:33 | 43.44 |
| 14 | 180 | C05 | 1 µM FCCP | 02:32:07 | 118.56 |
| 14 | 181 | C05 | 1 µM FCCP | 02:32:21 | 101.18 |
| 14 | 182 | C05 | 1 µM FCCP | 02:32:35 | 92.18 |
| 14 | 183 | C05 | 1 µM FCCP | 02:32:50 | 84.95 |
| 14 | 184 | C05 | 1 µM FCCP | 02:33:04 | 78.88 |
| 14 | 185 | C05 | 1 µM FCCP | 02:33:18 | 73.33 |
| 14 | 186 | C05 | 1 µM FCCP | 02:33:32 | 68.37 |
| 14 | 187 | C05 | 1 µM FCCP | 02:33:46 | 63.69 |
| 14 | 188 | C05 | 1 µM FCCP | 02:34:01 | 59.45 |
| 14 | 189 | C05 | 1 µM FCCP | 02:34:15 | 55.51 |
| 14 | 190 | C05 | 1 µM FCCP | 02:34:29 | 51.73 |
| 14 | 191 | C05 | 1 µM FCCP | 02:34:43 | 48.15 |
| 14 | 192 | C05 | 1 µM FCCP | 02:34:57 | 44.75 |
| 14 | 193 | C05 | 1 µM FCCP | 02:35:12 | 41.65 |
| 15 | 194 | C05 | 1 µM FCCP | 02:40:46 | 118.03 |
| 15 | 195 | C05 | 1 µM FCCP | 02:41:00 | 100.89 |
| 15 | 196 | C05 | 1 µM FCCP | 02:41:14 | 91.74 |
| 15 | 197 | C05 | 1 µM FCCP | 02:41:28 | 84.76 |
| 15 | 198 | C05 | 1 µM FCCP | 02:41:43 | 78.64 |
| 15 | 199 | C05 | 1 µM FCCP | 02:41:57 | 73.25 |
| 15 | 200 | C05 | 1 µM FCCP | 02:42:11 | 68.30 |
| 15 | 201 | C05 | 1 µM FCCP | 02:42:25 | 63.72 |
| 15 | 202 | C05 | 1 µM FCCP | 02:42:39 | 59.49 |
| 15 | 203 | C05 | 1 µM FCCP | 02:42:53 | 55.54 |
| 15 | 204 | C05 | 1 µM FCCP | 02:43:08 | 51.71 |
| 15 | 205 | C05 | 1 µM FCCP | 02:43:22 | 48.28 |
| 15 | 206 | C05 | 1 µM FCCP | 02:43:36 | 44.81 |
| 15 | 207 | C05 | 1 µM FCCP | 02:43:50 | 41.69 |
| 16 | 208 | C05 | 1 µM FCCP | 02:49:25 | 117.80 |
| 16 | 209 | C05 | 1 µM FCCP | 02:49:40 | 100.87 |
| 16 | 210 | C05 | 1 µM FCCP | 02:49:54 | 91.94 |
| 16 | 211 | C05 | 1 µM FCCP | 02:50:08 | 84.94 |
| 16 | 212 | C05 | 1 µM FCCP | 02:50:22 | 78.90 |
| 16 | 213 | C05 | 1 µM FCCP | 02:50:36 | 73.43 |
| 16 | 214 | C05 | 1 µM FCCP | 02:50:50 | 68.56 |
| 16 | 215 | C05 | 1 µM FCCP | 02:51:05 | 63.96 |
| 16 | 216 | C05 | 1 µM FCCP | 02:51:19 | 59.76 |
| 16 | 217 | C05 | 1 µM FCCP | 02:51:33 | 55.85 |
| 16 | 218 | C05 | 1 µM FCCP | 02:51:47 | 52.12 |
| 16 | 219 | C05 | 1 µM FCCP | 02:52:01 | 48.63 |
| 16 | 220 | C05 | 1 µM FCCP | 02:52:16 | 45.19 |
| 16 | 221 | C05 | 1 µM FCCP | 02:52:30 | 42.03 |
| 1 | 0 | C06 | 1 µM FCCP | 00:40:06 | 127.76 |
| 1 | 1 | C06 | 1 µM FCCP | 00:40:20 | 118.56 |
| 1 | 2 | C06 | 1 µM FCCP | 00:40:34 | 115.45 |
| 1 | 3 | C06 | 1 µM FCCP | 00:40:48 | 113.26 |
| 1 | 4 | C06 | 1 µM FCCP | 00:41:02 | 111.37 |
| 1 | 5 | C06 | 1 µM FCCP | 00:41:17 | 109.63 |
| 1 | 6 | C06 | 1 µM FCCP | 00:41:31 | 108.10 |
| 1 | 7 | C06 | 1 µM FCCP | 00:41:45 | 106.64 |
| 1 | 8 | C06 | 1 µM FCCP | 00:41:59 | 105.27 |
| 1 | 9 | C06 | 1 µM FCCP | 00:42:13 | 104.00 |
| 1 | 10 | C06 | 1 µM FCCP | 00:42:28 | 102.72 |
| 1 | 11 | C06 | 1 µM FCCP | 00:42:42 | 101.49 |
| 1 | 12 | C06 | 1 µM FCCP | 00:42:56 | 100.33 |
| 1 | 13 | C06 | 1 µM FCCP | 00:43:10 | 99.24 |
| 2 | 14 | C06 | 1 µM FCCP | 00:48:43 | 132.22 |
| 2 | 15 | C06 | 1 µM FCCP | 00:48:57 | 125.57 |
| 2 | 16 | C06 | 1 µM FCCP | 00:49:12 | 122.49 |
| 2 | 17 | C06 | 1 µM FCCP | 00:49:26 | 120.05 |
| 2 | 18 | C06 | 1 µM FCCP | 00:49:40 | 118.07 |
| 2 | 19 | C06 | 1 µM FCCP | 00:49:54 | 116.12 |
| 2 | 20 | C06 | 1 µM FCCP | 00:50:08 | 114.48 |
| 2 | 21 | C06 | 1 µM FCCP | 00:50:22 | 112.81 |
| 2 | 22 | C06 | 1 µM FCCP | 00:50:37 | 111.40 |
| 2 | 23 | C06 | 1 µM FCCP | 00:50:51 | 109.96 |
| 2 | 24 | C06 | 1 µM FCCP | 00:51:05 | 108.54 |
| 2 | 25 | C06 | 1 µM FCCP | 00:51:19 | 107.26 |
| 2 | 26 | C06 | 1 µM FCCP | 00:51:33 | 106.01 |
| 2 | 27 | C06 | 1 µM FCCP | 00:51:48 | 104.75 |
| 3 | 28 | C06 | 1 µM FCCP | 00:57:21 | 133.45 |
| 3 | 29 | C06 | 1 µM FCCP | 00:57:35 | 127.31 |
| 3 | 30 | C06 | 1 µM FCCP | 00:57:49 | 124.30 |
| 3 | 31 | C06 | 1 µM FCCP | 00:58:03 | 121.97 |
| 3 | 32 | C06 | 1 µM FCCP | 00:58:17 | 119.97 |
| 3 | 33 | C06 | 1 µM FCCP | 00:58:31 | 118.07 |
| 3 | 34 | C06 | 1 µM FCCP | 00:58:46 | 116.41 |
| 3 | 35 | C06 | 1 µM FCCP | 00:59:00 | 114.75 |
| 3 | 36 | C06 | 1 µM FCCP | 00:59:14 | 113.16 |
| 3 | 37 | C06 | 1 µM FCCP | 00:59:28 | 111.82 |
| 3 | 38 | C06 | 1 µM FCCP | 00:59:42 | 110.42 |
| 3 | 39 | C06 | 1 µM FCCP | 00:59:56 | 109.16 |
| 3 | 40 | C06 | 1 µM FCCP | 01:00:11 | 107.92 |
| 3 | 41 | C06 | 1 µM FCCP | 01:00:25 | 106.70 |
| 4 | 42 | C06 | 1 µM FCCP | 01:06:04 | 130.43 |
| 4 | 43 | C06 | 1 µM FCCP | 01:06:18 | 121.41 |
| 4 | 44 | C06 | 1 µM FCCP | 01:06:33 | 117.39 |
| 4 | 45 | C06 | 1 µM FCCP | 01:06:47 | 114.22 |
| 4 | 46 | C06 | 1 µM FCCP | 01:07:01 | 111.40 |
| 4 | 47 | C06 | 1 µM FCCP | 01:07:15 | 108.39 |
| 4 | 48 | C06 | 1 µM FCCP | 01:07:29 | 105.41 |
| 4 | 49 | C06 | 1 µM FCCP | 01:07:43 | 102.25 |
| 4 | 50 | C06 | 1 µM FCCP | 01:07:58 | 98.98 |
| 4 | 51 | C06 | 1 µM FCCP | 01:08:12 | 95.39 |
| 4 | 52 | C06 | 1 µM FCCP | 01:08:26 | 91.49 |
| 4 | 53 | C06 | 1 µM FCCP | 01:08:40 | 87.37 |
| 4 | 54 | C06 | 1 µM FCCP | 01:08:54 | 82.88 |
| 4 | 55 | C06 | 1 µM FCCP | 01:09:09 | 78.37 |
| 5 | 56 | C06 | 1 µM FCCP | 01:14:42 | 123.76 |
| 5 | 57 | C06 | 1 µM FCCP | 01:14:56 | 109.50 |
| 5 | 58 | C06 | 1 µM FCCP | 01:15:10 | 103.14 |
| 5 | 59 | C06 | 1 µM FCCP | 01:15:25 | 98.14 |
| 5 | 60 | C06 | 1 µM FCCP | 01:15:39 | 93.38 |
| 5 | 61 | C06 | 1 µM FCCP | 01:15:53 | 88.52 |
| 5 | 62 | C06 | 1 µM FCCP | 01:16:07 | 83.66 |
| 5 | 63 | C06 | 1 µM FCCP | 01:16:21 | 78.76 |
| 5 | 64 | C06 | 1 µM FCCP | 01:16:35 | 73.91 |
| 5 | 65 | C06 | 1 µM FCCP | 01:16:50 | 69.02 |
| 5 | 66 | C06 | 1 µM FCCP | 01:17:04 | 64.17 |
| 5 | 67 | C06 | 1 µM FCCP | 01:17:18 | 59.61 |
| 5 | 68 | C06 | 1 µM FCCP | 01:17:32 | 55.24 |
| 5 | 69 | C06 | 1 µM FCCP | 01:17:46 | 51.15 |
| 6 | 70 | C06 | 1 µM FCCP | 01:23:20 | 117.60 |
| 6 | 71 | C06 | 1 µM FCCP | 01:23:34 | 99.24 |
| 6 | 72 | C06 | 1 µM FCCP | 01:23:48 | 91.20 |
| 6 | 73 | C06 | 1 µM FCCP | 01:24:02 | 84.74 |
| 6 | 74 | C06 | 1 µM FCCP | 01:24:16 | 78.86 |
| 6 | 75 | C06 | 1 µM FCCP | 01:24:30 | 73.16 |
| 6 | 76 | C06 | 1 µM FCCP | 01:24:44 | 67.76 |
| 6 | 77 | C06 | 1 µM FCCP | 01:24:59 | 62.50 |
| 6 | 78 | C06 | 1 µM FCCP | 01:25:13 | 57.62 |
| 6 | 79 | C06 | 1 µM FCCP | 01:25:27 | 52.97 |
| 6 | 80 | C06 | 1 µM FCCP | 01:25:41 | 48.53 |
| 6 | 81 | C06 | 1 µM FCCP | 01:25:55 | 44.54 |
| 6 | 82 | C06 | 1 µM FCCP | 01:26:10 | 40.75 |
| 6 | 83 | C06 | 1 µM FCCP | 01:26:24 | 37.54 |
| 7 | 84 | C06 | 1 µM FCCP | 01:31:58 | 113.10 |
| 7 | 85 | C06 | 1 µM FCCP | 01:32:12 | 92.37 |
| 7 | 86 | C06 | 1 µM FCCP | 01:32:26 | 82.93 |
| 7 | 87 | C06 | 1 µM FCCP | 01:32:40 | 75.71 |
| 7 | 88 | C06 | 1 µM FCCP | 01:32:54 | 69.21 |
| 7 | 89 | C06 | 1 µM FCCP | 01:33:08 | 63.25 |
| 7 | 90 | C06 | 1 µM FCCP | 01:33:23 | 57.74 |
| 7 | 91 | C06 | 1 µM FCCP | 01:33:37 | 52.60 |
| 7 | 92 | C06 | 1 µM FCCP | 01:33:51 | 47.94 |
| 7 | 93 | C06 | 1 µM FCCP | 01:34:05 | 43.68 |
| 7 | 94 | C06 | 1 µM FCCP | 01:34:19 | 39.75 |
| 7 | 95 | C06 | 1 µM FCCP | 01:34:33 | 36.51 |
| 7 | 96 | C06 | 1 µM FCCP | 01:34:48 | 33.70 |
| 7 | 97 | C06 | 1 µM FCCP | 01:35:02 | 31.44 |
| 8 | 98 | C06 | 1 µM FCCP | 01:40:36 | 110.81 |
| 8 | 99 | C06 | 1 µM FCCP | 01:40:50 | 89.05 |
| 8 | 100 | C06 | 1 µM FCCP | 01:41:04 | 79.07 |
| 8 | 101 | C06 | 1 µM FCCP | 01:41:18 | 71.34 |
| 8 | 102 | C06 | 1 µM FCCP | 01:41:32 | 64.73 |
| 8 | 103 | C06 | 1 µM FCCP | 01:41:46 | 58.70 |
| 8 | 104 | C06 | 1 µM FCCP | 01:42:00 | 53.31 |
| 8 | 105 | C06 | 1 µM FCCP | 01:42:15 | 48.29 |
| 8 | 106 | C06 | 1 µM FCCP | 01:42:29 | 43.74 |
| 8 | 107 | C06 | 1 µM FCCP | 01:42:43 | 39.72 |
| 8 | 108 | C06 | 1 µM FCCP | 01:42:57 | 36.20 |
| 8 | 109 | C06 | 1 µM FCCP | 01:43:11 | 33.33 |
| 8 | 110 | C06 | 1 µM FCCP | 01:43:26 | 30.89 |
| 8 | 111 | C06 | 1 µM FCCP | 01:43:40 | 28.99 |
| 9 | 112 | C06 | 1 µM FCCP | 01:49:14 | 109.47 |
| 9 | 113 | C06 | 1 µM FCCP | 01:49:28 | 87.39 |
| 9 | 114 | C06 | 1 µM FCCP | 01:49:42 | 77.26 |
| 9 | 115 | C06 | 1 µM FCCP | 01:49:56 | 69.48 |
| 9 | 116 | C06 | 1 µM FCCP | 01:50:10 | 62.81 |
| 9 | 117 | C06 | 1 µM FCCP | 01:50:24 | 56.82 |
| 9 | 118 | C06 | 1 µM FCCP | 01:50:38 | 51.45 |
| 9 | 119 | C06 | 1 µM FCCP | 01:50:53 | 46.44 |
| 9 | 120 | C06 | 1 µM FCCP | 01:51:07 | 41.98 |
| 9 | 121 | C06 | 1 µM FCCP | 01:51:21 | 38.01 |
| 9 | 122 | C06 | 1 µM FCCP | 01:51:35 | 34.66 |
| 9 | 123 | C06 | 1 µM FCCP | 01:51:50 | 31.91 |
| 9 | 124 | C06 | 1 µM FCCP | 01:52:04 | 29.66 |
| 9 | 125 | C06 | 1 µM FCCP | 01:52:18 | 27.85 |
| 10 | 126 | C06 | 1 µM FCCP | 01:57:52 | 108.73 |
| 10 | 127 | C06 | 1 µM FCCP | 01:58:06 | 86.75 |
| 10 | 128 | C06 | 1 µM FCCP | 01:58:20 | 76.55 |
| 10 | 129 | C06 | 1 µM FCCP | 01:58:34 | 68.85 |
| 10 | 130 | C06 | 1 µM FCCP | 01:58:48 | 62.21 |
| 10 | 131 | C06 | 1 µM FCCP | 01:59:03 | 56.13 |
| 10 | 132 | C06 | 1 µM FCCP | 01:59:17 | 50.78 |
| 10 | 133 | C06 | 1 µM FCCP | 01:59:31 | 45.77 |
| 10 | 134 | C06 | 1 µM FCCP | 01:59:45 | 41.43 |
| 10 | 135 | C06 | 1 µM FCCP | 01:59:59 | 37.42 |
| 10 | 136 | C06 | 1 µM FCCP | 02:00:14 | 34.03 |
| 10 | 137 | C06 | 1 µM FCCP | 02:00:28 | 31.34 |
| 10 | 138 | C06 | 1 µM FCCP | 02:00:42 | 29.11 |
| 10 | 139 | C06 | 1 µM FCCP | 02:00:56 | 27.37 |
| 11 | 140 | C06 | 1 µM FCCP | 02:06:30 | 109.53 |
| 11 | 141 | C06 | 1 µM FCCP | 02:06:44 | 88.33 |
| 11 | 142 | C06 | 1 µM FCCP | 02:06:58 | 78.13 |
| 11 | 143 | C06 | 1 µM FCCP | 02:07:12 | 70.18 |
| 11 | 144 | C06 | 1 µM FCCP | 02:07:26 | 63.44 |
| 11 | 145 | C06 | 1 µM FCCP | 02:07:41 | 57.25 |
| 11 | 146 | C06 | 1 µM FCCP | 02:07:55 | 51.73 |
| 11 | 147 | C06 | 1 µM FCCP | 02:08:09 | 46.64 |
| 11 | 148 | C06 | 1 µM FCCP | 02:08:23 | 42.07 |
| 11 | 149 | C06 | 1 µM FCCP | 02:08:37 | 37.95 |
| 11 | 150 | C06 | 1 µM FCCP | 02:08:52 | 34.45 |
| 11 | 151 | C06 | 1 µM FCCP | 02:09:06 | 31.60 |
| 11 | 152 | C06 | 1 µM FCCP | 02:09:20 | 29.33 |
| 11 | 153 | C06 | 1 µM FCCP | 02:09:34 | 27.45 |
| 12 | 154 | C06 | 1 µM FCCP | 02:15:08 | 110.81 |
| 12 | 155 | C06 | 1 µM FCCP | 02:15:22 | 91.39 |
| 12 | 156 | C06 | 1 µM FCCP | 02:15:37 | 80.58 |
| 12 | 157 | C06 | 1 µM FCCP | 02:15:53 | 71.79 |
| 12 | 158 | C06 | 1 µM FCCP | 02:16:08 | 64.67 |
| 12 | 159 | C06 | 1 µM FCCP | 02:16:22 | 58.61 |
| 12 | 160 | C06 | 1 µM FCCP | 02:16:36 | 52.98 |
| 12 | 161 | C06 | 1 µM FCCP | 02:16:50 | 47.77 |
| 12 | 162 | C06 | 1 µM FCCP | 02:17:04 | 43.02 |
| 12 | 163 | C06 | 1 µM FCCP | 02:17:18 | 38.72 |
| 12 | 164 | C06 | 1 µM FCCP | 02:17:33 | 35.06 |
| 12 | 165 | C06 | 1 µM FCCP | 02:17:47 | 32.05 |
| 12 | 166 | C06 | 1 µM FCCP | 02:18:01 | 29.57 |
| 13 | 167 | C06 | 1 µM FCCP | 02:23:39 | 104.78 |
| 13 | 168 | C06 | 1 µM FCCP | 02:23:54 | 89.51 |
| 13 | 169 | C06 | 1 µM FCCP | 02:24:08 | 80.14 |
| 13 | 170 | C06 | 1 µM FCCP | 02:24:22 | 72.51 |
| 13 | 171 | C06 | 1 µM FCCP | 02:24:36 | 65.72 |
| 13 | 172 | C06 | 1 µM FCCP | 02:24:51 | 59.27 |
| 13 | 173 | C06 | 1 µM FCCP | 02:25:07 | 53.14 |
| 13 | 174 | C06 | 1 µM FCCP | 02:25:22 | 47.90 |
| 13 | 175 | C06 | 1 µM FCCP | 02:25:36 | 43.39 |
| 13 | 176 | C06 | 1 µM FCCP | 02:25:50 | 39.13 |
| 13 | 177 | C06 | 1 µM FCCP | 02:26:04 | 35.50 |
| 13 | 178 | C06 | 1 µM FCCP | 02:26:18 | 32.40 |
| 13 | 179 | C06 | 1 µM FCCP | 02:26:33 | 29.86 |
| 14 | 180 | C06 | 1 µM FCCP | 02:32:07 | 111.08 |
| 14 | 181 | C06 | 1 µM FCCP | 02:32:21 | 92.09 |
| 14 | 182 | C06 | 1 µM FCCP | 02:32:35 | 82.20 |
| 14 | 183 | C06 | 1 µM FCCP | 02:32:50 | 74.35 |
| 14 | 184 | C06 | 1 µM FCCP | 02:33:04 | 67.65 |
| 14 | 185 | C06 | 1 µM FCCP | 02:33:18 | 61.41 |
| 14 | 186 | C06 | 1 µM FCCP | 02:33:32 | 55.87 |
| 14 | 187 | C06 | 1 µM FCCP | 02:33:46 | 50.64 |
| 14 | 188 | C06 | 1 µM FCCP | 02:34:01 | 46.00 |
| 14 | 189 | C06 | 1 µM FCCP | 02:34:15 | 41.65 |
| 14 | 190 | C06 | 1 µM FCCP | 02:34:29 | 37.70 |
| 14 | 191 | C06 | 1 µM FCCP | 02:34:43 | 34.30 |
| 14 | 192 | C06 | 1 µM FCCP | 02:34:57 | 31.40 |
| 14 | 193 | C06 | 1 µM FCCP | 02:35:12 | 29.16 |
| 15 | 194 | C06 | 1 µM FCCP | 02:40:46 | 111.12 |
| 15 | 195 | C06 | 1 µM FCCP | 02:41:00 | 92.28 |
| 15 | 196 | C06 | 1 µM FCCP | 02:41:14 | 82.38 |
| 15 | 197 | C06 | 1 µM FCCP | 02:41:28 | 74.63 |
| 15 | 198 | C06 | 1 µM FCCP | 02:41:43 | 67.91 |
| 15 | 199 | C06 | 1 µM FCCP | 02:41:57 | 61.89 |
| 15 | 200 | C06 | 1 µM FCCP | 02:42:11 | 56.40 |
| 15 | 201 | C06 | 1 µM FCCP | 02:42:25 | 51.28 |
| 15 | 202 | C06 | 1 µM FCCP | 02:42:39 | 46.63 |
| 15 | 203 | C06 | 1 µM FCCP | 02:42:53 | 42.34 |
| 15 | 204 | C06 | 1 µM FCCP | 02:43:08 | 38.28 |
| 15 | 205 | C06 | 1 µM FCCP | 02:43:22 | 34.91 |
| 15 | 206 | C06 | 1 µM FCCP | 02:43:36 | 31.86 |
| 15 | 207 | C06 | 1 µM FCCP | 02:43:50 | 29.50 |
| 16 | 208 | C06 | 1 µM FCCP | 02:49:25 | 111.21 |
| 16 | 209 | C06 | 1 µM FCCP | 02:49:40 | 92.57 |
| 16 | 210 | C06 | 1 µM FCCP | 02:49:54 | 82.81 |
| 16 | 211 | C06 | 1 µM FCCP | 02:50:08 | 75.11 |
| 16 | 212 | C06 | 1 µM FCCP | 02:50:22 | 68.51 |
| 16 | 213 | C06 | 1 µM FCCP | 02:50:36 | 62.52 |
| 16 | 214 | C06 | 1 µM FCCP | 02:50:50 | 57.15 |
| 16 | 215 | C06 | 1 µM FCCP | 02:51:05 | 52.01 |
| 16 | 216 | C06 | 1 µM FCCP | 02:51:19 | 47.37 |
| 16 | 217 | C06 | 1 µM FCCP | 02:51:33 | 43.07 |
| 16 | 218 | C06 | 1 µM FCCP | 02:51:47 | 39.11 |
| 16 | 219 | C06 | 1 µM FCCP | 02:52:01 | 35.61 |
| 16 | 220 | C06 | 1 µM FCCP | 02:52:16 | 32.47 |
| 16 | 221 | C06 | 1 µM FCCP | 02:52:30 | 29.98 |
| 1 | 0 | F01 | 10 µM BAM15 | 00:40:06 | 138.20 |
| 1 | 1 | F01 | 10 µM BAM15 | 00:40:20 | 135.44 |
| 1 | 2 | F01 | 10 µM BAM15 | 00:40:34 | 133.84 |
| 1 | 3 | F01 | 10 µM BAM15 | 00:40:48 | 132.36 |
| 1 | 4 | F01 | 10 µM BAM15 | 00:41:02 | 131.14 |
| 1 | 5 | F01 | 10 µM BAM15 | 00:41:17 | 129.95 |
| 1 | 6 | F01 | 10 µM BAM15 | 00:41:31 | 128.99 |
| 1 | 7 | F01 | 10 µM BAM15 | 00:41:45 | 127.93 |
| 1 | 8 | F01 | 10 µM BAM15 | 00:41:59 | 127.02 |
| 1 | 9 | F01 | 10 µM BAM15 | 00:42:13 | 126.15 |
| 1 | 10 | F01 | 10 µM BAM15 | 00:42:28 | 125.29 |
| 1 | 11 | F01 | 10 µM BAM15 | 00:42:42 | 124.49 |
| 1 | 12 | F01 | 10 µM BAM15 | 00:42:56 | 123.56 |
| 1 | 13 | F01 | 10 µM BAM15 | 00:43:10 | 122.81 |
| 2 | 14 | F01 | 10 µM BAM15 | 00:48:43 | 139.21 |
| 2 | 15 | F01 | 10 µM BAM15 | 00:48:57 | 136.11 |
| 2 | 16 | F01 | 10 µM BAM15 | 00:49:12 | 134.41 |
| 2 | 17 | F01 | 10 µM BAM15 | 00:49:26 | 132.99 |
| 2 | 18 | F01 | 10 µM BAM15 | 00:49:40 | 131.76 |
| 2 | 19 | F01 | 10 µM BAM15 | 00:49:54 | 130.43 |
| 2 | 20 | F01 | 10 µM BAM15 | 00:50:08 | 129.42 |
| 2 | 21 | F01 | 10 µM BAM15 | 00:50:22 | 128.40 |
| 2 | 22 | F01 | 10 µM BAM15 | 00:50:37 | 127.40 |
| 2 | 23 | F01 | 10 µM BAM15 | 00:50:51 | 126.50 |
| 2 | 24 | F01 | 10 µM BAM15 | 00:51:05 | 125.67 |
| 2 | 25 | F01 | 10 µM BAM15 | 00:51:19 | 124.81 |
| 2 | 26 | F01 | 10 µM BAM15 | 00:51:33 | 123.92 |
| 2 | 27 | F01 | 10 µM BAM15 | 00:51:48 | 123.09 |
| 3 | 28 | F01 | 10 µM BAM15 | 00:57:21 | 139.66 |
| 3 | 29 | F01 | 10 µM BAM15 | 00:57:35 | 136.72 |
| 3 | 30 | F01 | 10 µM BAM15 | 00:57:49 | 134.99 |
| 3 | 31 | F01 | 10 µM BAM15 | 00:58:03 | 133.48 |
| 3 | 32 | F01 | 10 µM BAM15 | 00:58:17 | 132.33 |
| 3 | 33 | F01 | 10 µM BAM15 | 00:58:31 | 131.09 |
| 3 | 34 | F01 | 10 µM BAM15 | 00:58:46 | 130.01 |
| 3 | 35 | F01 | 10 µM BAM15 | 00:59:00 | 129.04 |
| 3 | 36 | F01 | 10 µM BAM15 | 00:59:14 | 127.89 |
| 3 | 37 | F01 | 10 µM BAM15 | 00:59:28 | 127.05 |
| 3 | 38 | F01 | 10 µM BAM15 | 00:59:42 | 126.08 |
| 3 | 39 | F01 | 10 µM BAM15 | 00:59:56 | 125.26 |
| 3 | 40 | F01 | 10 µM BAM15 | 01:00:11 | 124.35 |
| 3 | 41 | F01 | 10 µM BAM15 | 01:00:25 | 123.57 |
| 4 | 42 | F01 | 10 µM BAM15 | 01:06:04 | 131.06 |
| 4 | 43 | F01 | 10 µM BAM15 | 01:06:18 | 120.91 |
| 4 | 44 | F01 | 10 µM BAM15 | 01:06:33 | 114.79 |
| 4 | 45 | F01 | 10 µM BAM15 | 01:06:47 | 110.00 |
| 4 | 46 | F01 | 10 µM BAM15 | 01:07:01 | 105.86 |
| 4 | 47 | F01 | 10 µM BAM15 | 01:07:15 | 101.97 |
| 4 | 48 | F01 | 10 µM BAM15 | 01:07:29 | 98.39 |
| 4 | 49 | F01 | 10 µM BAM15 | 01:07:43 | 95.06 |
| 4 | 50 | F01 | 10 µM BAM15 | 01:07:58 | 92.08 |
| 4 | 51 | F01 | 10 µM BAM15 | 01:08:12 | 89.20 |
| 4 | 52 | F01 | 10 µM BAM15 | 01:08:26 | 86.43 |
| 4 | 53 | F01 | 10 µM BAM15 | 01:08:40 | 83.90 |
| 4 | 54 | F01 | 10 µM BAM15 | 01:08:54 | 81.38 |
| 4 | 55 | F01 | 10 µM BAM15 | 01:09:09 | 79.06 |
| 5 | 56 | F01 | 10 µM BAM15 | 01:14:42 | 122.73 |
| 5 | 57 | F01 | 10 µM BAM15 | 01:14:56 | 110.99 |
| 5 | 58 | F01 | 10 µM BAM15 | 01:15:10 | 104.50 |
| 5 | 59 | F01 | 10 µM BAM15 | 01:15:25 | 99.55 |
| 5 | 60 | F01 | 10 µM BAM15 | 01:15:39 | 95.24 |
| 5 | 61 | F01 | 10 µM BAM15 | 01:15:53 | 91.25 |
| 5 | 62 | F01 | 10 µM BAM15 | 01:16:07 | 87.72 |
| 5 | 63 | F01 | 10 µM BAM15 | 01:16:21 | 84.35 |
| 5 | 64 | F01 | 10 µM BAM15 | 01:16:35 | 81.26 |
| 5 | 65 | F01 | 10 µM BAM15 | 01:16:50 | 78.33 |
| 5 | 66 | F01 | 10 µM BAM15 | 01:17:04 | 75.49 |
| 5 | 67 | F01 | 10 µM BAM15 | 01:17:18 | 72.86 |
| 5 | 68 | F01 | 10 µM BAM15 | 01:17:32 | 70.36 |
| 5 | 69 | F01 | 10 µM BAM15 | 01:17:46 | 67.99 |
| 6 | 70 | F01 | 10 µM BAM15 | 01:23:20 | 118.13 |
| 6 | 71 | F01 | 10 µM BAM15 | 01:23:34 | 105.75 |
| 6 | 72 | F01 | 10 µM BAM15 | 01:23:48 | 99.10 |
| 6 | 73 | F01 | 10 µM BAM15 | 01:24:02 | 93.90 |
| 6 | 74 | F01 | 10 µM BAM15 | 01:24:16 | 89.43 |
| 6 | 75 | F01 | 10 µM BAM15 | 01:24:30 | 85.21 |
| 6 | 76 | F01 | 10 µM BAM15 | 01:24:44 | 81.58 |
| 6 | 77 | F01 | 10 µM BAM15 | 01:24:59 | 77.98 |
| 6 | 78 | F01 | 10 µM BAM15 | 01:25:13 | 74.75 |
| 6 | 79 | F01 | 10 µM BAM15 | 01:25:27 | 71.68 |
| 6 | 80 | F01 | 10 µM BAM15 | 01:25:41 | 68.74 |
| 6 | 81 | F01 | 10 µM BAM15 | 01:25:55 | 66.03 |
| 6 | 82 | F01 | 10 µM BAM15 | 01:26:10 | 63.30 |
| 6 | 83 | F01 | 10 µM BAM15 | 01:26:24 | 60.81 |
| 7 | 84 | F01 | 10 µM BAM15 | 01:31:58 | 115.27 |
| 7 | 85 | F01 | 10 µM BAM15 | 01:32:12 | 102.63 |
| 7 | 86 | F01 | 10 µM BAM15 | 01:32:26 | 95.87 |
| 7 | 87 | F01 | 10 µM BAM15 | 01:32:40 | 90.60 |
| 7 | 88 | F01 | 10 µM BAM15 | 01:32:54 | 85.96 |
| 7 | 89 | F01 | 10 µM BAM15 | 01:33:08 | 81.72 |
| 7 | 90 | F01 | 10 µM BAM15 | 01:33:23 | 77.88 |
| 7 | 91 | F01 | 10 µM BAM15 | 01:33:37 | 74.22 |
| 7 | 92 | F01 | 10 µM BAM15 | 01:33:51 | 70.92 |
| 7 | 93 | F01 | 10 µM BAM15 | 01:34:05 | 67.76 |
| 7 | 94 | F01 | 10 µM BAM15 | 01:34:19 | 64.70 |
| 7 | 95 | F01 | 10 µM BAM15 | 01:34:33 | 61.91 |
| 7 | 96 | F01 | 10 µM BAM15 | 01:34:48 | 59.22 |
| 7 | 97 | F01 | 10 µM BAM15 | 01:35:02 | 56.66 |
| 8 | 98 | F01 | 10 µM BAM15 | 01:40:36 | 113.55 |
| 8 | 99 | F01 | 10 µM BAM15 | 01:40:50 | 101.08 |
| 8 | 100 | F01 | 10 µM BAM15 | 01:41:04 | 94.25 |
| 8 | 101 | F01 | 10 µM BAM15 | 01:41:18 | 88.86 |
| 8 | 102 | F01 | 10 µM BAM15 | 01:41:32 | 84.19 |
| 8 | 103 | F01 | 10 µM BAM15 | 01:41:46 | 79.85 |
| 8 | 104 | F01 | 10 µM BAM15 | 01:42:00 | 75.98 |
| 8 | 105 | F01 | 10 µM BAM15 | 01:42:15 | 72.29 |
| 8 | 106 | F01 | 10 µM BAM15 | 01:42:29 | 68.87 |
| 8 | 107 | F01 | 10 µM BAM15 | 01:42:43 | 65.64 |
| 8 | 108 | F01 | 10 µM BAM15 | 01:42:57 | 62.56 |
| 8 | 109 | F01 | 10 µM BAM15 | 01:43:11 | 59.68 |
| 8 | 110 | F01 | 10 µM BAM15 | 01:43:26 | 56.84 |
| 8 | 111 | F01 | 10 µM BAM15 | 01:43:40 | 54.30 |
| 9 | 112 | F01 | 10 µM BAM15 | 01:49:14 | 114.20 |
| 9 | 113 | F01 | 10 µM BAM15 | 01:49:28 | 103.64 |
| 9 | 114 | F01 | 10 µM BAM15 | 01:49:42 | 97.23 |
| 9 | 115 | F01 | 10 µM BAM15 | 01:49:56 | 92.02 |
| 9 | 116 | F01 | 10 µM BAM15 | 01:50:10 | 87.39 |
| 9 | 117 | F01 | 10 µM BAM15 | 01:50:24 | 83.11 |
| 9 | 118 | F01 | 10 µM BAM15 | 01:50:38 | 79.22 |
| 9 | 119 | F01 | 10 µM BAM15 | 01:50:53 | 75.46 |
| 9 | 120 | F01 | 10 µM BAM15 | 01:51:07 | 71.99 |
| 9 | 121 | F01 | 10 µM BAM15 | 01:51:21 | 68.71 |
| 9 | 122 | F01 | 10 µM BAM15 | 01:51:35 | 65.56 |
| 9 | 123 | F01 | 10 µM BAM15 | 01:51:50 | 62.62 |
| 9 | 124 | F01 | 10 µM BAM15 | 01:52:04 | 59.77 |
| 9 | 125 | F01 | 10 µM BAM15 | 01:52:18 | 57.06 |
| 10 | 126 | F01 | 10 µM BAM15 | 01:57:52 | 114.39 |
| 10 | 127 | F01 | 10 µM BAM15 | 01:58:06 | 104.12 |
| 10 | 128 | F01 | 10 µM BAM15 | 01:58:20 | 97.86 |
| 10 | 129 | F01 | 10 µM BAM15 | 01:58:34 | 92.83 |
| 10 | 130 | F01 | 10 µM BAM15 | 01:58:48 | 88.21 |
| 10 | 131 | F01 | 10 µM BAM15 | 01:59:03 | 83.95 |
| 10 | 132 | F01 | 10 µM BAM15 | 01:59:17 | 80.13 |
| 10 | 133 | F01 | 10 µM BAM15 | 01:59:31 | 76.35 |
| 10 | 134 | F01 | 10 µM BAM15 | 01:59:45 | 73.05 |
| 10 | 135 | F01 | 10 µM BAM15 | 01:59:59 | 69.74 |
| 10 | 136 | F01 | 10 µM BAM15 | 02:00:14 | 66.66 |
| 10 | 137 | F01 | 10 µM BAM15 | 02:00:28 | 63.76 |
| 10 | 138 | F01 | 10 µM BAM15 | 02:00:42 | 60.90 |
| 10 | 139 | F01 | 10 µM BAM15 | 02:00:56 | 58.24 |
| 11 | 140 | F01 | 10 µM BAM15 | 02:06:30 | 114.44 |
| 11 | 141 | F01 | 10 µM BAM15 | 02:06:44 | 104.25 |
| 11 | 142 | F01 | 10 µM BAM15 | 02:06:58 | 98.05 |
| 11 | 143 | F01 | 10 µM BAM15 | 02:07:12 | 92.99 |
| 11 | 144 | F01 | 10 µM BAM15 | 02:07:26 | 88.52 |
| 11 | 145 | F01 | 10 µM BAM15 | 02:07:41 | 84.27 |
| 11 | 146 | F01 | 10 µM BAM15 | 02:07:55 | 80.43 |
| 11 | 147 | F01 | 10 µM BAM15 | 02:08:09 | 76.77 |
| 11 | 148 | F01 | 10 µM BAM15 | 02:08:23 | 73.37 |
| 11 | 149 | F01 | 10 µM BAM15 | 02:08:37 | 70.15 |
| 11 | 150 | F01 | 10 µM BAM15 | 02:08:52 | 67.07 |
| 11 | 151 | F01 | 10 µM BAM15 | 02:09:06 | 64.12 |
| 11 | 152 | F01 | 10 µM BAM15 | 02:09:20 | 61.32 |
| 11 | 153 | F01 | 10 µM BAM15 | 02:09:34 | 58.66 |
| 12 | 154 | F01 | 10 µM BAM15 | 02:15:08 | 114.30 |
| 12 | 155 | F01 | 10 µM BAM15 | 02:15:22 | 104.30 |
| 12 | 156 | F01 | 10 µM BAM15 | 02:15:37 | 97.66 |
| 12 | 157 | F01 | 10 µM BAM15 | 02:15:53 | 92.16 |
| 12 | 158 | F01 | 10 µM BAM15 | 02:16:08 | 87.62 |
| 12 | 159 | F01 | 10 µM BAM15 | 02:16:22 | 83.60 |
| 12 | 160 | F01 | 10 µM BAM15 | 02:16:36 | 79.76 |
| 12 | 161 | F01 | 10 µM BAM15 | 02:16:50 | 76.20 |
| 12 | 162 | F01 | 10 µM BAM15 | 02:17:04 | 72.85 |
| 12 | 163 | F01 | 10 µM BAM15 | 02:17:18 | 69.62 |
| 12 | 164 | F01 | 10 µM BAM15 | 02:17:33 | 66.64 |
| 12 | 165 | F01 | 10 µM BAM15 | 02:17:47 | 63.75 |
| 12 | 166 | F01 | 10 µM BAM15 | 02:18:01 | 60.93 |
| 13 | 167 | F01 | 10 µM BAM15 | 02:23:39 | 111.10 |
| 13 | 168 | F01 | 10 µM BAM15 | 02:23:54 | 102.69 |
| 13 | 169 | F01 | 10 µM BAM15 | 02:24:08 | 96.93 |
| 13 | 170 | F01 | 10 µM BAM15 | 02:24:22 | 92.08 |
| 13 | 171 | F01 | 10 µM BAM15 | 02:24:36 | 87.71 |
| 13 | 172 | F01 | 10 µM BAM15 | 02:24:51 | 83.39 |
| 13 | 173 | F01 | 10 µM BAM15 | 02:25:07 | 79.25 |
| 13 | 174 | F01 | 10 µM BAM15 | 02:25:22 | 75.77 |
| 13 | 175 | F01 | 10 µM BAM15 | 02:25:36 | 72.54 |
| 13 | 176 | F01 | 10 µM BAM15 | 02:25:50 | 69.33 |
| 13 | 177 | F01 | 10 µM BAM15 | 02:26:04 | 66.43 |
| 13 | 178 | F01 | 10 µM BAM15 | 02:26:18 | 63.55 |
| 13 | 179 | F01 | 10 µM BAM15 | 02:26:33 | 60.80 |
| 14 | 180 | F01 | 10 µM BAM15 | 02:32:07 | 114.18 |
| 14 | 181 | F01 | 10 µM BAM15 | 02:32:21 | 104.15 |
| 14 | 182 | F01 | 10 µM BAM15 | 02:32:35 | 98.10 |
| 14 | 183 | F01 | 10 µM BAM15 | 02:32:50 | 93.12 |
| 14 | 184 | F01 | 10 µM BAM15 | 02:33:04 | 88.67 |
| 14 | 185 | F01 | 10 µM BAM15 | 02:33:18 | 84.48 |
| 14 | 186 | F01 | 10 µM BAM15 | 02:33:32 | 80.64 |
| 14 | 187 | F01 | 10 µM BAM15 | 02:33:46 | 77.04 |
| 14 | 188 | F01 | 10 µM BAM15 | 02:34:01 | 73.73 |
| 14 | 189 | F01 | 10 µM BAM15 | 02:34:15 | 70.52 |
| 14 | 190 | F01 | 10 µM BAM15 | 02:34:29 | 67.49 |
| 14 | 191 | F01 | 10 µM BAM15 | 02:34:43 | 64.56 |
| 14 | 192 | F01 | 10 µM BAM15 | 02:34:57 | 61.74 |
| 14 | 193 | F01 | 10 µM BAM15 | 02:35:12 | 59.18 |
| 15 | 194 | F01 | 10 µM BAM15 | 02:40:46 | 114.09 |
| 15 | 195 | F01 | 10 µM BAM15 | 02:41:00 | 104.28 |
| 15 | 196 | F01 | 10 µM BAM15 | 02:41:14 | 98.17 |
| 15 | 197 | F01 | 10 µM BAM15 | 02:41:28 | 93.30 |
| 15 | 198 | F01 | 10 µM BAM15 | 02:41:43 | 88.81 |
| 15 | 199 | F01 | 10 µM BAM15 | 02:41:57 | 84.78 |
| 15 | 200 | F01 | 10 µM BAM15 | 02:42:11 | 80.97 |
| 15 | 201 | F01 | 10 µM BAM15 | 02:42:25 | 77.36 |
| 15 | 202 | F01 | 10 µM BAM15 | 02:42:39 | 74.03 |
| 15 | 203 | F01 | 10 µM BAM15 | 02:42:53 | 70.86 |
| 15 | 204 | F01 | 10 µM BAM15 | 02:43:08 | 67.75 |
| 15 | 205 | F01 | 10 µM BAM15 | 02:43:22 | 64.96 |
| 15 | 206 | F01 | 10 µM BAM15 | 02:43:36 | 62.08 |
| 15 | 207 | F01 | 10 µM BAM15 | 02:43:50 | 59.42 |
| 16 | 208 | F01 | 10 µM BAM15 | 02:49:25 | 114.04 |
| 16 | 209 | F01 | 10 µM BAM15 | 02:49:40 | 104.32 |
| 16 | 210 | F01 | 10 µM BAM15 | 02:49:54 | 98.31 |
| 16 | 211 | F01 | 10 µM BAM15 | 02:50:08 | 93.33 |
| 16 | 212 | F01 | 10 µM BAM15 | 02:50:22 | 88.96 |
| 16 | 213 | F01 | 10 µM BAM15 | 02:50:36 | 84.90 |
| 16 | 214 | F01 | 10 µM BAM15 | 02:50:50 | 81.15 |
| 16 | 215 | F01 | 10 µM BAM15 | 02:51:05 | 77.54 |
| 16 | 216 | F01 | 10 µM BAM15 | 02:51:19 | 74.22 |
| 16 | 217 | F01 | 10 µM BAM15 | 02:51:33 | 71.07 |
| 16 | 218 | F01 | 10 µM BAM15 | 02:51:47 | 68.06 |
| 16 | 219 | F01 | 10 µM BAM15 | 02:52:01 | 65.18 |
| 16 | 220 | F01 | 10 µM BAM15 | 02:52:16 | 62.32 |
| 16 | 221 | F01 | 10 µM BAM15 | 02:52:30 | 59.69 |
| 1 | 0 | F02 | 10 µM BAM15 | 00:40:06 | 130.25 |
| 1 | 1 | F02 | 10 µM BAM15 | 00:40:20 | 124.96 |
| 1 | 2 | F02 | 10 µM BAM15 | 00:40:34 | 122.43 |
| 1 | 3 | F02 | 10 µM BAM15 | 00:40:48 | 120.48 |
| 1 | 4 | F02 | 10 µM BAM15 | 00:41:02 | 118.87 |
| 1 | 5 | F02 | 10 µM BAM15 | 00:41:17 | 117.23 |
| 1 | 6 | F02 | 10 µM BAM15 | 00:41:31 | 115.90 |
| 1 | 7 | F02 | 10 µM BAM15 | 00:41:45 | 114.59 |
| 1 | 8 | F02 | 10 µM BAM15 | 00:41:59 | 113.31 |
| 1 | 9 | F02 | 10 µM BAM15 | 00:42:13 | 112.18 |
| 1 | 10 | F02 | 10 µM BAM15 | 00:42:28 | 111.04 |
| 1 | 11 | F02 | 10 µM BAM15 | 00:42:42 | 109.95 |
| 1 | 12 | F02 | 10 µM BAM15 | 00:42:56 | 108.96 |
| 1 | 13 | F02 | 10 µM BAM15 | 00:43:10 | 107.94 |
| 2 | 14 | F02 | 10 µM BAM15 | 00:48:43 | 133.15 |
| 2 | 15 | F02 | 10 µM BAM15 | 00:48:57 | 129.01 |
| 2 | 16 | F02 | 10 µM BAM15 | 00:49:12 | 126.71 |
| 2 | 17 | F02 | 10 µM BAM15 | 00:49:26 | 124.68 |
| 2 | 18 | F02 | 10 µM BAM15 | 00:49:40 | 123.07 |
| 2 | 19 | F02 | 10 µM BAM15 | 00:49:54 | 121.46 |
| 2 | 20 | F02 | 10 µM BAM15 | 00:50:08 | 119.99 |
| 2 | 21 | F02 | 10 µM BAM15 | 00:50:22 | 118.51 |
| 2 | 22 | F02 | 10 µM BAM15 | 00:50:37 | 117.35 |
| 2 | 23 | F02 | 10 µM BAM15 | 00:50:51 | 116.13 |
| 2 | 24 | F02 | 10 µM BAM15 | 00:51:05 | 114.84 |
| 2 | 25 | F02 | 10 µM BAM15 | 00:51:19 | 113.75 |
| 2 | 26 | F02 | 10 µM BAM15 | 00:51:33 | 112.65 |
| 2 | 27 | F02 | 10 µM BAM15 | 00:51:48 | 111.64 |
| 3 | 28 | F02 | 10 µM BAM15 | 00:57:21 | 134.76 |
| 3 | 29 | F02 | 10 µM BAM15 | 00:57:35 | 131.06 |
| 3 | 30 | F02 | 10 µM BAM15 | 00:57:49 | 128.86 |
| 3 | 31 | F02 | 10 µM BAM15 | 00:58:03 | 126.92 |
| 3 | 32 | F02 | 10 µM BAM15 | 00:58:17 | 125.25 |
| 3 | 33 | F02 | 10 µM BAM15 | 00:58:31 | 123.68 |
| 3 | 34 | F02 | 10 µM BAM15 | 00:58:46 | 122.19 |
| 3 | 35 | F02 | 10 µM BAM15 | 00:59:00 | 120.78 |
| 3 | 36 | F02 | 10 µM BAM15 | 00:59:14 | 119.42 |
| 3 | 37 | F02 | 10 µM BAM15 | 00:59:28 | 118.24 |
| 3 | 38 | F02 | 10 µM BAM15 | 00:59:42 | 117.01 |
| 3 | 39 | F02 | 10 µM BAM15 | 00:59:56 | 115.91 |
| 3 | 40 | F02 | 10 µM BAM15 | 01:00:11 | 114.78 |
| 3 | 41 | F02 | 10 µM BAM15 | 01:00:25 | 113.71 |
| 4 | 42 | F02 | 10 µM BAM15 | 01:06:04 | 119.26 |
| 4 | 43 | F02 | 10 µM BAM15 | 01:06:18 | 105.51 |
| 4 | 44 | F02 | 10 µM BAM15 | 01:06:33 | 97.09 |
| 4 | 45 | F02 | 10 µM BAM15 | 01:06:47 | 90.44 |
| 4 | 46 | F02 | 10 µM BAM15 | 01:07:01 | 84.60 |
| 4 | 47 | F02 | 10 µM BAM15 | 01:07:15 | 79.15 |
| 4 | 48 | F02 | 10 µM BAM15 | 01:07:29 | 74.16 |
| 4 | 49 | F02 | 10 µM BAM15 | 01:07:43 | 69.44 |
| 4 | 50 | F02 | 10 µM BAM15 | 01:07:58 | 65.15 |
| 4 | 51 | F02 | 10 µM BAM15 | 01:08:12 | 61.07 |
| 4 | 52 | F02 | 10 µM BAM15 | 01:08:26 | 57.15 |
| 4 | 53 | F02 | 10 µM BAM15 | 01:08:40 | 53.55 |
| 4 | 54 | F02 | 10 µM BAM15 | 01:08:54 | 50.04 |
| 4 | 55 | F02 | 10 µM BAM15 | 01:09:09 | 46.84 |
| 5 | 56 | F02 | 10 µM BAM15 | 01:14:42 | 109.91 |
| 5 | 57 | F02 | 10 µM BAM15 | 01:14:56 | 95.00 |
| 5 | 58 | F02 | 10 µM BAM15 | 01:15:10 | 86.43 |
| 5 | 59 | F02 | 10 µM BAM15 | 01:15:25 | 79.71 |
| 5 | 60 | F02 | 10 µM BAM15 | 01:15:39 | 73.80 |
| 5 | 61 | F02 | 10 µM BAM15 | 01:15:53 | 68.32 |
| 5 | 62 | F02 | 10 µM BAM15 | 01:16:07 | 63.37 |
| 5 | 63 | F02 | 10 µM BAM15 | 01:16:21 | 58.75 |
| 5 | 64 | F02 | 10 µM BAM15 | 01:16:35 | 54.51 |
| 5 | 65 | F02 | 10 µM BAM15 | 01:16:50 | 50.49 |
| 5 | 66 | F02 | 10 µM BAM15 | 01:17:04 | 46.69 |
| 5 | 67 | F02 | 10 µM BAM15 | 01:17:18 | 43.25 |
| 5 | 68 | F02 | 10 µM BAM15 | 01:17:32 | 40.05 |
| 5 | 69 | F02 | 10 µM BAM15 | 01:17:46 | 37.29 |
| 6 | 70 | F02 | 10 µM BAM15 | 01:23:20 | 106.18 |
| 6 | 71 | F02 | 10 µM BAM15 | 01:23:34 | 90.98 |
| 6 | 72 | F02 | 10 µM BAM15 | 01:23:48 | 82.49 |
| 6 | 73 | F02 | 10 µM BAM15 | 01:24:02 | 75.60 |
| 6 | 74 | F02 | 10 µM BAM15 | 01:24:16 | 69.72 |
| 6 | 75 | F02 | 10 µM BAM15 | 01:24:30 | 64.18 |
| 6 | 76 | F02 | 10 µM BAM15 | 01:24:44 | 59.22 |
| 6 | 77 | F02 | 10 µM BAM15 | 01:24:59 | 54.48 |
| 6 | 78 | F02 | 10 µM BAM15 | 01:25:13 | 50.16 |
| 6 | 79 | F02 | 10 µM BAM15 | 01:25:27 | 46.15 |
| 6 | 80 | F02 | 10 µM BAM15 | 01:25:41 | 42.39 |
| 6 | 81 | F02 | 10 µM BAM15 | 01:25:55 | 39.07 |
| 6 | 82 | F02 | 10 µM BAM15 | 01:26:10 | 36.03 |
| 6 | 83 | F02 | 10 µM BAM15 | 01:26:24 | 33.51 |
| 7 | 84 | F02 | 10 µM BAM15 | 01:31:58 | 104.17 |
| 7 | 85 | F02 | 10 µM BAM15 | 01:32:12 | 89.01 |
| 7 | 86 | F02 | 10 µM BAM15 | 01:32:26 | 80.40 |
| 7 | 87 | F02 | 10 µM BAM15 | 01:32:40 | 73.61 |
| 7 | 88 | F02 | 10 µM BAM15 | 01:32:54 | 67.55 |
| 7 | 89 | F02 | 10 µM BAM15 | 01:33:08 | 62.03 |
| 7 | 90 | F02 | 10 µM BAM15 | 01:33:23 | 56.96 |
| 7 | 91 | F02 | 10 µM BAM15 | 01:33:37 | 52.17 |
| 7 | 92 | F02 | 10 µM BAM15 | 01:33:51 | 47.85 |
| 7 | 93 | F02 | 10 µM BAM15 | 01:34:05 | 43.86 |
| 7 | 94 | F02 | 10 µM BAM15 | 01:34:19 | 40.10 |
| 7 | 95 | F02 | 10 µM BAM15 | 01:34:33 | 36.91 |
| 7 | 96 | F02 | 10 µM BAM15 | 01:34:48 | 34.07 |
| 7 | 97 | F02 | 10 µM BAM15 | 01:35:02 | 31.73 |
| 8 | 98 | F02 | 10 µM BAM15 | 01:40:36 | 103.02 |
| 8 | 99 | F02 | 10 µM BAM15 | 01:40:50 | 87.69 |
| 8 | 100 | F02 | 10 µM BAM15 | 01:41:04 | 78.98 |
| 8 | 101 | F02 | 10 µM BAM15 | 01:41:18 | 71.94 |
| 8 | 102 | F02 | 10 µM BAM15 | 01:41:32 | 65.82 |
| 8 | 103 | F02 | 10 µM BAM15 | 01:41:46 | 60.19 |
| 8 | 104 | F02 | 10 µM BAM15 | 01:42:00 | 55.05 |
| 8 | 105 | F02 | 10 µM BAM15 | 01:42:15 | 50.22 |
| 8 | 106 | F02 | 10 µM BAM15 | 01:42:29 | 45.77 |
| 8 | 107 | F02 | 10 µM BAM15 | 01:42:43 | 41.70 |
| 8 | 108 | F02 | 10 µM BAM15 | 01:42:57 | 37.99 |
| 8 | 109 | F02 | 10 µM BAM15 | 01:43:11 | 34.81 |
| 8 | 110 | F02 | 10 µM BAM15 | 01:43:26 | 32.06 |
| 8 | 111 | F02 | 10 µM BAM15 | 01:43:40 | 29.91 |
| 9 | 112 | F02 | 10 µM BAM15 | 01:49:14 | 102.21 |
| 9 | 113 | F02 | 10 µM BAM15 | 01:49:28 | 86.83 |
| 9 | 114 | F02 | 10 µM BAM15 | 01:49:42 | 77.98 |
| 9 | 115 | F02 | 10 µM BAM15 | 01:49:56 | 70.95 |
| 9 | 116 | F02 | 10 µM BAM15 | 01:50:10 | 64.65 |
| 9 | 117 | F02 | 10 µM BAM15 | 01:50:24 | 58.99 |
| 9 | 118 | F02 | 10 µM BAM15 | 01:50:38 | 53.82 |
| 9 | 119 | F02 | 10 µM BAM15 | 01:50:53 | 48.85 |
| 9 | 120 | F02 | 10 µM BAM15 | 01:51:07 | 44.39 |
| 9 | 121 | F02 | 10 µM BAM15 | 01:51:21 | 40.27 |
| 9 | 122 | F02 | 10 µM BAM15 | 01:51:35 | 36.61 |
| 9 | 123 | F02 | 10 µM BAM15 | 01:51:50 | 33.48 |
| 9 | 124 | F02 | 10 µM BAM15 | 01:52:04 | 30.93 |
| 9 | 125 | F02 | 10 µM BAM15 | 01:52:18 | 28.85 |
| 10 | 126 | F02 | 10 µM BAM15 | 01:57:52 | 101.57 |
| 10 | 127 | F02 | 10 µM BAM15 | 01:58:06 | 86.53 |
| 10 | 128 | F02 | 10 µM BAM15 | 01:58:20 | 77.73 |
| 10 | 129 | F02 | 10 µM BAM15 | 01:58:34 | 70.72 |
| 10 | 130 | F02 | 10 µM BAM15 | 01:58:48 | 64.44 |
| 10 | 131 | F02 | 10 µM BAM15 | 01:59:03 | 58.63 |
| 10 | 132 | F02 | 10 µM BAM15 | 01:59:17 | 53.38 |
| 10 | 133 | F02 | 10 µM BAM15 | 01:59:31 | 48.47 |
| 10 | 134 | F02 | 10 µM BAM15 | 01:59:45 | 44.03 |
| 10 | 135 | F02 | 10 µM BAM15 | 01:59:59 | 39.89 |
| 10 | 136 | F02 | 10 µM BAM15 | 02:00:14 | 36.23 |
| 10 | 137 | F02 | 10 µM BAM15 | 02:00:28 | 33.15 |
| 10 | 138 | F02 | 10 µM BAM15 | 02:00:42 | 30.63 |
| 10 | 139 | F02 | 10 µM BAM15 | 02:00:56 | 28.64 |
| 11 | 140 | F02 | 10 µM BAM15 | 02:06:30 | 103.38 |
| 11 | 141 | F02 | 10 µM BAM15 | 02:06:44 | 90.06 |
| 11 | 142 | F02 | 10 µM BAM15 | 02:06:58 | 81.76 |
| 11 | 143 | F02 | 10 µM BAM15 | 02:07:12 | 74.87 |
| 11 | 144 | F02 | 10 µM BAM15 | 02:07:26 | 68.75 |
| 11 | 145 | F02 | 10 µM BAM15 | 02:07:41 | 63.01 |
| 11 | 146 | F02 | 10 µM BAM15 | 02:07:55 | 57.78 |
| 11 | 147 | F02 | 10 µM BAM15 | 02:08:09 | 52.83 |
| 11 | 148 | F02 | 10 µM BAM15 | 02:08:23 | 48.30 |
| 11 | 149 | F02 | 10 µM BAM15 | 02:08:37 | 44.09 |
| 11 | 150 | F02 | 10 µM BAM15 | 02:08:52 | 40.19 |
| 11 | 151 | F02 | 10 µM BAM15 | 02:09:06 | 36.71 |
| 11 | 152 | F02 | 10 µM BAM15 | 02:09:20 | 33.70 |
| 11 | 153 | F02 | 10 µM BAM15 | 02:09:34 | 31.15 |
| 12 | 154 | F02 | 10 µM BAM15 | 02:15:08 | 104.88 |
| 12 | 155 | F02 | 10 µM BAM15 | 02:15:22 | 92.21 |
| 12 | 156 | F02 | 10 µM BAM15 | 02:15:37 | 83.57 |
| 12 | 157 | F02 | 10 µM BAM15 | 02:15:53 | 76.19 |
| 12 | 158 | F02 | 10 µM BAM15 | 02:16:08 | 70.06 |
| 12 | 159 | F02 | 10 µM BAM15 | 02:16:22 | 64.71 |
| 12 | 160 | F02 | 10 µM BAM15 | 02:16:36 | 59.60 |
| 12 | 161 | F02 | 10 µM BAM15 | 02:16:50 | 54.83 |
| 12 | 162 | F02 | 10 µM BAM15 | 02:17:04 | 50.35 |
| 12 | 163 | F02 | 10 µM BAM15 | 02:17:18 | 46.16 |
| 12 | 164 | F02 | 10 µM BAM15 | 02:17:33 | 42.30 |
| 12 | 165 | F02 | 10 µM BAM15 | 02:17:47 | 38.80 |
| 12 | 166 | F02 | 10 µM BAM15 | 02:18:01 | 35.65 |
| 13 | 167 | F02 | 10 µM BAM15 | 02:23:39 | 101.19 |
| 13 | 168 | F02 | 10 µM BAM15 | 02:23:54 | 90.54 |
| 13 | 169 | F02 | 10 µM BAM15 | 02:24:08 | 82.95 |
| 13 | 170 | F02 | 10 µM BAM15 | 02:24:22 | 76.53 |
| 13 | 171 | F02 | 10 µM BAM15 | 02:24:36 | 70.69 |
| 13 | 172 | F02 | 10 µM BAM15 | 02:24:51 | 64.89 |
| 13 | 173 | F02 | 10 µM BAM15 | 02:25:07 | 59.35 |
| 13 | 174 | F02 | 10 µM BAM15 | 02:25:22 | 54.66 |
| 13 | 175 | F02 | 10 µM BAM15 | 02:25:36 | 50.43 |
| 13 | 176 | F02 | 10 µM BAM15 | 02:25:50 | 46.30 |
| 13 | 177 | F02 | 10 µM BAM15 | 02:26:04 | 42.63 |
| 13 | 178 | F02 | 10 µM BAM15 | 02:26:18 | 39.16 |
| 13 | 179 | F02 | 10 µM BAM15 | 02:26:33 | 36.07 |
| 14 | 180 | F02 | 10 µM BAM15 | 02:32:07 | 105.28 |
| 14 | 181 | F02 | 10 µM BAM15 | 02:32:21 | 92.90 |
| 14 | 182 | F02 | 10 µM BAM15 | 02:32:35 | 85.21 |
| 14 | 183 | F02 | 10 µM BAM15 | 02:32:50 | 78.67 |
| 14 | 184 | F02 | 10 µM BAM15 | 02:33:04 | 72.86 |
| 14 | 185 | F02 | 10 µM BAM15 | 02:33:18 | 67.37 |
| 14 | 186 | F02 | 10 µM BAM15 | 02:33:32 | 62.39 |
| 14 | 187 | F02 | 10 µM BAM15 | 02:33:46 | 57.62 |
| 14 | 188 | F02 | 10 µM BAM15 | 02:34:01 | 53.28 |
| 14 | 189 | F02 | 10 µM BAM15 | 02:34:15 | 49.16 |
| 14 | 190 | F02 | 10 µM BAM15 | 02:34:29 | 45.28 |
| 14 | 191 | F02 | 10 µM BAM15 | 02:34:43 | 41.72 |
| 14 | 192 | F02 | 10 µM BAM15 | 02:34:57 | 38.39 |
| 14 | 193 | F02 | 10 µM BAM15 | 02:35:12 | 35.52 |
| 15 | 194 | F02 | 10 µM BAM15 | 02:40:46 | 105.57 |
| 15 | 195 | F02 | 10 µM BAM15 | 02:41:00 | 93.44 |
| 15 | 196 | F02 | 10 µM BAM15 | 02:41:14 | 85.72 |
| 15 | 197 | F02 | 10 µM BAM15 | 02:41:28 | 79.30 |
| 15 | 198 | F02 | 10 µM BAM15 | 02:41:43 | 73.53 |
| 15 | 199 | F02 | 10 µM BAM15 | 02:41:57 | 68.24 |
| 15 | 200 | F02 | 10 µM BAM15 | 02:42:11 | 63.28 |
| 15 | 201 | F02 | 10 µM BAM15 | 02:42:25 | 58.59 |
| 15 | 202 | F02 | 10 µM BAM15 | 02:42:39 | 54.27 |
| 15 | 203 | F02 | 10 µM BAM15 | 02:42:53 | 50.21 |
| 15 | 204 | F02 | 10 µM BAM15 | 02:43:08 | 46.28 |
| 15 | 205 | F02 | 10 µM BAM15 | 02:43:22 | 42.82 |
| 15 | 206 | F02 | 10 µM BAM15 | 02:43:36 | 39.40 |
| 15 | 207 | F02 | 10 µM BAM15 | 02:43:50 | 36.46 |
| 16 | 208 | F02 | 10 µM BAM15 | 02:49:25 | 105.67 |
| 16 | 209 | F02 | 10 µM BAM15 | 02:49:40 | 93.72 |
| 16 | 210 | F02 | 10 µM BAM15 | 02:49:54 | 86.13 |
| 16 | 211 | F02 | 10 µM BAM15 | 02:50:08 | 79.77 |
| 16 | 212 | F02 | 10 µM BAM15 | 02:50:22 | 74.09 |
| 16 | 213 | F02 | 10 µM BAM15 | 02:50:36 | 68.74 |
| 16 | 214 | F02 | 10 µM BAM15 | 02:50:50 | 63.89 |
| 16 | 215 | F02 | 10 µM BAM15 | 02:51:05 | 59.24 |
| 16 | 216 | F02 | 10 µM BAM15 | 02:51:19 | 54.95 |
| 16 | 217 | F02 | 10 µM BAM15 | 02:51:33 | 50.91 |
| 16 | 218 | F02 | 10 µM BAM15 | 02:51:47 | 47.12 |
| 16 | 219 | F02 | 10 µM BAM15 | 02:52:01 | 43.59 |
| 16 | 220 | F02 | 10 µM BAM15 | 02:52:16 | 40.22 |
| 16 | 221 | F02 | 10 µM BAM15 | 02:52:30 | 37.21 |
| 1 | 0 | F03 | 10 µM BAM15 | 00:40:06 | 129.41 |
| 1 | 1 | F03 | 10 µM BAM15 | 00:40:20 | 123.31 |
| 1 | 2 | F03 | 10 µM BAM15 | 00:40:34 | 120.56 |
| 1 | 3 | F03 | 10 µM BAM15 | 00:40:48 | 118.39 |
| 1 | 4 | F03 | 10 µM BAM15 | 00:41:02 | 116.51 |
| 1 | 5 | F03 | 10 µM BAM15 | 00:41:17 | 114.87 |
| 1 | 6 | F03 | 10 µM BAM15 | 00:41:31 | 113.36 |
| 1 | 7 | F03 | 10 µM BAM15 | 00:41:45 | 111.93 |
| 1 | 8 | F03 | 10 µM BAM15 | 00:41:59 | 110.65 |
| 1 | 9 | F03 | 10 µM BAM15 | 00:42:13 | 109.42 |
| 1 | 10 | F03 | 10 µM BAM15 | 00:42:28 | 108.07 |
| 1 | 11 | F03 | 10 µM BAM15 | 00:42:42 | 106.89 |
| 1 | 12 | F03 | 10 µM BAM15 | 00:42:56 | 105.79 |
| 1 | 13 | F03 | 10 µM BAM15 | 00:43:10 | 104.72 |
| 2 | 14 | F03 | 10 µM BAM15 | 00:48:43 | 132.54 |
| 2 | 15 | F03 | 10 µM BAM15 | 00:48:57 | 127.48 |
| 2 | 16 | F03 | 10 µM BAM15 | 00:49:12 | 124.69 |
| 2 | 17 | F03 | 10 µM BAM15 | 00:49:26 | 122.38 |
| 2 | 18 | F03 | 10 µM BAM15 | 00:49:40 | 120.46 |
| 2 | 19 | F03 | 10 µM BAM15 | 00:49:54 | 118.61 |
| 2 | 20 | F03 | 10 µM BAM15 | 00:50:08 | 117.01 |
| 2 | 21 | F03 | 10 µM BAM15 | 00:50:22 | 115.38 |
| 2 | 22 | F03 | 10 µM BAM15 | 00:50:37 | 114.00 |
| 2 | 23 | F03 | 10 µM BAM15 | 00:50:51 | 112.61 |
| 2 | 24 | F03 | 10 µM BAM15 | 00:51:05 | 111.27 |
| 2 | 25 | F03 | 10 µM BAM15 | 00:51:19 | 110.17 |
| 2 | 26 | F03 | 10 µM BAM15 | 00:51:33 | 108.88 |
| 2 | 27 | F03 | 10 µM BAM15 | 00:51:48 | 107.72 |
| 3 | 28 | F03 | 10 µM BAM15 | 00:57:21 | 133.96 |
| 3 | 29 | F03 | 10 µM BAM15 | 00:57:35 | 129.65 |
| 3 | 30 | F03 | 10 µM BAM15 | 00:57:49 | 126.98 |
| 3 | 31 | F03 | 10 µM BAM15 | 00:58:03 | 124.82 |
| 3 | 32 | F03 | 10 µM BAM15 | 00:58:17 | 122.86 |
| 3 | 33 | F03 | 10 µM BAM15 | 00:58:31 | 121.07 |
| 3 | 34 | F03 | 10 µM BAM15 | 00:58:46 | 119.47 |
| 3 | 35 | F03 | 10 µM BAM15 | 00:59:00 | 117.90 |
| 3 | 36 | F03 | 10 µM BAM15 | 00:59:14 | 116.43 |
| 3 | 37 | F03 | 10 µM BAM15 | 00:59:28 | 115.11 |
| 3 | 38 | F03 | 10 µM BAM15 | 00:59:42 | 113.70 |
| 3 | 39 | F03 | 10 µM BAM15 | 00:59:56 | 112.54 |
| 3 | 40 | F03 | 10 µM BAM15 | 01:00:11 | 111.27 |
| 3 | 41 | F03 | 10 µM BAM15 | 01:00:25 | 110.11 |
| 4 | 42 | F03 | 10 µM BAM15 | 01:06:04 | 117.86 |
| 4 | 43 | F03 | 10 µM BAM15 | 01:06:18 | 102.56 |
| 4 | 44 | F03 | 10 µM BAM15 | 01:06:33 | 93.10 |
| 4 | 45 | F03 | 10 µM BAM15 | 01:06:47 | 85.46 |
| 4 | 46 | F03 | 10 µM BAM15 | 01:07:01 | 78.83 |
| 4 | 47 | F03 | 10 µM BAM15 | 01:07:15 | 72.53 |
| 4 | 48 | F03 | 10 µM BAM15 | 01:07:29 | 66.87 |
| 4 | 49 | F03 | 10 µM BAM15 | 01:07:43 | 61.50 |
| 4 | 50 | F03 | 10 µM BAM15 | 01:07:58 | 56.63 |
| 4 | 51 | F03 | 10 µM BAM15 | 01:08:12 | 51.97 |
| 4 | 52 | F03 | 10 µM BAM15 | 01:08:26 | 47.53 |
| 4 | 53 | F03 | 10 µM BAM15 | 01:08:40 | 43.49 |
| 4 | 54 | F03 | 10 µM BAM15 | 01:08:54 | 39.60 |
| 4 | 55 | F03 | 10 µM BAM15 | 01:09:09 | 36.27 |
| 5 | 56 | F03 | 10 µM BAM15 | 01:14:42 | 108.29 |
| 5 | 57 | F03 | 10 µM BAM15 | 01:14:56 | 91.58 |
| 5 | 58 | F03 | 10 µM BAM15 | 01:15:10 | 81.91 |
| 5 | 59 | F03 | 10 µM BAM15 | 01:15:25 | 74.26 |
| 5 | 60 | F03 | 10 µM BAM15 | 01:15:39 | 67.44 |
| 5 | 61 | F03 | 10 µM BAM15 | 01:15:53 | 61.17 |
| 5 | 62 | F03 | 10 µM BAM15 | 01:16:07 | 55.51 |
| 5 | 63 | F03 | 10 µM BAM15 | 01:16:21 | 50.19 |
| 5 | 64 | F03 | 10 µM BAM15 | 01:16:35 | 45.40 |
| 5 | 65 | F03 | 10 µM BAM15 | 01:16:50 | 40.86 |
| 5 | 66 | F03 | 10 µM BAM15 | 01:17:04 | 36.81 |
| 5 | 67 | F03 | 10 µM BAM15 | 01:17:18 | 33.37 |
| 5 | 68 | F03 | 10 µM BAM15 | 01:17:32 | 30.63 |
| 5 | 69 | F03 | 10 µM BAM15 | 01:17:46 | 28.59 |
| 6 | 70 | F03 | 10 µM BAM15 | 01:23:20 | 104.68 |
| 6 | 71 | F03 | 10 µM BAM15 | 01:23:34 | 87.66 |
| 6 | 72 | F03 | 10 µM BAM15 | 01:23:48 | 77.97 |
| 6 | 73 | F03 | 10 µM BAM15 | 01:24:02 | 70.10 |
| 6 | 74 | F03 | 10 µM BAM15 | 01:24:16 | 63.25 |
| 6 | 75 | F03 | 10 µM BAM15 | 01:24:30 | 56.87 |
| 6 | 76 | F03 | 10 µM BAM15 | 01:24:44 | 51.12 |
| 6 | 77 | F03 | 10 µM BAM15 | 01:24:59 | 45.68 |
| 6 | 78 | F03 | 10 µM BAM15 | 01:25:13 | 40.83 |
| 6 | 79 | F03 | 10 µM BAM15 | 01:25:27 | 36.45 |
| 6 | 80 | F03 | 10 µM BAM15 | 01:25:41 | 32.77 |
| 6 | 81 | F03 | 10 µM BAM15 | 01:25:55 | 30.01 |
| 6 | 82 | F03 | 10 µM BAM15 | 01:26:10 | 27.85 |
| 6 | 83 | F03 | 10 µM BAM15 | 01:26:24 | 26.31 |
| 7 | 84 | F03 | 10 µM BAM15 | 01:31:58 | 102.36 |
| 7 | 85 | F03 | 10 µM BAM15 | 01:32:12 | 85.49 |
| 7 | 86 | F03 | 10 µM BAM15 | 01:32:26 | 75.59 |
| 7 | 87 | F03 | 10 µM BAM15 | 01:32:40 | 67.75 |
| 7 | 88 | F03 | 10 µM BAM15 | 01:32:54 | 60.68 |
| 7 | 89 | F03 | 10 µM BAM15 | 01:33:08 | 54.24 |
| 7 | 90 | F03 | 10 µM BAM15 | 01:33:23 | 48.34 |
| 7 | 91 | F03 | 10 µM BAM15 | 01:33:37 | 42.87 |
| 7 | 92 | F03 | 10 µM BAM15 | 01:33:51 | 38.00 |
| 7 | 93 | F03 | 10 µM BAM15 | 01:34:05 | 33.86 |
| 7 | 94 | F03 | 10 µM BAM15 | 01:34:19 | 30.55 |
| 7 | 95 | F03 | 10 µM BAM15 | 01:34:33 | 28.25 |
| 7 | 96 | F03 | 10 µM BAM15 | 01:34:48 | 26.52 |
| 7 | 97 | F03 | 10 µM BAM15 | 01:35:02 | 25.24 |
| 8 | 98 | F03 | 10 µM BAM15 | 01:40:36 | 100.92 |
| 8 | 99 | F03 | 10 µM BAM15 | 01:40:50 | 84.11 |
| 8 | 100 | F03 | 10 µM BAM15 | 01:41:04 | 74.23 |
| 8 | 101 | F03 | 10 µM BAM15 | 01:41:18 | 66.18 |
| 8 | 102 | F03 | 10 µM BAM15 | 01:41:32 | 59.16 |
| 8 | 103 | F03 | 10 µM BAM15 | 01:41:46 | 52.67 |
| 8 | 104 | F03 | 10 µM BAM15 | 01:42:00 | 46.75 |
| 8 | 105 | F03 | 10 µM BAM15 | 01:42:15 | 41.24 |
| 8 | 106 | F03 | 10 µM BAM15 | 01:42:29 | 36.38 |
| 8 | 107 | F03 | 10 µM BAM15 | 01:42:43 | 32.43 |
| 8 | 108 | F03 | 10 µM BAM15 | 01:42:57 | 29.42 |
| 8 | 109 | F03 | 10 µM BAM15 | 01:43:11 | 27.33 |
| 8 | 110 | F03 | 10 µM BAM15 | 01:43:26 | 25.76 |
| 8 | 111 | F03 | 10 µM BAM15 | 01:43:40 | 24.66 |
| 9 | 112 | F03 | 10 µM BAM15 | 01:49:14 | 100.19 |
| 9 | 113 | F03 | 10 µM BAM15 | 01:49:28 | 83.34 |
| 9 | 114 | F03 | 10 µM BAM15 | 01:49:42 | 73.39 |
| 9 | 115 | F03 | 10 µM BAM15 | 01:49:56 | 65.33 |
| 9 | 116 | F03 | 10 µM BAM15 | 01:50:10 | 58.23 |
| 9 | 117 | F03 | 10 µM BAM15 | 01:50:24 | 51.69 |
| 9 | 118 | F03 | 10 µM BAM15 | 01:50:38 | 45.77 |
| 9 | 119 | F03 | 10 µM BAM15 | 01:50:53 | 40.21 |
| 9 | 120 | F03 | 10 µM BAM15 | 01:51:07 | 35.45 |
| 9 | 121 | F03 | 10 µM BAM15 | 01:51:21 | 31.60 |
| 9 | 122 | F03 | 10 µM BAM15 | 01:51:35 | 28.81 |
| 9 | 123 | F03 | 10 µM BAM15 | 01:51:50 | 26.81 |
| 9 | 124 | F03 | 10 µM BAM15 | 01:52:04 | 25.38 |
| 9 | 125 | F03 | 10 µM BAM15 | 01:52:18 | 24.27 |
| 10 | 126 | F03 | 10 µM BAM15 | 01:57:52 | 99.78 |
| 10 | 127 | F03 | 10 µM BAM15 | 01:58:06 | 82.95 |
| 10 | 128 | F03 | 10 µM BAM15 | 01:58:20 | 72.88 |
| 10 | 129 | F03 | 10 µM BAM15 | 01:58:34 | 64.86 |
| 10 | 130 | F03 | 10 µM BAM15 | 01:58:48 | 57.69 |
| 10 | 131 | F03 | 10 µM BAM15 | 01:59:03 | 51.06 |
| 10 | 132 | F03 | 10 µM BAM15 | 01:59:17 | 45.05 |
| 10 | 133 | F03 | 10 µM BAM15 | 01:59:31 | 39.49 |
| 10 | 134 | F03 | 10 µM BAM15 | 01:59:45 | 34.85 |
| 10 | 135 | F03 | 10 µM BAM15 | 01:59:59 | 31.07 |
| 10 | 136 | F03 | 10 µM BAM15 | 02:00:14 | 28.41 |
| 10 | 137 | F03 | 10 µM BAM15 | 02:00:28 | 26.52 |
| 10 | 138 | F03 | 10 µM BAM15 | 02:00:42 | 25.14 |
| 10 | 139 | F03 | 10 µM BAM15 | 02:00:56 | 24.12 |
| 11 | 140 | F03 | 10 µM BAM15 | 02:06:30 | 99.64 |
| 11 | 141 | F03 | 10 µM BAM15 | 02:06:44 | 83.11 |
| 11 | 142 | F03 | 10 µM BAM15 | 02:06:58 | 73.10 |
| 11 | 143 | F03 | 10 µM BAM15 | 02:07:12 | 64.98 |
| 11 | 144 | F03 | 10 µM BAM15 | 02:07:26 | 57.83 |
| 11 | 145 | F03 | 10 µM BAM15 | 02:07:41 | 51.16 |
| 11 | 146 | F03 | 10 µM BAM15 | 02:07:55 | 45.13 |
| 11 | 147 | F03 | 10 µM BAM15 | 02:08:09 | 39.57 |
| 11 | 148 | F03 | 10 µM BAM15 | 02:08:23 | 34.80 |
| 11 | 149 | F03 | 10 µM BAM15 | 02:08:37 | 31.02 |
| 11 | 150 | F03 | 10 µM BAM15 | 02:08:52 | 28.29 |
| 11 | 151 | F03 | 10 µM BAM15 | 02:09:06 | 26.34 |
| 11 | 152 | F03 | 10 µM BAM15 | 02:09:20 | 25.00 |
| 11 | 153 | F03 | 10 µM BAM15 | 02:09:34 | 23.90 |
| 12 | 154 | F03 | 10 µM BAM15 | 02:15:08 | 101.38 |
| 12 | 155 | F03 | 10 µM BAM15 | 02:15:22 | 85.83 |
| 12 | 156 | F03 | 10 µM BAM15 | 02:15:37 | 75.24 |
| 12 | 157 | F03 | 10 µM BAM15 | 02:15:53 | 66.32 |
| 12 | 158 | F03 | 10 µM BAM15 | 02:16:08 | 59.03 |
| 12 | 159 | F03 | 10 µM BAM15 | 02:16:22 | 52.57 |
| 12 | 160 | F03 | 10 µM BAM15 | 02:16:36 | 46.49 |
| 12 | 161 | F03 | 10 µM BAM15 | 02:16:50 | 40.85 |
| 12 | 162 | F03 | 10 µM BAM15 | 02:17:04 | 35.94 |
| 12 | 163 | F03 | 10 µM BAM15 | 02:17:18 | 31.88 |
| 12 | 164 | F03 | 10 µM BAM15 | 02:17:33 | 28.92 |
| 12 | 165 | F03 | 10 µM BAM15 | 02:17:47 | 26.77 |
| 12 | 166 | F03 | 10 µM BAM15 | 02:18:01 | 25.21 |
| 13 | 167 | F03 | 10 µM BAM15 | 02:23:39 | 97.45 |
| 13 | 168 | F03 | 10 µM BAM15 | 02:23:54 | 84.44 |
| 13 | 169 | F03 | 10 µM BAM15 | 02:24:08 | 75.19 |
| 13 | 170 | F03 | 10 µM BAM15 | 02:24:22 | 67.37 |
| 13 | 171 | F03 | 10 µM BAM15 | 02:24:36 | 60.30 |
| 13 | 172 | F03 | 10 µM BAM15 | 02:24:51 | 53.28 |
| 13 | 173 | F03 | 10 µM BAM15 | 02:25:07 | 46.62 |
| 13 | 174 | F03 | 10 µM BAM15 | 02:25:22 | 41.15 |
| 13 | 175 | F03 | 10 µM BAM15 | 02:25:36 | 36.47 |
| 13 | 176 | F03 | 10 µM BAM15 | 02:25:50 | 32.48 |
| 13 | 177 | F03 | 10 µM BAM15 | 02:26:04 | 29.44 |
| 13 | 178 | F03 | 10 µM BAM15 | 02:26:18 | 27.13 |
| 13 | 179 | F03 | 10 µM BAM15 | 02:26:33 | 25.44 |
| 14 | 180 | F03 | 10 µM BAM15 | 02:32:07 | 102.54 |
| 14 | 181 | F03 | 10 µM BAM15 | 02:32:21 | 87.59 |
| 14 | 182 | F03 | 10 µM BAM15 | 02:32:35 | 78.01 |
| 14 | 183 | F03 | 10 µM BAM15 | 02:32:50 | 70.11 |
| 14 | 184 | F03 | 10 µM BAM15 | 02:33:04 | 62.99 |
| 14 | 185 | F03 | 10 µM BAM15 | 02:33:18 | 56.36 |
| 14 | 186 | F03 | 10 µM BAM15 | 02:33:32 | 50.34 |
| 14 | 187 | F03 | 10 µM BAM15 | 02:33:46 | 44.64 |
| 14 | 188 | F03 | 10 µM BAM15 | 02:34:01 | 39.65 |
| 14 | 189 | F03 | 10 µM BAM15 | 02:34:15 | 35.30 |
| 14 | 190 | F03 | 10 µM BAM15 | 02:34:29 | 31.64 |
| 14 | 191 | F03 | 10 µM BAM15 | 02:34:43 | 28.78 |
| 14 | 192 | F03 | 10 µM BAM15 | 02:34:57 | 26.60 |
| 14 | 193 | F03 | 10 µM BAM15 | 02:35:12 | 25.10 |
| 15 | 194 | F03 | 10 µM BAM15 | 02:40:46 | 102.75 |
| 15 | 195 | F03 | 10 µM BAM15 | 02:41:00 | 87.97 |
| 15 | 196 | F03 | 10 µM BAM15 | 02:41:14 | 78.45 |
| 15 | 197 | F03 | 10 µM BAM15 | 02:41:28 | 70.61 |
| 15 | 198 | F03 | 10 µM BAM15 | 02:41:43 | 63.57 |
| 15 | 199 | F03 | 10 µM BAM15 | 02:41:57 | 57.19 |
| 15 | 200 | F03 | 10 µM BAM15 | 02:42:11 | 51.15 |
| 15 | 201 | F03 | 10 µM BAM15 | 02:42:25 | 45.52 |
| 15 | 202 | F03 | 10 µM BAM15 | 02:42:39 | 40.50 |
| 15 | 203 | F03 | 10 µM BAM15 | 02:42:53 | 36.08 |
| 15 | 204 | F03 | 10 µM BAM15 | 02:43:08 | 32.26 |
| 15 | 205 | F03 | 10 µM BAM15 | 02:43:22 | 29.37 |
| 15 | 206 | F03 | 10 µM BAM15 | 02:43:36 | 26.98 |
| 15 | 207 | F03 | 10 µM BAM15 | 02:43:50 | 25.33 |
| 16 | 208 | F03 | 10 µM BAM15 | 02:49:25 | 103.02 |
| 16 | 209 | F03 | 10 µM BAM15 | 02:49:40 | 88.33 |
| 16 | 210 | F03 | 10 µM BAM15 | 02:49:54 | 78.89 |
| 16 | 211 | F03 | 10 µM BAM15 | 02:50:08 | 71.09 |
| 16 | 212 | F03 | 10 µM BAM15 | 02:50:22 | 64.14 |
| 16 | 213 | F03 | 10 µM BAM15 | 02:50:36 | 57.68 |
| 16 | 214 | F03 | 10 µM BAM15 | 02:50:50 | 51.77 |
| 16 | 215 | F03 | 10 µM BAM15 | 02:51:05 | 46.16 |
| 16 | 216 | F03 | 10 µM BAM15 | 02:51:19 | 41.11 |
| 16 | 217 | F03 | 10 µM BAM15 | 02:51:33 | 36.64 |
| 16 | 218 | F03 | 10 µM BAM15 | 02:51:47 | 32.82 |
| 16 | 219 | F03 | 10 µM BAM15 | 02:52:01 | 29.79 |
| 16 | 220 | F03 | 10 µM BAM15 | 02:52:16 | 27.32 |
| 16 | 221 | F03 | 10 µM BAM15 | 02:52:30 | 25.52 |
| 1 | 0 | F04 | 10 µM FCCP | 00:40:06 | 126.70 |
| 1 | 1 | F04 | 10 µM FCCP | 00:40:20 | 121.04 |
| 1 | 2 | F04 | 10 µM FCCP | 00:40:34 | 118.55 |
| 1 | 3 | F04 | 10 µM FCCP | 00:40:48 | 116.57 |
| 1 | 4 | F04 | 10 µM FCCP | 00:41:02 | 114.94 |
| 1 | 5 | F04 | 10 µM FCCP | 00:41:17 | 113.40 |
| 1 | 6 | F04 | 10 µM FCCP | 00:41:31 | 111.94 |
| 1 | 7 | F04 | 10 µM FCCP | 00:41:45 | 110.66 |
| 1 | 8 | F04 | 10 µM FCCP | 00:41:59 | 109.38 |
| 1 | 9 | F04 | 10 µM FCCP | 00:42:13 | 108.22 |
| 1 | 10 | F04 | 10 µM FCCP | 00:42:28 | 107.00 |
| 1 | 11 | F04 | 10 µM FCCP | 00:42:42 | 105.89 |
| 1 | 12 | F04 | 10 µM FCCP | 00:42:56 | 104.77 |
| 1 | 13 | F04 | 10 µM FCCP | 00:43:10 | 103.83 |
| 2 | 14 | F04 | 10 µM FCCP | 00:48:43 | 130.12 |
| 2 | 15 | F04 | 10 µM FCCP | 00:48:57 | 124.81 |
| 2 | 16 | F04 | 10 µM FCCP | 00:49:12 | 121.99 |
| 2 | 17 | F04 | 10 µM FCCP | 00:49:26 | 119.86 |
| 2 | 18 | F04 | 10 µM FCCP | 00:49:40 | 118.05 |
| 2 | 19 | F04 | 10 µM FCCP | 00:49:54 | 116.33 |
| 2 | 20 | F04 | 10 µM FCCP | 00:50:08 | 114.79 |
| 2 | 21 | F04 | 10 µM FCCP | 00:50:22 | 113.28 |
| 2 | 22 | F04 | 10 µM FCCP | 00:50:37 | 111.94 |
| 2 | 23 | F04 | 10 µM FCCP | 00:50:51 | 110.66 |
| 2 | 24 | F04 | 10 µM FCCP | 00:51:05 | 109.38 |
| 2 | 25 | F04 | 10 µM FCCP | 00:51:19 | 108.20 |
| 2 | 26 | F04 | 10 µM FCCP | 00:51:33 | 107.00 |
| 2 | 27 | F04 | 10 µM FCCP | 00:51:48 | 105.97 |
| 3 | 28 | F04 | 10 µM FCCP | 00:57:21 | 130.83 |
| 3 | 29 | F04 | 10 µM FCCP | 00:57:35 | 126.10 |
| 3 | 30 | F04 | 10 µM FCCP | 00:57:49 | 123.51 |
| 3 | 31 | F04 | 10 µM FCCP | 00:58:03 | 121.44 |
| 3 | 32 | F04 | 10 µM FCCP | 00:58:17 | 119.68 |
| 3 | 33 | F04 | 10 µM FCCP | 00:58:31 | 117.87 |
| 3 | 34 | F04 | 10 µM FCCP | 00:58:46 | 116.46 |
| 3 | 35 | F04 | 10 µM FCCP | 00:59:00 | 114.98 |
| 3 | 36 | F04 | 10 µM FCCP | 00:59:14 | 113.50 |
| 3 | 37 | F04 | 10 µM FCCP | 00:59:28 | 112.24 |
| 3 | 38 | F04 | 10 µM FCCP | 00:59:42 | 110.95 |
| 3 | 39 | F04 | 10 µM FCCP | 00:59:56 | 109.80 |
| 3 | 40 | F04 | 10 µM FCCP | 01:00:11 | 108.68 |
| 3 | 41 | F04 | 10 µM FCCP | 01:00:25 | 107.48 |
| 4 | 42 | F04 | 10 µM FCCP | 01:06:04 | 131.42 |
| 4 | 43 | F04 | 10 µM FCCP | 01:06:18 | 126.35 |
| 4 | 44 | F04 | 10 µM FCCP | 01:06:33 | 123.39 |
| 4 | 45 | F04 | 10 µM FCCP | 01:06:47 | 121.12 |
| 4 | 46 | F04 | 10 µM FCCP | 01:07:01 | 119.14 |
| 4 | 47 | F04 | 10 µM FCCP | 01:07:15 | 117.20 |
| 4 | 48 | F04 | 10 µM FCCP | 01:07:29 | 115.42 |
| 4 | 49 | F04 | 10 µM FCCP | 01:07:43 | 113.76 |
| 4 | 50 | F04 | 10 µM FCCP | 01:07:58 | 112.15 |
| 4 | 51 | F04 | 10 µM FCCP | 01:08:12 | 110.55 |
| 4 | 52 | F04 | 10 µM FCCP | 01:08:26 | 108.94 |
| 4 | 53 | F04 | 10 µM FCCP | 01:08:40 | 107.46 |
| 4 | 54 | F04 | 10 µM FCCP | 01:08:54 | 105.84 |
| 4 | 55 | F04 | 10 µM FCCP | 01:09:09 | 104.39 |
| 5 | 56 | F04 | 10 µM FCCP | 01:14:42 | 129.54 |
| 5 | 57 | F04 | 10 µM FCCP | 01:14:56 | 123.88 |
| 5 | 58 | F04 | 10 µM FCCP | 01:15:10 | 120.78 |
| 5 | 59 | F04 | 10 µM FCCP | 01:15:25 | 118.41 |
| 5 | 60 | F04 | 10 µM FCCP | 01:15:39 | 116.30 |
| 5 | 61 | F04 | 10 µM FCCP | 01:15:53 | 114.20 |
| 5 | 62 | F04 | 10 µM FCCP | 01:16:07 | 112.33 |
| 5 | 63 | F04 | 10 µM FCCP | 01:16:21 | 110.46 |
| 5 | 64 | F04 | 10 µM FCCP | 01:16:35 | 108.78 |
| 5 | 65 | F04 | 10 µM FCCP | 01:16:50 | 106.98 |
| 5 | 66 | F04 | 10 µM FCCP | 01:17:04 | 105.23 |
| 5 | 67 | F04 | 10 µM FCCP | 01:17:18 | 103.45 |
| 5 | 68 | F04 | 10 µM FCCP | 01:17:32 | 101.70 |
| 5 | 69 | F04 | 10 µM FCCP | 01:17:46 | 100.07 |
| 6 | 70 | F04 | 10 µM FCCP | 01:23:20 | 129.05 |
| 6 | 71 | F04 | 10 µM FCCP | 01:23:34 | 123.18 |
| 6 | 72 | F04 | 10 µM FCCP | 01:23:48 | 120.15 |
| 6 | 73 | F04 | 10 µM FCCP | 01:24:02 | 117.62 |
| 6 | 74 | F04 | 10 µM FCCP | 01:24:16 | 115.43 |
| 6 | 75 | F04 | 10 µM FCCP | 01:24:30 | 113.35 |
| 6 | 76 | F04 | 10 µM FCCP | 01:24:44 | 111.42 |
| 6 | 77 | F04 | 10 µM FCCP | 01:24:59 | 109.39 |
| 6 | 78 | F04 | 10 µM FCCP | 01:25:13 | 107.62 |
| 6 | 79 | F04 | 10 µM FCCP | 01:25:27 | 105.83 |
| 6 | 80 | F04 | 10 µM FCCP | 01:25:41 | 103.98 |
| 6 | 81 | F04 | 10 µM FCCP | 01:25:55 | 102.26 |
| 6 | 82 | F04 | 10 µM FCCP | 01:26:10 | 100.45 |
| 6 | 83 | F04 | 10 µM FCCP | 01:26:24 | 98.71 |
| 7 | 84 | F04 | 10 µM FCCP | 01:31:58 | 128.88 |
| 7 | 85 | F04 | 10 µM FCCP | 01:32:12 | 123.17 |
| 7 | 86 | F04 | 10 µM FCCP | 01:32:26 | 120.03 |
| 7 | 87 | F04 | 10 µM FCCP | 01:32:40 | 117.59 |
| 7 | 88 | F04 | 10 µM FCCP | 01:32:54 | 115.35 |
| 7 | 89 | F04 | 10 µM FCCP | 01:33:08 | 113.32 |
| 7 | 90 | F04 | 10 µM FCCP | 01:33:23 | 111.33 |
| 7 | 91 | F04 | 10 µM FCCP | 01:33:37 | 109.37 |
| 7 | 92 | F04 | 10 µM FCCP | 01:33:51 | 107.58 |
| 7 | 93 | F04 | 10 µM FCCP | 01:34:05 | 105.83 |
| 7 | 94 | F04 | 10 µM FCCP | 01:34:19 | 104.04 |
| 7 | 95 | F04 | 10 µM FCCP | 01:34:33 | 102.36 |
| 7 | 96 | F04 | 10 µM FCCP | 01:34:48 | 100.65 |
| 7 | 97 | F04 | 10 µM FCCP | 01:35:02 | 98.96 |
| 8 | 98 | F04 | 10 µM FCCP | 01:40:36 | 129.20 |
| 8 | 99 | F04 | 10 µM FCCP | 01:40:50 | 123.53 |
| 8 | 100 | F04 | 10 µM FCCP | 01:41:04 | 120.46 |
| 8 | 101 | F04 | 10 µM FCCP | 01:41:18 | 118.03 |
| 8 | 102 | F04 | 10 µM FCCP | 01:41:32 | 115.87 |
| 8 | 103 | F04 | 10 µM FCCP | 01:41:46 | 113.84 |
| 8 | 104 | F04 | 10 µM FCCP | 01:42:00 | 111.95 |
| 8 | 105 | F04 | 10 µM FCCP | 01:42:15 | 110.12 |
| 8 | 106 | F04 | 10 µM FCCP | 01:42:29 | 108.32 |
| 8 | 107 | F04 | 10 µM FCCP | 01:42:43 | 106.52 |
| 8 | 108 | F04 | 10 µM FCCP | 01:42:57 | 104.87 |
| 8 | 109 | F04 | 10 µM FCCP | 01:43:11 | 103.24 |
| 8 | 110 | F04 | 10 µM FCCP | 01:43:26 | 101.55 |
| 8 | 111 | F04 | 10 µM FCCP | 01:43:40 | 100.02 |
| 9 | 112 | F04 | 10 µM FCCP | 01:49:14 | 129.83 |
| 9 | 113 | F04 | 10 µM FCCP | 01:49:28 | 124.31 |
| 9 | 114 | F04 | 10 µM FCCP | 01:49:42 | 121.34 |
| 9 | 115 | F04 | 10 µM FCCP | 01:49:56 | 118.96 |
| 9 | 116 | F04 | 10 µM FCCP | 01:50:10 | 116.80 |
| 9 | 117 | F04 | 10 µM FCCP | 01:50:24 | 114.91 |
| 9 | 118 | F04 | 10 µM FCCP | 01:50:38 | 113.03 |
| 9 | 119 | F04 | 10 µM FCCP | 01:50:53 | 111.26 |
| 9 | 120 | F04 | 10 µM FCCP | 01:51:07 | 109.48 |
| 9 | 121 | F04 | 10 µM FCCP | 01:51:21 | 107.84 |
| 9 | 122 | F04 | 10 µM FCCP | 01:51:35 | 106.26 |
| 9 | 123 | F04 | 10 µM FCCP | 01:51:50 | 104.70 |
| 9 | 124 | F04 | 10 µM FCCP | 01:52:04 | 103.13 |
| 9 | 125 | F04 | 10 µM FCCP | 01:52:18 | 101.63 |
| 10 | 126 | F04 | 10 µM FCCP | 01:57:52 | 130.25 |
| 10 | 127 | F04 | 10 µM FCCP | 01:58:06 | 125.31 |
| 10 | 128 | F04 | 10 µM FCCP | 01:58:20 | 122.47 |
| 10 | 129 | F04 | 10 µM FCCP | 01:58:34 | 120.25 |
| 10 | 130 | F04 | 10 µM FCCP | 01:58:48 | 118.20 |
| 10 | 131 | F04 | 10 µM FCCP | 01:59:03 | 116.23 |
| 10 | 132 | F04 | 10 µM FCCP | 01:59:17 | 114.40 |
| 10 | 133 | F04 | 10 µM FCCP | 01:59:31 | 112.76 |
| 10 | 134 | F04 | 10 µM FCCP | 01:59:45 | 111.18 |
| 10 | 135 | F04 | 10 µM FCCP | 01:59:59 | 109.55 |
| 10 | 136 | F04 | 10 µM FCCP | 02:00:14 | 108.06 |
| 10 | 137 | F04 | 10 µM FCCP | 02:00:28 | 106.52 |
| 10 | 138 | F04 | 10 µM FCCP | 02:00:42 | 105.02 |
| 10 | 139 | F04 | 10 µM FCCP | 02:00:56 | 103.67 |
| 11 | 140 | F04 | 10 µM FCCP | 02:06:30 | 132.15 |
| 11 | 141 | F04 | 10 µM FCCP | 02:06:44 | 127.71 |
| 11 | 142 | F04 | 10 µM FCCP | 02:06:58 | 125.08 |
| 11 | 143 | F04 | 10 µM FCCP | 02:07:12 | 122.95 |
| 11 | 144 | F04 | 10 µM FCCP | 02:07:26 | 121.02 |
| 11 | 145 | F04 | 10 µM FCCP | 02:07:41 | 119.14 |
| 11 | 146 | F04 | 10 µM FCCP | 02:07:55 | 117.46 |
| 11 | 147 | F04 | 10 µM FCCP | 02:08:09 | 115.78 |
| 11 | 148 | F04 | 10 µM FCCP | 02:08:23 | 114.20 |
| 11 | 149 | F04 | 10 µM FCCP | 02:08:37 | 112.60 |
| 11 | 150 | F04 | 10 µM FCCP | 02:08:52 | 111.13 |
| 11 | 151 | F04 | 10 µM FCCP | 02:09:06 | 109.67 |
| 11 | 152 | F04 | 10 µM FCCP | 02:09:20 | 108.32 |
| 11 | 153 | F04 | 10 µM FCCP | 02:09:34 | 106.89 |
| 12 | 154 | F04 | 10 µM FCCP | 02:15:08 | 133.21 |
| 12 | 155 | F04 | 10 µM FCCP | 02:15:22 | 129.03 |
| 12 | 156 | F04 | 10 µM FCCP | 02:15:37 | 126.46 |
| 12 | 157 | F04 | 10 µM FCCP | 02:15:53 | 124.08 |
| 12 | 158 | F04 | 10 µM FCCP | 02:16:08 | 122.18 |
| 12 | 159 | F04 | 10 µM FCCP | 02:16:22 | 120.51 |
| 12 | 160 | F04 | 10 µM FCCP | 02:16:36 | 118.79 |
| 12 | 161 | F04 | 10 µM FCCP | 02:16:50 | 117.24 |
| 12 | 162 | F04 | 10 µM FCCP | 02:17:04 | 115.74 |
| 12 | 163 | F04 | 10 µM FCCP | 02:17:18 | 114.24 |
| 12 | 164 | F04 | 10 µM FCCP | 02:17:33 | 112.86 |
| 12 | 165 | F04 | 10 µM FCCP | 02:17:47 | 111.43 |
| 12 | 166 | F04 | 10 µM FCCP | 02:18:01 | 110.04 |
| 13 | 167 | F04 | 10 µM FCCP | 02:23:39 | 132.63 |
| 13 | 168 | F04 | 10 µM FCCP | 02:23:54 | 129.39 |
| 13 | 169 | F04 | 10 µM FCCP | 02:24:08 | 127.01 |
| 13 | 170 | F04 | 10 µM FCCP | 02:24:22 | 125.07 |
| 13 | 171 | F04 | 10 µM FCCP | 02:24:36 | 123.33 |
| 13 | 172 | F04 | 10 µM FCCP | 02:24:51 | 121.54 |
| 13 | 173 | F04 | 10 µM FCCP | 02:25:07 | 119.78 |
| 13 | 174 | F04 | 10 µM FCCP | 02:25:22 | 118.20 |
| 13 | 175 | F04 | 10 µM FCCP | 02:25:36 | 116.79 |
| 13 | 176 | F04 | 10 µM FCCP | 02:25:50 | 115.39 |
| 13 | 177 | F04 | 10 µM FCCP | 02:26:04 | 113.99 |
| 13 | 178 | F04 | 10 µM FCCP | 02:26:18 | 112.70 |
| 13 | 179 | F04 | 10 µM FCCP | 02:26:33 | 111.38 |
| 14 | 180 | F04 | 10 µM FCCP | 02:32:07 | 134.63 |
| 14 | 181 | F04 | 10 µM FCCP | 02:32:21 | 130.66 |
| 14 | 182 | F04 | 10 µM FCCP | 02:32:35 | 128.41 |
| 14 | 183 | F04 | 10 µM FCCP | 02:32:50 | 126.49 |
| 14 | 184 | F04 | 10 µM FCCP | 02:33:04 | 124.78 |
| 14 | 185 | F04 | 10 µM FCCP | 02:33:18 | 123.09 |
| 14 | 186 | F04 | 10 µM FCCP | 02:33:32 | 121.54 |
| 14 | 187 | F04 | 10 µM FCCP | 02:33:46 | 120.03 |
| 14 | 188 | F04 | 10 µM FCCP | 02:34:01 | 118.64 |
| 14 | 189 | F04 | 10 µM FCCP | 02:34:15 | 117.22 |
| 14 | 190 | F04 | 10 µM FCCP | 02:34:29 | 115.93 |
| 14 | 191 | F04 | 10 µM FCCP | 02:34:43 | 114.62 |
| 14 | 192 | F04 | 10 µM FCCP | 02:34:57 | 113.30 |
| 14 | 193 | F04 | 10 µM FCCP | 02:35:12 | 112.12 |
| 15 | 194 | F04 | 10 µM FCCP | 02:40:46 | 135.15 |
| 15 | 195 | F04 | 10 µM FCCP | 02:41:00 | 131.37 |
| 15 | 196 | F04 | 10 µM FCCP | 02:41:14 | 129.08 |
| 15 | 197 | F04 | 10 µM FCCP | 02:41:28 | 127.17 |
| 15 | 198 | F04 | 10 µM FCCP | 02:41:43 | 125.50 |
| 15 | 199 | F04 | 10 µM FCCP | 02:41:57 | 123.94 |
| 15 | 200 | F04 | 10 µM FCCP | 02:42:11 | 122.45 |
| 15 | 201 | F04 | 10 µM FCCP | 02:42:25 | 120.98 |
| 15 | 202 | F04 | 10 µM FCCP | 02:42:39 | 119.57 |
| 15 | 203 | F04 | 10 µM FCCP | 02:42:53 | 118.22 |
| 15 | 204 | F04 | 10 µM FCCP | 02:43:08 | 116.87 |
| 15 | 205 | F04 | 10 µM FCCP | 02:43:22 | 115.71 |
| 15 | 206 | F04 | 10 µM FCCP | 02:43:36 | 114.37 |
| 15 | 207 | F04 | 10 µM FCCP | 02:43:50 | 113.17 |
| 16 | 208 | F04 | 10 µM FCCP | 02:49:25 | 135.55 |
| 16 | 209 | F04 | 10 µM FCCP | 02:49:40 | 131.94 |
| 16 | 210 | F04 | 10 µM FCCP | 02:49:54 | 129.73 |
| 16 | 211 | F04 | 10 µM FCCP | 02:50:08 | 127.81 |
| 16 | 212 | F04 | 10 µM FCCP | 02:50:22 | 126.19 |
| 16 | 213 | F04 | 10 µM FCCP | 02:50:36 | 124.53 |
| 16 | 214 | F04 | 10 µM FCCP | 02:50:50 | 123.16 |
| 16 | 215 | F04 | 10 µM FCCP | 02:51:05 | 121.68 |
| 16 | 216 | F04 | 10 µM FCCP | 02:51:19 | 120.34 |
| 16 | 217 | F04 | 10 µM FCCP | 02:51:33 | 118.99 |
| 16 | 218 | F04 | 10 µM FCCP | 02:51:47 | 117.77 |
| 16 | 219 | F04 | 10 µM FCCP | 02:52:01 | 116.53 |
| 16 | 220 | F04 | 10 µM FCCP | 02:52:16 | 115.31 |
| 16 | 221 | F04 | 10 µM FCCP | 02:52:30 | 114.05 |
| 1 | 0 | F05 | 10 µM FCCP | 00:40:06 | 128.55 |
| 1 | 1 | F05 | 10 µM FCCP | 00:40:20 | 122.19 |
| 1 | 2 | F05 | 10 µM FCCP | 00:40:34 | 119.49 |
| 1 | 3 | F05 | 10 µM FCCP | 00:40:48 | 117.41 |
| 1 | 4 | F05 | 10 µM FCCP | 00:41:02 | 115.64 |
| 1 | 5 | F05 | 10 µM FCCP | 00:41:17 | 114.10 |
| 1 | 6 | F05 | 10 µM FCCP | 00:41:31 | 112.64 |
| 1 | 7 | F05 | 10 µM FCCP | 00:41:45 | 111.33 |
| 1 | 8 | F05 | 10 µM FCCP | 00:41:59 | 110.02 |
| 1 | 9 | F05 | 10 µM FCCP | 00:42:13 | 108.84 |
| 1 | 10 | F05 | 10 µM FCCP | 00:42:28 | 107.60 |
| 1 | 11 | F05 | 10 µM FCCP | 00:42:42 | 106.46 |
| 1 | 12 | F05 | 10 µM FCCP | 00:42:56 | 105.39 |
| 1 | 13 | F05 | 10 µM FCCP | 00:43:10 | 104.36 |
| 2 | 14 | F05 | 10 µM FCCP | 00:48:43 | 131.47 |
| 2 | 15 | F05 | 10 µM FCCP | 00:48:57 | 126.49 |
| 2 | 16 | F05 | 10 µM FCCP | 00:49:12 | 123.88 |
| 2 | 17 | F05 | 10 µM FCCP | 00:49:26 | 121.80 |
| 2 | 18 | F05 | 10 µM FCCP | 00:49:40 | 120.02 |
| 2 | 19 | F05 | 10 µM FCCP | 00:49:54 | 118.31 |
| 2 | 20 | F05 | 10 µM FCCP | 00:50:08 | 116.82 |
| 2 | 21 | F05 | 10 µM FCCP | 00:50:22 | 115.34 |
| 2 | 22 | F05 | 10 µM FCCP | 00:50:37 | 113.95 |
| 2 | 23 | F05 | 10 µM FCCP | 00:50:51 | 112.67 |
| 2 | 24 | F05 | 10 µM FCCP | 00:51:05 | 111.35 |
| 2 | 25 | F05 | 10 µM FCCP | 00:51:19 | 110.17 |
| 2 | 26 | F05 | 10 µM FCCP | 00:51:33 | 108.94 |
| 2 | 27 | F05 | 10 µM FCCP | 00:51:48 | 107.87 |
| 3 | 28 | F05 | 10 µM FCCP | 00:57:21 | 132.45 |
| 3 | 29 | F05 | 10 µM FCCP | 00:57:35 | 127.95 |
| 3 | 30 | F05 | 10 µM FCCP | 00:57:49 | 125.40 |
| 3 | 31 | F05 | 10 µM FCCP | 00:58:03 | 123.45 |
| 3 | 32 | F05 | 10 µM FCCP | 00:58:17 | 121.68 |
| 3 | 33 | F05 | 10 µM FCCP | 00:58:31 | 120.06 |
| 3 | 34 | F05 | 10 µM FCCP | 00:58:46 | 118.46 |
| 3 | 35 | F05 | 10 µM FCCP | 00:59:00 | 117.02 |
| 3 | 36 | F05 | 10 µM FCCP | 00:59:14 | 115.57 |
| 3 | 37 | F05 | 10 µM FCCP | 00:59:28 | 114.34 |
| 3 | 38 | F05 | 10 µM FCCP | 00:59:42 | 113.01 |
| 3 | 39 | F05 | 10 µM FCCP | 00:59:56 | 111.82 |
| 3 | 40 | F05 | 10 µM FCCP | 01:00:11 | 110.71 |
| 3 | 41 | F05 | 10 µM FCCP | 01:00:25 | 109.57 |
| 4 | 42 | F05 | 10 µM FCCP | 01:06:04 | 132.96 |
| 4 | 43 | F05 | 10 µM FCCP | 01:06:18 | 127.38 |
| 4 | 44 | F05 | 10 µM FCCP | 01:06:33 | 124.53 |
| 4 | 45 | F05 | 10 µM FCCP | 01:06:47 | 122.21 |
| 4 | 46 | F05 | 10 µM FCCP | 01:07:01 | 120.31 |
| 4 | 47 | F05 | 10 µM FCCP | 01:07:15 | 118.50 |
| 4 | 48 | F05 | 10 µM FCCP | 01:07:29 | 116.69 |
| 4 | 49 | F05 | 10 µM FCCP | 01:07:43 | 115.08 |
| 4 | 50 | F05 | 10 µM FCCP | 01:07:58 | 113.46 |
| 4 | 51 | F05 | 10 µM FCCP | 01:08:12 | 112.06 |
| 4 | 52 | F05 | 10 µM FCCP | 01:08:26 | 110.50 |
| 4 | 53 | F05 | 10 µM FCCP | 01:08:40 | 109.01 |
| 4 | 54 | F05 | 10 µM FCCP | 01:08:54 | 107.60 |
| 4 | 55 | F05 | 10 µM FCCP | 01:09:09 | 106.14 |
| 5 | 56 | F05 | 10 µM FCCP | 01:14:42 | 131.27 |
| 5 | 57 | F05 | 10 µM FCCP | 01:14:56 | 125.35 |
| 5 | 58 | F05 | 10 µM FCCP | 01:15:10 | 122.25 |
| 5 | 59 | F05 | 10 µM FCCP | 01:15:25 | 119.90 |
| 5 | 60 | F05 | 10 µM FCCP | 01:15:39 | 117.85 |
| 5 | 61 | F05 | 10 µM FCCP | 01:15:53 | 115.86 |
| 5 | 62 | F05 | 10 µM FCCP | 01:16:07 | 114.17 |
| 5 | 63 | F05 | 10 µM FCCP | 01:16:21 | 112.41 |
| 5 | 64 | F05 | 10 µM FCCP | 01:16:35 | 110.79 |
| 5 | 65 | F05 | 10 µM FCCP | 01:16:50 | 109.13 |
| 5 | 66 | F05 | 10 µM FCCP | 01:17:04 | 107.54 |
| 5 | 67 | F05 | 10 µM FCCP | 01:17:18 | 105.94 |
| 5 | 68 | F05 | 10 µM FCCP | 01:17:32 | 104.36 |
| 5 | 69 | F05 | 10 µM FCCP | 01:17:46 | 102.82 |
| 6 | 70 | F05 | 10 µM FCCP | 01:23:20 | 130.90 |
| 6 | 71 | F05 | 10 µM FCCP | 01:23:34 | 124.80 |
| 6 | 72 | F05 | 10 µM FCCP | 01:23:48 | 121.87 |
| 6 | 73 | F05 | 10 µM FCCP | 01:24:02 | 119.49 |
| 6 | 74 | F05 | 10 µM FCCP | 01:24:16 | 117.34 |
| 6 | 75 | F05 | 10 µM FCCP | 01:24:30 | 115.38 |
| 6 | 76 | F05 | 10 µM FCCP | 01:24:44 | 113.58 |
| 6 | 77 | F05 | 10 µM FCCP | 01:24:59 | 111.81 |
| 6 | 78 | F05 | 10 µM FCCP | 01:25:13 | 110.12 |
| 6 | 79 | F05 | 10 µM FCCP | 01:25:27 | 108.43 |
| 6 | 80 | F05 | 10 µM FCCP | 01:25:41 | 106.81 |
| 6 | 81 | F05 | 10 µM FCCP | 01:25:55 | 105.27 |
| 6 | 82 | F05 | 10 µM FCCP | 01:26:10 | 103.64 |
| 6 | 83 | F05 | 10 µM FCCP | 01:26:24 | 102.07 |
| 7 | 84 | F05 | 10 µM FCCP | 01:31:58 | 130.73 |
| 7 | 85 | F05 | 10 µM FCCP | 01:32:12 | 124.86 |
| 7 | 86 | F05 | 10 µM FCCP | 01:32:26 | 121.91 |
| 7 | 87 | F05 | 10 µM FCCP | 01:32:40 | 119.67 |
| 7 | 88 | F05 | 10 µM FCCP | 01:32:54 | 117.47 |
| 7 | 89 | F05 | 10 µM FCCP | 01:33:08 | 115.57 |
| 7 | 90 | F05 | 10 µM FCCP | 01:33:23 | 113.77 |
| 7 | 91 | F05 | 10 µM FCCP | 01:33:37 | 112.04 |
| 7 | 92 | F05 | 10 µM FCCP | 01:33:51 | 110.31 |
| 7 | 93 | F05 | 10 µM FCCP | 01:34:05 | 108.74 |
| 7 | 94 | F05 | 10 µM FCCP | 01:34:19 | 107.12 |
| 7 | 95 | F05 | 10 µM FCCP | 01:34:33 | 105.56 |
| 7 | 96 | F05 | 10 µM FCCP | 01:34:48 | 104.13 |
| 7 | 97 | F05 | 10 µM FCCP | 01:35:02 | 102.54 |
| 8 | 98 | F05 | 10 µM FCCP | 01:40:36 | 131.25 |
| 8 | 99 | F05 | 10 µM FCCP | 01:40:50 | 125.47 |
| 8 | 100 | F05 | 10 µM FCCP | 01:41:04 | 122.55 |
| 8 | 101 | F05 | 10 µM FCCP | 01:41:18 | 120.24 |
| 8 | 102 | F05 | 10 µM FCCP | 01:41:32 | 118.23 |
| 8 | 103 | F05 | 10 µM FCCP | 01:41:46 | 116.39 |
| 8 | 104 | F05 | 10 µM FCCP | 01:42:00 | 114.62 |
| 8 | 105 | F05 | 10 µM FCCP | 01:42:15 | 112.94 |
| 8 | 106 | F05 | 10 µM FCCP | 01:42:29 | 111.30 |
| 8 | 107 | F05 | 10 µM FCCP | 01:42:43 | 109.79 |
| 8 | 108 | F05 | 10 µM FCCP | 01:42:57 | 108.26 |
| 8 | 109 | F05 | 10 µM FCCP | 01:43:11 | 106.75 |
| 8 | 110 | F05 | 10 µM FCCP | 01:43:26 | 105.31 |
| 8 | 111 | F05 | 10 µM FCCP | 01:43:40 | 103.88 |
| 9 | 112 | F05 | 10 µM FCCP | 01:49:14 | 131.86 |
| 9 | 113 | F05 | 10 µM FCCP | 01:49:28 | 126.34 |
| 9 | 114 | F05 | 10 µM FCCP | 01:49:42 | 123.53 |
| 9 | 115 | F05 | 10 µM FCCP | 01:49:56 | 121.21 |
| 9 | 116 | F05 | 10 µM FCCP | 01:50:10 | 119.26 |
| 9 | 117 | F05 | 10 µM FCCP | 01:50:24 | 117.52 |
| 9 | 118 | F05 | 10 µM FCCP | 01:50:38 | 115.79 |
| 9 | 119 | F05 | 10 µM FCCP | 01:50:53 | 114.19 |
| 9 | 120 | F05 | 10 µM FCCP | 01:51:07 | 112.69 |
| 9 | 121 | F05 | 10 µM FCCP | 01:51:21 | 111.13 |
| 9 | 122 | F05 | 10 µM FCCP | 01:51:35 | 109.73 |
| 9 | 123 | F05 | 10 µM FCCP | 01:51:50 | 108.30 |
| 9 | 124 | F05 | 10 µM FCCP | 01:52:04 | 106.94 |
| 9 | 125 | F05 | 10 µM FCCP | 01:52:18 | 105.59 |
| 10 | 126 | F05 | 10 µM FCCP | 01:57:52 | 132.35 |
| 10 | 127 | F05 | 10 µM FCCP | 01:58:06 | 127.08 |
| 10 | 128 | F05 | 10 µM FCCP | 01:58:20 | 124.40 |
| 10 | 129 | F05 | 10 µM FCCP | 01:58:34 | 122.34 |
| 10 | 130 | F05 | 10 µM FCCP | 01:58:48 | 120.35 |
| 10 | 131 | F05 | 10 µM FCCP | 01:59:03 | 118.64 |
| 10 | 132 | F05 | 10 µM FCCP | 01:59:17 | 116.97 |
| 10 | 133 | F05 | 10 µM FCCP | 01:59:31 | 115.44 |
| 10 | 134 | F05 | 10 µM FCCP | 01:59:45 | 114.02 |
| 10 | 135 | F05 | 10 µM FCCP | 01:59:59 | 112.58 |
| 10 | 136 | F05 | 10 µM FCCP | 02:00:14 | 111.22 |
| 10 | 137 | F05 | 10 µM FCCP | 02:00:28 | 109.93 |
| 10 | 138 | F05 | 10 µM FCCP | 02:00:42 | 108.66 |
| 10 | 139 | F05 | 10 µM FCCP | 02:00:56 | 107.38 |
| 11 | 140 | F05 | 10 µM FCCP | 02:06:30 | 133.32 |
| 11 | 141 | F05 | 10 µM FCCP | 02:06:44 | 128.86 |
| 11 | 142 | F05 | 10 µM FCCP | 02:06:58 | 126.32 |
| 11 | 143 | F05 | 10 µM FCCP | 02:07:12 | 124.40 |
| 11 | 144 | F05 | 10 µM FCCP | 02:07:26 | 122.64 |
| 11 | 145 | F05 | 10 µM FCCP | 02:07:41 | 120.97 |
| 11 | 146 | F05 | 10 µM FCCP | 02:07:55 | 119.38 |
| 11 | 147 | F05 | 10 µM FCCP | 02:08:09 | 117.93 |
| 11 | 148 | F05 | 10 µM FCCP | 02:08:23 | 116.50 |
| 11 | 149 | F05 | 10 µM FCCP | 02:08:37 | 115.19 |
| 11 | 150 | F05 | 10 µM FCCP | 02:08:52 | 113.88 |
| 11 | 151 | F05 | 10 µM FCCP | 02:09:06 | 112.65 |
| 11 | 152 | F05 | 10 µM FCCP | 02:09:20 | 111.41 |
| 11 | 153 | F05 | 10 µM FCCP | 02:09:34 | 110.19 |
| 12 | 154 | F05 | 10 µM FCCP | 02:15:08 | 134.28 |
| 12 | 155 | F05 | 10 µM FCCP | 02:15:22 | 130.22 |
| 12 | 156 | F05 | 10 µM FCCP | 02:15:37 | 127.78 |
| 12 | 157 | F05 | 10 µM FCCP | 02:15:53 | 125.67 |
| 12 | 158 | F05 | 10 µM FCCP | 02:16:08 | 123.93 |
| 12 | 159 | F05 | 10 µM FCCP | 02:16:22 | 122.47 |
| 12 | 160 | F05 | 10 µM FCCP | 02:16:36 | 121.00 |
| 12 | 161 | F05 | 10 µM FCCP | 02:16:50 | 119.58 |
| 12 | 162 | F05 | 10 µM FCCP | 02:17:04 | 118.30 |
| 12 | 163 | F05 | 10 µM FCCP | 02:17:18 | 116.97 |
| 12 | 164 | F05 | 10 µM FCCP | 02:17:33 | 115.73 |
| 12 | 165 | F05 | 10 µM FCCP | 02:17:47 | 114.55 |
| 12 | 166 | F05 | 10 µM FCCP | 02:18:01 | 113.42 |
| 13 | 167 | F05 | 10 µM FCCP | 02:23:39 | 133.88 |
| 13 | 168 | F05 | 10 µM FCCP | 02:23:54 | 130.60 |
| 13 | 169 | F05 | 10 µM FCCP | 02:24:08 | 128.53 |
| 13 | 170 | F05 | 10 µM FCCP | 02:24:22 | 126.76 |
| 13 | 171 | F05 | 10 µM FCCP | 02:24:36 | 125.16 |
| 13 | 172 | F05 | 10 µM FCCP | 02:24:51 | 123.55 |
| 13 | 173 | F05 | 10 µM FCCP | 02:25:07 | 122.05 |
| 13 | 174 | F05 | 10 µM FCCP | 02:25:22 | 120.71 |
| 13 | 175 | F05 | 10 µM FCCP | 02:25:36 | 119.52 |
| 13 | 176 | F05 | 10 µM FCCP | 02:25:50 | 118.28 |
| 13 | 177 | F05 | 10 µM FCCP | 02:26:04 | 117.18 |
| 13 | 178 | F05 | 10 µM FCCP | 02:26:18 | 116.02 |
| 13 | 179 | F05 | 10 µM FCCP | 02:26:33 | 114.89 |
| 14 | 180 | F05 | 10 µM FCCP | 02:32:07 | 135.60 |
| 14 | 181 | F05 | 10 µM FCCP | 02:32:21 | 131.91 |
| 14 | 182 | F05 | 10 µM FCCP | 02:32:35 | 129.76 |
| 14 | 183 | F05 | 10 µM FCCP | 02:32:50 | 128.05 |
| 14 | 184 | F05 | 10 µM FCCP | 02:33:04 | 126.57 |
| 14 | 185 | F05 | 10 µM FCCP | 02:33:18 | 125.12 |
| 14 | 186 | F05 | 10 µM FCCP | 02:33:32 | 123.76 |
| 14 | 187 | F05 | 10 µM FCCP | 02:33:46 | 122.49 |
| 14 | 188 | F05 | 10 µM FCCP | 02:34:01 | 121.31 |
| 14 | 189 | F05 | 10 µM FCCP | 02:34:15 | 120.14 |
| 14 | 190 | F05 | 10 µM FCCP | 02:34:29 | 118.99 |
| 14 | 191 | F05 | 10 µM FCCP | 02:34:43 | 117.91 |
| 14 | 192 | F05 | 10 µM FCCP | 02:34:57 | 116.83 |
| 14 | 193 | F05 | 10 µM FCCP | 02:35:12 | 115.85 |
| 15 | 194 | F05 | 10 µM FCCP | 02:40:46 | 136.12 |
| 15 | 195 | F05 | 10 µM FCCP | 02:41:00 | 132.58 |
| 15 | 196 | F05 | 10 µM FCCP | 02:41:14 | 130.45 |
| 15 | 197 | F05 | 10 µM FCCP | 02:41:28 | 128.91 |
| 15 | 198 | F05 | 10 µM FCCP | 02:41:43 | 127.38 |
| 15 | 199 | F05 | 10 µM FCCP | 02:41:57 | 126.03 |
| 15 | 200 | F05 | 10 µM FCCP | 02:42:11 | 124.68 |
| 15 | 201 | F05 | 10 µM FCCP | 02:42:25 | 123.52 |
| 15 | 202 | F05 | 10 µM FCCP | 02:42:39 | 122.35 |
| 15 | 203 | F05 | 10 µM FCCP | 02:42:53 | 121.24 |
| 15 | 204 | F05 | 10 µM FCCP | 02:43:08 | 120.15 |
| 15 | 205 | F05 | 10 µM FCCP | 02:43:22 | 119.06 |
| 15 | 206 | F05 | 10 µM FCCP | 02:43:36 | 118.06 |
| 15 | 207 | F05 | 10 µM FCCP | 02:43:50 | 117.11 |
| 16 | 208 | F05 | 10 µM FCCP | 02:49:25 | 136.52 |
| 16 | 209 | F05 | 10 µM FCCP | 02:49:40 | 133.07 |
| 16 | 210 | F05 | 10 µM FCCP | 02:49:54 | 131.05 |
| 16 | 211 | F05 | 10 µM FCCP | 02:50:08 | 129.47 |
| 16 | 212 | F05 | 10 µM FCCP | 02:50:22 | 128.12 |
| 16 | 213 | F05 | 10 µM FCCP | 02:50:36 | 126.73 |
| 16 | 214 | F05 | 10 µM FCCP | 02:50:50 | 125.57 |
| 16 | 215 | F05 | 10 µM FCCP | 02:51:05 | 124.36 |
| 16 | 216 | F05 | 10 µM FCCP | 02:51:19 | 123.18 |
| 16 | 217 | F05 | 10 µM FCCP | 02:51:33 | 122.15 |
| 16 | 218 | F05 | 10 µM FCCP | 02:51:47 | 121.14 |
| 16 | 219 | F05 | 10 µM FCCP | 02:52:01 | 120.14 |
| 16 | 220 | F05 | 10 µM FCCP | 02:52:16 | 119.26 |
| 16 | 221 | F05 | 10 µM FCCP | 02:52:30 | 118.23 |
| 1 | 0 | F06 | 10 µM FCCP | 00:40:06 | 127.80 |
| 1 | 1 | F06 | 10 µM FCCP | 00:40:20 | 121.24 |
| 1 | 2 | F06 | 10 µM FCCP | 00:40:34 | 118.28 |
| 1 | 3 | F06 | 10 µM FCCP | 00:40:48 | 116.09 |
| 1 | 4 | F06 | 10 µM FCCP | 00:41:02 | 114.21 |
| 1 | 5 | F06 | 10 µM FCCP | 00:41:17 | 112.55 |
| 1 | 6 | F06 | 10 µM FCCP | 00:41:31 | 111.03 |
| 1 | 7 | F06 | 10 µM FCCP | 00:41:45 | 109.53 |
| 1 | 8 | F06 | 10 µM FCCP | 00:41:59 | 108.25 |
| 1 | 9 | F06 | 10 µM FCCP | 00:42:13 | 106.96 |
| 1 | 10 | F06 | 10 µM FCCP | 00:42:28 | 105.65 |
| 1 | 11 | F06 | 10 µM FCCP | 00:42:42 | 104.53 |
| 1 | 12 | F06 | 10 µM FCCP | 00:42:56 | 103.31 |
| 1 | 13 | F06 | 10 µM FCCP | 00:43:10 | 102.21 |
| 2 | 14 | F06 | 10 µM FCCP | 00:48:43 | 129.99 |
| 2 | 15 | F06 | 10 µM FCCP | 00:48:57 | 124.99 |
| 2 | 16 | F06 | 10 µM FCCP | 00:49:12 | 122.33 |
| 2 | 17 | F06 | 10 µM FCCP | 00:49:26 | 120.12 |
| 2 | 18 | F06 | 10 µM FCCP | 00:49:40 | 118.18 |
| 2 | 19 | F06 | 10 µM FCCP | 00:49:54 | 116.41 |
| 2 | 20 | F06 | 10 µM FCCP | 00:50:08 | 114.81 |
| 2 | 21 | F06 | 10 µM FCCP | 00:50:22 | 113.30 |
| 2 | 22 | F06 | 10 µM FCCP | 00:50:37 | 111.89 |
| 2 | 23 | F06 | 10 µM FCCP | 00:50:51 | 110.51 |
| 2 | 24 | F06 | 10 µM FCCP | 00:51:05 | 109.18 |
| 2 | 25 | F06 | 10 µM FCCP | 00:51:19 | 108.00 |
| 2 | 26 | F06 | 10 µM FCCP | 00:51:33 | 106.68 |
| 2 | 27 | F06 | 10 µM FCCP | 00:51:48 | 105.57 |
| 3 | 28 | F06 | 10 µM FCCP | 00:57:21 | 131.63 |
| 3 | 29 | F06 | 10 µM FCCP | 00:57:35 | 127.26 |
| 3 | 30 | F06 | 10 µM FCCP | 00:57:49 | 124.63 |
| 3 | 31 | F06 | 10 µM FCCP | 00:58:03 | 122.47 |
| 3 | 32 | F06 | 10 µM FCCP | 00:58:17 | 120.61 |
| 3 | 33 | F06 | 10 µM FCCP | 00:58:31 | 118.86 |
| 3 | 34 | F06 | 10 µM FCCP | 00:58:46 | 117.18 |
| 3 | 35 | F06 | 10 µM FCCP | 00:59:00 | 115.61 |
| 3 | 36 | F06 | 10 µM FCCP | 00:59:14 | 114.19 |
| 3 | 37 | F06 | 10 µM FCCP | 00:59:28 | 112.84 |
| 3 | 38 | F06 | 10 µM FCCP | 00:59:42 | 111.54 |
| 3 | 39 | F06 | 10 µM FCCP | 00:59:56 | 110.29 |
| 3 | 40 | F06 | 10 µM FCCP | 01:00:11 | 108.99 |
| 3 | 41 | F06 | 10 µM FCCP | 01:00:25 | 107.82 |
| 4 | 42 | F06 | 10 µM FCCP | 01:06:04 | 131.24 |
| 4 | 43 | F06 | 10 µM FCCP | 01:06:18 | 125.71 |
| 4 | 44 | F06 | 10 µM FCCP | 01:06:33 | 122.67 |
| 4 | 45 | F06 | 10 µM FCCP | 01:06:47 | 120.27 |
| 4 | 46 | F06 | 10 µM FCCP | 01:07:01 | 118.34 |
| 4 | 47 | F06 | 10 µM FCCP | 01:07:15 | 116.33 |
| 4 | 48 | F06 | 10 µM FCCP | 01:07:29 | 114.51 |
| 4 | 49 | F06 | 10 µM FCCP | 01:07:43 | 112.79 |
| 4 | 50 | F06 | 10 µM FCCP | 01:07:58 | 111.15 |
| 4 | 51 | F06 | 10 µM FCCP | 01:08:12 | 109.60 |
| 4 | 52 | F06 | 10 µM FCCP | 01:08:26 | 107.96 |
| 4 | 53 | F06 | 10 µM FCCP | 01:08:40 | 106.45 |
| 4 | 54 | F06 | 10 µM FCCP | 01:08:54 | 104.90 |
| 4 | 55 | F06 | 10 µM FCCP | 01:09:09 | 103.42 |
| 5 | 56 | F06 | 10 µM FCCP | 01:14:42 | 129.75 |
| 5 | 57 | F06 | 10 µM FCCP | 01:14:56 | 123.63 |
| 5 | 58 | F06 | 10 µM FCCP | 01:15:10 | 120.43 |
| 5 | 59 | F06 | 10 µM FCCP | 01:15:25 | 117.99 |
| 5 | 60 | F06 | 10 µM FCCP | 01:15:39 | 115.81 |
| 5 | 61 | F06 | 10 µM FCCP | 01:15:53 | 113.78 |
| 5 | 62 | F06 | 10 µM FCCP | 01:16:07 | 111.85 |
| 5 | 63 | F06 | 10 µM FCCP | 01:16:21 | 110.07 |
| 5 | 64 | F06 | 10 µM FCCP | 01:16:35 | 108.31 |
| 5 | 65 | F06 | 10 µM FCCP | 01:16:50 | 106.58 |
| 5 | 66 | F06 | 10 µM FCCP | 01:17:04 | 104.85 |
| 5 | 67 | F06 | 10 µM FCCP | 01:17:18 | 103.16 |
| 5 | 68 | F06 | 10 µM FCCP | 01:17:32 | 101.52 |
| 5 | 69 | F06 | 10 µM FCCP | 01:17:46 | 99.88 |
| 6 | 70 | F06 | 10 µM FCCP | 01:23:20 | 129.20 |
| 6 | 71 | F06 | 10 µM FCCP | 01:23:34 | 123.06 |
| 6 | 72 | F06 | 10 µM FCCP | 01:23:48 | 119.92 |
| 6 | 73 | F06 | 10 µM FCCP | 01:24:02 | 117.39 |
| 6 | 74 | F06 | 10 µM FCCP | 01:24:16 | 115.21 |
| 6 | 75 | F06 | 10 µM FCCP | 01:24:30 | 113.05 |
| 6 | 76 | F06 | 10 µM FCCP | 01:24:44 | 111.21 |
| 6 | 77 | F06 | 10 µM FCCP | 01:24:59 | 109.30 |
| 6 | 78 | F06 | 10 µM FCCP | 01:25:13 | 107.58 |
| 6 | 79 | F06 | 10 µM FCCP | 01:25:27 | 105.78 |
| 6 | 80 | F06 | 10 µM FCCP | 01:25:41 | 104.07 |
| 6 | 81 | F06 | 10 µM FCCP | 01:25:55 | 102.34 |
| 6 | 82 | F06 | 10 µM FCCP | 01:26:10 | 100.59 |
| 6 | 83 | F06 | 10 µM FCCP | 01:26:24 | 98.94 |
| 7 | 84 | F06 | 10 µM FCCP | 01:31:58 | 129.19 |
| 7 | 85 | F06 | 10 µM FCCP | 01:32:12 | 123.08 |
| 7 | 86 | F06 | 10 µM FCCP | 01:32:26 | 119.95 |
| 7 | 87 | F06 | 10 µM FCCP | 01:32:40 | 117.53 |
| 7 | 88 | F06 | 10 µM FCCP | 01:32:54 | 115.34 |
| 7 | 89 | F06 | 10 µM FCCP | 01:33:08 | 113.31 |
| 7 | 90 | F06 | 10 µM FCCP | 01:33:23 | 111.32 |
| 7 | 91 | F06 | 10 µM FCCP | 01:33:37 | 109.51 |
| 7 | 92 | F06 | 10 µM FCCP | 01:33:51 | 107.75 |
| 7 | 93 | F06 | 10 µM FCCP | 01:34:05 | 105.99 |
| 7 | 94 | F06 | 10 µM FCCP | 01:34:19 | 104.24 |
| 7 | 95 | F06 | 10 µM FCCP | 01:34:33 | 102.68 |
| 7 | 96 | F06 | 10 µM FCCP | 01:34:48 | 101.14 |
| 7 | 97 | F06 | 10 µM FCCP | 01:35:02 | 99.50 |
| 8 | 98 | F06 | 10 µM FCCP | 01:40:36 | 129.61 |
| 8 | 99 | F06 | 10 µM FCCP | 01:40:50 | 123.70 |
| 8 | 100 | F06 | 10 µM FCCP | 01:41:04 | 120.65 |
| 8 | 101 | F06 | 10 µM FCCP | 01:41:18 | 118.24 |
| 8 | 102 | F06 | 10 µM FCCP | 01:41:32 | 116.03 |
| 8 | 103 | F06 | 10 µM FCCP | 01:41:46 | 114.01 |
| 8 | 104 | F06 | 10 µM FCCP | 01:42:00 | 112.21 |
| 8 | 105 | F06 | 10 µM FCCP | 01:42:15 | 110.41 |
| 8 | 106 | F06 | 10 µM FCCP | 01:42:29 | 108.64 |
| 8 | 107 | F06 | 10 µM FCCP | 01:42:43 | 107.00 |
| 8 | 108 | F06 | 10 µM FCCP | 01:42:57 | 105.38 |
| 8 | 109 | F06 | 10 µM FCCP | 01:43:11 | 103.87 |
| 8 | 110 | F06 | 10 µM FCCP | 01:43:26 | 102.31 |
| 8 | 111 | F06 | 10 µM FCCP | 01:43:40 | 100.77 |
| 9 | 112 | F06 | 10 µM FCCP | 01:49:14 | 130.16 |
| 9 | 113 | F06 | 10 µM FCCP | 01:49:28 | 124.53 |
| 9 | 114 | F06 | 10 µM FCCP | 01:49:42 | 121.48 |
| 9 | 115 | F06 | 10 µM FCCP | 01:49:56 | 119.10 |
| 9 | 116 | F06 | 10 µM FCCP | 01:50:10 | 117.02 |
| 9 | 117 | F06 | 10 µM FCCP | 01:50:24 | 115.09 |
| 9 | 118 | F06 | 10 µM FCCP | 01:50:38 | 113.29 |
| 9 | 119 | F06 | 10 µM FCCP | 01:50:53 | 111.62 |
| 9 | 120 | F06 | 10 µM FCCP | 01:51:07 | 109.94 |
| 9 | 121 | F06 | 10 µM FCCP | 01:51:21 | 108.33 |
| 9 | 122 | F06 | 10 µM FCCP | 01:51:35 | 106.80 |
| 9 | 123 | F06 | 10 µM FCCP | 01:51:50 | 105.26 |
| 9 | 124 | F06 | 10 µM FCCP | 01:52:04 | 103.93 |
| 9 | 125 | F06 | 10 µM FCCP | 01:52:18 | 102.46 |
| 10 | 126 | F06 | 10 µM FCCP | 01:57:52 | 130.70 |
| 10 | 127 | F06 | 10 µM FCCP | 01:58:06 | 125.38 |
| 10 | 128 | F06 | 10 µM FCCP | 01:58:20 | 122.43 |
| 10 | 129 | F06 | 10 µM FCCP | 01:58:34 | 120.20 |
| 10 | 130 | F06 | 10 µM FCCP | 01:58:48 | 118.16 |
| 10 | 131 | F06 | 10 µM FCCP | 01:59:03 | 116.26 |
| 10 | 132 | F06 | 10 µM FCCP | 01:59:17 | 114.55 |
| 10 | 133 | F06 | 10 µM FCCP | 01:59:31 | 112.87 |
| 10 | 134 | F06 | 10 µM FCCP | 01:59:45 | 111.42 |
| 10 | 135 | F06 | 10 µM FCCP | 01:59:59 | 109.88 |
| 10 | 136 | F06 | 10 µM FCCP | 02:00:14 | 108.44 |
| 10 | 137 | F06 | 10 µM FCCP | 02:00:28 | 107.06 |
| 10 | 138 | F06 | 10 µM FCCP | 02:00:42 | 105.66 |
| 10 | 139 | F06 | 10 µM FCCP | 02:00:56 | 104.36 |
| 11 | 140 | F06 | 10 µM FCCP | 02:06:30 | 131.71 |
| 11 | 141 | F06 | 10 µM FCCP | 02:06:44 | 127.14 |
| 11 | 142 | F06 | 10 µM FCCP | 02:06:58 | 124.61 |
| 11 | 143 | F06 | 10 µM FCCP | 02:07:12 | 122.39 |
| 11 | 144 | F06 | 10 µM FCCP | 02:07:26 | 120.61 |
| 11 | 145 | F06 | 10 µM FCCP | 02:07:41 | 118.81 |
| 11 | 146 | F06 | 10 µM FCCP | 02:07:55 | 117.14 |
| 11 | 147 | F06 | 10 µM FCCP | 02:08:09 | 115.56 |
| 11 | 148 | F06 | 10 µM FCCP | 02:08:23 | 114.08 |
| 11 | 149 | F06 | 10 µM FCCP | 02:08:37 | 112.73 |
| 11 | 150 | F06 | 10 µM FCCP | 02:08:52 | 111.23 |
| 11 | 151 | F06 | 10 µM FCCP | 02:09:06 | 109.85 |
| 11 | 152 | F06 | 10 µM FCCP | 02:09:20 | 108.62 |
| 11 | 153 | F06 | 10 µM FCCP | 02:09:34 | 107.30 |
| 12 | 154 | F06 | 10 µM FCCP | 02:15:08 | 132.78 |
| 12 | 155 | F06 | 10 µM FCCP | 02:15:22 | 128.70 |
| 12 | 156 | F06 | 10 µM FCCP | 02:15:37 | 126.03 |
| 12 | 157 | F06 | 10 µM FCCP | 02:15:53 | 123.81 |
| 12 | 158 | F06 | 10 µM FCCP | 02:16:08 | 121.95 |
| 12 | 159 | F06 | 10 µM FCCP | 02:16:22 | 120.29 |
| 12 | 160 | F06 | 10 µM FCCP | 02:16:36 | 118.75 |
| 12 | 161 | F06 | 10 µM FCCP | 02:16:50 | 117.37 |
| 12 | 162 | F06 | 10 µM FCCP | 02:17:04 | 115.89 |
| 12 | 163 | F06 | 10 µM FCCP | 02:17:18 | 114.54 |
| 12 | 164 | F06 | 10 µM FCCP | 02:17:33 | 113.17 |
| 12 | 165 | F06 | 10 µM FCCP | 02:17:47 | 111.89 |
| 12 | 166 | F06 | 10 µM FCCP | 02:18:01 | 110.70 |
| 13 | 167 | F06 | 10 µM FCCP | 02:23:39 | 132.29 |
| 13 | 168 | F06 | 10 µM FCCP | 02:23:54 | 129.02 |
| 13 | 169 | F06 | 10 µM FCCP | 02:24:08 | 126.85 |
| 13 | 170 | F06 | 10 µM FCCP | 02:24:22 | 125.00 |
| 13 | 171 | F06 | 10 µM FCCP | 02:24:36 | 123.21 |
| 13 | 172 | F06 | 10 µM FCCP | 02:24:51 | 121.42 |
| 13 | 173 | F06 | 10 µM FCCP | 02:25:07 | 119.81 |
| 13 | 174 | F06 | 10 µM FCCP | 02:25:22 | 118.42 |
| 13 | 175 | F06 | 10 µM FCCP | 02:25:36 | 117.09 |
| 13 | 176 | F06 | 10 µM FCCP | 02:25:50 | 115.81 |
| 13 | 177 | F06 | 10 µM FCCP | 02:26:04 | 114.57 |
| 13 | 178 | F06 | 10 µM FCCP | 02:26:18 | 113.33 |
| 13 | 179 | F06 | 10 µM FCCP | 02:26:33 | 112.16 |
| 14 | 180 | F06 | 10 µM FCCP | 02:32:07 | 134.24 |
| 14 | 181 | F06 | 10 µM FCCP | 02:32:21 | 130.33 |
| 14 | 182 | F06 | 10 µM FCCP | 02:32:35 | 128.18 |
| 14 | 183 | F06 | 10 µM FCCP | 02:32:50 | 126.36 |
| 14 | 184 | F06 | 10 µM FCCP | 02:33:04 | 124.65 |
| 14 | 185 | F06 | 10 µM FCCP | 02:33:18 | 123.05 |
| 14 | 186 | F06 | 10 µM FCCP | 02:33:32 | 121.65 |
| 14 | 187 | F06 | 10 µM FCCP | 02:33:46 | 120.24 |
| 14 | 188 | F06 | 10 µM FCCP | 02:34:01 | 118.90 |
| 14 | 189 | F06 | 10 µM FCCP | 02:34:15 | 117.62 |
| 14 | 190 | F06 | 10 µM FCCP | 02:34:29 | 116.43 |
| 14 | 191 | F06 | 10 µM FCCP | 02:34:43 | 115.20 |
| 14 | 192 | F06 | 10 µM FCCP | 02:34:57 | 114.09 |
| 14 | 193 | F06 | 10 µM FCCP | 02:35:12 | 113.08 |
| 15 | 194 | F06 | 10 µM FCCP | 02:40:46 | 134.83 |
| 15 | 195 | F06 | 10 µM FCCP | 02:41:00 | 131.07 |
| 15 | 196 | F06 | 10 µM FCCP | 02:41:14 | 128.83 |
| 15 | 197 | F06 | 10 µM FCCP | 02:41:28 | 127.14 |
| 15 | 198 | F06 | 10 µM FCCP | 02:41:43 | 125.43 |
| 15 | 199 | F06 | 10 µM FCCP | 02:41:57 | 124.04 |
| 15 | 200 | F06 | 10 µM FCCP | 02:42:11 | 122.63 |
| 15 | 201 | F06 | 10 µM FCCP | 02:42:25 | 121.27 |
| 15 | 202 | F06 | 10 µM FCCP | 02:42:39 | 120.05 |
| 15 | 203 | F06 | 10 µM FCCP | 02:42:53 | 118.78 |
| 15 | 204 | F06 | 10 µM FCCP | 02:43:08 | 117.57 |
| 15 | 205 | F06 | 10 µM FCCP | 02:43:22 | 116.56 |
| 15 | 206 | F06 | 10 µM FCCP | 02:43:36 | 115.38 |
| 15 | 207 | F06 | 10 µM FCCP | 02:43:50 | 114.38 |
| 16 | 208 | F06 | 10 µM FCCP | 02:49:25 | 135.33 |
| 16 | 209 | F06 | 10 µM FCCP | 02:49:40 | 131.81 |
| 16 | 210 | F06 | 10 µM FCCP | 02:49:54 | 129.62 |
| 16 | 211 | F06 | 10 µM FCCP | 02:50:08 | 127.85 |
| 16 | 212 | F06 | 10 µM FCCP | 02:50:22 | 126.33 |
| 16 | 213 | F06 | 10 µM FCCP | 02:50:36 | 124.90 |
| 16 | 214 | F06 | 10 µM FCCP | 02:50:50 | 123.61 |
| 16 | 215 | F06 | 10 µM FCCP | 02:51:05 | 122.29 |
| 16 | 216 | F06 | 10 µM FCCP | 02:51:19 | 121.03 |
| 16 | 217 | F06 | 10 µM FCCP | 02:51:33 | 119.82 |
| 16 | 218 | F06 | 10 µM FCCP | 02:51:47 | 118.75 |
| 16 | 219 | F06 | 10 µM FCCP | 02:52:01 | 117.64 |
| 16 | 220 | F06 | 10 µM FCCP | 02:52:16 | 116.58 |
| 16 | 221 | F06 | 10 µM FCCP | 02:52:30 | 115.53 |
| 1 | 0 | D01 | 2.5 µM BAM15 | 00:40:06 | 139.03 |
| 1 | 1 | D01 | 2.5 µM BAM15 | 00:40:20 | 135.66 |
| 1 | 2 | D01 | 2.5 µM BAM15 | 00:40:34 | 134.02 |
| 1 | 3 | D01 | 2.5 µM BAM15 | 00:40:48 | 132.61 |
| 1 | 4 | D01 | 2.5 µM BAM15 | 00:41:02 | 131.48 |
| 1 | 5 | D01 | 2.5 µM BAM15 | 00:41:17 | 130.36 |
| 1 | 6 | D01 | 2.5 µM BAM15 | 00:41:31 | 129.34 |
| 1 | 7 | D01 | 2.5 µM BAM15 | 00:41:45 | 128.43 |
| 1 | 8 | D01 | 2.5 µM BAM15 | 00:41:59 | 127.53 |
| 1 | 9 | D01 | 2.5 µM BAM15 | 00:42:13 | 126.64 |
| 1 | 10 | D01 | 2.5 µM BAM15 | 00:42:28 | 125.78 |
| 1 | 11 | D01 | 2.5 µM BAM15 | 00:42:42 | 124.94 |
| 1 | 12 | D01 | 2.5 µM BAM15 | 00:42:56 | 124.10 |
| 1 | 13 | D01 | 2.5 µM BAM15 | 00:43:10 | 123.40 |
| 2 | 14 | D01 | 2.5 µM BAM15 | 00:48:43 | 140.95 |
| 2 | 15 | D01 | 2.5 µM BAM15 | 00:48:57 | 137.38 |
| 2 | 16 | D01 | 2.5 µM BAM15 | 00:49:12 | 135.69 |
| 2 | 17 | D01 | 2.5 µM BAM15 | 00:49:26 | 134.24 |
| 2 | 18 | D01 | 2.5 µM BAM15 | 00:49:40 | 133.02 |
| 2 | 19 | D01 | 2.5 µM BAM15 | 00:49:54 | 131.80 |
| 2 | 20 | D01 | 2.5 µM BAM15 | 00:50:08 | 130.77 |
| 2 | 21 | D01 | 2.5 µM BAM15 | 00:50:22 | 129.65 |
| 2 | 22 | D01 | 2.5 µM BAM15 | 00:50:37 | 128.76 |
| 2 | 23 | D01 | 2.5 µM BAM15 | 00:50:51 | 127.90 |
| 2 | 24 | D01 | 2.5 µM BAM15 | 00:51:05 | 126.92 |
| 2 | 25 | D01 | 2.5 µM BAM15 | 00:51:19 | 126.17 |
| 2 | 26 | D01 | 2.5 µM BAM15 | 00:51:33 | 125.28 |
| 2 | 27 | D01 | 2.5 µM BAM15 | 00:51:48 | 124.51 |
| 3 | 28 | D01 | 2.5 µM BAM15 | 00:57:21 | 141.53 |
| 3 | 29 | D01 | 2.5 µM BAM15 | 00:57:35 | 137.99 |
| 3 | 30 | D01 | 2.5 µM BAM15 | 00:57:49 | 136.29 |
| 3 | 31 | D01 | 2.5 µM BAM15 | 00:58:03 | 134.92 |
| 3 | 32 | D01 | 2.5 µM BAM15 | 00:58:17 | 133.68 |
| 3 | 33 | D01 | 2.5 µM BAM15 | 00:58:31 | 132.50 |
| 3 | 34 | D01 | 2.5 µM BAM15 | 00:58:46 | 131.37 |
| 3 | 35 | D01 | 2.5 µM BAM15 | 00:59:00 | 130.25 |
| 3 | 36 | D01 | 2.5 µM BAM15 | 00:59:14 | 129.32 |
| 3 | 37 | D01 | 2.5 µM BAM15 | 00:59:28 | 128.43 |
| 3 | 38 | D01 | 2.5 µM BAM15 | 00:59:42 | 127.55 |
| 3 | 39 | D01 | 2.5 µM BAM15 | 00:59:56 | 126.68 |
| 3 | 40 | D01 | 2.5 µM BAM15 | 01:00:11 | 125.82 |
| 3 | 41 | D01 | 2.5 µM BAM15 | 01:00:25 | 125.07 |
| 4 | 42 | D01 | 2.5 µM BAM15 | 01:06:04 | 134.02 |
| 4 | 43 | D01 | 2.5 µM BAM15 | 01:06:18 | 122.70 |
| 4 | 44 | D01 | 2.5 µM BAM15 | 01:06:33 | 116.93 |
| 4 | 45 | D01 | 2.5 µM BAM15 | 01:06:47 | 112.55 |
| 4 | 46 | D01 | 2.5 µM BAM15 | 01:07:01 | 108.55 |
| 4 | 47 | D01 | 2.5 µM BAM15 | 01:07:15 | 104.82 |
| 4 | 48 | D01 | 2.5 µM BAM15 | 01:07:29 | 101.26 |
| 4 | 49 | D01 | 2.5 µM BAM15 | 01:07:43 | 97.79 |
| 4 | 50 | D01 | 2.5 µM BAM15 | 01:07:58 | 94.50 |
| 4 | 51 | D01 | 2.5 µM BAM15 | 01:08:12 | 91.30 |
| 4 | 52 | D01 | 2.5 µM BAM15 | 01:08:26 | 88.06 |
| 4 | 53 | D01 | 2.5 µM BAM15 | 01:08:40 | 85.06 |
| 4 | 54 | D01 | 2.5 µM BAM15 | 01:08:54 | 82.07 |
| 4 | 55 | D01 | 2.5 µM BAM15 | 01:09:09 | 79.17 |
| 5 | 56 | D01 | 2.5 µM BAM15 | 01:14:42 | 126.61 |
| 5 | 57 | D01 | 2.5 µM BAM15 | 01:14:56 | 112.41 |
| 5 | 58 | D01 | 2.5 µM BAM15 | 01:15:10 | 105.69 |
| 5 | 59 | D01 | 2.5 µM BAM15 | 01:15:25 | 100.52 |
| 5 | 60 | D01 | 2.5 µM BAM15 | 01:15:39 | 96.05 |
| 5 | 61 | D01 | 2.5 µM BAM15 | 01:15:53 | 91.85 |
| 5 | 62 | D01 | 2.5 µM BAM15 | 01:16:07 | 88.06 |
| 5 | 63 | D01 | 2.5 µM BAM15 | 01:16:21 | 84.41 |
| 5 | 64 | D01 | 2.5 µM BAM15 | 01:16:35 | 81.10 |
| 5 | 65 | D01 | 2.5 µM BAM15 | 01:16:50 | 77.93 |
| 5 | 66 | D01 | 2.5 µM BAM15 | 01:17:04 | 74.85 |
| 5 | 67 | D01 | 2.5 µM BAM15 | 01:17:18 | 71.99 |
| 5 | 68 | D01 | 2.5 µM BAM15 | 01:17:32 | 69.17 |
| 5 | 69 | D01 | 2.5 µM BAM15 | 01:17:46 | 66.59 |
| 6 | 70 | D01 | 2.5 µM BAM15 | 01:23:20 | 122.71 |
| 6 | 71 | D01 | 2.5 µM BAM15 | 01:23:34 | 107.69 |
| 6 | 72 | D01 | 2.5 µM BAM15 | 01:23:48 | 100.69 |
| 6 | 73 | D01 | 2.5 µM BAM15 | 01:24:02 | 95.21 |
| 6 | 74 | D01 | 2.5 µM BAM15 | 01:24:16 | 90.58 |
| 6 | 75 | D01 | 2.5 µM BAM15 | 01:24:30 | 86.31 |
| 6 | 76 | D01 | 2.5 µM BAM15 | 01:24:44 | 82.52 |
| 6 | 77 | D01 | 2.5 µM BAM15 | 01:24:59 | 78.82 |
| 6 | 78 | D01 | 2.5 µM BAM15 | 01:25:13 | 75.50 |
| 6 | 79 | D01 | 2.5 µM BAM15 | 01:25:27 | 72.31 |
| 6 | 80 | D01 | 2.5 µM BAM15 | 01:25:41 | 69.23 |
| 6 | 81 | D01 | 2.5 µM BAM15 | 01:25:55 | 66.48 |
| 6 | 82 | D01 | 2.5 µM BAM15 | 01:26:10 | 63.72 |
| 6 | 83 | D01 | 2.5 µM BAM15 | 01:26:24 | 61.19 |
| 7 | 84 | D01 | 2.5 µM BAM15 | 01:31:58 | 120.02 |
| 7 | 85 | D01 | 2.5 µM BAM15 | 01:32:12 | 105.14 |
| 7 | 86 | D01 | 2.5 µM BAM15 | 01:32:26 | 97.97 |
| 7 | 87 | D01 | 2.5 µM BAM15 | 01:32:40 | 92.55 |
| 7 | 88 | D01 | 2.5 µM BAM15 | 01:32:54 | 87.80 |
| 7 | 89 | D01 | 2.5 µM BAM15 | 01:33:08 | 83.51 |
| 7 | 90 | D01 | 2.5 µM BAM15 | 01:33:23 | 79.60 |
| 7 | 91 | D01 | 2.5 µM BAM15 | 01:33:37 | 75.86 |
| 7 | 92 | D01 | 2.5 µM BAM15 | 01:33:51 | 72.52 |
| 7 | 93 | D01 | 2.5 µM BAM15 | 01:34:05 | 69.41 |
| 7 | 94 | D01 | 2.5 µM BAM15 | 01:34:19 | 66.29 |
| 7 | 95 | D01 | 2.5 µM BAM15 | 01:34:33 | 63.53 |
| 7 | 96 | D01 | 2.5 µM BAM15 | 01:34:48 | 60.83 |
| 7 | 97 | D01 | 2.5 µM BAM15 | 01:35:02 | 58.28 |
| 8 | 98 | D01 | 2.5 µM BAM15 | 01:40:36 | 118.95 |
| 8 | 99 | D01 | 2.5 µM BAM15 | 01:40:50 | 104.73 |
| 8 | 100 | D01 | 2.5 µM BAM15 | 01:41:04 | 97.68 |
| 8 | 101 | D01 | 2.5 µM BAM15 | 01:41:18 | 92.11 |
| 8 | 102 | D01 | 2.5 µM BAM15 | 01:41:32 | 87.32 |
| 8 | 103 | D01 | 2.5 µM BAM15 | 01:41:46 | 82.96 |
| 8 | 104 | D01 | 2.5 µM BAM15 | 01:42:00 | 79.01 |
| 8 | 105 | D01 | 2.5 µM BAM15 | 01:42:15 | 75.26 |
| 8 | 106 | D01 | 2.5 µM BAM15 | 01:42:29 | 71.78 |
| 8 | 107 | D01 | 2.5 µM BAM15 | 01:42:43 | 68.51 |
| 8 | 108 | D01 | 2.5 µM BAM15 | 01:42:57 | 65.44 |
| 8 | 109 | D01 | 2.5 µM BAM15 | 01:43:11 | 62.57 |
| 8 | 110 | D01 | 2.5 µM BAM15 | 01:43:26 | 59.75 |
| 8 | 111 | D01 | 2.5 µM BAM15 | 01:43:40 | 57.22 |
| 9 | 112 | D01 | 2.5 µM BAM15 | 01:49:14 | 119.40 |
| 9 | 113 | D01 | 2.5 µM BAM15 | 01:49:28 | 107.00 |
| 9 | 114 | D01 | 2.5 µM BAM15 | 01:49:42 | 100.15 |
| 9 | 115 | D01 | 2.5 µM BAM15 | 01:49:56 | 94.72 |
| 9 | 116 | D01 | 2.5 µM BAM15 | 01:50:10 | 89.83 |
| 9 | 117 | D01 | 2.5 µM BAM15 | 01:50:24 | 85.46 |
| 9 | 118 | D01 | 2.5 µM BAM15 | 01:50:38 | 81.38 |
| 9 | 119 | D01 | 2.5 µM BAM15 | 01:50:53 | 77.49 |
| 9 | 120 | D01 | 2.5 µM BAM15 | 01:51:07 | 73.96 |
| 9 | 121 | D01 | 2.5 µM BAM15 | 01:51:21 | 70.62 |
| 9 | 122 | D01 | 2.5 µM BAM15 | 01:51:35 | 67.41 |
| 9 | 123 | D01 | 2.5 µM BAM15 | 01:51:50 | 64.47 |
| 9 | 124 | D01 | 2.5 µM BAM15 | 01:52:04 | 61.57 |
| 9 | 125 | D01 | 2.5 µM BAM15 | 01:52:18 | 58.88 |
| 10 | 126 | D01 | 2.5 µM BAM15 | 01:57:52 | 119.22 |
| 10 | 127 | D01 | 2.5 µM BAM15 | 01:58:06 | 106.99 |
| 10 | 128 | D01 | 2.5 µM BAM15 | 01:58:20 | 100.30 |
| 10 | 129 | D01 | 2.5 µM BAM15 | 01:58:34 | 94.93 |
| 10 | 130 | D01 | 2.5 µM BAM15 | 01:58:48 | 90.13 |
| 10 | 131 | D01 | 2.5 µM BAM15 | 01:59:03 | 85.63 |
| 10 | 132 | D01 | 2.5 µM BAM15 | 01:59:17 | 81.61 |
| 10 | 133 | D01 | 2.5 µM BAM15 | 01:59:31 | 77.77 |
| 10 | 134 | D01 | 2.5 µM BAM15 | 01:59:45 | 74.33 |
| 10 | 135 | D01 | 2.5 µM BAM15 | 01:59:59 | 70.95 |
| 10 | 136 | D01 | 2.5 µM BAM15 | 02:00:14 | 67.80 |
| 10 | 137 | D01 | 2.5 µM BAM15 | 02:00:28 | 64.80 |
| 10 | 138 | D01 | 2.5 µM BAM15 | 02:00:42 | 61.95 |
| 10 | 139 | D01 | 2.5 µM BAM15 | 02:00:56 | 59.26 |
| 11 | 140 | D01 | 2.5 µM BAM15 | 02:06:30 | 118.98 |
| 11 | 141 | D01 | 2.5 µM BAM15 | 02:06:44 | 106.97 |
| 11 | 142 | D01 | 2.5 µM BAM15 | 02:06:58 | 100.33 |
| 11 | 143 | D01 | 2.5 µM BAM15 | 02:07:12 | 94.91 |
| 11 | 144 | D01 | 2.5 µM BAM15 | 02:07:26 | 90.17 |
| 11 | 145 | D01 | 2.5 µM BAM15 | 02:07:41 | 85.79 |
| 11 | 146 | D01 | 2.5 µM BAM15 | 02:07:55 | 81.73 |
| 11 | 147 | D01 | 2.5 µM BAM15 | 02:08:09 | 77.96 |
| 11 | 148 | D01 | 2.5 µM BAM15 | 02:08:23 | 74.44 |
| 11 | 149 | D01 | 2.5 µM BAM15 | 02:08:37 | 71.15 |
| 11 | 150 | D01 | 2.5 µM BAM15 | 02:08:52 | 67.99 |
| 11 | 151 | D01 | 2.5 µM BAM15 | 02:09:06 | 65.02 |
| 11 | 152 | D01 | 2.5 µM BAM15 | 02:09:20 | 62.17 |
| 11 | 153 | D01 | 2.5 µM BAM15 | 02:09:34 | 59.46 |
| 12 | 154 | D01 | 2.5 µM BAM15 | 02:15:08 | 118.81 |
| 12 | 155 | D01 | 2.5 µM BAM15 | 02:15:22 | 106.98 |
| 12 | 156 | D01 | 2.5 µM BAM15 | 02:15:37 | 99.92 |
| 12 | 157 | D01 | 2.5 µM BAM15 | 02:15:53 | 94.09 |
| 12 | 158 | D01 | 2.5 µM BAM15 | 02:16:08 | 89.24 |
| 12 | 159 | D01 | 2.5 µM BAM15 | 02:16:22 | 85.04 |
| 12 | 160 | D01 | 2.5 µM BAM15 | 02:16:36 | 81.15 |
| 12 | 161 | D01 | 2.5 µM BAM15 | 02:16:50 | 77.43 |
| 12 | 162 | D01 | 2.5 µM BAM15 | 02:17:04 | 74.00 |
| 12 | 163 | D01 | 2.5 µM BAM15 | 02:17:18 | 70.68 |
| 12 | 164 | D01 | 2.5 µM BAM15 | 02:17:33 | 67.54 |
| 12 | 165 | D01 | 2.5 µM BAM15 | 02:17:47 | 64.66 |
| 12 | 166 | D01 | 2.5 µM BAM15 | 02:18:01 | 61.83 |
| 13 | 167 | D01 | 2.5 µM BAM15 | 02:23:39 | 114.71 |
| 13 | 168 | D01 | 2.5 µM BAM15 | 02:23:54 | 105.02 |
| 13 | 169 | D01 | 2.5 µM BAM15 | 02:24:08 | 98.90 |
| 13 | 170 | D01 | 2.5 µM BAM15 | 02:24:22 | 93.72 |
| 13 | 171 | D01 | 2.5 µM BAM15 | 02:24:36 | 89.14 |
| 13 | 172 | D01 | 2.5 µM BAM15 | 02:24:51 | 84.67 |
| 13 | 173 | D01 | 2.5 µM BAM15 | 02:25:07 | 80.30 |
| 13 | 174 | D01 | 2.5 µM BAM15 | 02:25:22 | 76.63 |
| 13 | 175 | D01 | 2.5 µM BAM15 | 02:25:36 | 73.30 |
| 13 | 176 | D01 | 2.5 µM BAM15 | 02:25:50 | 70.05 |
| 13 | 177 | D01 | 2.5 µM BAM15 | 02:26:04 | 67.07 |
| 13 | 178 | D01 | 2.5 µM BAM15 | 02:26:18 | 64.09 |
| 13 | 179 | D01 | 2.5 µM BAM15 | 02:26:33 | 61.34 |
| 14 | 180 | D01 | 2.5 µM BAM15 | 02:32:07 | 118.45 |
| 14 | 181 | D01 | 2.5 µM BAM15 | 02:32:21 | 106.75 |
| 14 | 182 | D01 | 2.5 µM BAM15 | 02:32:35 | 100.31 |
| 14 | 183 | D01 | 2.5 µM BAM15 | 02:32:50 | 95.03 |
| 14 | 184 | D01 | 2.5 µM BAM15 | 02:33:04 | 90.44 |
| 14 | 185 | D01 | 2.5 µM BAM15 | 02:33:18 | 86.11 |
| 14 | 186 | D01 | 2.5 µM BAM15 | 02:33:32 | 82.14 |
| 14 | 187 | D01 | 2.5 µM BAM15 | 02:33:46 | 78.39 |
| 14 | 188 | D01 | 2.5 µM BAM15 | 02:34:01 | 75.04 |
| 14 | 189 | D01 | 2.5 µM BAM15 | 02:34:15 | 71.76 |
| 14 | 190 | D01 | 2.5 µM BAM15 | 02:34:29 | 68.60 |
| 14 | 191 | D01 | 2.5 µM BAM15 | 02:34:43 | 65.72 |
| 14 | 192 | D01 | 2.5 µM BAM15 | 02:34:57 | 62.81 |
| 14 | 193 | D01 | 2.5 µM BAM15 | 02:35:12 | 60.25 |
| 15 | 194 | D01 | 2.5 µM BAM15 | 02:40:46 | 118.62 |
| 15 | 195 | D01 | 2.5 µM BAM15 | 02:41:00 | 107.07 |
| 15 | 196 | D01 | 2.5 µM BAM15 | 02:41:14 | 100.63 |
| 15 | 197 | D01 | 2.5 µM BAM15 | 02:41:28 | 95.40 |
| 15 | 198 | D01 | 2.5 µM BAM15 | 02:41:43 | 90.77 |
| 15 | 199 | D01 | 2.5 µM BAM15 | 02:41:57 | 86.57 |
| 15 | 200 | D01 | 2.5 µM BAM15 | 02:42:11 | 82.68 |
| 15 | 201 | D01 | 2.5 µM BAM15 | 02:42:25 | 78.94 |
| 15 | 202 | D01 | 2.5 µM BAM15 | 02:42:39 | 75.53 |
| 15 | 203 | D01 | 2.5 µM BAM15 | 02:42:53 | 72.34 |
| 15 | 204 | D01 | 2.5 µM BAM15 | 02:43:08 | 69.18 |
| 15 | 205 | D01 | 2.5 µM BAM15 | 02:43:22 | 66.32 |
| 15 | 206 | D01 | 2.5 µM BAM15 | 02:43:36 | 63.44 |
| 15 | 207 | D01 | 2.5 µM BAM15 | 02:43:50 | 60.78 |
| 16 | 208 | D01 | 2.5 µM BAM15 | 02:49:25 | 118.52 |
| 16 | 209 | D01 | 2.5 µM BAM15 | 02:49:40 | 107.15 |
| 16 | 210 | D01 | 2.5 µM BAM15 | 02:49:54 | 100.73 |
| 16 | 211 | D01 | 2.5 µM BAM15 | 02:50:08 | 95.58 |
| 16 | 212 | D01 | 2.5 µM BAM15 | 02:50:22 | 90.98 |
| 16 | 213 | D01 | 2.5 µM BAM15 | 02:50:36 | 86.77 |
| 16 | 214 | D01 | 2.5 µM BAM15 | 02:50:50 | 82.91 |
| 16 | 215 | D01 | 2.5 µM BAM15 | 02:51:05 | 79.21 |
| 16 | 216 | D01 | 2.5 µM BAM15 | 02:51:19 | 75.82 |
| 16 | 217 | D01 | 2.5 µM BAM15 | 02:51:33 | 72.66 |
| 16 | 218 | D01 | 2.5 µM BAM15 | 02:51:47 | 69.57 |
| 16 | 219 | D01 | 2.5 µM BAM15 | 02:52:01 | 66.76 |
| 16 | 220 | D01 | 2.5 µM BAM15 | 02:52:16 | 63.85 |
| 16 | 221 | D01 | 2.5 µM BAM15 | 02:52:30 | 61.23 |
| 1 | 0 | D02 | 2.5 µM BAM15 | 00:40:06 | 130.07 |
| 1 | 1 | D02 | 2.5 µM BAM15 | 00:40:20 | 123.01 |
| 1 | 2 | D02 | 2.5 µM BAM15 | 00:40:34 | 120.25 |
| 1 | 3 | D02 | 2.5 µM BAM15 | 00:40:48 | 118.22 |
| 1 | 4 | D02 | 2.5 µM BAM15 | 00:41:02 | 116.53 |
| 1 | 5 | D02 | 2.5 µM BAM15 | 00:41:17 | 114.97 |
| 1 | 6 | D02 | 2.5 µM BAM15 | 00:41:31 | 113.46 |
| 1 | 7 | D02 | 2.5 µM BAM15 | 00:41:45 | 112.20 |
| 1 | 8 | D02 | 2.5 µM BAM15 | 00:41:59 | 111.00 |
| 1 | 9 | D02 | 2.5 µM BAM15 | 00:42:13 | 109.78 |
| 1 | 10 | D02 | 2.5 µM BAM15 | 00:42:28 | 108.58 |
| 1 | 11 | D02 | 2.5 µM BAM15 | 00:42:42 | 107.44 |
| 1 | 12 | D02 | 2.5 µM BAM15 | 00:42:56 | 106.44 |
| 1 | 13 | D02 | 2.5 µM BAM15 | 00:43:10 | 105.46 |
| 2 | 14 | D02 | 2.5 µM BAM15 | 00:48:43 | 132.79 |
| 2 | 15 | D02 | 2.5 µM BAM15 | 00:48:57 | 128.11 |
| 2 | 16 | D02 | 2.5 µM BAM15 | 00:49:12 | 125.67 |
| 2 | 17 | D02 | 2.5 µM BAM15 | 00:49:26 | 123.62 |
| 2 | 18 | D02 | 2.5 µM BAM15 | 00:49:40 | 121.95 |
| 2 | 19 | D02 | 2.5 µM BAM15 | 00:49:54 | 120.25 |
| 2 | 20 | D02 | 2.5 µM BAM15 | 00:50:08 | 118.74 |
| 2 | 21 | D02 | 2.5 µM BAM15 | 00:50:22 | 117.34 |
| 2 | 22 | D02 | 2.5 µM BAM15 | 00:50:37 | 116.00 |
| 2 | 23 | D02 | 2.5 µM BAM15 | 00:50:51 | 114.73 |
| 2 | 24 | D02 | 2.5 µM BAM15 | 00:51:05 | 113.49 |
| 2 | 25 | D02 | 2.5 µM BAM15 | 00:51:19 | 112.37 |
| 2 | 26 | D02 | 2.5 µM BAM15 | 00:51:33 | 111.13 |
| 2 | 27 | D02 | 2.5 µM BAM15 | 00:51:48 | 110.08 |
| 3 | 28 | D02 | 2.5 µM BAM15 | 00:57:21 | 133.57 |
| 3 | 29 | D02 | 2.5 µM BAM15 | 00:57:35 | 129.07 |
| 3 | 30 | D02 | 2.5 µM BAM15 | 00:57:49 | 126.68 |
| 3 | 31 | D02 | 2.5 µM BAM15 | 00:58:03 | 124.75 |
| 3 | 32 | D02 | 2.5 µM BAM15 | 00:58:17 | 123.06 |
| 3 | 33 | D02 | 2.5 µM BAM15 | 00:58:31 | 121.44 |
| 3 | 34 | D02 | 2.5 µM BAM15 | 00:58:46 | 119.95 |
| 3 | 35 | D02 | 2.5 µM BAM15 | 00:59:00 | 118.55 |
| 3 | 36 | D02 | 2.5 µM BAM15 | 00:59:14 | 117.19 |
| 3 | 37 | D02 | 2.5 µM BAM15 | 00:59:28 | 116.02 |
| 3 | 38 | D02 | 2.5 µM BAM15 | 00:59:42 | 114.69 |
| 3 | 39 | D02 | 2.5 µM BAM15 | 00:59:56 | 113.65 |
| 3 | 40 | D02 | 2.5 µM BAM15 | 01:00:11 | 112.46 |
| 3 | 41 | D02 | 2.5 µM BAM15 | 01:00:25 | 111.40 |
| 4 | 42 | D02 | 2.5 µM BAM15 | 01:06:04 | 122.58 |
| 4 | 43 | D02 | 2.5 µM BAM15 | 01:06:18 | 108.51 |
| 4 | 44 | D02 | 2.5 µM BAM15 | 01:06:33 | 101.44 |
| 4 | 45 | D02 | 2.5 µM BAM15 | 01:06:47 | 95.82 |
| 4 | 46 | D02 | 2.5 µM BAM15 | 01:07:01 | 90.86 |
| 4 | 47 | D02 | 2.5 µM BAM15 | 01:07:15 | 85.99 |
| 4 | 48 | D02 | 2.5 µM BAM15 | 01:07:29 | 81.35 |
| 4 | 49 | D02 | 2.5 µM BAM15 | 01:07:43 | 76.80 |
| 4 | 50 | D02 | 2.5 µM BAM15 | 01:07:58 | 72.42 |
| 4 | 51 | D02 | 2.5 µM BAM15 | 01:08:12 | 68.09 |
| 4 | 52 | D02 | 2.5 µM BAM15 | 01:08:26 | 63.82 |
| 4 | 53 | D02 | 2.5 µM BAM15 | 01:08:40 | 59.71 |
| 4 | 54 | D02 | 2.5 µM BAM15 | 01:08:54 | 55.51 |
| 4 | 55 | D02 | 2.5 µM BAM15 | 01:09:09 | 51.53 |
| 5 | 56 | D02 | 2.5 µM BAM15 | 01:14:42 | 113.73 |
| 5 | 57 | D02 | 2.5 µM BAM15 | 01:14:56 | 96.16 |
| 5 | 58 | D02 | 2.5 µM BAM15 | 01:15:10 | 87.61 |
| 5 | 59 | D02 | 2.5 µM BAM15 | 01:15:25 | 81.02 |
| 5 | 60 | D02 | 2.5 µM BAM15 | 01:15:39 | 75.06 |
| 5 | 61 | D02 | 2.5 µM BAM15 | 01:15:53 | 69.48 |
| 5 | 62 | D02 | 2.5 µM BAM15 | 01:16:07 | 64.36 |
| 5 | 63 | D02 | 2.5 µM BAM15 | 01:16:21 | 59.42 |
| 5 | 64 | D02 | 2.5 µM BAM15 | 01:16:35 | 54.78 |
| 5 | 65 | D02 | 2.5 µM BAM15 | 01:16:50 | 50.28 |
| 5 | 66 | D02 | 2.5 µM BAM15 | 01:17:04 | 45.89 |
| 5 | 67 | D02 | 2.5 µM BAM15 | 01:17:18 | 41.76 |
| 5 | 68 | D02 | 2.5 µM BAM15 | 01:17:32 | 37.87 |
| 5 | 69 | D02 | 2.5 µM BAM15 | 01:17:46 | 34.47 |
| 6 | 70 | D02 | 2.5 µM BAM15 | 01:23:20 | 109.43 |
| 6 | 71 | D02 | 2.5 µM BAM15 | 01:23:34 | 90.57 |
| 6 | 72 | D02 | 2.5 µM BAM15 | 01:23:48 | 81.44 |
| 6 | 73 | D02 | 2.5 µM BAM15 | 01:24:02 | 74.19 |
| 6 | 74 | D02 | 2.5 µM BAM15 | 01:24:16 | 68.00 |
| 6 | 75 | D02 | 2.5 µM BAM15 | 01:24:30 | 62.19 |
| 6 | 76 | D02 | 2.5 µM BAM15 | 01:24:44 | 56.95 |
| 6 | 77 | D02 | 2.5 µM BAM15 | 01:24:59 | 51.90 |
| 6 | 78 | D02 | 2.5 µM BAM15 | 01:25:13 | 47.31 |
| 6 | 79 | D02 | 2.5 µM BAM15 | 01:25:27 | 42.88 |
| 6 | 80 | D02 | 2.5 µM BAM15 | 01:25:41 | 38.76 |
| 6 | 81 | D02 | 2.5 µM BAM15 | 01:25:55 | 35.08 |
| 6 | 82 | D02 | 2.5 µM BAM15 | 01:26:10 | 31.79 |
| 6 | 83 | D02 | 2.5 µM BAM15 | 01:26:24 | 29.16 |
| 7 | 84 | D02 | 2.5 µM BAM15 | 01:31:58 | 107.10 |
| 7 | 85 | D02 | 2.5 µM BAM15 | 01:32:12 | 88.30 |
| 7 | 86 | D02 | 2.5 µM BAM15 | 01:32:26 | 78.93 |
| 7 | 87 | D02 | 2.5 µM BAM15 | 01:32:40 | 71.81 |
| 7 | 88 | D02 | 2.5 µM BAM15 | 01:32:54 | 65.40 |
| 7 | 89 | D02 | 2.5 µM BAM15 | 01:33:08 | 59.61 |
| 7 | 90 | D02 | 2.5 µM BAM15 | 01:33:23 | 54.30 |
| 7 | 91 | D02 | 2.5 µM BAM15 | 01:33:37 | 49.28 |
| 7 | 92 | D02 | 2.5 µM BAM15 | 01:33:51 | 44.71 |
| 7 | 93 | D02 | 2.5 µM BAM15 | 01:34:05 | 40.48 |
| 7 | 94 | D02 | 2.5 µM BAM15 | 01:34:19 | 36.47 |
| 7 | 95 | D02 | 2.5 µM BAM15 | 01:34:33 | 33.04 |
| 7 | 96 | D02 | 2.5 µM BAM15 | 01:34:48 | 30.07 |
| 7 | 97 | D02 | 2.5 µM BAM15 | 01:35:02 | 27.74 |
| 8 | 98 | D02 | 2.5 µM BAM15 | 01:40:36 | 105.81 |
| 8 | 99 | D02 | 2.5 µM BAM15 | 01:40:50 | 87.06 |
| 8 | 100 | D02 | 2.5 µM BAM15 | 01:41:04 | 77.78 |
| 8 | 101 | D02 | 2.5 µM BAM15 | 01:41:18 | 70.51 |
| 8 | 102 | D02 | 2.5 µM BAM15 | 01:41:32 | 64.19 |
| 8 | 103 | D02 | 2.5 µM BAM15 | 01:41:46 | 58.36 |
| 8 | 104 | D02 | 2.5 µM BAM15 | 01:42:00 | 53.12 |
| 8 | 105 | D02 | 2.5 µM BAM15 | 01:42:15 | 48.15 |
| 8 | 106 | D02 | 2.5 µM BAM15 | 01:42:29 | 43.58 |
| 8 | 107 | D02 | 2.5 µM BAM15 | 01:42:43 | 39.31 |
| 8 | 108 | D02 | 2.5 µM BAM15 | 01:42:57 | 35.46 |
| 8 | 109 | D02 | 2.5 µM BAM15 | 01:43:11 | 32.10 |
| 8 | 110 | D02 | 2.5 µM BAM15 | 01:43:26 | 29.22 |
| 8 | 111 | D02 | 2.5 µM BAM15 | 01:43:40 | 27.05 |
| 9 | 112 | D02 | 2.5 µM BAM15 | 01:49:14 | 105.03 |
| 9 | 113 | D02 | 2.5 µM BAM15 | 01:49:28 | 86.53 |
| 9 | 114 | D02 | 2.5 µM BAM15 | 01:49:42 | 77.19 |
| 9 | 115 | D02 | 2.5 µM BAM15 | 01:49:56 | 69.94 |
| 9 | 116 | D02 | 2.5 µM BAM15 | 01:50:10 | 63.62 |
| 9 | 117 | D02 | 2.5 µM BAM15 | 01:50:24 | 57.84 |
| 9 | 118 | D02 | 2.5 µM BAM15 | 01:50:38 | 52.59 |
| 9 | 119 | D02 | 2.5 µM BAM15 | 01:50:53 | 47.59 |
| 9 | 120 | D02 | 2.5 µM BAM15 | 01:51:07 | 43.03 |
| 9 | 121 | D02 | 2.5 µM BAM15 | 01:51:21 | 38.82 |
| 9 | 122 | D02 | 2.5 µM BAM15 | 01:51:35 | 34.95 |
| 9 | 123 | D02 | 2.5 µM BAM15 | 01:51:50 | 31.62 |
| 9 | 124 | D02 | 2.5 µM BAM15 | 01:52:04 | 28.80 |
| 9 | 125 | D02 | 2.5 µM BAM15 | 01:52:18 | 26.63 |
| 10 | 126 | D02 | 2.5 µM BAM15 | 01:57:52 | 105.39 |
| 10 | 127 | D02 | 2.5 µM BAM15 | 01:58:06 | 88.46 |
| 10 | 128 | D02 | 2.5 µM BAM15 | 01:58:20 | 79.41 |
| 10 | 129 | D02 | 2.5 µM BAM15 | 01:58:34 | 72.28 |
| 10 | 130 | D02 | 2.5 µM BAM15 | 01:58:48 | 65.97 |
| 10 | 131 | D02 | 2.5 µM BAM15 | 01:59:03 | 60.04 |
| 10 | 132 | D02 | 2.5 µM BAM15 | 01:59:17 | 54.64 |
| 10 | 133 | D02 | 2.5 µM BAM15 | 01:59:31 | 49.62 |
| 10 | 134 | D02 | 2.5 µM BAM15 | 01:59:45 | 45.08 |
| 10 | 135 | D02 | 2.5 µM BAM15 | 01:59:59 | 40.68 |
| 10 | 136 | D02 | 2.5 µM BAM15 | 02:00:14 | 36.67 |
| 10 | 137 | D02 | 2.5 µM BAM15 | 02:00:28 | 33.14 |
| 10 | 138 | D02 | 2.5 µM BAM15 | 02:00:42 | 30.06 |
| 10 | 139 | D02 | 2.5 µM BAM15 | 02:00:56 | 27.64 |
| 11 | 140 | D02 | 2.5 µM BAM15 | 02:06:30 | 106.57 |
| 11 | 141 | D02 | 2.5 µM BAM15 | 02:06:44 | 90.89 |
| 11 | 142 | D02 | 2.5 µM BAM15 | 02:06:58 | 82.07 |
| 11 | 143 | D02 | 2.5 µM BAM15 | 02:07:12 | 74.85 |
| 11 | 144 | D02 | 2.5 µM BAM15 | 02:07:26 | 68.57 |
| 11 | 145 | D02 | 2.5 µM BAM15 | 02:07:41 | 62.62 |
| 11 | 146 | D02 | 2.5 µM BAM15 | 02:07:55 | 57.21 |
| 11 | 147 | D02 | 2.5 µM BAM15 | 02:08:09 | 52.14 |
| 11 | 148 | D02 | 2.5 µM BAM15 | 02:08:23 | 47.44 |
| 11 | 149 | D02 | 2.5 µM BAM15 | 02:08:37 | 43.03 |
| 11 | 150 | D02 | 2.5 µM BAM15 | 02:08:52 | 38.86 |
| 11 | 151 | D02 | 2.5 µM BAM15 | 02:09:06 | 35.09 |
| 11 | 152 | D02 | 2.5 µM BAM15 | 02:09:20 | 31.80 |
| 11 | 153 | D02 | 2.5 µM BAM15 | 02:09:34 | 29.00 |
| 12 | 154 | D02 | 2.5 µM BAM15 | 02:15:08 | 107.06 |
| 12 | 155 | D02 | 2.5 µM BAM15 | 02:15:22 | 91.79 |
| 12 | 156 | D02 | 2.5 µM BAM15 | 02:15:37 | 82.55 |
| 12 | 157 | D02 | 2.5 µM BAM15 | 02:15:53 | 74.77 |
| 12 | 158 | D02 | 2.5 µM BAM15 | 02:16:08 | 68.34 |
| 12 | 159 | D02 | 2.5 µM BAM15 | 02:16:22 | 62.75 |
| 12 | 160 | D02 | 2.5 µM BAM15 | 02:16:36 | 57.46 |
| 12 | 161 | D02 | 2.5 µM BAM15 | 02:16:50 | 52.48 |
| 12 | 162 | D02 | 2.5 µM BAM15 | 02:17:04 | 47.81 |
| 12 | 163 | D02 | 2.5 µM BAM15 | 02:17:18 | 43.40 |
| 12 | 164 | D02 | 2.5 µM BAM15 | 02:17:33 | 39.35 |
| 12 | 165 | D02 | 2.5 µM BAM15 | 02:17:47 | 35.62 |
| 12 | 166 | D02 | 2.5 µM BAM15 | 02:18:01 | 32.26 |
| 13 | 167 | D02 | 2.5 µM BAM15 | 02:23:39 | 101.70 |
| 13 | 168 | D02 | 2.5 µM BAM15 | 02:23:54 | 89.24 |
| 13 | 169 | D02 | 2.5 µM BAM15 | 02:24:08 | 81.17 |
| 13 | 170 | D02 | 2.5 µM BAM15 | 02:24:22 | 74.40 |
| 13 | 171 | D02 | 2.5 µM BAM15 | 02:24:36 | 68.27 |
| 13 | 172 | D02 | 2.5 µM BAM15 | 02:24:51 | 62.30 |
| 13 | 173 | D02 | 2.5 µM BAM15 | 02:25:07 | 56.49 |
| 13 | 174 | D02 | 2.5 µM BAM15 | 02:25:22 | 51.55 |
| 13 | 175 | D02 | 2.5 µM BAM15 | 02:25:36 | 47.13 |
| 13 | 176 | D02 | 2.5 µM BAM15 | 02:25:50 | 42.81 |
| 13 | 177 | D02 | 2.5 µM BAM15 | 02:26:04 | 38.89 |
| 13 | 178 | D02 | 2.5 µM BAM15 | 02:26:18 | 35.19 |
| 13 | 179 | D02 | 2.5 µM BAM15 | 02:26:33 | 31.93 |
| 14 | 180 | D02 | 2.5 µM BAM15 | 02:32:07 | 106.55 |
| 14 | 181 | D02 | 2.5 µM BAM15 | 02:32:21 | 91.50 |
| 14 | 182 | D02 | 2.5 µM BAM15 | 02:32:35 | 83.08 |
| 14 | 183 | D02 | 2.5 µM BAM15 | 02:32:50 | 76.11 |
| 14 | 184 | D02 | 2.5 µM BAM15 | 02:33:04 | 70.06 |
| 14 | 185 | D02 | 2.5 µM BAM15 | 02:33:18 | 64.30 |
| 14 | 186 | D02 | 2.5 µM BAM15 | 02:33:32 | 59.03 |
| 14 | 187 | D02 | 2.5 µM BAM15 | 02:33:46 | 54.00 |
| 14 | 188 | D02 | 2.5 µM BAM15 | 02:34:01 | 49.48 |
| 14 | 189 | D02 | 2.5 µM BAM15 | 02:34:15 | 45.12 |
| 14 | 190 | D02 | 2.5 µM BAM15 | 02:34:29 | 41.01 |
| 14 | 191 | D02 | 2.5 µM BAM15 | 02:34:43 | 37.22 |
| 14 | 192 | D02 | 2.5 µM BAM15 | 02:34:57 | 33.73 |
| 14 | 193 | D02 | 2.5 µM BAM15 | 02:35:12 | 30.80 |
| 15 | 194 | D02 | 2.5 µM BAM15 | 02:40:46 | 106.68 |
| 15 | 195 | D02 | 2.5 µM BAM15 | 02:41:00 | 91.84 |
| 15 | 196 | D02 | 2.5 µM BAM15 | 02:41:14 | 83.39 |
| 15 | 197 | D02 | 2.5 µM BAM15 | 02:41:28 | 76.56 |
| 15 | 198 | D02 | 2.5 µM BAM15 | 02:41:43 | 70.41 |
| 15 | 199 | D02 | 2.5 µM BAM15 | 02:41:57 | 64.85 |
| 15 | 200 | D02 | 2.5 µM BAM15 | 02:42:11 | 59.60 |
| 15 | 201 | D02 | 2.5 µM BAM15 | 02:42:25 | 54.71 |
| 15 | 202 | D02 | 2.5 µM BAM15 | 02:42:39 | 50.15 |
| 15 | 203 | D02 | 2.5 µM BAM15 | 02:42:53 | 45.85 |
| 15 | 204 | D02 | 2.5 µM BAM15 | 02:43:08 | 41.70 |
| 15 | 205 | D02 | 2.5 µM BAM15 | 02:43:22 | 38.01 |
| 15 | 206 | D02 | 2.5 µM BAM15 | 02:43:36 | 34.42 |
| 15 | 207 | D02 | 2.5 µM BAM15 | 02:43:50 | 31.39 |
| 16 | 208 | D02 | 2.5 µM BAM15 | 02:49:25 | 106.80 |
| 16 | 209 | D02 | 2.5 µM BAM15 | 02:49:40 | 92.10 |
| 16 | 210 | D02 | 2.5 µM BAM15 | 02:49:54 | 83.75 |
| 16 | 211 | D02 | 2.5 µM BAM15 | 02:50:08 | 76.94 |
| 16 | 212 | D02 | 2.5 µM BAM15 | 02:50:22 | 70.88 |
| 16 | 213 | D02 | 2.5 µM BAM15 | 02:50:36 | 65.31 |
| 16 | 214 | D02 | 2.5 µM BAM15 | 02:50:50 | 60.13 |
| 16 | 215 | D02 | 2.5 µM BAM15 | 02:51:05 | 55.22 |
| 16 | 216 | D02 | 2.5 µM BAM15 | 02:51:19 | 50.70 |
| 16 | 217 | D02 | 2.5 µM BAM15 | 02:51:33 | 46.44 |
| 16 | 218 | D02 | 2.5 µM BAM15 | 02:51:47 | 42.38 |
| 16 | 219 | D02 | 2.5 µM BAM15 | 02:52:01 | 38.65 |
| 16 | 220 | D02 | 2.5 µM BAM15 | 02:52:16 | 35.08 |
| 16 | 221 | D02 | 2.5 µM BAM15 | 02:52:30 | 31.97 |
| 1 | 0 | D03 | 2.5 µM BAM15 | 00:40:06 | 138.59 |
| 1 | 1 | D03 | 2.5 µM BAM15 | 00:40:20 | 134.82 |
| 1 | 2 | D03 | 2.5 µM BAM15 | 00:40:34 | 132.89 |
| 1 | 3 | D03 | 2.5 µM BAM15 | 00:40:48 | 131.35 |
| 1 | 4 | D03 | 2.5 µM BAM15 | 00:41:02 | 129.80 |
| 1 | 5 | D03 | 2.5 µM BAM15 | 00:41:17 | 128.59 |
| 1 | 6 | D03 | 2.5 µM BAM15 | 00:41:31 | 127.40 |
| 1 | 7 | D03 | 2.5 µM BAM15 | 00:41:45 | 126.26 |
| 1 | 8 | D03 | 2.5 µM BAM15 | 00:41:59 | 125.21 |
| 1 | 9 | D03 | 2.5 µM BAM15 | 00:42:13 | 124.20 |
| 1 | 10 | D03 | 2.5 µM BAM15 | 00:42:28 | 123.18 |
| 1 | 11 | D03 | 2.5 µM BAM15 | 00:42:42 | 122.18 |
| 1 | 12 | D03 | 2.5 µM BAM15 | 00:42:56 | 121.27 |
| 1 | 13 | D03 | 2.5 µM BAM15 | 00:43:10 | 120.41 |
| 2 | 14 | D03 | 2.5 µM BAM15 | 00:48:43 | 139.79 |
| 2 | 15 | D03 | 2.5 µM BAM15 | 00:48:57 | 135.80 |
| 2 | 16 | D03 | 2.5 µM BAM15 | 00:49:12 | 133.88 |
| 2 | 17 | D03 | 2.5 µM BAM15 | 00:49:26 | 132.14 |
| 2 | 18 | D03 | 2.5 µM BAM15 | 00:49:40 | 130.69 |
| 2 | 19 | D03 | 2.5 µM BAM15 | 00:49:54 | 129.25 |
| 2 | 20 | D03 | 2.5 µM BAM15 | 00:50:08 | 128.10 |
| 2 | 21 | D03 | 2.5 µM BAM15 | 00:50:22 | 126.87 |
| 2 | 22 | D03 | 2.5 µM BAM15 | 00:50:37 | 125.90 |
| 2 | 23 | D03 | 2.5 µM BAM15 | 00:50:51 | 124.88 |
| 2 | 24 | D03 | 2.5 µM BAM15 | 00:51:05 | 123.85 |
| 2 | 25 | D03 | 2.5 µM BAM15 | 00:51:19 | 122.92 |
| 2 | 26 | D03 | 2.5 µM BAM15 | 00:51:33 | 122.02 |
| 2 | 27 | D03 | 2.5 µM BAM15 | 00:51:48 | 121.09 |
| 3 | 28 | D03 | 2.5 µM BAM15 | 00:57:21 | 139.80 |
| 3 | 29 | D03 | 2.5 µM BAM15 | 00:57:35 | 136.17 |
| 3 | 30 | D03 | 2.5 µM BAM15 | 00:57:49 | 134.16 |
| 3 | 31 | D03 | 2.5 µM BAM15 | 00:58:03 | 132.49 |
| 3 | 32 | D03 | 2.5 µM BAM15 | 00:58:17 | 131.14 |
| 3 | 33 | D03 | 2.5 µM BAM15 | 00:58:31 | 129.78 |
| 3 | 34 | D03 | 2.5 µM BAM15 | 00:58:46 | 128.59 |
| 3 | 35 | D03 | 2.5 µM BAM15 | 00:59:00 | 127.46 |
| 3 | 36 | D03 | 2.5 µM BAM15 | 00:59:14 | 126.28 |
| 3 | 37 | D03 | 2.5 µM BAM15 | 00:59:28 | 125.33 |
| 3 | 38 | D03 | 2.5 µM BAM15 | 00:59:42 | 124.31 |
| 3 | 39 | D03 | 2.5 µM BAM15 | 00:59:56 | 123.41 |
| 3 | 40 | D03 | 2.5 µM BAM15 | 01:00:11 | 122.47 |
| 3 | 41 | D03 | 2.5 µM BAM15 | 01:00:25 | 121.67 |
| 4 | 42 | D03 | 2.5 µM BAM15 | 01:06:04 | 134.64 |
| 4 | 43 | D03 | 2.5 µM BAM15 | 01:06:18 | 126.75 |
| 4 | 44 | D03 | 2.5 µM BAM15 | 01:06:33 | 122.65 |
| 4 | 45 | D03 | 2.5 µM BAM15 | 01:06:47 | 119.53 |
| 4 | 46 | D03 | 2.5 µM BAM15 | 01:07:01 | 116.73 |
| 4 | 47 | D03 | 2.5 µM BAM15 | 01:07:15 | 114.03 |
| 4 | 48 | D03 | 2.5 µM BAM15 | 01:07:29 | 111.45 |
| 4 | 49 | D03 | 2.5 µM BAM15 | 01:07:43 | 108.97 |
| 4 | 50 | D03 | 2.5 µM BAM15 | 01:07:58 | 106.54 |
| 4 | 51 | D03 | 2.5 µM BAM15 | 01:08:12 | 104.17 |
| 4 | 52 | D03 | 2.5 µM BAM15 | 01:08:26 | 101.88 |
| 4 | 53 | D03 | 2.5 µM BAM15 | 01:08:40 | 99.64 |
| 4 | 54 | D03 | 2.5 µM BAM15 | 01:08:54 | 97.36 |
| 4 | 55 | D03 | 2.5 µM BAM15 | 01:09:09 | 95.18 |
| 5 | 56 | D03 | 2.5 µM BAM15 | 01:14:42 | 129.84 |
| 5 | 57 | D03 | 2.5 µM BAM15 | 01:14:56 | 120.08 |
| 5 | 58 | D03 | 2.5 µM BAM15 | 01:15:10 | 115.46 |
| 5 | 59 | D03 | 2.5 µM BAM15 | 01:15:25 | 112.02 |
| 5 | 60 | D03 | 2.5 µM BAM15 | 01:15:39 | 108.90 |
| 5 | 61 | D03 | 2.5 µM BAM15 | 01:15:53 | 105.94 |
| 5 | 62 | D03 | 2.5 µM BAM15 | 01:16:07 | 103.25 |
| 5 | 63 | D03 | 2.5 µM BAM15 | 01:16:21 | 100.58 |
| 5 | 64 | D03 | 2.5 µM BAM15 | 01:16:35 | 98.12 |
| 5 | 65 | D03 | 2.5 µM BAM15 | 01:16:50 | 95.68 |
| 5 | 66 | D03 | 2.5 µM BAM15 | 01:17:04 | 93.34 |
| 5 | 67 | D03 | 2.5 µM BAM15 | 01:17:18 | 91.02 |
| 5 | 68 | D03 | 2.5 µM BAM15 | 01:17:32 | 88.74 |
| 5 | 69 | D03 | 2.5 µM BAM15 | 01:17:46 | 86.55 |
| 6 | 70 | D03 | 2.5 µM BAM15 | 01:23:20 | 127.61 |
| 6 | 71 | D03 | 2.5 µM BAM15 | 01:23:34 | 117.02 |
| 6 | 72 | D03 | 2.5 µM BAM15 | 01:23:48 | 112.33 |
| 6 | 73 | D03 | 2.5 µM BAM15 | 01:24:02 | 108.55 |
| 6 | 74 | D03 | 2.5 µM BAM15 | 01:24:16 | 105.31 |
| 6 | 75 | D03 | 2.5 µM BAM15 | 01:24:30 | 102.22 |
| 6 | 76 | D03 | 2.5 µM BAM15 | 01:24:44 | 99.49 |
| 6 | 77 | D03 | 2.5 µM BAM15 | 01:24:59 | 96.76 |
| 6 | 78 | D03 | 2.5 µM BAM15 | 01:25:13 | 94.14 |
| 6 | 79 | D03 | 2.5 µM BAM15 | 01:25:27 | 91.70 |
| 6 | 80 | D03 | 2.5 µM BAM15 | 01:25:41 | 89.24 |
| 6 | 81 | D03 | 2.5 µM BAM15 | 01:25:55 | 86.95 |
| 6 | 82 | D03 | 2.5 µM BAM15 | 01:26:10 | 84.67 |
| 6 | 83 | D03 | 2.5 µM BAM15 | 01:26:24 | 82.47 |
| 7 | 84 | D03 | 2.5 µM BAM15 | 01:31:58 | 126.13 |
| 7 | 85 | D03 | 2.5 µM BAM15 | 01:32:12 | 115.56 |
| 7 | 86 | D03 | 2.5 µM BAM15 | 01:32:26 | 110.61 |
| 7 | 87 | D03 | 2.5 µM BAM15 | 01:32:40 | 106.84 |
| 7 | 88 | D03 | 2.5 µM BAM15 | 01:32:54 | 103.42 |
| 7 | 89 | D03 | 2.5 µM BAM15 | 01:33:08 | 100.37 |
| 7 | 90 | D03 | 2.5 µM BAM15 | 01:33:23 | 97.46 |
| 7 | 91 | D03 | 2.5 µM BAM15 | 01:33:37 | 94.65 |
| 7 | 92 | D03 | 2.5 µM BAM15 | 01:33:51 | 92.09 |
| 7 | 93 | D03 | 2.5 µM BAM15 | 01:34:05 | 89.63 |
| 7 | 94 | D03 | 2.5 µM BAM15 | 01:34:19 | 87.17 |
| 7 | 95 | D03 | 2.5 µM BAM15 | 01:34:33 | 84.92 |
| 7 | 96 | D03 | 2.5 µM BAM15 | 01:34:48 | 82.60 |
| 7 | 97 | D03 | 2.5 µM BAM15 | 01:35:02 | 80.49 |
| 8 | 98 | D03 | 2.5 µM BAM15 | 01:40:36 | 125.45 |
| 8 | 99 | D03 | 2.5 µM BAM15 | 01:40:50 | 114.81 |
| 8 | 100 | D03 | 2.5 µM BAM15 | 01:41:04 | 109.76 |
| 8 | 101 | D03 | 2.5 µM BAM15 | 01:41:18 | 105.84 |
| 8 | 102 | D03 | 2.5 µM BAM15 | 01:41:32 | 102.44 |
| 8 | 103 | D03 | 2.5 µM BAM15 | 01:41:46 | 99.34 |
| 8 | 104 | D03 | 2.5 µM BAM15 | 01:42:00 | 96.47 |
| 8 | 105 | D03 | 2.5 µM BAM15 | 01:42:15 | 93.74 |
| 8 | 106 | D03 | 2.5 µM BAM15 | 01:42:29 | 91.08 |
| 8 | 107 | D03 | 2.5 µM BAM15 | 01:42:43 | 88.59 |
| 8 | 108 | D03 | 2.5 µM BAM15 | 01:42:57 | 86.17 |
| 8 | 109 | D03 | 2.5 µM BAM15 | 01:43:11 | 83.90 |
| 8 | 110 | D03 | 2.5 µM BAM15 | 01:43:26 | 81.61 |
| 8 | 111 | D03 | 2.5 µM BAM15 | 01:43:40 | 79.54 |
| 9 | 112 | D03 | 2.5 µM BAM15 | 01:49:14 | 125.11 |
| 9 | 113 | D03 | 2.5 µM BAM15 | 01:49:28 | 114.43 |
| 9 | 114 | D03 | 2.5 µM BAM15 | 01:49:42 | 109.38 |
| 9 | 115 | D03 | 2.5 µM BAM15 | 01:49:56 | 105.47 |
| 9 | 116 | D03 | 2.5 µM BAM15 | 01:50:10 | 102.01 |
| 9 | 117 | D03 | 2.5 µM BAM15 | 01:50:24 | 98.98 |
| 9 | 118 | D03 | 2.5 µM BAM15 | 01:50:38 | 96.10 |
| 9 | 119 | D03 | 2.5 µM BAM15 | 01:50:53 | 93.25 |
| 9 | 120 | D03 | 2.5 µM BAM15 | 01:51:07 | 90.65 |
| 9 | 121 | D03 | 2.5 µM BAM15 | 01:51:21 | 88.17 |
| 9 | 122 | D03 | 2.5 µM BAM15 | 01:51:35 | 85.81 |
| 9 | 123 | D03 | 2.5 µM BAM15 | 01:51:50 | 83.57 |
| 9 | 124 | D03 | 2.5 µM BAM15 | 01:52:04 | 81.33 |
| 9 | 125 | D03 | 2.5 µM BAM15 | 01:52:18 | 79.23 |
| 10 | 126 | D03 | 2.5 µM BAM15 | 01:57:52 | 125.26 |
| 10 | 127 | D03 | 2.5 µM BAM15 | 01:58:06 | 114.96 |
| 10 | 128 | D03 | 2.5 µM BAM15 | 01:58:20 | 109.88 |
| 10 | 129 | D03 | 2.5 µM BAM15 | 01:58:34 | 106.07 |
| 10 | 130 | D03 | 2.5 µM BAM15 | 01:58:48 | 102.66 |
| 10 | 131 | D03 | 2.5 µM BAM15 | 01:59:03 | 99.43 |
| 10 | 132 | D03 | 2.5 µM BAM15 | 01:59:17 | 96.55 |
| 10 | 133 | D03 | 2.5 µM BAM15 | 01:59:31 | 93.72 |
| 10 | 134 | D03 | 2.5 µM BAM15 | 01:59:45 | 91.27 |
| 10 | 135 | D03 | 2.5 µM BAM15 | 01:59:59 | 88.69 |
| 10 | 136 | D03 | 2.5 µM BAM15 | 02:00:14 | 86.31 |
| 10 | 137 | D03 | 2.5 µM BAM15 | 02:00:28 | 84.06 |
| 10 | 138 | D03 | 2.5 µM BAM15 | 02:00:42 | 81.86 |
| 10 | 139 | D03 | 2.5 µM BAM15 | 02:00:56 | 79.79 |
| 11 | 140 | D03 | 2.5 µM BAM15 | 02:06:30 | 127.45 |
| 11 | 141 | D03 | 2.5 µM BAM15 | 02:06:44 | 118.86 |
| 11 | 142 | D03 | 2.5 µM BAM15 | 02:06:58 | 114.05 |
| 11 | 143 | D03 | 2.5 µM BAM15 | 02:07:12 | 110.32 |
| 11 | 144 | D03 | 2.5 µM BAM15 | 02:07:26 | 106.92 |
| 11 | 145 | D03 | 2.5 µM BAM15 | 02:07:41 | 103.65 |
| 11 | 146 | D03 | 2.5 µM BAM15 | 02:07:55 | 100.75 |
| 11 | 147 | D03 | 2.5 µM BAM15 | 02:08:09 | 97.95 |
| 11 | 148 | D03 | 2.5 µM BAM15 | 02:08:23 | 95.27 |
| 11 | 149 | D03 | 2.5 µM BAM15 | 02:08:37 | 92.72 |
| 11 | 150 | D03 | 2.5 µM BAM15 | 02:08:52 | 90.24 |
| 11 | 151 | D03 | 2.5 µM BAM15 | 02:09:06 | 87.83 |
| 11 | 152 | D03 | 2.5 µM BAM15 | 02:09:20 | 85.61 |
| 11 | 153 | D03 | 2.5 µM BAM15 | 02:09:34 | 83.42 |
| 12 | 154 | D03 | 2.5 µM BAM15 | 02:15:08 | 128.04 |
| 12 | 155 | D03 | 2.5 µM BAM15 | 02:15:22 | 120.05 |
| 12 | 156 | D03 | 2.5 µM BAM15 | 02:15:37 | 115.17 |
| 12 | 157 | D03 | 2.5 µM BAM15 | 02:15:53 | 111.14 |
| 12 | 158 | D03 | 2.5 µM BAM15 | 02:16:08 | 107.71 |
| 12 | 159 | D03 | 2.5 µM BAM15 | 02:16:22 | 104.73 |
| 12 | 160 | D03 | 2.5 µM BAM15 | 02:16:36 | 101.81 |
| 12 | 161 | D03 | 2.5 µM BAM15 | 02:16:50 | 99.12 |
| 12 | 162 | D03 | 2.5 µM BAM15 | 02:17:04 | 96.46 |
| 12 | 163 | D03 | 2.5 µM BAM15 | 02:17:18 | 93.96 |
| 12 | 164 | D03 | 2.5 µM BAM15 | 02:17:33 | 91.48 |
| 12 | 165 | D03 | 2.5 µM BAM15 | 02:17:47 | 89.18 |
| 12 | 166 | D03 | 2.5 µM BAM15 | 02:18:01 | 86.95 |
| 13 | 167 | D03 | 2.5 µM BAM15 | 02:23:39 | 125.88 |
| 13 | 168 | D03 | 2.5 µM BAM15 | 02:23:54 | 119.27 |
| 13 | 169 | D03 | 2.5 µM BAM15 | 02:24:08 | 115.13 |
| 13 | 170 | D03 | 2.5 µM BAM15 | 02:24:22 | 111.56 |
| 13 | 171 | D03 | 2.5 µM BAM15 | 02:24:36 | 108.34 |
| 13 | 172 | D03 | 2.5 µM BAM15 | 02:24:51 | 105.16 |
| 13 | 173 | D03 | 2.5 µM BAM15 | 02:25:07 | 102.02 |
| 13 | 174 | D03 | 2.5 µM BAM15 | 02:25:22 | 99.29 |
| 13 | 175 | D03 | 2.5 µM BAM15 | 02:25:36 | 96.79 |
| 13 | 176 | D03 | 2.5 µM BAM15 | 02:25:50 | 94.33 |
| 13 | 177 | D03 | 2.5 µM BAM15 | 02:26:04 | 91.99 |
| 13 | 178 | D03 | 2.5 µM BAM15 | 02:26:18 | 89.67 |
| 13 | 179 | D03 | 2.5 µM BAM15 | 02:26:33 | 87.50 |
| 14 | 180 | D03 | 2.5 µM BAM15 | 02:32:07 | 128.72 |
| 14 | 181 | D03 | 2.5 µM BAM15 | 02:32:21 | 120.89 |
| 14 | 182 | D03 | 2.5 µM BAM15 | 02:32:35 | 116.55 |
| 14 | 183 | D03 | 2.5 µM BAM15 | 02:32:50 | 112.89 |
| 14 | 184 | D03 | 2.5 µM BAM15 | 02:33:04 | 109.74 |
| 14 | 185 | D03 | 2.5 µM BAM15 | 02:33:18 | 106.64 |
| 14 | 186 | D03 | 2.5 µM BAM15 | 02:33:32 | 103.81 |
| 14 | 187 | D03 | 2.5 µM BAM15 | 02:33:46 | 101.12 |
| 14 | 188 | D03 | 2.5 µM BAM15 | 02:34:01 | 98.60 |
| 14 | 189 | D03 | 2.5 µM BAM15 | 02:34:15 | 96.12 |
| 14 | 190 | D03 | 2.5 µM BAM15 | 02:34:29 | 93.82 |
| 14 | 191 | D03 | 2.5 µM BAM15 | 02:34:43 | 91.56 |
| 14 | 192 | D03 | 2.5 µM BAM15 | 02:34:57 | 89.29 |
| 14 | 193 | D03 | 2.5 µM BAM15 | 02:35:12 | 87.26 |
| 15 | 194 | D03 | 2.5 µM BAM15 | 02:40:46 | 128.98 |
| 15 | 195 | D03 | 2.5 µM BAM15 | 02:41:00 | 121.22 |
| 15 | 196 | D03 | 2.5 µM BAM15 | 02:41:14 | 116.92 |
| 15 | 197 | D03 | 2.5 µM BAM15 | 02:41:28 | 113.33 |
| 15 | 198 | D03 | 2.5 µM BAM15 | 02:41:43 | 110.08 |
| 15 | 199 | D03 | 2.5 µM BAM15 | 02:41:57 | 107.19 |
| 15 | 200 | D03 | 2.5 µM BAM15 | 02:42:11 | 104.40 |
| 15 | 201 | D03 | 2.5 µM BAM15 | 02:42:25 | 101.72 |
| 15 | 202 | D03 | 2.5 µM BAM15 | 02:42:39 | 99.23 |
| 15 | 203 | D03 | 2.5 µM BAM15 | 02:42:53 | 96.87 |
| 15 | 204 | D03 | 2.5 µM BAM15 | 02:43:08 | 94.48 |
| 15 | 205 | D03 | 2.5 µM BAM15 | 02:43:22 | 92.37 |
| 15 | 206 | D03 | 2.5 µM BAM15 | 02:43:36 | 90.09 |
| 15 | 207 | D03 | 2.5 µM BAM15 | 02:43:50 | 88.08 |
| 16 | 208 | D03 | 2.5 µM BAM15 | 02:49:25 | 129.14 |
| 16 | 209 | D03 | 2.5 µM BAM15 | 02:49:40 | 121.52 |
| 16 | 210 | D03 | 2.5 µM BAM15 | 02:49:54 | 117.21 |
| 16 | 211 | D03 | 2.5 µM BAM15 | 02:50:08 | 113.70 |
| 16 | 212 | D03 | 2.5 µM BAM15 | 02:50:22 | 110.59 |
| 16 | 213 | D03 | 2.5 µM BAM15 | 02:50:36 | 107.72 |
| 16 | 214 | D03 | 2.5 µM BAM15 | 02:50:50 | 104.94 |
| 16 | 215 | D03 | 2.5 µM BAM15 | 02:51:05 | 102.31 |
| 16 | 216 | D03 | 2.5 µM BAM15 | 02:51:19 | 99.83 |
| 16 | 217 | D03 | 2.5 µM BAM15 | 02:51:33 | 97.43 |
| 16 | 218 | D03 | 2.5 µM BAM15 | 02:51:47 | 95.18 |
| 16 | 219 | D03 | 2.5 µM BAM15 | 02:52:01 | 93.00 |
| 16 | 220 | D03 | 2.5 µM BAM15 | 02:52:16 | 90.83 |
| 16 | 221 | D03 | 2.5 µM BAM15 | 02:52:30 | 88.89 |
| 1 | 0 | D04 | 2.5 µM FCCP | 00:40:06 | 127.06 |
| 1 | 1 | D04 | 2.5 µM FCCP | 00:40:20 | 119.68 |
| 1 | 2 | D04 | 2.5 µM FCCP | 00:40:34 | 116.89 |
| 1 | 3 | D04 | 2.5 µM FCCP | 00:40:48 | 114.68 |
| 1 | 4 | D04 | 2.5 µM FCCP | 00:41:02 | 112.83 |
| 1 | 5 | D04 | 2.5 µM FCCP | 00:41:17 | 111.08 |
| 1 | 6 | D04 | 2.5 µM FCCP | 00:41:31 | 109.60 |
| 1 | 7 | D04 | 2.5 µM FCCP | 00:41:45 | 108.18 |
| 1 | 8 | D04 | 2.5 µM FCCP | 00:41:59 | 106.74 |
| 1 | 9 | D04 | 2.5 µM FCCP | 00:42:13 | 105.50 |
| 1 | 10 | D04 | 2.5 µM FCCP | 00:42:28 | 104.21 |
| 1 | 11 | D04 | 2.5 µM FCCP | 00:42:42 | 103.02 |
| 1 | 12 | D04 | 2.5 µM FCCP | 00:42:56 | 101.77 |
| 1 | 13 | D04 | 2.5 µM FCCP | 00:43:10 | 100.72 |
| 2 | 14 | D04 | 2.5 µM FCCP | 00:48:43 | 130.30 |
| 2 | 15 | D04 | 2.5 µM FCCP | 00:48:57 | 123.95 |
| 2 | 16 | D04 | 2.5 µM FCCP | 00:49:12 | 121.07 |
| 2 | 17 | D04 | 2.5 µM FCCP | 00:49:26 | 118.71 |
| 2 | 18 | D04 | 2.5 µM FCCP | 00:49:40 | 116.76 |
| 2 | 19 | D04 | 2.5 µM FCCP | 00:49:54 | 114.86 |
| 2 | 20 | D04 | 2.5 µM FCCP | 00:50:08 | 113.25 |
| 2 | 21 | D04 | 2.5 µM FCCP | 00:50:22 | 111.61 |
| 2 | 22 | D04 | 2.5 µM FCCP | 00:50:37 | 110.20 |
| 2 | 23 | D04 | 2.5 µM FCCP | 00:50:51 | 108.78 |
| 2 | 24 | D04 | 2.5 µM FCCP | 00:51:05 | 107.38 |
| 2 | 25 | D04 | 2.5 µM FCCP | 00:51:19 | 106.17 |
| 2 | 26 | D04 | 2.5 µM FCCP | 00:51:33 | 104.82 |
| 2 | 27 | D04 | 2.5 µM FCCP | 00:51:48 | 103.70 |
| 3 | 28 | D04 | 2.5 µM FCCP | 00:57:21 | 131.92 |
| 3 | 29 | D04 | 2.5 µM FCCP | 00:57:35 | 126.49 |
| 3 | 30 | D04 | 2.5 µM FCCP | 00:57:49 | 123.58 |
| 3 | 31 | D04 | 2.5 µM FCCP | 00:58:03 | 121.30 |
| 3 | 32 | D04 | 2.5 µM FCCP | 00:58:17 | 119.36 |
| 3 | 33 | D04 | 2.5 µM FCCP | 00:58:31 | 117.43 |
| 3 | 34 | D04 | 2.5 µM FCCP | 00:58:46 | 115.78 |
| 3 | 35 | D04 | 2.5 µM FCCP | 00:59:00 | 114.20 |
| 3 | 36 | D04 | 2.5 µM FCCP | 00:59:14 | 112.65 |
| 3 | 37 | D04 | 2.5 µM FCCP | 00:59:28 | 111.30 |
| 3 | 38 | D04 | 2.5 µM FCCP | 00:59:42 | 109.87 |
| 3 | 39 | D04 | 2.5 µM FCCP | 00:59:56 | 108.64 |
| 3 | 40 | D04 | 2.5 µM FCCP | 01:00:11 | 107.34 |
| 3 | 41 | D04 | 2.5 µM FCCP | 01:00:25 | 106.13 |
| 4 | 42 | D04 | 2.5 µM FCCP | 01:06:04 | 130.56 |
| 4 | 43 | D04 | 2.5 µM FCCP | 01:06:18 | 123.30 |
| 4 | 44 | D04 | 2.5 µM FCCP | 01:06:33 | 119.72 |
| 4 | 45 | D04 | 2.5 µM FCCP | 01:06:47 | 116.91 |
| 4 | 46 | D04 | 2.5 µM FCCP | 01:07:01 | 114.40 |
| 4 | 47 | D04 | 2.5 µM FCCP | 01:07:15 | 111.98 |
| 4 | 48 | D04 | 2.5 µM FCCP | 01:07:29 | 109.62 |
| 4 | 49 | D04 | 2.5 µM FCCP | 01:07:43 | 107.24 |
| 4 | 50 | D04 | 2.5 µM FCCP | 01:07:58 | 104.91 |
| 4 | 51 | D04 | 2.5 µM FCCP | 01:08:12 | 102.43 |
| 4 | 52 | D04 | 2.5 µM FCCP | 01:08:26 | 99.82 |
| 4 | 53 | D04 | 2.5 µM FCCP | 01:08:40 | 97.09 |
| 4 | 54 | D04 | 2.5 µM FCCP | 01:08:54 | 94.15 |
| 4 | 55 | D04 | 2.5 µM FCCP | 01:09:09 | 91.22 |
| 5 | 56 | D04 | 2.5 µM FCCP | 01:14:42 | 126.51 |
| 5 | 57 | D04 | 2.5 µM FCCP | 01:14:56 | 117.20 |
| 5 | 58 | D04 | 2.5 µM FCCP | 01:15:10 | 112.78 |
| 5 | 59 | D04 | 2.5 µM FCCP | 01:15:25 | 109.15 |
| 5 | 60 | D04 | 2.5 µM FCCP | 01:15:39 | 105.95 |
| 5 | 61 | D04 | 2.5 µM FCCP | 01:15:53 | 102.69 |
| 5 | 62 | D04 | 2.5 µM FCCP | 01:16:07 | 99.58 |
| 5 | 63 | D04 | 2.5 µM FCCP | 01:16:21 | 96.31 |
| 5 | 64 | D04 | 2.5 µM FCCP | 01:16:35 | 93.10 |
| 5 | 65 | D04 | 2.5 µM FCCP | 01:16:50 | 89.64 |
| 5 | 66 | D04 | 2.5 µM FCCP | 01:17:04 | 86.02 |
| 5 | 67 | D04 | 2.5 µM FCCP | 01:17:18 | 82.47 |
| 5 | 68 | D04 | 2.5 µM FCCP | 01:17:32 | 78.59 |
| 5 | 69 | D04 | 2.5 µM FCCP | 01:17:46 | 74.78 |
| 6 | 70 | D04 | 2.5 µM FCCP | 01:23:20 | 123.67 |
| 6 | 71 | D04 | 2.5 µM FCCP | 01:23:34 | 113.34 |
| 6 | 72 | D04 | 2.5 µM FCCP | 01:23:48 | 108.56 |
| 6 | 73 | D04 | 2.5 µM FCCP | 01:24:02 | 104.55 |
| 6 | 74 | D04 | 2.5 µM FCCP | 01:24:16 | 101.04 |
| 6 | 75 | D04 | 2.5 µM FCCP | 01:24:30 | 97.46 |
| 6 | 76 | D04 | 2.5 µM FCCP | 01:24:44 | 94.09 |
| 6 | 77 | D04 | 2.5 µM FCCP | 01:24:59 | 90.47 |
| 6 | 78 | D04 | 2.5 µM FCCP | 01:25:13 | 86.88 |
| 6 | 79 | D04 | 2.5 µM FCCP | 01:25:27 | 83.20 |
| 6 | 80 | D04 | 2.5 µM FCCP | 01:25:41 | 79.31 |
| 6 | 81 | D04 | 2.5 µM FCCP | 01:25:55 | 75.53 |
| 6 | 82 | D04 | 2.5 µM FCCP | 01:26:10 | 71.40 |
| 6 | 83 | D04 | 2.5 µM FCCP | 01:26:24 | 67.37 |
| 7 | 84 | D04 | 2.5 µM FCCP | 01:31:58 | 121.66 |
| 7 | 85 | D04 | 2.5 µM FCCP | 01:32:12 | 110.84 |
| 7 | 86 | D04 | 2.5 µM FCCP | 01:32:26 | 105.60 |
| 7 | 87 | D04 | 2.5 µM FCCP | 01:32:40 | 101.54 |
| 7 | 88 | D04 | 2.5 µM FCCP | 01:32:54 | 97.62 |
| 7 | 89 | D04 | 2.5 µM FCCP | 01:33:08 | 93.88 |
| 7 | 90 | D04 | 2.5 µM FCCP | 01:33:23 | 90.12 |
| 7 | 91 | D04 | 2.5 µM FCCP | 01:33:37 | 86.33 |
| 7 | 92 | D04 | 2.5 µM FCCP | 01:33:51 | 82.44 |
| 7 | 93 | D04 | 2.5 µM FCCP | 01:34:05 | 78.55 |
| 7 | 94 | D04 | 2.5 µM FCCP | 01:34:19 | 74.44 |
| 7 | 95 | D04 | 2.5 µM FCCP | 01:34:33 | 70.46 |
| 7 | 96 | D04 | 2.5 µM FCCP | 01:34:48 | 66.22 |
| 7 | 97 | D04 | 2.5 µM FCCP | 01:35:02 | 62.08 |
| 8 | 98 | D04 | 2.5 µM FCCP | 01:40:36 | 120.30 |
| 8 | 99 | D04 | 2.5 µM FCCP | 01:40:50 | 108.93 |
| 8 | 100 | D04 | 2.5 µM FCCP | 01:41:04 | 103.49 |
| 8 | 101 | D04 | 2.5 µM FCCP | 01:41:18 | 98.99 |
| 8 | 102 | D04 | 2.5 µM FCCP | 01:41:32 | 94.89 |
| 8 | 103 | D04 | 2.5 µM FCCP | 01:41:46 | 90.88 |
| 8 | 104 | D04 | 2.5 µM FCCP | 01:42:00 | 86.93 |
| 8 | 105 | D04 | 2.5 µM FCCP | 01:42:15 | 82.86 |
| 8 | 106 | D04 | 2.5 µM FCCP | 01:42:29 | 78.78 |
| 8 | 107 | D04 | 2.5 µM FCCP | 01:42:43 | 74.71 |
| 8 | 108 | D04 | 2.5 µM FCCP | 01:42:57 | 70.46 |
| 8 | 109 | D04 | 2.5 µM FCCP | 01:43:11 | 66.26 |
| 8 | 110 | D04 | 2.5 µM FCCP | 01:43:26 | 61.93 |
| 8 | 111 | D04 | 2.5 µM FCCP | 01:43:40 | 57.74 |
| 9 | 112 | D04 | 2.5 µM FCCP | 01:49:14 | 119.38 |
| 9 | 113 | D04 | 2.5 µM FCCP | 01:49:28 | 107.64 |
| 9 | 114 | D04 | 2.5 µM FCCP | 01:49:42 | 101.90 |
| 9 | 115 | D04 | 2.5 µM FCCP | 01:49:56 | 97.24 |
| 9 | 116 | D04 | 2.5 µM FCCP | 01:50:10 | 92.92 |
| 9 | 117 | D04 | 2.5 µM FCCP | 01:50:24 | 88.74 |
| 9 | 118 | D04 | 2.5 µM FCCP | 01:50:38 | 84.55 |
| 9 | 119 | D04 | 2.5 µM FCCP | 01:50:53 | 80.26 |
| 9 | 120 | D04 | 2.5 µM FCCP | 01:51:07 | 76.08 |
| 9 | 121 | D04 | 2.5 µM FCCP | 01:51:21 | 71.80 |
| 9 | 122 | D04 | 2.5 µM FCCP | 01:51:35 | 67.47 |
| 9 | 123 | D04 | 2.5 µM FCCP | 01:51:50 | 63.18 |
| 9 | 124 | D04 | 2.5 µM FCCP | 01:52:04 | 58.82 |
| 9 | 125 | D04 | 2.5 µM FCCP | 01:52:18 | 54.52 |
| 10 | 126 | D04 | 2.5 µM FCCP | 01:57:52 | 118.54 |
| 10 | 127 | D04 | 2.5 µM FCCP | 01:58:06 | 106.71 |
| 10 | 128 | D04 | 2.5 µM FCCP | 01:58:20 | 100.78 |
| 10 | 129 | D04 | 2.5 µM FCCP | 01:58:34 | 96.07 |
| 10 | 130 | D04 | 2.5 µM FCCP | 01:58:48 | 91.62 |
| 10 | 131 | D04 | 2.5 µM FCCP | 01:59:03 | 87.19 |
| 10 | 132 | D04 | 2.5 µM FCCP | 01:59:17 | 82.94 |
| 10 | 133 | D04 | 2.5 µM FCCP | 01:59:31 | 78.64 |
| 10 | 134 | D04 | 2.5 µM FCCP | 01:59:45 | 74.43 |
| 10 | 135 | D04 | 2.5 µM FCCP | 01:59:59 | 70.02 |
| 10 | 136 | D04 | 2.5 µM FCCP | 02:00:14 | 65.66 |
| 10 | 137 | D04 | 2.5 µM FCCP | 02:00:28 | 61.36 |
| 10 | 138 | D04 | 2.5 µM FCCP | 02:00:42 | 56.92 |
| 10 | 139 | D04 | 2.5 µM FCCP | 02:00:56 | 52.61 |
| 11 | 140 | D04 | 2.5 µM FCCP | 02:06:30 | 117.98 |
| 11 | 141 | D04 | 2.5 µM FCCP | 02:06:44 | 107.60 |
| 11 | 142 | D04 | 2.5 µM FCCP | 02:06:58 | 102.35 |
| 11 | 143 | D04 | 2.5 µM FCCP | 02:07:12 | 97.85 |
| 11 | 144 | D04 | 2.5 µM FCCP | 02:07:26 | 93.85 |
| 11 | 145 | D04 | 2.5 µM FCCP | 02:07:41 | 89.66 |
| 11 | 146 | D04 | 2.5 µM FCCP | 02:07:55 | 85.54 |
| 11 | 147 | D04 | 2.5 µM FCCP | 02:08:09 | 81.37 |
| 11 | 148 | D04 | 2.5 µM FCCP | 02:08:23 | 77.20 |
| 11 | 149 | D04 | 2.5 µM FCCP | 02:08:37 | 72.95 |
| 11 | 150 | D04 | 2.5 µM FCCP | 02:08:52 | 68.61 |
| 11 | 151 | D04 | 2.5 µM FCCP | 02:09:06 | 64.27 |
| 11 | 152 | D04 | 2.5 µM FCCP | 02:09:20 | 59.93 |
| 11 | 153 | D04 | 2.5 µM FCCP | 02:09:34 | 55.58 |
| 12 | 154 | D04 | 2.5 µM FCCP | 02:15:08 | 118.74 |
| 12 | 155 | D04 | 2.5 µM FCCP | 02:15:22 | 109.36 |
| 12 | 156 | D04 | 2.5 µM FCCP | 02:15:37 | 104.19 |
| 12 | 157 | D04 | 2.5 µM FCCP | 02:15:53 | 99.50 |
| 12 | 158 | D04 | 2.5 µM FCCP | 02:16:08 | 95.38 |
| 12 | 159 | D04 | 2.5 µM FCCP | 02:16:22 | 91.53 |
| 12 | 160 | D04 | 2.5 µM FCCP | 02:16:36 | 87.59 |
| 12 | 161 | D04 | 2.5 µM FCCP | 02:16:50 | 83.57 |
| 12 | 162 | D04 | 2.5 µM FCCP | 02:17:04 | 79.47 |
| 12 | 163 | D04 | 2.5 µM FCCP | 02:17:18 | 75.25 |
| 12 | 164 | D04 | 2.5 µM FCCP | 02:17:33 | 71.08 |
| 12 | 165 | D04 | 2.5 µM FCCP | 02:17:47 | 66.84 |
| 12 | 166 | D04 | 2.5 µM FCCP | 02:18:01 | 62.49 |
| 13 | 167 | D04 | 2.5 µM FCCP | 02:23:39 | 115.86 |
| 13 | 168 | D04 | 2.5 µM FCCP | 02:23:54 | 108.58 |
| 13 | 169 | D04 | 2.5 µM FCCP | 02:24:08 | 103.95 |
| 13 | 170 | D04 | 2.5 µM FCCP | 02:24:22 | 99.87 |
| 13 | 171 | D04 | 2.5 µM FCCP | 02:24:36 | 95.92 |
| 13 | 172 | D04 | 2.5 µM FCCP | 02:24:51 | 91.69 |
| 13 | 173 | D04 | 2.5 µM FCCP | 02:25:07 | 87.31 |
| 13 | 174 | D04 | 2.5 µM FCCP | 02:25:22 | 83.19 |
| 13 | 175 | D04 | 2.5 µM FCCP | 02:25:36 | 79.17 |
| 13 | 176 | D04 | 2.5 µM FCCP | 02:25:50 | 75.02 |
| 13 | 177 | D04 | 2.5 µM FCCP | 02:26:04 | 70.87 |
| 13 | 178 | D04 | 2.5 µM FCCP | 02:26:18 | 66.65 |
| 13 | 179 | D04 | 2.5 µM FCCP | 02:26:33 | 62.40 |
| 14 | 180 | D04 | 2.5 µM FCCP | 02:32:07 | 119.29 |
| 14 | 181 | D04 | 2.5 µM FCCP | 02:32:21 | 109.98 |
| 14 | 182 | D04 | 2.5 µM FCCP | 02:32:35 | 105.13 |
| 14 | 183 | D04 | 2.5 µM FCCP | 02:32:50 | 100.91 |
| 14 | 184 | D04 | 2.5 µM FCCP | 02:33:04 | 96.99 |
| 14 | 185 | D04 | 2.5 µM FCCP | 02:33:18 | 92.90 |
| 14 | 186 | D04 | 2.5 µM FCCP | 02:33:32 | 88.92 |
| 14 | 187 | D04 | 2.5 µM FCCP | 02:33:46 | 84.83 |
| 14 | 188 | D04 | 2.5 µM FCCP | 02:34:01 | 80.82 |
| 14 | 189 | D04 | 2.5 µM FCCP | 02:34:15 | 76.78 |
| 14 | 190 | D04 | 2.5 µM FCCP | 02:34:29 | 72.64 |
| 14 | 191 | D04 | 2.5 µM FCCP | 02:34:43 | 68.48 |
| 14 | 192 | D04 | 2.5 µM FCCP | 02:34:57 | 64.21 |
| 14 | 193 | D04 | 2.5 µM FCCP | 02:35:12 | 60.17 |
| 15 | 194 | D04 | 2.5 µM FCCP | 02:40:46 | 119.04 |
| 15 | 195 | D04 | 2.5 µM FCCP | 02:41:00 | 109.72 |
| 15 | 196 | D04 | 2.5 µM FCCP | 02:41:14 | 104.68 |
| 15 | 197 | D04 | 2.5 µM FCCP | 02:41:28 | 100.43 |
| 15 | 198 | D04 | 2.5 µM FCCP | 02:41:43 | 96.34 |
| 15 | 199 | D04 | 2.5 µM FCCP | 02:41:57 | 92.38 |
| 15 | 200 | D04 | 2.5 µM FCCP | 02:42:11 | 88.39 |
| 15 | 201 | D04 | 2.5 µM FCCP | 02:42:25 | 84.27 |
| 15 | 202 | D04 | 2.5 µM FCCP | 02:42:39 | 80.28 |
| 15 | 203 | D04 | 2.5 µM FCCP | 02:42:53 | 76.19 |
| 15 | 204 | D04 | 2.5 µM FCCP | 02:43:08 | 71.95 |
| 15 | 205 | D04 | 2.5 µM FCCP | 02:43:22 | 67.98 |
| 15 | 206 | D04 | 2.5 µM FCCP | 02:43:36 | 63.71 |
| 15 | 207 | D04 | 2.5 µM FCCP | 02:43:50 | 59.71 |
| 16 | 208 | D04 | 2.5 µM FCCP | 02:49:25 | 119.10 |
| 16 | 209 | D04 | 2.5 µM FCCP | 02:49:40 | 109.81 |
| 16 | 210 | D04 | 2.5 µM FCCP | 02:49:54 | 104.76 |
| 16 | 211 | D04 | 2.5 µM FCCP | 02:50:08 | 100.37 |
| 16 | 212 | D04 | 2.5 µM FCCP | 02:50:22 | 96.36 |
| 16 | 213 | D04 | 2.5 µM FCCP | 02:50:36 | 92.27 |
| 16 | 214 | D04 | 2.5 µM FCCP | 02:50:50 | 88.26 |
| 16 | 215 | D04 | 2.5 µM FCCP | 02:51:05 | 84.18 |
| 16 | 216 | D04 | 2.5 µM FCCP | 02:51:19 | 80.13 |
| 16 | 217 | D04 | 2.5 µM FCCP | 02:51:33 | 76.06 |
| 16 | 218 | D04 | 2.5 µM FCCP | 02:51:47 | 71.96 |
| 16 | 219 | D04 | 2.5 µM FCCP | 02:52:01 | 67.89 |
| 16 | 220 | D04 | 2.5 µM FCCP | 02:52:16 | 63.74 |
| 16 | 221 | D04 | 2.5 µM FCCP | 02:52:30 | 59.73 |
| 1 | 0 | D05 | 2.5 µM FCCP | 00:40:06 | 133.66 |
| 1 | 1 | D05 | 2.5 µM FCCP | 00:40:20 | 126.04 |
| 1 | 2 | D05 | 2.5 µM FCCP | 00:40:34 | 123.24 |
| 1 | 3 | D05 | 2.5 µM FCCP | 00:40:48 | 121.23 |
| 1 | 4 | D05 | 2.5 µM FCCP | 00:41:02 | 119.52 |
| 1 | 5 | D05 | 2.5 µM FCCP | 00:41:17 | 117.90 |
| 1 | 6 | D05 | 2.5 µM FCCP | 00:41:31 | 116.56 |
| 1 | 7 | D05 | 2.5 µM FCCP | 00:41:45 | 115.33 |
| 1 | 8 | D05 | 2.5 µM FCCP | 00:41:59 | 113.97 |
| 1 | 9 | D05 | 2.5 µM FCCP | 00:42:13 | 112.83 |
| 1 | 10 | D05 | 2.5 µM FCCP | 00:42:28 | 111.73 |
| 1 | 11 | D05 | 2.5 µM FCCP | 00:42:42 | 110.56 |
| 1 | 12 | D05 | 2.5 µM FCCP | 00:42:56 | 109.56 |
| 1 | 13 | D05 | 2.5 µM FCCP | 00:43:10 | 108.56 |
| 2 | 14 | D05 | 2.5 µM FCCP | 00:48:43 | 137.05 |
| 2 | 15 | D05 | 2.5 µM FCCP | 00:48:57 | 130.38 |
| 2 | 16 | D05 | 2.5 µM FCCP | 00:49:12 | 127.49 |
| 2 | 17 | D05 | 2.5 µM FCCP | 00:49:26 | 125.09 |
| 2 | 18 | D05 | 2.5 µM FCCP | 00:49:40 | 123.25 |
| 2 | 19 | D05 | 2.5 µM FCCP | 00:49:54 | 121.54 |
| 2 | 20 | D05 | 2.5 µM FCCP | 00:50:08 | 119.99 |
| 2 | 21 | D05 | 2.5 µM FCCP | 00:50:22 | 118.51 |
| 2 | 22 | D05 | 2.5 µM FCCP | 00:50:37 | 117.22 |
| 2 | 23 | D05 | 2.5 µM FCCP | 00:50:51 | 115.92 |
| 2 | 24 | D05 | 2.5 µM FCCP | 00:51:05 | 114.67 |
| 2 | 25 | D05 | 2.5 µM FCCP | 00:51:19 | 113.51 |
| 2 | 26 | D05 | 2.5 µM FCCP | 00:51:33 | 112.38 |
| 2 | 27 | D05 | 2.5 µM FCCP | 00:51:48 | 111.34 |
| 3 | 28 | D05 | 2.5 µM FCCP | 00:57:21 | 137.66 |
| 3 | 29 | D05 | 2.5 µM FCCP | 00:57:35 | 131.65 |
| 3 | 30 | D05 | 2.5 µM FCCP | 00:57:49 | 128.70 |
| 3 | 31 | D05 | 2.5 µM FCCP | 00:58:03 | 126.45 |
| 3 | 32 | D05 | 2.5 µM FCCP | 00:58:17 | 124.56 |
| 3 | 33 | D05 | 2.5 µM FCCP | 00:58:31 | 122.86 |
| 3 | 34 | D05 | 2.5 µM FCCP | 00:58:46 | 121.29 |
| 3 | 35 | D05 | 2.5 µM FCCP | 00:59:00 | 119.77 |
| 3 | 36 | D05 | 2.5 µM FCCP | 00:59:14 | 118.32 |
| 3 | 37 | D05 | 2.5 µM FCCP | 00:59:28 | 117.17 |
| 3 | 38 | D05 | 2.5 µM FCCP | 00:59:42 | 115.88 |
| 3 | 39 | D05 | 2.5 µM FCCP | 00:59:56 | 114.73 |
| 3 | 40 | D05 | 2.5 µM FCCP | 01:00:11 | 113.66 |
| 3 | 41 | D05 | 2.5 µM FCCP | 01:00:25 | 112.58 |
| 4 | 42 | D05 | 2.5 µM FCCP | 01:06:04 | 136.08 |
| 4 | 43 | D05 | 2.5 µM FCCP | 01:06:18 | 128.96 |
| 4 | 44 | D05 | 2.5 µM FCCP | 01:06:33 | 125.72 |
| 4 | 45 | D05 | 2.5 µM FCCP | 01:06:47 | 123.07 |
| 4 | 46 | D05 | 2.5 µM FCCP | 01:07:01 | 120.92 |
| 4 | 47 | D05 | 2.5 µM FCCP | 01:07:15 | 118.78 |
| 4 | 48 | D05 | 2.5 µM FCCP | 01:07:29 | 116.71 |
| 4 | 49 | D05 | 2.5 µM FCCP | 01:07:43 | 114.69 |
| 4 | 50 | D05 | 2.5 µM FCCP | 01:07:58 | 112.65 |
| 4 | 51 | D05 | 2.5 µM FCCP | 01:08:12 | 110.67 |
| 4 | 52 | D05 | 2.5 µM FCCP | 01:08:26 | 108.50 |
| 4 | 53 | D05 | 2.5 µM FCCP | 01:08:40 | 106.35 |
| 4 | 54 | D05 | 2.5 µM FCCP | 01:08:54 | 103.98 |
| 4 | 55 | D05 | 2.5 µM FCCP | 01:09:09 | 101.53 |
| 5 | 56 | D05 | 2.5 µM FCCP | 01:14:42 | 133.04 |
| 5 | 57 | D05 | 2.5 µM FCCP | 01:14:56 | 123.90 |
| 5 | 58 | D05 | 2.5 µM FCCP | 01:15:10 | 119.71 |
| 5 | 59 | D05 | 2.5 µM FCCP | 01:15:25 | 116.46 |
| 5 | 60 | D05 | 2.5 µM FCCP | 01:15:39 | 113.52 |
| 5 | 61 | D05 | 2.5 µM FCCP | 01:15:53 | 110.71 |
| 5 | 62 | D05 | 2.5 µM FCCP | 01:16:07 | 108.02 |
| 5 | 63 | D05 | 2.5 µM FCCP | 01:16:21 | 105.31 |
| 5 | 64 | D05 | 2.5 µM FCCP | 01:16:35 | 102.55 |
| 5 | 65 | D05 | 2.5 µM FCCP | 01:16:50 | 99.64 |
| 5 | 66 | D05 | 2.5 µM FCCP | 01:17:04 | 96.68 |
| 5 | 67 | D05 | 2.5 µM FCCP | 01:17:18 | 93.66 |
| 5 | 68 | D05 | 2.5 µM FCCP | 01:17:32 | 90.53 |
| 5 | 69 | D05 | 2.5 µM FCCP | 01:17:46 | 87.36 |
| 6 | 70 | D05 | 2.5 µM FCCP | 01:23:20 | 130.59 |
| 6 | 71 | D05 | 2.5 µM FCCP | 01:23:34 | 120.39 |
| 6 | 72 | D05 | 2.5 µM FCCP | 01:23:48 | 115.87 |
| 6 | 73 | D05 | 2.5 µM FCCP | 01:24:02 | 112.23 |
| 6 | 74 | D05 | 2.5 µM FCCP | 01:24:16 | 109.12 |
| 6 | 75 | D05 | 2.5 µM FCCP | 01:24:30 | 106.06 |
| 6 | 76 | D05 | 2.5 µM FCCP | 01:24:44 | 103.10 |
| 6 | 77 | D05 | 2.5 µM FCCP | 01:24:59 | 100.08 |
| 6 | 78 | D05 | 2.5 µM FCCP | 01:25:13 | 97.03 |
| 6 | 79 | D05 | 2.5 µM FCCP | 01:25:27 | 93.88 |
| 6 | 80 | D05 | 2.5 µM FCCP | 01:25:41 | 90.67 |
| 6 | 81 | D05 | 2.5 µM FCCP | 01:25:55 | 87.53 |
| 6 | 82 | D05 | 2.5 µM FCCP | 01:26:10 | 84.22 |
| 6 | 83 | D05 | 2.5 µM FCCP | 01:26:24 | 80.88 |
| 7 | 84 | D05 | 2.5 µM FCCP | 01:31:58 | 128.74 |
| 7 | 85 | D05 | 2.5 µM FCCP | 01:32:12 | 118.28 |
| 7 | 86 | D05 | 2.5 µM FCCP | 01:32:26 | 113.34 |
| 7 | 87 | D05 | 2.5 µM FCCP | 01:32:40 | 109.59 |
| 7 | 88 | D05 | 2.5 µM FCCP | 01:32:54 | 106.18 |
| 7 | 89 | D05 | 2.5 µM FCCP | 01:33:08 | 103.02 |
| 7 | 90 | D05 | 2.5 µM FCCP | 01:33:23 | 99.80 |
| 7 | 91 | D05 | 2.5 µM FCCP | 01:33:37 | 96.62 |
| 7 | 92 | D05 | 2.5 µM FCCP | 01:33:51 | 93.38 |
| 7 | 93 | D05 | 2.5 µM FCCP | 01:34:05 | 90.12 |
| 7 | 94 | D05 | 2.5 µM FCCP | 01:34:19 | 86.80 |
| 7 | 95 | D05 | 2.5 µM FCCP | 01:34:33 | 83.51 |
| 7 | 96 | D05 | 2.5 µM FCCP | 01:34:48 | 80.14 |
| 7 | 97 | D05 | 2.5 µM FCCP | 01:35:02 | 76.70 |
| 8 | 98 | D05 | 2.5 µM FCCP | 01:40:36 | 127.78 |
| 8 | 99 | D05 | 2.5 µM FCCP | 01:40:50 | 116.90 |
| 8 | 100 | D05 | 2.5 µM FCCP | 01:41:04 | 111.85 |
| 8 | 101 | D05 | 2.5 µM FCCP | 01:41:18 | 107.85 |
| 8 | 102 | D05 | 2.5 µM FCCP | 01:41:32 | 104.32 |
| 8 | 103 | D05 | 2.5 µM FCCP | 01:41:46 | 100.91 |
| 8 | 104 | D05 | 2.5 µM FCCP | 01:42:00 | 97.59 |
| 8 | 105 | D05 | 2.5 µM FCCP | 01:42:15 | 94.23 |
| 8 | 106 | D05 | 2.5 µM FCCP | 01:42:29 | 90.87 |
| 8 | 107 | D05 | 2.5 µM FCCP | 01:42:43 | 87.52 |
| 8 | 108 | D05 | 2.5 µM FCCP | 01:42:57 | 84.08 |
| 8 | 109 | D05 | 2.5 µM FCCP | 01:43:11 | 80.64 |
| 8 | 110 | D05 | 2.5 µM FCCP | 01:43:26 | 77.14 |
| 8 | 111 | D05 | 2.5 µM FCCP | 01:43:40 | 73.76 |
| 9 | 112 | D05 | 2.5 µM FCCP | 01:49:14 | 127.02 |
| 9 | 113 | D05 | 2.5 µM FCCP | 01:49:28 | 115.96 |
| 9 | 114 | D05 | 2.5 µM FCCP | 01:49:42 | 110.76 |
| 9 | 115 | D05 | 2.5 µM FCCP | 01:49:56 | 106.64 |
| 9 | 116 | D05 | 2.5 µM FCCP | 01:50:10 | 102.95 |
| 9 | 117 | D05 | 2.5 µM FCCP | 01:50:24 | 99.50 |
| 9 | 118 | D05 | 2.5 µM FCCP | 01:50:38 | 96.06 |
| 9 | 119 | D05 | 2.5 µM FCCP | 01:50:53 | 92.56 |
| 9 | 120 | D05 | 2.5 µM FCCP | 01:51:07 | 89.15 |
| 9 | 121 | D05 | 2.5 µM FCCP | 01:51:21 | 85.73 |
| 9 | 122 | D05 | 2.5 µM FCCP | 01:51:35 | 82.23 |
| 9 | 123 | D05 | 2.5 µM FCCP | 01:51:50 | 78.77 |
| 9 | 124 | D05 | 2.5 µM FCCP | 01:52:04 | 75.25 |
| 9 | 125 | D05 | 2.5 µM FCCP | 01:52:18 | 71.81 |
| 10 | 126 | D05 | 2.5 µM FCCP | 01:57:52 | 126.60 |
| 10 | 127 | D05 | 2.5 µM FCCP | 01:58:06 | 115.45 |
| 10 | 128 | D05 | 2.5 µM FCCP | 01:58:20 | 110.19 |
| 10 | 129 | D05 | 2.5 µM FCCP | 01:58:34 | 106.07 |
| 10 | 130 | D05 | 2.5 µM FCCP | 01:58:48 | 102.30 |
| 10 | 131 | D05 | 2.5 µM FCCP | 01:59:03 | 98.62 |
| 10 | 132 | D05 | 2.5 µM FCCP | 01:59:17 | 95.18 |
| 10 | 133 | D05 | 2.5 µM FCCP | 01:59:31 | 91.68 |
| 10 | 134 | D05 | 2.5 µM FCCP | 01:59:45 | 88.33 |
| 10 | 135 | D05 | 2.5 µM FCCP | 01:59:59 | 84.73 |
| 10 | 136 | D05 | 2.5 µM FCCP | 02:00:14 | 81.26 |
| 10 | 137 | D05 | 2.5 µM FCCP | 02:00:28 | 77.81 |
| 10 | 138 | D05 | 2.5 µM FCCP | 02:00:42 | 74.29 |
| 10 | 139 | D05 | 2.5 µM FCCP | 02:00:56 | 70.88 |
| 11 | 140 | D05 | 2.5 µM FCCP | 02:06:30 | 126.53 |
| 11 | 141 | D05 | 2.5 µM FCCP | 02:06:44 | 115.91 |
| 11 | 142 | D05 | 2.5 µM FCCP | 02:06:58 | 110.66 |
| 11 | 143 | D05 | 2.5 µM FCCP | 02:07:12 | 106.46 |
| 11 | 144 | D05 | 2.5 µM FCCP | 02:07:26 | 102.85 |
| 11 | 145 | D05 | 2.5 µM FCCP | 02:07:41 | 99.22 |
| 11 | 146 | D05 | 2.5 µM FCCP | 02:07:55 | 95.69 |
| 11 | 147 | D05 | 2.5 µM FCCP | 02:08:09 | 92.28 |
| 11 | 148 | D05 | 2.5 µM FCCP | 02:08:23 | 88.79 |
| 11 | 149 | D05 | 2.5 µM FCCP | 02:08:37 | 85.32 |
| 11 | 150 | D05 | 2.5 µM FCCP | 02:08:52 | 81.89 |
| 11 | 151 | D05 | 2.5 µM FCCP | 02:09:06 | 78.39 |
| 11 | 152 | D05 | 2.5 µM FCCP | 02:09:20 | 74.94 |
| 11 | 153 | D05 | 2.5 µM FCCP | 02:09:34 | 71.50 |
| 12 | 154 | D05 | 2.5 µM FCCP | 02:15:08 | 128.36 |
| 12 | 155 | D05 | 2.5 µM FCCP | 02:15:22 | 119.26 |
| 12 | 156 | D05 | 2.5 µM FCCP | 02:15:37 | 114.32 |
| 12 | 157 | D05 | 2.5 µM FCCP | 02:15:53 | 110.14 |
| 12 | 158 | D05 | 2.5 µM FCCP | 02:16:08 | 106.60 |
| 12 | 159 | D05 | 2.5 µM FCCP | 02:16:22 | 103.31 |
| 12 | 160 | D05 | 2.5 µM FCCP | 02:16:36 | 100.11 |
| 12 | 161 | D05 | 2.5 µM FCCP | 02:16:50 | 96.85 |
| 12 | 162 | D05 | 2.5 µM FCCP | 02:17:04 | 93.49 |
| 12 | 163 | D05 | 2.5 µM FCCP | 02:17:18 | 90.16 |
| 12 | 164 | D05 | 2.5 µM FCCP | 02:17:33 | 86.77 |
| 12 | 165 | D05 | 2.5 µM FCCP | 02:17:47 | 83.36 |
| 12 | 166 | D05 | 2.5 µM FCCP | 02:18:01 | 79.95 |
| 13 | 167 | D05 | 2.5 µM FCCP | 02:23:39 | 126.15 |
| 13 | 168 | D05 | 2.5 µM FCCP | 02:23:54 | 119.21 |
| 13 | 169 | D05 | 2.5 µM FCCP | 02:24:08 | 115.01 |
| 13 | 170 | D05 | 2.5 µM FCCP | 02:24:22 | 111.47 |
| 13 | 171 | D05 | 2.5 µM FCCP | 02:24:36 | 108.12 |
| 13 | 172 | D05 | 2.5 µM FCCP | 02:24:51 | 104.61 |
| 13 | 173 | D05 | 2.5 µM FCCP | 02:25:07 | 101.17 |
| 13 | 174 | D05 | 2.5 µM FCCP | 02:25:22 | 97.82 |
| 13 | 175 | D05 | 2.5 µM FCCP | 02:25:36 | 94.71 |
| 13 | 176 | D05 | 2.5 µM FCCP | 02:25:50 | 91.40 |
| 13 | 177 | D05 | 2.5 µM FCCP | 02:26:04 | 88.14 |
| 13 | 178 | D05 | 2.5 µM FCCP | 02:26:18 | 84.76 |
| 13 | 179 | D05 | 2.5 µM FCCP | 02:26:33 | 81.42 |
| 14 | 180 | D05 | 2.5 µM FCCP | 02:32:07 | 129.04 |
| 14 | 181 | D05 | 2.5 µM FCCP | 02:32:21 | 120.48 |
| 14 | 182 | D05 | 2.5 µM FCCP | 02:32:35 | 116.11 |
| 14 | 183 | D05 | 2.5 µM FCCP | 02:32:50 | 112.47 |
| 14 | 184 | D05 | 2.5 µM FCCP | 02:33:04 | 109.11 |
| 14 | 185 | D05 | 2.5 µM FCCP | 02:33:18 | 105.82 |
| 14 | 186 | D05 | 2.5 µM FCCP | 02:33:32 | 102.61 |
| 14 | 187 | D05 | 2.5 µM FCCP | 02:33:46 | 99.37 |
| 14 | 188 | D05 | 2.5 µM FCCP | 02:34:01 | 96.20 |
| 14 | 189 | D05 | 2.5 µM FCCP | 02:34:15 | 93.00 |
| 14 | 190 | D05 | 2.5 µM FCCP | 02:34:29 | 89.69 |
| 14 | 191 | D05 | 2.5 µM FCCP | 02:34:43 | 86.42 |
| 14 | 192 | D05 | 2.5 µM FCCP | 02:34:57 | 83.02 |
| 14 | 193 | D05 | 2.5 µM FCCP | 02:35:12 | 79.87 |
| 15 | 194 | D05 | 2.5 µM FCCP | 02:40:46 | 128.99 |
| 15 | 195 | D05 | 2.5 µM FCCP | 02:41:00 | 120.55 |
| 15 | 196 | D05 | 2.5 µM FCCP | 02:41:14 | 115.97 |
| 15 | 197 | D05 | 2.5 µM FCCP | 02:41:28 | 112.32 |
| 15 | 198 | D05 | 2.5 µM FCCP | 02:41:43 | 108.82 |
| 15 | 199 | D05 | 2.5 µM FCCP | 02:41:57 | 105.64 |
| 15 | 200 | D05 | 2.5 µM FCCP | 02:42:11 | 102.39 |
| 15 | 201 | D05 | 2.5 µM FCCP | 02:42:25 | 99.21 |
| 15 | 202 | D05 | 2.5 µM FCCP | 02:42:39 | 95.95 |
| 15 | 203 | D05 | 2.5 µM FCCP | 02:42:53 | 92.76 |
| 15 | 204 | D05 | 2.5 µM FCCP | 02:43:08 | 89.41 |
| 15 | 205 | D05 | 2.5 µM FCCP | 02:43:22 | 86.26 |
| 15 | 206 | D05 | 2.5 µM FCCP | 02:43:36 | 82.92 |
| 15 | 207 | D05 | 2.5 µM FCCP | 02:43:50 | 79.71 |
| 16 | 208 | D05 | 2.5 µM FCCP | 02:49:25 | 129.02 |
| 16 | 209 | D05 | 2.5 µM FCCP | 02:49:40 | 120.53 |
| 16 | 210 | D05 | 2.5 µM FCCP | 02:49:54 | 116.11 |
| 16 | 211 | D05 | 2.5 µM FCCP | 02:50:08 | 112.35 |
| 16 | 212 | D05 | 2.5 µM FCCP | 02:50:22 | 108.92 |
| 16 | 213 | D05 | 2.5 µM FCCP | 02:50:36 | 105.58 |
| 16 | 214 | D05 | 2.5 µM FCCP | 02:50:50 | 102.41 |
| 16 | 215 | D05 | 2.5 µM FCCP | 02:51:05 | 99.21 |
| 16 | 216 | D05 | 2.5 µM FCCP | 02:51:19 | 95.97 |
| 16 | 217 | D05 | 2.5 µM FCCP | 02:51:33 | 92.75 |
| 16 | 218 | D05 | 2.5 µM FCCP | 02:51:47 | 89.52 |
| 16 | 219 | D05 | 2.5 µM FCCP | 02:52:01 | 86.33 |
| 16 | 220 | D05 | 2.5 µM FCCP | 02:52:16 | 83.07 |
| 16 | 221 | D05 | 2.5 µM FCCP | 02:52:30 | 79.91 |
| 1 | 0 | D06 | 2.5 µM FCCP | 00:40:06 | 128.41 |
| 1 | 1 | D06 | 2.5 µM FCCP | 00:40:20 | 120.73 |
| 1 | 2 | D06 | 2.5 µM FCCP | 00:40:34 | 117.82 |
| 1 | 3 | D06 | 2.5 µM FCCP | 00:40:48 | 115.60 |
| 1 | 4 | D06 | 2.5 µM FCCP | 00:41:02 | 113.73 |
| 1 | 5 | D06 | 2.5 µM FCCP | 00:41:17 | 112.05 |
| 1 | 6 | D06 | 2.5 µM FCCP | 00:41:31 | 110.45 |
| 1 | 7 | D06 | 2.5 µM FCCP | 00:41:45 | 109.08 |
| 1 | 8 | D06 | 2.5 µM FCCP | 00:41:59 | 107.64 |
| 1 | 9 | D06 | 2.5 µM FCCP | 00:42:13 | 106.46 |
| 1 | 10 | D06 | 2.5 µM FCCP | 00:42:28 | 105.14 |
| 1 | 11 | D06 | 2.5 µM FCCP | 00:42:42 | 103.96 |
| 1 | 12 | D06 | 2.5 µM FCCP | 00:42:56 | 102.80 |
| 1 | 13 | D06 | 2.5 µM FCCP | 00:43:10 | 101.77 |
| 2 | 14 | D06 | 2.5 µM FCCP | 00:48:43 | 132.49 |
| 2 | 15 | D06 | 2.5 µM FCCP | 00:48:57 | 126.36 |
| 2 | 16 | D06 | 2.5 µM FCCP | 00:49:12 | 123.39 |
| 2 | 17 | D06 | 2.5 µM FCCP | 00:49:26 | 121.00 |
| 2 | 18 | D06 | 2.5 µM FCCP | 00:49:40 | 118.98 |
| 2 | 19 | D06 | 2.5 µM FCCP | 00:49:54 | 117.10 |
| 2 | 20 | D06 | 2.5 µM FCCP | 00:50:08 | 115.39 |
| 2 | 21 | D06 | 2.5 µM FCCP | 00:50:22 | 113.75 |
| 2 | 22 | D06 | 2.5 µM FCCP | 00:50:37 | 112.36 |
| 2 | 23 | D06 | 2.5 µM FCCP | 00:50:51 | 110.94 |
| 2 | 24 | D06 | 2.5 µM FCCP | 00:51:05 | 109.52 |
| 2 | 25 | D06 | 2.5 µM FCCP | 00:51:19 | 108.34 |
| 2 | 26 | D06 | 2.5 µM FCCP | 00:51:33 | 107.03 |
| 2 | 27 | D06 | 2.5 µM FCCP | 00:51:48 | 105.86 |
| 3 | 28 | D06 | 2.5 µM FCCP | 00:57:21 | 133.35 |
| 3 | 29 | D06 | 2.5 µM FCCP | 00:57:35 | 128.13 |
| 3 | 30 | D06 | 2.5 µM FCCP | 00:57:49 | 125.29 |
| 3 | 31 | D06 | 2.5 µM FCCP | 00:58:03 | 123.07 |
| 3 | 32 | D06 | 2.5 µM FCCP | 00:58:17 | 121.08 |
| 3 | 33 | D06 | 2.5 µM FCCP | 00:58:31 | 119.29 |
| 3 | 34 | D06 | 2.5 µM FCCP | 00:58:46 | 117.62 |
| 3 | 35 | D06 | 2.5 µM FCCP | 00:59:00 | 116.00 |
| 3 | 36 | D06 | 2.5 µM FCCP | 00:59:14 | 114.48 |
| 3 | 37 | D06 | 2.5 µM FCCP | 00:59:28 | 113.15 |
| 3 | 38 | D06 | 2.5 µM FCCP | 00:59:42 | 111.76 |
| 3 | 39 | D06 | 2.5 µM FCCP | 00:59:56 | 110.55 |
| 3 | 40 | D06 | 2.5 µM FCCP | 01:00:11 | 109.31 |
| 3 | 41 | D06 | 2.5 µM FCCP | 01:00:25 | 108.06 |
| 4 | 42 | D06 | 2.5 µM FCCP | 01:06:04 | 131.99 |
| 4 | 43 | D06 | 2.5 µM FCCP | 01:06:18 | 124.94 |
| 4 | 44 | D06 | 2.5 µM FCCP | 01:06:33 | 121.48 |
| 4 | 45 | D06 | 2.5 µM FCCP | 01:06:47 | 118.78 |
| 4 | 46 | D06 | 2.5 µM FCCP | 01:07:01 | 116.47 |
| 4 | 47 | D06 | 2.5 µM FCCP | 01:07:15 | 114.15 |
| 4 | 48 | D06 | 2.5 µM FCCP | 01:07:29 | 111.92 |
| 4 | 49 | D06 | 2.5 µM FCCP | 01:07:43 | 109.70 |
| 4 | 50 | D06 | 2.5 µM FCCP | 01:07:58 | 107.56 |
| 4 | 51 | D06 | 2.5 µM FCCP | 01:08:12 | 105.35 |
| 4 | 52 | D06 | 2.5 µM FCCP | 01:08:26 | 103.01 |
| 4 | 53 | D06 | 2.5 µM FCCP | 01:08:40 | 100.62 |
| 4 | 54 | D06 | 2.5 µM FCCP | 01:08:54 | 98.07 |
| 4 | 55 | D06 | 2.5 µM FCCP | 01:09:09 | 95.43 |
| 5 | 56 | D06 | 2.5 µM FCCP | 01:14:42 | 128.39 |
| 5 | 57 | D06 | 2.5 µM FCCP | 01:14:56 | 119.43 |
| 5 | 58 | D06 | 2.5 µM FCCP | 01:15:10 | 115.08 |
| 5 | 59 | D06 | 2.5 µM FCCP | 01:15:25 | 111.77 |
| 5 | 60 | D06 | 2.5 µM FCCP | 01:15:39 | 108.74 |
| 5 | 61 | D06 | 2.5 µM FCCP | 01:15:53 | 105.76 |
| 5 | 62 | D06 | 2.5 µM FCCP | 01:16:07 | 102.96 |
| 5 | 63 | D06 | 2.5 µM FCCP | 01:16:21 | 99.98 |
| 5 | 64 | D06 | 2.5 µM FCCP | 01:16:35 | 97.12 |
| 5 | 65 | D06 | 2.5 µM FCCP | 01:16:50 | 94.06 |
| 5 | 66 | D06 | 2.5 µM FCCP | 01:17:04 | 90.88 |
| 5 | 67 | D06 | 2.5 µM FCCP | 01:17:18 | 87.65 |
| 5 | 68 | D06 | 2.5 µM FCCP | 01:17:32 | 84.30 |
| 5 | 69 | D06 | 2.5 µM FCCP | 01:17:46 | 80.93 |
| 6 | 70 | D06 | 2.5 µM FCCP | 01:23:20 | 125.85 |
| 6 | 71 | D06 | 2.5 µM FCCP | 01:23:34 | 115.79 |
| 6 | 72 | D06 | 2.5 µM FCCP | 01:23:48 | 111.19 |
| 6 | 73 | D06 | 2.5 µM FCCP | 01:24:02 | 107.45 |
| 6 | 74 | D06 | 2.5 µM FCCP | 01:24:16 | 104.19 |
| 6 | 75 | D06 | 2.5 µM FCCP | 01:24:30 | 100.87 |
| 6 | 76 | D06 | 2.5 µM FCCP | 01:24:44 | 97.84 |
| 6 | 77 | D06 | 2.5 µM FCCP | 01:24:59 | 94.63 |
| 6 | 78 | D06 | 2.5 µM FCCP | 01:25:13 | 91.44 |
| 6 | 79 | D06 | 2.5 µM FCCP | 01:25:27 | 88.12 |
| 6 | 80 | D06 | 2.5 µM FCCP | 01:25:41 | 84.75 |
| 6 | 81 | D06 | 2.5 µM FCCP | 01:25:55 | 81.35 |
| 6 | 82 | D06 | 2.5 µM FCCP | 01:26:10 | 77.74 |
| 6 | 83 | D06 | 2.5 µM FCCP | 01:26:24 | 74.22 |
| 7 | 84 | D06 | 2.5 µM FCCP | 01:31:58 | 124.24 |
| 7 | 85 | D06 | 2.5 µM FCCP | 01:32:12 | 113.73 |
| 7 | 86 | D06 | 2.5 µM FCCP | 01:32:26 | 108.68 |
| 7 | 87 | D06 | 2.5 µM FCCP | 01:32:40 | 104.86 |
| 7 | 88 | D06 | 2.5 µM FCCP | 01:32:54 | 101.30 |
| 7 | 89 | D06 | 2.5 µM FCCP | 01:33:08 | 97.97 |
| 7 | 90 | D06 | 2.5 µM FCCP | 01:33:23 | 94.62 |
| 7 | 91 | D06 | 2.5 µM FCCP | 01:33:37 | 91.16 |
| 7 | 92 | D06 | 2.5 µM FCCP | 01:33:51 | 87.80 |
| 7 | 93 | D06 | 2.5 µM FCCP | 01:34:05 | 84.40 |
| 7 | 94 | D06 | 2.5 µM FCCP | 01:34:19 | 80.81 |
| 7 | 95 | D06 | 2.5 µM FCCP | 01:34:33 | 77.36 |
| 7 | 96 | D06 | 2.5 µM FCCP | 01:34:48 | 73.69 |
| 7 | 97 | D06 | 2.5 µM FCCP | 01:35:02 | 70.00 |
| 8 | 98 | D06 | 2.5 µM FCCP | 01:40:36 | 123.29 |
| 8 | 99 | D06 | 2.5 µM FCCP | 01:40:50 | 112.40 |
| 8 | 100 | D06 | 2.5 µM FCCP | 01:41:04 | 107.24 |
| 8 | 101 | D06 | 2.5 µM FCCP | 01:41:18 | 103.14 |
| 8 | 102 | D06 | 2.5 µM FCCP | 01:41:32 | 99.41 |
| 8 | 103 | D06 | 2.5 µM FCCP | 01:41:46 | 95.86 |
| 8 | 104 | D06 | 2.5 µM FCCP | 01:42:00 | 92.37 |
| 8 | 105 | D06 | 2.5 µM FCCP | 01:42:15 | 88.88 |
| 8 | 106 | D06 | 2.5 µM FCCP | 01:42:29 | 85.33 |
| 8 | 107 | D06 | 2.5 µM FCCP | 01:42:43 | 81.74 |
| 8 | 108 | D06 | 2.5 µM FCCP | 01:42:57 | 78.08 |
| 8 | 109 | D06 | 2.5 µM FCCP | 01:43:11 | 74.44 |
| 8 | 110 | D06 | 2.5 µM FCCP | 01:43:26 | 70.67 |
| 8 | 111 | D06 | 2.5 µM FCCP | 01:43:40 | 67.02 |
| 9 | 112 | D06 | 2.5 µM FCCP | 01:49:14 | 122.63 |
| 9 | 113 | D06 | 2.5 µM FCCP | 01:49:28 | 111.51 |
| 9 | 114 | D06 | 2.5 µM FCCP | 01:49:42 | 106.20 |
| 9 | 115 | D06 | 2.5 µM FCCP | 01:49:56 | 101.99 |
| 9 | 116 | D06 | 2.5 µM FCCP | 01:50:10 | 98.15 |
| 9 | 117 | D06 | 2.5 µM FCCP | 01:50:24 | 94.46 |
| 9 | 118 | D06 | 2.5 µM FCCP | 01:50:38 | 90.84 |
| 9 | 119 | D06 | 2.5 µM FCCP | 01:50:53 | 87.16 |
| 9 | 120 | D06 | 2.5 µM FCCP | 01:51:07 | 83.58 |
| 9 | 121 | D06 | 2.5 µM FCCP | 01:51:21 | 79.94 |
| 9 | 122 | D06 | 2.5 µM FCCP | 01:51:35 | 76.22 |
| 9 | 123 | D06 | 2.5 µM FCCP | 01:51:50 | 72.52 |
| 9 | 124 | D06 | 2.5 µM FCCP | 01:52:04 | 68.76 |
| 9 | 125 | D06 | 2.5 µM FCCP | 01:52:18 | 65.05 |
| 10 | 126 | D06 | 2.5 µM FCCP | 01:57:52 | 122.29 |
| 10 | 127 | D06 | 2.5 µM FCCP | 01:58:06 | 111.08 |
| 10 | 128 | D06 | 2.5 µM FCCP | 01:58:20 | 105.63 |
| 10 | 129 | D06 | 2.5 µM FCCP | 01:58:34 | 101.48 |
| 10 | 130 | D06 | 2.5 µM FCCP | 01:58:48 | 97.56 |
| 10 | 131 | D06 | 2.5 µM FCCP | 01:59:03 | 93.72 |
| 10 | 132 | D06 | 2.5 µM FCCP | 01:59:17 | 90.02 |
| 10 | 133 | D06 | 2.5 µM FCCP | 01:59:31 | 86.30 |
| 10 | 134 | D06 | 2.5 µM FCCP | 01:59:45 | 82.78 |
| 10 | 135 | D06 | 2.5 µM FCCP | 01:59:59 | 79.02 |
| 10 | 136 | D06 | 2.5 µM FCCP | 02:00:14 | 75.32 |
| 10 | 137 | D06 | 2.5 µM FCCP | 02:00:28 | 71.64 |
| 10 | 138 | D06 | 2.5 µM FCCP | 02:00:42 | 67.85 |
| 10 | 139 | D06 | 2.5 µM FCCP | 02:00:56 | 64.19 |
| 11 | 140 | D06 | 2.5 µM FCCP | 02:06:30 | 122.09 |
| 11 | 141 | D06 | 2.5 µM FCCP | 02:06:44 | 111.12 |
| 11 | 142 | D06 | 2.5 µM FCCP | 02:06:58 | 105.71 |
| 11 | 143 | D06 | 2.5 µM FCCP | 02:07:12 | 101.46 |
| 11 | 144 | D06 | 2.5 µM FCCP | 02:07:26 | 97.66 |
| 11 | 145 | D06 | 2.5 µM FCCP | 02:07:41 | 93.86 |
| 11 | 146 | D06 | 2.5 µM FCCP | 02:07:55 | 90.15 |
| 11 | 147 | D06 | 2.5 µM FCCP | 02:08:09 | 86.49 |
| 11 | 148 | D06 | 2.5 µM FCCP | 02:08:23 | 82.84 |
| 11 | 149 | D06 | 2.5 µM FCCP | 02:08:37 | 79.19 |
| 11 | 150 | D06 | 2.5 µM FCCP | 02:08:52 | 75.49 |
| 11 | 151 | D06 | 2.5 µM FCCP | 02:09:06 | 71.77 |
| 11 | 152 | D06 | 2.5 µM FCCP | 02:09:20 | 68.10 |
| 11 | 153 | D06 | 2.5 µM FCCP | 02:09:34 | 64.38 |
| 12 | 154 | D06 | 2.5 µM FCCP | 02:15:08 | 123.92 |
| 12 | 155 | D06 | 2.5 µM FCCP | 02:15:22 | 114.69 |
| 12 | 156 | D06 | 2.5 µM FCCP | 02:15:37 | 109.55 |
| 12 | 157 | D06 | 2.5 µM FCCP | 02:15:53 | 105.27 |
| 12 | 158 | D06 | 2.5 µM FCCP | 02:16:08 | 101.54 |
| 12 | 159 | D06 | 2.5 µM FCCP | 02:16:22 | 98.16 |
| 12 | 160 | D06 | 2.5 µM FCCP | 02:16:36 | 94.65 |
| 12 | 161 | D06 | 2.5 µM FCCP | 02:16:50 | 91.23 |
| 12 | 162 | D06 | 2.5 µM FCCP | 02:17:04 | 87.71 |
| 12 | 163 | D06 | 2.5 µM FCCP | 02:17:18 | 84.15 |
| 12 | 164 | D06 | 2.5 µM FCCP | 02:17:33 | 80.61 |
| 12 | 165 | D06 | 2.5 µM FCCP | 02:17:47 | 77.01 |
| 12 | 166 | D06 | 2.5 µM FCCP | 02:18:01 | 73.32 |
| 13 | 167 | D06 | 2.5 µM FCCP | 02:23:39 | 123.09 |
| 13 | 168 | D06 | 2.5 µM FCCP | 02:23:54 | 116.54 |
| 13 | 169 | D06 | 2.5 µM FCCP | 02:24:08 | 112.35 |
| 13 | 170 | D06 | 2.5 µM FCCP | 02:24:22 | 108.71 |
| 13 | 171 | D06 | 2.5 µM FCCP | 02:24:36 | 105.25 |
| 13 | 172 | D06 | 2.5 µM FCCP | 02:24:51 | 101.67 |
| 13 | 173 | D06 | 2.5 µM FCCP | 02:25:07 | 98.00 |
| 13 | 174 | D06 | 2.5 µM FCCP | 02:25:22 | 94.63 |
| 13 | 175 | D06 | 2.5 µM FCCP | 02:25:36 | 91.29 |
| 13 | 176 | D06 | 2.5 µM FCCP | 02:25:50 | 87.85 |
| 13 | 177 | D06 | 2.5 µM FCCP | 02:26:04 | 84.51 |
| 13 | 178 | D06 | 2.5 µM FCCP | 02:26:18 | 80.93 |
| 13 | 179 | D06 | 2.5 µM FCCP | 02:26:33 | 77.43 |
| 14 | 180 | D06 | 2.5 µM FCCP | 02:32:07 | 126.91 |
| 14 | 181 | D06 | 2.5 µM FCCP | 02:32:21 | 119.28 |
| 14 | 182 | D06 | 2.5 µM FCCP | 02:32:35 | 114.93 |
| 14 | 183 | D06 | 2.5 µM FCCP | 02:32:50 | 111.25 |
| 14 | 184 | D06 | 2.5 µM FCCP | 02:33:04 | 107.88 |
| 14 | 185 | D06 | 2.5 µM FCCP | 02:33:18 | 104.51 |
| 14 | 186 | D06 | 2.5 µM FCCP | 02:33:32 | 101.24 |
| 14 | 187 | D06 | 2.5 µM FCCP | 02:33:46 | 97.89 |
| 14 | 188 | D06 | 2.5 µM FCCP | 02:34:01 | 94.62 |
| 14 | 189 | D06 | 2.5 µM FCCP | 02:34:15 | 91.28 |
| 14 | 190 | D06 | 2.5 µM FCCP | 02:34:29 | 87.89 |
| 14 | 191 | D06 | 2.5 µM FCCP | 02:34:43 | 84.53 |
| 14 | 192 | D06 | 2.5 µM FCCP | 02:34:57 | 80.97 |
| 14 | 193 | D06 | 2.5 µM FCCP | 02:35:12 | 77.59 |
| 15 | 194 | D06 | 2.5 µM FCCP | 02:40:46 | 126.88 |
| 15 | 195 | D06 | 2.5 µM FCCP | 02:41:00 | 118.98 |
| 15 | 196 | D06 | 2.5 µM FCCP | 02:41:14 | 114.51 |
| 15 | 197 | D06 | 2.5 µM FCCP | 02:41:28 | 110.84 |
| 15 | 198 | D06 | 2.5 µM FCCP | 02:41:43 | 107.38 |
| 15 | 199 | D06 | 2.5 µM FCCP | 02:41:57 | 103.99 |
| 15 | 200 | D06 | 2.5 µM FCCP | 02:42:11 | 100.69 |
| 15 | 201 | D06 | 2.5 µM FCCP | 02:42:25 | 97.33 |
| 15 | 202 | D06 | 2.5 µM FCCP | 02:42:39 | 94.05 |
| 15 | 203 | D06 | 2.5 µM FCCP | 02:42:53 | 90.70 |
| 15 | 204 | D06 | 2.5 µM FCCP | 02:43:08 | 87.22 |
| 15 | 205 | D06 | 2.5 µM FCCP | 02:43:22 | 83.91 |
| 15 | 206 | D06 | 2.5 µM FCCP | 02:43:36 | 80.32 |
| 15 | 207 | D06 | 2.5 µM FCCP | 02:43:50 | 77.01 |
| 16 | 208 | D06 | 2.5 µM FCCP | 02:49:25 | 126.68 |
| 16 | 209 | D06 | 2.5 µM FCCP | 02:49:40 | 118.72 |
| 16 | 210 | D06 | 2.5 µM FCCP | 02:49:54 | 114.18 |
| 16 | 211 | D06 | 2.5 µM FCCP | 02:50:08 | 110.40 |
| 16 | 212 | D06 | 2.5 µM FCCP | 02:50:22 | 106.93 |
| 16 | 213 | D06 | 2.5 µM FCCP | 02:50:36 | 103.49 |
| 16 | 214 | D06 | 2.5 µM FCCP | 02:50:50 | 100.14 |
| 16 | 215 | D06 | 2.5 µM FCCP | 02:51:05 | 96.77 |
| 16 | 216 | D06 | 2.5 µM FCCP | 02:51:19 | 93.39 |
| 16 | 217 | D06 | 2.5 µM FCCP | 02:51:33 | 90.02 |
| 16 | 218 | D06 | 2.5 µM FCCP | 02:51:47 | 86.63 |
| 16 | 219 | D06 | 2.5 µM FCCP | 02:52:01 | 83.32 |
| 16 | 220 | D06 | 2.5 µM FCCP | 02:52:16 | 79.84 |
| 16 | 221 | D06 | 2.5 µM FCCP | 02:52:30 | 76.52 |
| 1 | 0 | G01 | 25 µM BAM15 | 00:40:06 | 138.21 |
| 1 | 1 | G01 | 25 µM BAM15 | 00:40:20 | 135.71 |
| 1 | 2 | G01 | 25 µM BAM15 | 00:40:34 | 134.11 |
| 1 | 3 | G01 | 25 µM BAM15 | 00:40:48 | 132.82 |
| 1 | 4 | G01 | 25 µM BAM15 | 00:41:02 | 131.59 |
| 1 | 5 | G01 | 25 µM BAM15 | 00:41:17 | 130.48 |
| 1 | 6 | G01 | 25 µM BAM15 | 00:41:31 | 129.47 |
| 1 | 7 | G01 | 25 µM BAM15 | 00:41:45 | 128.36 |
| 1 | 8 | G01 | 25 µM BAM15 | 00:41:59 | 127.48 |
| 1 | 9 | G01 | 25 µM BAM15 | 00:42:13 | 126.61 |
| 1 | 10 | G01 | 25 µM BAM15 | 00:42:28 | 125.62 |
| 1 | 11 | G01 | 25 µM BAM15 | 00:42:42 | 124.81 |
| 1 | 12 | G01 | 25 µM BAM15 | 00:42:56 | 123.94 |
| 1 | 13 | G01 | 25 µM BAM15 | 00:43:10 | 123.23 |
| 2 | 14 | G01 | 25 µM BAM15 | 00:48:43 | 138.50 |
| 2 | 15 | G01 | 25 µM BAM15 | 00:48:57 | 135.77 |
| 2 | 16 | G01 | 25 µM BAM15 | 00:49:12 | 134.15 |
| 2 | 17 | G01 | 25 µM BAM15 | 00:49:26 | 132.70 |
| 2 | 18 | G01 | 25 µM BAM15 | 00:49:40 | 131.45 |
| 2 | 19 | G01 | 25 µM BAM15 | 00:49:54 | 130.25 |
| 2 | 20 | G01 | 25 µM BAM15 | 00:50:08 | 129.29 |
| 2 | 21 | G01 | 25 µM BAM15 | 00:50:22 | 128.21 |
| 2 | 22 | G01 | 25 µM BAM15 | 00:50:37 | 127.22 |
| 2 | 23 | G01 | 25 µM BAM15 | 00:50:51 | 126.34 |
| 2 | 24 | G01 | 25 µM BAM15 | 00:51:05 | 125.37 |
| 2 | 25 | G01 | 25 µM BAM15 | 00:51:19 | 124.62 |
| 2 | 26 | G01 | 25 µM BAM15 | 00:51:33 | 123.80 |
| 2 | 27 | G01 | 25 µM BAM15 | 00:51:48 | 123.00 |
| 3 | 28 | G01 | 25 µM BAM15 | 00:57:21 | 138.66 |
| 3 | 29 | G01 | 25 µM BAM15 | 00:57:35 | 136.01 |
| 3 | 30 | G01 | 25 µM BAM15 | 00:57:49 | 134.37 |
| 3 | 31 | G01 | 25 µM BAM15 | 00:58:03 | 132.90 |
| 3 | 32 | G01 | 25 µM BAM15 | 00:58:17 | 131.76 |
| 3 | 33 | G01 | 25 µM BAM15 | 00:58:31 | 130.52 |
| 3 | 34 | G01 | 25 µM BAM15 | 00:58:46 | 129.47 |
| 3 | 35 | G01 | 25 µM BAM15 | 00:59:00 | 128.50 |
| 3 | 36 | G01 | 25 µM BAM15 | 00:59:14 | 127.45 |
| 3 | 37 | G01 | 25 µM BAM15 | 00:59:28 | 126.61 |
| 3 | 38 | G01 | 25 µM BAM15 | 00:59:42 | 125.61 |
| 3 | 39 | G01 | 25 µM BAM15 | 00:59:56 | 124.77 |
| 3 | 40 | G01 | 25 µM BAM15 | 01:00:11 | 123.96 |
| 3 | 41 | G01 | 25 µM BAM15 | 01:00:25 | 123.26 |
| 4 | 42 | G01 | 25 µM BAM15 | 01:06:04 | 129.07 |
| 4 | 43 | G01 | 25 µM BAM15 | 01:06:18 | 120.18 |
| 4 | 44 | G01 | 25 µM BAM15 | 01:06:33 | 114.43 |
| 4 | 45 | G01 | 25 µM BAM15 | 01:06:47 | 109.90 |
| 4 | 46 | G01 | 25 µM BAM15 | 01:07:01 | 105.99 |
| 4 | 47 | G01 | 25 µM BAM15 | 01:07:15 | 102.39 |
| 4 | 48 | G01 | 25 µM BAM15 | 01:07:29 | 99.07 |
| 4 | 49 | G01 | 25 µM BAM15 | 01:07:43 | 96.01 |
| 4 | 50 | G01 | 25 µM BAM15 | 01:07:58 | 93.15 |
| 4 | 51 | G01 | 25 µM BAM15 | 01:08:12 | 90.51 |
| 4 | 52 | G01 | 25 µM BAM15 | 01:08:26 | 87.94 |
| 4 | 53 | G01 | 25 µM BAM15 | 01:08:40 | 85.66 |
| 4 | 54 | G01 | 25 µM BAM15 | 01:08:54 | 83.32 |
| 4 | 55 | G01 | 25 µM BAM15 | 01:09:09 | 81.24 |
| 5 | 56 | G01 | 25 µM BAM15 | 01:14:42 | 120.44 |
| 5 | 57 | G01 | 25 µM BAM15 | 01:14:56 | 110.04 |
| 5 | 58 | G01 | 25 µM BAM15 | 01:15:10 | 103.80 |
| 5 | 59 | G01 | 25 µM BAM15 | 01:15:25 | 98.90 |
| 5 | 60 | G01 | 25 µM BAM15 | 01:15:39 | 94.70 |
| 5 | 61 | G01 | 25 µM BAM15 | 01:15:53 | 90.71 |
| 5 | 62 | G01 | 25 µM BAM15 | 01:16:07 | 87.23 |
| 5 | 63 | G01 | 25 µM BAM15 | 01:16:21 | 83.87 |
| 5 | 64 | G01 | 25 µM BAM15 | 01:16:35 | 80.86 |
| 5 | 65 | G01 | 25 µM BAM15 | 01:16:50 | 78.05 |
| 5 | 66 | G01 | 25 µM BAM15 | 01:17:04 | 75.30 |
| 5 | 67 | G01 | 25 µM BAM15 | 01:17:18 | 72.71 |
| 5 | 68 | G01 | 25 µM BAM15 | 01:17:32 | 70.20 |
| 5 | 69 | G01 | 25 µM BAM15 | 01:17:46 | 67.89 |
| 6 | 70 | G01 | 25 µM BAM15 | 01:23:20 | 115.56 |
| 6 | 71 | G01 | 25 µM BAM15 | 01:23:34 | 104.32 |
| 6 | 72 | G01 | 25 µM BAM15 | 01:23:48 | 97.79 |
| 6 | 73 | G01 | 25 µM BAM15 | 01:24:02 | 92.55 |
| 6 | 74 | G01 | 25 µM BAM15 | 01:24:16 | 88.04 |
| 6 | 75 | G01 | 25 µM BAM15 | 01:24:30 | 83.88 |
| 6 | 76 | G01 | 25 µM BAM15 | 01:24:44 | 80.13 |
| 6 | 77 | G01 | 25 µM BAM15 | 01:24:59 | 76.59 |
| 6 | 78 | G01 | 25 µM BAM15 | 01:25:13 | 73.34 |
| 6 | 79 | G01 | 25 µM BAM15 | 01:25:27 | 70.25 |
| 6 | 80 | G01 | 25 µM BAM15 | 01:25:41 | 67.30 |
| 6 | 81 | G01 | 25 µM BAM15 | 01:25:55 | 64.57 |
| 6 | 82 | G01 | 25 µM BAM15 | 01:26:10 | 61.85 |
| 6 | 83 | G01 | 25 µM BAM15 | 01:26:24 | 59.36 |
| 7 | 84 | G01 | 25 µM BAM15 | 01:31:58 | 112.66 |
| 7 | 85 | G01 | 25 µM BAM15 | 01:32:12 | 101.30 |
| 7 | 86 | G01 | 25 µM BAM15 | 01:32:26 | 94.50 |
| 7 | 87 | G01 | 25 µM BAM15 | 01:32:40 | 89.19 |
| 7 | 88 | G01 | 25 µM BAM15 | 01:32:54 | 84.48 |
| 7 | 89 | G01 | 25 µM BAM15 | 01:33:08 | 80.26 |
| 7 | 90 | G01 | 25 µM BAM15 | 01:33:23 | 76.33 |
| 7 | 91 | G01 | 25 µM BAM15 | 01:33:37 | 72.63 |
| 7 | 92 | G01 | 25 µM BAM15 | 01:33:51 | 69.29 |
| 7 | 93 | G01 | 25 µM BAM15 | 01:34:05 | 66.10 |
| 7 | 94 | G01 | 25 µM BAM15 | 01:34:19 | 62.98 |
| 7 | 95 | G01 | 25 µM BAM15 | 01:34:33 | 60.16 |
| 7 | 96 | G01 | 25 µM BAM15 | 01:34:48 | 57.36 |
| 7 | 97 | G01 | 25 µM BAM15 | 01:35:02 | 54.77 |
| 8 | 98 | G01 | 25 µM BAM15 | 01:40:36 | 111.20 |
| 8 | 99 | G01 | 25 µM BAM15 | 01:40:50 | 100.05 |
| 8 | 100 | G01 | 25 µM BAM15 | 01:41:04 | 93.35 |
| 8 | 101 | G01 | 25 µM BAM15 | 01:41:18 | 87.91 |
| 8 | 102 | G01 | 25 µM BAM15 | 01:41:32 | 83.16 |
| 8 | 103 | G01 | 25 µM BAM15 | 01:41:46 | 78.78 |
| 8 | 104 | G01 | 25 µM BAM15 | 01:42:00 | 74.85 |
| 8 | 105 | G01 | 25 µM BAM15 | 01:42:15 | 71.08 |
| 8 | 106 | G01 | 25 µM BAM15 | 01:42:29 | 67.55 |
| 8 | 107 | G01 | 25 µM BAM15 | 01:42:43 | 64.30 |
| 8 | 108 | G01 | 25 µM BAM15 | 01:42:57 | 61.15 |
| 8 | 109 | G01 | 25 µM BAM15 | 01:43:11 | 58.18 |
| 8 | 110 | G01 | 25 µM BAM15 | 01:43:26 | 55.30 |
| 8 | 111 | G01 | 25 µM BAM15 | 01:43:40 | 52.67 |
| 9 | 112 | G01 | 25 µM BAM15 | 01:49:14 | 112.31 |
| 9 | 113 | G01 | 25 µM BAM15 | 01:49:28 | 102.43 |
| 9 | 114 | G01 | 25 µM BAM15 | 01:49:42 | 96.02 |
| 9 | 115 | G01 | 25 µM BAM15 | 01:49:56 | 90.74 |
| 9 | 116 | G01 | 25 µM BAM15 | 01:50:10 | 85.99 |
| 9 | 117 | G01 | 25 µM BAM15 | 01:50:24 | 81.65 |
| 9 | 118 | G01 | 25 µM BAM15 | 01:50:38 | 77.61 |
| 9 | 119 | G01 | 25 µM BAM15 | 01:50:53 | 73.73 |
| 9 | 120 | G01 | 25 µM BAM15 | 01:51:07 | 70.20 |
| 9 | 121 | G01 | 25 µM BAM15 | 01:51:21 | 66.88 |
| 9 | 122 | G01 | 25 µM BAM15 | 01:51:35 | 63.69 |
| 9 | 123 | G01 | 25 µM BAM15 | 01:51:50 | 60.65 |
| 9 | 124 | G01 | 25 µM BAM15 | 01:52:04 | 57.72 |
| 9 | 125 | G01 | 25 µM BAM15 | 01:52:18 | 55.01 |
| 10 | 126 | G01 | 25 µM BAM15 | 01:57:52 | 112.92 |
| 10 | 127 | G01 | 25 µM BAM15 | 01:58:06 | 103.19 |
| 10 | 128 | G01 | 25 µM BAM15 | 01:58:20 | 96.78 |
| 10 | 129 | G01 | 25 µM BAM15 | 01:58:34 | 91.53 |
| 10 | 130 | G01 | 25 µM BAM15 | 01:58:48 | 86.87 |
| 10 | 131 | G01 | 25 µM BAM15 | 01:59:03 | 82.45 |
| 10 | 132 | G01 | 25 µM BAM15 | 01:59:17 | 78.44 |
| 10 | 133 | G01 | 25 µM BAM15 | 01:59:31 | 74.67 |
| 10 | 134 | G01 | 25 µM BAM15 | 01:59:45 | 71.22 |
| 10 | 135 | G01 | 25 µM BAM15 | 01:59:59 | 67.86 |
| 10 | 136 | G01 | 25 µM BAM15 | 02:00:14 | 64.71 |
| 10 | 137 | G01 | 25 µM BAM15 | 02:00:28 | 61.73 |
| 10 | 138 | G01 | 25 µM BAM15 | 02:00:42 | 58.87 |
| 10 | 139 | G01 | 25 µM BAM15 | 02:00:56 | 56.19 |
| 11 | 140 | G01 | 25 µM BAM15 | 02:06:30 | 113.28 |
| 11 | 141 | G01 | 25 µM BAM15 | 02:06:44 | 103.69 |
| 11 | 142 | G01 | 25 µM BAM15 | 02:06:58 | 97.36 |
| 11 | 143 | G01 | 25 µM BAM15 | 02:07:12 | 92.11 |
| 11 | 144 | G01 | 25 µM BAM15 | 02:07:26 | 87.51 |
| 11 | 145 | G01 | 25 µM BAM15 | 02:07:41 | 83.11 |
| 11 | 146 | G01 | 25 µM BAM15 | 02:07:55 | 79.18 |
| 11 | 147 | G01 | 25 µM BAM15 | 02:08:09 | 75.49 |
| 11 | 148 | G01 | 25 µM BAM15 | 02:08:23 | 72.00 |
| 11 | 149 | G01 | 25 µM BAM15 | 02:08:37 | 68.70 |
| 11 | 150 | G01 | 25 µM BAM15 | 02:08:52 | 65.66 |
| 11 | 151 | G01 | 25 µM BAM15 | 02:09:06 | 62.67 |
| 11 | 152 | G01 | 25 µM BAM15 | 02:09:20 | 59.87 |
| 11 | 153 | G01 | 25 µM BAM15 | 02:09:34 | 57.19 |
| 12 | 154 | G01 | 25 µM BAM15 | 02:15:08 | 113.43 |
| 12 | 155 | G01 | 25 µM BAM15 | 02:15:22 | 103.87 |
| 12 | 156 | G01 | 25 µM BAM15 | 02:15:37 | 97.17 |
| 12 | 157 | G01 | 25 µM BAM15 | 02:15:53 | 91.48 |
| 12 | 158 | G01 | 25 µM BAM15 | 02:16:08 | 86.86 |
| 12 | 159 | G01 | 25 µM BAM15 | 02:16:22 | 82.80 |
| 12 | 160 | G01 | 25 µM BAM15 | 02:16:36 | 78.93 |
| 12 | 161 | G01 | 25 µM BAM15 | 02:16:50 | 75.31 |
| 12 | 162 | G01 | 25 µM BAM15 | 02:17:04 | 71.98 |
| 12 | 163 | G01 | 25 µM BAM15 | 02:17:18 | 68.69 |
| 12 | 164 | G01 | 25 µM BAM15 | 02:17:33 | 65.71 |
| 12 | 165 | G01 | 25 µM BAM15 | 02:17:47 | 62.85 |
| 12 | 166 | G01 | 25 µM BAM15 | 02:18:01 | 60.07 |
| 13 | 167 | G01 | 25 µM BAM15 | 02:23:39 | 110.84 |
| 13 | 168 | G01 | 25 µM BAM15 | 02:23:54 | 102.65 |
| 13 | 169 | G01 | 25 µM BAM15 | 02:24:08 | 96.69 |
| 13 | 170 | G01 | 25 µM BAM15 | 02:24:22 | 91.73 |
| 13 | 171 | G01 | 25 µM BAM15 | 02:24:36 | 87.28 |
| 13 | 172 | G01 | 25 µM BAM15 | 02:24:51 | 82.83 |
| 13 | 173 | G01 | 25 µM BAM15 | 02:25:07 | 78.63 |
| 13 | 174 | G01 | 25 µM BAM15 | 02:25:22 | 75.06 |
| 13 | 175 | G01 | 25 µM BAM15 | 02:25:36 | 71.89 |
| 13 | 176 | G01 | 25 µM BAM15 | 02:25:50 | 68.71 |
| 13 | 177 | G01 | 25 µM BAM15 | 02:26:04 | 65.80 |
| 13 | 178 | G01 | 25 µM BAM15 | 02:26:18 | 62.94 |
| 13 | 179 | G01 | 25 µM BAM15 | 02:26:33 | 60.28 |
| 14 | 180 | G01 | 25 µM BAM15 | 02:32:07 | 113.92 |
| 14 | 181 | G01 | 25 µM BAM15 | 02:32:21 | 104.62 |
| 14 | 182 | G01 | 25 µM BAM15 | 02:32:35 | 98.50 |
| 14 | 183 | G01 | 25 µM BAM15 | 02:32:50 | 93.42 |
| 14 | 184 | G01 | 25 µM BAM15 | 02:33:04 | 88.89 |
| 14 | 185 | G01 | 25 µM BAM15 | 02:33:18 | 84.69 |
| 14 | 186 | G01 | 25 µM BAM15 | 02:33:32 | 80.87 |
| 14 | 187 | G01 | 25 µM BAM15 | 02:33:46 | 77.26 |
| 14 | 188 | G01 | 25 µM BAM15 | 02:34:01 | 73.96 |
| 14 | 189 | G01 | 25 µM BAM15 | 02:34:15 | 70.81 |
| 14 | 190 | G01 | 25 µM BAM15 | 02:34:29 | 67.77 |
| 14 | 191 | G01 | 25 µM BAM15 | 02:34:43 | 64.92 |
| 14 | 192 | G01 | 25 µM BAM15 | 02:34:57 | 62.18 |
| 14 | 193 | G01 | 25 µM BAM15 | 02:35:12 | 59.66 |
| 15 | 194 | G01 | 25 µM BAM15 | 02:40:46 | 114.27 |
| 15 | 195 | G01 | 25 µM BAM15 | 02:41:00 | 104.92 |
| 15 | 196 | G01 | 25 µM BAM15 | 02:41:14 | 98.79 |
| 15 | 197 | G01 | 25 µM BAM15 | 02:41:28 | 93.76 |
| 15 | 198 | G01 | 25 µM BAM15 | 02:41:43 | 89.24 |
| 15 | 199 | G01 | 25 µM BAM15 | 02:41:57 | 85.18 |
| 15 | 200 | G01 | 25 µM BAM15 | 02:42:11 | 81.36 |
| 15 | 201 | G01 | 25 µM BAM15 | 02:42:25 | 77.78 |
| 15 | 202 | G01 | 25 µM BAM15 | 02:42:39 | 74.52 |
| 15 | 203 | G01 | 25 µM BAM15 | 02:42:53 | 71.37 |
| 15 | 204 | G01 | 25 µM BAM15 | 02:43:08 | 68.36 |
| 15 | 205 | G01 | 25 µM BAM15 | 02:43:22 | 65.64 |
| 15 | 206 | G01 | 25 µM BAM15 | 02:43:36 | 62.82 |
| 15 | 207 | G01 | 25 µM BAM15 | 02:43:50 | 60.30 |
| 16 | 208 | G01 | 25 µM BAM15 | 02:49:25 | 114.36 |
| 16 | 209 | G01 | 25 µM BAM15 | 02:49:40 | 105.25 |
| 16 | 210 | G01 | 25 µM BAM15 | 02:49:54 | 99.18 |
| 16 | 211 | G01 | 25 µM BAM15 | 02:50:08 | 94.16 |
| 16 | 212 | G01 | 25 µM BAM15 | 02:50:22 | 89.80 |
| 16 | 213 | G01 | 25 µM BAM15 | 02:50:36 | 85.58 |
| 16 | 214 | G01 | 25 µM BAM15 | 02:50:50 | 81.88 |
| 16 | 215 | G01 | 25 µM BAM15 | 02:51:05 | 78.31 |
| 16 | 216 | G01 | 25 µM BAM15 | 02:51:19 | 74.99 |
| 16 | 217 | G01 | 25 µM BAM15 | 02:51:33 | 71.94 |
| 16 | 218 | G01 | 25 µM BAM15 | 02:51:47 | 68.98 |
| 16 | 219 | G01 | 25 µM BAM15 | 02:52:01 | 66.22 |
| 16 | 220 | G01 | 25 µM BAM15 | 02:52:16 | 63.43 |
| 16 | 221 | G01 | 25 µM BAM15 | 02:52:30 | 60.88 |
| 1 | 0 | G02 | 25 µM BAM15 | 00:40:06 | 130.65 |
| 1 | 1 | G02 | 25 µM BAM15 | 00:40:20 | 126.45 |
| 1 | 2 | G02 | 25 µM BAM15 | 00:40:34 | 124.23 |
| 1 | 3 | G02 | 25 µM BAM15 | 00:40:48 | 122.28 |
| 1 | 4 | G02 | 25 µM BAM15 | 00:41:02 | 120.65 |
| 1 | 5 | G02 | 25 µM BAM15 | 00:41:17 | 119.12 |
| 1 | 6 | G02 | 25 µM BAM15 | 00:41:31 | 117.66 |
| 1 | 7 | G02 | 25 µM BAM15 | 00:41:45 | 116.46 |
| 1 | 8 | G02 | 25 µM BAM15 | 00:41:59 | 115.21 |
| 1 | 9 | G02 | 25 µM BAM15 | 00:42:13 | 114.09 |
| 1 | 10 | G02 | 25 µM BAM15 | 00:42:28 | 112.84 |
| 1 | 11 | G02 | 25 µM BAM15 | 00:42:42 | 111.80 |
| 1 | 12 | G02 | 25 µM BAM15 | 00:42:56 | 110.66 |
| 1 | 13 | G02 | 25 µM BAM15 | 00:43:10 | 109.63 |
| 2 | 14 | G02 | 25 µM BAM15 | 00:48:43 | 133.58 |
| 2 | 15 | G02 | 25 µM BAM15 | 00:48:57 | 130.23 |
| 2 | 16 | G02 | 25 µM BAM15 | 00:49:12 | 128.15 |
| 2 | 17 | G02 | 25 µM BAM15 | 00:49:26 | 126.30 |
| 2 | 18 | G02 | 25 µM BAM15 | 00:49:40 | 124.69 |
| 2 | 19 | G02 | 25 µM BAM15 | 00:49:54 | 123.08 |
| 2 | 20 | G02 | 25 µM BAM15 | 00:50:08 | 121.71 |
| 2 | 21 | G02 | 25 µM BAM15 | 00:50:22 | 120.29 |
| 2 | 22 | G02 | 25 µM BAM15 | 00:50:37 | 119.08 |
| 2 | 23 | G02 | 25 µM BAM15 | 00:50:51 | 117.85 |
| 2 | 24 | G02 | 25 µM BAM15 | 00:51:05 | 116.66 |
| 2 | 25 | G02 | 25 µM BAM15 | 00:51:19 | 115.56 |
| 2 | 26 | G02 | 25 µM BAM15 | 00:51:33 | 114.34 |
| 2 | 27 | G02 | 25 µM BAM15 | 00:51:48 | 113.37 |
| 3 | 28 | G02 | 25 µM BAM15 | 00:57:21 | 134.12 |
| 3 | 29 | G02 | 25 µM BAM15 | 00:57:35 | 130.81 |
| 3 | 30 | G02 | 25 µM BAM15 | 00:57:49 | 128.73 |
| 3 | 31 | G02 | 25 µM BAM15 | 00:58:03 | 126.90 |
| 3 | 32 | G02 | 25 µM BAM15 | 00:58:17 | 125.34 |
| 3 | 33 | G02 | 25 µM BAM15 | 00:58:31 | 123.81 |
| 3 | 34 | G02 | 25 µM BAM15 | 00:58:46 | 122.44 |
| 3 | 35 | G02 | 25 µM BAM15 | 00:59:00 | 121.17 |
| 3 | 36 | G02 | 25 µM BAM15 | 00:59:14 | 119.82 |
| 3 | 37 | G02 | 25 µM BAM15 | 00:59:28 | 118.61 |
| 3 | 38 | G02 | 25 µM BAM15 | 00:59:42 | 117.45 |
| 3 | 39 | G02 | 25 µM BAM15 | 00:59:56 | 116.36 |
| 3 | 40 | G02 | 25 µM BAM15 | 01:00:11 | 115.29 |
| 3 | 41 | G02 | 25 µM BAM15 | 01:00:25 | 114.23 |
| 4 | 42 | G02 | 25 µM BAM15 | 01:06:04 | 120.41 |
| 4 | 43 | G02 | 25 µM BAM15 | 01:06:18 | 109.46 |
| 4 | 44 | G02 | 25 µM BAM15 | 01:06:33 | 102.20 |
| 4 | 45 | G02 | 25 µM BAM15 | 01:06:47 | 96.22 |
| 4 | 46 | G02 | 25 µM BAM15 | 01:07:01 | 90.90 |
| 4 | 47 | G02 | 25 µM BAM15 | 01:07:15 | 85.84 |
| 4 | 48 | G02 | 25 µM BAM15 | 01:07:29 | 81.18 |
| 4 | 49 | G02 | 25 µM BAM15 | 01:07:43 | 76.79 |
| 4 | 50 | G02 | 25 µM BAM15 | 01:07:58 | 72.70 |
| 4 | 51 | G02 | 25 µM BAM15 | 01:08:12 | 68.83 |
| 4 | 52 | G02 | 25 µM BAM15 | 01:08:26 | 64.98 |
| 4 | 53 | G02 | 25 µM BAM15 | 01:08:40 | 61.50 |
| 4 | 54 | G02 | 25 µM BAM15 | 01:08:54 | 57.96 |
| 4 | 55 | G02 | 25 µM BAM15 | 01:09:09 | 54.70 |
| 5 | 56 | G02 | 25 µM BAM15 | 01:14:42 | 108.71 |
| 5 | 57 | G02 | 25 µM BAM15 | 01:14:56 | 95.71 |
| 5 | 58 | G02 | 25 µM BAM15 | 01:15:10 | 87.53 |
| 5 | 59 | G02 | 25 µM BAM15 | 01:15:25 | 80.94 |
| 5 | 60 | G02 | 25 µM BAM15 | 01:15:39 | 74.97 |
| 5 | 61 | G02 | 25 µM BAM15 | 01:15:53 | 69.37 |
| 5 | 62 | G02 | 25 µM BAM15 | 01:16:07 | 64.19 |
| 5 | 63 | G02 | 25 µM BAM15 | 01:16:21 | 59.29 |
| 5 | 64 | G02 | 25 µM BAM15 | 01:16:35 | 54.74 |
| 5 | 65 | G02 | 25 µM BAM15 | 01:16:50 | 50.33 |
| 5 | 66 | G02 | 25 µM BAM15 | 01:17:04 | 46.17 |
| 5 | 67 | G02 | 25 µM BAM15 | 01:17:18 | 42.42 |
| 5 | 68 | G02 | 25 µM BAM15 | 01:17:32 | 39.19 |
| 5 | 69 | G02 | 25 µM BAM15 | 01:17:46 | 36.52 |
| 6 | 70 | G02 | 25 µM BAM15 | 01:23:20 | 103.27 |
| 6 | 71 | G02 | 25 µM BAM15 | 01:23:34 | 89.42 |
| 6 | 72 | G02 | 25 µM BAM15 | 01:23:48 | 80.99 |
| 6 | 73 | G02 | 25 µM BAM15 | 01:24:02 | 73.98 |
| 6 | 74 | G02 | 25 µM BAM15 | 01:24:16 | 67.72 |
| 6 | 75 | G02 | 25 µM BAM15 | 01:24:30 | 61.81 |
| 6 | 76 | G02 | 25 µM BAM15 | 01:24:44 | 56.40 |
| 6 | 77 | G02 | 25 µM BAM15 | 01:24:59 | 51.14 |
| 6 | 78 | G02 | 25 µM BAM15 | 01:25:13 | 46.26 |
| 6 | 79 | G02 | 25 µM BAM15 | 01:25:27 | 41.79 |
| 6 | 80 | G02 | 25 µM BAM15 | 01:25:41 | 37.91 |
| 6 | 81 | G02 | 25 µM BAM15 | 01:25:55 | 34.89 |
| 6 | 82 | G02 | 25 µM BAM15 | 01:26:10 | 32.34 |
| 6 | 83 | G02 | 25 µM BAM15 | 01:26:24 | 30.32 |
| 7 | 84 | G02 | 25 µM BAM15 | 01:31:58 | 100.48 |
| 7 | 85 | G02 | 25 µM BAM15 | 01:32:12 | 86.55 |
| 7 | 86 | G02 | 25 µM BAM15 | 01:32:26 | 77.90 |
| 7 | 87 | G02 | 25 µM BAM15 | 01:32:40 | 70.84 |
| 7 | 88 | G02 | 25 µM BAM15 | 01:32:54 | 64.37 |
| 7 | 89 | G02 | 25 µM BAM15 | 01:33:08 | 58.35 |
| 7 | 90 | G02 | 25 µM BAM15 | 01:33:23 | 52.72 |
| 7 | 91 | G02 | 25 µM BAM15 | 01:33:37 | 47.30 |
| 7 | 92 | G02 | 25 µM BAM15 | 01:33:51 | 42.33 |
| 7 | 93 | G02 | 25 µM BAM15 | 01:34:05 | 38.03 |
| 7 | 94 | G02 | 25 µM BAM15 | 01:34:19 | 34.50 |
| 7 | 95 | G02 | 25 µM BAM15 | 01:34:33 | 31.85 |
| 7 | 96 | G02 | 25 µM BAM15 | 01:34:48 | 29.73 |
| 7 | 97 | G02 | 25 µM BAM15 | 01:35:02 | 28.11 |
| 8 | 98 | G02 | 25 µM BAM15 | 01:40:36 | 98.96 |
| 8 | 99 | G02 | 25 µM BAM15 | 01:40:50 | 84.90 |
| 8 | 100 | G02 | 25 µM BAM15 | 01:41:04 | 76.19 |
| 8 | 101 | G02 | 25 µM BAM15 | 01:41:18 | 68.93 |
| 8 | 102 | G02 | 25 µM BAM15 | 01:41:32 | 62.47 |
| 8 | 103 | G02 | 25 µM BAM15 | 01:41:46 | 56.36 |
| 8 | 104 | G02 | 25 µM BAM15 | 01:42:00 | 50.65 |
| 8 | 105 | G02 | 25 µM BAM15 | 01:42:15 | 45.16 |
| 8 | 106 | G02 | 25 µM BAM15 | 01:42:29 | 40.15 |
| 8 | 107 | G02 | 25 µM BAM15 | 01:42:43 | 35.98 |
| 8 | 108 | G02 | 25 µM BAM15 | 01:42:57 | 32.75 |
| 8 | 109 | G02 | 25 µM BAM15 | 01:43:11 | 30.30 |
| 8 | 110 | G02 | 25 µM BAM15 | 01:43:26 | 28.38 |
| 8 | 111 | G02 | 25 µM BAM15 | 01:43:40 | 27.04 |
| 9 | 112 | G02 | 25 µM BAM15 | 01:49:14 | 98.50 |
| 9 | 113 | G02 | 25 µM BAM15 | 01:49:28 | 84.36 |
| 9 | 114 | G02 | 25 µM BAM15 | 01:49:42 | 75.56 |
| 9 | 115 | G02 | 25 µM BAM15 | 01:49:56 | 68.24 |
| 9 | 116 | G02 | 25 µM BAM15 | 01:50:10 | 61.67 |
| 9 | 117 | G02 | 25 µM BAM15 | 01:50:24 | 55.47 |
| 9 | 118 | G02 | 25 µM BAM15 | 01:50:38 | 49.70 |
| 9 | 119 | G02 | 25 µM BAM15 | 01:50:53 | 44.10 |
| 9 | 120 | G02 | 25 µM BAM15 | 01:51:07 | 39.13 |
| 9 | 121 | G02 | 25 µM BAM15 | 01:51:21 | 35.08 |
| 9 | 122 | G02 | 25 µM BAM15 | 01:51:35 | 31.96 |
| 9 | 123 | G02 | 25 µM BAM15 | 01:51:50 | 29.62 |
| 9 | 124 | G02 | 25 µM BAM15 | 01:52:04 | 27.85 |
| 9 | 125 | G02 | 25 µM BAM15 | 01:52:18 | 26.53 |
| 10 | 126 | G02 | 25 µM BAM15 | 01:57:52 | 99.17 |
| 10 | 127 | G02 | 25 µM BAM15 | 01:58:06 | 86.44 |
| 10 | 128 | G02 | 25 µM BAM15 | 01:58:20 | 77.98 |
| 10 | 129 | G02 | 25 µM BAM15 | 01:58:34 | 70.93 |
| 10 | 130 | G02 | 25 µM BAM15 | 01:58:48 | 64.38 |
| 10 | 131 | G02 | 25 µM BAM15 | 01:59:03 | 58.14 |
| 10 | 132 | G02 | 25 µM BAM15 | 01:59:17 | 52.32 |
| 10 | 133 | G02 | 25 µM BAM15 | 01:59:31 | 46.67 |
| 10 | 134 | G02 | 25 µM BAM15 | 01:59:45 | 41.54 |
| 10 | 135 | G02 | 25 µM BAM15 | 01:59:59 | 36.81 |
| 10 | 136 | G02 | 25 µM BAM15 | 02:00:14 | 33.13 |
| 10 | 137 | G02 | 25 µM BAM15 | 02:00:28 | 30.41 |
| 10 | 138 | G02 | 25 µM BAM15 | 02:00:42 | 28.36 |
| 10 | 139 | G02 | 25 µM BAM15 | 02:00:56 | 26.88 |
| 11 | 140 | G02 | 25 µM BAM15 | 02:06:30 | 101.90 |
| 11 | 141 | G02 | 25 µM BAM15 | 02:06:44 | 89.60 |
| 11 | 142 | G02 | 25 µM BAM15 | 02:06:58 | 81.27 |
| 11 | 143 | G02 | 25 µM BAM15 | 02:07:12 | 74.16 |
| 11 | 144 | G02 | 25 µM BAM15 | 02:07:26 | 67.72 |
| 11 | 145 | G02 | 25 µM BAM15 | 02:07:41 | 61.49 |
| 11 | 146 | G02 | 25 µM BAM15 | 02:07:55 | 55.69 |
| 11 | 147 | G02 | 25 µM BAM15 | 02:08:09 | 50.11 |
| 11 | 148 | G02 | 25 µM BAM15 | 02:08:23 | 44.86 |
| 11 | 149 | G02 | 25 µM BAM15 | 02:08:37 | 39.98 |
| 11 | 150 | G02 | 25 µM BAM15 | 02:08:52 | 35.78 |
| 11 | 151 | G02 | 25 µM BAM15 | 02:09:06 | 32.49 |
| 11 | 152 | G02 | 25 µM BAM15 | 02:09:20 | 30.00 |
| 11 | 153 | G02 | 25 µM BAM15 | 02:09:34 | 28.06 |
| 12 | 154 | G02 | 25 µM BAM15 | 02:15:08 | 101.99 |
| 12 | 155 | G02 | 25 µM BAM15 | 02:15:22 | 89.94 |
| 12 | 156 | G02 | 25 µM BAM15 | 02:15:37 | 81.05 |
| 12 | 157 | G02 | 25 µM BAM15 | 02:15:53 | 73.27 |
| 12 | 158 | G02 | 25 µM BAM15 | 02:16:08 | 66.81 |
| 12 | 159 | G02 | 25 µM BAM15 | 02:16:22 | 60.92 |
| 12 | 160 | G02 | 25 µM BAM15 | 02:16:36 | 55.28 |
| 12 | 161 | G02 | 25 µM BAM15 | 02:16:50 | 49.82 |
| 12 | 162 | G02 | 25 µM BAM15 | 02:17:04 | 44.74 |
| 12 | 163 | G02 | 25 µM BAM15 | 02:17:18 | 39.94 |
| 12 | 164 | G02 | 25 µM BAM15 | 02:17:33 | 35.98 |
| 12 | 165 | G02 | 25 µM BAM15 | 02:17:47 | 32.80 |
| 12 | 166 | G02 | 25 µM BAM15 | 02:18:01 | 30.25 |
| 13 | 167 | G02 | 25 µM BAM15 | 02:23:39 | 98.74 |
| 13 | 168 | G02 | 25 µM BAM15 | 02:23:54 | 88.29 |
| 13 | 169 | G02 | 25 µM BAM15 | 02:24:08 | 80.44 |
| 13 | 170 | G02 | 25 µM BAM15 | 02:24:22 | 73.61 |
| 13 | 171 | G02 | 25 µM BAM15 | 02:24:36 | 67.32 |
| 13 | 172 | G02 | 25 µM BAM15 | 02:24:51 | 60.93 |
| 13 | 173 | G02 | 25 µM BAM15 | 02:25:07 | 54.74 |
| 13 | 174 | G02 | 25 µM BAM15 | 02:25:22 | 49.43 |
| 13 | 175 | G02 | 25 µM BAM15 | 02:25:36 | 44.60 |
| 13 | 176 | G02 | 25 µM BAM15 | 02:25:50 | 39.98 |
| 13 | 177 | G02 | 25 µM BAM15 | 02:26:04 | 36.14 |
| 13 | 178 | G02 | 25 µM BAM15 | 02:26:18 | 32.93 |
| 13 | 179 | G02 | 25 µM BAM15 | 02:26:33 | 30.41 |
| 14 | 180 | G02 | 25 µM BAM15 | 02:32:07 | 102.86 |
| 14 | 181 | G02 | 25 µM BAM15 | 02:32:21 | 90.93 |
| 14 | 182 | G02 | 25 µM BAM15 | 02:32:35 | 82.90 |
| 14 | 183 | G02 | 25 µM BAM15 | 02:32:50 | 76.05 |
| 14 | 184 | G02 | 25 µM BAM15 | 02:33:04 | 69.76 |
| 14 | 185 | G02 | 25 µM BAM15 | 02:33:18 | 63.80 |
| 14 | 186 | G02 | 25 µM BAM15 | 02:33:32 | 58.25 |
| 14 | 187 | G02 | 25 µM BAM15 | 02:33:46 | 52.89 |
| 14 | 188 | G02 | 25 µM BAM15 | 02:34:01 | 47.97 |
| 14 | 189 | G02 | 25 µM BAM15 | 02:34:15 | 43.30 |
| 14 | 190 | G02 | 25 µM BAM15 | 02:34:29 | 39.03 |
| 14 | 191 | G02 | 25 µM BAM15 | 02:34:43 | 35.39 |
| 14 | 192 | G02 | 25 µM BAM15 | 02:34:57 | 32.39 |
| 14 | 193 | G02 | 25 µM BAM15 | 02:35:12 | 30.09 |
| 15 | 194 | G02 | 25 µM BAM15 | 02:40:46 | 103.28 |
| 15 | 195 | G02 | 25 µM BAM15 | 02:41:00 | 91.60 |
| 15 | 196 | G02 | 25 µM BAM15 | 02:41:14 | 83.55 |
| 15 | 197 | G02 | 25 µM BAM15 | 02:41:28 | 76.80 |
| 15 | 198 | G02 | 25 µM BAM15 | 02:41:43 | 70.57 |
| 15 | 199 | G02 | 25 µM BAM15 | 02:41:57 | 64.80 |
| 15 | 200 | G02 | 25 µM BAM15 | 02:42:11 | 59.34 |
| 15 | 201 | G02 | 25 µM BAM15 | 02:42:25 | 54.12 |
| 15 | 202 | G02 | 25 µM BAM15 | 02:42:39 | 49.28 |
| 15 | 203 | G02 | 25 µM BAM15 | 02:42:53 | 44.69 |
| 15 | 204 | G02 | 25 µM BAM15 | 02:43:08 | 40.30 |
| 15 | 205 | G02 | 25 µM BAM15 | 02:43:22 | 36.65 |
| 15 | 206 | G02 | 25 µM BAM15 | 02:43:36 | 33.40 |
| 15 | 207 | G02 | 25 µM BAM15 | 02:43:50 | 30.91 |
| 16 | 208 | G02 | 25 µM BAM15 | 02:49:25 | 103.85 |
| 16 | 209 | G02 | 25 µM BAM15 | 02:49:40 | 92.34 |
| 16 | 210 | G02 | 25 µM BAM15 | 02:49:54 | 84.48 |
| 16 | 211 | G02 | 25 µM BAM15 | 02:50:08 | 77.80 |
| 16 | 212 | G02 | 25 µM BAM15 | 02:50:22 | 71.67 |
| 16 | 213 | G02 | 25 µM BAM15 | 02:50:36 | 65.87 |
| 16 | 214 | G02 | 25 µM BAM15 | 02:50:50 | 60.56 |
| 16 | 215 | G02 | 25 µM BAM15 | 02:51:05 | 55.32 |
| 16 | 216 | G02 | 25 µM BAM15 | 02:51:19 | 50.58 |
| 16 | 217 | G02 | 25 µM BAM15 | 02:51:33 | 46.02 |
| 16 | 218 | G02 | 25 µM BAM15 | 02:51:47 | 41.76 |
| 16 | 219 | G02 | 25 µM BAM15 | 02:52:01 | 37.94 |
| 16 | 220 | G02 | 25 µM BAM15 | 02:52:16 | 34.56 |
| 16 | 221 | G02 | 25 µM BAM15 | 02:52:30 | 31.85 |
| 1 | 0 | G03 | 25 µM BAM15 | 00:40:06 | 129.22 |
| 1 | 1 | G03 | 25 µM BAM15 | 00:40:20 | 123.93 |
| 1 | 2 | G03 | 25 µM BAM15 | 00:40:34 | 121.44 |
| 1 | 3 | G03 | 25 µM BAM15 | 00:40:48 | 119.57 |
| 1 | 4 | G03 | 25 µM BAM15 | 00:41:02 | 117.90 |
| 1 | 5 | G03 | 25 µM BAM15 | 00:41:17 | 116.44 |
| 1 | 6 | G03 | 25 µM BAM15 | 00:41:31 | 115.15 |
| 1 | 7 | G03 | 25 µM BAM15 | 00:41:45 | 113.86 |
| 1 | 8 | G03 | 25 µM BAM15 | 00:41:59 | 112.71 |
| 1 | 9 | G03 | 25 µM BAM15 | 00:42:13 | 111.57 |
| 1 | 10 | G03 | 25 µM BAM15 | 00:42:28 | 110.42 |
| 1 | 11 | G03 | 25 µM BAM15 | 00:42:42 | 109.39 |
| 1 | 12 | G03 | 25 µM BAM15 | 00:42:56 | 108.43 |
| 1 | 13 | G03 | 25 µM BAM15 | 00:43:10 | 107.45 |
| 2 | 14 | G03 | 25 µM BAM15 | 00:48:43 | 133.93 |
| 2 | 15 | G03 | 25 µM BAM15 | 00:48:57 | 129.97 |
| 2 | 16 | G03 | 25 µM BAM15 | 00:49:12 | 127.62 |
| 2 | 17 | G03 | 25 µM BAM15 | 00:49:26 | 125.64 |
| 2 | 18 | G03 | 25 µM BAM15 | 00:49:40 | 123.98 |
| 2 | 19 | G03 | 25 µM BAM15 | 00:49:54 | 122.27 |
| 2 | 20 | G03 | 25 µM BAM15 | 00:50:08 | 120.82 |
| 2 | 21 | G03 | 25 µM BAM15 | 00:50:22 | 119.41 |
| 2 | 22 | G03 | 25 µM BAM15 | 00:50:37 | 118.21 |
| 2 | 23 | G03 | 25 µM BAM15 | 00:50:51 | 116.89 |
| 2 | 24 | G03 | 25 µM BAM15 | 00:51:05 | 115.68 |
| 2 | 25 | G03 | 25 µM BAM15 | 00:51:19 | 114.58 |
| 2 | 26 | G03 | 25 µM BAM15 | 00:51:33 | 113.46 |
| 2 | 27 | G03 | 25 µM BAM15 | 00:51:48 | 112.36 |
| 3 | 28 | G03 | 25 µM BAM15 | 00:57:21 | 134.73 |
| 3 | 29 | G03 | 25 µM BAM15 | 00:57:35 | 131.22 |
| 3 | 30 | G03 | 25 µM BAM15 | 00:57:49 | 128.90 |
| 3 | 31 | G03 | 25 µM BAM15 | 00:58:03 | 126.96 |
| 3 | 32 | G03 | 25 µM BAM15 | 00:58:17 | 125.34 |
| 3 | 33 | G03 | 25 µM BAM15 | 00:58:31 | 123.73 |
| 3 | 34 | G03 | 25 µM BAM15 | 00:58:46 | 122.21 |
| 3 | 35 | G03 | 25 µM BAM15 | 00:59:00 | 120.93 |
| 3 | 36 | G03 | 25 µM BAM15 | 00:59:14 | 119.54 |
| 3 | 37 | G03 | 25 µM BAM15 | 00:59:28 | 118.41 |
| 3 | 38 | G03 | 25 µM BAM15 | 00:59:42 | 117.14 |
| 3 | 39 | G03 | 25 µM BAM15 | 00:59:56 | 116.03 |
| 3 | 40 | G03 | 25 µM BAM15 | 01:00:11 | 114.94 |
| 3 | 41 | G03 | 25 µM BAM15 | 01:00:25 | 113.85 |
| 4 | 42 | G03 | 25 µM BAM15 | 01:06:04 | 122.50 |
| 4 | 43 | G03 | 25 µM BAM15 | 01:06:18 | 110.78 |
| 4 | 44 | G03 | 25 µM BAM15 | 01:06:33 | 103.37 |
| 4 | 45 | G03 | 25 µM BAM15 | 01:06:47 | 97.43 |
| 4 | 46 | G03 | 25 µM BAM15 | 01:07:01 | 92.22 |
| 4 | 47 | G03 | 25 µM BAM15 | 01:07:15 | 87.37 |
| 4 | 48 | G03 | 25 µM BAM15 | 01:07:29 | 82.91 |
| 4 | 49 | G03 | 25 µM BAM15 | 01:07:43 | 78.70 |
| 4 | 50 | G03 | 25 µM BAM15 | 01:07:58 | 74.87 |
| 4 | 51 | G03 | 25 µM BAM15 | 01:08:12 | 71.24 |
| 4 | 52 | G03 | 25 µM BAM15 | 01:08:26 | 67.74 |
| 4 | 53 | G03 | 25 µM BAM15 | 01:08:40 | 64.51 |
| 4 | 54 | G03 | 25 µM BAM15 | 01:08:54 | 61.30 |
| 4 | 55 | G03 | 25 µM BAM15 | 01:09:09 | 58.35 |
| 5 | 56 | G03 | 25 µM BAM15 | 01:14:42 | 112.07 |
| 5 | 57 | G03 | 25 µM BAM15 | 01:14:56 | 98.34 |
| 5 | 58 | G03 | 25 µM BAM15 | 01:15:10 | 90.15 |
| 5 | 59 | G03 | 25 µM BAM15 | 01:15:25 | 83.60 |
| 5 | 60 | G03 | 25 µM BAM15 | 01:15:39 | 77.86 |
| 5 | 61 | G03 | 25 µM BAM15 | 01:15:53 | 72.52 |
| 5 | 62 | G03 | 25 µM BAM15 | 01:16:07 | 67.70 |
| 5 | 63 | G03 | 25 µM BAM15 | 01:16:21 | 63.12 |
| 5 | 64 | G03 | 25 µM BAM15 | 01:16:35 | 58.91 |
| 5 | 65 | G03 | 25 µM BAM15 | 01:16:50 | 54.87 |
| 5 | 66 | G03 | 25 µM BAM15 | 01:17:04 | 51.00 |
| 5 | 67 | G03 | 25 µM BAM15 | 01:17:18 | 47.44 |
| 5 | 68 | G03 | 25 µM BAM15 | 01:17:32 | 44.09 |
| 5 | 69 | G03 | 25 µM BAM15 | 01:17:46 | 41.14 |
| 6 | 70 | G03 | 25 µM BAM15 | 01:23:20 | 106.85 |
| 6 | 71 | G03 | 25 µM BAM15 | 01:23:34 | 92.09 |
| 6 | 72 | G03 | 25 µM BAM15 | 01:23:48 | 83.48 |
| 6 | 73 | G03 | 25 µM BAM15 | 01:24:02 | 76.47 |
| 6 | 74 | G03 | 25 µM BAM15 | 01:24:16 | 70.35 |
| 6 | 75 | G03 | 25 µM BAM15 | 01:24:30 | 64.65 |
| 6 | 76 | G03 | 25 µM BAM15 | 01:24:44 | 59.53 |
| 6 | 77 | G03 | 25 µM BAM15 | 01:24:59 | 54.56 |
| 6 | 78 | G03 | 25 µM BAM15 | 01:25:13 | 50.05 |
| 6 | 79 | G03 | 25 µM BAM15 | 01:25:27 | 45.81 |
| 6 | 80 | G03 | 25 µM BAM15 | 01:25:41 | 41.90 |
| 6 | 81 | G03 | 25 µM BAM15 | 01:25:55 | 38.58 |
| 6 | 82 | G03 | 25 µM BAM15 | 01:26:10 | 35.56 |
| 6 | 83 | G03 | 25 µM BAM15 | 01:26:24 | 32.96 |
| 7 | 84 | G03 | 25 µM BAM15 | 01:31:58 | 104.05 |
| 7 | 85 | G03 | 25 µM BAM15 | 01:32:12 | 89.07 |
| 7 | 86 | G03 | 25 µM BAM15 | 01:32:26 | 80.15 |
| 7 | 87 | G03 | 25 µM BAM15 | 01:32:40 | 73.09 |
| 7 | 88 | G03 | 25 µM BAM15 | 01:32:54 | 66.70 |
| 7 | 89 | G03 | 25 µM BAM15 | 01:33:08 | 60.86 |
| 7 | 90 | G03 | 25 µM BAM15 | 01:33:23 | 55.50 |
| 7 | 91 | G03 | 25 µM BAM15 | 01:33:37 | 50.37 |
| 7 | 92 | G03 | 25 µM BAM15 | 01:33:51 | 45.74 |
| 7 | 93 | G03 | 25 µM BAM15 | 01:34:05 | 41.55 |
| 7 | 94 | G03 | 25 µM BAM15 | 01:34:19 | 37.80 |
| 7 | 95 | G03 | 25 µM BAM15 | 01:34:33 | 34.69 |
| 7 | 96 | G03 | 25 µM BAM15 | 01:34:48 | 31.92 |
| 7 | 97 | G03 | 25 µM BAM15 | 01:35:02 | 29.62 |
| 8 | 98 | G03 | 25 µM BAM15 | 01:40:36 | 102.90 |
| 8 | 99 | G03 | 25 µM BAM15 | 01:40:50 | 87.71 |
| 8 | 100 | G03 | 25 µM BAM15 | 01:41:04 | 78.66 |
| 8 | 101 | G03 | 25 µM BAM15 | 01:41:18 | 71.36 |
| 8 | 102 | G03 | 25 µM BAM15 | 01:41:32 | 64.97 |
| 8 | 103 | G03 | 25 µM BAM15 | 01:41:46 | 58.99 |
| 8 | 104 | G03 | 25 µM BAM15 | 01:42:00 | 53.52 |
| 8 | 105 | G03 | 25 µM BAM15 | 01:42:15 | 48.28 |
| 8 | 106 | G03 | 25 µM BAM15 | 01:42:29 | 43.52 |
| 8 | 107 | G03 | 25 µM BAM15 | 01:42:43 | 39.32 |
| 8 | 108 | G03 | 25 µM BAM15 | 01:42:57 | 35.65 |
| 8 | 109 | G03 | 25 µM BAM15 | 01:43:11 | 32.58 |
| 8 | 110 | G03 | 25 µM BAM15 | 01:43:26 | 29.92 |
| 8 | 111 | G03 | 25 µM BAM15 | 01:43:40 | 27.89 |
| 9 | 112 | G03 | 25 µM BAM15 | 01:49:14 | 102.35 |
| 9 | 113 | G03 | 25 µM BAM15 | 01:49:28 | 87.19 |
| 9 | 114 | G03 | 25 µM BAM15 | 01:49:42 | 78.05 |
| 9 | 115 | G03 | 25 µM BAM15 | 01:49:56 | 70.75 |
| 9 | 116 | G03 | 25 µM BAM15 | 01:50:10 | 64.21 |
| 9 | 117 | G03 | 25 µM BAM15 | 01:50:24 | 58.21 |
| 9 | 118 | G03 | 25 µM BAM15 | 01:50:38 | 52.63 |
| 9 | 119 | G03 | 25 µM BAM15 | 01:50:53 | 47.30 |
| 9 | 120 | G03 | 25 µM BAM15 | 01:51:07 | 42.52 |
| 9 | 121 | G03 | 25 µM BAM15 | 01:51:21 | 38.34 |
| 9 | 122 | G03 | 25 µM BAM15 | 01:51:35 | 34.75 |
| 9 | 123 | G03 | 25 µM BAM15 | 01:51:50 | 31.69 |
| 9 | 124 | G03 | 25 µM BAM15 | 01:52:04 | 29.16 |
| 9 | 125 | G03 | 25 µM BAM15 | 01:52:18 | 27.23 |
| 10 | 126 | G03 | 25 µM BAM15 | 01:57:52 | 101.88 |
| 10 | 127 | G03 | 25 µM BAM15 | 01:58:06 | 87.84 |
| 10 | 128 | G03 | 25 µM BAM15 | 01:58:20 | 78.99 |
| 10 | 129 | G03 | 25 µM BAM15 | 01:58:34 | 71.85 |
| 10 | 130 | G03 | 25 µM BAM15 | 01:58:48 | 65.39 |
| 10 | 131 | G03 | 25 µM BAM15 | 01:59:03 | 59.30 |
| 10 | 132 | G03 | 25 µM BAM15 | 01:59:17 | 53.75 |
| 10 | 133 | G03 | 25 µM BAM15 | 01:59:31 | 48.44 |
| 10 | 134 | G03 | 25 µM BAM15 | 01:59:45 | 43.66 |
| 10 | 135 | G03 | 25 µM BAM15 | 01:59:59 | 39.26 |
| 10 | 136 | G03 | 25 µM BAM15 | 02:00:14 | 35.47 |
| 10 | 137 | G03 | 25 µM BAM15 | 02:00:28 | 32.31 |
| 10 | 138 | G03 | 25 µM BAM15 | 02:00:42 | 29.63 |
| 10 | 139 | G03 | 25 µM BAM15 | 02:00:56 | 27.59 |
| 11 | 140 | G03 | 25 µM BAM15 | 02:06:30 | 104.89 |
| 11 | 141 | G03 | 25 µM BAM15 | 02:06:44 | 91.89 |
| 11 | 142 | G03 | 25 µM BAM15 | 02:06:58 | 83.31 |
| 11 | 143 | G03 | 25 µM BAM15 | 02:07:12 | 76.20 |
| 11 | 144 | G03 | 25 µM BAM15 | 02:07:26 | 69.81 |
| 11 | 145 | G03 | 25 µM BAM15 | 02:07:41 | 63.76 |
| 11 | 146 | G03 | 25 µM BAM15 | 02:07:55 | 58.16 |
| 11 | 147 | G03 | 25 µM BAM15 | 02:08:09 | 52.85 |
| 11 | 148 | G03 | 25 µM BAM15 | 02:08:23 | 47.86 |
| 11 | 149 | G03 | 25 µM BAM15 | 02:08:37 | 43.20 |
| 11 | 150 | G03 | 25 µM BAM15 | 02:08:52 | 38.97 |
| 11 | 151 | G03 | 25 µM BAM15 | 02:09:06 | 35.35 |
| 11 | 152 | G03 | 25 µM BAM15 | 02:09:20 | 32.25 |
| 11 | 153 | G03 | 25 µM BAM15 | 02:09:34 | 29.64 |
| 12 | 154 | G03 | 25 µM BAM15 | 02:15:08 | 105.19 |
| 12 | 155 | G03 | 25 µM BAM15 | 02:15:22 | 92.34 |
| 12 | 156 | G03 | 25 µM BAM15 | 02:15:37 | 83.22 |
| 12 | 157 | G03 | 25 µM BAM15 | 02:15:53 | 75.55 |
| 12 | 158 | G03 | 25 µM BAM15 | 02:16:08 | 69.18 |
| 12 | 159 | G03 | 25 µM BAM15 | 02:16:22 | 63.46 |
| 12 | 160 | G03 | 25 µM BAM15 | 02:16:36 | 58.03 |
| 12 | 161 | G03 | 25 µM BAM15 | 02:16:50 | 52.88 |
| 12 | 162 | G03 | 25 µM BAM15 | 02:17:04 | 48.02 |
| 12 | 163 | G03 | 25 µM BAM15 | 02:17:18 | 43.45 |
| 12 | 164 | G03 | 25 µM BAM15 | 02:17:33 | 39.35 |
| 12 | 165 | G03 | 25 µM BAM15 | 02:17:47 | 35.76 |
| 12 | 166 | G03 | 25 µM BAM15 | 02:18:01 | 32.68 |
| 13 | 167 | G03 | 25 µM BAM15 | 02:23:39 | 101.79 |
| 13 | 168 | G03 | 25 µM BAM15 | 02:23:54 | 90.84 |
| 13 | 169 | G03 | 25 µM BAM15 | 02:24:08 | 82.83 |
| 13 | 170 | G03 | 25 µM BAM15 | 02:24:22 | 76.09 |
| 13 | 171 | G03 | 25 µM BAM15 | 02:24:36 | 69.91 |
| 13 | 172 | G03 | 25 µM BAM15 | 02:24:51 | 63.70 |
| 13 | 173 | G03 | 25 µM BAM15 | 02:25:07 | 57.75 |
| 13 | 174 | G03 | 25 µM BAM15 | 02:25:22 | 52.76 |
| 13 | 175 | G03 | 25 µM BAM15 | 02:25:36 | 48.16 |
| 13 | 176 | G03 | 25 µM BAM15 | 02:25:50 | 43.65 |
| 13 | 177 | G03 | 25 µM BAM15 | 02:26:04 | 39.69 |
| 13 | 178 | G03 | 25 µM BAM15 | 02:26:18 | 36.15 |
| 13 | 179 | G03 | 25 µM BAM15 | 02:26:33 | 33.08 |
| 14 | 180 | G03 | 25 µM BAM15 | 02:32:07 | 106.05 |
| 14 | 181 | G03 | 25 µM BAM15 | 02:32:21 | 93.44 |
| 14 | 182 | G03 | 25 µM BAM15 | 02:32:35 | 85.21 |
| 14 | 183 | G03 | 25 µM BAM15 | 02:32:50 | 78.31 |
| 14 | 184 | G03 | 25 µM BAM15 | 02:33:04 | 72.09 |
| 14 | 185 | G03 | 25 µM BAM15 | 02:33:18 | 66.28 |
| 14 | 186 | G03 | 25 µM BAM15 | 02:33:32 | 60.96 |
| 14 | 187 | G03 | 25 µM BAM15 | 02:33:46 | 55.83 |
| 14 | 188 | G03 | 25 µM BAM15 | 02:34:01 | 51.12 |
| 14 | 189 | G03 | 25 µM BAM15 | 02:34:15 | 46.65 |
| 14 | 190 | G03 | 25 µM BAM15 | 02:34:29 | 42.44 |
| 14 | 191 | G03 | 25 µM BAM15 | 02:34:43 | 38.66 |
| 14 | 192 | G03 | 25 µM BAM15 | 02:34:57 | 35.29 |
| 14 | 193 | G03 | 25 µM BAM15 | 02:35:12 | 32.54 |
| 15 | 194 | G03 | 25 µM BAM15 | 02:40:46 | 106.25 |
| 15 | 195 | G03 | 25 µM BAM15 | 02:41:00 | 93.81 |
| 15 | 196 | G03 | 25 µM BAM15 | 02:41:14 | 85.57 |
| 15 | 197 | G03 | 25 µM BAM15 | 02:41:28 | 78.74 |
| 15 | 198 | G03 | 25 µM BAM15 | 02:41:43 | 72.59 |
| 15 | 199 | G03 | 25 µM BAM15 | 02:41:57 | 66.92 |
| 15 | 200 | G03 | 25 µM BAM15 | 02:42:11 | 61.62 |
| 15 | 201 | G03 | 25 µM BAM15 | 02:42:25 | 56.61 |
| 15 | 202 | G03 | 25 µM BAM15 | 02:42:39 | 51.98 |
| 15 | 203 | G03 | 25 µM BAM15 | 02:42:53 | 47.54 |
| 15 | 204 | G03 | 25 µM BAM15 | 02:43:08 | 43.30 |
| 15 | 205 | G03 | 25 µM BAM15 | 02:43:22 | 39.59 |
| 15 | 206 | G03 | 25 µM BAM15 | 02:43:36 | 36.17 |
| 15 | 207 | G03 | 25 µM BAM15 | 02:43:50 | 33.32 |
| 16 | 208 | G03 | 25 µM BAM15 | 02:49:25 | 106.63 |
| 16 | 209 | G03 | 25 µM BAM15 | 02:49:40 | 94.26 |
| 16 | 210 | G03 | 25 µM BAM15 | 02:49:54 | 86.19 |
| 16 | 211 | G03 | 25 µM BAM15 | 02:50:08 | 79.34 |
| 16 | 212 | G03 | 25 µM BAM15 | 02:50:22 | 73.30 |
| 16 | 213 | G03 | 25 µM BAM15 | 02:50:36 | 67.64 |
| 16 | 214 | G03 | 25 µM BAM15 | 02:50:50 | 62.40 |
| 16 | 215 | G03 | 25 µM BAM15 | 02:51:05 | 57.37 |
| 16 | 216 | G03 | 25 µM BAM15 | 02:51:19 | 52.74 |
| 16 | 217 | G03 | 25 µM BAM15 | 02:51:33 | 48.39 |
| 16 | 218 | G03 | 25 µM BAM15 | 02:51:47 | 44.23 |
| 16 | 219 | G03 | 25 µM BAM15 | 02:52:01 | 40.52 |
| 16 | 220 | G03 | 25 µM BAM15 | 02:52:16 | 37.03 |
| 16 | 221 | G03 | 25 µM BAM15 | 02:52:30 | 34.11 |
| 1 | 0 | G04 | 25 µM FCCP | 00:40:06 | 126.10 |
| 1 | 1 | G04 | 25 µM FCCP | 00:40:20 | 121.03 |
| 1 | 2 | G04 | 25 µM FCCP | 00:40:34 | 118.54 |
| 1 | 3 | G04 | 25 µM FCCP | 00:40:48 | 116.65 |
| 1 | 4 | G04 | 25 µM FCCP | 00:41:02 | 114.91 |
| 1 | 5 | G04 | 25 µM FCCP | 00:41:17 | 113.41 |
| 1 | 6 | G04 | 25 µM FCCP | 00:41:31 | 111.90 |
| 1 | 7 | G04 | 25 µM FCCP | 00:41:45 | 110.62 |
| 1 | 8 | G04 | 25 µM FCCP | 00:41:59 | 109.31 |
| 1 | 9 | G04 | 25 µM FCCP | 00:42:13 | 108.19 |
| 1 | 10 | G04 | 25 µM FCCP | 00:42:28 | 106.96 |
| 1 | 11 | G04 | 25 µM FCCP | 00:42:42 | 105.83 |
| 1 | 12 | G04 | 25 µM FCCP | 00:42:56 | 104.72 |
| 1 | 13 | G04 | 25 µM FCCP | 00:43:10 | 103.74 |
| 2 | 14 | G04 | 25 µM FCCP | 00:48:43 | 130.69 |
| 2 | 15 | G04 | 25 µM FCCP | 00:48:57 | 126.33 |
| 2 | 16 | G04 | 25 µM FCCP | 00:49:12 | 123.75 |
| 2 | 17 | G04 | 25 µM FCCP | 00:49:26 | 121.56 |
| 2 | 18 | G04 | 25 µM FCCP | 00:49:40 | 119.68 |
| 2 | 19 | G04 | 25 µM FCCP | 00:49:54 | 117.93 |
| 2 | 20 | G04 | 25 µM FCCP | 00:50:08 | 116.37 |
| 2 | 21 | G04 | 25 µM FCCP | 00:50:22 | 114.90 |
| 2 | 22 | G04 | 25 µM FCCP | 00:50:37 | 113.46 |
| 2 | 23 | G04 | 25 µM FCCP | 00:50:51 | 112.18 |
| 2 | 24 | G04 | 25 µM FCCP | 00:51:05 | 110.89 |
| 2 | 25 | G04 | 25 µM FCCP | 00:51:19 | 109.75 |
| 2 | 26 | G04 | 25 µM FCCP | 00:51:33 | 108.47 |
| 2 | 27 | G04 | 25 µM FCCP | 00:51:48 | 107.43 |
| 3 | 28 | G04 | 25 µM FCCP | 00:57:21 | 129.46 |
| 3 | 29 | G04 | 25 µM FCCP | 00:57:35 | 125.47 |
| 3 | 30 | G04 | 25 µM FCCP | 00:57:49 | 123.07 |
| 3 | 31 | G04 | 25 µM FCCP | 00:58:03 | 121.08 |
| 3 | 32 | G04 | 25 µM FCCP | 00:58:17 | 119.35 |
| 3 | 33 | G04 | 25 µM FCCP | 00:58:31 | 117.70 |
| 3 | 34 | G04 | 25 µM FCCP | 00:58:46 | 116.19 |
| 3 | 35 | G04 | 25 µM FCCP | 00:59:00 | 114.78 |
| 3 | 36 | G04 | 25 µM FCCP | 00:59:14 | 113.45 |
| 3 | 37 | G04 | 25 µM FCCP | 00:59:28 | 112.17 |
| 3 | 38 | G04 | 25 µM FCCP | 00:59:42 | 110.89 |
| 3 | 39 | G04 | 25 µM FCCP | 00:59:56 | 109.81 |
| 3 | 40 | G04 | 25 µM FCCP | 01:00:11 | 108.64 |
| 3 | 41 | G04 | 25 µM FCCP | 01:00:25 | 107.58 |
| 4 | 42 | G04 | 25 µM FCCP | 01:06:04 | 131.52 |
| 4 | 43 | G04 | 25 µM FCCP | 01:06:18 | 127.47 |
| 4 | 44 | G04 | 25 µM FCCP | 01:06:33 | 124.91 |
| 4 | 45 | G04 | 25 µM FCCP | 01:06:47 | 122.99 |
| 4 | 46 | G04 | 25 µM FCCP | 01:07:01 | 121.24 |
| 4 | 47 | G04 | 25 µM FCCP | 01:07:15 | 119.60 |
| 4 | 48 | G04 | 25 µM FCCP | 01:07:29 | 118.02 |
| 4 | 49 | G04 | 25 µM FCCP | 01:07:43 | 116.61 |
| 4 | 50 | G04 | 25 µM FCCP | 01:07:58 | 115.25 |
| 4 | 51 | G04 | 25 µM FCCP | 01:08:12 | 113.86 |
| 4 | 52 | G04 | 25 µM FCCP | 01:08:26 | 112.46 |
| 4 | 53 | G04 | 25 µM FCCP | 01:08:40 | 111.24 |
| 4 | 54 | G04 | 25 µM FCCP | 01:08:54 | 109.95 |
| 4 | 55 | G04 | 25 µM FCCP | 01:09:09 | 108.80 |
| 5 | 56 | G04 | 25 µM FCCP | 01:14:42 | 130.57 |
| 5 | 57 | G04 | 25 µM FCCP | 01:14:56 | 126.06 |
| 5 | 58 | G04 | 25 µM FCCP | 01:15:10 | 123.43 |
| 5 | 59 | G04 | 25 µM FCCP | 01:15:25 | 121.37 |
| 5 | 60 | G04 | 25 µM FCCP | 01:15:39 | 119.65 |
| 5 | 61 | G04 | 25 µM FCCP | 01:15:53 | 117.98 |
| 5 | 62 | G04 | 25 µM FCCP | 01:16:07 | 116.35 |
| 5 | 63 | G04 | 25 µM FCCP | 01:16:21 | 114.88 |
| 5 | 64 | G04 | 25 µM FCCP | 01:16:35 | 113.52 |
| 5 | 65 | G04 | 25 µM FCCP | 01:16:50 | 112.16 |
| 5 | 66 | G04 | 25 µM FCCP | 01:17:04 | 110.88 |
| 5 | 67 | G04 | 25 µM FCCP | 01:17:18 | 109.63 |
| 5 | 68 | G04 | 25 µM FCCP | 01:17:32 | 108.40 |
| 5 | 69 | G04 | 25 µM FCCP | 01:17:46 | 107.23 |
| 6 | 70 | G04 | 25 µM FCCP | 01:23:20 | 131.14 |
| 6 | 71 | G04 | 25 µM FCCP | 01:23:34 | 126.58 |
| 6 | 72 | G04 | 25 µM FCCP | 01:23:48 | 124.12 |
| 6 | 73 | G04 | 25 µM FCCP | 01:24:02 | 121.99 |
| 6 | 74 | G04 | 25 µM FCCP | 01:24:16 | 120.26 |
| 6 | 75 | G04 | 25 µM FCCP | 01:24:30 | 118.53 |
| 6 | 76 | G04 | 25 µM FCCP | 01:24:44 | 117.06 |
| 6 | 77 | G04 | 25 µM FCCP | 01:24:59 | 115.57 |
| 6 | 78 | G04 | 25 µM FCCP | 01:25:13 | 114.25 |
| 6 | 79 | G04 | 25 µM FCCP | 01:25:27 | 112.88 |
| 6 | 80 | G04 | 25 µM FCCP | 01:25:41 | 111.60 |
| 6 | 81 | G04 | 25 µM FCCP | 01:25:55 | 110.38 |
| 6 | 82 | G04 | 25 µM FCCP | 01:26:10 | 109.16 |
| 6 | 83 | G04 | 25 µM FCCP | 01:26:24 | 108.02 |
| 7 | 84 | G04 | 25 µM FCCP | 01:31:58 | 131.79 |
| 7 | 85 | G04 | 25 µM FCCP | 01:32:12 | 127.54 |
| 7 | 86 | G04 | 25 µM FCCP | 01:32:26 | 125.08 |
| 7 | 87 | G04 | 25 µM FCCP | 01:32:40 | 123.17 |
| 7 | 88 | G04 | 25 µM FCCP | 01:32:54 | 121.34 |
| 7 | 89 | G04 | 25 µM FCCP | 01:33:08 | 119.75 |
| 7 | 90 | G04 | 25 µM FCCP | 01:33:23 | 118.23 |
| 7 | 91 | G04 | 25 µM FCCP | 01:33:37 | 116.83 |
| 7 | 92 | G04 | 25 µM FCCP | 01:33:51 | 115.44 |
| 7 | 93 | G04 | 25 µM FCCP | 01:34:05 | 114.22 |
| 7 | 94 | G04 | 25 µM FCCP | 01:34:19 | 112.93 |
| 7 | 95 | G04 | 25 µM FCCP | 01:34:33 | 111.82 |
| 7 | 96 | G04 | 25 µM FCCP | 01:34:48 | 110.64 |
| 7 | 97 | G04 | 25 µM FCCP | 01:35:02 | 109.59 |
| 8 | 98 | G04 | 25 µM FCCP | 01:40:36 | 132.70 |
| 8 | 99 | G04 | 25 µM FCCP | 01:40:50 | 128.61 |
| 8 | 100 | G04 | 25 µM FCCP | 01:41:04 | 126.13 |
| 8 | 101 | G04 | 25 µM FCCP | 01:41:18 | 124.23 |
| 8 | 102 | G04 | 25 µM FCCP | 01:41:32 | 122.60 |
| 8 | 103 | G04 | 25 µM FCCP | 01:41:46 | 121.01 |
| 8 | 104 | G04 | 25 µM FCCP | 01:42:00 | 119.58 |
| 8 | 105 | G04 | 25 µM FCCP | 01:42:15 | 118.20 |
| 8 | 106 | G04 | 25 µM FCCP | 01:42:29 | 116.89 |
| 8 | 107 | G04 | 25 µM FCCP | 01:42:43 | 115.65 |
| 8 | 108 | G04 | 25 µM FCCP | 01:42:57 | 114.45 |
| 8 | 109 | G04 | 25 µM FCCP | 01:43:11 | 113.34 |
| 8 | 110 | G04 | 25 µM FCCP | 01:43:26 | 112.22 |
| 8 | 111 | G04 | 25 µM FCCP | 01:43:40 | 111.23 |
| 9 | 112 | G04 | 25 µM FCCP | 01:49:14 | 133.77 |
| 9 | 113 | G04 | 25 µM FCCP | 01:49:28 | 129.70 |
| 9 | 114 | G04 | 25 µM FCCP | 01:49:42 | 127.41 |
| 9 | 115 | G04 | 25 µM FCCP | 01:49:56 | 125.65 |
| 9 | 116 | G04 | 25 µM FCCP | 01:50:10 | 123.82 |
| 9 | 117 | G04 | 25 µM FCCP | 01:50:24 | 122.45 |
| 9 | 118 | G04 | 25 µM FCCP | 01:50:38 | 121.03 |
| 9 | 119 | G04 | 25 µM FCCP | 01:50:53 | 119.74 |
| 9 | 120 | G04 | 25 µM FCCP | 01:51:07 | 118.47 |
| 9 | 121 | G04 | 25 µM FCCP | 01:51:21 | 117.29 |
| 9 | 122 | G04 | 25 µM FCCP | 01:51:35 | 116.24 |
| 9 | 123 | G04 | 25 µM FCCP | 01:51:50 | 115.13 |
| 9 | 124 | G04 | 25 µM FCCP | 01:52:04 | 114.10 |
| 9 | 125 | G04 | 25 µM FCCP | 01:52:18 | 113.08 |
| 10 | 126 | G04 | 25 µM FCCP | 01:57:52 | 134.60 |
| 10 | 127 | G04 | 25 µM FCCP | 01:58:06 | 130.97 |
| 10 | 128 | G04 | 25 µM FCCP | 01:58:20 | 128.68 |
| 10 | 129 | G04 | 25 µM FCCP | 01:58:34 | 127.00 |
| 10 | 130 | G04 | 25 µM FCCP | 01:58:48 | 125.42 |
| 10 | 131 | G04 | 25 µM FCCP | 01:59:03 | 123.92 |
| 10 | 132 | G04 | 25 µM FCCP | 01:59:17 | 122.58 |
| 10 | 133 | G04 | 25 µM FCCP | 01:59:31 | 121.26 |
| 10 | 134 | G04 | 25 µM FCCP | 01:59:45 | 120.23 |
| 10 | 135 | G04 | 25 µM FCCP | 01:59:59 | 119.00 |
| 10 | 136 | G04 | 25 µM FCCP | 02:00:14 | 117.92 |
| 10 | 137 | G04 | 25 µM FCCP | 02:00:28 | 116.99 |
| 10 | 138 | G04 | 25 µM FCCP | 02:00:42 | 115.94 |
| 10 | 139 | G04 | 25 µM FCCP | 02:00:56 | 115.08 |
| 11 | 140 | G04 | 25 µM FCCP | 02:06:30 | 136.83 |
| 11 | 141 | G04 | 25 µM FCCP | 02:06:44 | 133.52 |
| 11 | 142 | G04 | 25 µM FCCP | 02:06:58 | 131.36 |
| 11 | 143 | G04 | 25 µM FCCP | 02:07:12 | 129.60 |
| 11 | 144 | G04 | 25 µM FCCP | 02:07:26 | 128.08 |
| 11 | 145 | G04 | 25 µM FCCP | 02:07:41 | 126.64 |
| 11 | 146 | G04 | 25 µM FCCP | 02:07:55 | 125.42 |
| 11 | 147 | G04 | 25 µM FCCP | 02:08:09 | 124.09 |
| 11 | 148 | G04 | 25 µM FCCP | 02:08:23 | 122.88 |
| 11 | 149 | G04 | 25 µM FCCP | 02:08:37 | 121.75 |
| 11 | 150 | G04 | 25 µM FCCP | 02:08:52 | 120.80 |
| 11 | 151 | G04 | 25 µM FCCP | 02:09:06 | 119.65 |
| 11 | 152 | G04 | 25 µM FCCP | 02:09:20 | 118.81 |
| 11 | 153 | G04 | 25 µM FCCP | 02:09:34 | 117.88 |
| 12 | 154 | G04 | 25 µM FCCP | 02:15:08 | 137.74 |
| 12 | 155 | G04 | 25 µM FCCP | 02:15:22 | 134.71 |
| 12 | 156 | G04 | 25 µM FCCP | 02:15:37 | 132.52 |
| 12 | 157 | G04 | 25 µM FCCP | 02:15:53 | 130.78 |
| 12 | 158 | G04 | 25 µM FCCP | 02:16:08 | 129.25 |
| 12 | 159 | G04 | 25 µM FCCP | 02:16:22 | 127.90 |
| 12 | 160 | G04 | 25 µM FCCP | 02:16:36 | 126.73 |
| 12 | 161 | G04 | 25 µM FCCP | 02:16:50 | 125.54 |
| 12 | 162 | G04 | 25 µM FCCP | 02:17:04 | 124.41 |
| 12 | 163 | G04 | 25 µM FCCP | 02:17:18 | 123.39 |
| 12 | 164 | G04 | 25 µM FCCP | 02:17:33 | 122.36 |
| 12 | 165 | G04 | 25 µM FCCP | 02:17:47 | 121.47 |
| 12 | 166 | G04 | 25 µM FCCP | 02:18:01 | 120.44 |
| 13 | 167 | G04 | 25 µM FCCP | 02:23:39 | 137.65 |
| 13 | 168 | G04 | 25 µM FCCP | 02:23:54 | 135.05 |
| 13 | 169 | G04 | 25 µM FCCP | 02:24:08 | 133.31 |
| 13 | 170 | G04 | 25 µM FCCP | 02:24:22 | 131.82 |
| 13 | 171 | G04 | 25 µM FCCP | 02:24:36 | 130.47 |
| 13 | 172 | G04 | 25 µM FCCP | 02:24:51 | 128.95 |
| 13 | 173 | G04 | 25 µM FCCP | 02:25:07 | 127.77 |
| 13 | 174 | G04 | 25 µM FCCP | 02:25:22 | 126.60 |
| 13 | 175 | G04 | 25 µM FCCP | 02:25:36 | 125.59 |
| 13 | 176 | G04 | 25 µM FCCP | 02:25:50 | 124.64 |
| 13 | 177 | G04 | 25 µM FCCP | 02:26:04 | 123.73 |
| 13 | 178 | G04 | 25 µM FCCP | 02:26:18 | 122.92 |
| 13 | 179 | G04 | 25 µM FCCP | 02:26:33 | 122.01 |
| 14 | 180 | G04 | 25 µM FCCP | 02:32:07 | 139.18 |
| 14 | 181 | G04 | 25 µM FCCP | 02:32:21 | 136.44 |
| 14 | 182 | G04 | 25 µM FCCP | 02:32:35 | 134.67 |
| 14 | 183 | G04 | 25 µM FCCP | 02:32:50 | 133.22 |
| 14 | 184 | G04 | 25 µM FCCP | 02:33:04 | 131.85 |
| 14 | 185 | G04 | 25 µM FCCP | 02:33:18 | 130.65 |
| 14 | 186 | G04 | 25 µM FCCP | 02:33:32 | 129.45 |
| 14 | 187 | G04 | 25 µM FCCP | 02:33:46 | 128.40 |
| 14 | 188 | G04 | 25 µM FCCP | 02:34:01 | 127.49 |
| 14 | 189 | G04 | 25 µM FCCP | 02:34:15 | 126.48 |
| 14 | 190 | G04 | 25 µM FCCP | 02:34:29 | 125.61 |
| 14 | 191 | G04 | 25 µM FCCP | 02:34:43 | 124.72 |
| 14 | 192 | G04 | 25 µM FCCP | 02:34:57 | 123.92 |
| 14 | 193 | G04 | 25 µM FCCP | 02:35:12 | 123.11 |
| 15 | 194 | G04 | 25 µM FCCP | 02:40:46 | 139.83 |
| 15 | 195 | G04 | 25 µM FCCP | 02:41:00 | 137.24 |
| 15 | 196 | G04 | 25 µM FCCP | 02:41:14 | 135.48 |
| 15 | 197 | G04 | 25 µM FCCP | 02:41:28 | 134.06 |
| 15 | 198 | G04 | 25 µM FCCP | 02:41:43 | 132.72 |
| 15 | 199 | G04 | 25 µM FCCP | 02:41:57 | 131.63 |
| 15 | 200 | G04 | 25 µM FCCP | 02:42:11 | 130.59 |
| 15 | 201 | G04 | 25 µM FCCP | 02:42:25 | 129.54 |
| 15 | 202 | G04 | 25 µM FCCP | 02:42:39 | 128.63 |
| 15 | 203 | G04 | 25 µM FCCP | 02:42:53 | 127.77 |
| 15 | 204 | G04 | 25 µM FCCP | 02:43:08 | 126.94 |
| 15 | 205 | G04 | 25 µM FCCP | 02:43:22 | 126.13 |
| 15 | 206 | G04 | 25 µM FCCP | 02:43:36 | 125.23 |
| 15 | 207 | G04 | 25 µM FCCP | 02:43:50 | 124.54 |
| 16 | 208 | G04 | 25 µM FCCP | 02:49:25 | 140.37 |
| 16 | 209 | G04 | 25 µM FCCP | 02:49:40 | 137.83 |
| 16 | 210 | G04 | 25 µM FCCP | 02:49:54 | 136.21 |
| 16 | 211 | G04 | 25 µM FCCP | 02:50:08 | 134.88 |
| 16 | 212 | G04 | 25 µM FCCP | 02:50:22 | 133.63 |
| 16 | 213 | G04 | 25 µM FCCP | 02:50:36 | 132.55 |
| 16 | 214 | G04 | 25 µM FCCP | 02:50:50 | 131.52 |
| 16 | 215 | G04 | 25 µM FCCP | 02:51:05 | 130.64 |
| 16 | 216 | G04 | 25 µM FCCP | 02:51:19 | 129.64 |
| 16 | 217 | G04 | 25 µM FCCP | 02:51:33 | 128.77 |
| 16 | 218 | G04 | 25 µM FCCP | 02:51:47 | 128.07 |
| 16 | 219 | G04 | 25 µM FCCP | 02:52:01 | 127.37 |
| 16 | 220 | G04 | 25 µM FCCP | 02:52:16 | 126.51 |
| 16 | 221 | G04 | 25 µM FCCP | 02:52:30 | 125.73 |
| 1 | 0 | G05 | 25 µM FCCP | 00:40:06 | 128.40 |
| 1 | 1 | G05 | 25 µM FCCP | 00:40:20 | 122.92 |
| 1 | 2 | G05 | 25 µM FCCP | 00:40:34 | 120.35 |
| 1 | 3 | G05 | 25 µM FCCP | 00:40:48 | 118.40 |
| 1 | 4 | G05 | 25 µM FCCP | 00:41:02 | 116.69 |
| 1 | 5 | G05 | 25 µM FCCP | 00:41:17 | 115.12 |
| 1 | 6 | G05 | 25 µM FCCP | 00:41:31 | 113.74 |
| 1 | 7 | G05 | 25 µM FCCP | 00:41:45 | 112.41 |
| 1 | 8 | G05 | 25 µM FCCP | 00:41:59 | 111.20 |
| 1 | 9 | G05 | 25 µM FCCP | 00:42:13 | 110.00 |
| 1 | 10 | G05 | 25 µM FCCP | 00:42:28 | 108.73 |
| 1 | 11 | G05 | 25 µM FCCP | 00:42:42 | 107.62 |
| 1 | 12 | G05 | 25 µM FCCP | 00:42:56 | 106.57 |
| 1 | 13 | G05 | 25 µM FCCP | 00:43:10 | 105.57 |
| 2 | 14 | G05 | 25 µM FCCP | 00:48:43 | 132.11 |
| 2 | 15 | G05 | 25 µM FCCP | 00:48:57 | 127.56 |
| 2 | 16 | G05 | 25 µM FCCP | 00:49:12 | 125.23 |
| 2 | 17 | G05 | 25 µM FCCP | 00:49:26 | 123.06 |
| 2 | 18 | G05 | 25 µM FCCP | 00:49:40 | 121.27 |
| 2 | 19 | G05 | 25 µM FCCP | 00:49:54 | 119.65 |
| 2 | 20 | G05 | 25 µM FCCP | 00:50:08 | 118.12 |
| 2 | 21 | G05 | 25 µM FCCP | 00:50:22 | 116.57 |
| 2 | 22 | G05 | 25 µM FCCP | 00:50:37 | 115.28 |
| 2 | 23 | G05 | 25 µM FCCP | 00:50:51 | 114.06 |
| 2 | 24 | G05 | 25 µM FCCP | 00:51:05 | 112.77 |
| 2 | 25 | G05 | 25 µM FCCP | 00:51:19 | 111.58 |
| 2 | 26 | G05 | 25 µM FCCP | 00:51:33 | 110.33 |
| 2 | 27 | G05 | 25 µM FCCP | 00:51:48 | 109.25 |
| 3 | 28 | G05 | 25 µM FCCP | 00:57:21 | 133.25 |
| 3 | 29 | G05 | 25 µM FCCP | 00:57:35 | 129.19 |
| 3 | 30 | G05 | 25 µM FCCP | 00:57:49 | 126.73 |
| 3 | 31 | G05 | 25 µM FCCP | 00:58:03 | 124.81 |
| 3 | 32 | G05 | 25 µM FCCP | 00:58:17 | 123.04 |
| 3 | 33 | G05 | 25 µM FCCP | 00:58:31 | 121.37 |
| 3 | 34 | G05 | 25 µM FCCP | 00:58:46 | 119.93 |
| 3 | 35 | G05 | 25 µM FCCP | 00:59:00 | 118.44 |
| 3 | 36 | G05 | 25 µM FCCP | 00:59:14 | 117.03 |
| 3 | 37 | G05 | 25 µM FCCP | 00:59:28 | 115.79 |
| 3 | 38 | G05 | 25 µM FCCP | 00:59:42 | 114.55 |
| 3 | 39 | G05 | 25 µM FCCP | 00:59:56 | 113.35 |
| 3 | 40 | G05 | 25 µM FCCP | 01:00:11 | 112.13 |
| 3 | 41 | G05 | 25 µM FCCP | 01:00:25 | 111.06 |
| 4 | 42 | G05 | 25 µM FCCP | 01:06:04 | 134.58 |
| 4 | 43 | G05 | 25 µM FCCP | 01:06:18 | 130.19 |
| 4 | 44 | G05 | 25 µM FCCP | 01:06:33 | 127.73 |
| 4 | 45 | G05 | 25 µM FCCP | 01:06:47 | 125.75 |
| 4 | 46 | G05 | 25 µM FCCP | 01:07:01 | 124.06 |
| 4 | 47 | G05 | 25 µM FCCP | 01:07:15 | 122.46 |
| 4 | 48 | G05 | 25 µM FCCP | 01:07:29 | 120.89 |
| 4 | 49 | G05 | 25 µM FCCP | 01:07:43 | 119.46 |
| 4 | 50 | G05 | 25 µM FCCP | 01:07:58 | 118.07 |
| 4 | 51 | G05 | 25 µM FCCP | 01:08:12 | 116.77 |
| 4 | 52 | G05 | 25 µM FCCP | 01:08:26 | 115.36 |
| 4 | 53 | G05 | 25 µM FCCP | 01:08:40 | 114.07 |
| 4 | 54 | G05 | 25 µM FCCP | 01:08:54 | 112.87 |
| 4 | 55 | G05 | 25 µM FCCP | 01:09:09 | 111.66 |
| 5 | 56 | G05 | 25 µM FCCP | 01:14:42 | 133.25 |
| 5 | 57 | G05 | 25 µM FCCP | 01:14:56 | 128.49 |
| 5 | 58 | G05 | 25 µM FCCP | 01:15:10 | 125.88 |
| 5 | 59 | G05 | 25 µM FCCP | 01:15:25 | 123.78 |
| 5 | 60 | G05 | 25 µM FCCP | 01:15:39 | 122.04 |
| 5 | 61 | G05 | 25 µM FCCP | 01:15:53 | 120.31 |
| 5 | 62 | G05 | 25 µM FCCP | 01:16:07 | 118.80 |
| 5 | 63 | G05 | 25 µM FCCP | 01:16:21 | 117.36 |
| 5 | 64 | G05 | 25 µM FCCP | 01:16:35 | 116.00 |
| 5 | 65 | G05 | 25 µM FCCP | 01:16:50 | 114.72 |
| 5 | 66 | G05 | 25 µM FCCP | 01:17:04 | 113.44 |
| 5 | 67 | G05 | 25 µM FCCP | 01:17:18 | 112.15 |
| 5 | 68 | G05 | 25 µM FCCP | 01:17:32 | 110.95 |
| 5 | 69 | G05 | 25 µM FCCP | 01:17:46 | 109.78 |
| 6 | 70 | G05 | 25 µM FCCP | 01:23:20 | 133.39 |
| 6 | 71 | G05 | 25 µM FCCP | 01:23:34 | 128.54 |
| 6 | 72 | G05 | 25 µM FCCP | 01:23:48 | 126.09 |
| 6 | 73 | G05 | 25 µM FCCP | 01:24:02 | 124.02 |
| 6 | 74 | G05 | 25 µM FCCP | 01:24:16 | 122.26 |
| 6 | 75 | G05 | 25 µM FCCP | 01:24:30 | 120.57 |
| 6 | 76 | G05 | 25 µM FCCP | 01:24:44 | 119.11 |
| 6 | 77 | G05 | 25 µM FCCP | 01:24:59 | 117.71 |
| 6 | 78 | G05 | 25 µM FCCP | 01:25:13 | 116.34 |
| 6 | 79 | G05 | 25 µM FCCP | 01:25:27 | 115.05 |
| 6 | 80 | G05 | 25 µM FCCP | 01:25:41 | 113.77 |
| 6 | 81 | G05 | 25 µM FCCP | 01:25:55 | 112.60 |
| 6 | 82 | G05 | 25 µM FCCP | 01:26:10 | 111.40 |
| 6 | 83 | G05 | 25 µM FCCP | 01:26:24 | 110.19 |
| 7 | 84 | G05 | 25 µM FCCP | 01:31:58 | 133.74 |
| 7 | 85 | G05 | 25 µM FCCP | 01:32:12 | 129.19 |
| 7 | 86 | G05 | 25 µM FCCP | 01:32:26 | 126.65 |
| 7 | 87 | G05 | 25 µM FCCP | 01:32:40 | 124.79 |
| 7 | 88 | G05 | 25 µM FCCP | 01:32:54 | 122.94 |
| 7 | 89 | G05 | 25 µM FCCP | 01:33:08 | 121.40 |
| 7 | 90 | G05 | 25 µM FCCP | 01:33:23 | 119.95 |
| 7 | 91 | G05 | 25 µM FCCP | 01:33:37 | 118.52 |
| 7 | 92 | G05 | 25 µM FCCP | 01:33:51 | 117.22 |
| 7 | 93 | G05 | 25 µM FCCP | 01:34:05 | 116.10 |
| 7 | 94 | G05 | 25 µM FCCP | 01:34:19 | 114.79 |
| 7 | 95 | G05 | 25 µM FCCP | 01:34:33 | 113.57 |
| 7 | 96 | G05 | 25 µM FCCP | 01:34:48 | 112.52 |
| 7 | 97 | G05 | 25 µM FCCP | 01:35:02 | 111.42 |
| 8 | 98 | G05 | 25 µM FCCP | 01:40:36 | 134.64 |
| 8 | 99 | G05 | 25 µM FCCP | 01:40:50 | 130.15 |
| 8 | 100 | G05 | 25 µM FCCP | 01:41:04 | 127.75 |
| 8 | 101 | G05 | 25 µM FCCP | 01:41:18 | 125.80 |
| 8 | 102 | G05 | 25 µM FCCP | 01:41:32 | 124.10 |
| 8 | 103 | G05 | 25 µM FCCP | 01:41:46 | 122.65 |
| 8 | 104 | G05 | 25 µM FCCP | 01:42:00 | 121.27 |
| 8 | 105 | G05 | 25 µM FCCP | 01:42:15 | 119.87 |
| 8 | 106 | G05 | 25 µM FCCP | 01:42:29 | 118.63 |
| 8 | 107 | G05 | 25 µM FCCP | 01:42:43 | 117.40 |
| 8 | 108 | G05 | 25 µM FCCP | 01:42:57 | 116.25 |
| 8 | 109 | G05 | 25 µM FCCP | 01:43:11 | 115.12 |
| 8 | 110 | G05 | 25 µM FCCP | 01:43:26 | 114.06 |
| 8 | 111 | G05 | 25 µM FCCP | 01:43:40 | 113.05 |
| 9 | 112 | G05 | 25 µM FCCP | 01:49:14 | 135.46 |
| 9 | 113 | G05 | 25 µM FCCP | 01:49:28 | 131.27 |
| 9 | 114 | G05 | 25 µM FCCP | 01:49:42 | 128.84 |
| 9 | 115 | G05 | 25 µM FCCP | 01:49:56 | 126.99 |
| 9 | 116 | G05 | 25 µM FCCP | 01:50:10 | 125.39 |
| 9 | 117 | G05 | 25 µM FCCP | 01:50:24 | 124.01 |
| 9 | 118 | G05 | 25 µM FCCP | 01:50:38 | 122.66 |
| 9 | 119 | G05 | 25 µM FCCP | 01:50:53 | 121.21 |
| 9 | 120 | G05 | 25 µM FCCP | 01:51:07 | 120.07 |
| 9 | 121 | G05 | 25 µM FCCP | 01:51:21 | 118.93 |
| 9 | 122 | G05 | 25 µM FCCP | 01:51:35 | 117.78 |
| 9 | 123 | G05 | 25 µM FCCP | 01:51:50 | 116.78 |
| 9 | 124 | G05 | 25 µM FCCP | 01:52:04 | 115.76 |
| 9 | 125 | G05 | 25 µM FCCP | 01:52:18 | 114.76 |
| 10 | 126 | G05 | 25 µM FCCP | 01:57:52 | 136.02 |
| 10 | 127 | G05 | 25 µM FCCP | 01:58:06 | 132.07 |
| 10 | 128 | G05 | 25 µM FCCP | 01:58:20 | 129.83 |
| 10 | 129 | G05 | 25 µM FCCP | 01:58:34 | 128.23 |
| 10 | 130 | G05 | 25 µM FCCP | 01:58:48 | 126.62 |
| 10 | 131 | G05 | 25 µM FCCP | 01:59:03 | 125.20 |
| 10 | 132 | G05 | 25 µM FCCP | 01:59:17 | 123.90 |
| 10 | 133 | G05 | 25 µM FCCP | 01:59:31 | 122.64 |
| 10 | 134 | G05 | 25 µM FCCP | 01:59:45 | 121.64 |
| 10 | 135 | G05 | 25 µM FCCP | 01:59:59 | 120.49 |
| 10 | 136 | G05 | 25 µM FCCP | 02:00:14 | 119.45 |
| 10 | 137 | G05 | 25 µM FCCP | 02:00:28 | 118.46 |
| 10 | 138 | G05 | 25 µM FCCP | 02:00:42 | 117.54 |
| 10 | 139 | G05 | 25 µM FCCP | 02:00:56 | 116.65 |
| 11 | 140 | G05 | 25 µM FCCP | 02:06:30 | 137.42 |
| 11 | 141 | G05 | 25 µM FCCP | 02:06:44 | 134.06 |
| 11 | 142 | G05 | 25 µM FCCP | 02:06:58 | 132.04 |
| 11 | 143 | G05 | 25 µM FCCP | 02:07:12 | 130.42 |
| 11 | 144 | G05 | 25 µM FCCP | 02:07:26 | 128.96 |
| 11 | 145 | G05 | 25 µM FCCP | 02:07:41 | 127.60 |
| 11 | 146 | G05 | 25 µM FCCP | 02:07:55 | 126.39 |
| 11 | 147 | G05 | 25 µM FCCP | 02:08:09 | 125.22 |
| 11 | 148 | G05 | 25 µM FCCP | 02:08:23 | 123.98 |
| 11 | 149 | G05 | 25 µM FCCP | 02:08:37 | 122.99 |
| 11 | 150 | G05 | 25 µM FCCP | 02:08:52 | 121.98 |
| 11 | 151 | G05 | 25 µM FCCP | 02:09:06 | 120.96 |
| 11 | 152 | G05 | 25 µM FCCP | 02:09:20 | 120.08 |
| 11 | 153 | G05 | 25 µM FCCP | 02:09:34 | 119.14 |
| 12 | 154 | G05 | 25 µM FCCP | 02:15:08 | 138.28 |
| 12 | 155 | G05 | 25 µM FCCP | 02:15:22 | 135.21 |
| 12 | 156 | G05 | 25 µM FCCP | 02:15:37 | 133.07 |
| 12 | 157 | G05 | 25 µM FCCP | 02:15:53 | 131.41 |
| 12 | 158 | G05 | 25 µM FCCP | 02:16:08 | 129.96 |
| 12 | 159 | G05 | 25 µM FCCP | 02:16:22 | 128.82 |
| 12 | 160 | G05 | 25 µM FCCP | 02:16:36 | 127.64 |
| 12 | 161 | G05 | 25 µM FCCP | 02:16:50 | 126.57 |
| 12 | 162 | G05 | 25 µM FCCP | 02:17:04 | 125.50 |
| 12 | 163 | G05 | 25 µM FCCP | 02:17:18 | 124.53 |
| 12 | 164 | G05 | 25 µM FCCP | 02:17:33 | 123.52 |
| 12 | 165 | G05 | 25 µM FCCP | 02:17:47 | 122.57 |
| 12 | 166 | G05 | 25 µM FCCP | 02:18:01 | 121.70 |
| 13 | 167 | G05 | 25 µM FCCP | 02:23:39 | 138.09 |
| 13 | 168 | G05 | 25 µM FCCP | 02:23:54 | 135.49 |
| 13 | 169 | G05 | 25 µM FCCP | 02:24:08 | 133.85 |
| 13 | 170 | G05 | 25 µM FCCP | 02:24:22 | 132.40 |
| 13 | 171 | G05 | 25 µM FCCP | 02:24:36 | 131.11 |
| 13 | 172 | G05 | 25 µM FCCP | 02:24:51 | 129.73 |
| 13 | 173 | G05 | 25 µM FCCP | 02:25:07 | 128.60 |
| 13 | 174 | G05 | 25 µM FCCP | 02:25:22 | 127.49 |
| 13 | 175 | G05 | 25 µM FCCP | 02:25:36 | 126.58 |
| 13 | 176 | G05 | 25 µM FCCP | 02:25:50 | 125.66 |
| 13 | 177 | G05 | 25 µM FCCP | 02:26:04 | 124.80 |
| 13 | 178 | G05 | 25 µM FCCP | 02:26:18 | 123.91 |
| 13 | 179 | G05 | 25 µM FCCP | 02:26:33 | 123.05 |
| 14 | 180 | G05 | 25 µM FCCP | 02:32:07 | 139.56 |
| 14 | 181 | G05 | 25 µM FCCP | 02:32:21 | 136.64 |
| 14 | 182 | G05 | 25 µM FCCP | 02:32:35 | 135.09 |
| 14 | 183 | G05 | 25 µM FCCP | 02:32:50 | 133.59 |
| 14 | 184 | G05 | 25 µM FCCP | 02:33:04 | 132.36 |
| 14 | 185 | G05 | 25 µM FCCP | 02:33:18 | 131.21 |
| 14 | 186 | G05 | 25 µM FCCP | 02:33:32 | 130.07 |
| 14 | 187 | G05 | 25 µM FCCP | 02:33:46 | 129.11 |
| 14 | 188 | G05 | 25 µM FCCP | 02:34:01 | 128.17 |
| 14 | 189 | G05 | 25 µM FCCP | 02:34:15 | 127.22 |
| 14 | 190 | G05 | 25 µM FCCP | 02:34:29 | 126.40 |
| 14 | 191 | G05 | 25 µM FCCP | 02:34:43 | 125.66 |
| 14 | 192 | G05 | 25 µM FCCP | 02:34:57 | 124.77 |
| 14 | 193 | G05 | 25 µM FCCP | 02:35:12 | 124.11 |
| 15 | 194 | G05 | 25 µM FCCP | 02:40:46 | 140.05 |
| 15 | 195 | G05 | 25 µM FCCP | 02:41:00 | 137.40 |
| 15 | 196 | G05 | 25 µM FCCP | 02:41:14 | 135.73 |
| 15 | 197 | G05 | 25 µM FCCP | 02:41:28 | 134.39 |
| 15 | 198 | G05 | 25 µM FCCP | 02:41:43 | 133.20 |
| 15 | 199 | G05 | 25 µM FCCP | 02:41:57 | 132.18 |
| 15 | 200 | G05 | 25 µM FCCP | 02:42:11 | 131.04 |
| 15 | 201 | G05 | 25 µM FCCP | 02:42:25 | 130.12 |
| 15 | 202 | G05 | 25 µM FCCP | 02:42:39 | 129.31 |
| 15 | 203 | G05 | 25 µM FCCP | 02:42:53 | 128.39 |
| 15 | 204 | G05 | 25 µM FCCP | 02:43:08 | 127.55 |
| 15 | 205 | G05 | 25 µM FCCP | 02:43:22 | 126.87 |
| 15 | 206 | G05 | 25 µM FCCP | 02:43:36 | 126.11 |
| 15 | 207 | G05 | 25 µM FCCP | 02:43:50 | 125.33 |
| 16 | 208 | G05 | 25 µM FCCP | 02:49:25 | 140.58 |
| 16 | 209 | G05 | 25 µM FCCP | 02:49:40 | 137.93 |
| 16 | 210 | G05 | 25 µM FCCP | 02:49:54 | 136.41 |
| 16 | 211 | G05 | 25 µM FCCP | 02:50:08 | 135.06 |
| 16 | 212 | G05 | 25 µM FCCP | 02:50:22 | 134.00 |
| 16 | 213 | G05 | 25 µM FCCP | 02:50:36 | 132.90 |
| 16 | 214 | G05 | 25 µM FCCP | 02:50:50 | 131.96 |
| 16 | 215 | G05 | 25 µM FCCP | 02:51:05 | 131.03 |
| 16 | 216 | G05 | 25 µM FCCP | 02:51:19 | 130.10 |
| 16 | 217 | G05 | 25 µM FCCP | 02:51:33 | 129.33 |
| 16 | 218 | G05 | 25 µM FCCP | 02:51:47 | 128.64 |
| 16 | 219 | G05 | 25 µM FCCP | 02:52:01 | 127.98 |
| 16 | 220 | G05 | 25 µM FCCP | 02:52:16 | 127.15 |
| 16 | 221 | G05 | 25 µM FCCP | 02:52:30 | 126.45 |
| 1 | 0 | G06 | 25 µM FCCP | 00:40:06 | 127.09 |
| 1 | 1 | G06 | 25 µM FCCP | 00:40:20 | 122.12 |
| 1 | 2 | G06 | 25 µM FCCP | 00:40:34 | 119.64 |
| 1 | 3 | G06 | 25 µM FCCP | 00:40:48 | 117.67 |
| 1 | 4 | G06 | 25 µM FCCP | 00:41:02 | 115.90 |
| 1 | 5 | G06 | 25 µM FCCP | 00:41:17 | 114.38 |
| 1 | 6 | G06 | 25 µM FCCP | 00:41:31 | 112.90 |
| 1 | 7 | G06 | 25 µM FCCP | 00:41:45 | 111.63 |
| 1 | 8 | G06 | 25 µM FCCP | 00:41:59 | 110.25 |
| 1 | 9 | G06 | 25 µM FCCP | 00:42:13 | 109.08 |
| 1 | 10 | G06 | 25 µM FCCP | 00:42:28 | 107.77 |
| 1 | 11 | G06 | 25 µM FCCP | 00:42:42 | 106.69 |
| 1 | 12 | G06 | 25 µM FCCP | 00:42:56 | 105.55 |
| 1 | 13 | G06 | 25 µM FCCP | 00:43:10 | 104.51 |
| 2 | 14 | G06 | 25 µM FCCP | 00:48:43 | 130.63 |
| 2 | 15 | G06 | 25 µM FCCP | 00:48:57 | 126.90 |
| 2 | 16 | G06 | 25 µM FCCP | 00:49:12 | 124.52 |
| 2 | 17 | G06 | 25 µM FCCP | 00:49:26 | 122.50 |
| 2 | 18 | G06 | 25 µM FCCP | 00:49:40 | 120.74 |
| 2 | 19 | G06 | 25 µM FCCP | 00:49:54 | 119.09 |
| 2 | 20 | G06 | 25 µM FCCP | 00:50:08 | 117.54 |
| 2 | 21 | G06 | 25 µM FCCP | 00:50:22 | 116.05 |
| 2 | 22 | G06 | 25 µM FCCP | 00:50:37 | 114.72 |
| 2 | 23 | G06 | 25 µM FCCP | 00:50:51 | 113.44 |
| 2 | 24 | G06 | 25 µM FCCP | 00:51:05 | 112.13 |
| 2 | 25 | G06 | 25 µM FCCP | 00:51:19 | 110.94 |
| 2 | 26 | G06 | 25 µM FCCP | 00:51:33 | 109.70 |
| 2 | 27 | G06 | 25 µM FCCP | 00:51:48 | 108.64 |
| 3 | 28 | G06 | 25 µM FCCP | 00:57:21 | 132.06 |
| 3 | 29 | G06 | 25 µM FCCP | 00:57:35 | 128.52 |
| 3 | 30 | G06 | 25 µM FCCP | 00:57:49 | 126.16 |
| 3 | 31 | G06 | 25 µM FCCP | 00:58:03 | 124.16 |
| 3 | 32 | G06 | 25 µM FCCP | 00:58:17 | 122.52 |
| 3 | 33 | G06 | 25 µM FCCP | 00:58:31 | 120.79 |
| 3 | 34 | G06 | 25 µM FCCP | 00:58:46 | 119.27 |
| 3 | 35 | G06 | 25 µM FCCP | 00:59:00 | 117.82 |
| 3 | 36 | G06 | 25 µM FCCP | 00:59:14 | 116.41 |
| 3 | 37 | G06 | 25 µM FCCP | 00:59:28 | 115.18 |
| 3 | 38 | G06 | 25 µM FCCP | 00:59:42 | 113.81 |
| 3 | 39 | G06 | 25 µM FCCP | 00:59:56 | 112.69 |
| 3 | 40 | G06 | 25 µM FCCP | 01:00:11 | 111.53 |
| 3 | 41 | G06 | 25 µM FCCP | 01:00:25 | 110.35 |
| 4 | 42 | G06 | 25 µM FCCP | 01:06:04 | 132.92 |
| 4 | 43 | G06 | 25 µM FCCP | 01:06:18 | 128.95 |
| 4 | 44 | G06 | 25 µM FCCP | 01:06:33 | 126.44 |
| 4 | 45 | G06 | 25 µM FCCP | 01:06:47 | 124.41 |
| 4 | 46 | G06 | 25 µM FCCP | 01:07:01 | 122.64 |
| 4 | 47 | G06 | 25 µM FCCP | 01:07:15 | 120.92 |
| 4 | 48 | G06 | 25 µM FCCP | 01:07:29 | 119.27 |
| 4 | 49 | G06 | 25 µM FCCP | 01:07:43 | 117.69 |
| 4 | 50 | G06 | 25 µM FCCP | 01:07:58 | 116.29 |
| 4 | 51 | G06 | 25 µM FCCP | 01:08:12 | 114.89 |
| 4 | 52 | G06 | 25 µM FCCP | 01:08:26 | 113.47 |
| 4 | 53 | G06 | 25 µM FCCP | 01:08:40 | 112.12 |
| 4 | 54 | G06 | 25 µM FCCP | 01:08:54 | 110.86 |
| 4 | 55 | G06 | 25 µM FCCP | 01:09:09 | 109.46 |
| 5 | 56 | G06 | 25 µM FCCP | 01:14:42 | 131.25 |
| 5 | 57 | G06 | 25 µM FCCP | 01:14:56 | 126.74 |
| 5 | 58 | G06 | 25 µM FCCP | 01:15:10 | 123.90 |
| 5 | 59 | G06 | 25 µM FCCP | 01:15:25 | 121.79 |
| 5 | 60 | G06 | 25 µM FCCP | 01:15:39 | 119.89 |
| 5 | 61 | G06 | 25 µM FCCP | 01:15:53 | 118.07 |
| 5 | 62 | G06 | 25 µM FCCP | 01:16:07 | 116.45 |
| 5 | 63 | G06 | 25 µM FCCP | 01:16:21 | 114.86 |
| 5 | 64 | G06 | 25 µM FCCP | 01:16:35 | 113.43 |
| 5 | 65 | G06 | 25 µM FCCP | 01:16:50 | 112.01 |
| 5 | 66 | G06 | 25 µM FCCP | 01:17:04 | 110.63 |
| 5 | 67 | G06 | 25 µM FCCP | 01:17:18 | 109.27 |
| 5 | 68 | G06 | 25 µM FCCP | 01:17:32 | 107.98 |
| 5 | 69 | G06 | 25 µM FCCP | 01:17:46 | 106.75 |
| 6 | 70 | G06 | 25 µM FCCP | 01:23:20 | 131.05 |
| 6 | 71 | G06 | 25 µM FCCP | 01:23:34 | 126.36 |
| 6 | 72 | G06 | 25 µM FCCP | 01:23:48 | 123.75 |
| 6 | 73 | G06 | 25 µM FCCP | 01:24:02 | 121.54 |
| 6 | 74 | G06 | 25 µM FCCP | 01:24:16 | 119.72 |
| 6 | 75 | G06 | 25 µM FCCP | 01:24:30 | 117.92 |
| 6 | 76 | G06 | 25 µM FCCP | 01:24:44 | 116.31 |
| 6 | 77 | G06 | 25 µM FCCP | 01:24:59 | 114.80 |
| 6 | 78 | G06 | 25 µM FCCP | 01:25:13 | 113.31 |
| 6 | 79 | G06 | 25 µM FCCP | 01:25:27 | 111.93 |
| 6 | 80 | G06 | 25 µM FCCP | 01:25:41 | 110.54 |
| 6 | 81 | G06 | 25 µM FCCP | 01:25:55 | 109.29 |
| 6 | 82 | G06 | 25 µM FCCP | 01:26:10 | 107.98 |
| 6 | 83 | G06 | 25 µM FCCP | 01:26:24 | 106.79 |
| 7 | 84 | G06 | 25 µM FCCP | 01:31:58 | 131.48 |
| 7 | 85 | G06 | 25 µM FCCP | 01:32:12 | 126.96 |
| 7 | 86 | G06 | 25 µM FCCP | 01:32:26 | 124.38 |
| 7 | 87 | G06 | 25 µM FCCP | 01:32:40 | 122.34 |
| 7 | 88 | G06 | 25 µM FCCP | 01:32:54 | 120.42 |
| 7 | 89 | G06 | 25 µM FCCP | 01:33:08 | 118.77 |
| 7 | 90 | G06 | 25 µM FCCP | 01:33:23 | 117.10 |
| 7 | 91 | G06 | 25 µM FCCP | 01:33:37 | 115.68 |
| 7 | 92 | G06 | 25 µM FCCP | 01:33:51 | 114.18 |
| 7 | 93 | G06 | 25 µM FCCP | 01:34:05 | 112.91 |
| 7 | 94 | G06 | 25 µM FCCP | 01:34:19 | 111.54 |
| 7 | 95 | G06 | 25 µM FCCP | 01:34:33 | 110.35 |
| 7 | 96 | G06 | 25 µM FCCP | 01:34:48 | 109.15 |
| 7 | 97 | G06 | 25 µM FCCP | 01:35:02 | 108.02 |
| 8 | 98 | G06 | 25 µM FCCP | 01:40:36 | 132.11 |
| 8 | 99 | G06 | 25 µM FCCP | 01:40:50 | 127.78 |
| 8 | 100 | G06 | 25 µM FCCP | 01:41:04 | 125.36 |
| 8 | 101 | G06 | 25 µM FCCP | 01:41:18 | 123.32 |
| 8 | 102 | G06 | 25 µM FCCP | 01:41:32 | 121.46 |
| 8 | 103 | G06 | 25 µM FCCP | 01:41:46 | 119.78 |
| 8 | 104 | G06 | 25 µM FCCP | 01:42:00 | 118.38 |
| 8 | 105 | G06 | 25 µM FCCP | 01:42:15 | 116.97 |
| 8 | 106 | G06 | 25 µM FCCP | 01:42:29 | 115.59 |
| 8 | 107 | G06 | 25 µM FCCP | 01:42:43 | 114.23 |
| 8 | 108 | G06 | 25 µM FCCP | 01:42:57 | 112.96 |
| 8 | 109 | G06 | 25 µM FCCP | 01:43:11 | 111.84 |
| 8 | 110 | G06 | 25 µM FCCP | 01:43:26 | 110.58 |
| 8 | 111 | G06 | 25 µM FCCP | 01:43:40 | 109.52 |
| 9 | 112 | G06 | 25 µM FCCP | 01:49:14 | 132.92 |
| 9 | 113 | G06 | 25 µM FCCP | 01:49:28 | 128.80 |
| 9 | 114 | G06 | 25 µM FCCP | 01:49:42 | 126.36 |
| 9 | 115 | G06 | 25 µM FCCP | 01:49:56 | 124.35 |
| 9 | 116 | G06 | 25 µM FCCP | 01:50:10 | 122.62 |
| 9 | 117 | G06 | 25 µM FCCP | 01:50:24 | 121.05 |
| 9 | 118 | G06 | 25 µM FCCP | 01:50:38 | 119.67 |
| 9 | 119 | G06 | 25 µM FCCP | 01:50:53 | 118.28 |
| 9 | 120 | G06 | 25 µM FCCP | 01:51:07 | 116.97 |
| 9 | 121 | G06 | 25 µM FCCP | 01:51:21 | 115.74 |
| 9 | 122 | G06 | 25 µM FCCP | 01:51:35 | 114.60 |
| 9 | 123 | G06 | 25 µM FCCP | 01:51:50 | 113.43 |
| 9 | 124 | G06 | 25 µM FCCP | 01:52:04 | 112.42 |
| 9 | 125 | G06 | 25 µM FCCP | 01:52:18 | 111.30 |
| 10 | 126 | G06 | 25 µM FCCP | 01:57:52 | 133.38 |
| 10 | 127 | G06 | 25 µM FCCP | 01:58:06 | 129.64 |
| 10 | 128 | G06 | 25 µM FCCP | 01:58:20 | 127.32 |
| 10 | 129 | G06 | 25 µM FCCP | 01:58:34 | 125.54 |
| 10 | 130 | G06 | 25 µM FCCP | 01:58:48 | 123.91 |
| 10 | 131 | G06 | 25 µM FCCP | 01:59:03 | 122.37 |
| 10 | 132 | G06 | 25 µM FCCP | 01:59:17 | 120.96 |
| 10 | 133 | G06 | 25 µM FCCP | 01:59:31 | 119.58 |
| 10 | 134 | G06 | 25 µM FCCP | 01:59:45 | 118.53 |
| 10 | 135 | G06 | 25 µM FCCP | 01:59:59 | 117.27 |
| 10 | 136 | G06 | 25 µM FCCP | 02:00:14 | 116.15 |
| 10 | 137 | G06 | 25 µM FCCP | 02:00:28 | 115.12 |
| 10 | 138 | G06 | 25 µM FCCP | 02:00:42 | 114.05 |
| 10 | 139 | G06 | 25 µM FCCP | 02:00:56 | 113.15 |
| 11 | 140 | G06 | 25 µM FCCP | 02:06:30 | 134.92 |
| 11 | 141 | G06 | 25 µM FCCP | 02:06:44 | 131.77 |
| 11 | 142 | G06 | 25 µM FCCP | 02:06:58 | 129.59 |
| 11 | 143 | G06 | 25 µM FCCP | 02:07:12 | 127.84 |
| 11 | 144 | G06 | 25 µM FCCP | 02:07:26 | 126.36 |
| 11 | 145 | G06 | 25 µM FCCP | 02:07:41 | 124.85 |
| 11 | 146 | G06 | 25 µM FCCP | 02:07:55 | 123.53 |
| 11 | 147 | G06 | 25 µM FCCP | 02:08:09 | 122.25 |
| 11 | 148 | G06 | 25 µM FCCP | 02:08:23 | 121.03 |
| 11 | 149 | G06 | 25 µM FCCP | 02:08:37 | 119.85 |
| 11 | 150 | G06 | 25 µM FCCP | 02:08:52 | 118.81 |
| 11 | 151 | G06 | 25 µM FCCP | 02:09:06 | 117.77 |
| 11 | 152 | G06 | 25 µM FCCP | 02:09:20 | 116.86 |
| 11 | 153 | G06 | 25 µM FCCP | 02:09:34 | 115.89 |
| 12 | 154 | G06 | 25 µM FCCP | 02:15:08 | 135.77 |
| 12 | 155 | G06 | 25 µM FCCP | 02:15:22 | 132.89 |
| 12 | 156 | G06 | 25 µM FCCP | 02:15:37 | 130.79 |
| 12 | 157 | G06 | 25 µM FCCP | 02:15:53 | 128.90 |
| 12 | 158 | G06 | 25 µM FCCP | 02:16:08 | 127.45 |
| 12 | 159 | G06 | 25 µM FCCP | 02:16:22 | 126.09 |
| 12 | 160 | G06 | 25 µM FCCP | 02:16:36 | 124.90 |
| 12 | 161 | G06 | 25 µM FCCP | 02:16:50 | 123.80 |
| 12 | 162 | G06 | 25 µM FCCP | 02:17:04 | 122.57 |
| 12 | 163 | G06 | 25 µM FCCP | 02:17:18 | 121.43 |
| 12 | 164 | G06 | 25 µM FCCP | 02:17:33 | 120.44 |
| 12 | 165 | G06 | 25 µM FCCP | 02:17:47 | 119.44 |
| 12 | 166 | G06 | 25 µM FCCP | 02:18:01 | 118.55 |
| 13 | 167 | G06 | 25 µM FCCP | 02:23:39 | 135.80 |
| 13 | 168 | G06 | 25 µM FCCP | 02:23:54 | 133.29 |
| 13 | 169 | G06 | 25 µM FCCP | 02:24:08 | 131.55 |
| 13 | 170 | G06 | 25 µM FCCP | 02:24:22 | 130.01 |
| 13 | 171 | G06 | 25 µM FCCP | 02:24:36 | 128.59 |
| 13 | 172 | G06 | 25 µM FCCP | 02:24:51 | 127.16 |
| 13 | 173 | G06 | 25 µM FCCP | 02:25:07 | 125.97 |
| 13 | 174 | G06 | 25 µM FCCP | 02:25:22 | 124.67 |
| 13 | 175 | G06 | 25 µM FCCP | 02:25:36 | 123.79 |
| 13 | 176 | G06 | 25 µM FCCP | 02:25:50 | 122.80 |
| 13 | 177 | G06 | 25 µM FCCP | 02:26:04 | 121.87 |
| 13 | 178 | G06 | 25 µM FCCP | 02:26:18 | 120.90 |
| 13 | 179 | G06 | 25 µM FCCP | 02:26:33 | 120.00 |
| 14 | 180 | G06 | 25 µM FCCP | 02:32:07 | 137.32 |
| 14 | 181 | G06 | 25 µM FCCP | 02:32:21 | 134.57 |
| 14 | 182 | G06 | 25 µM FCCP | 02:32:35 | 132.84 |
| 14 | 183 | G06 | 25 µM FCCP | 02:32:50 | 131.28 |
| 14 | 184 | G06 | 25 µM FCCP | 02:33:04 | 129.97 |
| 14 | 185 | G06 | 25 µM FCCP | 02:33:18 | 128.65 |
| 14 | 186 | G06 | 25 µM FCCP | 02:33:32 | 127.59 |
| 14 | 187 | G06 | 25 µM FCCP | 02:33:46 | 126.51 |
| 14 | 188 | G06 | 25 µM FCCP | 02:34:01 | 125.49 |
| 14 | 189 | G06 | 25 µM FCCP | 02:34:15 | 124.57 |
| 14 | 190 | G06 | 25 µM FCCP | 02:34:29 | 123.69 |
| 14 | 191 | G06 | 25 µM FCCP | 02:34:43 | 122.69 |
| 14 | 192 | G06 | 25 µM FCCP | 02:34:57 | 121.86 |
| 14 | 193 | G06 | 25 µM FCCP | 02:35:12 | 121.12 |
| 15 | 194 | G06 | 25 µM FCCP | 02:40:46 | 137.89 |
| 15 | 195 | G06 | 25 µM FCCP | 02:41:00 | 135.38 |
| 15 | 196 | G06 | 25 µM FCCP | 02:41:14 | 133.64 |
| 15 | 197 | G06 | 25 µM FCCP | 02:41:28 | 132.22 |
| 15 | 198 | G06 | 25 µM FCCP | 02:41:43 | 130.87 |
| 15 | 199 | G06 | 25 µM FCCP | 02:41:57 | 129.75 |
| 15 | 200 | G06 | 25 µM FCCP | 02:42:11 | 128.64 |
| 15 | 201 | G06 | 25 µM FCCP | 02:42:25 | 127.56 |
| 15 | 202 | G06 | 25 µM FCCP | 02:42:39 | 126.71 |
| 15 | 203 | G06 | 25 µM FCCP | 02:42:53 | 125.82 |
| 15 | 204 | G06 | 25 µM FCCP | 02:43:08 | 124.87 |
| 15 | 205 | G06 | 25 µM FCCP | 02:43:22 | 124.06 |
| 15 | 206 | G06 | 25 µM FCCP | 02:43:36 | 123.29 |
| 15 | 207 | G06 | 25 µM FCCP | 02:43:50 | 122.53 |
| 16 | 208 | G06 | 25 µM FCCP | 02:49:25 | 138.44 |
| 16 | 209 | G06 | 25 µM FCCP | 02:49:40 | 136.05 |
| 16 | 210 | G06 | 25 µM FCCP | 02:49:54 | 134.36 |
| 16 | 211 | G06 | 25 µM FCCP | 02:50:08 | 133.02 |
| 16 | 212 | G06 | 25 µM FCCP | 02:50:22 | 131.84 |
| 16 | 213 | G06 | 25 µM FCCP | 02:50:36 | 130.70 |
| 16 | 214 | G06 | 25 µM FCCP | 02:50:50 | 129.71 |
| 16 | 215 | G06 | 25 µM FCCP | 02:51:05 | 128.56 |
| 16 | 216 | G06 | 25 µM FCCP | 02:51:19 | 127.67 |
| 16 | 217 | G06 | 25 µM FCCP | 02:51:33 | 126.82 |
| 16 | 218 | G06 | 25 µM FCCP | 02:51:47 | 126.04 |
| 16 | 219 | G06 | 25 µM FCCP | 02:52:01 | 125.29 |
| 16 | 220 | G06 | 25 µM FCCP | 02:52:16 | 124.47 |
| 16 | 221 | G06 | 25 µM FCCP | 02:52:30 | 123.77 |
| 1 | 0 | E01 | 5 µM BAM15 | 00:40:06 | 140.67 |
| 1 | 1 | E01 | 5 µM BAM15 | 00:40:20 | 136.98 |
| 1 | 2 | E01 | 5 µM BAM15 | 00:40:34 | 135.11 |
| 1 | 3 | E01 | 5 µM BAM15 | 00:40:48 | 133.49 |
| 1 | 4 | E01 | 5 µM BAM15 | 00:41:02 | 132.07 |
| 1 | 5 | E01 | 5 µM BAM15 | 00:41:17 | 130.73 |
| 1 | 6 | E01 | 5 µM BAM15 | 00:41:31 | 129.71 |
| 1 | 7 | E01 | 5 µM BAM15 | 00:41:45 | 128.53 |
| 1 | 8 | E01 | 5 µM BAM15 | 00:41:59 | 127.46 |
| 1 | 9 | E01 | 5 µM BAM15 | 00:42:13 | 126.49 |
| 1 | 10 | E01 | 5 µM BAM15 | 00:42:28 | 125.47 |
| 1 | 11 | E01 | 5 µM BAM15 | 00:42:42 | 124.47 |
| 1 | 12 | E01 | 5 µM BAM15 | 00:42:56 | 123.60 |
| 1 | 13 | E01 | 5 µM BAM15 | 00:43:10 | 122.77 |
| 2 | 14 | E01 | 5 µM BAM15 | 00:48:43 | 141.65 |
| 2 | 15 | E01 | 5 µM BAM15 | 00:48:57 | 137.85 |
| 2 | 16 | E01 | 5 µM BAM15 | 00:49:12 | 135.92 |
| 2 | 17 | E01 | 5 µM BAM15 | 00:49:26 | 134.19 |
| 2 | 18 | E01 | 5 µM BAM15 | 00:49:40 | 132.84 |
| 2 | 19 | E01 | 5 µM BAM15 | 00:49:54 | 131.40 |
| 2 | 20 | E01 | 5 µM BAM15 | 00:50:08 | 130.18 |
| 2 | 21 | E01 | 5 µM BAM15 | 00:50:22 | 128.93 |
| 2 | 22 | E01 | 5 µM BAM15 | 00:50:37 | 127.97 |
| 2 | 23 | E01 | 5 µM BAM15 | 00:50:51 | 126.95 |
| 2 | 24 | E01 | 5 µM BAM15 | 00:51:05 | 125.82 |
| 2 | 25 | E01 | 5 µM BAM15 | 00:51:19 | 125.05 |
| 2 | 26 | E01 | 5 µM BAM15 | 00:51:33 | 123.85 |
| 2 | 27 | E01 | 5 µM BAM15 | 00:51:48 | 123.02 |
| 3 | 28 | E01 | 5 µM BAM15 | 00:57:21 | 142.32 |
| 3 | 29 | E01 | 5 µM BAM15 | 00:57:35 | 138.62 |
| 3 | 30 | E01 | 5 µM BAM15 | 00:57:49 | 136.54 |
| 3 | 31 | E01 | 5 µM BAM15 | 00:58:03 | 134.95 |
| 3 | 32 | E01 | 5 µM BAM15 | 00:58:17 | 133.50 |
| 3 | 33 | E01 | 5 µM BAM15 | 00:58:31 | 132.06 |
| 3 | 34 | E01 | 5 µM BAM15 | 00:58:46 | 130.92 |
| 3 | 35 | E01 | 5 µM BAM15 | 00:59:00 | 129.65 |
| 3 | 36 | E01 | 5 µM BAM15 | 00:59:14 | 128.54 |
| 3 | 37 | E01 | 5 µM BAM15 | 00:59:28 | 127.57 |
| 3 | 38 | E01 | 5 µM BAM15 | 00:59:42 | 126.49 |
| 3 | 39 | E01 | 5 µM BAM15 | 00:59:56 | 125.54 |
| 3 | 40 | E01 | 5 µM BAM15 | 01:00:11 | 124.57 |
| 3 | 41 | E01 | 5 µM BAM15 | 01:00:25 | 123.70 |
| 4 | 42 | E01 | 5 µM BAM15 | 01:06:04 | 134.70 |
| 4 | 43 | E01 | 5 µM BAM15 | 01:06:18 | 124.80 |
| 4 | 44 | E01 | 5 µM BAM15 | 01:06:33 | 119.61 |
| 4 | 45 | E01 | 5 µM BAM15 | 01:06:47 | 115.52 |
| 4 | 46 | E01 | 5 µM BAM15 | 01:07:01 | 112.01 |
| 4 | 47 | E01 | 5 µM BAM15 | 01:07:15 | 108.41 |
| 4 | 48 | E01 | 5 µM BAM15 | 01:07:29 | 105.05 |
| 4 | 49 | E01 | 5 µM BAM15 | 01:07:43 | 101.60 |
| 4 | 50 | E01 | 5 µM BAM15 | 01:07:58 | 98.36 |
| 4 | 51 | E01 | 5 µM BAM15 | 01:08:12 | 95.09 |
| 4 | 52 | E01 | 5 µM BAM15 | 01:08:26 | 91.73 |
| 4 | 53 | E01 | 5 µM BAM15 | 01:08:40 | 88.58 |
| 4 | 54 | E01 | 5 µM BAM15 | 01:08:54 | 85.29 |
| 4 | 55 | E01 | 5 µM BAM15 | 01:09:09 | 82.11 |
| 5 | 56 | E01 | 5 µM BAM15 | 01:14:42 | 126.72 |
| 5 | 57 | E01 | 5 µM BAM15 | 01:14:56 | 113.56 |
| 5 | 58 | E01 | 5 µM BAM15 | 01:15:10 | 106.70 |
| 5 | 59 | E01 | 5 µM BAM15 | 01:15:25 | 101.55 |
| 5 | 60 | E01 | 5 µM BAM15 | 01:15:39 | 96.90 |
| 5 | 61 | E01 | 5 µM BAM15 | 01:15:53 | 92.49 |
| 5 | 62 | E01 | 5 µM BAM15 | 01:16:07 | 88.42 |
| 5 | 63 | E01 | 5 µM BAM15 | 01:16:21 | 84.52 |
| 5 | 64 | E01 | 5 µM BAM15 | 01:16:35 | 80.83 |
| 5 | 65 | E01 | 5 µM BAM15 | 01:16:50 | 77.27 |
| 5 | 66 | E01 | 5 µM BAM15 | 01:17:04 | 73.64 |
| 5 | 67 | E01 | 5 µM BAM15 | 01:17:18 | 70.24 |
| 5 | 68 | E01 | 5 µM BAM15 | 01:17:32 | 66.84 |
| 5 | 69 | E01 | 5 µM BAM15 | 01:17:46 | 63.66 |
| 6 | 70 | E01 | 5 µM BAM15 | 01:23:20 | 121.70 |
| 6 | 71 | E01 | 5 µM BAM15 | 01:23:34 | 106.67 |
| 6 | 72 | E01 | 5 µM BAM15 | 01:23:48 | 98.99 |
| 6 | 73 | E01 | 5 µM BAM15 | 01:24:02 | 92.97 |
| 6 | 74 | E01 | 5 µM BAM15 | 01:24:16 | 87.75 |
| 6 | 75 | E01 | 5 µM BAM15 | 01:24:30 | 82.94 |
| 6 | 76 | E01 | 5 µM BAM15 | 01:24:44 | 78.66 |
| 6 | 77 | E01 | 5 µM BAM15 | 01:24:59 | 74.43 |
| 6 | 78 | E01 | 5 µM BAM15 | 01:25:13 | 70.56 |
| 6 | 79 | E01 | 5 µM BAM15 | 01:25:27 | 66.87 |
| 6 | 80 | E01 | 5 µM BAM15 | 01:25:41 | 63.22 |
| 6 | 81 | E01 | 5 µM BAM15 | 01:25:55 | 59.88 |
| 6 | 82 | E01 | 5 µM BAM15 | 01:26:10 | 56.57 |
| 6 | 83 | E01 | 5 µM BAM15 | 01:26:24 | 53.51 |
| 7 | 84 | E01 | 5 µM BAM15 | 01:31:58 | 118.69 |
| 7 | 85 | E01 | 5 µM BAM15 | 01:32:12 | 102.96 |
| 7 | 86 | E01 | 5 µM BAM15 | 01:32:26 | 94.83 |
| 7 | 87 | E01 | 5 µM BAM15 | 01:32:40 | 88.60 |
| 7 | 88 | E01 | 5 µM BAM15 | 01:32:54 | 83.15 |
| 7 | 89 | E01 | 5 µM BAM15 | 01:33:08 | 78.22 |
| 7 | 90 | E01 | 5 µM BAM15 | 01:33:23 | 73.76 |
| 7 | 91 | E01 | 5 µM BAM15 | 01:33:37 | 69.51 |
| 7 | 92 | E01 | 5 µM BAM15 | 01:33:51 | 65.64 |
| 7 | 93 | E01 | 5 µM BAM15 | 01:34:05 | 61.95 |
| 7 | 94 | E01 | 5 µM BAM15 | 01:34:19 | 58.41 |
| 7 | 95 | E01 | 5 µM BAM15 | 01:34:33 | 55.21 |
| 7 | 96 | E01 | 5 µM BAM15 | 01:34:48 | 52.09 |
| 7 | 97 | E01 | 5 µM BAM15 | 01:35:02 | 49.23 |
| 8 | 98 | E01 | 5 µM BAM15 | 01:40:36 | 117.19 |
| 8 | 99 | E01 | 5 µM BAM15 | 01:40:50 | 101.65 |
| 8 | 100 | E01 | 5 µM BAM15 | 01:41:04 | 93.45 |
| 8 | 101 | E01 | 5 µM BAM15 | 01:41:18 | 86.99 |
| 8 | 102 | E01 | 5 µM BAM15 | 01:41:32 | 81.51 |
| 8 | 103 | E01 | 5 µM BAM15 | 01:41:46 | 76.47 |
| 8 | 104 | E01 | 5 µM BAM15 | 01:42:00 | 71.95 |
| 8 | 105 | E01 | 5 µM BAM15 | 01:42:15 | 67.68 |
| 8 | 106 | E01 | 5 µM BAM15 | 01:42:29 | 63.71 |
| 8 | 107 | E01 | 5 µM BAM15 | 01:42:43 | 60.07 |
| 8 | 108 | E01 | 5 µM BAM15 | 01:42:57 | 56.56 |
| 8 | 109 | E01 | 5 µM BAM15 | 01:43:11 | 53.35 |
| 8 | 110 | E01 | 5 µM BAM15 | 01:43:26 | 50.28 |
| 8 | 111 | E01 | 5 µM BAM15 | 01:43:40 | 47.49 |
| 9 | 112 | E01 | 5 µM BAM15 | 01:49:14 | 117.58 |
| 9 | 113 | E01 | 5 µM BAM15 | 01:49:28 | 104.01 |
| 9 | 114 | E01 | 5 µM BAM15 | 01:49:42 | 96.27 |
| 9 | 115 | E01 | 5 µM BAM15 | 01:49:56 | 89.95 |
| 9 | 116 | E01 | 5 µM BAM15 | 01:50:10 | 84.45 |
| 9 | 117 | E01 | 5 µM BAM15 | 01:50:24 | 79.39 |
| 9 | 118 | E01 | 5 µM BAM15 | 01:50:38 | 74.81 |
| 9 | 119 | E01 | 5 µM BAM15 | 01:50:53 | 70.40 |
| 9 | 120 | E01 | 5 µM BAM15 | 01:51:07 | 66.42 |
| 9 | 121 | E01 | 5 µM BAM15 | 01:51:21 | 62.57 |
| 9 | 122 | E01 | 5 µM BAM15 | 01:51:35 | 59.03 |
| 9 | 123 | E01 | 5 µM BAM15 | 01:51:50 | 55.70 |
| 9 | 124 | E01 | 5 µM BAM15 | 01:52:04 | 52.49 |
| 9 | 125 | E01 | 5 µM BAM15 | 01:52:18 | 49.58 |
| 10 | 126 | E01 | 5 µM BAM15 | 01:57:52 | 117.68 |
| 10 | 127 | E01 | 5 µM BAM15 | 01:58:06 | 104.64 |
| 10 | 128 | E01 | 5 µM BAM15 | 01:58:20 | 96.94 |
| 10 | 129 | E01 | 5 µM BAM15 | 01:58:34 | 90.80 |
| 10 | 130 | E01 | 5 µM BAM15 | 01:58:48 | 85.35 |
| 10 | 131 | E01 | 5 µM BAM15 | 01:59:03 | 80.28 |
| 10 | 132 | E01 | 5 µM BAM15 | 01:59:17 | 75.67 |
| 10 | 133 | E01 | 5 µM BAM15 | 01:59:31 | 71.31 |
| 10 | 134 | E01 | 5 µM BAM15 | 01:59:45 | 67.38 |
| 10 | 135 | E01 | 5 µM BAM15 | 01:59:59 | 63.55 |
| 10 | 136 | E01 | 5 µM BAM15 | 02:00:14 | 59.96 |
| 10 | 137 | E01 | 5 µM BAM15 | 02:00:28 | 56.65 |
| 10 | 138 | E01 | 5 µM BAM15 | 02:00:42 | 53.47 |
| 10 | 139 | E01 | 5 µM BAM15 | 02:00:56 | 50.58 |
| 11 | 140 | E01 | 5 µM BAM15 | 02:06:30 | 117.56 |
| 11 | 141 | E01 | 5 µM BAM15 | 02:06:44 | 104.59 |
| 11 | 142 | E01 | 5 µM BAM15 | 02:06:58 | 96.99 |
| 11 | 143 | E01 | 5 µM BAM15 | 02:07:12 | 90.85 |
| 11 | 144 | E01 | 5 µM BAM15 | 02:07:26 | 85.52 |
| 11 | 145 | E01 | 5 µM BAM15 | 02:07:41 | 80.44 |
| 11 | 146 | E01 | 5 µM BAM15 | 02:07:55 | 75.86 |
| 11 | 147 | E01 | 5 µM BAM15 | 02:08:09 | 71.54 |
| 11 | 148 | E01 | 5 µM BAM15 | 02:08:23 | 67.61 |
| 11 | 149 | E01 | 5 µM BAM15 | 02:08:37 | 63.89 |
| 11 | 150 | E01 | 5 µM BAM15 | 02:08:52 | 60.28 |
| 11 | 151 | E01 | 5 µM BAM15 | 02:09:06 | 56.91 |
| 11 | 152 | E01 | 5 µM BAM15 | 02:09:20 | 53.76 |
| 11 | 153 | E01 | 5 µM BAM15 | 02:09:34 | 50.80 |
| 12 | 154 | E01 | 5 µM BAM15 | 02:15:08 | 117.29 |
| 12 | 155 | E01 | 5 µM BAM15 | 02:15:22 | 104.45 |
| 12 | 156 | E01 | 5 µM BAM15 | 02:15:37 | 96.44 |
| 12 | 157 | E01 | 5 µM BAM15 | 02:15:53 | 89.75 |
| 12 | 158 | E01 | 5 µM BAM15 | 02:16:08 | 84.28 |
| 12 | 159 | E01 | 5 µM BAM15 | 02:16:22 | 79.54 |
| 12 | 160 | E01 | 5 µM BAM15 | 02:16:36 | 75.03 |
| 12 | 161 | E01 | 5 µM BAM15 | 02:16:50 | 70.85 |
| 12 | 162 | E01 | 5 µM BAM15 | 02:17:04 | 66.90 |
| 12 | 163 | E01 | 5 µM BAM15 | 02:17:18 | 63.14 |
| 12 | 164 | E01 | 5 µM BAM15 | 02:17:33 | 59.69 |
| 12 | 165 | E01 | 5 µM BAM15 | 02:17:47 | 56.41 |
| 12 | 166 | E01 | 5 µM BAM15 | 02:18:01 | 53.26 |
| 13 | 167 | E01 | 5 µM BAM15 | 02:23:39 | 113.09 |
| 13 | 168 | E01 | 5 µM BAM15 | 02:23:54 | 102.48 |
| 13 | 169 | E01 | 5 µM BAM15 | 02:24:08 | 95.39 |
| 13 | 170 | E01 | 5 µM BAM15 | 02:24:22 | 89.56 |
| 13 | 171 | E01 | 5 µM BAM15 | 02:24:36 | 84.24 |
| 13 | 172 | E01 | 5 µM BAM15 | 02:24:51 | 79.13 |
| 13 | 173 | E01 | 5 µM BAM15 | 02:25:07 | 74.18 |
| 13 | 174 | E01 | 5 µM BAM15 | 02:25:22 | 69.97 |
| 13 | 175 | E01 | 5 µM BAM15 | 02:25:36 | 66.25 |
| 13 | 176 | E01 | 5 µM BAM15 | 02:25:50 | 62.53 |
| 13 | 177 | E01 | 5 µM BAM15 | 02:26:04 | 59.15 |
| 13 | 178 | E01 | 5 µM BAM15 | 02:26:18 | 55.87 |
| 13 | 179 | E01 | 5 µM BAM15 | 02:26:33 | 52.82 |
| 14 | 180 | E01 | 5 µM BAM15 | 02:32:07 | 117.16 |
| 14 | 181 | E01 | 5 µM BAM15 | 02:32:21 | 104.55 |
| 14 | 182 | E01 | 5 µM BAM15 | 02:32:35 | 97.15 |
| 14 | 183 | E01 | 5 µM BAM15 | 02:32:50 | 91.12 |
| 14 | 184 | E01 | 5 µM BAM15 | 02:33:04 | 85.80 |
| 14 | 185 | E01 | 5 µM BAM15 | 02:33:18 | 80.83 |
| 14 | 186 | E01 | 5 µM BAM15 | 02:33:32 | 76.36 |
| 14 | 187 | E01 | 5 µM BAM15 | 02:33:46 | 72.07 |
| 14 | 188 | E01 | 5 µM BAM15 | 02:34:01 | 68.17 |
| 14 | 189 | E01 | 5 µM BAM15 | 02:34:15 | 64.54 |
| 14 | 190 | E01 | 5 µM BAM15 | 02:34:29 | 60.95 |
| 14 | 191 | E01 | 5 µM BAM15 | 02:34:43 | 57.66 |
| 14 | 192 | E01 | 5 µM BAM15 | 02:34:57 | 54.46 |
| 14 | 193 | E01 | 5 µM BAM15 | 02:35:12 | 51.62 |
| 15 | 194 | E01 | 5 µM BAM15 | 02:40:46 | 117.25 |
| 15 | 195 | E01 | 5 µM BAM15 | 02:41:00 | 104.84 |
| 15 | 196 | E01 | 5 µM BAM15 | 02:41:14 | 97.38 |
| 15 | 197 | E01 | 5 µM BAM15 | 02:41:28 | 91.32 |
| 15 | 198 | E01 | 5 µM BAM15 | 02:41:43 | 86.03 |
| 15 | 199 | E01 | 5 µM BAM15 | 02:41:57 | 81.24 |
| 15 | 200 | E01 | 5 µM BAM15 | 02:42:11 | 76.79 |
| 15 | 201 | E01 | 5 µM BAM15 | 02:42:25 | 72.50 |
| 15 | 202 | E01 | 5 µM BAM15 | 02:42:39 | 68.69 |
| 15 | 203 | E01 | 5 µM BAM15 | 02:42:53 | 64.99 |
| 15 | 204 | E01 | 5 µM BAM15 | 02:43:08 | 61.41 |
| 15 | 205 | E01 | 5 µM BAM15 | 02:43:22 | 58.23 |
| 15 | 206 | E01 | 5 µM BAM15 | 02:43:36 | 54.95 |
| 15 | 207 | E01 | 5 µM BAM15 | 02:43:50 | 52.08 |
| 16 | 208 | E01 | 5 µM BAM15 | 02:49:25 | 117.38 |
| 16 | 209 | E01 | 5 µM BAM15 | 02:49:40 | 104.90 |
| 16 | 210 | E01 | 5 µM BAM15 | 02:49:54 | 97.52 |
| 16 | 211 | E01 | 5 µM BAM15 | 02:50:08 | 91.57 |
| 16 | 212 | E01 | 5 µM BAM15 | 02:50:22 | 86.36 |
| 16 | 213 | E01 | 5 µM BAM15 | 02:50:36 | 81.49 |
| 16 | 214 | E01 | 5 µM BAM15 | 02:50:50 | 77.10 |
| 16 | 215 | E01 | 5 µM BAM15 | 02:51:05 | 72.87 |
| 16 | 216 | E01 | 5 µM BAM15 | 02:51:19 | 69.00 |
| 16 | 217 | E01 | 5 µM BAM15 | 02:51:33 | 65.31 |
| 16 | 218 | E01 | 5 µM BAM15 | 02:51:47 | 61.88 |
| 16 | 219 | E01 | 5 µM BAM15 | 02:52:01 | 58.63 |
| 16 | 220 | E01 | 5 µM BAM15 | 02:52:16 | 55.45 |
| 16 | 221 | E01 | 5 µM BAM15 | 02:52:30 | 52.54 |
| 1 | 0 | E02 | 5 µM BAM15 | 00:40:06 | 129.89 |
| 1 | 1 | E02 | 5 µM BAM15 | 00:40:20 | 123.01 |
| 1 | 2 | E02 | 5 µM BAM15 | 00:40:34 | 119.96 |
| 1 | 3 | E02 | 5 µM BAM15 | 00:40:48 | 117.85 |
| 1 | 4 | E02 | 5 µM BAM15 | 00:41:02 | 115.95 |
| 1 | 5 | E02 | 5 µM BAM15 | 00:41:17 | 114.23 |
| 1 | 6 | E02 | 5 µM BAM15 | 00:41:31 | 112.65 |
| 1 | 7 | E02 | 5 µM BAM15 | 00:41:45 | 111.32 |
| 1 | 8 | E02 | 5 µM BAM15 | 00:41:59 | 109.92 |
| 1 | 9 | E02 | 5 µM BAM15 | 00:42:13 | 108.69 |
| 1 | 10 | E02 | 5 µM BAM15 | 00:42:28 | 107.37 |
| 1 | 11 | E02 | 5 µM BAM15 | 00:42:42 | 106.21 |
| 1 | 12 | E02 | 5 µM BAM15 | 00:42:56 | 105.09 |
| 1 | 13 | E02 | 5 µM BAM15 | 00:43:10 | 103.94 |
| 2 | 14 | E02 | 5 µM BAM15 | 00:48:43 | 133.44 |
| 2 | 15 | E02 | 5 µM BAM15 | 00:48:57 | 128.75 |
| 2 | 16 | E02 | 5 µM BAM15 | 00:49:12 | 126.19 |
| 2 | 17 | E02 | 5 µM BAM15 | 00:49:26 | 123.97 |
| 2 | 18 | E02 | 5 µM BAM15 | 00:49:40 | 121.98 |
| 2 | 19 | E02 | 5 µM BAM15 | 00:49:54 | 120.14 |
| 2 | 20 | E02 | 5 µM BAM15 | 00:50:08 | 118.56 |
| 2 | 21 | E02 | 5 µM BAM15 | 00:50:22 | 116.98 |
| 2 | 22 | E02 | 5 µM BAM15 | 00:50:37 | 115.59 |
| 2 | 23 | E02 | 5 µM BAM15 | 00:50:51 | 114.24 |
| 2 | 24 | E02 | 5 µM BAM15 | 00:51:05 | 112.89 |
| 2 | 25 | E02 | 5 µM BAM15 | 00:51:19 | 111.63 |
| 2 | 26 | E02 | 5 µM BAM15 | 00:51:33 | 110.36 |
| 2 | 27 | E02 | 5 µM BAM15 | 00:51:48 | 109.17 |
| 3 | 28 | E02 | 5 µM BAM15 | 00:57:21 | 134.03 |
| 3 | 29 | E02 | 5 µM BAM15 | 00:57:35 | 129.74 |
| 3 | 30 | E02 | 5 µM BAM15 | 00:57:49 | 127.15 |
| 3 | 31 | E02 | 5 µM BAM15 | 00:58:03 | 125.03 |
| 3 | 32 | E02 | 5 µM BAM15 | 00:58:17 | 123.23 |
| 3 | 33 | E02 | 5 µM BAM15 | 00:58:31 | 121.41 |
| 3 | 34 | E02 | 5 µM BAM15 | 00:58:46 | 119.83 |
| 3 | 35 | E02 | 5 µM BAM15 | 00:59:00 | 118.33 |
| 3 | 36 | E02 | 5 µM BAM15 | 00:59:14 | 116.90 |
| 3 | 37 | E02 | 5 µM BAM15 | 00:59:28 | 115.49 |
| 3 | 38 | E02 | 5 µM BAM15 | 00:59:42 | 114.17 |
| 3 | 39 | E02 | 5 µM BAM15 | 00:59:56 | 113.00 |
| 3 | 40 | E02 | 5 µM BAM15 | 01:00:11 | 111.70 |
| 3 | 41 | E02 | 5 µM BAM15 | 01:00:25 | 110.53 |
| 4 | 42 | E02 | 5 µM BAM15 | 01:06:04 | 121.42 |
| 4 | 43 | E02 | 5 µM BAM15 | 01:06:18 | 106.42 |
| 4 | 44 | E02 | 5 µM BAM15 | 01:06:33 | 98.17 |
| 4 | 45 | E02 | 5 µM BAM15 | 01:06:47 | 91.61 |
| 4 | 46 | E02 | 5 µM BAM15 | 01:07:01 | 85.85 |
| 4 | 47 | E02 | 5 µM BAM15 | 01:07:15 | 80.25 |
| 4 | 48 | E02 | 5 µM BAM15 | 01:07:29 | 74.90 |
| 4 | 49 | E02 | 5 µM BAM15 | 01:07:43 | 69.66 |
| 4 | 50 | E02 | 5 µM BAM15 | 01:07:58 | 64.63 |
| 4 | 51 | E02 | 5 µM BAM15 | 01:08:12 | 59.64 |
| 4 | 52 | E02 | 5 µM BAM15 | 01:08:26 | 54.66 |
| 4 | 53 | E02 | 5 µM BAM15 | 01:08:40 | 49.87 |
| 4 | 54 | E02 | 5 µM BAM15 | 01:08:54 | 45.00 |
| 4 | 55 | E02 | 5 µM BAM15 | 01:09:09 | 40.64 |
| 5 | 56 | E02 | 5 µM BAM15 | 01:14:42 | 112.00 |
| 5 | 57 | E02 | 5 µM BAM15 | 01:14:56 | 94.39 |
| 5 | 58 | E02 | 5 µM BAM15 | 01:15:10 | 85.03 |
| 5 | 59 | E02 | 5 µM BAM15 | 01:15:25 | 77.65 |
| 5 | 60 | E02 | 5 µM BAM15 | 01:15:39 | 70.97 |
| 5 | 61 | E02 | 5 µM BAM15 | 01:15:53 | 64.67 |
| 5 | 62 | E02 | 5 µM BAM15 | 01:16:07 | 58.80 |
| 5 | 63 | E02 | 5 µM BAM15 | 01:16:21 | 53.06 |
| 5 | 64 | E02 | 5 µM BAM15 | 01:16:35 | 47.70 |
| 5 | 65 | E02 | 5 µM BAM15 | 01:16:50 | 42.54 |
| 5 | 66 | E02 | 5 µM BAM15 | 01:17:04 | 37.99 |
| 5 | 67 | E02 | 5 µM BAM15 | 01:17:18 | 34.39 |
| 5 | 68 | E02 | 5 µM BAM15 | 01:17:32 | 31.47 |
| 5 | 69 | E02 | 5 µM BAM15 | 01:17:46 | 29.12 |
| 6 | 70 | E02 | 5 µM BAM15 | 01:23:20 | 107.89 |
| 6 | 71 | E02 | 5 µM BAM15 | 01:23:34 | 89.13 |
| 6 | 72 | E02 | 5 µM BAM15 | 01:23:48 | 79.05 |
| 6 | 73 | E02 | 5 µM BAM15 | 01:24:02 | 71.01 |
| 6 | 74 | E02 | 5 µM BAM15 | 01:24:16 | 63.88 |
| 6 | 75 | E02 | 5 µM BAM15 | 01:24:30 | 57.16 |
| 6 | 76 | E02 | 5 µM BAM15 | 01:24:44 | 51.09 |
| 6 | 77 | E02 | 5 µM BAM15 | 01:24:59 | 45.14 |
| 6 | 78 | E02 | 5 µM BAM15 | 01:25:13 | 39.98 |
| 6 | 79 | E02 | 5 µM BAM15 | 01:25:27 | 35.78 |
| 6 | 80 | E02 | 5 µM BAM15 | 01:25:41 | 32.39 |
| 6 | 81 | E02 | 5 µM BAM15 | 01:25:55 | 29.79 |
| 6 | 82 | E02 | 5 µM BAM15 | 01:26:10 | 27.64 |
| 6 | 83 | E02 | 5 µM BAM15 | 01:26:24 | 26.07 |
| 7 | 84 | E02 | 5 µM BAM15 | 01:31:58 | 105.65 |
| 7 | 85 | E02 | 5 µM BAM15 | 01:32:12 | 86.79 |
| 7 | 86 | E02 | 5 µM BAM15 | 01:32:26 | 76.41 |
| 7 | 87 | E02 | 5 µM BAM15 | 01:32:40 | 68.17 |
| 7 | 88 | E02 | 5 µM BAM15 | 01:32:54 | 60.82 |
| 7 | 89 | E02 | 5 µM BAM15 | 01:33:08 | 54.04 |
| 7 | 90 | E02 | 5 µM BAM15 | 01:33:23 | 47.79 |
| 7 | 91 | E02 | 5 µM BAM15 | 01:33:37 | 42.00 |
| 7 | 92 | E02 | 5 µM BAM15 | 01:33:51 | 37.30 |
| 7 | 93 | E02 | 5 µM BAM15 | 01:34:05 | 33.60 |
| 7 | 94 | E02 | 5 µM BAM15 | 01:34:19 | 30.55 |
| 7 | 95 | E02 | 5 µM BAM15 | 01:34:33 | 28.29 |
| 7 | 96 | E02 | 5 µM BAM15 | 01:34:48 | 26.50 |
| 7 | 97 | E02 | 5 µM BAM15 | 01:35:02 | 25.11 |
| 8 | 98 | E02 | 5 µM BAM15 | 01:40:36 | 104.71 |
| 8 | 99 | E02 | 5 µM BAM15 | 01:40:50 | 85.80 |
| 8 | 100 | E02 | 5 µM BAM15 | 01:41:04 | 75.30 |
| 8 | 101 | E02 | 5 µM BAM15 | 01:41:18 | 66.89 |
| 8 | 102 | E02 | 5 µM BAM15 | 01:41:32 | 59.57 |
| 8 | 103 | E02 | 5 µM BAM15 | 01:41:46 | 52.73 |
| 8 | 104 | E02 | 5 µM BAM15 | 01:42:00 | 46.55 |
| 8 | 105 | E02 | 5 µM BAM15 | 01:42:15 | 40.90 |
| 8 | 106 | E02 | 5 µM BAM15 | 01:42:29 | 36.32 |
| 8 | 107 | E02 | 5 µM BAM15 | 01:42:43 | 32.69 |
| 8 | 108 | E02 | 5 µM BAM15 | 01:42:57 | 29.80 |
| 8 | 109 | E02 | 5 µM BAM15 | 01:43:11 | 27.59 |
| 8 | 110 | E02 | 5 µM BAM15 | 01:43:26 | 25.83 |
| 8 | 111 | E02 | 5 µM BAM15 | 01:43:40 | 24.62 |
| 9 | 112 | E02 | 5 µM BAM15 | 01:49:14 | 102.90 |
| 9 | 113 | E02 | 5 µM BAM15 | 01:49:28 | 84.91 |
| 9 | 114 | E02 | 5 µM BAM15 | 01:49:42 | 74.77 |
| 9 | 115 | E02 | 5 µM BAM15 | 01:49:56 | 66.54 |
| 9 | 116 | E02 | 5 µM BAM15 | 01:50:10 | 59.26 |
| 9 | 117 | E02 | 5 µM BAM15 | 01:50:24 | 52.54 |
| 9 | 118 | E02 | 5 µM BAM15 | 01:50:38 | 46.36 |
| 9 | 119 | E02 | 5 µM BAM15 | 01:50:53 | 40.70 |
| 9 | 120 | E02 | 5 µM BAM15 | 01:51:07 | 36.09 |
| 9 | 121 | E02 | 5 µM BAM15 | 01:51:21 | 32.44 |
| 9 | 122 | E02 | 5 µM BAM15 | 01:51:35 | 29.56 |
| 9 | 123 | E02 | 5 µM BAM15 | 01:51:50 | 27.32 |
| 9 | 124 | E02 | 5 µM BAM15 | 01:52:04 | 25.64 |
| 9 | 125 | E02 | 5 µM BAM15 | 01:52:18 | 24.34 |
| 10 | 126 | E02 | 5 µM BAM15 | 01:57:52 | 103.63 |
| 10 | 127 | E02 | 5 µM BAM15 | 01:58:06 | 87.29 |
| 10 | 128 | E02 | 5 µM BAM15 | 01:58:20 | 77.52 |
| 10 | 129 | E02 | 5 µM BAM15 | 01:58:34 | 69.45 |
| 10 | 130 | E02 | 5 µM BAM15 | 01:58:48 | 62.21 |
| 10 | 131 | E02 | 5 µM BAM15 | 01:59:03 | 55.38 |
| 10 | 132 | E02 | 5 µM BAM15 | 01:59:17 | 49.14 |
| 10 | 133 | E02 | 5 µM BAM15 | 01:59:31 | 43.30 |
| 10 | 134 | E02 | 5 µM BAM15 | 01:59:45 | 38.34 |
| 10 | 135 | E02 | 5 µM BAM15 | 01:59:59 | 34.19 |
| 10 | 136 | E02 | 5 µM BAM15 | 02:00:14 | 30.90 |
| 10 | 137 | E02 | 5 µM BAM15 | 02:00:28 | 28.36 |
| 10 | 138 | E02 | 5 µM BAM15 | 02:00:42 | 26.37 |
| 10 | 139 | E02 | 5 µM BAM15 | 02:00:56 | 24.89 |
| 11 | 140 | E02 | 5 µM BAM15 | 02:06:30 | 104.37 |
| 11 | 141 | E02 | 5 µM BAM15 | 02:06:44 | 88.57 |
| 11 | 142 | E02 | 5 µM BAM15 | 02:06:58 | 78.88 |
| 11 | 143 | E02 | 5 µM BAM15 | 02:07:12 | 70.88 |
| 11 | 144 | E02 | 5 µM BAM15 | 02:07:26 | 63.74 |
| 11 | 145 | E02 | 5 µM BAM15 | 02:07:41 | 56.95 |
| 11 | 146 | E02 | 5 µM BAM15 | 02:07:55 | 50.76 |
| 11 | 147 | E02 | 5 µM BAM15 | 02:08:09 | 44.96 |
| 11 | 148 | E02 | 5 µM BAM15 | 02:08:23 | 39.79 |
| 11 | 149 | E02 | 5 µM BAM15 | 02:08:37 | 35.42 |
| 11 | 150 | E02 | 5 µM BAM15 | 02:08:52 | 31.91 |
| 11 | 151 | E02 | 5 µM BAM15 | 02:09:06 | 29.11 |
| 11 | 152 | E02 | 5 µM BAM15 | 02:09:20 | 26.98 |
| 11 | 153 | E02 | 5 µM BAM15 | 02:09:34 | 25.29 |
| 12 | 154 | E02 | 5 µM BAM15 | 02:15:08 | 104.27 |
| 12 | 155 | E02 | 5 µM BAM15 | 02:15:22 | 88.54 |
| 12 | 156 | E02 | 5 µM BAM15 | 02:15:37 | 78.41 |
| 12 | 157 | E02 | 5 µM BAM15 | 02:15:53 | 69.77 |
| 12 | 158 | E02 | 5 µM BAM15 | 02:16:08 | 62.53 |
| 12 | 159 | E02 | 5 µM BAM15 | 02:16:22 | 56.11 |
| 12 | 160 | E02 | 5 µM BAM15 | 02:16:36 | 50.07 |
| 12 | 161 | E02 | 5 µM BAM15 | 02:16:50 | 44.40 |
| 12 | 162 | E02 | 5 µM BAM15 | 02:17:04 | 39.36 |
| 12 | 163 | E02 | 5 µM BAM15 | 02:17:18 | 35.10 |
| 12 | 164 | E02 | 5 µM BAM15 | 02:17:33 | 31.76 |
| 12 | 165 | E02 | 5 µM BAM15 | 02:17:47 | 29.04 |
| 12 | 166 | E02 | 5 µM BAM15 | 02:18:01 | 26.88 |
| 13 | 167 | E02 | 5 µM BAM15 | 02:23:39 | 99.30 |
| 13 | 168 | E02 | 5 µM BAM15 | 02:23:54 | 86.20 |
| 13 | 169 | E02 | 5 µM BAM15 | 02:24:08 | 77.20 |
| 13 | 170 | E02 | 5 µM BAM15 | 02:24:22 | 69.59 |
| 13 | 171 | E02 | 5 µM BAM15 | 02:24:36 | 62.59 |
| 13 | 172 | E02 | 5 µM BAM15 | 02:24:51 | 55.73 |
| 13 | 173 | E02 | 5 µM BAM15 | 02:25:07 | 49.03 |
| 13 | 174 | E02 | 5 µM BAM15 | 02:25:22 | 43.47 |
| 13 | 175 | E02 | 5 µM BAM15 | 02:25:36 | 38.69 |
| 13 | 176 | E02 | 5 µM BAM15 | 02:25:50 | 34.58 |
| 13 | 177 | E02 | 5 µM BAM15 | 02:26:04 | 31.38 |
| 13 | 178 | E02 | 5 µM BAM15 | 02:26:18 | 28.72 |
| 13 | 179 | E02 | 5 µM BAM15 | 02:26:33 | 26.62 |
| 14 | 180 | E02 | 5 µM BAM15 | 02:32:07 | 104.43 |
| 14 | 181 | E02 | 5 µM BAM15 | 02:32:21 | 88.99 |
| 14 | 182 | E02 | 5 µM BAM15 | 02:32:35 | 79.67 |
| 14 | 183 | E02 | 5 µM BAM15 | 02:32:50 | 71.83 |
| 14 | 184 | E02 | 5 µM BAM15 | 02:33:04 | 64.87 |
| 14 | 185 | E02 | 5 µM BAM15 | 02:33:18 | 58.26 |
| 14 | 186 | E02 | 5 µM BAM15 | 02:33:32 | 52.26 |
| 14 | 187 | E02 | 5 µM BAM15 | 02:33:46 | 46.43 |
| 14 | 188 | E02 | 5 µM BAM15 | 02:34:01 | 41.36 |
| 14 | 189 | E02 | 5 µM BAM15 | 02:34:15 | 36.87 |
| 14 | 190 | E02 | 5 µM BAM15 | 02:34:29 | 33.15 |
| 14 | 191 | E02 | 5 µM BAM15 | 02:34:43 | 30.18 |
| 14 | 192 | E02 | 5 µM BAM15 | 02:34:57 | 27.74 |
| 14 | 193 | E02 | 5 µM BAM15 | 02:35:12 | 25.94 |
| 15 | 194 | E02 | 5 µM BAM15 | 02:40:46 | 104.48 |
| 15 | 195 | E02 | 5 µM BAM15 | 02:41:00 | 89.15 |
| 15 | 196 | E02 | 5 µM BAM15 | 02:41:14 | 79.84 |
| 15 | 197 | E02 | 5 µM BAM15 | 02:41:28 | 72.13 |
| 15 | 198 | E02 | 5 µM BAM15 | 02:41:43 | 65.15 |
| 15 | 199 | E02 | 5 µM BAM15 | 02:41:57 | 58.77 |
| 15 | 200 | E02 | 5 µM BAM15 | 02:42:11 | 52.76 |
| 15 | 201 | E02 | 5 µM BAM15 | 02:42:25 | 47.03 |
| 15 | 202 | E02 | 5 µM BAM15 | 02:42:39 | 41.91 |
| 15 | 203 | E02 | 5 µM BAM15 | 02:42:53 | 37.37 |
| 15 | 204 | E02 | 5 µM BAM15 | 02:43:08 | 33.51 |
| 15 | 205 | E02 | 5 µM BAM15 | 02:43:22 | 30.57 |
| 15 | 206 | E02 | 5 µM BAM15 | 02:43:36 | 27.99 |
| 15 | 207 | E02 | 5 µM BAM15 | 02:43:50 | 26.08 |
| 16 | 208 | E02 | 5 µM BAM15 | 02:49:25 | 104.58 |
| 16 | 209 | E02 | 5 µM BAM15 | 02:49:40 | 89.36 |
| 16 | 210 | E02 | 5 µM BAM15 | 02:49:54 | 80.12 |
| 16 | 211 | E02 | 5 µM BAM15 | 02:50:08 | 72.41 |
| 16 | 212 | E02 | 5 µM BAM15 | 02:50:22 | 65.55 |
| 16 | 213 | E02 | 5 µM BAM15 | 02:50:36 | 59.07 |
| 16 | 214 | E02 | 5 µM BAM15 | 02:50:50 | 53.14 |
| 16 | 215 | E02 | 5 µM BAM15 | 02:51:05 | 47.43 |
| 16 | 216 | E02 | 5 µM BAM15 | 02:51:19 | 42.31 |
| 16 | 217 | E02 | 5 µM BAM15 | 02:51:33 | 37.73 |
| 16 | 218 | E02 | 5 µM BAM15 | 02:51:47 | 33.87 |
| 16 | 219 | E02 | 5 µM BAM15 | 02:52:01 | 30.85 |
| 16 | 220 | E02 | 5 µM BAM15 | 02:52:16 | 28.22 |
| 16 | 221 | E02 | 5 µM BAM15 | 02:52:30 | 26.22 |
| 1 | 0 | E03 | 5 µM BAM15 | 00:40:06 | 130.12 |
| 1 | 1 | E03 | 5 µM BAM15 | 00:40:20 | 123.39 |
| 1 | 2 | E03 | 5 µM BAM15 | 00:40:34 | 120.60 |
| 1 | 3 | E03 | 5 µM BAM15 | 00:40:48 | 118.58 |
| 1 | 4 | E03 | 5 µM BAM15 | 00:41:02 | 116.80 |
| 1 | 5 | E03 | 5 µM BAM15 | 00:41:17 | 115.19 |
| 1 | 6 | E03 | 5 µM BAM15 | 00:41:31 | 113.75 |
| 1 | 7 | E03 | 5 µM BAM15 | 00:41:45 | 112.38 |
| 1 | 8 | E03 | 5 µM BAM15 | 00:41:59 | 111.21 |
| 1 | 9 | E03 | 5 µM BAM15 | 00:42:13 | 109.93 |
| 1 | 10 | E03 | 5 µM BAM15 | 00:42:28 | 108.82 |
| 1 | 11 | E03 | 5 µM BAM15 | 00:42:42 | 107.69 |
| 1 | 12 | E03 | 5 µM BAM15 | 00:42:56 | 106.53 |
| 1 | 13 | E03 | 5 µM BAM15 | 00:43:10 | 105.55 |
| 2 | 14 | E03 | 5 µM BAM15 | 00:48:43 | 132.56 |
| 2 | 15 | E03 | 5 µM BAM15 | 00:48:57 | 127.04 |
| 2 | 16 | E03 | 5 µM BAM15 | 00:49:12 | 124.36 |
| 2 | 17 | E03 | 5 µM BAM15 | 00:49:26 | 122.08 |
| 2 | 18 | E03 | 5 µM BAM15 | 00:49:40 | 120.32 |
| 2 | 19 | E03 | 5 µM BAM15 | 00:49:54 | 118.55 |
| 2 | 20 | E03 | 5 µM BAM15 | 00:50:08 | 117.00 |
| 2 | 21 | E03 | 5 µM BAM15 | 00:50:22 | 115.51 |
| 2 | 22 | E03 | 5 µM BAM15 | 00:50:37 | 114.19 |
| 2 | 23 | E03 | 5 µM BAM15 | 00:50:51 | 112.86 |
| 2 | 24 | E03 | 5 µM BAM15 | 00:51:05 | 111.59 |
| 2 | 25 | E03 | 5 µM BAM15 | 00:51:19 | 110.46 |
| 2 | 26 | E03 | 5 µM BAM15 | 00:51:33 | 109.26 |
| 2 | 27 | E03 | 5 µM BAM15 | 00:51:48 | 108.12 |
| 3 | 28 | E03 | 5 µM BAM15 | 00:57:21 | 133.98 |
| 3 | 29 | E03 | 5 µM BAM15 | 00:57:35 | 129.41 |
| 3 | 30 | E03 | 5 µM BAM15 | 00:57:49 | 126.79 |
| 3 | 31 | E03 | 5 µM BAM15 | 00:58:03 | 124.69 |
| 3 | 32 | E03 | 5 µM BAM15 | 00:58:17 | 122.90 |
| 3 | 33 | E03 | 5 µM BAM15 | 00:58:31 | 121.17 |
| 3 | 34 | E03 | 5 µM BAM15 | 00:58:46 | 119.59 |
| 3 | 35 | E03 | 5 µM BAM15 | 00:59:00 | 118.12 |
| 3 | 36 | E03 | 5 µM BAM15 | 00:59:14 | 116.66 |
| 3 | 37 | E03 | 5 µM BAM15 | 00:59:28 | 115.47 |
| 3 | 38 | E03 | 5 µM BAM15 | 00:59:42 | 114.08 |
| 3 | 39 | E03 | 5 µM BAM15 | 00:59:56 | 112.87 |
| 3 | 40 | E03 | 5 µM BAM15 | 01:00:11 | 111.78 |
| 3 | 41 | E03 | 5 µM BAM15 | 01:00:25 | 110.64 |
| 4 | 42 | E03 | 5 µM BAM15 | 01:06:04 | 122.46 |
| 4 | 43 | E03 | 5 µM BAM15 | 01:06:18 | 109.32 |
| 4 | 44 | E03 | 5 µM BAM15 | 01:06:33 | 102.30 |
| 4 | 45 | E03 | 5 µM BAM15 | 01:06:47 | 96.81 |
| 4 | 46 | E03 | 5 µM BAM15 | 01:07:01 | 91.87 |
| 4 | 47 | E03 | 5 µM BAM15 | 01:07:15 | 87.06 |
| 4 | 48 | E03 | 5 µM BAM15 | 01:07:29 | 82.43 |
| 4 | 49 | E03 | 5 µM BAM15 | 01:07:43 | 77.84 |
| 4 | 50 | E03 | 5 µM BAM15 | 01:07:58 | 73.48 |
| 4 | 51 | E03 | 5 µM BAM15 | 01:08:12 | 69.10 |
| 4 | 52 | E03 | 5 µM BAM15 | 01:08:26 | 64.71 |
| 4 | 53 | E03 | 5 µM BAM15 | 01:08:40 | 60.47 |
| 4 | 54 | E03 | 5 µM BAM15 | 01:08:54 | 56.14 |
| 4 | 55 | E03 | 5 µM BAM15 | 01:09:09 | 51.99 |
| 5 | 56 | E03 | 5 µM BAM15 | 01:14:42 | 114.10 |
| 5 | 57 | E03 | 5 µM BAM15 | 01:14:56 | 98.12 |
| 5 | 58 | E03 | 5 µM BAM15 | 01:15:10 | 89.81 |
| 5 | 59 | E03 | 5 µM BAM15 | 01:15:25 | 83.28 |
| 5 | 60 | E03 | 5 µM BAM15 | 01:15:39 | 77.38 |
| 5 | 61 | E03 | 5 µM BAM15 | 01:15:53 | 71.77 |
| 5 | 62 | E03 | 5 µM BAM15 | 01:16:07 | 66.56 |
| 5 | 63 | E03 | 5 µM BAM15 | 01:16:21 | 61.46 |
| 5 | 64 | E03 | 5 µM BAM15 | 01:16:35 | 56.71 |
| 5 | 65 | E03 | 5 µM BAM15 | 01:16:50 | 51.96 |
| 5 | 66 | E03 | 5 µM BAM15 | 01:17:04 | 47.24 |
| 5 | 67 | E03 | 5 µM BAM15 | 01:17:18 | 42.74 |
| 5 | 68 | E03 | 5 µM BAM15 | 01:17:32 | 38.35 |
| 5 | 69 | E03 | 5 µM BAM15 | 01:17:46 | 34.48 |
| 6 | 70 | E03 | 5 µM BAM15 | 01:23:20 | 109.43 |
| 6 | 71 | E03 | 5 µM BAM15 | 01:23:34 | 91.82 |
| 6 | 72 | E03 | 5 µM BAM15 | 01:23:48 | 82.65 |
| 6 | 73 | E03 | 5 µM BAM15 | 01:24:02 | 75.29 |
| 6 | 74 | E03 | 5 µM BAM15 | 01:24:16 | 68.83 |
| 6 | 75 | E03 | 5 µM BAM15 | 01:24:30 | 62.74 |
| 6 | 76 | E03 | 5 µM BAM15 | 01:24:44 | 57.15 |
| 6 | 77 | E03 | 5 µM BAM15 | 01:24:59 | 51.67 |
| 6 | 78 | E03 | 5 µM BAM15 | 01:25:13 | 46.58 |
| 6 | 79 | E03 | 5 µM BAM15 | 01:25:27 | 41.62 |
| 6 | 80 | E03 | 5 µM BAM15 | 01:25:41 | 37.01 |
| 6 | 81 | E03 | 5 µM BAM15 | 01:25:55 | 33.28 |
| 6 | 82 | E03 | 5 µM BAM15 | 01:26:10 | 30.17 |
| 6 | 83 | E03 | 5 µM BAM15 | 01:26:24 | 27.89 |
| 7 | 84 | E03 | 5 µM BAM15 | 01:31:58 | 106.66 |
| 7 | 85 | E03 | 5 µM BAM15 | 01:32:12 | 88.53 |
| 7 | 86 | E03 | 5 µM BAM15 | 01:32:26 | 78.77 |
| 7 | 87 | E03 | 5 µM BAM15 | 01:32:40 | 71.10 |
| 7 | 88 | E03 | 5 µM BAM15 | 01:32:54 | 64.27 |
| 7 | 89 | E03 | 5 µM BAM15 | 01:33:08 | 57.97 |
| 7 | 90 | E03 | 5 µM BAM15 | 01:33:23 | 52.10 |
| 7 | 91 | E03 | 5 µM BAM15 | 01:33:37 | 46.50 |
| 7 | 92 | E03 | 5 µM BAM15 | 01:33:51 | 41.39 |
| 7 | 93 | E03 | 5 µM BAM15 | 01:34:05 | 36.85 |
| 7 | 94 | E03 | 5 µM BAM15 | 01:34:19 | 32.96 |
| 7 | 95 | E03 | 5 µM BAM15 | 01:34:33 | 30.03 |
| 7 | 96 | E03 | 5 µM BAM15 | 01:34:48 | 27.75 |
| 7 | 97 | E03 | 5 µM BAM15 | 01:35:02 | 26.07 |
| 8 | 98 | E03 | 5 µM BAM15 | 01:40:36 | 105.30 |
| 8 | 99 | E03 | 5 µM BAM15 | 01:40:50 | 87.05 |
| 8 | 100 | E03 | 5 µM BAM15 | 01:41:04 | 77.12 |
| 8 | 101 | E03 | 5 µM BAM15 | 01:41:18 | 69.25 |
| 8 | 102 | E03 | 5 µM BAM15 | 01:41:32 | 62.38 |
| 8 | 103 | E03 | 5 µM BAM15 | 01:41:46 | 55.98 |
| 8 | 104 | E03 | 5 µM BAM15 | 01:42:00 | 50.11 |
| 8 | 105 | E03 | 5 µM BAM15 | 01:42:15 | 44.60 |
| 8 | 106 | E03 | 5 µM BAM15 | 01:42:29 | 39.56 |
| 8 | 107 | E03 | 5 µM BAM15 | 01:42:43 | 35.27 |
| 8 | 108 | E03 | 5 µM BAM15 | 01:42:57 | 31.70 |
| 8 | 109 | E03 | 5 µM BAM15 | 01:43:11 | 29.00 |
| 8 | 110 | E03 | 5 µM BAM15 | 01:43:26 | 26.91 |
| 8 | 111 | E03 | 5 µM BAM15 | 01:43:40 | 25.44 |
| 9 | 112 | E03 | 5 µM BAM15 | 01:49:14 | 104.31 |
| 9 | 113 | E03 | 5 µM BAM15 | 01:49:28 | 86.17 |
| 9 | 114 | E03 | 5 µM BAM15 | 01:49:42 | 76.31 |
| 9 | 115 | E03 | 5 µM BAM15 | 01:49:56 | 68.43 |
| 9 | 116 | E03 | 5 µM BAM15 | 01:50:10 | 61.53 |
| 9 | 117 | E03 | 5 µM BAM15 | 01:50:24 | 55.19 |
| 9 | 118 | E03 | 5 µM BAM15 | 01:50:38 | 49.32 |
| 9 | 119 | E03 | 5 µM BAM15 | 01:50:53 | 43.81 |
| 9 | 120 | E03 | 5 µM BAM15 | 01:51:07 | 38.89 |
| 9 | 121 | E03 | 5 µM BAM15 | 01:51:21 | 34.65 |
| 9 | 122 | E03 | 5 µM BAM15 | 01:51:35 | 31.19 |
| 9 | 123 | E03 | 5 µM BAM15 | 01:51:50 | 28.51 |
| 9 | 124 | E03 | 5 µM BAM15 | 01:52:04 | 26.56 |
| 9 | 125 | E03 | 5 µM BAM15 | 01:52:18 | 25.08 |
| 10 | 126 | E03 | 5 µM BAM15 | 01:57:52 | 103.96 |
| 10 | 127 | E03 | 5 µM BAM15 | 01:58:06 | 86.01 |
| 10 | 128 | E03 | 5 µM BAM15 | 01:58:20 | 76.07 |
| 10 | 129 | E03 | 5 µM BAM15 | 01:58:34 | 68.33 |
| 10 | 130 | E03 | 5 µM BAM15 | 01:58:48 | 61.41 |
| 10 | 131 | E03 | 5 µM BAM15 | 01:59:03 | 54.97 |
| 10 | 132 | E03 | 5 µM BAM15 | 01:59:17 | 49.14 |
| 10 | 133 | E03 | 5 µM BAM15 | 01:59:31 | 43.65 |
| 10 | 134 | E03 | 5 µM BAM15 | 01:59:45 | 38.85 |
| 10 | 135 | E03 | 5 µM BAM15 | 01:59:59 | 34.54 |
| 10 | 136 | E03 | 5 µM BAM15 | 02:00:14 | 31.07 |
| 10 | 137 | E03 | 5 µM BAM15 | 02:00:28 | 28.44 |
| 10 | 138 | E03 | 5 µM BAM15 | 02:00:42 | 26.43 |
| 10 | 139 | E03 | 5 µM BAM15 | 02:00:56 | 25.02 |
| 11 | 140 | E03 | 5 µM BAM15 | 02:06:30 | 103.92 |
| 11 | 141 | E03 | 5 µM BAM15 | 02:06:44 | 86.74 |
| 11 | 142 | E03 | 5 µM BAM15 | 02:06:58 | 77.00 |
| 11 | 143 | E03 | 5 µM BAM15 | 02:07:12 | 69.14 |
| 11 | 144 | E03 | 5 µM BAM15 | 02:07:26 | 62.33 |
| 11 | 145 | E03 | 5 µM BAM15 | 02:07:41 | 55.85 |
| 11 | 146 | E03 | 5 µM BAM15 | 02:07:55 | 50.03 |
| 11 | 147 | E03 | 5 µM BAM15 | 02:08:09 | 44.54 |
| 11 | 148 | E03 | 5 µM BAM15 | 02:08:23 | 39.52 |
| 11 | 149 | E03 | 5 µM BAM15 | 02:08:37 | 35.16 |
| 11 | 150 | E03 | 5 µM BAM15 | 02:08:52 | 31.49 |
| 11 | 151 | E03 | 5 µM BAM15 | 02:09:06 | 28.64 |
| 11 | 152 | E03 | 5 µM BAM15 | 02:09:20 | 26.59 |
| 11 | 153 | E03 | 5 µM BAM15 | 02:09:34 | 25.03 |
| 12 | 154 | E03 | 5 µM BAM15 | 02:15:08 | 105.42 |
| 12 | 155 | E03 | 5 µM BAM15 | 02:15:22 | 89.48 |
| 12 | 156 | E03 | 5 µM BAM15 | 02:15:37 | 79.35 |
| 12 | 157 | E03 | 5 µM BAM15 | 02:15:53 | 70.84 |
| 12 | 158 | E03 | 5 µM BAM15 | 02:16:08 | 63.82 |
| 12 | 159 | E03 | 5 µM BAM15 | 02:16:22 | 57.63 |
| 12 | 160 | E03 | 5 µM BAM15 | 02:16:36 | 51.76 |
| 12 | 161 | E03 | 5 µM BAM15 | 02:16:50 | 46.24 |
| 12 | 162 | E03 | 5 µM BAM15 | 02:17:04 | 41.20 |
| 12 | 163 | E03 | 5 µM BAM15 | 02:17:18 | 36.52 |
| 12 | 164 | E03 | 5 µM BAM15 | 02:17:33 | 32.67 |
| 12 | 165 | E03 | 5 µM BAM15 | 02:17:47 | 29.57 |
| 12 | 166 | E03 | 5 µM BAM15 | 02:18:01 | 27.17 |
| 13 | 167 | E03 | 5 µM BAM15 | 02:23:39 | 100.74 |
| 13 | 168 | E03 | 5 µM BAM15 | 02:23:54 | 87.53 |
| 13 | 169 | E03 | 5 µM BAM15 | 02:24:08 | 78.68 |
| 13 | 170 | E03 | 5 µM BAM15 | 02:24:22 | 71.19 |
| 13 | 171 | E03 | 5 µM BAM15 | 02:24:36 | 64.42 |
| 13 | 172 | E03 | 5 µM BAM15 | 02:24:51 | 57.76 |
| 13 | 173 | E03 | 5 µM BAM15 | 02:25:07 | 51.31 |
| 13 | 174 | E03 | 5 µM BAM15 | 02:25:22 | 45.87 |
| 13 | 175 | E03 | 5 µM BAM15 | 02:25:36 | 40.97 |
| 13 | 176 | E03 | 5 µM BAM15 | 02:25:50 | 36.39 |
| 13 | 177 | E03 | 5 µM BAM15 | 02:26:04 | 32.63 |
| 13 | 178 | E03 | 5 µM BAM15 | 02:26:18 | 29.52 |
| 13 | 179 | E03 | 5 µM BAM15 | 02:26:33 | 27.13 |
| 14 | 180 | E03 | 5 µM BAM15 | 02:32:07 | 105.91 |
| 14 | 181 | E03 | 5 µM BAM15 | 02:32:21 | 90.43 |
| 14 | 182 | E03 | 5 µM BAM15 | 02:32:35 | 81.22 |
| 14 | 183 | E03 | 5 µM BAM15 | 02:32:50 | 73.61 |
| 14 | 184 | E03 | 5 µM BAM15 | 02:33:04 | 66.82 |
| 14 | 185 | E03 | 5 µM BAM15 | 02:33:18 | 60.44 |
| 14 | 186 | E03 | 5 µM BAM15 | 02:33:32 | 54.59 |
| 14 | 187 | E03 | 5 µM BAM15 | 02:33:46 | 48.99 |
| 14 | 188 | E03 | 5 µM BAM15 | 02:34:01 | 43.96 |
| 14 | 189 | E03 | 5 µM BAM15 | 02:34:15 | 39.22 |
| 14 | 190 | E03 | 5 µM BAM15 | 02:34:29 | 34.99 |
| 14 | 191 | E03 | 5 µM BAM15 | 02:34:43 | 31.45 |
| 14 | 192 | E03 | 5 µM BAM15 | 02:34:57 | 28.60 |
| 14 | 193 | E03 | 5 µM BAM15 | 02:35:12 | 26.54 |
| 15 | 194 | E03 | 5 µM BAM15 | 02:40:46 | 106.36 |
| 15 | 195 | E03 | 5 µM BAM15 | 02:41:00 | 91.17 |
| 15 | 196 | E03 | 5 µM BAM15 | 02:41:14 | 81.92 |
| 15 | 197 | E03 | 5 µM BAM15 | 02:41:28 | 74.38 |
| 15 | 198 | E03 | 5 µM BAM15 | 02:41:43 | 67.57 |
| 15 | 199 | E03 | 5 µM BAM15 | 02:41:57 | 61.31 |
| 15 | 200 | E03 | 5 µM BAM15 | 02:42:11 | 55.49 |
| 15 | 201 | E03 | 5 µM BAM15 | 02:42:25 | 49.97 |
| 15 | 202 | E03 | 5 µM BAM15 | 02:42:39 | 44.91 |
| 15 | 203 | E03 | 5 µM BAM15 | 02:42:53 | 40.18 |
| 15 | 204 | E03 | 5 µM BAM15 | 02:43:08 | 35.79 |
| 15 | 205 | E03 | 5 µM BAM15 | 02:43:22 | 32.25 |
| 15 | 206 | E03 | 5 µM BAM15 | 02:43:36 | 29.21 |
| 15 | 207 | E03 | 5 µM BAM15 | 02:43:50 | 26.97 |
| 16 | 208 | E03 | 5 µM BAM15 | 02:49:25 | 106.50 |
| 16 | 209 | E03 | 5 µM BAM15 | 02:49:40 | 91.39 |
| 16 | 210 | E03 | 5 µM BAM15 | 02:49:54 | 82.26 |
| 16 | 211 | E03 | 5 µM BAM15 | 02:50:08 | 74.71 |
| 16 | 212 | E03 | 5 µM BAM15 | 02:50:22 | 68.04 |
| 16 | 213 | E03 | 5 µM BAM15 | 02:50:36 | 61.78 |
| 16 | 214 | E03 | 5 µM BAM15 | 02:50:50 | 56.04 |
| 16 | 215 | E03 | 5 µM BAM15 | 02:51:05 | 50.56 |
| 16 | 216 | E03 | 5 µM BAM15 | 02:51:19 | 45.48 |
| 16 | 217 | E03 | 5 µM BAM15 | 02:51:33 | 40.78 |
| 16 | 218 | E03 | 5 µM BAM15 | 02:51:47 | 36.47 |
| 16 | 219 | E03 | 5 µM BAM15 | 02:52:01 | 32.82 |
| 16 | 220 | E03 | 5 µM BAM15 | 02:52:16 | 29.72 |
| 16 | 221 | E03 | 5 µM BAM15 | 02:52:30 | 27.33 |
| 1 | 0 | E04 | 5 µM FCCP | 00:40:06 | 126.32 |
| 1 | 1 | E04 | 5 µM FCCP | 00:40:20 | 119.89 |
| 1 | 2 | E04 | 5 µM FCCP | 00:40:34 | 117.14 |
| 1 | 3 | E04 | 5 µM FCCP | 00:40:48 | 115.05 |
| 1 | 4 | E04 | 5 µM FCCP | 00:41:02 | 113.28 |
| 1 | 5 | E04 | 5 µM FCCP | 00:41:17 | 111.63 |
| 1 | 6 | E04 | 5 µM FCCP | 00:41:31 | 110.10 |
| 1 | 7 | E04 | 5 µM FCCP | 00:41:45 | 108.70 |
| 1 | 8 | E04 | 5 µM FCCP | 00:41:59 | 107.31 |
| 1 | 9 | E04 | 5 µM FCCP | 00:42:13 | 106.12 |
| 1 | 10 | E04 | 5 µM FCCP | 00:42:28 | 104.81 |
| 1 | 11 | E04 | 5 µM FCCP | 00:42:42 | 103.69 |
| 1 | 12 | E04 | 5 µM FCCP | 00:42:56 | 102.53 |
| 1 | 13 | E04 | 5 µM FCCP | 00:43:10 | 101.50 |
| 2 | 14 | E04 | 5 µM FCCP | 00:48:43 | 127.40 |
| 2 | 15 | E04 | 5 µM FCCP | 00:48:57 | 121.57 |
| 2 | 16 | E04 | 5 µM FCCP | 00:49:12 | 118.84 |
| 2 | 17 | E04 | 5 µM FCCP | 00:49:26 | 116.71 |
| 2 | 18 | E04 | 5 µM FCCP | 00:49:40 | 114.89 |
| 2 | 19 | E04 | 5 µM FCCP | 00:49:54 | 113.14 |
| 2 | 20 | E04 | 5 µM FCCP | 00:50:08 | 111.62 |
| 2 | 21 | E04 | 5 µM FCCP | 00:50:22 | 110.07 |
| 2 | 22 | E04 | 5 µM FCCP | 00:50:37 | 108.76 |
| 2 | 23 | E04 | 5 µM FCCP | 00:50:51 | 107.45 |
| 2 | 24 | E04 | 5 µM FCCP | 00:51:05 | 106.14 |
| 2 | 25 | E04 | 5 µM FCCP | 00:51:19 | 104.97 |
| 2 | 26 | E04 | 5 µM FCCP | 00:51:33 | 103.76 |
| 2 | 27 | E04 | 5 µM FCCP | 00:51:48 | 102.73 |
| 3 | 28 | E04 | 5 µM FCCP | 00:57:21 | 128.98 |
| 3 | 29 | E04 | 5 µM FCCP | 00:57:35 | 124.12 |
| 3 | 30 | E04 | 5 µM FCCP | 00:57:49 | 121.47 |
| 3 | 31 | E04 | 5 µM FCCP | 00:58:03 | 119.38 |
| 3 | 32 | E04 | 5 µM FCCP | 00:58:17 | 117.53 |
| 3 | 33 | E04 | 5 µM FCCP | 00:58:31 | 115.83 |
| 3 | 34 | E04 | 5 µM FCCP | 00:58:46 | 114.22 |
| 3 | 35 | E04 | 5 µM FCCP | 00:59:00 | 112.77 |
| 3 | 36 | E04 | 5 µM FCCP | 00:59:14 | 111.21 |
| 3 | 37 | E04 | 5 µM FCCP | 00:59:28 | 109.95 |
| 3 | 38 | E04 | 5 µM FCCP | 00:59:42 | 108.66 |
| 3 | 39 | E04 | 5 µM FCCP | 00:59:56 | 107.46 |
| 3 | 40 | E04 | 5 µM FCCP | 01:00:11 | 106.28 |
| 3 | 41 | E04 | 5 µM FCCP | 01:00:25 | 105.16 |
| 4 | 42 | E04 | 5 µM FCCP | 01:06:04 | 130.26 |
| 4 | 43 | E04 | 5 µM FCCP | 01:06:18 | 124.50 |
| 4 | 44 | E04 | 5 µM FCCP | 01:06:33 | 121.39 |
| 4 | 45 | E04 | 5 µM FCCP | 01:06:47 | 119.03 |
| 4 | 46 | E04 | 5 µM FCCP | 01:07:01 | 116.96 |
| 4 | 47 | E04 | 5 µM FCCP | 01:07:15 | 114.87 |
| 4 | 48 | E04 | 5 µM FCCP | 01:07:29 | 112.98 |
| 4 | 49 | E04 | 5 µM FCCP | 01:07:43 | 111.18 |
| 4 | 50 | E04 | 5 µM FCCP | 01:07:58 | 109.47 |
| 4 | 51 | E04 | 5 µM FCCP | 01:08:12 | 107.71 |
| 4 | 52 | E04 | 5 µM FCCP | 01:08:26 | 105.94 |
| 4 | 53 | E04 | 5 µM FCCP | 01:08:40 | 104.26 |
| 4 | 54 | E04 | 5 µM FCCP | 01:08:54 | 102.47 |
| 4 | 55 | E04 | 5 µM FCCP | 01:09:09 | 100.75 |
| 5 | 56 | E04 | 5 µM FCCP | 01:14:42 | 128.21 |
| 5 | 57 | E04 | 5 µM FCCP | 01:14:56 | 121.49 |
| 5 | 58 | E04 | 5 µM FCCP | 01:15:10 | 118.06 |
| 5 | 59 | E04 | 5 µM FCCP | 01:15:25 | 115.41 |
| 5 | 60 | E04 | 5 µM FCCP | 01:15:39 | 112.99 |
| 5 | 61 | E04 | 5 µM FCCP | 01:15:53 | 110.66 |
| 5 | 62 | E04 | 5 µM FCCP | 01:16:07 | 108.53 |
| 5 | 63 | E04 | 5 µM FCCP | 01:16:21 | 106.37 |
| 5 | 64 | E04 | 5 µM FCCP | 01:16:35 | 104.35 |
| 5 | 65 | E04 | 5 µM FCCP | 01:16:50 | 102.22 |
| 5 | 66 | E04 | 5 µM FCCP | 01:17:04 | 100.13 |
| 5 | 67 | E04 | 5 µM FCCP | 01:17:18 | 98.01 |
| 5 | 68 | E04 | 5 µM FCCP | 01:17:32 | 95.89 |
| 5 | 69 | E04 | 5 µM FCCP | 01:17:46 | 93.75 |
| 6 | 70 | E04 | 5 µM FCCP | 01:23:20 | 127.19 |
| 6 | 71 | E04 | 5 µM FCCP | 01:23:34 | 120.16 |
| 6 | 72 | E04 | 5 µM FCCP | 01:23:48 | 116.61 |
| 6 | 73 | E04 | 5 µM FCCP | 01:24:02 | 113.75 |
| 6 | 74 | E04 | 5 µM FCCP | 01:24:16 | 111.27 |
| 6 | 75 | E04 | 5 µM FCCP | 01:24:30 | 108.84 |
| 6 | 76 | E04 | 5 µM FCCP | 01:24:44 | 106.65 |
| 6 | 77 | E04 | 5 µM FCCP | 01:24:59 | 104.29 |
| 6 | 78 | E04 | 5 µM FCCP | 01:25:13 | 102.14 |
| 6 | 79 | E04 | 5 µM FCCP | 01:25:27 | 99.93 |
| 6 | 80 | E04 | 5 µM FCCP | 01:25:41 | 97.74 |
| 6 | 81 | E04 | 5 µM FCCP | 01:25:55 | 95.59 |
| 6 | 82 | E04 | 5 µM FCCP | 01:26:10 | 93.39 |
| 6 | 83 | E04 | 5 µM FCCP | 01:26:24 | 91.16 |
| 7 | 84 | E04 | 5 µM FCCP | 01:31:58 | 126.66 |
| 7 | 85 | E04 | 5 µM FCCP | 01:32:12 | 119.61 |
| 7 | 86 | E04 | 5 µM FCCP | 01:32:26 | 115.99 |
| 7 | 87 | E04 | 5 µM FCCP | 01:32:40 | 113.19 |
| 7 | 88 | E04 | 5 µM FCCP | 01:32:54 | 110.57 |
| 7 | 89 | E04 | 5 µM FCCP | 01:33:08 | 108.14 |
| 7 | 90 | E04 | 5 µM FCCP | 01:33:23 | 105.82 |
| 7 | 91 | E04 | 5 µM FCCP | 01:33:37 | 103.56 |
| 7 | 92 | E04 | 5 µM FCCP | 01:33:51 | 101.39 |
| 7 | 93 | E04 | 5 µM FCCP | 01:34:05 | 99.19 |
| 7 | 94 | E04 | 5 µM FCCP | 01:34:19 | 96.99 |
| 7 | 95 | E04 | 5 µM FCCP | 01:34:33 | 94.88 |
| 7 | 96 | E04 | 5 µM FCCP | 01:34:48 | 92.71 |
| 7 | 97 | E04 | 5 µM FCCP | 01:35:02 | 90.54 |
| 8 | 98 | E04 | 5 µM FCCP | 01:40:36 | 126.64 |
| 8 | 99 | E04 | 5 µM FCCP | 01:40:50 | 119.57 |
| 8 | 100 | E04 | 5 µM FCCP | 01:41:04 | 115.97 |
| 8 | 101 | E04 | 5 µM FCCP | 01:41:18 | 113.11 |
| 8 | 102 | E04 | 5 µM FCCP | 01:41:32 | 110.53 |
| 8 | 103 | E04 | 5 µM FCCP | 01:41:46 | 108.16 |
| 8 | 104 | E04 | 5 µM FCCP | 01:42:00 | 105.87 |
| 8 | 105 | E04 | 5 µM FCCP | 01:42:15 | 103.61 |
| 8 | 106 | E04 | 5 µM FCCP | 01:42:29 | 101.38 |
| 8 | 107 | E04 | 5 µM FCCP | 01:42:43 | 99.28 |
| 8 | 108 | E04 | 5 µM FCCP | 01:42:57 | 97.13 |
| 8 | 109 | E04 | 5 µM FCCP | 01:43:11 | 94.98 |
| 8 | 110 | E04 | 5 µM FCCP | 01:43:26 | 92.79 |
| 8 | 111 | E04 | 5 µM FCCP | 01:43:40 | 90.72 |
| 9 | 112 | E04 | 5 µM FCCP | 01:49:14 | 126.85 |
| 9 | 113 | E04 | 5 µM FCCP | 01:49:28 | 119.94 |
| 9 | 114 | E04 | 5 µM FCCP | 01:49:42 | 116.31 |
| 9 | 115 | E04 | 5 µM FCCP | 01:49:56 | 113.48 |
| 9 | 116 | E04 | 5 µM FCCP | 01:50:10 | 110.93 |
| 9 | 117 | E04 | 5 µM FCCP | 01:50:24 | 108.50 |
| 9 | 118 | E04 | 5 µM FCCP | 01:50:38 | 106.23 |
| 9 | 119 | E04 | 5 µM FCCP | 01:50:53 | 103.96 |
| 9 | 120 | E04 | 5 µM FCCP | 01:51:07 | 101.82 |
| 9 | 121 | E04 | 5 µM FCCP | 01:51:21 | 99.65 |
| 9 | 122 | E04 | 5 µM FCCP | 01:51:35 | 97.54 |
| 9 | 123 | E04 | 5 µM FCCP | 01:51:50 | 95.43 |
| 9 | 124 | E04 | 5 µM FCCP | 01:52:04 | 93.31 |
| 9 | 125 | E04 | 5 µM FCCP | 01:52:18 | 91.19 |
| 10 | 126 | E04 | 5 µM FCCP | 01:57:52 | 127.24 |
| 10 | 127 | E04 | 5 µM FCCP | 01:58:06 | 120.41 |
| 10 | 128 | E04 | 5 µM FCCP | 01:58:20 | 116.81 |
| 10 | 129 | E04 | 5 µM FCCP | 01:58:34 | 114.04 |
| 10 | 130 | E04 | 5 µM FCCP | 01:58:48 | 111.51 |
| 10 | 131 | E04 | 5 µM FCCP | 01:59:03 | 109.02 |
| 10 | 132 | E04 | 5 µM FCCP | 01:59:17 | 106.75 |
| 10 | 133 | E04 | 5 µM FCCP | 01:59:31 | 104.53 |
| 10 | 134 | E04 | 5 µM FCCP | 01:59:45 | 102.48 |
| 10 | 135 | E04 | 5 µM FCCP | 01:59:59 | 100.29 |
| 10 | 136 | E04 | 5 µM FCCP | 02:00:14 | 98.19 |
| 10 | 137 | E04 | 5 µM FCCP | 02:00:28 | 96.10 |
| 10 | 138 | E04 | 5 µM FCCP | 02:00:42 | 93.97 |
| 10 | 139 | E04 | 5 µM FCCP | 02:00:56 | 91.92 |
| 11 | 140 | E04 | 5 µM FCCP | 02:06:30 | 127.43 |
| 11 | 141 | E04 | 5 µM FCCP | 02:06:44 | 120.84 |
| 11 | 142 | E04 | 5 µM FCCP | 02:06:58 | 117.24 |
| 11 | 143 | E04 | 5 µM FCCP | 02:07:12 | 114.41 |
| 11 | 144 | E04 | 5 µM FCCP | 02:07:26 | 111.94 |
| 11 | 145 | E04 | 5 µM FCCP | 02:07:41 | 109.58 |
| 11 | 146 | E04 | 5 µM FCCP | 02:07:55 | 107.31 |
| 11 | 147 | E04 | 5 µM FCCP | 02:08:09 | 105.12 |
| 11 | 148 | E04 | 5 µM FCCP | 02:08:23 | 102.99 |
| 11 | 149 | E04 | 5 µM FCCP | 02:08:37 | 100.85 |
| 11 | 150 | E04 | 5 µM FCCP | 02:08:52 | 98.82 |
| 11 | 151 | E04 | 5 µM FCCP | 02:09:06 | 96.67 |
| 11 | 152 | E04 | 5 µM FCCP | 02:09:20 | 94.64 |
| 11 | 153 | E04 | 5 µM FCCP | 02:09:34 | 92.50 |
| 12 | 154 | E04 | 5 µM FCCP | 02:15:08 | 128.31 |
| 12 | 155 | E04 | 5 µM FCCP | 02:15:22 | 122.47 |
| 12 | 156 | E04 | 5 µM FCCP | 02:15:37 | 119.04 |
| 12 | 157 | E04 | 5 µM FCCP | 02:15:53 | 116.14 |
| 12 | 158 | E04 | 5 µM FCCP | 02:16:08 | 113.72 |
| 12 | 159 | E04 | 5 µM FCCP | 02:16:22 | 111.59 |
| 12 | 160 | E04 | 5 µM FCCP | 02:16:36 | 109.38 |
| 12 | 161 | E04 | 5 µM FCCP | 02:16:50 | 107.23 |
| 12 | 162 | E04 | 5 µM FCCP | 02:17:04 | 105.17 |
| 12 | 163 | E04 | 5 µM FCCP | 02:17:18 | 103.08 |
| 12 | 164 | E04 | 5 µM FCCP | 02:17:33 | 101.03 |
| 12 | 165 | E04 | 5 µM FCCP | 02:17:47 | 99.01 |
| 12 | 166 | E04 | 5 µM FCCP | 02:18:01 | 96.91 |
| 13 | 167 | E04 | 5 µM FCCP | 02:23:39 | 127.58 |
| 13 | 168 | E04 | 5 µM FCCP | 02:23:54 | 123.10 |
| 13 | 169 | E04 | 5 µM FCCP | 02:24:08 | 120.16 |
| 13 | 170 | E04 | 5 µM FCCP | 02:24:22 | 117.66 |
| 13 | 171 | E04 | 5 µM FCCP | 02:24:36 | 115.49 |
| 13 | 172 | E04 | 5 µM FCCP | 02:24:51 | 113.18 |
| 13 | 173 | E04 | 5 µM FCCP | 02:25:07 | 110.83 |
| 13 | 174 | E04 | 5 µM FCCP | 02:25:22 | 108.67 |
| 13 | 175 | E04 | 5 µM FCCP | 02:25:36 | 106.69 |
| 13 | 176 | E04 | 5 µM FCCP | 02:25:50 | 104.65 |
| 13 | 177 | E04 | 5 µM FCCP | 02:26:04 | 102.68 |
| 13 | 178 | E04 | 5 µM FCCP | 02:26:18 | 100.72 |
| 13 | 179 | E04 | 5 µM FCCP | 02:26:33 | 98.66 |
| 14 | 180 | E04 | 5 µM FCCP | 02:32:07 | 130.01 |
| 14 | 181 | E04 | 5 µM FCCP | 02:32:21 | 124.69 |
| 14 | 182 | E04 | 5 µM FCCP | 02:32:35 | 121.71 |
| 14 | 183 | E04 | 5 µM FCCP | 02:32:50 | 119.19 |
| 14 | 184 | E04 | 5 µM FCCP | 02:33:04 | 116.93 |
| 14 | 185 | E04 | 5 µM FCCP | 02:33:18 | 114.78 |
| 14 | 186 | E04 | 5 µM FCCP | 02:33:32 | 112.68 |
| 14 | 187 | E04 | 5 µM FCCP | 02:33:46 | 110.65 |
| 14 | 188 | E04 | 5 µM FCCP | 02:34:01 | 108.63 |
| 14 | 189 | E04 | 5 µM FCCP | 02:34:15 | 106.65 |
| 14 | 190 | E04 | 5 µM FCCP | 02:34:29 | 104.72 |
| 14 | 191 | E04 | 5 µM FCCP | 02:34:43 | 102.70 |
| 14 | 192 | E04 | 5 µM FCCP | 02:34:57 | 100.67 |
| 14 | 193 | E04 | 5 µM FCCP | 02:35:12 | 98.79 |
| 15 | 194 | E04 | 5 µM FCCP | 02:40:46 | 130.39 |
| 15 | 195 | E04 | 5 µM FCCP | 02:41:00 | 125.26 |
| 15 | 196 | E04 | 5 µM FCCP | 02:41:14 | 122.26 |
| 15 | 197 | E04 | 5 µM FCCP | 02:41:28 | 119.75 |
| 15 | 198 | E04 | 5 µM FCCP | 02:41:43 | 117.48 |
| 15 | 199 | E04 | 5 µM FCCP | 02:41:57 | 115.41 |
| 15 | 200 | E04 | 5 µM FCCP | 02:42:11 | 113.31 |
| 15 | 201 | E04 | 5 µM FCCP | 02:42:25 | 111.34 |
| 15 | 202 | E04 | 5 µM FCCP | 02:42:39 | 109.31 |
| 15 | 203 | E04 | 5 µM FCCP | 02:42:53 | 107.32 |
| 15 | 204 | E04 | 5 µM FCCP | 02:43:08 | 105.33 |
| 15 | 205 | E04 | 5 µM FCCP | 02:43:22 | 103.43 |
| 15 | 206 | E04 | 5 µM FCCP | 02:43:36 | 101.41 |
| 15 | 207 | E04 | 5 µM FCCP | 02:43:50 | 99.49 |
| 16 | 208 | E04 | 5 µM FCCP | 02:49:25 | 130.67 |
| 16 | 209 | E04 | 5 µM FCCP | 02:49:40 | 125.62 |
| 16 | 210 | E04 | 5 µM FCCP | 02:49:54 | 122.65 |
| 16 | 211 | E04 | 5 µM FCCP | 02:50:08 | 120.25 |
| 16 | 212 | E04 | 5 µM FCCP | 02:50:22 | 117.97 |
| 16 | 213 | E04 | 5 µM FCCP | 02:50:36 | 115.80 |
| 16 | 214 | E04 | 5 µM FCCP | 02:50:50 | 113.77 |
| 16 | 215 | E04 | 5 µM FCCP | 02:51:05 | 111.74 |
| 16 | 216 | E04 | 5 µM FCCP | 02:51:19 | 109.70 |
| 16 | 217 | E04 | 5 µM FCCP | 02:51:33 | 107.80 |
| 16 | 218 | E04 | 5 µM FCCP | 02:51:47 | 105.89 |
| 16 | 219 | E04 | 5 µM FCCP | 02:52:01 | 103.94 |
| 16 | 220 | E04 | 5 µM FCCP | 02:52:16 | 101.97 |
| 16 | 221 | E04 | 5 µM FCCP | 02:52:30 | 100.04 |
| 1 | 0 | E05 | 5 µM FCCP | 00:40:06 | 129.65 |
| 1 | 1 | E05 | 5 µM FCCP | 00:40:20 | 123.03 |
| 1 | 2 | E05 | 5 µM FCCP | 00:40:34 | 120.37 |
| 1 | 3 | E05 | 5 µM FCCP | 00:40:48 | 118.35 |
| 1 | 4 | E05 | 5 µM FCCP | 00:41:02 | 116.65 |
| 1 | 5 | E05 | 5 µM FCCP | 00:41:17 | 115.17 |
| 1 | 6 | E05 | 5 µM FCCP | 00:41:31 | 113.76 |
| 1 | 7 | E05 | 5 µM FCCP | 00:41:45 | 112.51 |
| 1 | 8 | E05 | 5 µM FCCP | 00:41:59 | 111.23 |
| 1 | 9 | E05 | 5 µM FCCP | 00:42:13 | 110.05 |
| 1 | 10 | E05 | 5 µM FCCP | 00:42:28 | 108.86 |
| 1 | 11 | E05 | 5 µM FCCP | 00:42:42 | 107.76 |
| 1 | 12 | E05 | 5 µM FCCP | 00:42:56 | 106.74 |
| 1 | 13 | E05 | 5 µM FCCP | 00:43:10 | 105.75 |
| 2 | 14 | E05 | 5 µM FCCP | 00:48:43 | 132.01 |
| 2 | 15 | E05 | 5 µM FCCP | 00:48:57 | 126.17 |
| 2 | 16 | E05 | 5 µM FCCP | 00:49:12 | 123.57 |
| 2 | 17 | E05 | 5 µM FCCP | 00:49:26 | 121.41 |
| 2 | 18 | E05 | 5 µM FCCP | 00:49:40 | 119.69 |
| 2 | 19 | E05 | 5 µM FCCP | 00:49:54 | 117.97 |
| 2 | 20 | E05 | 5 µM FCCP | 00:50:08 | 116.44 |
| 2 | 21 | E05 | 5 µM FCCP | 00:50:22 | 115.08 |
| 2 | 22 | E05 | 5 µM FCCP | 00:50:37 | 113.73 |
| 2 | 23 | E05 | 5 µM FCCP | 00:50:51 | 112.51 |
| 2 | 24 | E05 | 5 µM FCCP | 00:51:05 | 111.30 |
| 2 | 25 | E05 | 5 µM FCCP | 00:51:19 | 110.12 |
| 2 | 26 | E05 | 5 µM FCCP | 00:51:33 | 108.93 |
| 2 | 27 | E05 | 5 µM FCCP | 00:51:48 | 107.92 |
| 3 | 28 | E05 | 5 µM FCCP | 00:57:21 | 133.38 |
| 3 | 29 | E05 | 5 µM FCCP | 00:57:35 | 128.15 |
| 3 | 30 | E05 | 5 µM FCCP | 00:57:49 | 125.47 |
| 3 | 31 | E05 | 5 µM FCCP | 00:58:03 | 123.37 |
| 3 | 32 | E05 | 5 µM FCCP | 00:58:17 | 121.62 |
| 3 | 33 | E05 | 5 µM FCCP | 00:58:31 | 119.91 |
| 3 | 34 | E05 | 5 µM FCCP | 00:58:46 | 118.38 |
| 3 | 35 | E05 | 5 µM FCCP | 00:59:00 | 116.91 |
| 3 | 36 | E05 | 5 µM FCCP | 00:59:14 | 115.58 |
| 3 | 37 | E05 | 5 µM FCCP | 00:59:28 | 114.30 |
| 3 | 38 | E05 | 5 µM FCCP | 00:59:42 | 113.04 |
| 3 | 39 | E05 | 5 µM FCCP | 00:59:56 | 111.89 |
| 3 | 40 | E05 | 5 µM FCCP | 01:00:11 | 110.75 |
| 3 | 41 | E05 | 5 µM FCCP | 01:00:25 | 109.73 |
| 4 | 42 | E05 | 5 µM FCCP | 01:06:04 | 133.14 |
| 4 | 43 | E05 | 5 µM FCCP | 01:06:18 | 127.40 |
| 4 | 44 | E05 | 5 µM FCCP | 01:06:33 | 124.64 |
| 4 | 45 | E05 | 5 µM FCCP | 01:06:47 | 122.33 |
| 4 | 46 | E05 | 5 µM FCCP | 01:07:01 | 120.40 |
| 4 | 47 | E05 | 5 µM FCCP | 01:07:15 | 118.61 |
| 4 | 48 | E05 | 5 µM FCCP | 01:07:29 | 116.88 |
| 4 | 49 | E05 | 5 µM FCCP | 01:07:43 | 115.26 |
| 4 | 50 | E05 | 5 µM FCCP | 01:07:58 | 113.66 |
| 4 | 51 | E05 | 5 µM FCCP | 01:08:12 | 112.08 |
| 4 | 52 | E05 | 5 µM FCCP | 01:08:26 | 110.48 |
| 4 | 53 | E05 | 5 µM FCCP | 01:08:40 | 108.98 |
| 4 | 54 | E05 | 5 µM FCCP | 01:08:54 | 107.42 |
| 4 | 55 | E05 | 5 µM FCCP | 01:09:09 | 105.92 |
| 5 | 56 | E05 | 5 µM FCCP | 01:14:42 | 131.23 |
| 5 | 57 | E05 | 5 µM FCCP | 01:14:56 | 124.52 |
| 5 | 58 | E05 | 5 µM FCCP | 01:15:10 | 121.32 |
| 5 | 59 | E05 | 5 µM FCCP | 01:15:25 | 118.80 |
| 5 | 60 | E05 | 5 µM FCCP | 01:15:39 | 116.51 |
| 5 | 61 | E05 | 5 µM FCCP | 01:15:53 | 114.46 |
| 5 | 62 | E05 | 5 µM FCCP | 01:16:07 | 112.48 |
| 5 | 63 | E05 | 5 µM FCCP | 01:16:21 | 110.54 |
| 5 | 64 | E05 | 5 µM FCCP | 01:16:35 | 108.63 |
| 5 | 65 | E05 | 5 µM FCCP | 01:16:50 | 106.75 |
| 5 | 66 | E05 | 5 µM FCCP | 01:17:04 | 104.91 |
| 5 | 67 | E05 | 5 µM FCCP | 01:17:18 | 103.01 |
| 5 | 68 | E05 | 5 µM FCCP | 01:17:32 | 101.14 |
| 5 | 69 | E05 | 5 µM FCCP | 01:17:46 | 99.26 |
| 6 | 70 | E05 | 5 µM FCCP | 01:23:20 | 130.05 |
| 6 | 71 | E05 | 5 µM FCCP | 01:23:34 | 122.94 |
| 6 | 72 | E05 | 5 µM FCCP | 01:23:48 | 119.61 |
| 6 | 73 | E05 | 5 µM FCCP | 01:24:02 | 116.95 |
| 6 | 74 | E05 | 5 µM FCCP | 01:24:16 | 114.61 |
| 6 | 75 | E05 | 5 µM FCCP | 01:24:30 | 112.39 |
| 6 | 76 | E05 | 5 µM FCCP | 01:24:44 | 110.29 |
| 6 | 77 | E05 | 5 µM FCCP | 01:24:59 | 108.21 |
| 6 | 78 | E05 | 5 µM FCCP | 01:25:13 | 106.24 |
| 6 | 79 | E05 | 5 µM FCCP | 01:25:27 | 104.28 |
| 6 | 80 | E05 | 5 µM FCCP | 01:25:41 | 102.35 |
| 6 | 81 | E05 | 5 µM FCCP | 01:25:55 | 100.34 |
| 6 | 82 | E05 | 5 µM FCCP | 01:26:10 | 98.38 |
| 6 | 83 | E05 | 5 µM FCCP | 01:26:24 | 96.34 |
| 7 | 84 | E05 | 5 µM FCCP | 01:31:58 | 129.29 |
| 7 | 85 | E05 | 5 µM FCCP | 01:32:12 | 122.20 |
| 7 | 86 | E05 | 5 µM FCCP | 01:32:26 | 118.73 |
| 7 | 87 | E05 | 5 µM FCCP | 01:32:40 | 116.13 |
| 7 | 88 | E05 | 5 µM FCCP | 01:32:54 | 113.65 |
| 7 | 89 | E05 | 5 µM FCCP | 01:33:08 | 111.53 |
| 7 | 90 | E05 | 5 µM FCCP | 01:33:23 | 109.38 |
| 7 | 91 | E05 | 5 µM FCCP | 01:33:37 | 107.32 |
| 7 | 92 | E05 | 5 µM FCCP | 01:33:51 | 105.24 |
| 7 | 93 | E05 | 5 µM FCCP | 01:34:05 | 103.31 |
| 7 | 94 | E05 | 5 µM FCCP | 01:34:19 | 101.32 |
| 7 | 95 | E05 | 5 µM FCCP | 01:34:33 | 99.44 |
| 7 | 96 | E05 | 5 µM FCCP | 01:34:48 | 97.56 |
| 7 | 97 | E05 | 5 µM FCCP | 01:35:02 | 95.54 |
| 8 | 98 | E05 | 5 µM FCCP | 01:40:36 | 129.24 |
| 8 | 99 | E05 | 5 µM FCCP | 01:40:50 | 122.21 |
| 8 | 100 | E05 | 5 µM FCCP | 01:41:04 | 118.79 |
| 8 | 101 | E05 | 5 µM FCCP | 01:41:18 | 116.04 |
| 8 | 102 | E05 | 5 µM FCCP | 01:41:32 | 113.75 |
| 8 | 103 | E05 | 5 µM FCCP | 01:41:46 | 111.52 |
| 8 | 104 | E05 | 5 µM FCCP | 01:42:00 | 109.39 |
| 8 | 105 | E05 | 5 µM FCCP | 01:42:15 | 107.44 |
| 8 | 106 | E05 | 5 µM FCCP | 01:42:29 | 105.37 |
| 8 | 107 | E05 | 5 µM FCCP | 01:42:43 | 103.42 |
| 8 | 108 | E05 | 5 µM FCCP | 01:42:57 | 101.50 |
| 8 | 109 | E05 | 5 µM FCCP | 01:43:11 | 99.61 |
| 8 | 110 | E05 | 5 µM FCCP | 01:43:26 | 97.71 |
| 8 | 111 | E05 | 5 µM FCCP | 01:43:40 | 95.85 |
| 9 | 112 | E05 | 5 µM FCCP | 01:49:14 | 129.28 |
| 9 | 113 | E05 | 5 µM FCCP | 01:49:28 | 122.38 |
| 9 | 114 | E05 | 5 µM FCCP | 01:49:42 | 119.03 |
| 9 | 115 | E05 | 5 µM FCCP | 01:49:56 | 116.39 |
| 9 | 116 | E05 | 5 µM FCCP | 01:50:10 | 114.07 |
| 9 | 117 | E05 | 5 µM FCCP | 01:50:24 | 111.88 |
| 9 | 118 | E05 | 5 µM FCCP | 01:50:38 | 109.82 |
| 9 | 119 | E05 | 5 µM FCCP | 01:50:53 | 107.79 |
| 9 | 120 | E05 | 5 µM FCCP | 01:51:07 | 105.84 |
| 9 | 121 | E05 | 5 µM FCCP | 01:51:21 | 103.95 |
| 9 | 122 | E05 | 5 µM FCCP | 01:51:35 | 102.11 |
| 9 | 123 | E05 | 5 µM FCCP | 01:51:50 | 100.28 |
| 9 | 124 | E05 | 5 µM FCCP | 01:52:04 | 98.43 |
| 9 | 125 | E05 | 5 µM FCCP | 01:52:18 | 96.58 |
| 10 | 126 | E05 | 5 µM FCCP | 01:57:52 | 129.62 |
| 10 | 127 | E05 | 5 µM FCCP | 01:58:06 | 122.86 |
| 10 | 128 | E05 | 5 µM FCCP | 01:58:20 | 119.53 |
| 10 | 129 | E05 | 5 µM FCCP | 01:58:34 | 116.93 |
| 10 | 130 | E05 | 5 µM FCCP | 01:58:48 | 114.65 |
| 10 | 131 | E05 | 5 µM FCCP | 01:59:03 | 112.51 |
| 10 | 132 | E05 | 5 µM FCCP | 01:59:17 | 110.39 |
| 10 | 133 | E05 | 5 µM FCCP | 01:59:31 | 108.41 |
| 10 | 134 | E05 | 5 µM FCCP | 01:59:45 | 106.55 |
| 10 | 135 | E05 | 5 µM FCCP | 01:59:59 | 104.67 |
| 10 | 136 | E05 | 5 µM FCCP | 02:00:14 | 102.81 |
| 10 | 137 | E05 | 5 µM FCCP | 02:00:28 | 101.01 |
| 10 | 138 | E05 | 5 µM FCCP | 02:00:42 | 99.22 |
| 10 | 139 | E05 | 5 µM FCCP | 02:00:56 | 97.42 |
| 11 | 140 | E05 | 5 µM FCCP | 02:06:30 | 130.02 |
| 11 | 141 | E05 | 5 µM FCCP | 02:06:44 | 123.41 |
| 11 | 142 | E05 | 5 µM FCCP | 02:06:58 | 120.06 |
| 11 | 143 | E05 | 5 µM FCCP | 02:07:12 | 117.47 |
| 11 | 144 | E05 | 5 µM FCCP | 02:07:26 | 115.27 |
| 11 | 145 | E05 | 5 µM FCCP | 02:07:41 | 113.07 |
| 11 | 146 | E05 | 5 µM FCCP | 02:07:55 | 111.07 |
| 11 | 147 | E05 | 5 µM FCCP | 02:08:09 | 109.13 |
| 11 | 148 | E05 | 5 µM FCCP | 02:08:23 | 107.36 |
| 11 | 149 | E05 | 5 µM FCCP | 02:08:37 | 105.53 |
| 11 | 150 | E05 | 5 µM FCCP | 02:08:52 | 103.71 |
| 11 | 151 | E05 | 5 µM FCCP | 02:09:06 | 101.88 |
| 11 | 152 | E05 | 5 µM FCCP | 02:09:20 | 100.15 |
| 11 | 153 | E05 | 5 µM FCCP | 02:09:34 | 98.44 |
| 12 | 154 | E05 | 5 µM FCCP | 02:15:08 | 130.27 |
| 12 | 155 | E05 | 5 µM FCCP | 02:15:22 | 124.07 |
| 12 | 156 | E05 | 5 µM FCCP | 02:15:37 | 120.74 |
| 12 | 157 | E05 | 5 µM FCCP | 02:15:53 | 118.00 |
| 12 | 158 | E05 | 5 µM FCCP | 02:16:08 | 115.79 |
| 12 | 159 | E05 | 5 µM FCCP | 02:16:22 | 113.75 |
| 12 | 160 | E05 | 5 µM FCCP | 02:16:36 | 111.89 |
| 12 | 161 | E05 | 5 µM FCCP | 02:16:50 | 110.02 |
| 12 | 162 | E05 | 5 µM FCCP | 02:17:04 | 108.10 |
| 12 | 163 | E05 | 5 µM FCCP | 02:17:18 | 106.37 |
| 12 | 164 | E05 | 5 µM FCCP | 02:17:33 | 104.61 |
| 12 | 165 | E05 | 5 µM FCCP | 02:17:47 | 102.84 |
| 12 | 166 | E05 | 5 µM FCCP | 02:18:01 | 101.16 |
| 13 | 167 | E05 | 5 µM FCCP | 02:23:39 | 129.87 |
| 13 | 168 | E05 | 5 µM FCCP | 02:23:54 | 125.29 |
| 13 | 169 | E05 | 5 µM FCCP | 02:24:08 | 122.56 |
| 13 | 170 | E05 | 5 µM FCCP | 02:24:22 | 120.18 |
| 13 | 171 | E05 | 5 µM FCCP | 02:24:36 | 118.11 |
| 13 | 172 | E05 | 5 µM FCCP | 02:24:51 | 115.95 |
| 13 | 173 | E05 | 5 µM FCCP | 02:25:07 | 113.88 |
| 13 | 174 | E05 | 5 µM FCCP | 02:25:22 | 112.05 |
| 13 | 175 | E05 | 5 µM FCCP | 02:25:36 | 110.32 |
| 13 | 176 | E05 | 5 µM FCCP | 02:25:50 | 108.59 |
| 13 | 177 | E05 | 5 µM FCCP | 02:26:04 | 106.92 |
| 13 | 178 | E05 | 5 µM FCCP | 02:26:18 | 105.20 |
| 13 | 179 | E05 | 5 µM FCCP | 02:26:33 | 103.48 |
| 14 | 180 | E05 | 5 µM FCCP | 02:32:07 | 132.51 |
| 14 | 181 | E05 | 5 µM FCCP | 02:32:21 | 127.15 |
| 14 | 182 | E05 | 5 µM FCCP | 02:32:35 | 124.39 |
| 14 | 183 | E05 | 5 µM FCCP | 02:32:50 | 122.02 |
| 14 | 184 | E05 | 5 µM FCCP | 02:33:04 | 120.00 |
| 14 | 185 | E05 | 5 µM FCCP | 02:33:18 | 118.07 |
| 14 | 186 | E05 | 5 µM FCCP | 02:33:32 | 116.14 |
| 14 | 187 | E05 | 5 µM FCCP | 02:33:46 | 114.32 |
| 14 | 188 | E05 | 5 µM FCCP | 02:34:01 | 112.57 |
| 14 | 189 | E05 | 5 µM FCCP | 02:34:15 | 110.92 |
| 14 | 190 | E05 | 5 µM FCCP | 02:34:29 | 109.21 |
| 14 | 191 | E05 | 5 µM FCCP | 02:34:43 | 107.49 |
| 14 | 192 | E05 | 5 µM FCCP | 02:34:57 | 105.86 |
| 14 | 193 | E05 | 5 µM FCCP | 02:35:12 | 104.25 |
| 15 | 194 | E05 | 5 µM FCCP | 02:40:46 | 132.97 |
| 15 | 195 | E05 | 5 µM FCCP | 02:41:00 | 127.88 |
| 15 | 196 | E05 | 5 µM FCCP | 02:41:14 | 125.04 |
| 15 | 197 | E05 | 5 µM FCCP | 02:41:28 | 122.85 |
| 15 | 198 | E05 | 5 µM FCCP | 02:41:43 | 120.67 |
| 15 | 199 | E05 | 5 µM FCCP | 02:41:57 | 118.81 |
| 15 | 200 | E05 | 5 µM FCCP | 02:42:11 | 117.01 |
| 15 | 201 | E05 | 5 µM FCCP | 02:42:25 | 115.24 |
| 15 | 202 | E05 | 5 µM FCCP | 02:42:39 | 113.49 |
| 15 | 203 | E05 | 5 µM FCCP | 02:42:53 | 111.83 |
| 15 | 204 | E05 | 5 µM FCCP | 02:43:08 | 110.09 |
| 15 | 205 | E05 | 5 µM FCCP | 02:43:22 | 108.49 |
| 15 | 206 | E05 | 5 µM FCCP | 02:43:36 | 106.77 |
| 15 | 207 | E05 | 5 µM FCCP | 02:43:50 | 105.17 |
| 16 | 208 | E05 | 5 µM FCCP | 02:49:25 | 133.25 |
| 16 | 209 | E05 | 5 µM FCCP | 02:49:40 | 128.35 |
| 16 | 210 | E05 | 5 µM FCCP | 02:49:54 | 125.61 |
| 16 | 211 | E05 | 5 µM FCCP | 02:50:08 | 123.31 |
| 16 | 212 | E05 | 5 µM FCCP | 02:50:22 | 121.31 |
| 16 | 213 | E05 | 5 µM FCCP | 02:50:36 | 119.38 |
| 16 | 214 | E05 | 5 µM FCCP | 02:50:50 | 117.53 |
| 16 | 215 | E05 | 5 µM FCCP | 02:51:05 | 115.81 |
| 16 | 216 | E05 | 5 µM FCCP | 02:51:19 | 114.08 |
| 16 | 217 | E05 | 5 µM FCCP | 02:51:33 | 112.43 |
| 16 | 218 | E05 | 5 µM FCCP | 02:51:47 | 110.81 |
| 16 | 219 | E05 | 5 µM FCCP | 02:52:01 | 109.22 |
| 16 | 220 | E05 | 5 µM FCCP | 02:52:16 | 107.56 |
| 16 | 221 | E05 | 5 µM FCCP | 02:52:30 | 105.94 |
| 1 | 0 | E06 | 5 µM FCCP | 00:40:06 | 127.23 |
| 1 | 1 | E06 | 5 µM FCCP | 00:40:20 | 121.12 |
| 1 | 2 | E06 | 5 µM FCCP | 00:40:34 | 118.48 |
| 1 | 3 | E06 | 5 µM FCCP | 00:40:48 | 116.42 |
| 1 | 4 | E06 | 5 µM FCCP | 00:41:02 | 114.69 |
| 1 | 5 | E06 | 5 µM FCCP | 00:41:17 | 113.10 |
| 1 | 6 | E06 | 5 µM FCCP | 00:41:31 | 111.64 |
| 1 | 7 | E06 | 5 µM FCCP | 00:41:45 | 110.29 |
[truncated: 78,736 more chars]
